# The effect of aldafermin expressing-*Escherichia coli* Nissle 1917 along with dietary change on visceral adipose tissue in MASLD mouse model

**DOI:** 10.1038/s41366-025-01774-w

**Published:** 2025-04-10

**Authors:** Valeria Iannone, Maija Vaittinen, Carlos Gómez-Gallego, Santtu Mikkonen, Johnson Lok, Giuseppe D’Auria, Ruben Vazquez-Uribe, Ida Tikkanen, Morten Otto Alexander Sommer, Hani El-Nezami, Marjukka Kolehmainen

**Affiliations:** 1 School of Medicine, Institute of Public Health and Clinical Nutrition, University of Eastern Finland, Kuopio, Finland; 2 Department of Environmental and Biological Sciences, University of Eastern Finland, Kuopio, Finland; 3 Sequencing and Bioinformatics Service, Foundation for the Promotion of Health and Biomedical Research of Valencia Region, FISABIO, Valencia, Spain; 4 CIBER in Epidemiology and Public Health (CIBERESP), Instituto de Salud Carlos III, Madrid, Spain; 5 The Novo Nordisk Foundation Center for Biosustainability, Technical University of Denmark, Kongens Lyngby, Denmark; 6 Molecular and Cell Biology Research Area, School of Biological Sciences, University of Hong Kong, Hong Kong SAR, Hong Kong, China

**Keywords:** Metabolic syndrome, Mouse, Preclinical research

## Abstract

**Background:**

Visceral adipose tissue (VAT) accumulation in obesity has been implicated as a key factor in the development of metabolic dysfunction-associated steatotic liver disease (MASLD). Apart from lifestyle change interventions, there is no effective therapy against MASLD. In this study, the effect of a novel microbial therapy along with dietary change on VAT and VAT-liver crosstalk was evaluated in a MASLD mouse model.

**Methods:**

MASLD was induced by feeding eighteen C57BL/6J male mice with the American Lifestyle-Induced Obesity diet for fourteen weeks. Subsequently, during the following seven weeks, all mice were switched to standard diet and the intervention group received single gelatine cubes containing 10^9^ CFU each of aldafermin-expressing *Escherichia coli* Nissle (EcNA, *n* = 6); while the control groups received either 10^9^ CFU/gelatine cube of non-modified *Escherichia coli* Nissle (EcN, *n* = 6) or gelatin cube with no treatment (CTRL, *n* = 6). The effect of EcNA on epididymal visceral adipose tissue (eVAT) morphology was evaluated by histology and the gene expression profile in eVAT and liver by RNA-sequencing analysis.

**Results:**

After seven weeks of intervention, EcNA, when compared to CTRL group, induced smaller adipocytes (*p*-value = 0.0217 for diameter, *p*-value = 0.0386 for area). Gene Set Enrichment Analysis in eVAT showed significant upregulation of fatty acid metabolism (FDR-adjusted *p*-value = 0.001), oxidative phosphorylation (FDR-adjusted *p*-value < 2.2e-16), peroxisome (FDR-adjusted *p*-value = 0.0185), and thermogenesis (FDR-adjusted *p*-value = 0.0199) pathways when EcNA was compared to EcN group. In addition, the impact of EcNA in eVAT-liver gene expression crosstalk was underlined by the upregulation of *Bcl6* and *Cnst* expression in both tissues when EcNA was compared to CTRL and EcN groups.

**Conclusions:**

These results support the beneficial effects of EcNA, along with dietary change intervention, in obesity-associated MASLD. This microbial therapy could potentially boost the improvements induced by dietary change in eVAT metabolism and its crosstalk with the liver.

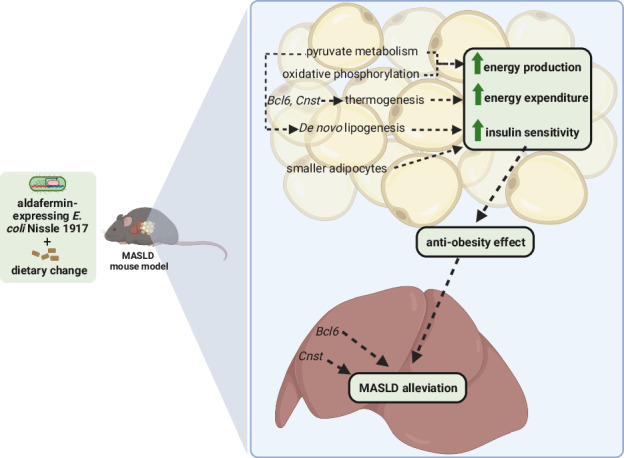

## Introduction

Obesity represents the major risk factor for the development of metabolic dysfunction-associated steatotic liver disease (MASLD), formerly known as non-alcoholic fatty liver disease [1]. While excess fat deposition in both subcutaneous adipose tissue (SAT) and visceral adipose tissue (VAT) has been associated with MASLD [2], visceral adiposity in obesity has been implicated as a key factor in the development of MASLD [1, 3–5]. This might be attributed to the fact that VAT is more metabolically active and susceptible to lipolysis and being insulin resistant than SAT [6]. VAT expansion has also been shown to contribute to hepatic steatosis in patients with type 2 diabetes [7]. Decreased mitochondrial respiration in VAT associated with reduced insulin sensitivity has been observed in patients with MASLD and obesity [8]. In addition, visceral adipocyte hypertrophy (increased adipocyte size) was found associated with the severity of liver histopathology in patients with MASLD [9]. Indeed, hypertrophic adipocytes exhibit insulin resistance (IR) [10], which results in increased lipolytic activity, promoting the release of free fatty acids (FAs) [11]. Free FAs can accumulate in the liver contributing to MASLD pathogenesis [3].

There is no approved pharmacological therapy to MASLD. Instead lifestyle modifications are recommended for managing the disease. However, the long-term efficacy of these interventions is often limited due to a lack of adherence [12]. New pharmacological interventions have targeted bile acid metabolism as a therapeutic approach for MASLD [13] Fibroblast growth factor 19 (FGF19) is a key regulator of bile acid and glucose metabolism in the liver [13] also involved in white adipose tissue browning, which subsequently increasing energy expenditure and protects against obesity [14, 15]. These dual functions of FGF19 highlight its potential as a therapeutic target for addressing both hepatic and adipose tissue dysfunction in MASLD. Interestingly, patients with MASLD exhibit lower levels of plasma FGF19 [16]. In this context, aldafermin, a non-tumorigenic analog of FGF19, has been identified as a promising target for the resolution of hepatic steatosis in patients with MASLD [17]. We have previously demonstrated [18] reduction in hepatic steatosis, body weight and expression of genes and metabolites enriched in pathways associated with oxidative stress and IR in response to a microbial therapy intervention utilizing *Escherichia (E.) coli* Nissle 1917 expressing aldafermin, along with dietary changes, in MASLD mice [18].

Considering the beneficial effects of aldafermin against MASLD and its potential as a FGF19 analog to modulate adipose tissue and the liver metabolism, we hypothesized that aldafermin-producing *E. coli* Nissle 1917 could improve VAT metabolism and modulate VAT-liver crosstalk, therefore alleviating MASLD.

## Methods

All methods were performed in accordance with the relevant guidelines and regulations.

### *E. coli* Nissle 1917 strains preparation in gelatine cubes

A pMUT-1-based plasmid native to *E. coli* Nissle 1917 was engineered to constitutively express aldafermin ensuring a steady and continuous production of aldafermin [18]. The preparation of gelatine cubes was performed as previously described [18]. Briefly, *E. coli* Nissle strains (with/without aldafermin expression) were cultured overnight in Luria-Bertani (LB) broth and plated onto LB agar plates. The next day, the strains were collected in a sweetener solution and mixed with a gelatine solution in a 96-well plate. The concentration of 10^9^ colony-forming unit (CFU) per gelatine cube of the bacteria was confirmed by plating onto LB agar plates.

### Animal study

The use of mouse model was approved by the National Ethics Committee for Animal Experiments in Finland (license number: ESAVI/21371/2019). Five- to eight-weeks-old C57BL/6 J male mice were bred and housed in animal facilities at the University of Eastern Finland (Kuopio, Finland). Eighteen mice were fed with the American Lifestyle-Induced Obesity (ALIOS) diet (TD06303, Harlan Teklad, Madison, WI) diet for MASLD induction. After fourteen weeks, all mice were switched to the standard diet (2016, Harlan Teklad, Madison, WI) and randomly separated into three groups for seven weeks. During these seven weeks, mice individually consumed daily gelatine cubes containing 10^9^ CFU each of *E. coli* Nissle expressing aldafermin (EcNA, n = 6), 10^9^ CFU each of *E. coli* Nissle without aldafermin expression (EcN, *n* = 6), and gelatine cubes without any bacteria or aldafermin (CTRL, *n* = 6). Additional information regarding the animal study (sample size, husbandry and intervention) is available in Supplementary data. After the intervention, terminal anesthesia with pentobarbital (Orion Pharma, Finland) and cardiac perfusion were performed. The liver and visceral epididymal adipose tissue (eVAT) were collected.

### Histology

The eVAT samples from all mice were fixed overnight in 4% paraformaldehyde (Sigma-Aldrich, Germany). Tissues were embedded into paraffin blocks and cut into 5 µm sections. For H&E staining, one image at 4× and six images each at 10× and 20× objectives were acquired. Fiji (ImageJ) software (version 1.52p) and Adiposoft automated open-source [19] were used for the quantification of area and diameter of adipocytes in eVAT sections. Adipocyte areas and diameters were automatically calculated based upon the pixel and unit information provided by the users (0.1722 pixels per μm for all images) and recorded in an Excel file. Parameters for minimum and maximum diameters were set as follows: minimum = 10, maximum = 250, images and tissue artefacts that were not entirely in the image were excluded, and adipocytes that were not clearly defined were manually adjusted with the option “Adiposoft manually”.

### RNA-sequencing

The liver and eVAT tissue from all mice samples were collected and immediately submerged in RNA stabilization solution (Qiagen, Germany), frozen in liquid nitrogen, and stored at −80 °C. The liver was homogenized with Buffer RLT (Qiagen, Germany), and RNA was extracted with RNeasy mini kit (Qiagen, Germany). eVAT was homogenized in QIAzol Lysis Reagent (Qiagen, Germany), and RNeasy Lipid Tissue Mini Kit (Qiagen, Germany) was used for RNA extraction. The RNase-free DNase I (Qiagen, Germany) treatment was performed in both sample tissues. Integrity and quantification of RNA were evaluated with RNA Nano 6000 Assay Kit of the Bioanalyzer 2100 system (Agilent Technologies, CA, USA). Five mice in each group were selected for transcriptome sequencing analysis based on the RNA purity (OD260/280 ≥ 2.0, OD260/230 ≥ 1.8) and quality (RIN ≥ 8.60). Library preparation, and sequencing using illumina platform Novaseq 6000 were performed as previously described [18].

### Statistical analysis

The differences in adipocyte area and diameter between the groups were tested using Kruskal–Wallis test with Games-Howell post-hoc tests by using R software version 3.6.3. Graphics were done using GraphPad Prism (version 9.2.0). This test was used because the normality assumption required for parametric testing was not fulfilled [20]. As severe heterogeneity can increase the risk of type I error in Kruskal-Wallis test [21], a sensitivity test for the analyses with Welch’ Anova test [22], was performed to confirm the results (Supplementary data). The effect size for the diameter and area of adipocytes was calculated using eta-squared (η²[H]) with rstatix [23] R studio package by using R software version 3.6.3. and reported in Supplementary Table 2.

Genes were pre-filtered, excluding those with zero or low counts (the sum of all counts was less than 10) to avoid including uncertain low counts in further gene analysis [24, 25]. Gene Set Enrichment Analysis (GSEA) using KEGG functional database was performed with WEB-based GEne SeT AnaLysis Toolkit [21] (details are available in Supplementary data). Venn diagrams were created using Venny [22]. Principal component analysis was conducted with ExpressAnalyst [23] (version 1.0-2023). Volcano plots of fold changes and FDR-adjusted p-values for the transcriptomic data were created using VolcaNoseR [24].

Spearman’s correlation analysis using the Fragments Per Kilobase of transcript per Million mapped reads (FPKM) values of differentially expressed genes (DEGs) (FDR-adjusted *p*-value < 0.05) overlapping between the liver and eVAT in EcNA compared to CTRL and in EcNA compared to EcN were performed using GraphPad Prism (version 9.2.0). Heatmaps with complete cluster method and correlation distance method were performed by https://www.bioinformatics.com.cn/en.

## Results

### EcNA intervention showed smaller adipocytes size in eVAT

To investigate whether E. *coli* Nissle 1917 expressing aldafermin in combination with dietary changes (EcNA) intervention could accelerate MASLD amelioration in a MASLD mouse model and whether the effects observed were specifically induced by aldafermin, the intervention group EcNA, was compared to control groups that either received dietary changes alone (CTRL) or E. *coli* Nissle 1917 without aldafermin expression (EcN).

**Fig. 1:**
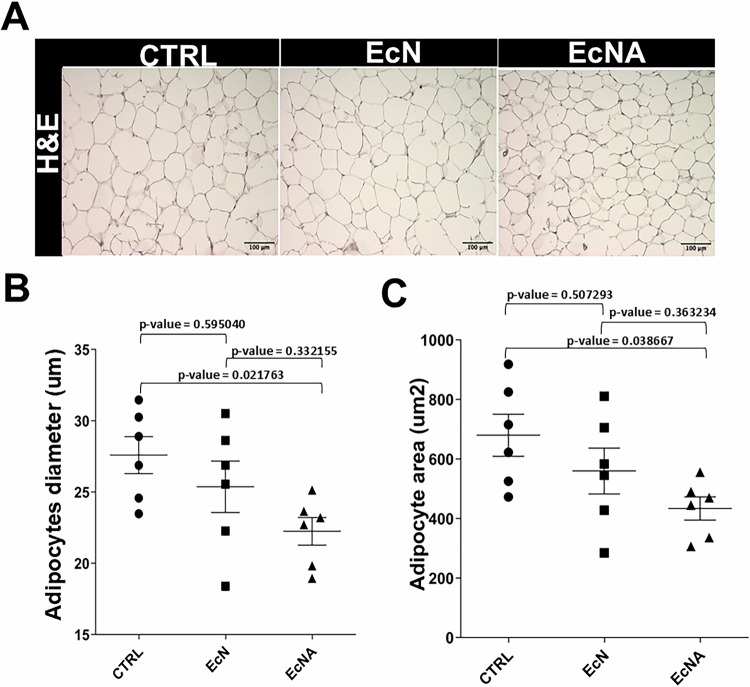
EcNA intervention reduced the size of adipocytes in eVAT. **A** Representative picture of H&E staining after seven weeks of intervention, *n* = 6 mice/group, scale bar 100 µm, 10× objective. Quantification of adipocyte by using Adiposoft [19] **B** diameter (µm) and **C** area (µm^2^), *n* = 6 mice/group, 6 acquisitions/mouse. Chart results were presented as average values, error bars denote SD. eVAT epididymal adipose tissue; H&E Haematoxylin and Eosin.

To investigate whether EcNA intervention could modify the size of adipocytes of eVAT, morphological analysis were performed. The mean diameter of adipocytes was significantly smaller in EcNA group (22.24 ± 2.38 µm, Mean ± SD) compared to CTRL group (27.59 ± 3.17 µm, Mean ± SD) (*p*-value = 0.0217). Similarly, the mean of the adipocyte area was significantly smaller in the EcNA group (433.78 ± 95.24 µm²) than in CTRL group (679.81 ± 172.92 µm²) (*p*-value = 0.038) (Fig. 1A, B, C, Supplementary Table 1). A trend toward a smaller diameter and area was also observed in EcNA group compared to EcN group (25.37 ± 4.42 µm, 559.57 ± 188.86 µm², Mean ± SD), though the differences were not statistically significant (*p*-value = 0.3321 for diameter, *p*-value = 0.3632 for area) (Fig. 1A, B, C, Supplementary Table 1). The effect size was 0.232 for diameter and 0.244 for area, both indicating a large effect (Supplementary Table 2). These results supported our earlier findings [18] showing a consistent body weight reduction, hepatic, plasma levels of aspartate aminotransferase (AST), and total cholesterol in EcNA intervention group when compared with control groups (Supplementary Tables 1 and 2).

### EcNA intervention exhibited altered transcript profiles in eVAT and the liver

To identify the effects of the intervention on gene expression profiles in eVAT and liver tissue, transcriptomics analysis was performed on the intervention group EcNA, as well as on the control groups, EcN and CTRL. Group differences in the transcript profiles were observed in both eVAT (Supplementary Figure 1A) and liver (Supplementary Figure 1B) tissues using principal component analysis, between the EcNA and EcN groups, and between the EcNA and CTRL groups, suggesting that the EcNA intervention induced changes in gene expression profile in both tissues when compared to both control groups.

**Fig. 2:**
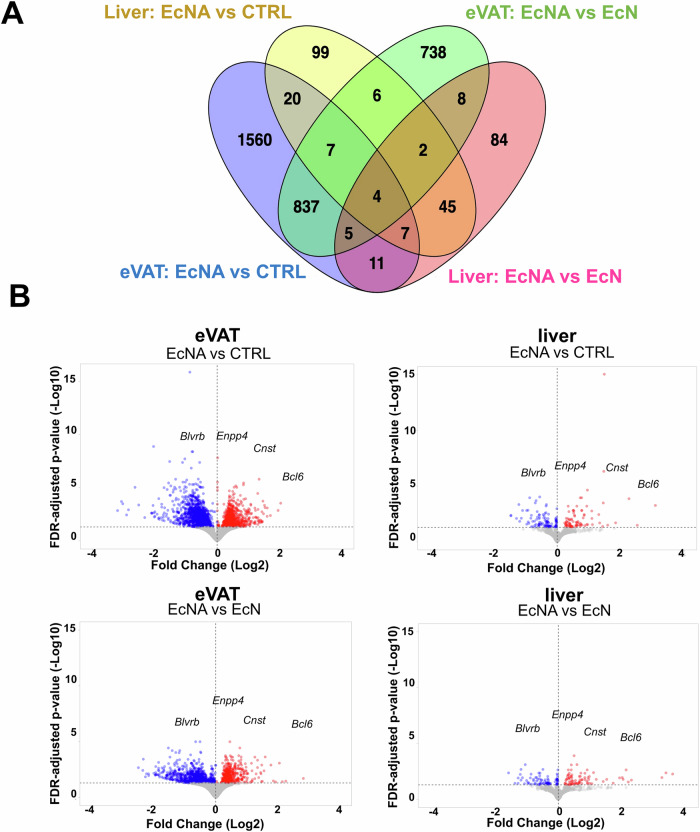
eVAT and liver gene expression change in EcNA intervention group. **A** Venn diagram showing the number of DEGs and **B** Volcano plot showing the proportion of up- (in red) and downregulated (in blue) genes with FDR-adjusted *p*-value < 0.05 in EcNA compared to either CTRL or EcN in both, in eVAT and the liver. eVAT epididymal visceral adipose tissue; DEGs differentially expressed genes.

Transcript profiles in eVAT and liver tissue after EcNA intervention were compared to EcN and CTRL groups (Supplementary Table 3, Supplementary Table 4). Transcriptomic analysis in eVAT showed 2451 DEGs in EcNA compared to CTRL (Fig. 2A), of which 942 were upregulated and 1509 were downregulated (Fig. 2B). There were 1607 DEGs found in EcNA when compared to EcN (Fig. 2A), of which 711 were upregulated and 896 were downregulated (Fig. 2B). In addition, 417 upregulated and 63 downregulated DEGs were observed in EcN compared to CTRL, highlighting the host impacts of the *E. coli* Nissle in eVAT (Supplementary Figs. 2A, 4, Supplementary Table 5).

In the liver tissue samples, 190 DEGs were observed in EcNA compared to CTRL (Fig. 2A, Supplementary Table 6) of which 83 genes were upregulated and 107 were downregulated (Fig. 2B). There were 166 DEGs observed in EcNA compared to EcN (Fig. 2A, Supplementary Table 7), of which 81 were upregulated and 85 were downregulated (Fig. 2B). Moreover, 8 upregulated DEGs were observed in EcN compared to CTRL, highlighting the mild host impacts of the *E. coli* Nissle 1917 in the liver tissue (Supplementary Fig. 2A, Supplementary Table 8).

The DEGs that were shared in both comparisons and in both tissues were Ectonucleotide Pyrophosphatase/Phosphodiesterase 4 (*Enpp4)*, Biliverdin Reductase B *(Blvrb)*, B-Cell Lymphoma 6 Protein *(Bcl6)* and Consortin (*Cnst)* (Fig. 2A, B). While *Enpp4, Bcl6*, and *Cnst* were upregulated, *Blvrb* was downregulated.

**Fig. 3:**
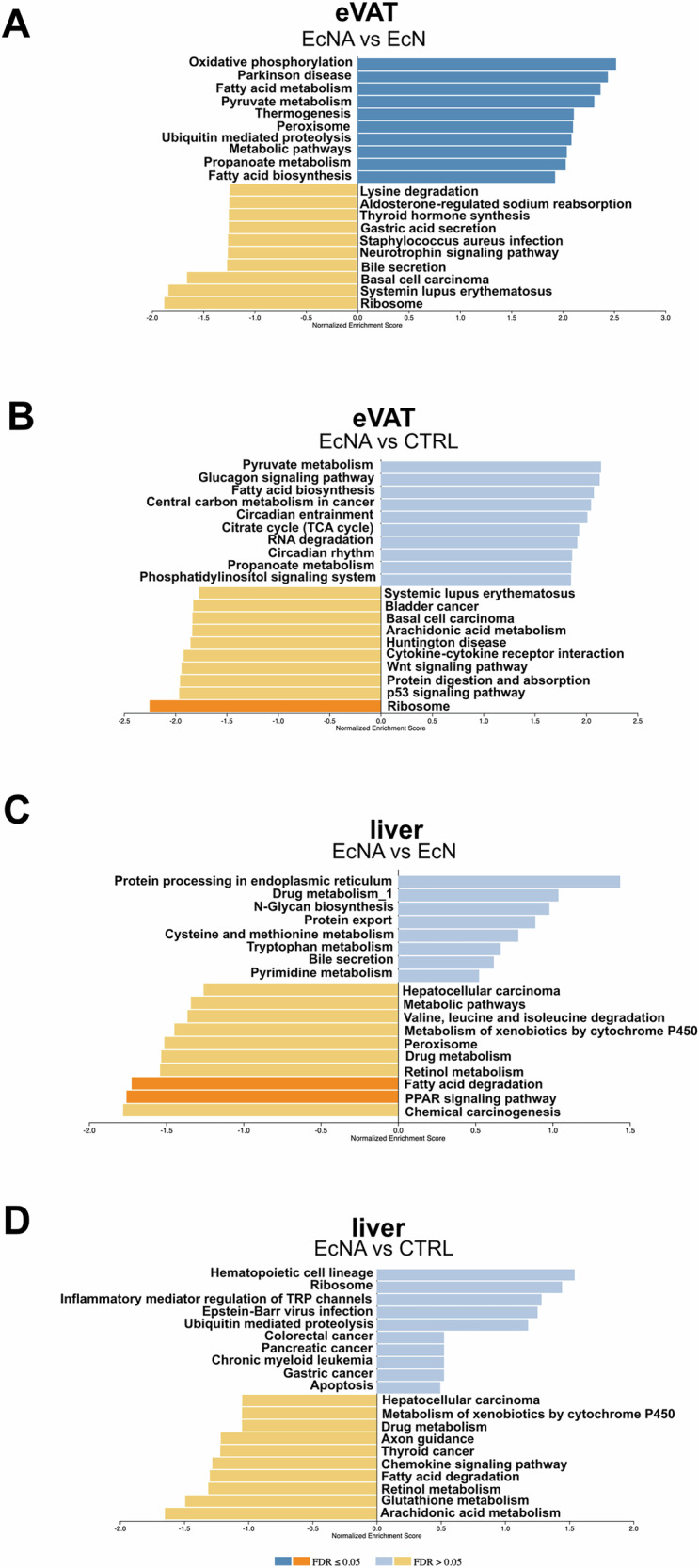
EcNA intervention induced changes in gene expression pathways in eVAT and liver. Bar plots showing the upregulated (in blue) and downregulated (in orange) pathways in **A** EcNA vs EcN in eVAT, **B** EcNA vs CTRL in eVAT, **C** EcNA vs EcN in the liver and **D** EcNA vs CTRL in the liver. Pathways with FDR-adjusted *p*-value < 0.05 were considered significant. eVAT: epididymal visceral adipose tissue. Details of the pathways can be found in Supplementary Table 9.

GSEA was performed on DEGs in eVAT and liver tissue to identify enriched pathways when comparing EcNA to EcN and CTRL (Fig. 3A, B, C, D).

In eVAT, the top ten upregulated pathways in EcNA compared to EcN were: oxidative phosphorylation, Parkinson's disease, FAs and pyruvate metabolism, thermogenesis, peroxisome, ubiquitin-mediated proteolysis, metabolic pathways, propanoate metabolism, and FAs biosynthesis (Fig. 3A, Supplementary table 9). Furthermore, a significant (FDR-adjusted *p*-value < 0.05) downregulation of transcripts enriched in the ribosome pathway was observed in EcNA when compared to CTRL (Fig. 3B, Supplementary Table 9). In addition, significant downregulation (FDR-adjusted *p*-value < 0.05) of complement and coagulation cascades and tyrosine metabolism was found in EcN compared to CTRL (Supplementary Fig. 2B, Supplementary Table 9).

In the liver, significant (FDR-adjusted *p*-value < 0.05) downregulation of transcripts enriched in FA degradation and peroxisome proliferator-activated receptor (Ppar) signaling pathway were observed in the EcNA group compared to the EcN group (Fig. 3C). However, no significantly enriched pathways were observed in the comparison between EcNA and CTRL groups (Fig. 3D). Moreover, when EcN was compared to CTRL, no up- or downregulated pathways were observed. These results suggest that the intervention had a stronger effect on eVAT than the liver metabolism and was observed to impact tissue metabolism at the gene expression level differently from the control groups.

**Fig. 4:**
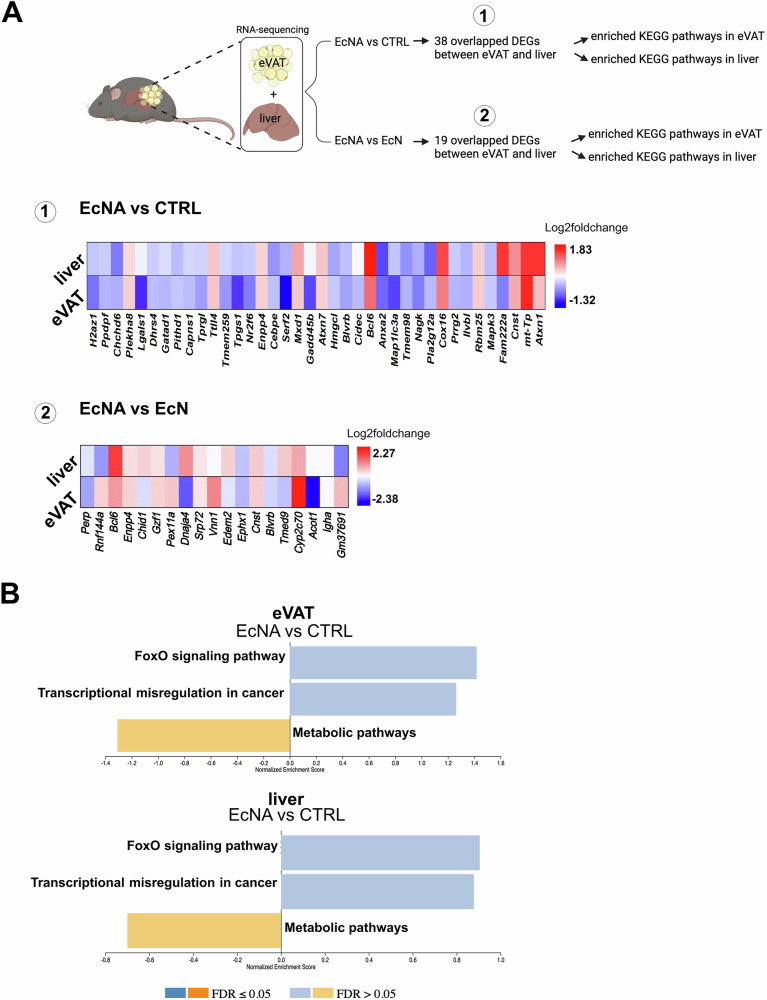
EcNA effect on shared gene expression patterns between eVAT and liver. **A** Heatmaps showing the log2foldchange values (positive in red and negative in blue) of the overlapped DEGs with FDR-adjusted *p*-value < 0.05 between eVAT and the liver in EcNA compared to EcN or CTRL groups. **B** Bar plots showing the upregulated (in blue) and downregulated (in orange) pathways in EcNA vs CTRL in both eVAT and the liver. Pathways with FDR-adjusted *p*-value < 0.05 were considered significant. eVAT epididymal visceral adipose tissue; DEGs differentially expressed genes. Details of the pathways can be found in Supplementary Table 9.

To explore the effect of the intervention on shared gene expression patterns between eVAT and the liver, DEGs shared between eVAT and the liver were selected for GSEA. There were 38 DEGs shared between eVAT and the liver when EcNA was compared to CTRL (Fig. 4A), and 19 DEGs shared between eVAT and the liver when EcNA was compared to EcN (Fig. 4A). However, GSEA showed non-significant changes in pathways when EcNA was compared to EcN and CTRL (Fig. 4B).

To examine the tissue-specific effect of EcNA on gene expression, DEGs found exclusively in either eVAT or the liver when EcNA was compared to EcN or CTRL groups were selected and GSEA was performed. This analysis aimed to identify pathways associated with the identified DEGs in each tissue comparison separately. Many of the pathways identified in both eVAT and liver tissue when comparing EcNA to EcN and CTRL (Supplementary Fig. 3A, B, C, D, E) matched with pathways identified previously based on the DEGs shared in eVAT and liver tissue (Fig. 3A, B, C, D), confirming the higher efficacy of EcNA in eVAT than the liver as well as in EcNA compared to EcN or CTRL group.

### Correlation analysis suggested possible eVAT-liver crosstalk in response to EcNA

**Fig. 5:**
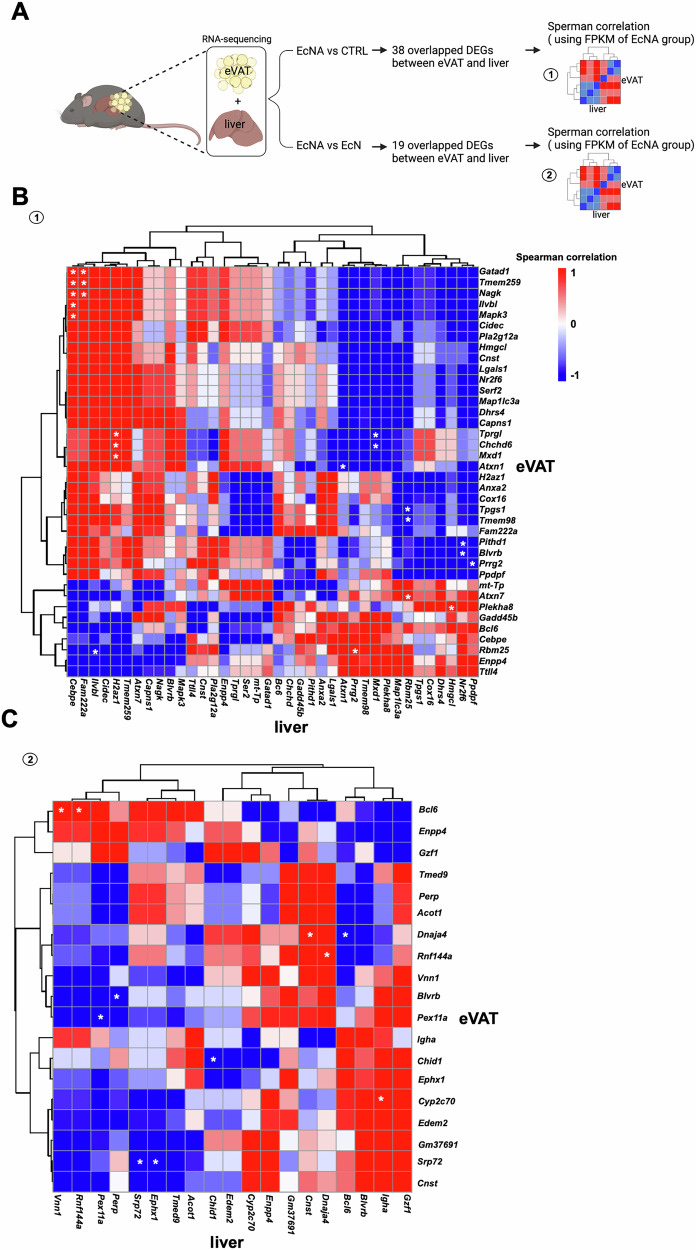
Correlation analysis showing the eVAT-liver gene expression crosstalk in EcNA intervention group. **A** Diagram showing the analysis approach for Spearman correlation analysis. **B** heatmap showing the Spearman correlation analysis done using the FPKM of the 38 overlapped DEGs in both eVAT and liver in EcNA compared to CTRL. **C** Heatmap showing the Spearman correlation analysis done using the FPKM of the 19 overlapped DEGs in both eVAT and liver in EcNA compared to EcN. Positive correlation in red and negative correlation in blue. White asterisks identified notable correlations. FPKM Fragments Per Kilobase per Million mapped fragments; DEGs differentially expressed genes; eVAT epididymal visceral adipose tissue.

To determine the effect of the intervention on eVAT-liver gene expression crosstalk, Spearman’s rank correlation and cluster analysis were performed on the 38 (EcNA vs. CTRL) and 19 (EcNA vs. EcN) DEGs shared in eVAT and liver tissue. Furthermore, the FPKM values of the aforementioned 38 and 19 DEGs were computed and correlated (Fig. 5A).

The heatmap generated using the FPKM values from the 38 DEGs shared between eVAT and liver tissue (EcNA vs. CTRL) showed distinct clusters and several notable correlations (Fig. 5B), suggesting interactions between the gene expression patterns in eVAT and the liver.

The heatmap generated using the FPKM values of the 19 DEGs shared between eVAT and liver tissue (EcNA vs EcN) also revealed distinct clusters and notable correlations (Fig. 5C).

To ensure that the significant correlations of the shared DEGs in eVAT and liver tissue observed in the EcNA intervention group were specifically attributed to the EcNA effect, correlation analyses of the shared DEGs in eVAT and liver tissue were also performed for the CTRL and EcN groups (Supplementary Fig. 4). Any notable correlations between the DEGs in eVAT and liver tissue from either the CTRL or EcN group that overlapped with those observed in the EcNA intervention group were discarded as non-specific to the EcNA intervention, ensuring that only correlations unique to the EcNA effect were considered.

## Discussion

In this study, we describe the potential beneficial effect of using aldafermin expressing *E. coli* Nissle 1917 in conjunction with dietary change (EcNA), on eVAT in the MASLD mouse model. An anti-obesity effect on eVAT was shown after EcNA intervention based on gene expression in both eVAT and liver and histological observations in eVAT. Transcriptomics analyses in eVAT suggested potential amelioration of the energy metabolism. Additionally, the enrichment of de novo lipogenesis-associated genes may indicate improved insulin sensitivity which was further supported by the decreased size of adipocytes observed by histological findings. These fundings are supported by the demonstrated capability of EcNA intervention to alleviate MASLD in mice, with reductions in body weight, hepatic steatosis, and plasma markers associated with liver functions observed, while also possibly ameliorating oxidative stress and insulin resistance in the liver of the MASLD mouse model [18].

VAT, in addition to the liver, has been suggested as a therapeutic target for treatment of MASLD [1, 3]. In our study, the EcNA intervention, when compared to EcN group, showed consistently to influence eVAT metabolism at the gene expression level. Specifically, genes associated with FA synthesis and elongation were upregulated, such as Acetyl-CoA Carboxylase Beta *(Acacab)*, Acetyl-CoA Carboxylase Alpha *(Acaca)*, Acyl-CoA Synthetase Long-Chain Family Member 1 (*Acsl1*), ELOVL fatty acid elongase *(Elovl)6* and hydroxysteroid 17-beta dehydrogenase *(H*sd17b)12, suggesting an increase in de novo lipogenesis. Of note, de novo lipogenesis has been shown to be positively correlated with smaller adipocytes and increased insulin sensitivity [26]. Thus, de novo lipogenesis in adipose tissue is important for maintaining systemic insulin sensitivity.

In addition, upregulation of the pyruvate metabolism pathway was found, including increased expression of Pyruvate Dehydrogenase E1 Subunit Alpha 1 *(Pdha1)*, Pyruvate Dehydrogenase E1 Subunit Beta (*Pdhb)* and Dihydrolipoamide S-Acetyltransferase *(Dlat)*. Those genes regulate the conversion of pyruvate to acetyl-coenzyme A in mitochondria [27], driving energy production via oxidative phosphorylation but also providing substrate for de novo lipogenesis [28]. Therefore, our results may support the possible role of EcNA intervention in enhancement of de novo lipogenesis, likely *via* increased pyruvate metabolism.

We observed upregulated pathways for oxidative phosphorylation and thermogenesis in eVAT after EcNA intervention when compared to EcN, supporting the potential beneficial effect of EcNA on the enhancement of mitochondrial function in eVAT. Decreased mitochondrial respiration has been observed in the VAT of patients with obesity and MASLD, and it has been correlated with impaired insulin sensitivity [8]. Moreover, the upregulation of the thermogenesis pathway observed in our study could be attributed to the capability of FGF19 to induce an anti-obesity effect on adipose tissue, via stimulation of white adipose tissue browning [14, 15]. This was further supported by the observed upregulation of Klotho Beta (*Klb*) expression, which encodes for the FGF19 obligatory coreceptor [29]. FGF19 stimulates thermogenesis by inducing the upregulation of Uncoupling Protein 1 (*Ucp1)* [14, 15]. In our study, *Ucp1* was found to be upregulated, although non-statistically significant (p-value 0.9267). However, the expression of another thermogenic gene, activating transcription factor 2 (*Atf2)*, which is known to stimulate *Ucp1* expression [30], was found to be upregulated. Furthermore, the downregulation of cyclin-dependent kinase Inhibitor 2A (*Cdkn2a)* supports the notion of a suggested increase in thermogenesis in response to EcNA. *Cdkn2a-*deficient mice have been previously observed to have increased energy expenditure, *Ucp1* expression, improved IR, and a reduction in body weight [31]. Interestingly, *Cdkn2a* expression is increased in adipose tissue of patients with obesity [31].

Collectively, these results suggest that the EcNA intervention may promote metabolic homeostasis in eVAT when compared to EcN, possibly by improving insulin sensitivity and stimulating energy production and expenditure, thereby exerting an anti-obesity effect in eVAT in the MASLD setting. These findings are further supported by the reduced body weight observed in mice after EcNA intervention [18].

The interplay between VAT and the liver is intricate, and VAT dysfunctions have been shown to contribute to MASLD pathogenesis [1, 3]. In our study, the transcriptomic changes observed in eVAT after the EcNA intervention compared to EcN may demonstrate a direct beneficial effect on liver metabolism through crosstalk between eVAT and the liver. Downregulation of FA degradation and Ppar signaling pathways was observed in the EcNA intervention group compared to EcN in the liver. Specifically, Solute Carrier Family 27 Member 1 (S*lc27a1*) involved in FA uptake [32], *Pparγ* stimulating FA storage as triacylglycerol [33], and the acetyl-Coenzyme A acyltransferase 1B (*Acaa1b*) involved in FA beta-oxidation [34] were downregulated. The downregulation of these genes may be explained by reduced free FA release from eVAT. As previously reported [18], the integration of transcriptomics and metabolomics showed lower steatosis and downregulation of b-oxidization pathway in the liver of mice after the EcNA intervention.

Pathway analysis conducted on both eVAT and liver tissue did not reveal significant differences between the EcNA and CTRL groups, except for a downregulation of the ribosome pathway in eVAT. However, eVAT and liver histological findings support the notion of beneficial effects of EcNA intervention when compared also to the CTRL group. Indeed, the absence of hepatic steatosis in response to the EcNA intervention when compared to EcN and CTRL groups was observed [18]. In addition, this was further supported by our previous work, combining metabolomics with transcriptomics analysis revealing significant changes in liver-related pathways when EcNA was compared to CTRL. This suggests that the combination of techniques enhances the power of the results.

Furthermore, the size of adipocytes in eVAT in the EcNA group was significantly smaller than that of the CTRL group in this study. Small adipocytes are associated with increased insulin sensitivity [26], while hypertrophic adipocytes with IR [10]. Smaller adipocytes in eVAT, coupled with potentially increased insulin sensitivity, lend credence to the idea that the EcNA intervention could alleviate MASLD via eVAT-liver crosstalk. This aspect should be investigated in future studies.

EcNA exerted a significant influence on expression of *Bcl6* and *Cnst*. In eVAT and liver the transcription factor BCL6 has been implicated in thermogenesis in brown adipocytes [35]. Mice lacking BCL6 and under cold stimulus showed reduced oxidative phosphorylation capacity in brown adipocytes [35]. Instead mice, without exposure to cold and impaired of BCL6, showed reduced thermogenic capacity in white adipose tissue [36]. In addition, IR and hepatic steatosis were observed to improve in mice fed with high fat diet (HFD) that overexpressed BCL6 protein levels, while MASLD conditions were aggravated in those mice where BCL6 protein levels were downregulated [37]. BCL6 protein levels have been found to be decreased in the liver of patients with MASLD [37]. On the contrary, it has been reported that in mice fed HFD, *Bcl6* knockout in the liver attenuated the hepatic steatosis [38]. The discrepancies observed in the different studies may be due to experimental design and methods adopted or different hepatic liver disease stages. Our results suggest a potential role of *Bcl6* in supporting an enhanced thermogenic effect in eVAT, alleviating steatosis and associated IR in the liver, in response to EcNA intervention.

*Cnst* gene codes for a protein called consortin, which acts as a trans-Golgi network receptor involved in connexin recycling [39]. Connexins are components of gap junctions and are important factors in intercellular communication [39]. To the best of our knowledge, the function of *Cnst* is not fully characterized yet however, the disruption of gap junctions has previously been described in the liver of MASLD murine models contributing to the disease progression [40, 41]. Connexins have also been shown to play an important role in adipose tissue as well, with the downregulation of connexin previously observed to be associated with reduced thermogenic capacity in mice under cold stimulus [42],. Therefore, it is plausible that the upregulation of *Cnst*, in response to the EcNA intervention, may be associated with enhanced intercellular communication in both eVAT and liver. However, the mechanism remains to be elucidated.

Crosstalk between eVAT and the liver was supported by findings from the correlation analysis of gene expression. In EcNA, when compared to EcN, the upregulation of Peroxisomal Biogenesis Factor 11 Alpha (*Pex11a*) in eVAT and its downregulation in the liver were negatively correlated. *Pex11a* plays a key role in peroxisome proliferation [43] which is involved in the oxidation of FAs [44]. We observed an upregulation of the peroxisome pathway in eVAT, which may support increased FAs oxidation for energy production.

EcNA, when compared to CTRL, highlighted genes that could suggest an anti-obesity effect. The downregulation of *Mapk3*, is concordant with a previous study in which mice lacking *Mapk3* showed less adiposity and did not develop obesity or IR with an HFD [45]. We also found Nuclear Receptor Subfamily 2 Group F Member (*Nr2f)6* to be downregulated. Overexpression of *Nr2f6* mice has been observed to induce hepatic steatosis in MASLD mouse model, whereas suppression of *Nr2f6* was observed to improve IR and hepatic steatosis [46].

Our results suggest that there are potential benefits to utilize the microbial therapy along with dietary changes in obesity-associated MASLD. EcNA intervention induces an anti-obesity effect on eVAT, possibly by stimulating energy production, and expenditure and by improving insulin sensitivity in MASLD mouse model. This beneficial effect observed in eVAT could contribute to the observed alleviation of MASLD in the liver described in our previous work [18]. Our study needs additional research to confirm the findings based on the gene expression levels, and to thoroughly characterize the molecular mechanisms underlying EcNA action.

## Supplementary information

SUPPLEMENTAL MATERIAL

Supplementary table 3

Supplementary table 4

Supplementary table 5

Supplementary table 6

Supplementary table 7

Supplementary table 8

Supplementary table 9

## Data Availability

RNA-sequencing data are deposited in NCBI’s [47] (GSE232030).
