# Supplementary material for: The effect of aldafermin expressing-Escherichia coli Nissle 1917 along with dietary change on visceral adipose tissue in MASLD mouse model
Source: Int J Obes (Lond). 2025 Apr 10;49(7):1334–44. doi: 10.1038/s41366-025-01774-w (PMC12283412; doi:10.1038/s41366-025-01774-w)
Supplement: Supplementary file 1 — SUPPLEMENTAL MATERIAL [file 41366_2025_1774_MOESM1_ESM.docx]

Supplementary Material

# Supplementary Data

**Animal study design:**

All mice housed in the animal facility were under pathogen-free conditions in single ventilated cages under a 12/12h light cycle at 20.6°C (± 0.2°C) with 55%(± 0.8%) humidity. The environmental enrichment of the cage included nesting material (aspen shaving, Tapvei), plastic tubes and aspen chip (Tapvei) as bedding. All the mice included in the animal study came from three separate batches based on their date of birth. The mice were randomly assigned to different treatment groups by drawing lots using the cage numbers, ensuring that each treatment group included mice from different batches. To minimize any potential effects from group housing, we made sure that mice from the same treatment group were housed in two different cages. Each cage contained between two to four mice from the same group. Additionally, during the gelatin cube one-week training and seven-weeks intervention, all mice were individually placed in single cages without any enrichment, in order to make sure they were specifically focused in eating the gelatin cube. After consuming the gelatin cube, the mice were returned to their original cages. The mice were trained in order to voluntary consume gelatine cubes containing *E. coli* Nissle rather than using oral gavage. One experimental unit was a single animal. Power analysis was performed using the “G*Power” program (Faul, et al., Behavior Research Methods 41(4): 1149–60 (2009) for estimating the number of mice per group, based on a two-tailed t-test comparing two independent groups of mice. An a priori approach was used with a significance level (α) of 0.05 and statistical power (1-β) of 0.8 to ensure an 80% probability of detecting a true effect. The analysis was initially based on data from a prior study *(Lee HY, et al., Biochim Biophys Acta. 2006;1761(7):736-744)* which included body weight, epididymal white adipose tissue weight, liver weight, serum levels of glucose, leptin and high-density lipoprotein (HDL) cholesterol. The power analysis calculation was further recalculated by using additional outcomes from our previous study (Iannone et al., *Journal of Nutritional Biochemistry*. 115 (2023) including body weight, liver fat content (measured using liver histology with Oil Red O staining) and magnetic resonance imaging. Combining the data from both studies, the power analysis indicated that six mice per group were sufficient to achieve reliable results. None of the mice was excluded from the current study. Researchers involved in the study were aware of the group allocation during the conduction of the animal study. However, during the subsequent data analysis, the process was conducted in a blind manner to prevent any potential biases. The humane endpoints used in the study include laborious inhalation, crooked posture, loss of exercise and weight loss of more than 10%.

The C57BL/6 mouse model used in the current study has been proven to be a good model for studying MASLD (Tetri LH et al., *Am J Physiol Gastrointest Liver Physiol*. 2008, 295(5): G987-G995; Van.

**Data analysis:**

The differences in adipocyte area and diameter between the groups were tested using Kruskal–Wallis test with Games-Howell post-hoc tests was further confirmed with Welch’ Anova sensitivity test as shown below:

Adipocytes area:

- Kruskal-Wallis: chi-squared = 5.6608, df = 2, p-value = 0.05899
- Sensitivity test, Welch One-way analysis of means: F = 4.6471, num df = 2.0000, denom df = 9.0289, **p-value = 0.04097**
- Pairwise comparisons using Games-Howell test, p-value:

EcN vs CTRL== 0.507293

EcNA vs CTRL== **0.038667 ***

EcNA vs EcN == 0.363234

Adipocytes diameter:

- Kruskal-Wallis: chi-squared = 5.4854, df = 2, p-value = 0.0644
- Sensitivity test, Welch One-way analysis of means: F = 5.2742, num df = 2.0000, denom df = 9.4661**, p-value = 0.0289**
- Pairwise comparisons using Games-Howell test, p-value:

EcN vs CTRL== 0.595040

EcNA vs CTRL== **0.021763** *

EcNA vs EcN == 0.332155

WEB-based Gene SeT AnaLysis Toolkit has been used for functional enrichment analysis GSEA (<https://www.webgestalt.org/>; Nucleic Acids Res. 2017 Jul 3;45(Web Server issue). The following parameters have been considered:

- Enrichment method: GSEA
- Organism: mmusculus
- Enrichment Categories: pathway_KEGG

Parameters for the enrichment analysis:

- Minimum number of IDs in the category: 3
- Maximum number of IDs in the category: 2000
- Significance Level: Top 10
- Number of permutation: 1000

Based on the above parameters, the top 10 upregulated pathways and the top 10 downregulated pathways are identified as enriched categories. The final report for each pathway includes: Gene set (KEGG entry of the specific pathway), description, size, leading edge number (number of genes in our dataset included in that specific pathway), **Enrichment Score (**ES), Normalized Enrichment Score (NES), p-value, FDR (**Supplementary table 9**). Additionally, in supplementary table 9, the gene symbols for all genes included in each specific pathway have been reported.

# Supplementary Figures and tables


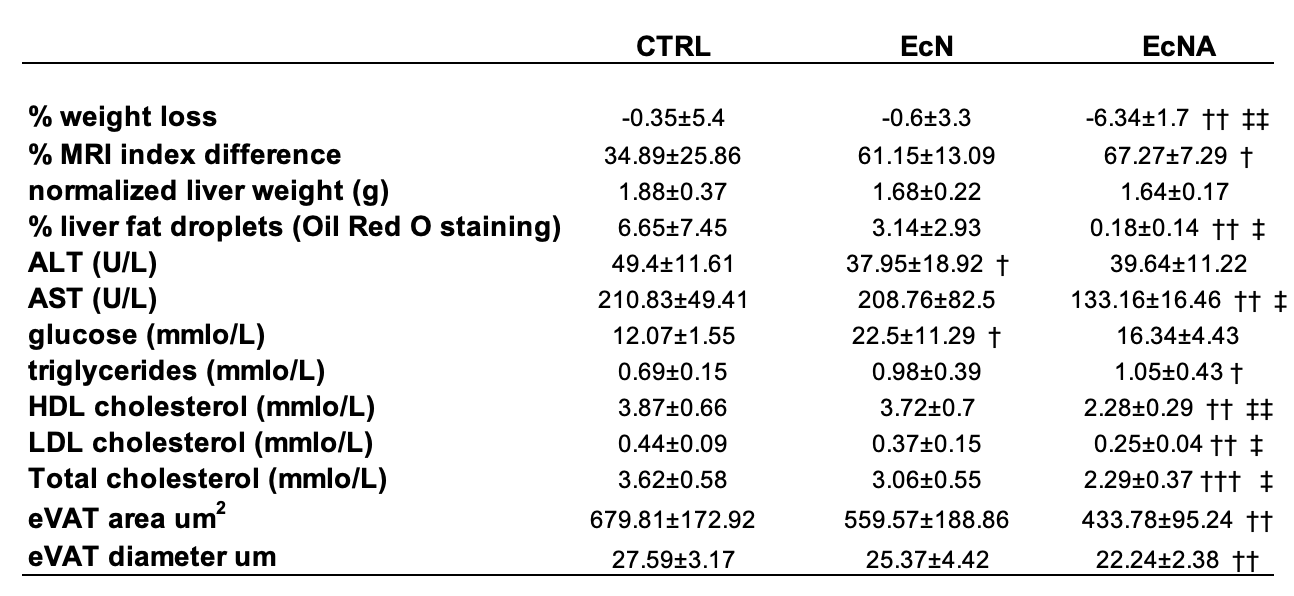


**Supplementary Table 1. Effect of EcNA intervention on body, liver weight, hepatic fat accumulation (MRI and Oil Red O staining), plasma biochemical markers (ALT, AST, glucose, triglycerides, HDL, LDL, total cholesterol) and size of adipocytes in eVAT (eVAT area and diameter).** Results presented as Mean ± SD (n=6/group for all parameters evaluated, with exception for MRI index n=5 mice/CTRL, n=3 mice/ EcNA and EcN. † represented the significance compared to CTRL († p-value <0.05, †† p-value <0.01, ††† p-value < 0.001); ‡ represented the significance compared to EcN (‡ p-value <0.05, ‡‡ p-value <0.01).


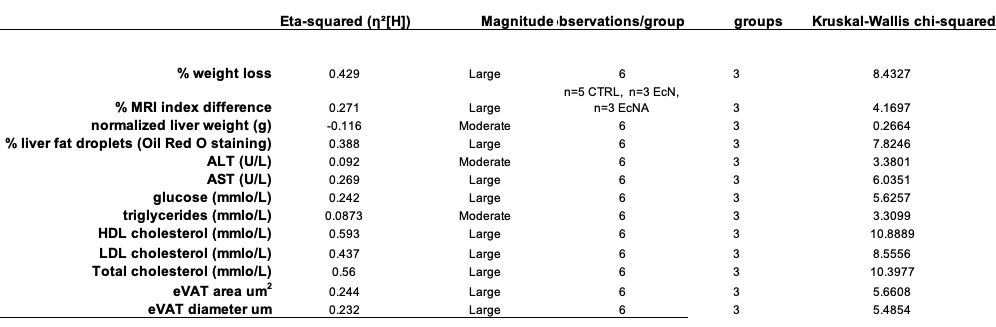
**Supplementary Table 2. Effect size calculated for all parameters included in the study based on Kruskal-Wallis analysis.** Resulting Eta-squared and relative magnitude have been reported in the table.


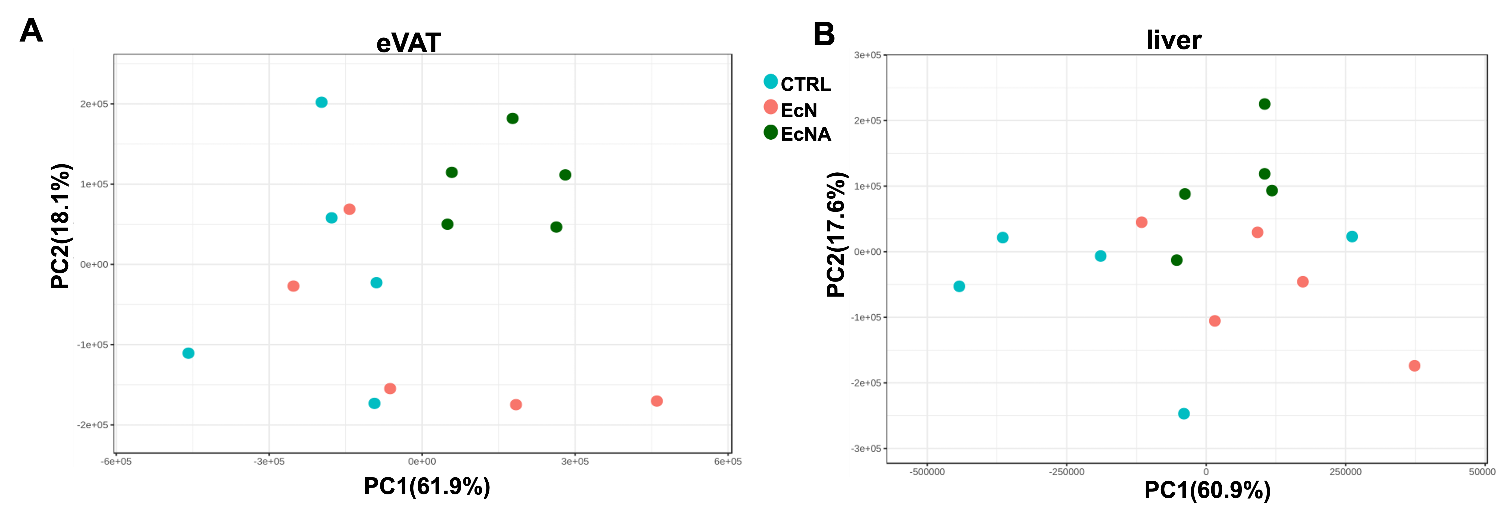


**Supplementary Figure 1. Gene Expression Patterns in Liver and eVAT: EcNA, EcN, and CTRL groups**. PCA of gene count values from RNA-sequencing analysis in **A)** eVAT and **B**) the liver in EcNA, EcN and CTRL groups (n=5/group). PCA: principal component analysis; eVAT: epididymal visceral adipose tissue.


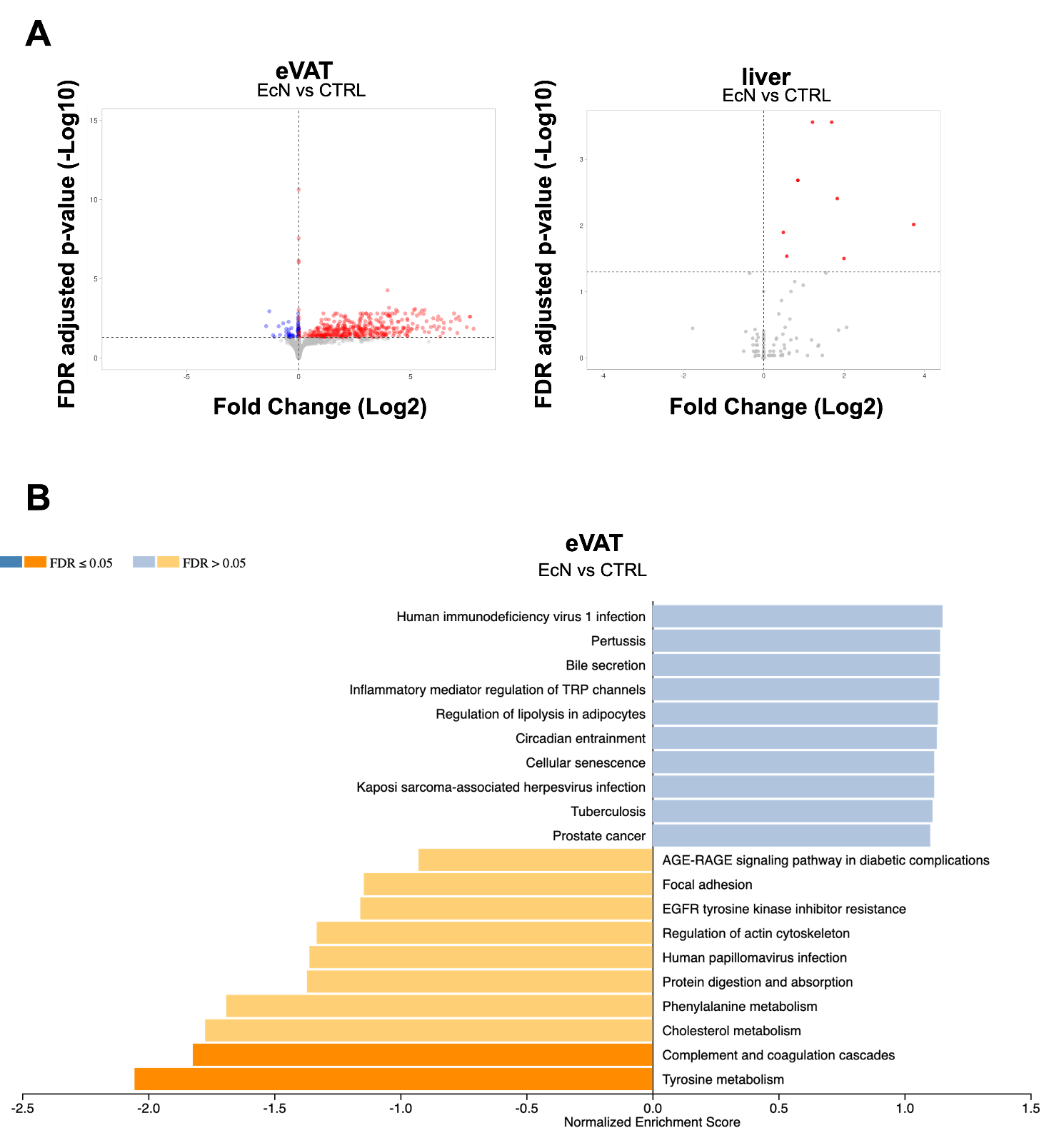


**Supplementary Figure 2. Gene expression changes in both eVAT and the liver in EcN compared to CTRL group. A)** Volcano plot showing the proportion of up- (in red) and downregulated (in blue) genes with FDR adjusted p-value <0.05 in EcN compared to CTRL group in both, the liver and eVAT. **B)** Bar plot showing (in blue) the upregulated and in downregulated (in orange) pathways in eVAT. In the liver, no up- or downregulated pathways were identified. Pathways with FDR adjusted p-value <0.05 were considered significant. eVAT: epididymal visceral adipose tissue. Details of the pathways can be found in Supplementary Table 9.


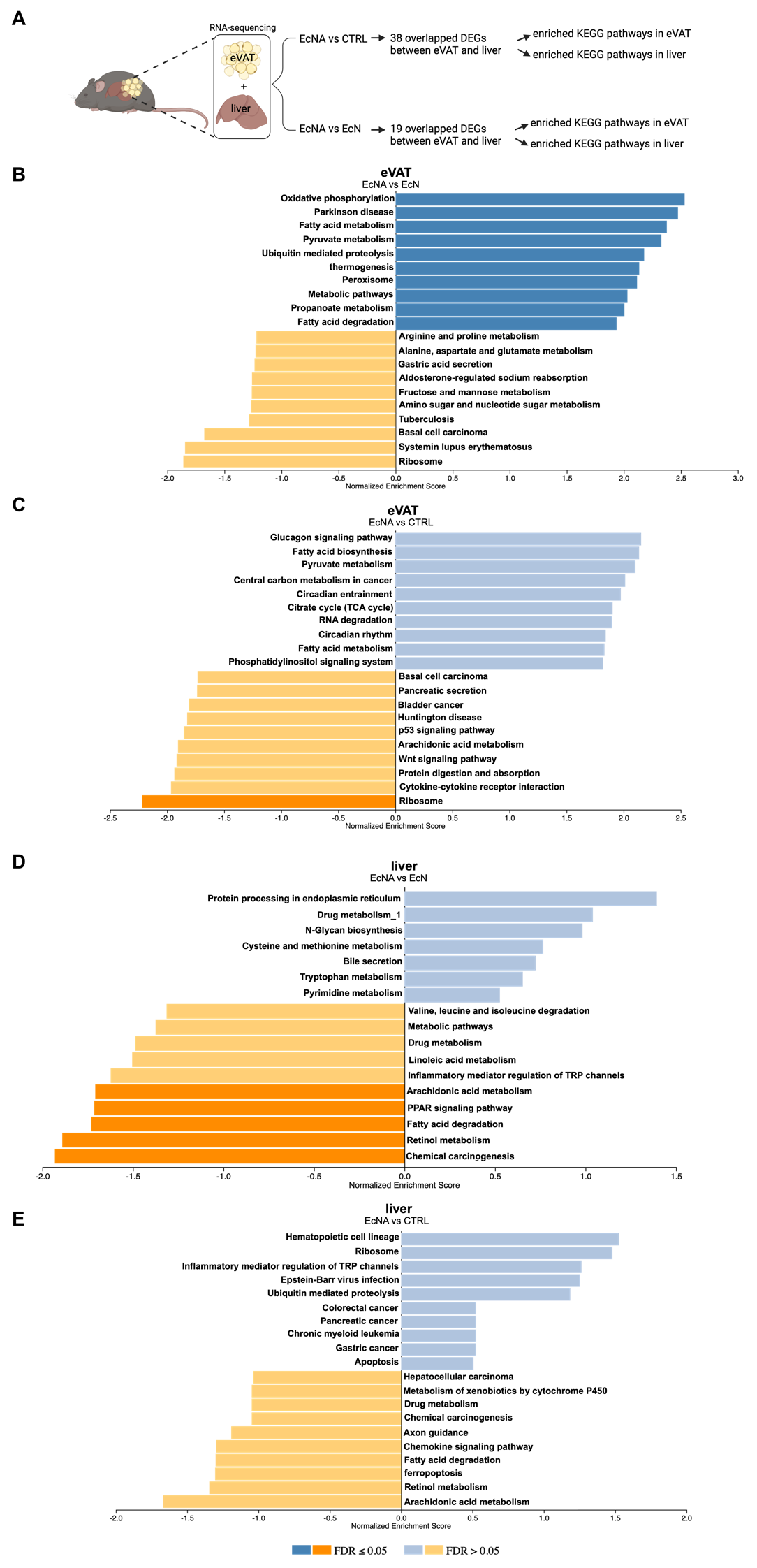


**Supplementary Figure 3**. **Tissue-specific effects of EcNA intervention on gene expression.** **A)** Diagram showing analysis approach for DEGs selection in both tissues and comparisons. Bar plots showing the upregulated (in blue) and the downregulated (in orange) pathways in EcNA vs EcN in eVAT, **C)** EcNA vs CTRL in eVAT, **d)** EcNA vs EcN in the liver and **E)** EcNA vs CTRL in the liver. Pathways with FDR p-value adjusted < 0.05 were considered significant. eVAT: epididymal visceral adipose tissue; DEGs: differentially expressed genes.
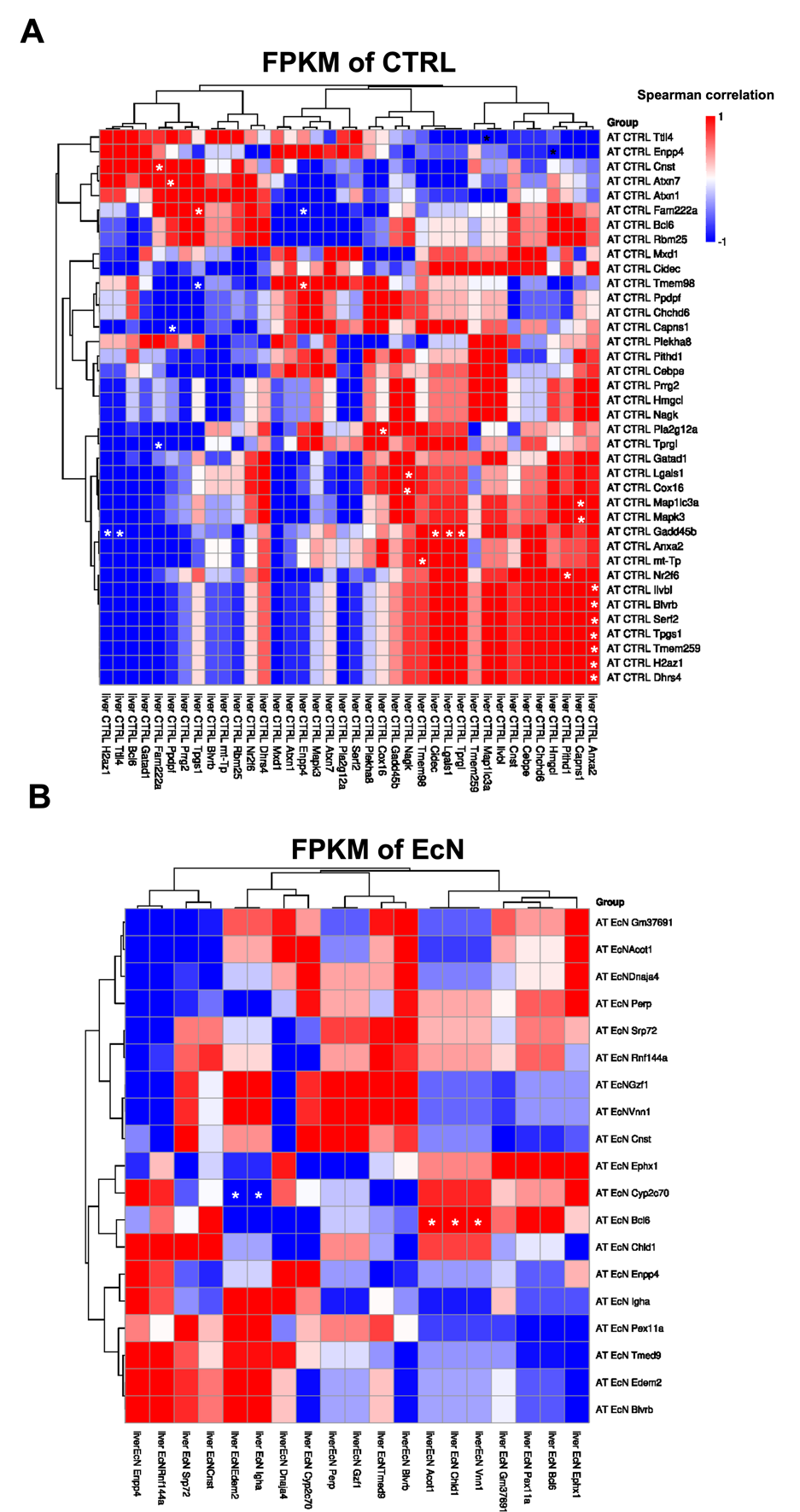


**Supplementary Figure 4. Heatmap showing the Spearman correlation analysis** using the FPKM values of **A**) the CTRL control group (n=5). The FPKM of the 38 overlapped DEGs in both eVAT and liver in EcNA compared to CTRL were selected; **B**) the EcN control group. The FPKM of the 19 overlapped DEGs in both eVAT and liver in EcNA compared to EcN were selected. Positive correlations in red color and negative correlations in blue color. Asterisk identified significant correlations with p-value <0.05. FPKM: Fragments Per Kilobase per Million mapped fragments; DEGs: differentially expressed genes; eVAT/AT: epididymal visceral adipose tissue.
