# Supplementary material for: The effect of aldafermin expressing-Escherichia coli Nissle 1917 along with dietary change on visceral adipose tissue in MASLD mouse model
Source: Int J Obes (Lond). 2025 Apr 10;49(7):1334–44. doi: 10.1038/s41366-025-01774-w (PMC12283412; doi:10.1038/s41366-025-01774-w)
Supplement: Supplementary file 2 — Supplementary table 3 [file 41366_2025_1774_MOESM2_ESM.pdf]

Supplementary table 3. DEGs observed in eVAT when EcNA was compared to EcN

EcNA vs EcN eVAT

ENSEMBL	baseMean	log2FoldChange	lfcSE	pvalue	padj	UD	entrezgene_id	description	external_gene_name	gene_biotype
ENSMUSG00000000093	140.1864307	-0.162211755	0.303417926	0.004183674	0.04696359	Down	21385	T-box 2 [Source:MGI Symbol;Acc:MGI:98494]	Tbx2	protein_coding
ENSMUSG00000000168	2696.694623	0.456450715	0.122791085	2.25E-05	0.002705768	Up	235339	dihydrolipoamide S-acetyltransferase (E2 component of pyruvate dehydrogenase complex) [Source:MGI Symbol;Acc:MGI:2385311]	Dlat	protein_coding
ENSMUSG00000000263	17.46784688	-0.173690786	0.329783128	0.003537544	0.043004611	Down	14654	glycine receptor, alpha 1 subunit [Source:MGI Symbol;Acc:MGI:95747]	Glra1	protein_coding
ENSMUSG00000000266	714.7775747	0.352003417	0.157114673	0.003354569	0.0417232	Up	23947	midline 2 [Source:MGI Symbol;Acc:MGI:1344333]	Mid2	protein_coding
ENSMUSG00000000275	1125.555071	0.348883226	0.134452537	0.001393829	0.02591718	Up	217069	tripartite motif-containing 25 [Source:MGI Symbol;Acc:MGI:102749]	Trim25	protein_coding
ENSMUSG00000000276	466.5732006	0.374648629	0.162714108	0.002603001	0.036231825	Up	56077	diacylglycerol kinase, epsilon [Source:MGI Symbol;Acc:MGI:1889276]	Dgke	protein_coding
ENSMUSG00000000340	2033.423975	0.39672546	0.167626299	0.001972988	0.031241133	Up	13171	dihydrolipoamide branched chain transacylase E2 [Source:MGI Symbol;Acc:MGI:105386]	Dbt	protein_coding
ENSMUSG00000000530	1135.372188	-0.254434182	0.107667143	0.003822964	0.045022918	Down	11482	activin A receptor, type II-like 1 [Source:MGI Symbol;Acc:MGI:1338946]	Acvrl1	protein_coding
ENSMUSG00000000600	768.5700918	0.303028647	0.127165975	0.003013361	0.038803026	Up	79264	KRIT1, ankyrin repeat containing [Source:MGI Symbol;Acc:MGI:1930618]	Krit1	protein_coding
ENSMUSG00000000708	1822.716558	0.384734201	0.12467514	0.000248634	0.009737966	Up	18519	K(lysine) acetyltransferase 2B [Source:MGI Symbol;Acc:MGI:1343094]	Kat2b	protein_coding
ENSMUSG00000000740	7628.807071	-0.692839675	0.203543441	4.71E-05	0.004134689	Down	270106	ribosomal protein L13 [Source:MGI Symbol;Acc:MGI:105922]	Rpl13	protein_coding
ENSMUSG00000000776	182.8244568	-0.311647858	0.137669363	0.004171971	0.04696359	Down	67065	polymerase (RNA) III (DNA directed) polypeptide D [Source:MGI Symbol;Acc:MGI:1914315]	Polr3d	protein_coding
ENSMUSG00000001025	2967.893201	-0.663708828	0.258409123	0.000573667	0.016152764	Down	20200	S100 calcium binding protein A6 (calcyclin) [Source:MGI Symbol;Acc:MGI:1339467]	S100a6	protein_coding
ENSMUSG00000001052	1617.9432	0.636050719	0.172712563	1.59E-05	0.002315292	Up	99683	Sec24 related gene family, member B (S. cerevisiae) [Source:MGI Symbol;Acc:MGI:2139764]	Sec24b	protein_coding
ENSMUSG00000001056	452.4624545	-0.443697062	0.236628035	0.004656116	0.049683496	Down	52530	NHP2 ribonucleoprotein [Source:MGI Symbol;Acc:MGI:1098547]	Nhp2	protein_coding
ENSMUSG00000001082	383.9647439	-0.360681654	0.113580644	0.000190318	0.00835522	Down	68294	major facilitator superfamily domain containing 10 [Source:MGI Symbol;Acc:MGI:1915544]	Mfsd10	protein_coding
ENSMUSG00000001119	10095.51491	-0.482094287	0.264370729	0.0043969	0.048144948	Down	12833	collagen, type VI, alpha 1 [Source:MGI Symbol;Acc:MGI:88459]	Col6a1	protein_coding
ENSMUSG00000001131	75.05840834	-0.92011878	0.364218553	0.000464534	0.014269724	Down	21857	tissue inhibitor of metalloproteinase 1 [Source:MGI Symbol;Acc:MGI:98752]	Timp1	protein_coding
ENSMUSG00000001156	251.7611405	0.33953993	0.156185455	0.004232058	0.047149397	Up	17119	MAX dimerization protein 1 [Source:MGI Symbol;Acc:MGI:96908]	Mxd1	protein_coding
ENSMUSG00000001270	1839.416631	-0.801189056	0.267062888	0.000141852	0.007452872	Down	12709	creatine kinase, brain [Source:MGI Symbol;Acc:MGI:88407]	Ckb	protein_coding
ENSMUSG00000001313	143.0290093	-0.688999632	0.245517243	0.000279801	0.010259187	Down	11858	Rho family GTPase 2 [Source:MGI Symbol;Acc:MGI:1338755]	Rnd2	protein_coding
ENSMUSG00000001333	60.02755721	-0.816117267	0.325458225	0.000535735	0.015438654	Down	68828	syncoilin [Source:MGI Symbol;Acc:MGI:1916078]	Sync	protein_coding
ENSMUSG00000001383	846.3007789	-0.459635283	0.154006077	0.000273767	0.010102419	Down	66492	zinc finger, matrin type 2 [Source:MGI Symbol;Acc:MGI:1913742]	Zmat2	protein_coding
ENSMUSG00000001435	6009.178858	-0.673362298	0.228029476	0.000196587	0.008533442	Down	12822	collagen, type XVIII, alpha 1 [Source:MGI Symbol;Acc:MGI:88451]	Col18a1	protein_coding
ENSMUSG00000001558	10.55074015	-0.133987622	0.255079291	0.002591556	0.036162167	Down	66720	kelch-like 10 [Source:MGI Symbol;Acc:MGI:2181067]	Klhl10	protein_coding
ENSMUSG00000001583	52.68700489	-0.825237699	0.348561045	0.000746018	0.018768079	Down	83813	tyrosine kinase, non-receptor, 1 [Source:MGI Symbol;Acc:MGI:1930958]	Tnk1	protein_coding
ENSMUSG00000001604	64.20682766	-0.502381136	0.251551094	0.002905949	0.038239246	Down	21401	transcription elongation factor A (SII), 3 [Source:MGI Symbol;Acc:MGI:1196908]	Tcea3	protein_coding
ENSMUSG00000001763	61.35005598	-1.403346796	0.513435302	0.000211752	0.009008922	Down	232670	tetraspanin 33 [Source:MGI Symbol;Acc:MGI:1919012]	Tspan33	protein_coding
ENSMUSG00000001773	476.9642828	-0.577401236	0.289418597	0.002379891	0.034681162	Down	53320	folate hydrolase 1 [Source:MGI Symbol;Acc:MGI:1858193]	Folh1	protein_coding
ENSMUSG00000001827	41.0699813	-1.585895408	0.609426617	0.000284419	0.010362345	Down	14275	folate receptor 1 (adult) [Source:MGI Symbol;Acc:MGI:95568]	Folr1	protein_coding
ENSMUSG00000002043	301.7588475	-0.504102195	0.216901559	0.001418779	0.026099657	Down	67091	trafficking protein particle complex 6A [Source:MGI Symbol;Acc:MGI:1914341]	Trappc6a	protein_coding
ENSMUSG00000002107	5604.749384	0.280180989	0.118828522	0.004138282	0.046825357	Up	14007	CUGBP, Elav-like family member 2 [Source:MGI Symbol;Acc:MGI:1338822]	Celf2	protein_coding
ENSMUSG00000002190	5.376668181	-0.042226759	0.175803733	0.004637609	0.049578114	Down	12745	calmegin [Source:MGI Symbol;Acc:MGI:107472]	Clgn	protein_coding
ENSMUSG00000002204	81.62668858	-1.738910323	0.49052764	1.47E-05	0.002198172	Down	16541	napsin A aspartic peptidase [Source:MGI Symbol;Acc:MGI:109365]	Napsa	protein_coding
ENSMUSG00000002222	2339.638977	0.264372759	0.070390494	4.87E-05	0.004178515	Up	68477	required for meiotic nuclear division 5 homolog A [Source:MGI Symbol;Acc:MGI:1915727]	Rmnd5a	protein_coding
ENSMUSG00000002233	1581.475394	-0.759230799	0.235249333	6.71E-05	0.004965078	Down	11853	ras homolog family member C [Source:MGI Symbol;Acc:MGI:106028]	Rhoc	protein_coding
ENSMUSG00000002249	196.6678743	-0.472008055	0.227031532	0.00280058	0.0374395	Down	21678	TEA domain family member 3 [Source:MGI Symbol;Acc:MGI:109241]	Tead3	protein_coding
ENSMUSG00000002274	29.9321473	-0.212602137	0.48433155	0.001729465	0.029034732	Down	70083	meteorin, glial cell differentiation regulator [Source:MGI Symbol;Acc:MGI:1917333]	Metrn	protein_coding
ENSMUSG00000002393	428.0588247	-0.782344463	0.237183697	5.02E-05	0.004262805	Down	13864	nuclear receptor subfamily 2, group F, member 6 [Source:MGI Symbol;Acc:MGI:1352453]	Nr2f6	protein_coding
ENSMUSG00000002580	235.655204	-1.018038466	0.405655882	0.000447925	0.01398387	Down	103742	migration and invasion enhancer 1 [Source:MGI Symbol;Acc:MGI:1913678]	Mien1	protein_coding
ENSMUSG00000002658	571.3674277	-0.555719411	0.214058992	0.000625018	0.017069631	Down	98053	general transcription factor IIF, polypeptide 1 [Source:MGI Symbol;Acc:MGI:1923848]	Gtf2f1	protein_coding
ENSMUSG00000002718	960.6425638	0.358390125	0.101304637	6.91E-05	0.0050266	Up	110750	chromosome segregation 1-like (S. cerevisiae) [Source:MGI Symbol;Acc:MGI:1339951]	Cse1l	protein_coding
ENSMUSG00000002768	493.1502737	-0.484810224	0.26461363	0.004195896	0.04696359	Down	17256	male enhanced antigen 1 [Source:MGI Symbol;Acc:MGI:96957]	Mea1	protein_coding
ENSMUSG00000002804	126.0831237	-0.77752434	0.18866197	2.28E-06	0.000834238	Down	66174	nudix (nucleoside diphosphate linked moiety X)-type motif 14 [Source:MGI Symbol;Acc:MGI:1913424]	Nudt14	protein_coding
ENSMUSG00000002835	73.37254785	-0.616217509	0.37317688	0.003988779	0.045835427	Down	27221	chromatin assembly factor 1, subunit A (p150) [Source:MGI Symbol;Acc:MGI:1351331]	Chaf1a	protein_coding
ENSMUSG00000002847	1058.396241	-0.437476578	0.220146961	0.003543582	0.043004611	Down	85031	phospholipase A1 member A [Source:MGI Symbol;Acc:MGI:1934677]	Pla1a	protein_coding
ENSMUSG00000002881	1210.593212	0.326417853	0.093694137	9.32E-05	0.005856894	Up	17936	Ngfi-A binding protein 1 [Source:MGI Symbol;Acc:MGI:107564]	Nab1	protein_coding
ENSMUSG00000002910	315.960251	-0.707149044	0.189607342	1.19E-05	0.00193348	Down	70807	arrestin domain containing 2 [Source:MGI Symbol;Acc:MGI:1918057]	Arrdc2	protein_coding
ENSMUSG00000002944	76252.27429	0.462238158	0.130946904	5.65E-05	0.00449019	Up	12491	CD36 molecule [Source:MGI Symbol;Acc:MGI:107899]	Cd36	protein_coding
ENSMUSG00000002980	1669.911749	-0.564188679	0.162255644	4.28E-05	0.003960507	Down	57278	basal cell adhesion molecule [Source:MGI Symbol;Acc:MGI:1929940]	Bcam	protein_coding
ENSMUSG00000002985	66291.16679	-0.685623068	0.229120835	0.000157676	0.007675767	Down	11816	apolipoprotein E [Source:MGI Symbol;Acc:MGI:88057]	Apoe	protein_coding
ENSMUSG00000003200	775.5612086	-0.330016808	0.130538704	0.001801968	0.029687686	Down	20405	SH3-domain GRB2-like 1 [Source:MGI Symbol;Acc:MGI:700010]	Sh3gl1	protein_coding
ENSMUSG00000003345	2273.655459	-0.509908749	0.089119647	5.87E-10	1.01E-05	Down	103236	casein kinase 1, gamma 2 [Source:MGI Symbol;Acc:MGI:1920014]	Csnk1g2	protein_coding

ENSMUSG00000003346	1387.392584	-0.361076544	0.16997162	0.004044021	0.046192933	Down	216169	abhydrolase domain containing 17A [Source:MGI Symbol;Acc:MGI:106388]	Abhd17a	protein_coding
ENSMUSG00000003363	1249.391625	-0.326747593	0.128680066	0.001642202	0.028305152	Down	18807	phospholipase D family, member 3 [Source:MGI Symbol;Acc:MGI:1333782]	Pld3	protein_coding
ENSMUSG00000003379	57.01029057	-0.076258389	0.193489765	0.00426901	0.047401959	Down	12518	CD79A antigen (immunoglobulin-associated alpha) [Source:MGI Symbol;Acc:MGI:101774]	Cd79a	protein_coding
ENSMUSG00000003380	3362.016937	-0.468387349	0.139704295	8.06E-05	0.00531048	Down	14470	Rab acceptor 1 (prenylated) [Source:MGI Symbol;Acc:MGI:1201692]	Rabac1	protein_coding
ENSMUSG00000003534	1838.960142	-0.749531688	0.255386762	0.000170119	0.007988188	Down	12305	discoidin domain receptor family, member 1 [Source:MGI Symbol;Acc:MGI:99216]	Ddr1	protein_coding
ENSMUSG00000003546	373.4300524	-0.407415265	0.153361381	0.000881857	0.020528033	Down	74764	kinesin light chain 4 [Source:MGI Symbol;Acc:MGI:1922014]	Klc4	protein_coding
ENSMUSG00000003559	199.8240862	-0.44434258	0.213467354	0.003029797	0.0388775	Down	57344	arsenite methyltransferase [Source:MGI Symbol;Acc:MGI:1929882]	As3mt	protein_coding
ENSMUSG00000003865	2781.583243	0.419666984	0.179105401	0.002041028	0.031917456	Up	14936	glycogen synthase 1, muscle [Source:MGI Symbol;Acc:MGI:101805]	Gys1	protein_coding
ENSMUSG00000003873	368.6272607	-0.527754295	0.240373218	0.00183341	0.030037054	Down	12028	BCL2-associated X protein [Source:MGI Symbol;Acc:MGI:99702]	Bax	protein_coding
ENSMUSG00000003970	6465.675347	-0.445410341	0.184054061	0.001378037	0.025872156	Down	26961	ribosomal protein L8 [Source:MGI Symbol;Acc:MGI:1350927]	Rpl8	protein_coding
ENSMUSG00000004035	249.1386854	-1.545228771	0.708653085	0.00076391	0.019013207	Down	68312	glutathione S-transferase, mu 7 [Source:MGI Symbol;Acc:MGI:1915562]	Gstm7	protein_coding
ENSMUSG00000004043	1296.242961	0.344918875	0.140372103	0.002195583	0.033336104	Up	20850	signal transducer and activator of transcription 5A [Source:MGI Symbol;Acc:MGI:103036]	Stat5a	protein_coding
ENSMUSG00000004110	258.4324349	-1.452097547	0.479315385	8.99E-05	0.005761676	Down	12290	calcium channel, voltage-dependent, R type, alpha 1E subunit [Source:MGI Symbol;Acc:MGI:106217]	Cacna1e	protein_coding
ENSMUSG00000004285	827.3277757	-0.462290737	0.1536671	0.00025008	0.009765934	Down	66144	ATPase, H+ transporting, lysosomal V1 subunit F [Source:MGI Symbol;Acc:MGI:1913394]	Atp6v1f	protein_coding
ENSMUSG00000004360	390.2729174	0.644228645	0.304650086	0.001708818	0.028849913	Up	212448	RIKEN cDNA 9330159F19 gene [Source:MGI Symbol;Acc:MGI:3036239]	9330159F19Rik	protein_coding
ENSMUSG00000004364	2175.801261	0.305910324	0.092240809	0.000194673	0.008493181	Up	26554	cullin 3 [Source:MGI Symbol;Acc:MGI:1347360]	Cul3	protein_coding
ENSMUSG00000004665	925.5244399	-0.708766073	0.164280858	1.02E-06	0.000478129	Down	12798	calponin 2 [Source:MGI Symbol;Acc:MGI:105093]	Cnn2	protein_coding
ENSMUSG00000004814	316.8040305	-0.636706703	0.233917995	0.000380812	0.012610537	Down	56221	chemokine (C-C motif) ligand 24 [Source:MGI Symbol;Acc:MGI:1928953]	Ccl24	protein_coding
ENSMUSG00000004864	18.12302172	-1.579335621	0.774978612	0.001033728	0.022436061	Down	26415	mitogen-activated protein kinase 13 [Source:MGI Symbol;Acc:MGI:1346864]	Mapk13	protein_coding
ENSMUSG00000004929	420.1286178	-0.400260202	0.125475455	0.000177809	0.008185484	Down	50492	thimet oligopeptidase 1 [Source:MGI Symbol;Acc:MGI:1354165]	Thop1	protein_coding
ENSMUSG00000004951	2389.220763	-0.591235801	0.212523342	0.000386324	0.012681007	Down	15507	heat shock protein 1 [Source:MGI Symbol;Acc:MGI:96240]	Hspb1	protein_coding
ENSMUSG00000005237	36.77826623	-0.15721026	0.303649266	0.000568889	0.016114087	Down	327954	dynein, axonemal, heavy chain 2 [Source:MGI Symbol;Acc:MGI:107731]	Dnah2	protein_coding
ENSMUSG00000005312	5461.225852	0.244860545	0.083645486	0.001054779	0.022664606	Up	56085	ubiquilin 1 [Source:MGI Symbol;Acc:MGI:1860276]	Ubqln1	protein_coding
ENSMUSG00000005360	4214.505023	0.850780145	0.39081307	0.001126728	0.023450356	Up	20512	solute carrier family 1 (glial high affinity glutamate transporter), member 3 [Source:MGI Symbol;Acc:MGI:99917]	Slc1a3	protein_coding
ENSMUSG00000005371	1159.332736	0.395868727	0.097250464	6.83E-06	0.001444279	Up	225055	F-box protein 11 [Source:MGI Symbol;Acc:MGI:2147134]	Fbxo11	protein_coding
ENSMUSG00000005373	1823.726199	0.521124695	0.230362101	0.00155097	0.027385104	Up	58805	MLX interacting protein-like [Source:MGI Symbol;Acc:MGI:1927999]	Mlxipl	protein_coding
ENSMUSG00000005447	60.75218199	-1.178833216	0.354654825	3.71E-05	0.003584747	Down	18476	platelet-activating factor acetylhydrolase, isoform 1b, subunit 3 [Source:MGI Symbol;Acc:MGI:108414]	Pafah1b3	protein_coding
ENSMUSG00000005501	723.1147426	0.248918007	0.104533132	0.004544635	0.049102004	Up	227334	ubiquitin specific peptidase 40 [Source:MGI Symbol;Acc:MGI:2443184]	Usp40	protein_coding
ENSMUSG00000005683	9318.131715	0.427807704	0.128374498	0.0001978	0.006812419	Up	12974	citrate synthase [Source:MGI Symbol;Acc:MGI:88529]	Cs	protein_coding
ENSMUSG00000005698	956.0595291	0.273134126	0.080938704	0.00019006	0.00835522	Up	13018	CCCTC-binding factor [Source:MGI Symbol;Acc:MGI:109447]	Ctcf	protein_coding
ENSMUSG00000005802	614.1761656	0.382210006	0.123173681	0.000263323	0.009995247	Up	22785	solute carrier family 30 (zinc transporter), member 4 [Source:MGI Symbol;Acc:MGI:1345282]	Slc30a4	protein_coding
ENSMUSG00000005897	334.737966	0.364804946	0.098643637	3.40E-05	0.003431462	Up	22025	nuclear receptor subfamily 2, group C, member 1 [Source:MGI Symbol;Acc:MGI:1352465]	Nr2c1	protein_coding
ENSMUSG00000005907	423.5897806	0.425315432	0.119548644	4.80E-05	0.004153054	Up	71382	peroxisomal biogenesis factor 1 [Source:MGI Symbol;Acc:MGI:1918632]	Pex1	protein_coding
ENSMUSG00000005973	1104.127301	-0.304762176	0.113804385	0.00144363	0.026307269	Down	19672	reticulocalbin 1 [Source:MGI Symbol;Acc:MGI:104559]	Rcn1	protein_coding
ENSMUSG00000006095	765.7349365	-0.366610714	0.151487709	0.001947242	0.030956483	Down	66411	tubulin folding cofactor B [Source:MGI Symbol;Acc:MGI:1913661]	Tbcb	protein_coding
ENSMUSG00000006134	771.4898195	0.183538273	0.069880823	0.004009864	0.045941738	Up	12929	v-crk avian sarcoma virus CT10 oncogene homolog-like [Source:MGI Symbol;Acc:MGI:104686]	Crkl	protein_coding
ENSMUSG00000006215	238.3896293	-0.329169044	0.130692408	0.001842613	0.030090232	Down	22642	zinc finger and BTB domain containing 17 [Source:MGI Symbol;Acc:MGI:107410]	Zbtb17	protein_coding
ENSMUSG00000006221	1298.197782	-1.743429373	0.734373994	0.000476938	0.014368997	Down	29818	heat shock protein family, member 7 (cardiovascular) [Source:MGI Symbol;Acc:MGI:1352494]	Hspb7	protein_coding
ENSMUSG00000006262	841.6622079	0.330305528	0.091699774	5.88E-05	0.004584839	Up	68473	MOB kinase activator 1B [Source:MGI Symbol;Acc:MGI:1915723]	Mob1b	protein_coding
ENSMUSG00000006333	6805.566749	-0.629201646	0.216092864	0.000233291	0.009327844	Down	76846	ribosomal protein S9 [Source:MGI Symbol;Acc:MGI:1924096]	Rps9	protein_coding
ENSMUSG00000006335	180.8908117	-0.691840117	0.280246063	0.000683979	0.017940666	Down	69714	TCF3 (E2A) fusion partner [Source:MGI Symbol;Acc:MGI:1916964]	Tfpt	protein_coding
ENSMUSG00000006342	227.2536486	-0.561220588	0.258216457	0.001724239	0.029034732	Down	71733	sushi domain containing 2 [Source:MGI Symbol;Acc:MGI:1918983]	Susd2	protein_coding
ENSMUSG00000006356	1492.152139	-0.56974401	0.23785874	0.001054352	0.022664606	Down	68337	cysteine rich protein 2 [Source:MGI Symbol;Acc:MGI:1915587]	Crip2	protein_coding
ENSMUSG00000006360	2404.662748	-1.482077191	0.329266388	2.95E-07	0.000241407	Down	12925	cysteine-rich protein 1 (intestinal) [Source:MGI Symbol;Acc:MGI:88501]	Crip1	protein_coding
ENSMUSG00000006395	334.7955442	-0.422741282	0.143360595	0.000349175	0.011798701	Down	NA	hydroxypyruvate isomerase (putative) [Source:MGI Symbol;Acc:MGI:1915430]	Hyi	protein_coding
ENSMUSG00000006423	1737.555017	0.275820922	0.113291152	0.003426838	0.042478059	Up	77644	STING1 ER exit protein 1 [Source:MGI Symbol;Acc:MGI:1924894]	Steep1	protein_coding
ENSMUSG00000006494	987.9848994	0.723512253	0.199891621	1.85E-05	0.002550282	Up	228026	pyruvate dehydrogenase kinase, isoenzyme 1 [Source:MGI Symbol;Acc:MGI:1926119]	Pdk1	protein_coding
ENSMUSG00000006519	747.5082435	-0.614234239	0.278371491	0.001432548	0.026179313	Down	13057	cytochrome b-245, alpha polypeptide [Source:MGI Symbol;Acc:MGI:1316658]	Cyba	protein_coding
ENSMUSG00000006641	836.5296049	1.351474103	0.374887506	1.31E-05	0.002075983	Up	330064	solute carrier family 5 (sodium-dependent vitamin transporter), member 6 [Source:MGI Symbol;Acc:MGI:2660847]	Slc5a6	protein_coding
ENSMUSG00000006728	867.8218093	-0.301221676	0.123117118	0.002799901	0.0374395	Down	12567	cyclin-dependent kinase 4 [Source:MGI Symbol;Acc:MGI:88357]	Cdk4	protein_coding
ENSMUSG00000006782	422.9093365	-0.455855335	0.132411331	5.45E-05	0.004447279	Down	12799	2',3'-cyclic nucleotide 3' phosphodiesterase [Source:MGI Symbol;Acc:MGI:88437]	Cnp	protein_coding
ENSMUSG00000006906	521.1930038	0.257172041	0.092178003	0.001415474	0.026099657	Up	70527	STAM binding protein [Source:MGI Symbol;Acc:MGI:1917777]	Stambp	protein_coding
ENSMUSG00000007035	21.03777168	-1.18842864	0.425726834	0.000203126	0.008729343	Down	17687	mutS homolog 5 [Source:MGI Symbol;Acc:MGI:1329021]	Msh5	protein_coding

ENSMUSG00000007039	448.7605024	-0.52310122	0.149744096	4.09E-05	0.003847331	Down	51793	dimethylarginine dimethylaminohydrolase 2 [Source:MGI Symbol;Acc:MGI:1859016]	Ddah2	protein_coding
ENSMUSG00000007107	49.70434999	-2.003245317	0.690590943	0.000119419	0.006812419	Down	27222	ATPase, Na+/K+ transporting, alpha 4 polypeptide [Source:MGI Symbol;Acc:MGI:1351335]	Atp1a4	protein_coding
ENSMUSG00000007207	67.72048331	-0.544754824	0.255833962	0.001986231	0.031373704	Down	20907	syntaxin 1A (brain) [Source:MGI Symbol;Acc:MGI:109355]	Stx1a	protein_coding
ENSMUSG00000007812	1121.854102	0.311003846	0.135226207	0.003825476	0.045022918	Up	72611	zinc finger protein 655 [Source:MGI Symbol;Acc:MGI:1919861]	Zfp655	protein_coding
ENSMUSG00000007833	376.3961657	-0.391937124	0.155560074	0.001358305	0.025674417	Down	69748	aldehyde dehydrogenase 16 family, member A1 [Source:MGI Symbol;Acc:MGI:1916998]	Aldh16a1	protein_coding
ENSMUSG00000007836	2654.935481	-0.654327691	0.210786489	0.000120966	0.0068438	Down	77134	heterogeneous nuclear ribonucleoprotein A0 [Source:MGI Symbol;Acc:MGI:1924384]	Hnrnpa0	protein_coding
ENSMUSG00000007867	213.1866551	-0.474984535	0.168008632	0.000421269	0.013519063	Down	76411	intraflagellar transport 43 [Source:MGI Symbol;Acc:MGI:1923661]	Ift43	protein_coding
ENSMUSG00000007987	311.9831816	-0.527146553	0.142668857	1.88E-05	0.002550282	Down	67286	intraflagellar transport 22 [Source:MGI Symbol;Acc:MGI:1914536]	Ift22	protein_coding
ENSMUSG00000008167	143.5786249	-0.523266186	0.259677677	0.002649707	0.036638703	Down	68628	F-box and WD-40 domain protein 9 [Source:MGI Symbol;Acc:MGI:1915878]	Fbxw9	protein_coding
ENSMUSG00000008200	814.1544305	0.276984483	0.118632116	0.004138048	0.046825357	Up	55935	formin binding protein 4 [Source:MGI Symbol;Acc:MGI:1860513]	Fnbp4	protein_coding
ENSMUSG00000008318	76.48197873	-0.758146989	0.332788421	0.00104294	0.022532874	Down	320100	RELT tumor necrosis factor receptor [Source:MGI Symbol;Acc:MGI:2443373]	Relt	protein_coding
ENSMUSG00000008429	1339.687652	0.26558819	0.081581972	0.00030593	0.010759386	Up	80517	HERPUD family member 2 [Source:MGI Symbol;Acc:MGI:1915393]	Herpud2	protein_coding
ENSMUSG00000008668	4988.405802	-0.434036878	0.169099825	0.000998416	0.021974067	Down	20084	ribosomal protein S18 [Source:MGI Symbol;Acc:MGI:98146]	Rps18	protein_coding
ENSMUSG00000008690	1481.170492	-0.202045968	0.076673935	0.003008045	0.038784775	Down	52683	non-SMC condensin II complex, subunit H2 [Source:MGI Symbol;Acc:MGI:1289164]	Ncaph2	protein_coding
ENSMUSG00000008822	148.2318973	-0.741805848	0.248860711	0.000152413	0.007613147	Down	66204	acylphosphatase 1, erythrocyte (common) type [Source:MGI Symbol;Acc:MGI:1913454]	Acyp1	protein_coding
ENSMUSG00000008976	927.7852784	0.23552544	0.083645973	0.001511793	0.027025659	Up	14390	GA repeat binding protein, alpha [Source:MGI Symbol;Acc:MGI:95610]	Gabpa	protein_coding
ENSMUSG00000009076	161.6411131	-0.601486778	0.239045558	0.000707526	0.018307504	Down	67178	zinc finger, matrin type 5 [Source:MGI Symbol;Acc:MGI:1914428]	Zmat5	protein_coding
ENSMUSG00000009281	4472.09587	-0.591743283	0.198996907	0.000198607	0.008556482	Down	71660	retinoic acid receptor responder (tazarotene induced) 2 [Source:MGI Symbol;Acc:MGI:1918910]	Rarres2	protein_coding
ENSMUSG00000009545	58.78260896	-0.896197758	0.280129378	6.79E-05	0.004991657	Down	16535	potassium voltage-gated channel, subfamily Q, member 1 [Source:MGI Symbol;Acc:MGI:108083]	Kcnq1	protein_coding
ENSMUSG00000009566	237.7152772	-0.306907606	0.12314388	0.00228589	0.033813517	Down	14287	folylpolyglutamyl synthetase [Source:MGI Symbol;Acc:MGI:95576]	Fpgs	protein_coding
ENSMUSG00000009772	133.8948332	-0.488482492	0.274880014	0.004581538	0.049276724	Down	74137	NUAK family, SNF1-like kinase, 2 [Source:MGI Symbol;Acc:MGI:1921387]	Nuak2	protein_coding
ENSMUSG00000010095	2281.388748	-0.436439114	0.205815095	0.002713808	0.036824446	Down	17254	solute carrier family 3 (activators of dibasic and neutral amino acid transport), member 2 [Source:MGI Symbol;Acc:MGI:96955]	Slc3a2	protein_coding
ENSMUSG00000010607	296.6992336	-0.561258191	0.237777418	0.001100652	0.02321297	Down	66268	phosphatidylinositol glycan anchor biosynthesis, class Y-like [Source:MGI Symbol;Acc:MGI:1913518]	Pigyl	protein_coding
ENSMUSG00000010797	10.71613348	-1.828196141	0.685085538	0.000302941	0.010741943	Down	22413	wingless-type MMTV integration site family, member 2 [Source:MGI Symbol;Acc:MGI:98954]	Wnt2	protein_coding
ENSMUSG00000010914	941.9163746	0.359214387	0.170638527	0.004351209	0.047943985	Up	27402	pyruvate dehydrogenase complex, component X [Source:MGI Symbol;Acc:MGI:1351627]	Pdhx	protein_coding
ENSMUSG00000011658	214.8762018	-0.362727296	0.16219526	0.002804471	0.0374395	Down	70300	fuzzy planar cell polarity protein [Source:MGI Symbol;Acc:MGI:1917550]	Fuz	protein_coding
ENSMUSG00000012076	357.5215071	0.276028023	0.110830233	0.00283754	0.037752196	Up	52592	breast cancer metastasis-suppressor 1-like [Source:MGI Symbol;Acc:MGI:1196337]	Brms1l	protein_coding
ENSMUSG00000012126	48.53146514	-1.795378638	0.616381188	0.000113032	0.006537211	Down	67586	UBX domain protein 11 [Source:MGI Symbol;Acc:MGI:1914836]	Ubxn11	protein_coding
ENSMUSG00000012405	3069.443254	-0.562680283	0.170534872	7.46E-05	0.005101815	Down	66480	ribosomal protein L15 [Source:MGI Symbol;Acc:MGI:1913730]	Rpl15	protein_coding
ENSMUSG00000012640	334.689404	0.393581214	0.152249504	0.001151728	0.023741292	Up	69930	zinc finger protein 715 [Source:MGI Symbol;Acc:MGI:1917180]	Zfp715	protein_coding
ENSMUSG00000013663	3750.270164	0.620000526	0.152197983	3.65E-06	0.000946988	Up	19211	phosphatase and tensin homolog [Source:MGI Symbol;Acc:MGI:109583]	Pten	protein_coding
ENSMUSG00000014294	1494.972209	-0.751706355	0.3098955	0.00066608	0.017670571	Down	17991	NADH:ubiquinone oxidoreductase subunit A2 [Source:MGI Symbol;Acc:MGI:1343103]	Ndufa2	protein_coding
ENSMUSG00000014418	1108.40694	0.235165478	0.08027155	0.001095863	0.023171785	Up	246694	HPS5, biogenesis of lysosomal organelles complex 2 subunit 2 [Source:MGI Symbol;Acc:MGI:2180307]	Hps5	protein_coding
ENSMUSG00000014776	784.5519163	-0.49068043	0.162738794	0.000221876	0.009224534	Down	78688	nucleolar protein 3 (apoptosis repressor with CARD domain) [Source:MGI Symbol;Acc:MGI:1925938]	Nol3	protein_coding
ENSMUSG00000014791	425.5586234	-0.361225571	0.134041602	0.001095333	0.023171785	Down	234683	engulfment and cell motility 3 [Source:MGI Symbol;Acc:MGI:2679007]	Elmo3	protein_coding
ENSMUSG00000014850	288.4860326	0.316575097	0.106936033	0.000581358	0.01626388	Up	17686	mutS homolog 3 [Source:MGI Symbol;Acc:MGI:109519]	Msh3	protein_coding
ENSMUSG00000015094	2745.824665	-0.301037687	0.109726949	0.001190625	0.024167305	Down	18146	neural proliferation, differentiation and control 1 [Source:MGI Symbol;Acc:MGI:1099802]	Npdc1	protein_coding
ENSMUSG00000015126	247.2272023	-0.400898559	0.146246997	0.00072056	0.018505817	Down	68327	TSR3 20S rRNA accumulation [Source:MGI Symbol;Acc:MGI:1915577]	Tsr3	protein_coding
ENSMUSG00000015214	711.1992002	0.363360473	0.138634907	0.0012458	0.024468519	Up	53332	myotubularin related protein 1 [Source:MGI Symbol;Acc:MGI:1858271]	Mtmr1	protein_coding
ENSMUSG00000015337	305.0974958	-0.693420229	0.161285511	1.24E-06	0.000546369	Down	13804	endonuclease G [Source:MGI Symbol;Acc:MGI:1261433]	Endog	protein_coding
ENSMUSG00000015354	1221.204305	-0.637346665	0.320220759	0.00213455	0.032697518	Down	76477	procollagen C-endopeptidase enhancer 2 [Source:MGI Symbol;Acc:MGI:1923727]	Pcolce2	protein_coding
ENSMUSG00000015478	440.4116365	-0.538120208	0.245870978	0.001810017	0.029736295	Down	54197	ring finger protein 5 [Source:MGI Symbol;Acc:MGI:1860076]	Rnf5	protein_coding
ENSMUSG00000015597	635.5190331	0.292299642	0.128667261	0.00448706	0.048662455	Up	57908	zinc finger protein 318 [Source:MGI Symbol;Acc:MGI:1889348]	Zfp318	protein_coding
ENSMUSG00000015749	1152.669682	0.269363577	0.092277334	0.000903879	0.020741077	Up	66471	acidic (leucine-rich) nuclear phosphoprotein 32 family, member E [Source:MGI Symbol;Acc:MGI:1913721]	Anp32e	protein_coding
ENSMUSG00000015755	1756.248569	0.35198642	0.102727321	9.79E-05	0.00598163	Up	68652	TGF-beta activated kinase 1/MAP3K7 binding protein 2 [Source:MGI Symbol;Acc:MGI:1915902]	Tab2	protein_coding
ENSMUSG00000015757	543.3670843	0.359752227	0.139312286	0.001319167	0.02526617	Up	67418	peptidylprolyl isomerase (cyclophilin)-like 4 [Source:MGI Symbol;Acc:MGI:1914668]	Ppil4	protein_coding
ENSMUSG00000015854	35.91751848	-0.068553699	0.188546045	0.003889502	0.045354164	Down	11801	CD5 antigen-like [Source:MGI Symbol;Acc:MGI:1334419]	Cd5l	protein_coding
ENSMUSG00000015943	263.6532546	-0.638770856	0.210505879	0.000149627	0.007539551	Down	69168	bolA-like 1 (E. coli) [Source:MGI Symbol;Acc:MGI:1916418]	Bola1	protein_coding
ENSMUSG00000015981	40.18941861	-1.729296926	0.577121875	9.40E-05	0.005856894	Down	57740	serine/threonine kinase 32C [Source:MGI Symbol;Acc:MGI:2385336]	Stk32c	protein_coding
ENSMUSG00000016018	899.056703	0.236843667	0.091680218	0.003042943	0.038930238	Up	72198	Mtr4 exosome RNA helicase [Source:MGI Symbol;Acc:MGI:1919448]	Mtrex	protein_coding
ENSMUSG00000016503	185.1660243	-0.389943048	0.190391907	0.004111875	0.04664907	Down	66596	general transcription factor III A [Source:MGI Symbol;Acc:MGI:1913846]	Gtf3a	protein_coding

ENSMUSG00000016758	34.17887409	-1.076232236	0.375342994	0.00017137	0.008025051	Down	12124	BCL2-interacting killer [Source:MGI Symbol;Acc:MGI:1206591]	Bik	protein_coding
ENSMUSG00000017167	283.9350292	0.420300372	0.175577575	0.00164414	0.028305152	Up	53321	contactin associated protein-like 1 [Source:MGI Symbol;Acc:MGI:1858201]	Cntnap1	protein_coding
ENSMUSG00000017309	1910.26232	0.517257264	0.233614794	0.001708412	0.028849913	Up	52685	CD300 molecule like family member G [Source:MGI Symbol;Acc:MGI:1289168]	Cd300lg	protein_coding
ENSMUSG00000017390	33.73934961	-1.188510457	0.584664038	0.001185219	0.024085936	Down	11676	aldolase C, fructose-bisphosphate [Source:MGI Symbol;Acc:MGI:101863]	Aldoc	protein_coding
ENSMUSG00000017417	54.50614044	-0.795398697	0.476024652	0.002952307	0.038485071	Down	72324	plexin domain containing 1 [Source:MGI Symbol;Acc:MGI:1919574]	Plxdc1	protein_coding
ENSMUSG00000017421	2759.15622	0.31294036	0.108975037	0.000641214	0.017324783	Up	22680	zinc finger protein 207 [Source:MGI Symbol;Acc:MGI:1340045]	Zfp207	protein_coding
ENSMUSG00000017493	19505.55611	-0.478942575	0.095471633	6.85E-08	9.84E-05	Down	16010	insulin-like growth factor binding protein 4 [Source:MGI Symbol;Acc:MGI:96439]	Igfbp4	protein_coding
ENSMUSG00000017548	786.2354811	0.480057982	0.119864129	5.80E-06	0.001281	Up	52615	SUZ12 polycomb repressive complex 2 subunit [Source:MGI Symbol;Acc:MGI:1261758]	Suz12	protein_coding
ENSMUSG00000017639	141.4982032	-1.941565199	0.530509747	9.40E-06	0.001688134	Down	268451	RAB11 family interacting protein 4 (class II) [Source:MGI Symbol;Acc:MGI:2442920]	Rab11fip4	protein_coding
ENSMUSG00000017652	51.22743198	-0.85504051	0.618780159	0.003983175	0.045835427	Down	21939	CD40 antigen [Source:MGI Symbol;Acc:MGI:88336]	Cd40	protein_coding
ENSMUSG00000017747	451.0499583	-0.263848168	0.1064127	0.003173037	0.040151016	Down	80860	GH3 domain containing [Source:MGI Symbol;Acc:MGI:1931556]	Ghdc	protein_coding
ENSMUSG00000017754	4383.646753	-0.382326684	0.167456596	0.002475191	0.03526538	Down	18830	phospholipid transfer protein [Source:MGI Symbol;Acc:MGI:103151]	Pltp	protein_coding
ENSMUSG00000017831	1270.332418	0.27653479	0.103963318	0.001786437	0.029521437	Up	271457	RAB5A, member RAS oncogene family [Source:MGI Symbol;Acc:MGI:105926]	Rab5a	protein_coding
ENSMUSG00000017832	6.531432879	-0.072383983	0.191115699	0.00271076	0.036812084	Down	75482	heat shock protein, alpha-crystallin-related, B9 [Source:MGI Symbol;Acc:MGI:1922732]	Hspb9	protein_coding
ENSMUSG00000017897	345.2520859	-0.593090312	0.183001475	8.37E-05	0.005465984	Down	14049	EYA transcriptional coactivator and phosphatase 2 [Source:MGI Symbol;Acc:MGI:109341]	Eya2	protein_coding
ENSMUSG00000018040	757.0509005	-0.336724255	0.122987685	0.000964737	0.021479733	Down	74778	ribosomal RNA processing 7 homolog A [Source:MGI Symbol;Acc:MGI:1922028]	Rrp7a	protein_coding
ENSMUSG00000018042	5890.981083	-0.270799272	0.097641178	0.001666947	0.028583577	Down	109754	cytochrome b5 reductase 3 [Source:MGI Symbol;Acc:MGI:94893]	Cyb5r3	protein_coding
ENSMUSG00000018166	112.8119182	-1.106695695	0.480808949	0.000703119	0.018275798	Down	13867	erb-b2 receptor tyrosine kinase 3 [Source:MGI Symbol;Acc:MGI:95411]	Erbb3	protein_coding
ENSMUSG00000018293	4261.840053	-0.436486543	0.210808882	0.003514661	0.042895291	Down	18643	profilin 1 [Source:MGI Symbol;Acc:MGI:97549]	Pfn1	protein_coding
ENSMUSG00000018372	245.979552	0.418118801	0.145600829	0.000453693	0.014062048	Up	320162	centrosomal protein 95 [Source:MGI Symbol;Acc:MGI:2443502]	Cep95	protein_coding
ENSMUSG00000018378	926.0075547	-0.580277733	0.187775122	0.000151814	0.007605279	Down	103841	CUE domain containing 1 [Source:MGI Symbol;Acc:MGI:2144281]	Cuedc1	protein_coding
ENSMUSG00000018401	825.2377278	0.426385329	0.159777356	0.000795603	0.019496604	Up	170749	myotubularin related protein 4 [Source:MGI Symbol;Acc:MGI:2180699]	Mtmr4	protein_coding
ENSMUSG00000018417	749.0151776	0.50743251	0.231306219	0.001939862	0.030905523	Up	17912	myosin IB [Source:MGI Symbol;Acc:MGI:107752]	Myo1b	protein_coding
ENSMUSG00000018433	383.8553608	0.331886563	0.136292321	0.002317218	0.034072198	Up	68979	nucleolar protein 11 [Source:MGI Symbol;Acc:MGI:1916229]	Nol11	protein_coding
ENSMUSG00000018500	43.75695911	-0.678793582	0.351473702	0.002294375	0.033880863	Down	11541	adenosine A2b receptor [Source:MGI Symbol;Acc:MGI:99403]	Adora2b	protein_coding
ENSMUSG00000018648	36.90937248	-1.037059733	0.484547194	0.001044726	0.022532874	Down	56405	dual specificity phosphatase 14 [Source:MGI Symbol;Acc:MGI:1927168]	Dusp14	protein_coding
ENSMUSG00000018669	1116.422097	-0.3210357	0.142381799	0.003766085	0.044759267	Down	80280	CDK5 regulatory subunit associated protein 3 [Source:MGI Symbol;Acc:MGI:1933126]	Cdk5rap3	protein_coding
ENSMUSG00000018740	782.70228	0.365561833	0.145016625	0.001508757	0.02699939	Up	71998	solute carrier family 25, member 35 [Source:MGI Symbol;Acc:MGI:1919248]	Slc25a35	protein_coding
ENSMUSG00000018796	23240.98076	0.629587044	0.181718469	3.67E-05	0.003584747	Up	14081	acyl-CoA synthetase long-chain family member 1 [Source:MGI Symbol;Acc:MGI:102797]	Acsl1	protein_coding
ENSMUSG00000018822	3621.894257	-1.712986523	0.558291704	7.34E-05	0.005101093	Down	54612	secreted frizzled-related sequence protein 5 [Source:MGI Symbol;Acc:MGI:1860298]	Sfrp5	protein_coding
ENSMUSG00000018846	11872.84664	0.611572402	0.161789235	1.16E-05	0.00193348	Up	211347	pantothenate kinase 3 [Source:MGI Symbol;Acc:MGI:2387464]	Pank3	protein_coding
ENSMUSG00000018849	205.6423792	-0.145807413	0.272935414	0.003552558	0.043008531	Down	211652	WW, C2 and coiled-coil domain containing 1 [Source:MGI Symbol;Acc:MGI:2388637]	Wwc1	protein_coding
ENSMUSG00000018865	16.65236874	-0.205328309	0.464039204	0.000831489	0.019736989	Down	29859	sulfotransferase family 4A, member 1 [Source:MGI Symbol;Acc:MGI:1888971]	Sult4a1	protein_coding
ENSMUSG00000018999	1274.270971	0.243554049	0.08835257	0.001732009	0.029034732	Up	58246	solute carrier family 35, member B4 [Source:MGI Symbol;Acc:MGI:1931249]	Slc35b4	protein_coding
ENSMUSG00000019054	1167.785083	-0.525118022	0.234190779	0.001669031	0.028590867	Down	66437	fission, mitochondrial 1 [Source:MGI Symbol;Acc:MGI:1913687]	Fis1	protein_coding
ENSMUSG00000019158	603.7398248	-0.723364073	0.289153211	0.000617225	0.016964332	Down	69094	transmembrane protein 160 [Source:MGI Symbol;Acc:MGI:1916344]	Tmem160	protein_coding
ENSMUSG00000019178	18.59141088	-1.010286566	0.460602319	0.000992301	0.02186742	Down	76571	serine/threonine/tyrosine interacting-like 1 [Source:MGI Symbol;Acc:MGI:1923821]	Styxl1	protein_coding
ENSMUSG00000019194	781.3656558	-0.489323924	0.191708256	0.000919387	0.020957408	Down	20266	sodium channel, voltage-gated, type I, beta [Source:MGI Symbol;Acc:MGI:98247]	Scn1b	protein_coding
ENSMUSG00000019303	24.57178284	-0.754160395	0.45842602	0.003305084	0.04130277	Down	19183	proteasome (prosome, macropain) 26S subunit, ATPase 3, interacting protein [Source:MGI Symbol;Acc:MGI:1098610]	Psmc3ip	protein_coding
ENSMUSG00000019312	54.1683705	-1.61286925	0.749926596	0.000802284	0.019540309	Down	14786	growth factor receptor bound protein 7 [Source:MGI Symbol;Acc:MGI:102683]	Grb7	protein_coding
ENSMUSG00000019362	821.5158324	-0.606628783	0.281095332	0.001698199	0.028804204	Down	101966	DNA segment, Chr 8, ERATO Doi 738, expressed [Source:MGI Symbol;Acc:MGI:1289231]	D8Ertd738e	protein_coding
ENSMUSG00000019370	3378.309945	-0.429513783	0.10249216	3.60E-06	0.000946988	Down	12315	calmodulin 3 [Source:MGI Symbol;Acc:MGI:103249]	Calm3	protein_coding
ENSMUSG00000019428	3579.617389	-0.397714252	0.160372093	0.001422719	0.026099657	Down	14232	FK506 binding protein 8 [Source:MGI Symbol;Acc:MGI:1341070]	Fkbp8	protein_coding
ENSMUSG00000019433	431.7084913	-0.824992936	0.230442014	1.87E-05	0.002550282	Down	67903	GIPC PDZ domain containing family, member 1 [Source:MGI Symbol;Acc:MGI:1926252]	Gipc1	protein_coding
ENSMUSG00000019461	850.6199617	-0.225760015	0.078594153	0.001183313	0.024075597	Down	70310	phospholipid scramblase 3 [Source:MGI Symbol;Acc:MGI:1917560]	Plscr3	protein_coding
ENSMUSG00000019467	350.1562822	-0.335037409	0.134262122	0.001882451	0.030289224	Down	52666	Rho guanine nucleotide exchange factor (GEF) 25 [Source:MGI Symbol;Acc:MGI:1277173]	Arhgef25	protein_coding
ENSMUSG00000019539	881.6851871	-0.451083052	0.244546509	0.00455698	0.049112217	Down	52377	reticulocalbin 3, EF-hand calcium binding domain [Source:MGI Symbol;Acc:MGI:1277122]	Rcn3	protein_coding
ENSMUSG00000019558	570.4451567	-0.544980075	0.258517854	0.00207076	0.032236135	Down	102857	solute carrier family 6 (neurotransmitter transporter, creatine), member 8 [Source:MGI Symbol;Acc:MGI:2147834]	Slc6a8	protein_coding
ENSMUSG00000019579	651.1640785	-0.406779545	0.172621154	0.001942243	0.030905523	Down	28106	myeloid derived growth factor [Source:MGI Symbol;Acc:MGI:2156020]	Mydgf	protein_coding
ENSMUSG00000019590	295.6067032	-0.780018899	0.27631946	0.000231629	0.009282919	Down	13056	cytochrome b-561 [Source:MGI Symbol;Acc:MGI:103253]	Cyb561	protein_coding
ENSMUSG00000019659	261.3480173	-0.831957617	0.284561719	0.000162796	0.007749885	Down	72654	coiled-coil domain containing 12 [Source:MGI Symbol;Acc:MGI:1919904]	Ccdc12	protein_coding

ENSMUSG00000019689	213.9600767	-0.538251718	0.271627192	0.002770664	0.03719373	Down	66117	formation of mitochondrial complex V assembly factor 1 [Source:MGI Symbol;Acc:MGI:1913367]	Fmc1	protein_coding
ENSMUSG00000019809	1119.532715	0.566515266	0.172273487	7.93E-05	0.005295104	Up	56535	peroxisomal biogenesis factor 3 [Source:MGI Symbol;Acc:MGI:1929646]	Pex3	protein_coding
ENSMUSG00000019810	638.5500645	-0.450057069	0.170030227	0.000760185	0.018990119	Down	66848	fucosidase, alpha-L- 2, plasma [Source:MGI Symbol;Acc:MGI:1914098]	Fuca2	protein_coding
ENSMUSG00000019851	113.0283895	-0.829432432	0.556283098	0.003579574	0.043228314	Down	64058	PERP, TP53 apoptosis effector [Source:MGI Symbol;Acc:MGI:1929938]	Perp	protein_coding
ENSMUSG00000019873	2149.083895	0.323099304	0.120489676	0.001319535	0.02526617	Up	28193	receptor accessory protein 3 [Source:MGI Symbol;Acc:MGI:88930]	Reep3	protein_coding
ENSMUSG00000019892	8.095427467	-0.053728936	0.180839465	0.00124538	0.024468519	Down	74978	leucine-rich repeats and IQ motif containing 1 [Source:MGI Symbol;Acc:MGI:1922228]	Lrriq1	protein_coding
ENSMUSG00000019917	689.1563535	0.273290131	0.092508226	0.000783961	0.019315327	Up	103080	septin 10 [Source:MGI Symbol;Acc:MGI:1918110]	Septin10	protein_coding
ENSMUSG00000019966	1208.939959	0.482404415	0.178703005	0.000616853	0.016964332	Up	17311	kit ligand [Source:MGI Symbol;Acc:MGI:96974]	Kitl	protein_coding
ENSMUSG00000019984	524.8043239	0.321089652	0.127293576	0.001991015	0.031391734	Up	70208	mediator complex subunit 23 [Source:MGI Symbol;Acc:MGI:1917458]	Med23	protein_coding
ENSMUSG00000020051	22.91281588	2.833988505	1.37921803	0.00081928	0.019680555	Up	18478	phenylalanine hydroxylase [Source:MGI Symbol;Acc:MGI:97473]	Pah	protein_coding
ENSMUSG00000020053	4639.718544	0.375561427	0.142588112	0.0011557	0.023794719	Up	16000	insulin-like growth factor 1 [Source:MGI Symbol;Acc:MGI:96432]	Igf1	protein_coding
ENSMUSG00000020063	411.5570242	0.334873964	0.154222114	0.003979487	0.045835427	Up	93759	sirtuin 1 [Source:MGI Symbol;Acc:MGI:2135607]	Sirt1	protein_coding
ENSMUSG00000020083	52.07790052	-0.856341449	0.486061478	0.00239675	0.03472722	Down	69894	family with sequence similarity 241, member B [Source:MGI Symbol;Acc:MGI:1917144]	Fam241b	protein_coding
ENSMUSG00000020098	287.2266628	-1.248929304	0.314818023	3.60E-06	0.000946988	Down	13180	pterin 4 alpha carbinolamine dehydratase/dimerization cofactor of hepatocyte nuclear factor 1 alpha (TCF1) 1 [Source:MGI Symbol;Acc:MGI:94873]	Pcbd1	protein_coding
ENSMUSG00000020107	1043.718596	-0.468574705	0.127350136	2.27E-05	0.002705768	Down	52717	anaphase promoting complex subunit 16 [Source:MGI Symbol;Acc:MGI:1289325]	Anapc16	protein_coding
ENSMUSG00000020114	1807.370535	0.231700395	0.074812001	0.000653112	0.017558636	Up	71902	cullin associated and neddylation disassociated 1 [Source:MGI Symbol;Acc:MGI:1261820]	Cand1	protein_coding
ENSMUSG00000020124	1178.499113	0.315319575	0.101987975	0.000382625	0.012631761	Up	14479	ubiquitin specific peptidase 15 [Source:MGI Symbol;Acc:MGI:101857]	Usp15	protein_coding
ENSMUSG00000020128	1170.46865	0.393215805	0.112063818	5.52E-05	0.004447279	Up	245944	VPS54 GARP complex subunit [Source:MGI Symbol;Acc:MGI:2178798]	Vps54	protein_coding
ENSMUSG00000020142	180.1196407	-0.650531934	0.260517097	0.000686105	0.017968169	Down	55963	solute carrier family 1 (glutamate/neutral amino acid transporter), member 4 [Source:MGI Symbol;Acc:MGI:2135601]	Slc1a4	protein_coding
ENSMUSG00000020150	972.3337633	-0.738118961	0.228840924	7.03E-05	0.00504748	Down	14431	guanidinoacetate methyltransferase [Source:MGI Symbol;Acc:MGI:1098221]	Gamt	protein_coding
ENSMUSG00000020163	1218.229872	-0.988524866	0.424929927	0.000707151	0.018307504	Down	66594	ubiquinol-cytochrome c reductase, complex III subunit XI [Source:MGI Symbol;Acc:MGI:1913844]	Uqcr11	protein_coding
ENSMUSG00000020166	871.3863141	0.200409461	0.077905519	0.004089391	0.046455158	Up	72068	CCR4-NOT transcription complex, subunit 2 [Source:MGI Symbol;Acc:MGI:1919318]	Cnot2	protein_coding
ENSMUSG00000020219	863.9918197	-0.684733481	0.307015837	0.001224897	0.024468519	Down	30055	translocase of inner mitochondrial membrane 13 [Source:MGI Symbol;Acc:MGI:1353432]	Timm13	protein_coding
ENSMUSG00000020230	441.2343307	-0.348364922	0.104114697	0.000129599	0.007045341	Down	15468	protein arginine N-methyltransferase 2 [Source:MGI Symbol;Acc:MGI:1316652]	Prmt2	protein_coding
ENSMUSG00000020246	491.7389234	0.242775354	0.087131185	0.001505945	0.026977079	Up	67933	host cell factor C2 [Source:MGI Symbol;Acc:MGI:1915183]	Hcfc2	protein_coding
ENSMUSG00000020260	868.0785794	-0.245286636	0.098923348	0.003863015	0.045255836	Down	80294	protein O-fucosyltransferase 2 [Source:MGI Symbol;Acc:MGI:1916863]	Pofut2	protein_coding
ENSMUSG00000020264	3194.258368	0.629821303	0.162314515	7.60E-06	0.001487719	Up	246049	solute carrier family 36 (proton/amino acid symporter), member 2 [Source:MGI Symbol;Acc:MGI:1891430]	Slc36a2	protein_coding
ENSMUSG00000020280	583.5175355	0.498725339	0.138109706	2.86E-05	0.003106886	Up	74467	pseudouridylate synthase 10 [Source:MGI Symbol;Acc:MGI:1921717]	Pus10	protein_coding
ENSMUSG00000020283	2148.290245	0.385589884	0.165235622	0.002275132	0.033800769	Up	72129	peroxisomal biogenesis factor 13 [Source:MGI Symbol;Acc:MGI:1919379]	Pex13	protein_coding
ENSMUSG00000020308	413.5519553	-0.599927855	0.237913209	0.000666504	0.017670571	Down	110012	tubulin polyglutamylase complex subunit 1 [Source:MGI Symbol;Acc:MGI:106618]	Tpgs1	protein_coding
ENSMUSG00000020325	180.2005375	-1.169263662	0.270049226	7.28E-07	0.000413505	Down	83554	follistatin-like 3 [Source:MGI Symbol;Acc:MGI:1890391]	Fstl3	protein_coding
ENSMUSG00000020358	2142.098055	-0.592005546	0.235616236	0.000727926	0.018535693	Down	15384	heterogeneous nuclear ribonucleoprotein A/B [Source:MGI Symbol;Acc:MGI:1330294]	Hnrnpab	protein_coding
ENSMUSG00000020364	92.9647781	0.474852472	0.255325168	0.004196827	0.04696359	Up	21408	zinc finger protein 354A [Source:MGI Symbol;Acc:MGI:103172]	Zfp354a	protein_coding
ENSMUSG00000020366	1714.357216	0.257109326	0.088617298	0.001000347	0.021977601	Up	26420	mitogen-activated protein kinase 9 [Source:MGI Symbol;Acc:MGI:1346862]	Mapk9	protein_coding
ENSMUSG00000020375	487.5136911	-0.223776081	0.077460508	0.001305264	0.025189625	Down	216724	RUN and FYVE domain containing 1 [Source:MGI Symbol;Acc:MGI:2429762]	Rufy1	protein_coding
ENSMUSG00000020388	283.0127422	-0.420073396	0.212361468	0.004184101	0.04696359	Down	30794	PDZ and LIM domain 4 [Source:MGI Symbol;Acc:MGI:1353470]	Pdlim4	protein_coding
ENSMUSG00000020441	193.9403322	-0.46160872	0.193087894	0.001408337	0.026068609	Down	67862	RIKEN cDNA 2310033P09 gene [Source:MGI Symbol;Acc:MGI:1915112]	2310033P09Rik	protein_coding
ENSMUSG00000020448	579.892723	0.306782116	0.092178943	0.000169406	0.007976426	Up	193670	ring finger protein 185 [Source:MGI Symbol;Acc:MGI:1922078]	Rnf185	protein_coding
ENSMUSG00000020459	582.3527273	0.271098337	0.091484282	0.000720416	0.018505817	Up	76784	mitochondrial translational initiation factor 2 [Source:MGI Symbol;Acc:MGI:1924034]	Mtif2	protein_coding
ENSMUSG00000020464	561.7273515	0.367594952	0.134236369	0.000865002	0.02019861	Up	71701	polyribonucleotide nucleotidyltransferase 1 [Source:MGI Symbol;Acc:MGI:1918951]	Pnpt1	protein_coding
ENSMUSG00000020475	19.33692126	-1.416235445	0.581868617	0.000490146	0.014636612	Down	56012	phosphoglycerate mutase 2 [Source:MGI Symbol;Acc:MGI:1933118]	Pgam2	protein_coding
ENSMUSG00000020532	20863.90195	0.908196928	0.369205348	0.000565555	0.016114087	Up	107476	acetyl-Coenzyme A carboxylase alpha [Source:MGI Symbol;Acc:MGI:108451]	Acaca	protein_coding
ENSMUSG00000020564	726.096833	0.327570865	0.117050922	0.00089277	0.020651151	Up	380753	ataxin 7-like 1 [Source:MGI Symbol;Acc:MGI:3584458]	Atxn7l1	protein_coding
ENSMUSG00000020566	53.15344479	-0.875316812	0.368872047	0.000711356	0.018379013	Down	68775	ATPase, H+ transporting, lysosomal V1 subunit C2 [Source:MGI Symbol;Acc:MGI:1916025]	Atp6v1c2	protein_coding
ENSMUSG00000020570	2667.110521	0.3210113	0.111448729	0.000744834	0.018765676	Up	19027	synaptophysin-like protein [Source:MGI Symbol;Acc:MGI:108081]	Sypl	protein_coding
ENSMUSG00000020572	2592.974017	0.408514536	0.181510982	0.002471563	0.03526538	Up	59027	nicotinamide phosphoribosyltransferase [Source:MGI Symbol;Acc:MGI:1929865]	Nampt	protein_coding
ENSMUSG00000020580	5373.24837	0.349654831	0.151559621	0.002900378	0.038239246	Up	19878	Rho-associated coiled-coil containing protein kinase 2 [Source:MGI Symbol;Acc:MGI:107926]	Rock2	protein_coding
ENSMUSG00000020590	1053.806052	0.309019758	0.074614073	7.43E-06	0.001472195	Up	217463	sorting nexin 13 [Source:MGI Symbol;Acc:MGI:2661416]	Snx13	protein_coding
ENSMUSG00000020594	2832.677516	0.352466155	0.106799302	0.000154879	0.007647655	Up	80913	pumilio RNA-binding family member 2 [Source:MGI Symbol;Acc:MGI:1931751]	Pum2	protein_coding
ENSMUSG00000020599	10.91626958	-2.36046254	0.718687717	3.94E-05	0.003733612	Down	19739	regulator of G-protein signaling 9 [Source:MGI Symbol;Acc:MGI:1338824]	Rgs9	protein_coding
ENSMUSG00000020611	1201.677393	0.212982549	0.08431259	0.004210715	0.047027379	Up	14674	guanine nucleotide binding protein, alpha 13 [Source:MGI Symbol;Acc:MGI:95768]	Gna13	protein_coding

ENSMUSG00000020623	97.24917508	-0.847199855	0.305074663	0.00025351	0.009817405	Down	26399	mitogen-activated protein kinase kinase 6 [Source:MGI Symbol;Acc:MGI:1346870]	Map2k6	protein_coding
ENSMUSG00000020635	25.48796768	-0.854381583	0.327225969	0.0004258	0.013588537	Down	14226	FK506 binding protein 1b [Source:MGI Symbol;Acc:MGI:1336205]	Fkbp1b	protein_coding
ENSMUSG00000020642	959.4361252	0.415414378	0.098968666	3.47E-06	0.000946988	Up	108089	ring finger protein 144A [Source:MGI Symbol;Acc:MGI:1344401]	Rnf144a	protein_coding
ENSMUSG00000020658	119.7765155	0.532908612	0.261016798	0.00249724	0.035419704	Up	668212	EFR3 homolog B [Source:MGI Symbol;Acc:MGI:2444851]	Efr3b	protein_coding
ENSMUSG00000020664	4490.714777	0.346124769	0.137250119	0.001728381	0.029034732	Up	13382	dihydrolipoamide dehydrogenase [Source:MGI Symbol;Acc:MGI:107450]	Dld	protein_coding
ENSMUSG00000020671	3303.118118	0.247839645	0.074184813	0.000258154	0.009930304	Up	19325	RAB10, member RAS oncogene family [Source:MGI Symbol;Acc:MGI:105066]	Rab10	protein_coding
ENSMUSG00000020687	1633.370901	0.401865304	0.109291089	3.15E-05	0.003266187	Up	217232	cell division cycle 27 [Source:MGI Symbol;Acc:MGI:102685]	Cdc27	protein_coding
ENSMUSG00000020694	736.3098949	0.230993264	0.081441655	0.001454701	0.02641609	Up	24086	tousled-like kinase 2 (Arabidopsis) [Source:MGI Symbol;Acc:MGI:1346023]	Tlk2	protein_coding
ENSMUSG00000020695	928.878388	-0.221307815	0.521277882	0.002515244	0.035528854	Down	17534	mannose receptor, C type 2 [Source:MGI Symbol;Acc:MGI:107818]	Mrc2	protein_coding
ENSMUSG00000020736	459.6014297	-0.544250723	0.234629248	0.001284659	0.024930783	Down	50773	5',3'-nucleotidase, cytosolic [Source:MGI Symbol;Acc:MGI:1354954]	Nt5c	protein_coding
ENSMUSG00000020766	302.6064216	-0.593135351	0.246548666	0.000981057	0.021758091	Down	14635	galactokinase 1 [Source:MGI Symbol;Acc:MGI:95730]	Galk1	protein_coding
ENSMUSG00000020782	243.9388525	-0.633324646	0.291265557	0.0015845	0.027693405	Down	217325	LLGL2 scribble cell polarity complex component [Source:MGI Symbol;Acc:MGI:1918843]	Llgl2	protein_coding
ENSMUSG00000020798	18.23090966	-0.813507767	0.487519186	0.003009067	0.038784775	Down	77577	spinster homolog 3 [Source:MGI Symbol;Acc:MGI:1924827]	Spns3	protein_coding
ENSMUSG00000020808	18.55709389	-0.227517181	0.554120941	0.002693049	0.036812084	Down	109212	PICALM interacting mitotic regulator [Source:MGI Symbol;Acc:MGI:1924434]	Pimreg	protein_coding
ENSMUSG00000020814	328.8119995	-0.445224937	0.211360697	0.0028677	0.037986425	Down	67622	matrix-remodelling associated 7 [Source:MGI Symbol;Acc:MGI:1914872]	Mxra7	protein_coding
ENSMUSG00000020844	410.5020807	-0.629869982	0.151943677	2.61E-06	0.000864464	Down	18230	nucleoredoxin [Source:MGI Symbol;Acc:MGI:109331]	Nxn	protein_coding
ENSMUSG00000020877	90.22068506	-0.75117322	0.19484204	6.96E-06	0.001444279	Down	217140	secernin 2 [Source:MGI Symbol;Acc:MGI:1343092]	Scrn2	protein_coding
ENSMUSG00000020878	12.40049181	-0.137983965	0.27116509	8.34E-06	0.001563424	Down	69297	leucine rich repeat containing 46 [Source:MGI Symbol;Acc:MGI:1916547]	Lrrc46	protein_coding
ENSMUSG00000020917	23919.501	1.008287138	0.499714584	0.001391889	0.02591718	Up	104112	ATP citrate lyase [Source:MGI Symbol;Acc:MGI:103251]	Acly	protein_coding
ENSMUSG00000020926	86.31859162	-1.615207381	0.674017023	0.000466613	0.014308079	Down	11488	a disintegrin and metallopeptidase domain 11 [Source:MGI Symbol;Acc:MGI:1098667]	Adam11	protein_coding
ENSMUSG00000020962	928.8988444	0.469952569	0.09754469	1.61E-07	0.000181415	Up	83602	general transcription factor II A, 1 [Source:MGI Symbol;Acc:MGI:1933277]	Gtf2a1	protein_coding
ENSMUSG00000020963	3979.172996	0.290067696	0.089023315	0.000266515	0.010072064	Up	22095	thyroid stimulating hormone receptor [Source:MGI Symbol;Acc:MGI:98849]	Tshr	protein_coding
ENSMUSG00000021033	14552.70968	0.24987891	0.103635997	0.004050841	0.046199963	Up	14874	glutathione transferase zeta 1 (maleylacetoacetate isomerase) [Source:MGI Symbol;Acc:MGI:1341859]	Gstz1	protein_coding
ENSMUSG00000021193	1473.962805	0.392382361	0.108885941	4.41E-05	0.004044798	Up	69617	pitrilysin metallepetidase 1 [Source:MGI Symbol;Acc:MGI:1916867]	Pitrm1	protein_coding
ENSMUSG00000021203	82.55325431	-0.471719682	0.208934371	0.001881838	0.030289224	Down	68149	OTU domain, ubiquitin aldehyde binding 2 [Source:MGI Symbol;Acc:MGI:1915399]	Otub2	protein_coding
ENSMUSG00000021209	172.5807193	0.814024954	0.255988209	7.87E-05	0.005295104	Up	74521	protein phosphatase 4, regulatory subunit 4 [Source:MGI Symbol;Acc:MGI:1921771]	Ppp4r4	protein_coding
ENSMUSG00000021238	12038.82254	0.438243461	0.213172243	0.003314788	0.041394011	Up	104776	aldehyde dehydrogenase family 6, subfamily A1 [Source:MGI Symbol;Acc:MGI:1915077]	Aldh6a1	protein_coding
ENSMUSG00000021257	186.4377602	-0.344326887	0.150371091	0.00282421	0.03761176	Down	68737	angel homolog 1 [Source:MGI Symbol;Acc:MGI:1915987]	Angel1	protein_coding
ENSMUSG00000021282	5271.434215	0.34317019	0.098783332	7.63E-05	0.005194396	Up	217869	eukaryotic translation initiation factor 5 [Source:MGI Symbol;Acc:MGI:95309]	Eif5	protein_coding
ENSMUSG00000021288	1402.663676	-0.257038748	0.108096161	0.004478328	0.048662455	Down	16593	kinesin light chain 1 [Source:MGI Symbol;Acc:MGI:107978]	Klc1	protein_coding
ENSMUSG00000021327	1108.047674	0.480875257	0.186897597	0.000890532	0.020651151	Up	72739	zinc finger with KRAB and SCAN domains 3 [Source:MGI Symbol;Acc:MGI:1919989]	Zkscan3	protein_coding
ENSMUSG00000021339	738.8899281	0.265240746	0.080194075	0.000273249	0.010102419	Up	380836	MRS2 magnesium transporter [Source:MGI Symbol;Acc:MGI:2685748]	Mrs2	protein_coding
ENSMUSG00000021363	17.93211409	-2.27644276	0.967451622	0.000458036	0.014147708	Down	17152	male germ cell-associated kinase [Source:MGI Symbol;Acc:MGI:96913]	Mak	protein_coding
ENSMUSG00000021371	3082.291486	0.295025281	0.082831217	8.84E-05	0.00570255	Up	76137	mitochondrial calcium uniporter regulator 1 [Source:MGI Symbol;Acc:MGI:1923387]	Mcur1	protein_coding
ENSMUSG00000021379	75.53400591	-0.650892912	0.414724931	0.004159402	0.046948364	Down	15904	inhibitor of DNA binding 4 [Source:MGI Symbol;Acc:MGI:99414]	Id4	protein_coding
ENSMUSG00000021384	87.08215708	-1.327627145	0.349381917	6.19E-06	0.001334103	Down	66329	sushi domain containing 3 [Source:MGI Symbol;Acc:MGI:1913579]	Susd3	protein_coding
ENSMUSG00000021408	821.9576586	0.248919047	0.089511385	0.00156746	0.027507171	Up	19766	receptor (TNFRSF)-interacting serine-threonine kinase 1 [Source:MGI Symbol;Acc:MGI:108212]	Ripk1	protein_coding
ENSMUSG00000021411	694.9728526	-0.661031379	0.179800619	1.54E-05	0.002273816	Down	66895	PX domain containing 1 [Source:MGI Symbol;Acc:MGI:1914145]	Pxdc1	protein_coding
ENSMUSG00000021456	39.33415572	-1.866372614	0.69972419	0.000225913	0.009224534	Down	14120	fructose bisphosphatase 2 [Source:MGI Symbol;Acc:MGI:95491]	Fbp2	protein_coding
ENSMUSG00000021470	689.9117866	0.355671523	0.13147311	0.001021038	0.022244687	Up	76251	excision repair cross-complementing rodent repair deficiency, complementation group 6 like 2 [Source:MGI Symbol;Acc:MGI:1923501]	Ercc6l2	protein_coding
ENSMUSG00000021474	4043.969763	0.676432647	0.304064722	0.001268326	0.024669378	Up	14057	sideroflexin 1 [Source:MGI Symbol;Acc:MGI:2137677]	Sfxn1	protein_coding
ENSMUSG00000021483	181.4711744	-0.600842191	0.268591987	0.001393883	0.02591718	Down	105278	cyclin-dependent kinase 20 [Source:MGI Symbol;Acc:MGI:2145349]	Cdk20	protein_coding
ENSMUSG00000021493	954.4360639	-0.581512207	0.23998141	0.000926329	0.021087761	Down	67399	PDZ and LIM domain 7 [Source:MGI Symbol;Acc:MGI:1914649]	Pdlim7	protein_coding
ENSMUSG00000021496	181.7328304	-0.687312075	0.296480824	0.000951102	0.021369409	Down	72562	pterin 4 alpha carbinolamine dehydratase/dimerization cofactor of hepatocyte nuclear factor 1 alpha (TCF1) 2 [Source:MGI Symbol;Acc:MGI:1919812]	Pcbd2	protein_coding
ENSMUSG00000021540	1437.91039	0.187134097	0.072200091	0.004219482	0.047094779	Up	17129	SMAD family member 5 [Source:MGI Symbol;Acc:MGI:1328787]	Smad5	protein_coding
ENSMUSG00000021549	979.3954226	0.307869999	0.123451374	0.002311679	0.034019775	Up	218397	RAS p21 protein activator 1 [Source:MGI Symbol;Acc:MGI:97860]	Rasa1	protein_coding
ENSMUSG00000021565	8.350453289	-0.04629207	0.177444351	0.003966705	0.045835427	Down	74338	solute carrier family 6 (neurotransmitter transporter), member 19 [Source:MGI Symbol;Acc:MGI:1921588]	Slc6a19	protein_coding
ENSMUSG00000021573	1301.477548	0.850272838	0.393855122	0.00114982	0.02373035	Up	72948	tubulin polymerization promoting protein [Source:MGI Symbol;Acc:MGI:1920198]	Tppp	protein_coding
ENSMUSG00000021583	1329.047281	0.322909861	0.137574694	0.002862247	0.037986425	Up	80898	endoplasmic reticulum aminopeptidase 1 [Source:MGI Symbol;Acc:MGI:1933403]	Erap1	protein_coding
ENSMUSG00000021606	525.6306926	-0.567571583	0.266629703	0.001873472	0.030289224	Down	407785	NADH:ubiquinone oxidoreductase core subunit S6 [Source:MGI Symbol;Acc:MGI:107932]	Ndufs6	protein_coding
ENSMUSG00000021638	12.47509251	-0.069958016	0.189890923	0.001352149	0.02566253	Down	18260	occludin [Source:MGI Symbol;Acc:MGI:106183]	Ocln	protein_coding
ENSMUSG00000021666	794.0249872	0.269552753	0.09697215	0.001307626	0.025189625	Up	320806	G elongation factor, mitochondrial 2 [Source:MGI Symbol;Acc:MGI:2444783]	Gfm2	protein_coding
ENSMUSG00000021693	561.1520346	0.280032944	0.106490706	0.001937676	0.030905523	Up	16563	kinesin family member 2A [Source:MGI Symbol;Acc:MGI:108390]	Kif2a	protein_coding

ENSMUSG00000021713	182.1663124	0.451699739	0.137874328	0.000109981	0.006430038	Up	238831	peptidylprolyl isomerase domain and WD repeat containing 1 [Source:MGI Symbol;Acc:MGI:2443069]	Ppwd1	protein_coding
ENSMUSG00000021714	28.62137191	0.55703344	0.313524032	0.003825263	0.045022918	Up	60411	centromere protein K [Source:MGI Symbol;Acc:MGI:1926210]	Cenpk	protein_coding
ENSMUSG00000021731	479.8558662	0.432628525	0.219074517	0.004088704	0.046455158	Up	59054	mitochondrial ribosomal protein S30 [Source:MGI Symbol;Acc:MGI:1926237]	Mrps30	protein_coding
ENSMUSG00000021748	4715.443712	0.587695436	0.2177777	0.000444236	0.013919129	Up	68263	pyruvate dehydrogenase (lipoamide) beta [Source:MGI Symbol;Acc:MGI:1915513]	Pdhb	protein_coding
ENSMUSG00000021770	623.0288728	0.341656271	0.099630228	0.000100468	0.006096337	Up	67630	sterile alpha motif domain containing 8 [Source:MGI Symbol;Acc:MGI:1914880]	Samd8	protein_coding
ENSMUSG00000021779	564.9682754	0.778247675	0.149905329	1.13E-08	3.65E-05	Up	21834	thyroid hormone receptor beta [Source:MGI Symbol;Acc:MGI:98743]	Thrb	protein_coding
ENSMUSG00000021786	621.2367271	0.256258693	0.086961492	0.000894478	0.020653966	Up	71147	3-oxoacyl-ACP synthase, mitochondrial [Source:MGI Symbol;Acc:MGI:1918397]	Oxsm	protein_coding
ENSMUSG00000021796	2329.952048	0.304456262	0.109460555	0.001121928	0.023417446	Up	12166	bone morphogenetic protein receptor, type 1A [Source:MGI Symbol;Acc:MGI:1338938]	Bmpr1a	protein_coding
ENSMUSG00000021840	858.25303	0.274119958	0.108341258	0.002582239	0.036119909	Up	218975	mitogen-activated protein kinase 1 interacting protein 1-like [Source:MGI Symbol;Acc:MGI:2444022]	Mapk1ip1l	protein_coding
ENSMUSG00000021870	1687.054452	0.329975062	0.147723389	0.003875591	0.045341523	Up	83997	sarcolemma associated protein [Source:MGI Symbol;Acc:MGI:1933549]	Slmap	protein_coding
ENSMUSG00000021911	540.5429557	0.251577623	0.103511318	0.004016038	0.045941738	Up	26430	poly (ADP-ribose) glycohydrolase [Source:MGI Symbol;Acc:MGI:1347094]	Parg	protein_coding
ENSMUSG00000021913	98.0469085	-1.478537261	0.523740817	0.000158257	0.007675767	Down	239017	oxoglutarate dehydrogenase-like [Source:MGI Symbol;Acc:MGI:3616088]	Ogdhl	protein_coding
ENSMUSG00000021929	1320.75015	0.248850173	0.098309406	0.003173312	0.040151016	Up	16648	karyopherin (importin) alpha 3 [Source:MGI Symbol;Acc:MGI:1100863]	Kpna3	protein_coding
ENSMUSG00000021930	512.2283416	0.364003899	0.132844359	0.000836234	0.019767938	Up	66674	SPRY domain containing 7 [Source:MGI Symbol;Acc:MGI:1913924]	Spryd7	protein_coding
ENSMUSG00000021945	883.5227591	0.409773206	0.153815341	0.000802793	0.019540309	Up	76007	zinc finger, MYM-type 2 [Source:MGI Symbol;Acc:MGI:1923257]	Zmym2	protein_coding
ENSMUSG00000021957	18459.60062	0.717766635	0.348274993	0.001677823	0.028711005	Up	21881	transketolase [Source:MGI Symbol;Acc:MGI:105992]	Tkt	protein_coding
ENSMUSG00000022020	244.4438053	0.47307585	0.156478336	0.000238661	0.009465746	Up	66897	N(alpha)-acetyltransferase 16, NatA auxiliary subunit [Source:MGI Symbol;Acc:MGI:1914147]	Naa16	protein_coding
ENSMUSG00000022025	10.7003881	-0.108197981	0.221881	0.002654429	0.036638703	Down	16840	chondromodulin [Source:MGI Symbol;Acc:MGI:1341171]	Cnmd	protein_coding
ENSMUSG00000022053	828.2174562	0.574502292	0.236639249	0.000985165	0.021758091	Up	13592	early B cell factor 2 [Source:MGI Symbol;Acc:MGI:894332]	Ebf2	protein_coding
ENSMUSG00000022090	390.2726368	-0.481869605	0.25706052	0.003918751	0.045585372	Down	213019	PDZ and LIM domain 2 [Source:MGI Symbol;Acc:MGI:2384850]	Pdlim2	protein_coding
ENSMUSG00000022100	1231.647019	0.333600071	0.118567118	0.000831024	0.019736989	Up	65246	exportin 7 [Source:MGI Symbol;Acc:MGI:1929705]	Xpo7	protein_coding
ENSMUSG00000022105	1883.468753	0.50717326	0.11288796	6.86E-07	0.000407937	Up	19645	RB transcriptional corepressor 1 [Source:MGI Symbol;Acc:MGI:97874]	Rb1	protein_coding
ENSMUSG00000022119	727.4270233	0.297967641	0.122771904	0.002952414	0.038485071	Up	74213	RNA binding motif protein 26 [Source:MGI Symbol;Acc:MGI:1921463]	Rbm26	protein_coding
ENSMUSG00000022129	45.89788194	0.616309018	0.391540203	0.004539428	0.049102004	Up	13190	dopachrome tautomerase [Source:MGI Symbol;Acc:MGI:102563]	Dct	protein_coding
ENSMUSG00000022160	230.9188605	-0.282123911	0.102631559	0.001259767	0.024669378	Down	56335	methyltransferase like 3 [Source:MGI Symbol;Acc:MGI:1927165]	Mettl3	protein_coding
ENSMUSG00000022177	129.4989633	-0.493853486	0.204307764	0.001169853	0.024013736	Down	219072	HAUS augmin-like complex, subunit 4 [Source:MGI Symbol;Acc:MGI:1261794]	Haus4	protein_coding
ENSMUSG00000022199	484.1859129	-0.813981978	0.170844926	1.15E-07	0.000152499	Down	59049	solute carrier family 22 (organic cation transporter), member 17 [Source:MGI Symbol;Acc:MGI:1926225]	Slc22a17	protein_coding
ENSMUSG00000022200	1654.395348	0.253146159	0.103534635	0.003858284	0.045255836	Up	66629	golgi phosphoprotein 3 [Source:MGI Symbol;Acc:MGI:1913879]	Golph3	protein_coding
ENSMUSG00000022201	2323.297223	0.415744505	0.120968324	7.37E-05	0.005101093	Up	22763	zinc finger RNA binding protein [Source:MGI Symbol;Acc:MGI:1341890]	Zfr	protein_coding
ENSMUSG00000022203	213.6027892	-0.52669632	0.295026629	0.003955084	0.045753568	Down	13644	embryonal Fyn-associated substrate [Source:MGI Symbol;Acc:MGI:105311]	Efs	protein_coding
ENSMUSG00000022217	715.2803507	-0.489579361	0.190863696	0.000810224	0.019613515	Down	85308	ER membrane protein complex subunit 9 [Source:MGI Symbol;Acc:MGI:1934682]	Emc9	protein_coding
ENSMUSG00000022228	1298.819719	0.389496226	0.14888521	0.001068704	0.022737013	Up	432731	zinc finger and SCAN domain containing 26 [Source:MGI Symbol;Acc:MGI:3531417]	Zscan26	protein_coding
ENSMUSG00000022307	1673.829261	0.272029459	0.083929564	0.000303642	0.010744702	Up	170719	oxidation resistance 1 [Source:MGI Symbol;Acc:MGI:2179326]	Oxr1	protein_coding
ENSMUSG00000022309	267.6447156	1.303606196	0.456609067	0.000158566	0.007675767	Up	11600	angiopoietin 1 [Source:MGI Symbol;Acc:MGI:108448]	Angpt1	protein_coding
ENSMUSG00000022332	187.6693927	-0.422090632	0.184668129	0.002099243	0.0325458	Down	13992	KH domain containing, RNA binding, signal transduction associated 3 [Source:MGI Symbol;Acc:MGI:1313312]	Khdrbs3	protein_coding
ENSMUSG00000022401	917.3211026	0.36410234	0.138044556	0.001171884	0.024013736	Up	321003	X-prolyl aminopeptidase 3, mitochondrial [Source:MGI Symbol;Acc:MGI:2445217]	Xpnpep3	protein_coding
ENSMUSG00000022419	2996.202082	0.438642345	0.188905026	0.001990682	0.031391734	Up	97998	DEP domain containing MTOR-interacting protein [Source:MGI Symbol;Acc:MGI:2146322]	Deptor	protein_coding
ENSMUSG00000022420	283.7535255	-0.444814132	0.137479432	0.000128977	0.007036523	Down	54152	dynein, axonemal, light chain 4 [Source:MGI Symbol;Acc:MGI:1859217]	Dnal4	protein_coding
ENSMUSG00000022428	179.3010643	-0.492521264	0.189963091	0.000759233	0.018990119	Down	73739	chibby family member 1, beta catenin antagonist [Source:MGI Symbol;Acc:MGI:1920989]	Cby1	protein_coding
ENSMUSG00000022453	982.4464785	-0.336240645	0.124065022	0.001005563	0.022038737	Down	17939	N-acetyl galactosaminidase, alpha [Source:MGI Symbol;Acc:MGI:1261422]	Naga	protein_coding
ENSMUSG00000022476	349.6665787	-0.381695781	0.106561229	4.85E-05	0.004178515	Down	78929	polymerase (RNA) III (DNA directed) polypeptide H [Source:MGI Symbol;Acc:MGI:1926179]	Polr3h	protein_coding
ENSMUSG00000022501	6.800026191	-0.00337219	0.16820346	6.09E-06	0.001329454	Down	19118	protamine 1 [Source:MGI Symbol;Acc:MGI:97765]	Prm1	protein_coding
ENSMUSG00000022508	1362.257731	0.853215434	0.424465081	0.001572398	0.027565759	Up	12053	B cell leukemia/lymphoma 6 [Source:MGI Symbol;Acc:MGI:107187]	Bcl6	protein_coding
ENSMUSG00000022540	465.2147299	-0.359676746	0.170950744	0.00427971	0.0474291	Down	66049	rogdi homolog [Source:MGI Symbol;Acc:MGI:1913299]	Rogdi	protein_coding
ENSMUSG00000022554	149.5033573	-0.400994178	0.196261431	0.003980467	0.045835427	Down	59053	HGH1 homolog [Source:MGI Symbol;Acc:MGI:1930628]	Hgh1	protein_coding
ENSMUSG00000022555	4064.474165	0.36496297	0.152616099	0.002396673	0.03472722	Up	13350	diacylglycerol O-acyltransferase 1 [Source:MGI Symbol;Acc:MGI:1333825]	Dgat1	protein_coding
ENSMUSG00000022564	11046.25769	-0.240467152	0.098171775	0.004160051	0.046948364	Down	66168	glutamate receptor, ionotropic, N-methyl D-aspartate-associated protein 1 (glutamate binding) [Source:MGI Symbol;Acc:MGI:1913418]	Grina	protein_coding
ENSMUSG00000022594	527.2326701	-0.388102435	0.184206532	0.003446444	0.042548859	Down	23936	Ly6/neurotoxin 1 [Source:MGI Symbol;Acc:MGI:1345180]	Lynx1	protein_coding
ENSMUSG00000022601	277.9166958	0.356782018	0.137777075	0.00131234	0.025189625	Up	271377	zinc finger and BTB domain containing 11 [Source:MGI Symbol;Acc:MGI:2443876]	Zbtb11	protein_coding
ENSMUSG00000022615	23.73152136	-0.175370756	0.333845393	0.003795315	0.044920786	Down	72962	thymidine phosphorylase [Source:MGI Symbol;Acc:MGI:1920212]	Tymp	protein_coding
ENSMUSG00000022698	2333.18064	0.319239416	0.12400093	0.001757533	0.029263352	Up	72117	N(alpha)-acetyltransferase 50, NatE catalytic subunit [Source:MGI Symbol;Acc:MGI:1919367]	Naa50	protein_coding
ENSMUSG00000022704	218.0278396	0.337180709	0.144816114	0.002958546	0.038508028	Up	106248	queuine tRNA-ribosyltransferase accessory subunit 2 [Source:MGI Symbol;Acc:MGI:1922194]	Qtrt2	protein_coding

ENSMUSG00000022707	2264.866668	0.714946781	0.185988318	8.35E-06	0.001563424	Up	74185	glucan (1,4-alpha-), branching enzyme 1 [Source:MGI Symbol;Acc:MGI:1921435]	Gbe1	protein_coding
ENSMUSG00000022747	383.630558	0.490227081	0.227690401	0.002281542	0.033800769	Up	54613	ST3 beta-galactoside alpha-2,3-sialyltransferase 6 [Source:MGI Symbol;Acc:MGI:1888707]	St3gal6	protein_coding
ENSMUSG00000022769	282.5675341	-0.602545414	0.314541662	0.002664159	0.036638703	Down	64136	stromal cell-derived factor 2-like 1 [Source:MGI Symbol;Acc:MGI:2149842]	Sdf2l1	protein_coding
ENSMUSG00000022781	2495.644796	0.263373532	0.064848831	1.39E-05	0.002132486	Up	224105	p21 (RAC1) activated kinase 2 [Source:MGI Symbol;Acc:MGI:1339984]	Pak2	protein_coding
ENSMUSG00000022789	1641.469405	0.392564275	0.123954514	0.000188777	0.00835522	Up	74006	dynamin 1-like [Source:MGI Symbol;Acc:MGI:1921256]	Dnm1l	protein_coding
ENSMUSG00000022811	1117.60455	0.301194204	0.124901834	0.002920819	0.038296458	Up	22661	zinc finger protein 148 [Source:MGI Symbol;Acc:MGI:1332234]	Zfp148	protein_coding
ENSMUSG00000022853	1476.521484	0.679819441	0.218234689	0.000110071	0.006430038	Up	74147	enoyl-Coenzyme A, hydratase/3-hydroxyacyl Coenzyme A dehydrogenase [Source:MGI Symbol;Acc:MGI:1277964]	Ehhadh	protein_coding
ENSMUSG00000022887	132.6073719	0.772646474	0.497049478	0.00363445	0.043615929	Up	17174	mannan-binding lectin serine peptidase 1 [Source:MGI Symbol;Acc:MGI:88492]	Masp1	protein_coding
ENSMUSG00000022897	815.1493678	0.232220838	0.087369451	0.002508948	0.035468996	Up	13548	dual-specificity tyrosine-(Y)-phosphorylation regulated kinase 1a [Source:MGI Symbol;Acc:MGI:1330299]	Dyrk1a	protein_coding
ENSMUSG00000022905	1119.833933	0.300069388	0.119696864	0.002358981	0.034480332	Up	16646	karyopherin (importin) alpha 1 [Source:MGI Symbol;Acc:MGI:103560]	Kpna1	protein_coding
ENSMUSG00000022972	121.4153855	-0.59912416	0.233411927	0.000629745	0.01711734	Down	68001	cilia and flagella associate protien 298 [Source:MGI Symbol;Acc:MGI:1915251]	Cfap298	protein_coding
ENSMUSG00000023025	1140.395223	0.2974908	0.128265333	0.003938558	0.045675085	Up	207214	La ribonucleoprotein domain family, member 4 [Source:MGI Symbol;Acc:MGI:2443114]	Larp4	protein_coding
ENSMUSG00000023033	19.91838976	-0.157962572	0.303684335	0.000819416	0.019680555	Down	20273	sodium channel, voltage-gated, type VIII, alpha [Source:MGI Symbol;Acc:MGI:103169]	Scn8a	protein_coding
ENSMUSG00000023045	8.422166842	-1.021532212	0.788812693	0.004494017	0.048662455	Down	223920	sterol O-acyltransferase 2 [Source:MGI Symbol;Acc:MGI:1332226]	Soat2	protein_coding
ENSMUSG00000023064	13165.6407	-1.184143469	0.24459642	6.35E-08	9.84E-05	Down	20618	synuclein, gamma [Source:MGI Symbol;Acc:MGI:1298397]	Sncg	protein_coding
ENSMUSG00000023067	1536.86555	-0.901284637	0.519744483	0.002353667	0.034434609	Down	12575	cyclin-dependent kinase inhibitor 1A (P21) [Source:MGI Symbol;Acc:MGI:104556]	Cdkn1a	protein_coding
ENSMUSG00000023074	722.9795419	0.468121754	0.18370374	0.000950666	0.021369409	Up	70380	motile sperm domain containing 1 [Source:MGI Symbol;Acc:MGI:1917630]	Mospd1	protein_coding
ENSMUSG00000023078	232.8069489	-0.076409013	0.194335553	0.00114649	0.023690413	Down	55985	chemokine (C-X-C motif) ligand 13 [Source:MGI Symbol;Acc:MGI:1888499]	Cxcl13	protein_coding
ENSMUSG00000023243	86.61888281	-0.779672993	0.548355892	0.004070429	0.046331377	Down	16529	potassium channel, subfamily K, member 5 [Source:MGI Symbol;Acc:MGI:1336175]	Kcnk5	protein_coding
ENSMUSG00000023262	132.2506746	-0.431009625	0.178273586	0.001489447	0.026877103	Down	109652	aminoacylase 1 [Source:MGI Symbol;Acc:MGI:87913]	Acy1	protein_coding
ENSMUSG00000023367	1010.041537	-0.513504789	0.255547976	0.00270415	0.036812084	Down	66058	transmembrane protein 176A [Source:MGI Symbol;Acc:MGI:1913308]	Tmem176a	protein_coding
ENSMUSG00000023467	16.54505983	-1.236795557	0.76148813	0.002366787	0.034565124	Down	56734	tubby-like protein 2 [Source:MGI Symbol;Acc:MGI:1861600]	Tulp2	protein_coding
ENSMUSG00000023832	1845.109376	0.492993482	0.190232846	0.000769705	0.019073957	Up	110460	acetyl-Coenzyme A acetyltransferase 2 [Source:MGI Symbol;Acc:MGI:87871]	Acat2	protein_coding
ENSMUSG00000023861	1784.97846	0.716429924	0.232114834	0.000123159	0.006868632	Up	55951	mitochondrial pyruvate carrier 1 [Source:MGI Symbol;Acc:MGI:1915240]	Mpc1	protein_coding
ENSMUSG00000023873	28.91570047	-0.881231009	0.56903102	0.003294747	0.041233388	Down	66931	RIKEN cDNA 1700010I14 gene [Source:MGI Symbol;Acc:MGI:1914181]	1700010I14Rik	protein_coding
ENSMUSG00000023905	270.2201344	-0.650055435	0.342138731	0.002495379	0.035419704	Down	27279	tumor necrosis factor receptor superfamily, member 12a [Source:MGI Symbol;Acc:MGI:1351484]	Tnfrsf12a	protein_coding
ENSMUSG00000023911	19.66618479	-1.16211614	0.406773389	0.000167314	0.007913736	Down	76917	FLYWCH family member 2 [Source:MGI Symbol;Acc:MGI:1924167]	Flywch2	protein_coding
ENSMUSG00000023921	3263.115456	0.242349547	0.093329999	0.002804758	0.0374395	Up	17850	methylmalonyl-Coenzyme A mutase [Source:MGI Symbol;Acc:MGI:97239]	Mmut	protein_coding
ENSMUSG00000023930	20.38793105	-0.112689105	0.231335122	2.26E-05	0.002705768	Down	22024	cysteine-rich secretory protein 2 [Source:MGI Symbol;Acc:MGI:98815]	Crisp2	protein_coding
ENSMUSG00000023939	411.4270919	-0.628472025	0.247371834	0.000617186	0.016964332	Down	68463	mitochondrial ribosomal protein L14 [Source:MGI Symbol;Acc:MGI:1333864]	Mrpl14	protein_coding
ENSMUSG00000023961	756.8163312	0.386457316	0.122753307	0.00025054	0.009765934	Up	224794	ectonucleotide pyrophosphatase/phosphodiesterase 4 [Source:MGI Symbol;Acc:MGI:2682634]	Enpp4	protein_coding
ENSMUSG00000023972	170.5101705	-1.037355458	0.274392631	7.40E-06	0.001472195	Down	71461	PTK7 protein tyrosine kinase 7 [Source:MGI Symbol;Acc:MGI:1918711]	Ptk7	protein_coding
ENSMUSG00000024006	2059.759732	0.306395888	0.082679228	4.65E-05	0.004130681	Up	106504	serine/threonine kinase 38 [Source:MGI Symbol;Acc:MGI:2442572]	Stk38	protein_coding
ENSMUSG00000024011	1416.470121	-0.52941147	0.258668986	0.002467175	0.03526538	Down	74116	peptidase inhibitor 16 [Source:MGI Symbol;Acc:MGI:1921366]	Pi16	protein_coding
ENSMUSG00000024012	3717.231356	-0.446672836	0.143202338	0.000180978	0.008185824	Down	56462	mitochondrial carrier 1 [Source:MGI Symbol;Acc:MGI:1929261]	Mtch1	protein_coding
ENSMUSG00000024033	29.6612365	-0.140393193	0.274810847	2.46E-05	0.002828234	Down	22092	radial spoke head 1 homolog (Chlamydomonas) [Source:MGI Symbol;Acc:MGI:1194909]	Rsph1	protein_coding
ENSMUSG00000024039	155.1989236	-0.649822081	0.306602702	0.001577139	0.027620776	Down	12411	cystathionine beta-synthase [Source:MGI Symbol;Acc:MGI:88285]	Cbs	protein_coding
ENSMUSG00000024053	2604.644979	-0.595687631	0.253098152	0.001159338	0.023841127	Down	246707	elastin microfibril interfacer 2 [Source:MGI Symbol;Acc:MGI:2389136]	Emilin2	protein_coding
ENSMUSG00000024059	71.90609693	-0.900149097	0.312046416	0.000177158	0.008185484	Down	78785	CAP-GLY domain containing linker protein family, member 4 [Source:MGI Symbol;Acc:MGI:1919100]	Clip4	protein_coding
ENSMUSG00000024064	13.46218986	-0.067163925	0.188041134	0.001777205	0.029514117	Down	71685	polypeptide N-acetylgalactosaminyltransferase 14 [Source:MGI Symbol;Acc:MGI:1918935]	Galnt14	protein_coding
ENSMUSG00000024076	345.4353225	-0.523295962	0.266060179	0.003026575	0.0388775	Down	74199	vitrin [Source:MGI Symbol;Acc:MGI:1921449]	Vit	protein_coding
ENSMUSG00000024120	1279.69553	0.437793074	0.136483812	0.000149111	0.007535595	Up	72416	leucine-rich PPR-motif containing [Source:MGI Symbol;Acc:MGI:1919666]	Lrpprc	protein_coding
ENSMUSG00000024122	1949.709839	0.275432026	0.106379168	0.002105244	0.03255949	Up	18607	3-phosphoinositide dependent protein kinase 1 [Source:MGI Symbol;Acc:MGI:1338068]	Pdpk1	protein_coding
ENSMUSG00000024165	965.5082399	-0.576307377	0.232014453	0.000836073	0.019767938	Down	52009	Jupiter microtubule associated homolog 2 [Source:MGI Symbol;Acc:MGI:1196260]	Jpt2	protein_coding
ENSMUSG00000024186	31.44281229	-1.08341042	0.59974333	0.001937606	0.030905523	Down	50782	regulator of G-protein signaling 11 [Source:MGI Symbol;Acc:MGI:1354739]	Rgs11	protein_coding
ENSMUSG00000024197	1284.486033	-0.36381823	0.14409612	0.001593294	0.027818888	Down	66905	perilipin 3 [Source:MGI Symbol;Acc:MGI:1914155]	Plin3	protein_coding
ENSMUSG00000024208	351.7128502	-0.569140214	0.182228697	0.000122119	0.00685497	Down	67267	ubiquinol-cytochrome c reductase complex assembly factor 2 [Source:MGI Symbol;Acc:MGI:1914517]	Uqcc2	protein_coding
ENSMUSG00000024228	624.3727066	0.552111606	0.148926616	1.99E-05	0.002566184	Up	67993	nudix (nucleoside diphosphate linked moiety X)-type motif 12 [Source:MGI Symbol;Acc:MGI:1915243]	Nudt12	protein_coding
ENSMUSG00000024231	759.8919462	0.326799268	0.121439538	0.001208042	0.024318448	Up	71745	cullin 2 [Source:MGI Symbol;Acc:MGI:1918995]	Cul2	protein_coding
ENSMUSG00000024234	642.7600829	0.351587734	0.114659607	0.00033899	0.011545099	Up	67440	mitochondrial poly(A) polymerase [Source:MGI Symbol;Acc:MGI:1914690]	Mtpap	protein_coding
ENSMUSG00000024238	965.9070918	0.308017546	0.133041047	0.003428994	0.042478059	Up	21417	zinc finger E-box binding homeobox 1 [Source:MGI Symbol;Acc:MGI:1344313]	Zeb1	protein_coding
ENSMUSG00000024241	715.0232845	0.45018646	0.144143333	0.000187578	0.00835522	Up	20662	SOS Ras/Rac guanine nucleotide exchange factor 1 [Source:MGI Symbol;Acc:MGI:98354]	Sos1	protein_coding

ENSMUSG00000024242	1370.98625	0.422360699	0.108961706	1.28E-05	0.002036641	Up	225028	mitogen-activated protein kinase kinase kinase 3 [Source:MGI Symbol;Acc:MGI:2154405]	Map4k3	protein_coding
ENSMUSG00000024258	338.6580644	-0.365069095	0.155469408	0.00243018	0.034957676	Down	69241	polymerase (RNA) II (DNA directed) polypeptide D [Source:MGI Symbol;Acc:MGI:1916491]	Polr2d	protein_coding
ENSMUSG00000024259	1329.295618	0.324860915	0.132970276	0.002443102	0.035055764	Up	67453	solute carrier family 25, member 46 [Source:MGI Symbol;Acc:MGI:1914703]	Slc25a46	protein_coding
ENSMUSG00000024283	1560.01708	0.339705283	0.108174742	0.000286376	0.010411628	Up	225131	WW domain containing adaptor with coiled-coil [Source:MGI Symbol;Acc:MGI:2387357]	Wac	protein_coding
ENSMUSG00000024290	1448.774112	0.311158743	0.135255795	0.003769602	0.044767499	Up	19877	Rho-associated coiled-coil containing protein kinase 1 [Source:MGI Symbol;Acc:MGI:107927]	Rock1	protein_coding
ENSMUSG00000024294	1651.711326	0.227223287	0.090765203	0.004183282	0.04696359	Up	225164	MIB E3 ubiquitin protein ligase 1 [Source:MGI Symbol;Acc:MGI:2443157]	Mib1	protein_coding
ENSMUSG00000024309	330.7467141	-0.820965186	0.357980744	0.000821045	0.019680555	Down	14976	prefoldin subunit 6 [Source:MGI Symbol;Acc:MGI:95908]	Pfdn6	protein_coding
ENSMUSG00000024317	348.2600152	0.286106522	0.111189758	0.002281307	0.033800769	Up	56515	ring finger protein 138 [Source:MGI Symbol;Acc:MGI:1929211]	Rnf138	protein_coding
ENSMUSG00000024352	70.94232911	-1.023489008	0.335422142	9.77E-05	0.00598163	Down	71242	spermatogenesis associated 24 [Source:MGI Symbol;Acc:MGI:1918492]	Spata24	protein_coding
ENSMUSG00000024359	8907.052745	0.331041772	0.11583777	0.000748806	0.01881074	Up	15526	heat shock protein 9 [Source:MGI Symbol;Acc:MGI:96245]	Hspa9	protein_coding
ENSMUSG00000024360	2907.314686	0.270041503	0.106590967	0.002753155	0.037110507	Up	225363	eukaryotic translation termination factor 1 [Source:MGI Symbol;Acc:MGI:2385071]	Etf1	protein_coding
ENSMUSG00000024404	2536.590948	0.301589718	0.109537693	0.001179431	0.024053404	Up	66878	RIO kinase 3 [Source:MGI Symbol;Acc:MGI:1914128]	Riok3	protein_coding
ENSMUSG00000024431	4066.294426	0.333401233	0.142729267	0.003096769	0.039386907	Up	14815	nuclear receptor subfamily 3, group C, member 1 [Source:MGI Symbol;Acc:MGI:95824]	Nr3c1	protein_coding
ENSMUSG00000024491	533.1973007	0.471223316	0.122984616	1.15E-05	0.00193348	Up	225432	RNA binding motif protein 27 [Source:MGI Symbol;Acc:MGI:2147194]	Rbm27	protein_coding
ENSMUSG00000024521	67.46230769	-0.711263673	0.327291345	0.00133976	0.025483542	Down	58801	phorbol-12-myristate-13-acetate-induced protein 1 [Source:MGI Symbol;Acc:MGI:1930146]	Pmaip1	protein_coding
ENSMUSG00000024530	17.53565676	0.958666573	0.615043884	0.00309139	0.039374662	Up	225655	PRELI domain containing 3A [Source:MGI Symbol;Acc:MGI:2442865]	Prelid3a	protein_coding
ENSMUSG00000024533	704.7669138	0.382667265	0.15942455	0.002047416	0.031973755	Up	68166	spire type actin nucleation factor 1 [Source:MGI Symbol;Acc:MGI:1915416]	Spire1	protein_coding
ENSMUSG00000024603	1433.596135	0.247391659	0.095067154	0.002619873	0.036337993	Up	67665	dynactin 4 [Source:MGI Symbol;Acc:MGI:1914915]	Dctn4	protein_coding
ENSMUSG00000024639	2176.972845	0.28950227	0.111585559	0.001996216	0.031444956	Up	14682	guanine nucleotide binding protein, alpha q polypeptide [Source:MGI Symbol;Acc:MGI:95776]	Gnaq	protein_coding
ENSMUSG00000024644	1801.348994	-0.576621124	0.164167107	3.17E-05	0.003266187	Down	66054	CNDP dipeptidase 2 (metallopeptidase M20 family) [Source:MGI Symbol;Acc:MGI:1913304]	Cndp2	protein_coding
ENSMUSG00000024726	364.4810239	0.306196648	0.130488148	0.003329887	0.041522393	Up	67383	carnosine N-methyltransferase 1 [Source:MGI Symbol;Acc:MGI:1914633]	Carnmt1	protein_coding
ENSMUSG00000024759	5837.585262	0.341127239	0.095915729	6.26E-05	0.004826458	Up	109168	atlastin GTPase 3 [Source:MGI Symbol;Acc:MGI:1924270]	Atl3	protein_coding
ENSMUSG00000024767	869.1359937	-0.301294038	0.118859335	0.002153885	0.0328768	Down	107260	OTU domain, ubiquitin aldehyde binding 1 [Source:MGI Symbol;Acc:MGI:2147616]	Otub1	protein_coding
ENSMUSG00000024790	208.393128	-0.398371908	0.188722976	0.003616275	0.043549456	Down	66406	SAC3 domain containing 1 [Source:MGI Symbol;Acc:MGI:1913656]	Sac3d1	protein_coding
ENSMUSG00000024811	3022.70558	0.346809297	0.110510584	0.000275775	0.010154752	Up	74493	tankyrase, TRF1-interacting ankyrin-related ADP-ribose polymerase 2 [Source:MGI Symbol;Acc:MGI:1921743]	Tnks2	protein_coding
ENSMUSG00000024867	12.08135177	-0.130265561	0.251168936	0.001483824	0.026860016	Down	18719	phosphatidylinositol-4-phosphate 5-kinase, type 1 beta [Source:MGI Symbol;Acc:MGI:107930]	Pip5k1b	protein_coding
ENSMUSG00000024885	347.1992702	-0.822963713	0.273312421	0.0001283	0.007036523	Down	67689	aldehyde dehydrogenase 3 family, member B1 [Source:MGI Symbol;Acc:MGI:1914939]	Aldh3b1	protein_coding
ENSMUSG00000024892	31271.38495	0.502731469	0.251587509	0.002973443	0.038516848	Up	18563	pyruvate carboxylase [Source:MGI Symbol;Acc:MGI:97520]	Pcx	protein_coding
ENSMUSG00000024908	1784.217031	0.36164423	0.104009423	8.07E-05	0.00531048	Up	52036	protein phosphatase 6, regulatory subunit 3 [Source:MGI Symbol;Acc:MGI:1921807]	Ppp6r3	protein_coding
ENSMUSG00000024909	440.8506275	-0.475124631	0.15172335	0.000163369	0.007755767	Down	58859	epidermal growth factor-containing fibulin-like extracellular matrix protein 2 [Source:MGI Symbol;Acc:MGI:1891209]	Efemp2	protein_coding
ENSMUSG00000024925	267.4079065	-0.538212024	0.320270356	0.00455266	0.049112217	Down	68209	ribonuclease H2, subunit C [Source:MGI Symbol;Acc:MGI:1915459]	Rnaseh2c	protein_coding
ENSMUSG00000024959	426.1721954	-0.499896101	0.157921184	0.000144108	0.007505743	Down	12015	BCL2-associated agonist of cell death [Source:MGI Symbol;Acc:MGI:1096330]	Bad	protein_coding
ENSMUSG00000024972	5275.43333	0.803678838	0.193483733	1.80E-06	0.000720041	Up	56072	lectin, galactose binding, soluble 12 [Source:MGI Symbol;Acc:MGI:1929094]	Lgals12	protein_coding
ENSMUSG00000024976	1038.47394	0.343544936	0.129639278	0.001228202	0.024468519	Up	56392	Shoc2, leucine rich repeat scaffold protein [Source:MGI Symbol;Acc:MGI:1927197]	Shoc2	protein_coding
ENSMUSG00000024985	571.0161687	0.610112305	0.156016942	7.12E-06	0.001460938	Up	21416	transcription factor 7 like 2, T cell specific, HMG box [Source:MGI Symbol;Acc:MGI:1202879]	Tcf7l2	protein_coding
ENSMUSG00000024993	705.4909261	0.370932657	0.125994753	0.000451468	0.014018295	Up	67894	DENN domain containing 10 [Source:MGI Symbol;Acc:MGI:1915144]	Dennd10	protein_coding
ENSMUSG00000025024	552.8064067	0.368079098	0.118892004	0.000283003	0.010332602	Up	76479	survival motor neuron domain containing 1 [Source:MGI Symbol;Acc:MGI:1923729]	Smndc1	protein_coding
ENSMUSG00000025035	377.0234908	-0.636592773	0.275222979	0.001093462	0.023171785	Down	56350	ADP-ribosylation factor-like 3 [Source:MGI Symbol;Acc:MGI:1929699]	Arl3	protein_coding
ENSMUSG00000025059	353.8114569	-0.98268941	0.448669339	0.000957784	0.021461264	Down	14933	glycerol kinase [Source:MGI Symbol;Acc:MGI:106594]	Gk	protein_coding
ENSMUSG00000025104	319.5968704	0.335387411	0.151503856	0.003825502	0.045022918	Up	29877	HDGF like 3 [Source:MGI Symbol;Acc:MGI:1352760]	Hdgfl3	protein_coding
ENSMUSG00000025161	65.14725034	-0.911511531	0.316458201	0.000177391	0.008185484	Down	80879	solute carrier family 16 (monocarboxylic acid transporters), member 3 [Source:MGI Symbol;Acc:MGI:1933438]	Slc16a3	protein_coding
ENSMUSG00000025165	5.858577462	-0.063425193	0.186045899	0.000744042	0.018765676	Down	209588	secreted and transmembrane 1A [Source:MGI Symbol;Acc:MGI:2384805]	Sectm1a	protein_coding
ENSMUSG00000025171	393.4448288	-0.512678409	0.227609629	0.001664303	0.028566663	Down	226122	ubiquitin domain containing 1 [Source:MGI Symbol;Acc:MGI:2385092]	Ubtd1	protein_coding
ENSMUSG00000025184	408.4037293	0.298838614	0.114002235	0.001697783	0.028804204	Up	52013	R3H domain and coiled-coil containing 1 like [Source:MGI Symbol;Acc:MGI:1196316]	R3hcc1l	protein_coding
ENSMUSG00000025199	1060.705598	0.331076965	0.106474003	0.000333716	0.011403904	Up	12675	conserved helix-loop-helix ubiquitous kinase [Source:MGI Symbol;Acc:MGI:99484]	Chuk	protein_coding
ENSMUSG00000025226	96.00255075	-0.522479004	0.292440908	0.003989621	0.045835427	Down	68431	F-box and leucine-rich repeat protein 15 [Source:MGI Symbol;Acc:MGI:1915681]	Fbxl15	protein_coding
ENSMUSG00000025234	2220.121082	0.313491935	0.106220878	0.000549981	0.015743889	Up	23806	ariadne RBR E3 ubiquitin protein ligase 1 [Source:MGI Symbol;Acc:MGI:1344363]	Arih1	protein_coding
ENSMUSG00000025235	163.8496466	-0.467686803	0.158807629	0.000300069	0.0107062	Down	102774	Bardet-Biedl syndrome 4 (human) [Source:MGI Symbol;Acc:MGI:2143311]	Bbs4	protein_coding
ENSMUSG00000025268	612.0716238	-0.330792009	0.11970462	0.00094775	0.021358851	Down	80884	MAGE family member D2 [Source:MGI Symbol;Acc:MGI:1933391]	Maged2	protein_coding
ENSMUSG00000025271	838.1534471	0.822380243	0.178980306	2.38E-07	0.000233309	Up	18639	6-phosphofructo-2-kinase/fructose-2,6-biphosphatase 1 [Source:MGI Symbol;Acc:MGI:107816]	Pfkfb1	protein_coding
ENSMUSG00000025326	1367.776172	0.327092775	0.122599785	0.001308046	0.025189625	Up	22215	ubiquitin protein ligase E3A [Source:MGI Symbol;Acc:MGI:105098]	Ube3a	protein_coding
ENSMUSG00000025330	46.85555892	-1.961543306	0.747871034	0.000251804	0.009773282	Down	18602	peptidyl arginine deiminase, type IV [Source:MGI Symbol;Acc:MGI:1338898]	Padi4	protein_coding

ENSMUSG00000025362	4022.798676	-0.475739435	0.257185598	0.004187551	0.04696359	Down	27370	ribosomal protein S26 [Source:MGI Symbol;Acc:MGI:1351628]	Rps26	protein_coding
ENSMUSG00000025371	362.6078441	-0.442424577	0.227868183	0.004014471	0.045941738	Down	208092	charged multivesicular body protein 6 [Source:MGI Symbol;Acc:MGI:3583942]	Chmp6	protein_coding
ENSMUSG00000025403	429.7110587	-0.429014799	0.110053835	1.13E-05	0.00193348	Down	108037	serine hydroxymethyltransferase 2 (mitochondrial) [Source:MGI Symbol;Acc:MGI:1277989]	Shmt2	protein_coding
ENSMUSG00000025407	36.25675004	-1.447209703	0.430909076	3.23E-05	0.003305157	Down	14632	GLI-Kruppel family member GLI1 [Source:MGI Symbol;Acc:MGI:95727]	Gli1	protein_coding
ENSMUSG00000025504	129.8593609	-0.913555332	0.548501118	0.002710265	0.036812084	Down	98845	EPS8-like 2 [Source:MGI Symbol;Acc:MGI:2138828]	Eps8l2	protein_coding
ENSMUSG00000025512	675.7830735	-0.327740919	0.147680274	0.003759416	0.044714298	Down	68038	chitinase domain containing 1 [Source:MGI Symbol;Acc:MGI:1915288]	Chid1	protein_coding
ENSMUSG00000025537	365.8417624	0.425784261	0.179221609	0.001615481	0.028120795	Up	18682	phosphorylase kinase gamma 1 [Source:MGI Symbol;Acc:MGI:97579]	Phkg1	protein_coding
ENSMUSG00000025572	486.0056746	-0.633293363	0.17498085	2.00E-05	0.002566184	Down	217353	transmembrane channel-like gene family 6 [Source:MGI Symbol;Acc:MGI:1098686]	Tmc6	protein_coding
ENSMUSG00000025609	1530.841156	0.412509172	0.097129523	2.98E-06	0.000921546	Up	27418	muskelin 1, intracellular mediator containing kelch motifs [Source:MGI Symbol;Acc:MGI:1351638]	Mkln1	protein_coding
ENSMUSG00000025742	530.7741509	-0.444427625	0.198929349	0.002111734	0.032569253	Down	110639	phosphoribosyl pyrophosphate synthetase 2 [Source:MGI Symbol;Acc:MGI:97776]	Prps2	protein_coding
ENSMUSG00000025759	897.513356	0.277115251	0.104503574	0.001740628	0.029122562	Up	72175	major facilitator superfamily domain containing 8 [Source:MGI Symbol;Acc:MGI:1919425]	Mfsd8	protein_coding
ENSMUSG00000025764	1546.891149	0.451900192	0.150361972	0.000298629	0.010690053	Up	269424	jade family PHD finger 1 [Source:MGI Symbol;Acc:MGI:1925835]	Jade1	protein_coding
ENSMUSG00000025784	2872.193563	-0.730890557	0.185059622	4.85E-06	0.001194835	Down	21922	C-type lectin domain family 3, member b [Source:MGI Symbol;Acc:MGI:104540]	Clec3b	protein_coding
ENSMUSG00000025790	595.8532567	-0.496575707	0.245276614	0.003059699	0.03902871	Down	108116	solute carrier organic anion transporter family, member 3a1 [Source:MGI Symbol;Acc:MGI:1351867]	Slco3a1	protein_coding
ENSMUSG00000025816	418.5916034	0.386811847	0.158378844	0.001630574	0.028229163	Up	57743	Sec61, alpha subunit 2 (S. cerevisiae) [Source:MGI Symbol;Acc:MGI:1931071]	Sec61a2	protein_coding
ENSMUSG00000025856	284.8095061	-0.388281952	0.195445811	0.004581606	0.049276724	Down	18590	platelet derived growth factor, alpha [Source:MGI Symbol;Acc:MGI:97527]	Pdgfa	protein_coding
ENSMUSG00000025858	884.8993388	-0.244754969	0.091111448	0.001681454	0.028711005	Down	67604	golgi to ER traffic protein 4 [Source:MGI Symbol;Acc:MGI:1914854]	Get4	protein_coding
ENSMUSG00000025860	2065.085611	0.413312431	0.124691073	0.000107382	0.006425415	Up	11798	X-linked inhibitor of apoptosis [Source:MGI Symbol;Acc:MGI:107572]	Xiap	protein_coding
ENSMUSG00000025862	2024.450011	0.335048328	0.130447964	0.001738453	0.029114442	Up	20843	stromal antigen 2 [Source:MGI Symbol;Acc:MGI:1098583]	Stag2	protein_coding
ENSMUSG00000025875	1748.970847	-0.578043936	0.257776957	0.001419312	0.026099657	Down	74257	tetraspanin 17 [Source:MGI Symbol;Acc:MGI:1921507]	Tspan17	protein_coding
ENSMUSG00000025898	420.1933233	0.334346228	0.114554115	0.000578303	0.016231111	Up	244672	CWF19-like 2, cell cycle control (S. pombe) [Source:MGI Symbol;Acc:MGI:1918023]	Cwf19l2	protein_coding
ENSMUSG00000025911	3950.350478	0.383635896	0.139535282	0.000770352	0.019073957	Up	76187	alcohol dehydrogenase, iron containing, 1 [Source:MGI Symbol;Acc:MGI:1923437]	Adhfe1	protein_coding
ENSMUSG00000025937	1441.637104	0.359592764	0.140359157	0.001445656	0.026307269	Up	212442	lactamase, beta 2 [Source:MGI Symbol;Acc:MGI:2442551]	Lactb2	protein_coding
ENSMUSG00000025958	791.4112587	0.264203509	0.089230833	0.000825446	0.019702098	Up	12912	cAMP responsive element binding protein 1 [Source:MGI Symbol;Acc:MGI:88494]	Creb1	protein_coding
ENSMUSG00000025968	2610.934319	0.322932297	0.125864965	0.001883706	0.030289224	Up	227197	NADH:ubiquinone oxidoreductase core subunit S1 [Source:MGI Symbol;Acc:MGI:2443241]	Ndufs1	protein_coding
ENSMUSG00000025982	4873.88479	0.276712841	0.101498929	0.00153169	0.027183952	Up	81898	splicing factor 3b, subunit 1 [Source:MGI Symbol;Acc:MGI:1932339]	Sf3b1	protein_coding
ENSMUSG00000025991	59.00190915	0.042603634	0.175958063	0.00439411	0.048144948	Up	227231	carbamoyl-phosphate synthetase 1 [Source:MGI Symbol;Acc:MGI:891996]	Cps1	protein_coding
ENSMUSG00000026082	272.5289977	0.411349273	0.172665604	0.001755389	0.029255918	Up	56210	REV1, DNA directed polymerase [Source:MGI Symbol;Acc:MGI:1929074]	Rev1	protein_coding
ENSMUSG00000026100	12.45517262	-0.140640342	0.273101629	0.000132823	0.007086499	Down	17700	myostatin [Source:MGI Symbol;Acc:MGI:95691]	Mstn	protein_coding
ENSMUSG00000026112	3255.649726	0.289307058	0.103181453	0.001201265	0.024267616	Up	76178	cytochrome C oxidase assembly factor 5 [Source:MGI Symbol;Acc:MGI:1923428]	Coa5	protein_coding
ENSMUSG00000026150	1323.63352	0.281107886	0.100818437	0.001175652	0.024035335	Up	75734	mitochondrial fission factor [Source:MGI Symbol;Acc:MGI:1922984]	Mff	protein_coding
ENSMUSG00000026223	2678.762948	-0.610989916	0.149964645	3.42E-06	0.000946988	Down	64294	integral membrane protein 2C [Source:MGI Symbol;Acc:MGI:1927594]	Itm2c	protein_coding
ENSMUSG00000026229	2433.761342	0.346343471	0.102164761	0.000160489	0.007725435	Up	70247	proteasome (prosome, macropain) 26S subunit, non-ATPase, 1 [Source:MGI Symbol;Acc:MGI:1917497]	Psmd1	protein_coding
ENSMUSG00000026259	27.75192861	-1.859032696	0.567934281	3.78E-05	0.003617499	Down	53972	neuronal guanine nucleotide exchange factor [Source:MGI Symbol;Acc:MGI:1858414]	Ngef	protein_coding
ENSMUSG00000026349	981.9723164	0.386932838	0.114129694	9.54E-05	0.005868571	Up	72949	cyclin T2 [Source:MGI Symbol;Acc:MGI:1920199]	Ccnt2	protein_coding
ENSMUSG00000026361	974.5567798	0.27892917	0.11055108	0.002600226	0.036231825	Up	214498	cell division cycle 73, Paf1/RNA polymerase II complex component [Source:MGI Symbol;Acc:MGI:2384876]	Cdc73	protein_coding
ENSMUSG00000026384	912.095903	0.546562292	0.117872909	3.22E-07	0.000241407	Up	19258	protein tyrosine phosphatase, non-receptor type 4 [Source:MGI Symbol;Acc:MGI:1099792]	Ptpn4	protein_coding
ENSMUSG00000026390	41.79040025	-0.093826967	0.209930224	5.76E-05	0.004534424	Down	17167	macrophage receptor with collagenous structure [Source:MGI Symbol;Acc:MGI:1309998]	Marco	protein_coding
ENSMUSG00000026398	34.58477519	0.694143842	0.372416184	0.002556161	0.035883103	Up	26424	nuclear receptor subfamily 5, group A, member 2 [Source:MGI Symbol;Acc:MGI:1346834]	Nr5a2	protein_coding
ENSMUSG00000026411	431.021036	-0.488871203	0.121391252	5.48E-06	0.001275029	Down	66241	transmembrane protein 9 [Source:MGI Symbol;Acc:MGI:1913491]	Tmem9	protein_coding
ENSMUSG00000026413	25.27831423	-0.090733352	0.204731817	0.003508436	0.042853972	Down	18772	plakophilin 1 [Source:MGI Symbol;Acc:MGI:1328359]	Pkp1	protein_coding
ENSMUSG00000026414	8.995990196	-0.033485118	0.173034057	0.00269946	0.036812084	Down	21956	troponin T2, cardiac [Source:MGI Symbol;Acc:MGI:104597]	Tnnt2	protein_coding
ENSMUSG00000026421	2846.616915	-0.494586696	0.097807081	4.40E-08	8.42E-05	Down	13007	cysteine and glycine-rich protein 1 [Source:MGI Symbol;Acc:MGI:88549]	Csrp1	protein_coding
ENSMUSG00000026434	1712.535133	0.456056804	0.081194296	2.35E-09	1.01E-05	Up	98415	nuclear casein kinase and cyclin-dependent kinase substrate 1 [Source:MGI Symbol;Acc:MGI:1934811]	Nucks1	protein_coding
ENSMUSG00000026466	1877.566611	0.359651384	0.106654863	0.000119349	0.006812419	Up	208263	torsin A interacting protein 1 [Source:MGI Symbol;Acc:MGI:3582693]	Tor1aip1	protein_coding
ENSMUSG00000026471	910.7077361	0.30648153	0.133855791	0.003928181	0.04561614	Up	15064	major histocompatibility complex, class I-related [Source:MGI Symbol;Acc:MGI:1195463]	Mr1	protein_coding
ENSMUSG00000026479	82.08589021	-0.683380521	0.477929234	0.0044946	0.048662455	Down	16782	laminin, gamma 2 [Source:MGI Symbol;Acc:MGI:99913]	Lamc2	protein_coding
ENSMUSG00000026491	1017.632533	0.356652814	0.135678378	0.001235849	0.024468519	Up	226747	AT hook containing transcription factor 1 [Source:MGI Symbol;Acc:MGI:1915033]	Ahctf1	protein_coding
ENSMUSG00000026492	645.5370515	0.412099723	0.149692728	0.00066126	0.017650281	Up	15278	transcription factor B2, mitochondrial [Source:MGI Symbol;Acc:MGI:107937]	Tfb2m	protein_coding
ENSMUSG00000026494	16.04614036	-0.149647711	0.28095976	0.002756985	0.037118067	Down	269152	kinesin family member 26B [Source:MGI Symbol;Acc:MGI:2447076]	Kif26b	protein_coding
ENSMUSG00000026520	506.4636159	-0.559288491	0.154152488	2.29E-05	0.002706968	Down	69051	pyrroline-5-carboxylate reductase family, member 2 [Source:MGI Symbol;Acc:MGI:1277956]	Pycr2	protein_coding
ENSMUSG00000026546	32.71621576	-0.945066448	0.773217182	0.004232584	0.047149397	Down	71870	cilia and flagella associated protein 45 [Source:MGI Symbol;Acc:MGI:1919120]	Cfap45	protein_coding
ENSMUSG00000026571	1094.819982	0.249064109	0.087019733	0.001236126	0.024468519	Up	74106	DDB1 and CUL4 associated factor 6 [Source:MGI Symbol;Acc:MGI:1921356]	Dcaf6	protein_coding

ENSMUSG00000026574	6625.685042	-0.314490159	0.142607981	0.004163676	0.046958532	Down	56429	dermatopontin [Source:MGI Symbol;Acc:MGI:1928392]	Dpt	protein_coding
ENSMUSG00000026618	2575.069302	0.334557053	0.103809779	0.000273232	0.010102419	Up	381314	isoleucine-tRNA synthetase 2, mitochondrial [Source:MGI Symbol;Acc:MGI:1919586]	Iars2	protein_coding
ENSMUSG00000026622	57.0634697	-1.01154864	0.370486505	0.000251048	0.009765934	Down	18005	NIMA (never in mitosis gene a)-related expressed kinase 2 [Source:MGI Symbol;Acc:MGI:109359]	Nek2	protein_coding
ENSMUSG00000026634	927.1473478	0.211316815	0.077913856	0.002507894	0.035468996	Up	52477	angel homolog 2 [Source:MGI Symbol;Acc:MGI:1196310]	Angel2	protein_coding
ENSMUSG00000026643	702.0022875	0.323282385	0.111852814	0.000671979	0.017734889	Up	18108	N-myristoyltransferase 2 [Source:MGI Symbol;Acc:MGI:1202298]	Nmt2	protein_coding
ENSMUSG00000026649	106.3503742	-0.713786743	0.38549047	0.002435424	0.034974724	Down	75472	cilia and flagella associated protein 126 [Source:MGI Symbol;Acc:MGI:1922722]	Cfap126	protein_coding
ENSMUSG00000026650	6.480408663	-0.034980228	0.173493803	0.00180411	0.02969459	Down	104362	meiosis expressed gene 1 [Source:MGI Symbol;Acc:MGI:1202878]	Meig1	protein_coding
ENSMUSG00000026739	1030.656048	0.426967723	0.116777703	3.16E-05	0.003266187	Up	12151	Bmi1 polycomb ring finger oncogene [Source:MGI Symbol;Acc:MGI:88174]	Bmi1	protein_coding
ENSMUSG00000026743	595.1739119	0.352288317	0.159595183	0.003787694	0.044892246	Up	17354	myeloid/lymphoid or mixed-lineage leukemia; translocated to, 10 [Source:MGI Symbol;Acc:MGI:1329038]	Mllt10	protein_coding
ENSMUSG00000026775	2186.140594	0.319487167	0.120900119	0.001631535	0.028229163	Up	27377	YME1-like 1 (S. cerevisiae) [Source:MGI Symbol;Acc:MGI:1351651]	Yme1l1	protein_coding
ENSMUSG00000026781	1729.080643	0.35474675	0.114999572	0.000316209	0.010964242	Up	74159	acyl-Coenzyme A binding domain containing 5 [Source:MGI Symbol;Acc:MGI:1921409]	Acbd5	protein_coding
ENSMUSG00000026807	30.71595025	-1.34181838	0.489220026	0.000223533	0.009224534	Down	68870	adenylate kinase 8 [Source:MGI Symbol;Acc:MGI:1916120]	Ak8	protein_coding
ENSMUSG00000026809	9.105123928	-2.175576804	0.755207183	0.000148642	0.00753397	Down	69987	sperm acrosome associated 9 [Source:MGI Symbol;Acc:MGI:1917237]	Spaca9	protein_coding
ENSMUSG00000026811	516.5179527	-0.352273506	0.156383521	0.003112337	0.039495505	Down	50935	ST6 (alpha-N-acetyl-neuraminyl-2,3-beta-galactosyl-1,3)-N-acetylgalactosaminide alpha-2,6-sialyltransferase 6 [Source:MGI Symbol;Acc:MGI:1355316]	St6galnac6	protein_coding
ENSMUSG00000026817	269.2223608	-0.708125908	0.244886173	0.000209467	0.008965847	Down	11636	adenylate kinase 1 [Source:MGI Symbol;Acc:MGI:87977]	Ak1	protein_coding
ENSMUSG00000026822	386.5789905	-0.118035802	0.233421404	0.00274769	0.037079831	Down	16819	lipocalin 2 [Source:MGI Symbol;Acc:MGI:96757]	Lcn2	protein_coding
ENSMUSG00000026827	1768.490941	0.92428771	0.318302741	0.000157531	0.007675767	Up	14571	glycerol phosphate dehydrogenase 2, mitochondrial [Source:MGI Symbol;Acc:MGI:99778]	Gpd2	protein_coding
ENSMUSG00000026870	11.75667946	-0.069998801	0.189749797	0.002039284	0.031917456	Down	77996	cutA divalent cation tolerance homolog-like [Source:MGI Symbol;Acc:MGI:1925246]	Cutal	protein_coding
ENSMUSG00000026878	2950.478	0.227310219	0.065040313	0.000162023	0.007749885	Up	68365	RAB14, member RAS oncogene family [Source:MGI Symbol;Acc:MGI:1915615]	Rab14	protein_coding
ENSMUSG00000026894	5.410721012	-0.081226735	0.197387586	0.002385578	0.034692551	Down	75495	MORN repeat containing 5 [Source:MGI Symbol;Acc:MGI:1922745]	Morn5	protein_coding
ENSMUSG00000026896	659.2739521	0.312850037	0.117395055	0.00143054	0.026170378	Up	71586	interferon induced with helicase C domain 1 [Source:MGI Symbol;Acc:MGI:1918836]	Ifih1	protein_coding
ENSMUSG00000026926	1287.953563	0.243735746	0.088160727	0.001682611	0.028711005	Up	66865	peptidase (mitochondrial processing) alpha [Source:MGI Symbol;Acc:MGI:1918568]	Pmpca	protein_coding
ENSMUSG00000026928	77.86573381	-0.596402666	0.277856197	0.00170839	0.028849913	Down	332579	caspase recruitment domain family, member 9 [Source:MGI Symbol;Acc:MGI:2685628]	Card9	protein_coding
ENSMUSG00000027002	4540.108693	0.260783068	0.100474049	0.002132434	0.032697518	Up	50884	NCK-associated protein 1 [Source:MGI Symbol;Acc:MGI:1355333]	Nckap1	protein_coding
ENSMUSG00000027070	554.8176024	-1.323068265	0.689007155	0.001360224	0.025674417	Down	14725	low density lipoprotein receptor-related protein 2 [Source:MGI Symbol;Acc:MGI:95794]	Lrp2	protein_coding
ENSMUSG00000027075	833.4676045	0.658619664	0.440869943	0.004623545	0.04955072	Up	72401	solute carrier family 43, member 1 [Source:MGI Symbol;Acc:MGI:1931352]	Slc43a1	protein_coding
ENSMUSG00000027086	264.4060353	0.353358886	0.14191125	0.001785763	0.029521437	Up	320720	FAST kinase domains 1 [Source:MGI Symbol;Acc:MGI:2444596]	Fastkd1	protein_coding
ENSMUSG00000027104	1138.194852	0.316573399	0.121859131	0.001626358	0.028224608	Up	11909	activating transcription factor 2 [Source:MGI Symbol;Acc:MGI:109349]	Atf2	protein_coding
ENSMUSG00000027133	516.4712761	-0.46022074	0.22347213	0.002839137	0.037752196	Down	66181	NOP10 ribonucleoprotein [Source:MGI Symbol;Acc:MGI:1913431]	Nop10	protein_coding
ENSMUSG00000027160	178.9115906	-0.415129225	0.179245069	0.002083361	0.032373815	Down	68201	coiled-coil domain containing 34 [Source:MGI Symbol;Acc:MGI:1915451]	Ccdc34	protein_coding
ENSMUSG00000027164	530.6513201	0.333676424	0.145537176	0.003276113	0.041089706	Up	22034	TNF receptor-associated factor 6 [Source:MGI Symbol;Acc:MGI:108072]	Traf6	protein_coding
ENSMUSG00000027173	22.29290828	-1.076266371	0.385632214	0.000224114	0.009224534	Down	211896	DEP domain containing 7 [Source:MGI Symbol;Acc:MGI:2139258]	Depdc7	protein_coding
ENSMUSG00000027180	1586.645994	0.258213629	0.087986421	0.000946722	0.021358851	Up	57443	F-box protein 3 [Source:MGI Symbol;Acc:MGI:1929084]	Fbxo3	protein_coding
ENSMUSG00000027184	6130.520153	0.273388893	0.06548702	8.20E-06	0.001563424	Up	53872	cell cycle associated protein 1 [Source:MGI Symbol;Acc:MGI:1858234]	Caprin1	protein_coding
ENSMUSG00000027193	1560.078926	0.272444359	0.093226452	0.000849303	0.019994595	Up	11800	apoptosis inhibitor 5 [Source:MGI Symbol;Acc:MGI:1888993]	Api5	protein_coding
ENSMUSG00000027195	4368.252265	0.431773225	0.170020462	0.001209872	0.024318448	Up	56348	hydroxysteroid (17-beta) dehydrogenase 12 [Source:MGI Symbol;Acc:MGI:1926967]	Hsd17b12	protein_coding
ENSMUSG00000027215	712.5169708	-0.555145868	0.183031449	0.000220016	0.009207591	Down	12521	CD82 antigen [Source:MGI Symbol;Acc:MGI:104651]	Cd82	protein_coding
ENSMUSG00000027221	4121.999028	0.449050262	0.231146156	0.003955946	0.045753568	Up	76969	carbohydrate sulfotransferase 1 [Source:MGI Symbol;Acc:MGI:1924219]	Chst1	protein_coding
ENSMUSG00000027254	930.8538567	-1.211637882	0.480912107	0.000404748	0.013131231	Down	17754	microtubule-associated protein 1 A [Source:MGI Symbol;Acc:MGI:1306776]	Map1a	protein_coding
ENSMUSG00000027257	472.0146167	-0.48509596	0.270317105	0.00442185	0.048228949	Down	80708	protein kinase C and casein kinase substrate in neurons 3 [Source:MGI Symbol;Acc:MGI:1891410]	Pacsin3	protein_coding
ENSMUSG00000027276	1107.581417	0.4000254	0.13986287	0.000525811	0.015280444	Up	16449	jagged 1 [Source:MGI Symbol;Acc:MGI:1095416]	Jag1	protein_coding
ENSMUSG00000027333	177.939286	-0.395830967	0.192499856	0.003974431	0.045835427	Down	228608	spermine oxidase [Source:MGI Symbol;Acc:MGI:2445356]	Smox	protein_coding
ENSMUSG00000027346	968.2825488	0.329142828	0.116219816	0.00080239	0.019540309	Up	74182	glycerophosphocholine phosphodiesterase 1 [Source:MGI Symbol;Acc:MGI:104898]	Gpcpd1	protein_coding
ENSMUSG00000027365	1832.094371	0.344444379	0.141045252	0.002204994	0.03336142	Up	58800	transient receptor potential cation channel, subfamily M, member 7 [Source:MGI Symbol;Acc:MGI:1929996]	Trpm7	protein_coding
ENSMUSG00000027367	1579.544711	0.236583592	0.097013548	0.004440315	0.048399716	Up	99138	START domain containing 7 [Source:MGI Symbol;Acc:MGI:2139090]	Stard7	protein_coding
ENSMUSG00000027420	42.68691257	-0.758387415	0.338031396	0.001075302	0.022821025	Down	12075	beaded filament structural protein 1, in lens-CP94 [Source:MGI Symbol;Acc:MGI:101770]	Bfsp1	protein_coding
ENSMUSG00000027422	5934.216671	-0.393231062	0.164192582	0.001844536	0.030090232	Down	81910	ribosome binding protein 1 [Source:MGI Symbol;Acc:MGI:1932395]	Rrbp1	protein_coding
ENSMUSG00000027439	1289.457448	0.386770132	0.12555248	0.000269337	0.010102419	Up	74533	GDNF-inducible zinc finger protein 1 [Source:MGI Symbol;Acc:MGI:1921783]	Gzf1	protein_coding
ENSMUSG00000027455	1155.304762	-0.398288218	0.198646286	0.004196026	0.04696359	Down	386649	NSFL1 (p97) cofactor (p47) [Source:MGI Symbol;Acc:MGI:3042273]	Nsfl1c	protein_coding
ENSMUSG00000027459	94.11558044	-0.726397152	0.259360109	0.000273537	0.010102419	Down	73847	family with sequence similarity 110, member A [Source:MGI Symbol;Acc:MGI:1921097]	Fam110a	protein_coding
ENSMUSG00000027463	79.22356281	-0.959054191	0.405550341	0.000658892	0.017631495	Down	69698	solute carrier protein family 52, member 3 [Source:MGI Symbol;Acc:MGI:1916948]	Slc52a3	protein_coding
ENSMUSG00000027488	2242.812844	-0.45696519	0.240346924	0.004256464	0.047293134	Down	20648	syntrophin, acidic 1 [Source:MGI Symbol;Acc:MGI:101772]	Snta1	protein_coding
ENSMUSG00000027490	233.0535071	-0.550845414	0.236676018	0.001268083	0.024669378	Down	13555	E2F transcription factor 1 [Source:MGI Symbol;Acc:MGI:101941]	E2f1	protein_coding

ENSMUSG00000027556	44.49767815	0.582579352	0.308186172	0.002992074	0.038681479	Up	12346	carbonic anhydrase 1 [Source:MGI Symbol;Acc:MGI:88268]	Car1	protein_coding
ENSMUSG00000027583	361.1162606	0.389658888	0.168637729	0.002323055	0.034099835	Up	72147	zinc finger and BTB domain containing 46 [Source:MGI Symbol;Acc:MGI:1919397]	Zbtb46	protein_coding
ENSMUSG00000027630	767.7883102	0.306998351	0.113531769	0.001322895	0.025274335	Up	81004	transducin (beta)-like 1X-linked receptor 1 [Source:MGI Symbol;Acc:MGI:2441730]	Tbl1xr1	protein_coding
ENSMUSG00000027634	1086.519338	0.286311512	0.11611755	0.002914166	0.038277303	Up	29812	N-myc downstream regulated gene 3 [Source:MGI Symbol;Acc:MGI:1352499]	Ndrg3	protein_coding
ENSMUSG00000027641	309.6498292	0.335983879	0.119256431	0.000765184	0.019013207	Up	19650	RB transcriptional corepressor like 1 [Source:MGI Symbol;Acc:MGI:103300]	Rbl1	protein_coding
ENSMUSG00000027646	417.7631939	-0.405674576	0.175248746	0.002122603	0.032652559	Down	20779	Rous sarcoma oncogene [Source:MGI Symbol;Acc:MGI:98397]	Src	protein_coding
ENSMUSG00000027684	178.6773009	0.397494165	0.197295713	0.004245209	0.047259485	Up	14013	MDS1 and EVI1 complex locus [Source:MGI Symbol;Acc:MGI:95457]	Mecom	protein_coding
ENSMUSG00000027709	3053.657622	0.308622786	0.131268356	0.003812766	0.045022918	Up	72039	methylcrotonoyl-Coenzyme A carboxylase 1 (alpha) [Source:MGI Symbol;Acc:MGI:1919289]	Mccc1	protein_coding
ENSMUSG00000027763	5184.449986	0.317917217	0.101055793	0.000289174	0.010469191	Up	56758	muscleblind like splicing factor 1 [Source:MGI Symbol;Acc:MGI:1928482]	Mbnl1	protein_coding
ENSMUSG00000027770	881.7962386	0.326282739	0.075978381	3.40E-06	0.000946988	Up	72162	DEAH (Asp-Glu-Ala-His) box polypeptide 36 [Source:MGI Symbol;Acc:MGI:1919412]	Dhx36	protein_coding
ENSMUSG00000027792	4440.334886	0.509800588	0.151483583	7.26E-05	0.005101093	Up	12038	butyrylcholinesterase [Source:MGI Symbol;Acc:MGI:894278]	Bche	protein_coding
ENSMUSG00000027809	2077.360948	0.299445521	0.090221453	0.000210055	0.008965847	Up	66841	electron transferring flavoprotein, dehydrogenase [Source:MGI Symbol;Acc:MGI:106100]	Etfdh	protein_coding
ENSMUSG00000027829	1278.49538	0.455968608	0.171630684	0.000761456	0.018990119	Up	56706	cyclin L1 [Source:MGI Symbol;Acc:MGI:1922664]	Ccnl1	protein_coding
ENSMUSG00000027848	439.5930901	-1.075296033	0.393603221	0.000241876	0.009560194	Down	99543	olfactomedin-like 3 [Source:MGI Symbol;Acc:MGI:1914877]	Olfml3	protein_coding
ENSMUSG00000027932	154.3212173	-0.562548013	0.168491574	6.56E-05	0.004891251	Down	26568	solute carrier family 27 (fatty acid transporter), member 3 [Source:MGI Symbol;Acc:MGI:1347358]	Slc27a3	protein_coding
ENSMUSG00000028024	4833.367722	0.573924218	0.301373694	0.002924014	0.038296458	Up	13809	glutamyl aminopeptidase [Source:MGI Symbol;Acc:MGI:106645]	Enpep	protein_coding
ENSMUSG00000028030	448.4760051	0.323728212	0.13347158	0.002529058	0.035665506	Up	271981	TBC1 domain containing kinase [Source:MGI Symbol;Acc:MGI:2445052]	Tbck	protein_coding
ENSMUSG00000028041	4096.481082	-0.267491558	0.097574058	0.001550254	0.027385104	Down	11490	a disintegrin and metallopeptidase domain 15 (metargidin) [Source:MGI Symbol;Acc:MGI:1333882]	Adam15	protein_coding
ENSMUSG00000028060	1104.678839	0.297824595	0.103392273	0.000822502	0.019686367	Up	74200	KH domain containing 4, pre-mRNA splicing factor [Source:MGI Symbol;Acc:MGI:1921450]	Khdc4	protein_coding
ENSMUSG00000028062	836.8403515	-0.680308816	0.289469061	0.00093809	0.021271189	Down	83409	late endosomal/lysosomal adaptor, MAPK and MTOR activator 2 [Source:MGI Symbol;Acc:MGI:1932697]	Lamtor2	protein_coding
ENSMUSG00000028063	2948.203774	-0.331641102	0.133209926	0.001854264	0.030173051	Down	16905	lamin A [Source:MGI Symbol;Acc:MGI:96794]	Lmna	protein_coding
ENSMUSG00000028070	717.2052093	-0.382367995	0.159266677	0.001914576	0.030663472	Down	246703	NAD(P)HX epimerase [Source:MGI Symbol;Acc:MGI:2180167]	Naxe	protein_coding
ENSMUSG00000028106	702.2465097	0.327457339	0.108491951	0.00048182	0.01446551	Up	75137	regulation of nuclear pre-mRNA domain containing 2 [Source:MGI Symbol;Acc:MGI:1922387]	Rprd2	protein_coding
ENSMUSG00000028127	2233.849184	0.348433152	0.073748622	3.72E-07	0.000267179	Up	19299	ATP-binding cassette, sub-family D (ALD), member 3 [Source:MGI Symbol;Acc:MGI:1349216]	Abcd3	protein_coding
ENSMUSG00000028136	1084.912512	0.248379599	0.083045141	0.00081263	0.019613515	Up	76742	sorting nexin family member 27 [Source:MGI Symbol;Acc:MGI:1923992]	Snx27	protein_coding
ENSMUSG00000028164	621.6869361	-0.422881502	0.148301414	0.000461003	0.014186548	Down	110173	mannosidase, beta A, lysosomal [Source:MGI Symbol;Acc:MGI:88175]	Manba	protein_coding
ENSMUSG00000028194	165.3593633	-1.124397684	0.428406743	0.000309787	0.010806814	Down	69219	dimethylarginine dimethylaminohydrolase 1 [Source:MGI Symbol;Acc:MGI:1916469]	Ddah1	protein_coding
ENSMUSG00000028224	552.4314051	0.294644906	0.118788705	0.002620133	0.036337993	Up	27354	nibrin [Source:MGI Symbol;Acc:MGI:1351625]	Nbn	protein_coding
ENSMUSG00000028228	1734.406125	0.445407563	0.178214457	0.001113239	0.023364734	Up	70568	copine III [Source:MGI Symbol;Acc:MGI:1917818]	Cpne3	protein_coding
ENSMUSG00000028233	488.7057174	0.336598361	0.130304585	0.001529951	0.027181071	Up	116940	trimethylguanosine synthase 1 [Source:MGI Symbol;Acc:MGI:2151797]	Tgs1	protein_coding
ENSMUSG00000028238	83.13329801	1.111038147	0.880340341	0.003740875	0.044598452	Up	242341	ATPase, H+ transporting, lysosomal V0 subunit D2 [Source:MGI Symbol;Acc:MGI:1924415]	Atp6v0d2	protein_coding
ENSMUSG00000028277	1697.602754	0.223112982	0.085293376	0.003064962	0.039066933	Up	56228	ubiquitin-conjugating enzyme E2J 1 [Source:MGI Symbol;Acc:MGI:1926245]	Ube2j1	protein_coding
ENSMUSG00000028278	23.82919657	-0.135571393	0.257789096	0.00253437	0.035682028	Down	52187	Ras-related GTP binding D [Source:MGI Symbol;Acc:MGI:1098604]	Rragd	protein_coding
ENSMUSG00000028287	5.98426055	-0.071133784	0.19118133	0.000193429	0.008460315	Down	75471	RIKEN cDNA 1700009N14 gene [Source:MGI Symbol;Acc:MGI:1922721]	1700009N14Rik	protein_coding
ENSMUSG00000028334	432.765476	-0.630095659	0.191079443	6.47E-05	0.004869134	Down	94181	N-acetylneuraminic acid synthase (sialic acid synthase) [Source:MGI Symbol;Acc:MGI:2149820]	Nans	protein_coding
ENSMUSG00000028345	337.3766086	0.359314821	0.107146178	0.000123914	0.006874031	Up	269536	testis expressed gene 10 [Source:MGI Symbol;Acc:MGI:1344413]	Tex10	protein_coding
ENSMUSG00000028378	206.2365083	-0.526939077	0.289402587	0.003808107	0.045010358	Down	67103	prostaglandin reductase 1 [Source:MGI Symbol;Acc:MGI:1914353]	Ptgr1	protein_coding
ENSMUSG00000028382	2205.233742	0.360053988	0.12552143	0.000687113	0.017968169	Up	230257	polypyrimidine tract binding protein 3 [Source:MGI Symbol;Acc:MGI:1923334]	Ptbp3	protein_coding
ENSMUSG00000028391	19.08285903	-1.404458086	0.745241539	0.001452084	0.026396378	Down	71354	WD repeat domain 31 [Source:MGI Symbol;Acc:MGI:1918604]	Wdr31	protein_coding
ENSMUSG00000028394	785.5111773	-0.368186456	0.128461466	0.000569346	0.016114087	Down	59001	polymerase (DNA directed), epsilon 3 (p17 subunit) [Source:MGI Symbol;Acc:MGI:1933378]	Pole3	protein_coding
ENSMUSG00000028405	5715.657671	0.395464159	0.153015435	0.001246046	0.024468519	Up	11428	aconitase 1 [Source:MGI Symbol;Acc:MGI:87879]	Aco1	protein_coding
ENSMUSG00000028445	110.0614576	-0.906911829	0.310031819	0.000159889	0.007718096	Down	69638	energy homeostasis associated [Source:MGI Symbol;Acc:MGI:1916888]	Enho	protein_coding
ENSMUSG00000028496	371.0008546	0.425937931	0.15002902	0.000473101	0.014353791	Up	70122	myeloid/lymphoid or mixed-lineage leukemia; translocated to, 3 [Source:MGI Symbol;Acc:MGI:1917372]	Mllt3	protein_coding
ENSMUSG00000028541	219.8542939	-0.665835941	0.289008756	0.001047748	0.022569798	Down	53418	UDP-Gal:betaGlcNAc beta 1,4- galactosyltransferase, polypeptide 2 [Source:MGI Symbol;Acc:MGI:1858493]	B4galt2	protein_coding
ENSMUSG00000028555	26.26972386	-0.137405316	0.261101755	0.002233285	0.033651183	Down	230603	tetratricopeptide repeat domain 39A [Source:MGI Symbol;Acc:MGI:2444350]	Ttc39a	protein_coding
ENSMUSG00000028558	15.04887691	-1.394933925	0.719726998	0.001488031	0.026877103	Down	108802	calreticulin 4 [Source:MGI Symbol;Acc:MGI:2140435]	Calr4	protein_coding
ENSMUSG00000028582	698.6766948	-0.23817903	0.089171196	0.002216442	0.033505218	Down	319965	coiled-coil and C2 domain containing 1B [Source:MGI Symbol;Acc:MGI:2443076]	Cc2d1b	protein_coding
ENSMUSG00000028607	2760.916678	0.419923612	0.170324317	0.001392216	0.02591718	Up	12896	carnitine palmitoyltransferase 2 [Source:MGI Symbol;Acc:MGI:109176]	Cpt2	protein_coding
ENSMUSG00000028614	580.7174748	0.305332286	0.128059556	0.003155909	0.039989548	Up	72787	NDC1 transmembrane nucleoporin [Source:MGI Symbol;Acc:MGI:1920037]	Ndc1	protein_coding
ENSMUSG00000028637	35.2743041	-1.607251621	0.703723427	0.000618306	0.016966997	Down	73332	coiled-coil domain containing 30 [Source:MGI Symbol;Acc:MGI:1920582]	Ccdc30	protein_coding
ENSMUSG00000028643	247.4508882	-0.517582755	0.169930205	0.000178121	0.008185484	Down	69216	small vasohibin binding protein [Source:MGI Symbol;Acc:MGI:1916466]	Svbp	protein_coding
ENSMUSG00000028673	2349.683269	-0.371553563	0.112586679	0.000137625	0.007275122	Down	71665	fucosidase, alpha-L- 1, tissue [Source:MGI Symbol;Acc:MGI:95593]	Fuca1	protein_coding
ENSMUSG00000028688	132.5881705	-0.319602422	0.14473517	0.004360311	0.047982906	Down	68276	target of EGR1, member 1 (nuclear) [Source:MGI Symbol;Acc:MGI:1915526]	Toe1	protein_coding

ENSMUSG00000028756	4355.50124	-0.411434887	0.156660895	0.000914043	0.02094642	Down	68943	PTEN induced putative kinase 1 [Source:MGI Symbol;Acc:MGI:1916193]	Pink1	protein_coding
ENSMUSG00000028758	22.09542118	-1.671283722	0.525668702	5.51E-05	0.004447279	Down	16559	kinesin family member 17 [Source:MGI Symbol;Acc:MGI:1098229]	Kif17	protein_coding
ENSMUSG00000028759	2922.176724	0.178187837	0.066410658	0.003541092	0.043004611	Up	15441	heterochromatin protein 1, binding protein 3 [Source:MGI Symbol;Acc:MGI:109369]	Hp1bp3	protein_coding
ENSMUSG00000028789	453.4515187	-0.474954198	0.248147751	0.003738656	0.044598452	Down	242669	antizyme inhibitor 2 [Source:MGI Symbol;Acc:MGI:2442093]	Azin2	protein_coding
ENSMUSG00000028832	164.1374775	-0.816536638	0.386075969	0.001304217	0.025189625	Down	16765	stathmin 1 [Source:MGI Symbol;Acc:MGI:96739]	Stmn1	protein_coding
ENSMUSG00000028836	65.60981462	-0.096234044	0.210168047	0.002283067	0.033800769	Down	230810	solute carrier family 30 (zinc transporter), member 2 [Source:MGI Symbol;Acc:MGI:106637]	Slc30a2	protein_coding
ENSMUSG00000028838	28.89850448	-1.356017936	0.533683085	0.000365005	0.012142269	Down	56219	exostosin-like glycosyltransferase 1 [Source:MGI Symbol;Acc:MGI:1888742]	Extl1	protein_coding
ENSMUSG00000028851	1163.631111	-0.544434803	0.185523879	0.000262748	0.009995247	Down	18221	nudC nuclear distribution protein [Source:MGI Symbol;Acc:MGI:106014]	Nudc	protein_coding
ENSMUSG00000028919	40.90351067	-0.248689458	0.737634014	0.002974869	0.038516848	Down	213649	Rho guanine nucleotide exchange factor (GEF) 19 [Source:MGI Symbol;Acc:MGI:1925912]	Arhgef19	protein_coding
ENSMUSG00000028937	549.7033842	-0.305538531	0.126448448	0.002670613	0.036638703	Down	70025	acyl-CoA thioesterase 7 [Source:MGI Symbol;Acc:MGI:1917275]	Acot7	protein_coding
ENSMUSG00000028943	216.0313134	-1.344898037	0.459641151	0.000121132	0.0068438	Down	56226	espin [Source:MGI Symbol;Acc:MGI:1861630]	Espn	protein_coding
ENSMUSG00000028998	384.950259	-0.912414311	0.295251674	9.18E-05	0.005821151	Down	66169	translocase of outer mitochondrial membrane 7 [Source:MGI Symbol;Acc:MGI:1913419]	Tomm7	protein_coding
ENSMUSG00000029007	553.3524099	-0.518628725	0.154559488	6.67E-05	0.004952194	Down	11610	angiotensin II, type I receptor-associated protein [Source:MGI Symbol;Acc:MGI:1339977]	Agtrap	protein_coding
ENSMUSG00000029032	28.42367824	-0.114448632	0.229066709	0.002726433	0.036914178	Down	230972	Rho guanine nucleotide exchange factor (GEF) 16 [Source:MGI Symbol;Acc:MGI:2446219]	Arhgef16	protein_coding
ENSMUSG00000029049	60.43439449	-0.79746299	0.371951163	0.00126689	0.024669378	Down	76866	MORN repeat containing 1 [Source:MGI Symbol;Acc:MGI:1924116]	Morn1	protein_coding
ENSMUSG00000029068	3989.251622	0.391287966	0.114812773	7.41E-05	0.005101815	Up	56036	cyclin L2 [Source:MGI Symbol;Acc:MGI:1927119]	Ccnl2	protein_coding
ENSMUSG00000029070	3011.977429	-0.373508295	0.18167554	0.004401498	0.048144948	Down	74761	matrix-remodelling associated 8 [Source:MGI Symbol;Acc:MGI:1922011]	Mxra8	protein_coding
ENSMUSG00000029094	2146.0946	0.381768282	0.172805435	0.003052641	0.038967523	Up	70292	actin filament associated protein 1 [Source:MGI Symbol;Acc:MGI:1917542]	Afap1	protein_coding
ENSMUSG00000029110	2490.678589	0.348703835	0.074803966	5.62E-07	0.000372469	Up	19822	ring finger protein 4 [Source:MGI Symbol;Acc:MGI:1201691]	Rnf4	protein_coding
ENSMUSG00000029126	197.0380245	-0.792012817	0.248435151	7.79E-05	0.005267261	Down	18196	neuron specific gene family member 1 [Source:MGI Symbol;Acc:MGI:109149]	Nsg1	protein_coding
ENSMUSG00000029153	61.4794496	-1.005624266	0.371745556	0.000273296	0.010102419	Down	433904	OCIA domain containing 2 [Source:MGI Symbol;Acc:MGI:1916377]	Ociad2	protein_coding
ENSMUSG00000029169	1909.676722	0.257432675	0.093927741	0.001631174	0.028229163	Up	13204	DEAH (Asp-Glu-Ala-His) box polypeptide 15 [Source:MGI Symbol;Acc:MGI:1099786]	Dhx15	protein_coding
ENSMUSG00000029178	1602.411149	0.315689791	0.125057865	0.002114837	0.032569253	Up	16599	Kruppel-like factor 3 (basic) [Source:MGI Symbol;Acc:MGI:1342773]	Klf3	protein_coding
ENSMUSG00000029190	2889.959179	0.259972558	0.101630144	0.002728989	0.036914178	Up	320661	DNA segment, Chr 5, ERATO Doi 579, expressed [Source:MGI Symbol;Acc:MGI:1261849]	D5Ertd579e	protein_coding
ENSMUSG00000029195	1408.33851	0.38526299	0.184007021	0.004016385	0.045941738	Up	83379	klotho beta [Source:MGI Symbol;Acc:MGI:1932466]	Klb	protein_coding
ENSMUSG00000029202	1821.368443	0.319403296	0.1012373	0.000305058	0.010757394	Up	71521	PDS5 cohesin associated factor A [Source:MGI Symbol;Acc:MGI:1918771]	Pds5a	protein_coding
ENSMUSG00000029217	185.5113966	-0.581380076	0.343058778	0.004231215	0.047149397	Down	21682	tec protein tyrosine kinase [Source:MGI Symbol;Acc:MGI:98662]	Tec	protein_coding
ENSMUSG00000029287	4222.264097	0.373996113	0.151689228	0.001847355	0.030090232	Up	21814	transforming growth factor, beta receptor III [Source:MGI Symbol;Acc:MGI:104637]	Tgfbr3	protein_coding
ENSMUSG00000029304	74.55620777	-0.122107048	0.242490072	0.000228762	0.009224534	Down	20750	secreted phosphoprotein 1 [Source:MGI Symbol;Acc:MGI:98389]	Spp1	protein_coding
ENSMUSG00000029310	820.1220493	-0.379301236	0.186943293	0.004201473	0.046985069	Down	74167	nudix (nucleoside diphosphate linked moiety X)-type motif 9 [Source:MGI Symbol;Acc:MGI:1921417]	Nudt9	protein_coding
ENSMUSG00000029314	1825.406334	0.400414412	0.199226086	0.004643046	0.049605466	Up	231510	glycerol-3-phosphate acyltransferase 3 [Source:MGI Symbol;Acc:MGI:3603816]	Gpat3	protein_coding
ENSMUSG00000029330	431.2228276	-0.476836528	0.258116519	0.00427929	0.0474291	Down	74596	CDP-diacylglycerol synthase 1 [Source:MGI Symbol;Acc:MGI:1921846]	Cds1	protein_coding
ENSMUSG00000029344	1039.595346	-0.41076107	0.122054309	8.59E-05	0.005567852	Down	22022	protein-tyrosine sulfotransferase 2 [Source:MGI Symbol;Acc:MGI:1309516]	Tpst2	protein_coding
ENSMUSG00000029359	36.58528658	-1.488146444	0.479267649	7.02E-05	0.00504748	Down	57816	tescalcin [Source:MGI Symbol;Acc:MGI:1930803]	Tesc	protein_coding
ENSMUSG00000029368	1167.779297	1.477604935	1.060371564	0.002924513	0.038296458	Up	11657	albumin [Source:MGI Symbol;Acc:MGI:87991]	Alb	protein_coding
ENSMUSG00000029370	32.00337392	1.158299658	0.732316543	0.00260492	0.036231825	Up	73246	Ras association (RalGDS/AF-6) domain family member 6 [Source:MGI Symbol;Acc:MGI:1920496]	Rassf6	protein_coding
ENSMUSG00000029392	201.6567041	-0.445388263	0.167332026	0.000741244	0.018765676	Down	75695	Rab interacting lysosomal protein-like 1 [Source:MGI Symbol;Acc:MGI:1922945]	Rilpl1	protein_coding
ENSMUSG00000029438	321.9876532	-0.568237766	0.275279852	0.002143514	0.032776559	Down	77045	B cell CLL/lymphoma 7A [Source:MGI Symbol;Acc:MGI:1924295]	Bcl7a	protein_coding
ENSMUSG00000029442	109.1357497	-0.902295191	0.439575357	0.001345811	0.025570409	Down	269701	WD repeat domain 66 [Source:MGI Symbol;Acc:MGI:1918495]	Wdr66	protein_coding
ENSMUSG00000029484	1950.333396	-0.567898742	0.168548089	5.73E-05	0.004529643	Down	11745	annexin A3 [Source:MGI Symbol;Acc:MGI:1201378]	Anxa3	protein_coding
ENSMUSG00000029501	886.7601487	0.241064683	0.067850824	0.000122653	0.006862581	Up	71782	ankyrin repeat and LEM domain containing 2 [Source:MGI Symbol;Acc:MGI:1261856]	Ankle2	protein_coding
ENSMUSG00000029528	1370.197056	-0.345683404	0.135595631	0.001860713	0.030193654	Down	19303	paxillin [Source:MGI Symbol;Acc:MGI:108295]	Pxn	protein_coding
ENSMUSG00000029594	238.5646111	-0.280753654	0.116750948	0.003346737	0.041702333	Down	74111	RNA binding motif protein 19 [Source:MGI Symbol;Acc:MGI:1921361]	Rbm19	protein_coding
ENSMUSG00000029596	271.9648532	-0.556139484	0.330130911	0.004133694	0.046825357	Down	257635	serine dehydratase-like [Source:MGI Symbol;Acc:MGI:2182607]	Sdsl	protein_coding
ENSMUSG00000029616	1235.293381	-0.437404888	0.184548831	0.001660449	0.028528929	Down	67397	endoplasmic reticulum protein 29 [Source:MGI Symbol;Acc:MGI:1914647]	Erp29	protein_coding
ENSMUSG00000029622	3877.361944	-0.356066451	0.161798145	0.003463589	0.042634311	Down	11867	actin related protein 2/3 complex, subunit 1B [Source:MGI Symbol;Acc:MGI:1343142]	Arpc1b	protein_coding
ENSMUSG00000029634	928.613522	-0.23146755	0.088212584	0.002771651	0.03719373	Down	74132	ring finger protein (C3H2C3 type) 6 [Source:MGI Symbol;Acc:MGI:1921382]	Rnf6	protein_coding
ENSMUSG00000029647	739.4580317	0.275928191	0.098421711	0.001202527	0.024267616	Up	72587	PAN3 poly(A) specific ribonuclease subunit [Source:MGI Symbol;Acc:MGI:1919837]	Pan3	protein_coding
ENSMUSG00000029658	40.8321986	-0.10284582	0.216170878	0.003130128	0.039692044	Down	381693	WD40 repeat domain 95 [Source:MGI Symbol;Acc:MGI:1923042]	Wdr95	protein_coding
ENSMUSG00000029669	2859.136349	0.362041278	0.138371722	0.001242152	0.024468519	Up	269831	tetraspanin 12 [Source:MGI Symbol;Acc:MGI:1889818]	Tspan12	protein_coding
ENSMUSG00000029670	308.535391	0.459615837	0.181858776	0.001036663	0.022443242	Up	71777	inhibitor of growth family, member 3 [Source:MGI Symbol;Acc:MGI:1919027]	Ing3	protein_coding
ENSMUSG00000029675	2028.269355	-0.536028158	0.295903394	0.003773931	0.044767499	Down	13717	elastin [Source:MGI Symbol;Acc:MGI:95317]	Eln	protein_coding
ENSMUSG00000029684	1281.252569	0.307565634	0.081762964	3.69E-05	0.003584747	Up	73178	WASP like actin nucleation promoting factor [Source:MGI Symbol;Acc:MGI:1920428]	Wasl	protein_coding
ENSMUSG00000029686	1937.835932	0.327784262	0.095885677	0.000117023	0.006722209	Up	26965	cullin 1 [Source:MGI Symbol;Acc:MGI:1349658]	Cul1	protein_coding
ENSMUSG00000029695	61.89430903	-1.318811616	0.394659159	3.42E-05	0.003431462	Down	30956	aminoadipate-semialdehyde synthase [Source:MGI Symbol;Acc:MGI:1353573]	Aass	protein_coding

ENSMUSG00000029712	9.430334931	-1.236723461	0.614705696	0.001466408	0.026572664	Down	83766	actin-like 6B [Source:MGI Symbol;Acc:MGI:1933548]	Actl6b	protein_coding
ENSMUSG00000029716	251.2824176	-1.564188304	0.621980592	0.00035452	0.011906857	Down	50765	transferrin receptor 2 [Source:MGI Symbol;Acc:MGI:1354956]	Tfr2	protein_coding
ENSMUSG00000029723	1014.834758	-0.29176959	0.12794437	0.00434606	0.047917882	Down	78829	sperm acrosome developmental regulator [Source:MGI Symbol;Acc:MGI:1926079]	Spacdr	protein_coding
ENSMUSG00000029725	212.60121	-0.501026735	0.236251404	0.002342642	0.034328875	Down	69871	protein phosphatase 1, regulatory subunit 35 [Source:MGI Symbol;Acc:MGI:1922853]	Ppp1r35	protein_coding
ENSMUSG00000029729	419.2490955	0.442677649	0.10590477	3.40E-06	0.000946988	Up	74570	zinc finger with KRAB and SCAN domains 1 [Source:MGI Symbol;Acc:MGI:1921820]	Zkscan1	protein_coding
ENSMUSG00000029759	2526.65068	0.279238477	0.10736374	0.00226185	0.033800769	Up	269823	paraoxonase 3 [Source:MGI Symbol;Acc:MGI:106686]	Pon3	protein_coding
ENSMUSG00000029769	26.0780477	-2.503701626	0.707435061	1.40E-05	0.002135875	Down	232664	coiled-coil domain containing 136 [Source:MGI Symbol;Acc:MGI:1918128]	Ccdc136	protein_coding
ENSMUSG00000029777	1732.038958	0.249734478	0.103673955	0.004483932	0.048662455	Up	353172	glycyl-tRNA synthetase [Source:MGI Symbol;Acc:MGI:2449057]	Gars	protein_coding
ENSMUSG00000029823	2966.200939	0.393112481	0.114073984	7.00E-05	0.00504748	Up	192196	LUC7-like 2 (S. cerevisiae) [Source:MGI Symbol;Acc:MGI:2183260]	Luc7l2	protein_coding
ENSMUSG00000029836	724.3000063	0.271854105	0.092505459	0.000857353	0.020074414	Up	12417	chromobox 3 [Source:MGI Symbol;Acc:MGI:108515]	Cbx3	protein_coding
ENSMUSG00000029920	462.1688775	0.425633413	0.152925028	0.000573448	0.016152764	Up	13990	SWI/SNF-related, matrix-associated actin-dependent regulator of chromatin, subfamily a, containing DEAD/H box 1 [Source:MGI Symbol;Acc:MGI:95453]	Smarcad1	protein_coding
ENSMUSG00000029922	841.6066322	-0.374390356	0.145095783	0.001267513	0.024669378	Down	54484	makorin, ring finger protein, 1 [Source:MGI Symbol;Acc:MGI:1859353]	Mkrn1	protein_coding
ENSMUSG00000030002	1792.881523	0.306817475	0.120549867	0.001921137	0.030739972	Up	72102	dual specificity phosphatase 11 (RNA/RNP complex 1-interacting) [Source:MGI Symbol;Acc:MGI:1919352]	Dusp11	protein_coding
ENSMUSG00000030016	1231.327366	0.460889813	0.156089902	0.000329373	0.011284452	Up	18139	zinc finger protein 638 [Source:MGI Symbol;Acc:MGI:1203484]	Zfp638	protein_coding
ENSMUSG00000030030	22.39339903	-1.949874755	0.744599772	0.000259003	0.009940748	Down	71837	RIKEN cDNA 1700003E16 gene [Source:MGI Symbol;Acc:MGI:1919087]	1700003E16Rik	protein_coding
ENSMUSG00000030034	229.4653517	-0.944156334	0.296763562	6.86E-05	0.005010397	Down	70020	INO80 complex subunit B [Source:MGI Symbol;Acc:MGI:1917270]	Ino80b	protein_coding
ENSMUSG00000030079	370.2976443	-0.366382218	0.169999124	0.003431176	0.042478059	Down	56505	RuvB-like protein 1 [Source:MGI Symbol;Acc:MGI:1928760]	Ruvbl1	protein_coding
ENSMUSG00000030089	209.4963146	-0.48896214	0.224135501	0.002132432	0.032697518	Down	71699	solute carrier family 41, member 3 [Source:MGI Symbol;Acc:MGI:1918949]	Slc41a3	protein_coding
ENSMUSG00000030105	2232.70467	0.303681245	0.1226643	0.002565888	0.035978797	Up	67166	ADP-ribosylation factor-like 8B [Source:MGI Symbol;Acc:MGI:1914416]	Arl8b	protein_coding
ENSMUSG00000030125	26.27951367	-0.105359604	0.219560854	0.001710939	0.028849913	Down	16977	leucine rich repeat containing 23 [Source:MGI Symbol;Acc:MGI:1315192]	Lrrc23	protein_coding
ENSMUSG00000030168	12149.38848	0.438021607	0.203400755	0.00300139	0.038743781	Up	68465	adiponectin receptor 2 [Source:MGI Symbol;Acc:MGI:93830]	Adipor2	protein_coding
ENSMUSG00000030170	115.0393043	-0.896685548	0.203555658	6.06E-07	0.000383541	Down	22419	wingless-type MMTV integration site family, member 5B [Source:MGI Symbol;Acc:MGI:98959]	Wnt5b	protein_coding
ENSMUSG00000030218	1157.297681	-0.9481482	0.43699836	0.001026205	0.022329031	Down	17313	matrix Gla protein [Source:MGI Symbol;Acc:MGI:96976]	Mgp	protein_coding
ENSMUSG00000030272	1665.11201	-0.432660439	0.104054485	4.00E-06	0.000999284	Down	52163	calcium/calmodulin-dependent protein kinase I [Source:MGI Symbol;Acc:MGI:1098535]	Camk1	protein_coding
ENSMUSG00000030275	2150.826504	0.257688154	0.106729018	0.004327211	0.047801817	Up	75320	ethanolamine kinase 1 [Source:MGI Symbol;Acc:MGI:1922570]	Etnk1	protein_coding
ENSMUSG00000030276	89.98519963	-1.064195989	0.587700465	0.001876912	0.030289224	Down	101100	tubulin tyrosine ligase-like family, member 3 [Source:MGI Symbol;Acc:MGI:2141418]	Ttll3	protein_coding
ENSMUSG00000030304	560.2530192	0.275548079	0.119092457	0.004555145	0.049112217	Up	67456	ERGIC and golgi 2 [Source:MGI Symbol;Acc:MGI:1914706]	Ergic2	protein_coding
ENSMUSG00000030321	16.38645951	-0.131846822	0.251672416	0.002898365	0.038239246	Down	212516	EF-hand calcium binding domain 12 [Source:MGI Symbol;Acc:MGI:2681834]	Efcab12	protein_coding
ENSMUSG00000030327	577.6906139	0.334877818	0.097988767	0.00011059	0.006438521	Up	67602	NECAP endocytosis associated 1 [Source:MGI Symbol;Acc:MGI:1914852]	Necap1	protein_coding
ENSMUSG00000030401	700.8845478	-0.596785946	0.225925858	0.000479176	0.014411254	Down	20167	reticulon 2 (Z-band associated protein) [Source:MGI Symbol;Acc:MGI:107612]	Rtn2	protein_coding
ENSMUSG00000030403	878.3561864	-0.528163227	0.30646981	0.004411604	0.048208736	Down	22323	vasodilator-stimulated phosphoprotein [Source:MGI Symbol;Acc:MGI:109268]	Vasp	protein_coding
ENSMUSG00000030421	1109.728754	0.256903107	0.102837644	0.004109356	0.04664907	Up	19777	URI1, prefoldin-like chaperone [Source:MGI Symbol;Acc:MGI:1342294]	Uri1	protein_coding
ENSMUSG00000030432	3944.863203	-0.532828649	0.303738985	0.004396738	0.048144948	Down	19943	ribosomal protein L28 [Source:MGI Symbol;Acc:MGI:101839]	Rpl28	protein_coding
ENSMUSG00000030471	243.312301	0.416597541	0.178673213	0.001977162	0.031259108	Up	243983	zinc finger, DHHC domain containing 13 [Source:MGI Symbol;Acc:MGI:1919227]	Zdhhc13	protein_coding
ENSMUSG00000030522	621.0748057	0.336156548	0.116780353	0.000662735	0.017650586	Up	233315	myotubularin related protein 10 [Source:MGI Symbol;Acc:MGI:2142292]	Mtmr10	protein_coding
ENSMUSG00000030538	465.4631847	-0.484478828	0.176437511	0.000510371	0.014983339	Down	23991	calcium and integrin binding 1 (calmyrin) [Source:MGI Symbol;Acc:MGI:1344418]	Cib1	protein_coding
ENSMUSG00000030541	2596.492608	-0.276453266	0.102070186	0.001564534	0.027507171	Down	269951	isocitrate dehydrogenase 2 (NADP+), mitochondrial [Source:MGI Symbol;Acc:MGI:96414]	Idh2	protein_coding
ENSMUSG00000030545	1157.634682	0.488560979	0.177888629	0.000508684	0.014959307	Up	18631	peroxisomal biogenesis factor 11 alpha [Source:MGI Symbol;Acc:MGI:1338788]	Pex11a	protein_coding
ENSMUSG00000030588	534.9345264	-0.542619518	0.149922588	2.41E-05	0.002792968	Down	77254	Yip1 interacting factor homolog B (S. cerevisiae) [Source:MGI Symbol;Acc:MGI:1924504]	Yif1b	protein_coding
ENSMUSG00000030629	2116.375041	0.377564372	0.136589511	0.000824695	0.019702098	Up	65098	zinc finger, AN1-type domain 6 [Source:MGI Symbol;Acc:MGI:1929510]	Zfand6	protein_coding
ENSMUSG00000030671	9344.989014	0.636869419	0.197395781	0.000104616	0.006303682	Up	18576	phosphodiesterase 3B, cGMP-inhibited [Source:MGI Symbol;Acc:MGI:1333863]	Pde3b	protein_coding
ENSMUSG00000030681	1778.865899	-0.442470148	0.137052877	0.000116938	0.006722209	Down	78388	major vault protein [Source:MGI Symbol;Acc:MGI:1925638]	Mvp	protein_coding
ENSMUSG00000030688	239.1866658	-1.003325077	0.51220062	0.001495061	0.026950204	Down	56018	START domain containing 10 [Source:MGI Symbol;Acc:MGI:1860093]	Stard10	protein_coding
ENSMUSG00000030704	1753.178209	0.282219862	0.122252006	0.004421056	0.048228949	Up	19346	RAB6A, member RAS oncogene family [Source:MGI Symbol;Acc:MGI:894313]	Rab6a	protein_coding
ENSMUSG00000030708	231.3547413	-0.633857334	0.272858074	0.001068332	0.022737013	Down	69387	DnaJ heat shock protein family (Hsp40) member B13 [Source:MGI Symbol;Acc:MGI:1916637]	Dnajb13	protein_coding
ENSMUSG00000030744	6019.513243	-0.421906351	0.204157556	0.003601236	0.04339867	Down	27050	ribosomal protein S3 [Source:MGI Symbol;Acc:MGI:1350917]	Rps3	protein_coding
ENSMUSG00000030754	2417.759994	0.278697849	0.118268096	0.004176843	0.04696359	Up	70349	coatomer protein complex, subunit beta 1 [Source:MGI Symbol;Acc:MGI:1917599]	Copb1	protein_coding
ENSMUSG00000030792	6.513686109	-0.069874062	0.189422102	0.003268023	0.041018087	Down	50722	dickkopf-like 1 [Source:MGI Symbol;Acc:MGI:1354963]	Dkkl1	protein_coding
ENSMUSG00000030798	434.1057201	-0.511950544	0.280122832	0.003759706	0.044714298	Down	12493	CD37 antigen [Source:MGI Symbol;Acc:MGI:88330]	Cd37	protein_coding
ENSMUSG00000030814	250.7797505	-0.767074261	0.257026593	0.000148595	0.00753397	Down	12055	B cell CLL/lymphoma 7C [Source:MGI Symbol;Acc:MGI:1332237]	Bcl7c	protein_coding
ENSMUSG00000030846	1015.552413	0.221188915	0.077580862	0.001564359	0.027507171	Up	21843	Tia1 cytotoxic granule-associated RNA binding protein-like 1 [Source:MGI Symbol;Acc:MGI:107913]	Tial1	protein_coding
ENSMUSG00000030850	1004.4226	0.281195303	0.098753056	0.001036396	0.022443242	Up	11907	arginyltransferase 1 [Source:MGI Symbol;Acc:MGI:1333870]	Ate1	protein_coding
ENSMUSG00000030884	3202.652949	0.46339051	0.176854604	0.000789679	0.019413043	Up	67003	ubiquinol cytochrome c reductase core protein 2 [Source:MGI Symbol;Acc:MGI:1914253]	Uqcrc2	protein_coding
ENSMUSG00000030890	15.18640206	-1.132722693	0.427543339	0.000346457	0.011729838	Down	16202	integrin linked kinase [Source:MGI Symbol;Acc:MGI:1195267]	Ilk	protein_coding

ENSMUSG00000030924	240.1424758	0.771405501	0.217823766	2.28E-05	0.002705768	Up	434234	RNA exonuclease 5 [Source:MGI Symbol;Acc:MGI:1919402]	Rexo5	protein_coding
ENSMUSG00000030929	161.1658586	0.468917012	0.213748743	0.002197666	0.033338368	Up	71151	exoribonuclease 2 [Source:MGI Symbol;Acc:MGI:1918401]	Eri2	protein_coding
ENSMUSG00000031012	696.9672162	0.2510755	0.102420785	0.003941288	0.045676004	Up	12361	calcium/calmodulin-dependent serine protein kinase (MAGUK family) [Source:MGI Symbol;Acc:MGI:1309489]	Cask	protein_coding
ENSMUSG00000031072	398.7748224	-0.370722394	0.139886968	0.001043997	0.022532874	Down	72284	ABCE maturation factor [Source:MGI Symbol;Acc:MGI:1919534]	LTO1	protein_coding
ENSMUSG00000031095	838.1819057	0.454410772	0.152550574	0.000287787	0.010440902	Up	72584	cullin 4B [Source:MGI Symbol;Acc:MGI:1919834]	Cul4b	protein_coding
ENSMUSG00000031144	511.9160649	-1.194258815	0.353658502	3.10E-05	0.003266187	Down	20977	synaptophysin [Source:MGI Symbol;Acc:MGI:98467]	Syp	protein_coding
ENSMUSG00000031209	2543.474479	0.331176269	0.115303903	0.000601316	0.016686748	Up	15203	hephaestin [Source:MGI Symbol;Acc:MGI:1332240]	Heph	protein_coding
ENSMUSG00000031256	603.6753646	0.29587056	0.117885027	0.002426323	0.034953036	Up	108062	cleavage stimulation factor, 3' pre-RNA subunit 2 [Source:MGI Symbol;Acc:MGI:1343054]	Cstf2	protein_coding
ENSMUSG00000031289	24.87873365	-0.11055901	0.224475728	0.002813122	0.03749307	Down	16165	interleukin 13 receptor, alpha 2 [Source:MGI Symbol;Acc:MGI:1277954]	Il13ra2	protein_coding
ENSMUSG00000031295	2160.288195	0.38572655	0.185880096	0.004017536	0.045941738	Up	110094	phosphorylase kinase alpha 2 [Source:MGI Symbol;Acc:MGI:97577]	Phka2	protein_coding
ENSMUSG00000031299	5251.837349	0.531907414	0.181428733	0.000261452	0.009990243	Up	18597	pyruvate dehydrogenase E1 alpha 1 [Source:MGI Symbol;Acc:MGI:97532]	Pdha1	protein_coding
ENSMUSG00000031333	400.9139507	0.346655424	0.118289512	0.000535176	0.015438654	Up	11306	ATP-binding cassette, sub-family B (MDR/TAP), member 7 [Source:MGI Symbol;Acc:MGI:109533]	Abcb7	protein_coding
ENSMUSG00000031358	646.4655441	0.279027974	0.095150408	0.000760684	0.018990119	Up	17692	MSL complex subunit 3 [Source:MGI Symbol;Acc:MGI:1341851]	Msl3	protein_coding
ENSMUSG00000031373	9195.459335	0.627636768	0.205382993	0.000100156	0.006096337	Up	56078	carbonic anhydrase 5b, mitochondrial [Source:MGI Symbol;Acc:MGI:1926249]	Car5b	protein_coding
ENSMUSG00000031387	202.5790184	-0.568473448	0.205990917	0.000383669	0.012641988	Down	19703	renin binding protein [Source:MGI Symbol;Acc:MGI:105940]	Renbp	protein_coding
ENSMUSG00000031446	2182.801792	0.198237612	0.07498462	0.003489095	0.042734596	Up	99375	cullin 4A [Source:MGI Symbol;Acc:MGI:1914487]	Cul4a	protein_coding
ENSMUSG00000031523	8406.859046	0.62108392	0.166179165	1.46E-05	0.002198172	Up	50768	deleted in liver cancer 1 [Source:MGI Symbol;Acc:MGI:1354949]	Dlc1	protein_coding
ENSMUSG00000031532	2802.717491	-0.296168746	0.118578739	0.002461733	0.035235081	Down	67887	store-operated calcium entry-associated regulatory factor [Source:MGI Symbol;Acc:MGI:1915137]	Saraf	protein_coding
ENSMUSG00000031539	557.0053071	-0.341497459	0.140170463	0.002189448	0.033272275	Down	64933	adaptor-related protein complex 3, mu 2 subunit [Source:MGI Symbol;Acc:MGI:1929214]	Ap3m2	protein_coding
ENSMUSG00000031554	11.28204109	-0.143922223	0.275589235	0.000728176	0.018535693	Down	11499	a disintegrin and metallopeptidase domain 5 [Source:MGI Symbol;Acc:MGI:104730]	Adam5	protein_coding
ENSMUSG00000031600	733.9709635	0.264939715	0.10451058	0.002731928	0.036924957	Up	52348	vacuolar protein sorting 37A [Source:MGI Symbol;Acc:MGI:1261835]	Vps37a	protein_coding
ENSMUSG00000031616	295.1468502	0.864085658	0.242775831	2.02E-05	0.002580307	Up	13617	endothelin receptor type A [Source:MGI Symbol;Acc:MGI:105923]	Ednra	protein_coding
ENSMUSG00000031652	743.3686254	0.297077295	0.106577461	0.00114651	0.023690413	Up	80750	NEDD4 binding protein 1 [Source:MGI Symbol;Acc:MGI:2136825]	N4bp1	protein_coding
ENSMUSG00000031661	632.3949118	-0.984265668	0.379089288	0.000353663	0.011903661	Down	93960	naked cuticle 1 [Source:MGI Symbol;Acc:MGI:2135954]	Nkd1	protein_coding
ENSMUSG00000031696	3804.185287	0.295358081	0.110953026	0.00160729	0.028006496	Up	65114	VPS35 retromer complex component [Source:MGI Symbol;Acc:MGI:1890467]	Vps35	protein_coding
ENSMUSG00000031727	7.082585852	-0.066070424	0.187106167	0.003469754	0.042649262	Down	56523	polyamine modulated factor 1 binding protein 1 [Source:MGI Symbol;Acc:MGI:1930136]	Pmfbp1	protein_coding
ENSMUSG00000031731	1458.27221	0.264100611	0.106441502	0.003266645	0.041018087	Up	11765	adaptor protein complex AP-1, gamma 1 subunit [Source:MGI Symbol;Acc:MGI:101919]	Ap1g1	protein_coding
ENSMUSG00000031732	417.4626099	0.321996192	0.114247819	0.000861283	0.020139062	Up	244650	PH domain and leucine rich repeat protein phosphatase 2 [Source:MGI Symbol;Acc:MGI:2444928]	Phlpp2	protein_coding
ENSMUSG00000031750	485.242966	-0.488528672	0.135151423	2.82E-05	0.003106886	Down	76527	interleukin 34 [Source:MGI Symbol;Acc:MGI:1923777]	Il34	protein_coding
ENSMUSG00000031767	1088.800045	0.325917238	0.142564916	0.003656989	0.043855876	Up	67528	nudix (nucleoside diphosphate linked moiety X)-type motif 7 [Source:MGI Symbol;Acc:MGI:1914778]	Nudt7	protein_coding
ENSMUSG00000031788	406.7104578	-0.513314572	0.220416619	0.001377847	0.025872156	Down	16582	kinesin family member C3 [Source:MGI Symbol;Acc:MGI:109202]	Kifc3	protein_coding
ENSMUSG00000031813	331.5452231	-0.393566768	0.175042046	0.002604957	0.036231825	Down	73711	multivesicular body subunit 12A [Source:MGI Symbol;Acc:MGI:1920961]	Mvb12a	protein_coding
ENSMUSG00000031821	132.133311	-0.452810536	0.230738044	0.003728412	0.044588284	Down	272551	GINS complex subunit 2 (Psf2 homolog) [Source:MGI Symbol;Acc:MGI:1921019]	Gins2	protein_coding
ENSMUSG00000031827	1156.587597	-0.523429522	0.279287864	0.003734239	0.044596084	Down	72042	coactosin-like 1 (Dictyostelium) [Source:MGI Symbol;Acc:MGI:1919292]	Cotl1	protein_coding
ENSMUSG00000031828	182.2248554	-0.462717611	0.181084888	0.000969217	0.021551591	Down	234796	kelch-like 36 [Source:MGI Symbol;Acc:MGI:2385305]	Klhl36	protein_coding
ENSMUSG00000031831	11.25932727	-0.044987874	0.176934133	0.002421665	0.034942197	Down	68270	dynein, axonemal assembly factor 1 [Source:MGI Symbol;Acc:MGI:1915520]	Dnaaf1	protein_coding
ENSMUSG00000031847	14.3756049	-0.158397527	0.29642332	0.003446771	0.042548859	Down	69528	RIKEN cDNA 1700030J22 gene [Source:MGI Symbol;Acc:MGI:1916778]	1700030J22Rik	protein_coding
ENSMUSG00000031853	40.97978798	-0.093722446	0.20758918	0.002904832	0.038239246	Down	234878	mitogen-activated protein kinase kinase kinase 21 [Source:MGI Symbol;Acc:MGI:2385307]	Map3k21	protein_coding
ENSMUSG00000031860	15.70719995	-0.862044401	0.57306869	0.003627892	0.043597946	Down	80720	pre B cell leukemia homeobox 4 [Source:MGI Symbol;Acc:MGI:1931321]	Pbx4	protein_coding
ENSMUSG00000031864	701.9624553	0.28015687	0.092711904	0.000583912	0.016308839	Up	70885	integrator complex subunit 10 [Source:MGI Symbol;Acc:MGI:1918135]	Ints10	protein_coding
ENSMUSG00000031880	83.37989531	-1.111152074	0.658342922	0.002246439	0.033722017	Down	56437	Ras-related associated with diabetes [Source:MGI Symbol;Acc:MGI:1930943]	Rrad	protein_coding
ENSMUSG00000031902	1374.386503	0.341759592	0.144007324	0.002573358	0.036024921	Up	18021	nuclear factor of activated T cells, cytoplasmic, calcineurin dependent 3 [Source:MGI Symbol;Acc:MGI:103296]	Nfatc3	protein_coding
ENSMUSG00000031918	998.3481137	0.264030344	0.068684842	3.42E-05	0.003431462	Up	77116	myotubularin related protein 2 [Source:MGI Symbol;Acc:MGI:1924366]	Mtmr2	protein_coding
ENSMUSG00000031979	1073.114114	0.304184289	0.128516001	0.003438458	0.04250714	Up	76332	component of oligomeric golgi complex 2 [Source:MGI Symbol;Acc:MGI:1923582]	Cog2	protein_coding
ENSMUSG00000031990	182.3634238	-0.48073287	0.198684159	0.001233043	0.024468519	Down	83964	junction adhesion molecule 3 [Source:MGI Symbol;Acc:MGI:1933825]	Jam3	protein_coding
ENSMUSG00000031995	36.31028583	-0.132163493	0.253735611	0.001731496	0.029034732	Down	19143	suppression of tumorigenicity 14 (colon carcinoma) [Source:MGI Symbol;Acc:MGI:1338881]	St14	protein_coding
ENSMUSG00000032011	402.8405005	-0.490409265	0.222445191	0.001950562	0.030980672	Down	21838	thymus cell antigen 1, theta [Source:MGI Symbol;Acc:MGI:98747]	Thy1	protein_coding
ENSMUSG00000032030	852.9434495	0.317553795	0.086068776	4.73E-05	0.004134689	Up	75717	cullin 5 [Source:MGI Symbol;Acc:MGI:1922967]	Cul5	protein_coding
ENSMUSG00000032058	6204.738443	0.548012403	0.162335646	6.43E-05	0.004869134	Up	73699	protein phosphatase 2, regulatory subunit A, beta [Source:MGI Symbol;Acc:MGI:1920949]	Ppp2r1b	protein_coding
ENSMUSG00000032119	348.2954324	0.359489765	0.103561822	7.98E-05	0.005299588	Up	102423	histone H4 transcription factor [Source:MGI Symbol;Acc:MGI:2429620]	Hinfp	protein_coding
ENSMUSG00000032217	743.2300677	0.50306138	0.106784708	2.57E-07	0.000233309	Up	93836	ring finger 111 [Source:MGI Symbol;Acc:MGI:1934919]	Rnf111	protein_coding
ENSMUSG00000032231	16046.36374	-0.377959562	0.187313863	0.004635221	0.049578114	Down	12306	annexin A2 [Source:MGI Symbol;Acc:MGI:88246]	Anxa2	protein_coding
ENSMUSG00000032246	123.1890426	-1.290589263	0.473960975	0.000223435	0.009224534	Down	75600	calmodulin-like 4 [Source:MGI Symbol;Acc:MGI:1922850]	Calml4	protein_coding
ENSMUSG00000032264	318.1620063	0.34700947	0.124143465	0.000737042	0.018706115	Up	26951	zw10 kinetochore protein [Source:MGI Symbol;Acc:MGI:1349478]	Zw10	protein_coding
ENSMUSG00000032285	281.9566372	-1.512111532	0.561527383	0.000226098	0.009224534	Down	58233	DnaJ heat shock protein family (Hsp40) member A4 [Source:MGI Symbol;Acc:MGI:1927638]	Dnaja4	protein_coding

ENSMUSG00000032307	1398.441744	0.307471262	0.110456936	0.001009531	0.022038737	Up	109161	ubiquitin-conjugating enzyme E2Q family member 2 [Source:MGI Symbol;Acc:MGI:2388672]	Ube2q2	protein_coding
ENSMUSG00000032323	9.214414353	-1.104710132	0.552689247	0.001540635	0.027286494	Down	13070	cytochrome P450, family 11, subfamily a, polypeptide 1 [Source:MGI Symbol;Acc:MGI:88582]	Cyp11a1	protein_coding
ENSMUSG00000032359	1309.621242	-0.35585613	0.16652219	0.003994444	0.045860266	Down	13036	cathepsin H [Source:MGI Symbol;Acc:MGI:107285]	Ctsh	protein_coding
ENSMUSG00000032366	1738.720622	-0.41006802	0.117102007	5.35E-05	0.004409981	Down	22003	tropomyosin 1, alpha [Source:MGI Symbol;Acc:MGI:98809]	Tpm1	protein_coding
ENSMUSG00000032369	148.6129272	-0.753175102	0.224702863	4.52E-05	0.004073797	Down	22038	phospholipid scramblase 1 [Source:MGI Symbol;Acc:MGI:893575]	Plscr1	protein_coding
ENSMUSG00000032373	32.26402459	-0.165143682	0.315800112	0.001846681	0.030090232	Down	76459	carbonic anhydrase 12 [Source:MGI Symbol;Acc:MGI:1923709]	Car12	protein_coding
ENSMUSG00000032386	831.8672726	0.306461188	0.082981782	4.70E-05	0.004134689	Up	56404	thyroid hormone receptor interactor 4 [Source:MGI Symbol;Acc:MGI:1928469]	Trip4	protein_coding
ENSMUSG00000032398	321.0032507	-0.465671269	0.213920967	0.002270321	0.033800769	Down	330959	small nuclear RNA activating complex, polypeptide 5 [Source:MGI Symbol;Acc:MGI:1914282]	Snapc5	protein_coding
ENSMUSG00000032407	1082.6473	0.343298478	0.116224733	0.000490916	0.014636612	Up	67958	U2 snRNP-associated SURP domain containing [Source:MGI Symbol;Acc:MGI:1915208]	U2surp	protein_coding
ENSMUSG00000032413	949.6569566	0.412895105	0.154667374	0.000829702	0.019736989	Up	114713	RAS p21 protein activator 2 [Source:MGI Symbol;Acc:MGI:2149960]	Rasa2	protein_coding
ENSMUSG00000032418	19071.92353	0.815216991	0.288417021	0.000229101	0.009224534	Up	17436	malic enzyme 1, NADP(+)-dependent, cytosolic [Source:MGI Symbol;Acc:MGI:97043]	Me1	protein_coding
ENSMUSG00000032423	2300.641823	0.324248769	0.098562488	0.000197846	0.008556482	Up	56403	synaptotagmin binding, cytoplasmic RNA interacting protein [Source:MGI Symbol;Acc:MGI:1891690]	Syncrip	protein_coding
ENSMUSG00000032431	1868.10613	-0.225700141	0.085428139	0.002705984	0.036812084	Down	56693	cartilage associated protein [Source:MGI Symbol;Acc:MGI:1891221]	Crtap	protein_coding
ENSMUSG00000032437	6144.515168	0.283479172	0.104867702	0.001564798	0.027507171	Up	68292	STT3, subunit of the oligosaccharyltransferase complex, homolog B (S. cerevisiae) [Source:MGI Symbol;Acc:MGI:1915542]	Stt3b	protein_coding
ENSMUSG00000032449	1486.501296	0.259916729	0.089689588	0.001001127	0.021977601	Up	192287	solute carrier family 25, member 36 [Source:MGI Symbol;Acc:MGI:1924909]	Slc25a36	protein_coding
ENSMUSG00000032462	1566.263048	0.582075928	0.193480121	0.000189074	0.00835522	Up	74769	phosphatidylinositol-4,5-bisphosphate 3-kinase catalytic subunit beta [Source:MGI Symbol;Acc:MGI:1922019]	Pik3cb	protein_coding
ENSMUSG00000032489	65.65108137	-1.10779091	0.525403678	0.001067234	0.022737013	Down	16578	kinesin family member 9 [Source:MGI Symbol;Acc:MGI:1098237]	Kif9	protein_coding
ENSMUSG00000032492	194.0666534	1.037556858	0.352538752	0.000135395	0.007179257	Up	19228	parathyroid hormone 1 receptor [Source:MGI Symbol;Acc:MGI:97801]	Pth1r	protein_coding
ENSMUSG00000032500	133.7908789	-1.850224471	0.452231454	1.79E-06	0.000720041	Down	245038	doublecortin-like kinase 3 [Source:MGI Symbol;Acc:MGI:3039580]	Dclk3	protein_coding
ENSMUSG00000032504	3804.038699	0.273642105	0.093449397	0.000771826	0.019083029	Up	18571	programmed cell death 6 interacting protein [Source:MGI Symbol;Acc:MGI:1333753]	Pdcd6ip	protein_coding
ENSMUSG00000032519	346.3042205	-0.266653166	0.104169095	0.002533702	0.035682028	Down	208638	solute carrier family 25, member 38 [Source:MGI Symbol;Acc:MGI:2384782]	Slc25a38	protein_coding
ENSMUSG00000032540	2080.701746	0.420705966	0.154223575	0.000697943	0.018169318	Up	67469	abhydrolase domain containing 5 [Source:MGI Symbol;Acc:MGI:1914719]	Abhd5	protein_coding
ENSMUSG00000032562	6688.844356	-0.274766573	0.091285456	0.000705583	0.018307504	Down	14678	guanine nucleotide binding protein (G protein), alpha inhibiting 2 [Source:MGI Symbol;Acc:MGI:95772]	Gnai2	protein_coding
ENSMUSG00000032580	1962.56922	0.310281442	0.119885829	0.001653084	0.028430729	Up	83486	RNA binding motif protein 5 [Source:MGI Symbol;Acc:MGI:1933204]	Rbm5	protein_coding
ENSMUSG00000032680	18.22059867	-0.077108278	0.194476147	0.002282946	0.033800769	Down	228778	RIKEN cDNA 6820408C15 gene [Source:MGI Symbol;Acc:MGI:3045333]	6820408C15Rik	protein_coding
ENSMUSG00000032715	147.0655187	-0.851497494	0.294820667	0.000183257	0.008267175	Down	228775	tribbles pseudokinase 3 [Source:MGI Symbol;Acc:MGI:1345675]	Trib3	protein_coding
ENSMUSG00000032717	12.4740192	-1.454532209	0.515854291	0.000190541	0.00835522	Down	17240	MyoD family inhibitor [Source:MGI Symbol;Acc:MGI:107687]	Mdfi	protein_coding
ENSMUSG00000032725	210.4210818	-0.780639346	0.346489189	0.001010306	0.022038737	Down	14276	folate receptor 2 (fetal) [Source:MGI Symbol;Acc:MGI:95569]	Folr2	protein_coding
ENSMUSG00000032727	406.0706245	0.381190616	0.13255323	0.000531063	0.015375846	Up	218613	MIER family member 3 [Source:MGI Symbol;Acc:MGI:2442317]	Mier3	protein_coding
ENSMUSG00000032763	562.8061411	-0.360156166	0.121838003	0.000460815	0.014186548	Down	216136	ilvB (bacterial acetolactate synthase)-like [Source:MGI Symbol;Acc:MGI:1351911]	Ilvbl	protein_coding
ENSMUSG00000032959	2520.406112	-0.409917573	0.176424065	0.002032675	0.031844633	Down	23980	phosphatidylethanolamine binding protein 1 [Source:MGI Symbol;Acc:MGI:1344408]	Pebp1	protein_coding
ENSMUSG00000032977	278.164333	-0.362790122	0.158245011	0.00264793	0.036638703	Down	108707	family with sequence similarity 207, member A [Source:MGI Symbol;Acc:MGI:1916334]	Fam207a	protein_coding
ENSMUSG00000032997	1223.668469	-0.374775121	0.16107541	0.00228105	0.033800769	Down	74241	chondroitin polymerizing factor [Source:MGI Symbol;Acc:MGI:106576]	Chpf	protein_coding
ENSMUSG00000032998	821.0364507	0.333533489	0.133874272	0.002106646	0.03255949	Up	230700	forkhead box J3 [Source:MGI Symbol;Acc:MGI:2443432]	Foxj3	protein_coding
ENSMUSG00000033020	446.2765631	-0.454952565	0.162607402	0.000518149	0.015123297	Down	69833	polymerase (RNA) II (DNA directed) polypeptide F [Source:MGI Symbol;Acc:MGI:1349393]	Polr2f	protein_coding
ENSMUSG00000033029	23.20429023	-1.266833527	0.492320022	0.000357028	0.011923763	Down	27660	RIKEN cDNA 1700088E04 gene [Source:MGI Symbol;Acc:MGI:1920774]	1700088E04Rik	protein_coding
ENSMUSG00000033152	151.6743101	-0.743152363	0.258956538	0.00021019	0.008965847	Down	319655	podocalyxin-like 2 [Source:MGI Symbol;Acc:MGI:2442488]	Podxl2	protein_coding
ENSMUSG00000033161	3204.641774	-0.735390201	0.257464297	0.00022843	0.009224534	Down	11928	ATPase, Na+/K+ transporting, alpha 1 polypeptide [Source:MGI Symbol;Acc:MGI:88105]	Atp1a1	protein_coding
ENSMUSG00000033208	39.29049341	-1.687648721	0.485501364	2.08E-05	0.002600071	Down	20203	S100 protein, beta polypeptide, neural [Source:MGI Symbol;Acc:MGI:98217]	S100b	protein_coding
ENSMUSG00000033276	57.07217605	-0.819654958	0.537354842	0.003482716	0.042717186	Down	269209	serine/threonine kinase 36 [Source:MGI Symbol;Acc:MGI:1920831]	Stk36	protein_coding
ENSMUSG00000033295	1204.66993	-0.714390703	0.293149258	0.000680127	0.017894095	Down	19268	protein tyrosine phosphatase, receptor type, F [Source:MGI Symbol;Acc:MGI:102695]	Ptprf	protein_coding
ENSMUSG00000033307	701.6129282	-0.520337497	0.213530324	0.001064464	0.022737013	Down	17319	macrophage migration inhibitory factor (glycosylation-inhibiting factor) [Source:MGI Symbol;Acc:MGI:96982]	Mif	protein_coding
ENSMUSG00000033313	118.7881996	-0.471684437	0.199198352	0.001457132	0.02643237	Down	50788	F-box and leucine-rich repeat protein 8 [Source:MGI Symbol;Acc:MGI:1354697]	Fbxl8	protein_coding
ENSMUSG00000033352	854.0385056	0.32621149	0.100674357	0.000222087	0.009224534	Up	26398	mitogen-activated protein kinase kinase 4 [Source:MGI Symbol;Acc:MGI:1346869]	Map2k4	protein_coding
ENSMUSG00000033382	1486.424598	0.264578977	0.109759759	0.003924166	0.045600241	Up	75964	trafficking protein particle complex 8 [Source:MGI Symbol;Acc:MGI:2443008]	Trappc8	protein_coding
ENSMUSG00000033400	1183.186184	0.301411234	0.117167377	0.001966936	0.031211973	Up	77559	amylo-1,6-glucosidase, 4-alpha-glucanotransferase [Source:MGI Symbol;Acc:MGI:1924809]	Agl	protein_coding
ENSMUSG00000033460	240.7461687	-0.301464925	0.13070233	0.00383007	0.045022918	Down	78248	armadillo repeat containing, X-linked 1 [Source:MGI Symbol;Acc:MGI:1925498]	Armcx1	protein_coding
ENSMUSG00000033467	577.3482422	-0.672419022	0.263507539	0.000568325	0.016114087	Down	57914	cytokine receptor-like factor 2 [Source:MGI Symbol;Acc:MGI:1889506]	Crlf2	protein_coding
ENSMUSG00000033581	189.9840627	0.499888589	0.231237942	0.002100095	0.0325458	Up	319765	insulin-like growth factor 2 mRNA binding protein 2 [Source:MGI Symbol;Acc:MGI:1890358]	Igf2bp2	protein_coding
ENSMUSG00000033585	713.124545	-0.624790835	0.191221808	7.30E-05	0.005101093	Down	17984	necdin, MAGE family member [Source:MGI Symbol;Acc:MGI:97290]	Ndn	protein_coding

ENSMUSG00000033596	592.4068395	0.265797627	0.078550356	0.000189673	0.00835522	Up	234736	ring finger and WD repeat domain 3 [Source:MGI Symbol;Acc:MGI:2384584]	Rfwd3	protein_coding
ENSMUSG00000033713	1185.41801	0.326729085	0.120767672	0.001171912	0.024013736	Up	71375	forkhead box N3 [Source:MGI Symbol;Acc:MGI:1918625]	Foxn3	protein_coding
ENSMUSG00000033722	180.5350539	-0.646629357	0.341148005	0.002475218	0.03526538	Down	207792	cDNA sequence BC034090 [Source:MGI Symbol;Acc:MGI:2672904]	BC034090	protein_coding
ENSMUSG00000033760	311.5062523	0.320955521	0.143297378	0.003919743	0.045585372	Up	66704	RNA binding motif protein 4B [Source:MGI Symbol;Acc:MGI:1913954]	Rbm4b	protein_coding
ENSMUSG00000033768	95.8273839	-1.159077074	0.326932329	1.71E-05	0.002413321	Down	18190	neurexin II [Source:MGI Symbol;Acc:MGI:1096362]	Nrxn2	protein_coding
ENSMUSG00000033813	945.3063373	0.340720725	0.1588345	0.004275259	0.0474291	Up	21399	transcription elongation factor A (SII) 1 [Source:MGI Symbol;Acc:MGI:1196624]	Tcea1	protein_coding
ENSMUSG00000033819	822.2110376	-0.328037318	0.108332354	0.000438503	0.013865547	Down	73062	protein phosphatase 1, regulatory subunit 16A [Source:MGI Symbol;Acc:MGI:1920312]	Ppp1r16a	protein_coding
ENSMUSG00000033826	17.339884	-1.816864846	0.764477718	0.000503529	0.014833024	Down	13417	dynein, axonemal, heavy chain 8 [Source:MGI Symbol;Acc:MGI:107714]	Dnah8	protein_coding
ENSMUSG00000033849	5842.20717	0.971072815	0.291997645	4.18E-05	0.003911493	Up	26878	UDP-Gal:betaGlcNAc beta 1,3-galactosyltransferase, polypeptide 2 [Source:MGI Symbol;Acc:MGI:1349461]	B3galt2	protein_coding
ENSMUSG00000033871	91.68378066	0.80779848	0.284140038	0.000214407	0.009078301	Up	170826	peroxisome proliferative activated receptor, gamma, coactivator 1 beta [Source:MGI Symbol;Acc:MGI:2444934]	Ppargc1b	protein_coding
ENSMUSG00000033883	297.8929193	0.36479869	0.150800347	0.001974212	0.031241133	Up	241944	zinc finger protein 267 [Source:MGI Symbol;Acc:MGI:1098769]	Zfp267	protein_coding
ENSMUSG00000033898	11.57589288	1.864429576	1.159743411	0.002146634	0.032795163	Up	545366	complement factor H-related 2 [Source:MGI Symbol;Acc:MGI:3611575]	Cfhr2	protein_coding
ENSMUSG00000033964	751.9960306	0.297154404	0.101844831	0.000742443	0.018765676	Up	226470	zinc finger and BTB domain containing 41 [Source:MGI Symbol;Acc:MGI:2444487]	Zbtb41	protein_coding
ENSMUSG00000034021	704.5356307	0.34324316	0.131910086	0.001327238	0.025315087	Up	100710	PDS5 cohesin associated factor B [Source:MGI Symbol;Acc:MGI:2140945]	Pds5b	protein_coding
ENSMUSG00000034035	112.7706959	-0.504555068	0.283557352	0.00433375	0.047843379	Down	622665	coiled-coil domain containing 17 [Source:MGI Symbol;Acc:MGI:1915667]	Ccdc17	protein_coding
ENSMUSG00000034041	290.4617532	-0.510939078	0.27596921	0.00375347	0.04470183	Down	17095	lymphoblastomic leukemia 1 [Source:MGI Symbol;Acc:MGI:96891]	Lyl1	protein_coding
ENSMUSG00000034042	904.1109738	0.40445179	0.094450387	2.51E-06	0.000864464	Up	77110	GC-rich promoter binding protein 1-like 1 [Source:MGI Symbol;Acc:MGI:1924360]	Gpbp1l1	protein_coding
ENSMUSG00000034108	366.6439066	-0.639768197	0.210390177	0.000148428	0.00753397	Down	12460	copper chaperone for superoxide dismutase [Source:MGI Symbol;Acc:MGI:1333783]	Ccs	protein_coding
ENSMUSG00000034157	587.3994043	0.332599078	0.114738676	0.000627376	0.017106903	Up	217732	CLOCK interacting protein, circadian [Source:MGI Symbol;Acc:MGI:1919185]	Cipc	protein_coding
ENSMUSG00000034158	4759.046973	0.498508673	0.12010915	3.45E-06	0.000946988	Up	320184	leucine rich repeat containing 58 [Source:MGI Symbol;Acc:MGI:2443542]	Lrrc58	protein_coding
ENSMUSG00000034194	408.0641544	-0.504609811	0.230515748	0.0019412	0.030905523	Down	71843	R3H domain and coiled-coil containing 1 [Source:MGI Symbol;Acc:MGI:1919093]	R3hcc1	protein_coding
ENSMUSG00000034245	133.9253782	-1.165584673	0.232885136	2.86E-08	6.29E-05	Down	232232	histone deacetylase 11 [Source:MGI Symbol;Acc:MGI:2385252]	Hdac11	protein_coding
ENSMUSG00000034282	159.7152528	-0.652865969	0.433667288	0.004495481	0.048662455	Down	14027	envoplakin [Source:MGI Symbol;Acc:MGI:107507]	Evpl	protein_coding
ENSMUSG00000034353	72.50152493	-1.289312596	0.311294379	1.62E-06	0.000679737	Down	51801	receptor (calcitonin) activity modifying protein 1 [Source:MGI Symbol;Acc:MGI:1858418]	Ramp1	protein_coding
ENSMUSG00000034463	704.5095783	-0.46399667	0.204552171	0.001810095	0.029736295	Down	219151	scavenger receptor class A, member 3 [Source:MGI Symbol;Acc:MGI:2444418]	Scara3	protein_coding
ENSMUSG00000034467	15.88149264	-0.051449402	0.17966174	0.002637669	0.036539346	Down	75465	dynein light chain roadblock-type 2 [Source:MGI Symbol;Acc:MGI:1922715]	Dynlrb2	protein_coding
ENSMUSG00000034480	735.6820395	0.429857453	0.208786641	0.003482174	0.042717186	Up	54004	diaphanous related formin 2 [Source:MGI Symbol;Acc:MGI:1858500]	Diaph2	protein_coding
ENSMUSG00000034543	863.1329347	0.339481843	0.130539127	0.001421214	0.026099657	Up	74522	microrchidia 2A [Source:MGI Symbol;Acc:MGI:1921772]	Morc2a	protein_coding
ENSMUSG00000034557	564.096524	0.363739784	0.133738621	0.000898129	0.020691796	Up	230597	zinc finger, FYVE domain containing 9 [Source:MGI Symbol;Acc:MGI:2652838]	Zfyve9	protein_coding
ENSMUSG00000034570	35.88373575	-0.943681601	0.600667447	0.002974026	0.038516848	Down	170835	inositol polyphosphate 5-phosphatase J [Source:MGI Symbol;Acc:MGI:2158663]	Inpp5j	protein_coding
ENSMUSG00000034612	344.4663602	0.420765922	0.217104478	0.00460628	0.049427168	Up	58250	carbohydrate sulfotransferase 11 [Source:MGI Symbol;Acc:MGI:1927166]	Chst11	protein_coding
ENSMUSG00000034634	26.04623371	-0.068042393	0.188520178	0.002243969	0.033722017	Down	17068	lymphocyte antigen 6 complex, locus D [Source:MGI Symbol;Acc:MGI:96881]	Ly6d	protein_coding
ENSMUSG00000034636	1808.545845	0.327286182	0.14247915	0.003435743	0.042504065	Up	414872	zyg-ll family member B, cell cycle regulator [Source:MGI Symbol;Acc:MGI:2685277]	Zyg11b	protein_coding
ENSMUSG00000034641	272.5227384	0.391201743	0.175766047	0.002809039	0.037467626	Up	217305	CD300 molecule like family member d [Source:MGI Symbol;Acc:MGI:2442358]	Cd300ld	protein_coding
ENSMUSG00000034645	84.3791516	1.464040807	0.5118321	0.000144165	0.007505743	Up	230590	zyg-11 family member A, cell cycle regulator [Source:MGI Symbol;Acc:MGI:2446208]	Zyg11a	protein_coding
ENSMUSG00000034667	1346.129526	0.174622755	0.067397656	0.004399337	0.048144948	Up	73192	exportin, tRNA (nuclear export receptor for tRNAs) [Source:MGI Symbol;Acc:MGI:1920442]	Xpot	protein_coding
ENSMUSG00000034675	970.3915577	-0.44679334	0.170062674	0.000810286	0.019613515	Down	56320	drebrin 1 [Source:MGI Symbol;Acc:MGI:1931838]	Dbn1	protein_coding
ENSMUSG00000034685	137.6179781	-0.707500407	0.192269545	1.38E-05	0.002132486	Down	217219	family with sequence similarity 171, member A2 [Source:MGI Symbol;Acc:MGI:2448496]	Fam171a2	protein_coding
ENSMUSG00000034686	28.91805823	-0.726171637	0.502748435	0.004458009	0.04853119	Down	432763	proline rich 7 (synaptic) [Source:MGI Symbol;Acc:MGI:3487246]	Prr7	protein_coding
ENSMUSG00000034706	25.45450121	-1.944378581	0.653857423	9.53E-05	0.005868571	Down	432611	dynein axonemal intermediate chain 2 [Source:MGI Symbol;Acc:MGI:2685574]	Dnai2	protein_coding
ENSMUSG00000034724	770.4515062	0.336477266	0.113673832	0.000496092	0.014714561	Up	231464	CCR4-NOT transcription complex, subunit 6-like [Source:MGI Symbol;Acc:MGI:2443154]	Cnot6l	protein_coding
ENSMUSG00000034786	183.1782007	-0.606903187	0.244970061	0.000750003	0.018813405	Down	106512	G-protein signalling modulator 3 (AGS3-like, C. elegans) [Source:MGI Symbol;Acc:MGI:2146785]	Gpsm3	protein_coding
ENSMUSG00000034801	898.9060326	0.391914797	0.103273967	2.25E-05	0.002705768	Up	20663	SOS Ras/Rho guanine nucleotide exchange factor 2 [Source:MGI Symbol;Acc:MGI:98355]	Sos2	protein_coding
ENSMUSG00000034853	58.78955335	-0.820509645	0.416214118	0.001703234	0.028849913	Down	329910	acyl-CoA thioesterase 11 [Source:MGI Symbol;Acc:MGI:1913736]	Acot11	protein_coding
ENSMUSG00000034881	29.86098498	-0.784745478	0.455846379	0.002867773	0.037986425	Down	21390	thromboxane A2 receptor [Source:MGI Symbol;Acc:MGI:98496]	Tbxa2r	protein_coding
ENSMUSG00000034917	37.29819262	-1.657107421	0.75985245	0.000742869	0.018765676	Down	27375	tight junction protein 3 [Source:MGI Symbol;Acc:MGI:1351650]	Tjp3	protein_coding
ENSMUSG00000034932	501.113644	-0.704893291	0.246715695	0.000228658	0.009224534	Down	66047	mitochondrial ribosomal protein L54 [Source:MGI Symbol;Acc:MGI:1913297]	Mrpl54	protein_coding
ENSMUSG00000034981	581.0976043	1.002756038	0.344742596	0.000150587	0.007565779	Up	231440	prostate androgen-regulated mucin-like protein 1 [Source:MGI Symbol;Acc:MGI:2443349]	Parm1	protein_coding
ENSMUSG00000034993	2433.84377	-0.323108638	0.101287948	0.000236297	0.009412881	Down	26949	vesicle amine transport 1 [Source:MGI Symbol;Acc:MGI:1349450]	Vat1	protein_coding
ENSMUSG00000035024	426.1221964	0.329777149	0.151729999	0.004482791	0.048662455	Up	78658	non-SMC condensin II complex, subunit D3 [Source:MGI Symbol;Acc:MGI:2142989]	Ncapd3	protein_coding
ENSMUSG00000035095	39.18069593	-0.142482893	0.269422164	0.002277474	0.033800769	Down	219148	family with sequence similarity 167, member A [Source:MGI Symbol;Acc:MGI:3606565]	Fam167a	protein_coding

ENSMUSG00000035126	121.9617783	-0.729381381	0.424860728	0.002954549	0.038485071	Down	242584	dynein axonemal intermediate chain 4 [Source:MGI Symbol;Acc:MGI:2385328]	Dnai4	protein_coding
ENSMUSG00000035133	2229.737088	0.423966424	0.162903468	0.001009853	0.022038737	Up	11855	Rho GTPase activating protein 5 [Source:MGI Symbol;Acc:MGI:1332637]	Arhgap5	protein_coding
ENSMUSG00000035150	1181.853534	0.290637555	0.102261324	0.000915793	0.020957408	Up	26905	eukaryotic translation initiation factor 2, subunit 3, structural gene X-linked [Source:MGI Symbol;Acc:MGI:1349431]	Eif2s3x	protein_coding
ENSMUSG00000035151	781.5800305	0.401986486	0.124169164	0.000144789	0.007509348	Up	244548	ELMO/CED-12 domain containing 2 [Source:MGI Symbol;Acc:MGI:2445165]	Elmod2	protein_coding
ENSMUSG00000035161	467.9961185	0.396178542	0.180540593	0.002946728	0.038470425	Up	18130	integrator complex subunit 6 [Source:MGI Symbol;Acc:MGI:1202397]	Ints6	protein_coding
ENSMUSG00000035212	1183.265052	-0.308041831	0.103175029	0.000530373	0.015375846	Down	230514	leptin receptor overlapping transcript [Source:MGI Symbol;Acc:MGI:2687005]	Leprot	protein_coding
ENSMUSG00000035234	183.1551563	0.402383297	0.126545807	0.000180763	0.008185824	Up	70681	BRCA1 A complex subunit [Source:MGI Symbol;Acc:MGI:1917931]	Abraxas1	protein_coding
ENSMUSG00000035268	553.2867818	-0.566178195	0.176552893	9.45E-05	0.005856894	Down	18769	protein kinase inhibitor, gamma [Source:MGI Symbol;Acc:MGI:1343086]	Pkig	protein_coding
ENSMUSG00000035476	861.2516434	0.548851181	0.235763575	0.001328591	0.025315087	Up	66724	TGF-beta activated kinase 1/MAP3K7 binding protein 3 [Source:MGI Symbol;Acc:MGI:1913974]	Tab3	protein_coding
ENSMUSG00000035505	296.0028974	0.38341904	0.16141185	0.002030226	0.031835195	Up	231430	cytochrome c oxidase assembly protein 18 [Source:MGI Symbol;Acc:MGI:2448532]	Cox18	protein_coding
ENSMUSG00000035578	34.07799587	-1.320988317	0.649232411	0.001129598	0.023453446	Down	69707	IQ motif containing G [Source:MGI Symbol;Acc:MGI:1916957]	Iqcg	protein_coding
ENSMUSG00000035597	823.1700234	0.325087138	0.147064787	0.003889831	0.045354164	Up	328110	pre-mRNA processing factor 39 [Source:MGI Symbol;Acc:MGI:104602]	Prpf39	protein_coding
ENSMUSG00000035725	634.6898611	0.365338785	0.147710914	0.001745115	0.029169321	Up	19108	protein kinase, X-linked [Source:MGI Symbol;Acc:MGI:1309999]	Prkx	protein_coding
ENSMUSG00000035759	91.53451813	0.472181712	0.216750169	0.002245109	0.033722017	Up	71769	Bardet-Biedl syndrome 10 (human) [Source:MGI Symbol;Acc:MGI:1919019]	Bbs10	protein_coding
ENSMUSG00000035762	235.1559848	0.447662027	0.128504672	5.34E-05	0.004409981	Up	72745	transmembrane protein 161B [Source:MGI Symbol;Acc:MGI:1919995]	Tmem161b	protein_coding
ENSMUSG00000035769	95.01675143	-0.212357163	0.48333743	0.001786738	0.029521437	Down	102448	xylulokinase homolog (H. influenzae) [Source:MGI Symbol;Acc:MGI:2142985]	Xylb	protein_coding
ENSMUSG00000035772	518.9217069	0.339694792	0.14038966	0.002308108	0.034019775	Up	118451	mitochondrial ribosomal protein S2 [Source:MGI Symbol;Acc:MGI:2153089]	Mrps2	protein_coding
ENSMUSG00000035948	1723.448613	0.454555387	0.187328749	0.001441338	0.026307269	Up	380660	acyl-CoA synthetase short-chain family member 3 [Source:MGI Symbol;Acc:MGI:2685720]	Acss3	protein_coding
ENSMUSG00000035992	801.996398	0.387512917	0.160321876	0.00176431	0.029347838	Up	216742	folliculin interacting protein 1 [Source:MGI Symbol;Acc:MGI:2444668]	Fnip1	protein_coding
ENSMUSG00000036006	138.6122068	-0.568434142	0.332688814	0.003984343	0.045835427	Down	193385	RHO family interacting cell polarization regulator 2 [Source:MGI Symbol;Acc:MGI:2444879]	Ripor2	protein_coding
ENSMUSG00000036053	548.4055247	0.347256445	0.151391813	0.002973616	0.038516848	Up	71409	formin-like 2 [Source:MGI Symbol;Acc:MGI:1918659]	Fmnl2	protein_coding
ENSMUSG00000036091	12.52830354	-0.953207585	0.579056115	0.002916625	0.038280426	Down	109685	hyaluronoglucosaminidase 3 [Source:MGI Symbol;Acc:MGI:1330288]	Hyal3	protein_coding
ENSMUSG00000036093	1412.422029	0.259826127	0.088600368	0.000917122	0.020957408	Up	75423	ADP-ribosylation factor-like 5A [Source:MGI Symbol;Acc:MGI:1922673]	Arl5a	protein_coding
ENSMUSG00000036097	639.8858644	0.335844391	0.154056906	0.004340565	0.047887934	Up	226151	SMC5-SMC6 complex localization factor 2 [Source:MGI Symbol;Acc:MGI:1924968]	Slf2	protein_coding
ENSMUSG00000036106	642.8445595	-0.506525219	0.162724899	0.000155951	0.007656725	Down	109270	proline rich 5 (renal) [Source:MGI Symbol;Acc:MGI:1924714]	Prr5	protein_coding
ENSMUSG00000036167	872.9615765	0.317633652	0.107593526	0.000682915	0.017940047	Up	223828	periphilin 1 [Source:MGI Symbol;Acc:MGI:1917029]	Pphln1	protein_coding
ENSMUSG00000036199	1369.036868	-0.426872462	0.204718653	0.003285409	0.041176327	Down	67184	NADH:ubiquinone oxidoreductase subunit A13 [Source:MGI Symbol;Acc:MGI:1914434]	Ndufa13	protein_coding
ENSMUSG00000036256	5808.750243	-0.551811958	0.165538543	6.46E-05	0.004869134	Down	29817	insulin-like growth factor binding protein 7 [Source:MGI Symbol;Acc:MGI:1352480]	Igfbp7	protein_coding
ENSMUSG00000036257	4193.069678	0.341172147	0.106471731	0.00024589	0.009696605	Up	67452	patatin-like phospholipase domain containing 8 [Source:MGI Symbol;Acc:MGI:1914702]	Pnpla8	protein_coding
ENSMUSG00000036275	3262.917532	-0.228039238	0.077515684	0.001177478	0.024042026	Down	213673	RIKEN cDNA 9530068E07 gene [Source:MGI Symbol;Acc:MGI:2654705]	9530068E07Rik	protein_coding
ENSMUSG00000036282	681.7654566	0.344899745	0.089583072	2.04E-05	0.002582034	Up	70646	N(alpha)-acetyltransferase 30, NatC catalytic subunit [Source:MGI Symbol;Acc:MGI:1922259]	Naa30	protein_coding
ENSMUSG00000036323	1866.213439	0.231771172	0.092753023	0.003857001	0.045255836	Up	66661	signal recognition particle 72 [Source:MGI Symbol;Acc:MGI:1333795]	Srp72	protein_coding
ENSMUSG00000036390	189.730981	-0.820121309	0.362176019	0.000947536	0.021358851	Down	13197	growth arrest and DNA-damage-inducible 45 alpha [Source:MGI Symbol;Acc:MGI:107799]	Gadd45a	protein_coding
ENSMUSG00000036391	790.6036278	0.520015644	0.151991066	5.33E-05	0.004409981	Up	77371	Sec24 related gene family, member A (S. cerevisiae) [Source:MGI Symbol;Acc:MGI:1924621]	Sec24a	protein_coding
ENSMUSG00000036430	231.2732871	-0.690567661	0.228159851	0.000145658	0.007515362	Down	72726	tubulin-specific chaperone C [Source:MGI Symbol;Acc:MGI:1919976]	Tbcc	protein_coding
ENSMUSG00000036442	444.2446353	-0.266833197	0.111000926	0.003631127	0.043606416	Down	59016	THAP domain containing 11 [Source:MGI Symbol;Acc:MGI:1930964]	Thap11	protein_coding
ENSMUSG00000036545	3772.473149	-0.392840776	0.170351639	0.002294346	0.033880863	Down	216725	a disintegrin-like and metallopeptidase (reprolysin type) with thrombospondin type 1 motif, 2 [Source:MGI Symbol;Acc:MGI:1347356]	Adamts2	protein_coding
ENSMUSG00000036564	180.4409144	-0.42240195	0.205630913	0.003534796	0.043004611	Down	234593	N-myc downstream regulated gene 4 [Source:MGI Symbol;Acc:MGI:2384590]	Ndrg4	protein_coding
ENSMUSG00000036672	154.4695919	-0.412708704	0.165522219	0.001395575	0.02591718	Down	320394	centromere protein T [Source:MGI Symbol;Acc:MGI:2443939]	Cenpt	protein_coding
ENSMUSG00000036737	1412.04968	0.224385278	0.075784676	0.0010686	0.022737013	Up	108737	oxidative-stress responsive 1 [Source:MGI Symbol;Acc:MGI:1917378]	Oxsr1	protein_coding
ENSMUSG00000036748	755.991318	-0.387851985	0.149614655	0.001066592	0.022737013	Down	67116	CUE domain containing 2 [Source:MGI Symbol;Acc:MGI:1914366]	Cuedc2	protein_coding
ENSMUSG00000036752	3450.334681	-0.352821778	0.130060593	0.000919042	0.020957408	Down	227613	tubulin, beta 4B class IVB [Source:MGI Symbol;Acc:MGI:1915472]	Tubb4b	protein_coding
ENSMUSG00000036779	636.0945181	0.252582324	0.088905513	0.001261806	0.024669378	Up	214627	terminal nucleotidyltransferase 4B [Source:MGI Symbol;Acc:MGI:1917820]	Tent4b	protein_coding
ENSMUSG00000036856	36.25213879	-0.163129817	0.321994342	0.000180187	0.008185824	Down	22417	wingless-type MMTV integration site family, member 4 [Source:MGI Symbol;Acc:MGI:98957]	Wnt4	protein_coding
ENSMUSG00000036879	1650.060506	0.282970781	0.087070745	0.000273238	0.010102419	Up	102093	phosphorylase kinase beta [Source:MGI Symbol;Acc:MGI:97578]	Phkb	protein_coding
ENSMUSG00000036887	2770.927203	-0.959566492	0.345363991	0.000218065	0.009173591	Down	12259	complement component 1, q subcomponent, alpha polypeptide [Source:MGI Symbol;Acc:MGI:88223]	C1qa	protein_coding
ENSMUSG00000036916	267.9435269	0.659979254	0.182123595	1.94E-05	0.002566184	Up	208968	zinc finger protein 280C [Source:MGI Symbol;Acc:MGI:2387585]	Zfp280c	protein_coding
ENSMUSG00000036966	507.9586778	-0.306385469	0.121808996	0.002299944	0.033934017	Down	223918	SPRY domain containing 3 [Source:MGI Symbol;Acc:MGI:2446175]	Spryd3	protein_coding
ENSMUSG00000036990	1248.400018	0.246296601	0.090502021	0.001882007	0.030289224	Up	73945	OTU domain containing 4 [Source:MGI Symbol;Acc:MGI:1098801]	Otud4	protein_coding

ENSMUSG00000037012	735.161739	-0.462510495	0.169108734	0.000497772	0.014738999	Down	15275	hexokinase 1 [Source:MGI Symbol;Acc:MGI:96103]	Hk1	protein_coding
ENSMUSG00000037032	256.0199758	-0.437534487	0.175279531	0.001231988	0.024468519	Down	11785	amyloid beta (A4) precursor protein-binding, family B, member 1 [Source:MGI Symbol;Acc:MGI:107765]	Apbb1	protein_coding
ENSMUSG00000037035	1337.095993	-0.478838789	0.229331748	0.003027626	0.0388775	Down	16324	inhibin beta-B [Source:MGI Symbol;Acc:MGI:96571]	Inhbb	protein_coding
ENSMUSG00000037060	5505.547835	-0.649340964	0.228441135	0.000262978	0.009995247	Down	109042	caveolae associated 3 [Source:MGI Symbol;Acc:MGI:1923422]	Cavin3	protein_coding
ENSMUSG00000037062	9555.11216	0.327362258	0.118553848	0.000986077	0.021758091	Up	54673	SH3-domain GRB2-like B1 (endophilin) [Source:MGI Symbol;Acc:MGI:1859730]	Sh3glb1	protein_coding
ENSMUSG00000037086	493.0900754	-0.700815557	0.259503117	0.000365684	0.012142269	Down	68800	proline rich 32 [Source:MGI Symbol;Acc:MGI:1916050]	Prr32	protein_coding
ENSMUSG00000037139	130.9680864	-0.62061201	0.291646327	0.001692667	0.028766989	Down	242702	myomesin family, member 3 [Source:MGI Symbol;Acc:MGI:2685280]	Myom3	protein_coding
ENSMUSG00000037151	157.522512	-0.425705465	0.177472068	0.001505709	0.026977079	Down	216011	leucine rich repeat containing 20 [Source:MGI Symbol;Acc:MGI:2387182]	Lrrc20	protein_coding
ENSMUSG00000037185	168.4667823	-0.825953245	0.247024731	4.30E-05	0.003960507	Down	74127	keratin 80 [Source:MGI Symbol;Acc:MGI:1921377]	Krt80	protein_coding
ENSMUSG00000037190	191.8119512	-0.595944621	0.21551919	0.00038125	0.012610537	Down	56368	cytochrome b-561 domain containing 2 [Source:MGI Symbol;Acc:MGI:1929280]	Cyb561d2	protein_coding
ENSMUSG00000037196	13.54852479	-0.158238569	0.305318783	0.00063313	0.017182247	Down	69310	PARK2 co-regulated [Source:MGI Symbol;Acc:MGI:1916560]	Pacrg	protein_coding
ENSMUSG00000037343	676.8081827	0.243775291	0.085879175	0.001370095	0.025832433	Up	319944	TATA-box binding protein associated factor 2 [Source:MGI Symbol;Acc:MGI:2443028]	Taf2	protein_coding
ENSMUSG00000037348	774.8621538	-0.830500243	0.26163976	7.37E-05	0.005101093	Down	71904	progestin and adipoQ receptor family member VII [Source:MGI Symbol;Acc:MGI:1919154]	Paqr7	protein_coding
ENSMUSG00000037440	211.4466439	1.02601542	0.347675099	0.000132746	0.007086499	Up	22361	vanin 1 [Source:MGI Symbol;Acc:MGI:108395]	Vnn1	protein_coding
ENSMUSG00000037446	6.351278212	-0.247286897	0.798669831	0.001908195	0.030589699	Down	22157	tubby like protein 1 [Source:MGI Symbol;Acc:MGI:109571]	Tulp1	protein_coding
ENSMUSG00000037475	1128.900014	0.346883232	0.161968234	0.004359736	0.047982906	Up	331401	THO complex 2 [Source:MGI Symbol;Acc:MGI:2442413]	Thoc2	protein_coding
ENSMUSG00000037499	1208.186783	-0.459607028	0.178422794	0.000902645	0.02074065	Down	66208	neuron derived neurotrophic factor [Source:MGI Symbol;Acc:MGI:1913458]	Nenf	protein_coding
ENSMUSG00000037523	1078.973065	0.265194422	0.090203852	0.000855582	0.020060187	Up	228607	mitochondrial antiviral signaling protein [Source:MGI Symbol;Acc:MGI:2444773]	Mavs	protein_coding
ENSMUSG00000037541	65.13102593	-0.932148648	0.674960259	0.003670492	0.043987202	Down	210274	SH3 and multiple ankyrin repeat domains 2 [Source:MGI Symbol;Acc:MGI:2671987]	Shank2	protein_coding
ENSMUSG00000037548	96.58235497	-1.31277447	0.41359717	5.80E-05	0.004545525	Down	15000	histocompatibility 2, class II, locus Mb2 [Source:MGI Symbol;Acc:MGI:95923]	H2-DMb2	protein_coding
ENSMUSG00000037563	3854.857325	-0.710722664	0.203146659	2.87E-05	0.003106886	Down	20055	ribosomal protein S16 [Source:MGI Symbol;Acc:MGI:98118]	Rps16	protein_coding
ENSMUSG00000037579	30.19511851	-0.962944669	0.53360605	0.002075181	0.032275804	Down	16512	potassium voltage-gated channel, subfamily H (eag-related), member 3 [Source:MGI Symbol;Acc:MGI:1341723]	Kcnh3	protein_coding
ENSMUSG00000037594	50.33780941	-1.112177786	0.377363972	0.000131741	0.007086499	Down	217887	clathrin binding box of aftiphilin containing 1 [Source:MGI Symbol;Acc:MGI:2443738]	Clba1	protein_coding
ENSMUSG00000037643	1840.400087	0.336787083	0.1138646	0.000547145	0.015688767	Up	18759	protein kinase C, iota [Source:MGI Symbol;Acc:MGI:99260]	Prkci	protein_coding
ENSMUSG00000037683	8.979936136	-0.083324565	0.199366588	0.001386244	0.02591718	Down	70882	armadillo repeat containing 3 [Source:MGI Symbol;Acc:MGI:1918132]	Armc3	protein_coding
ENSMUSG00000037703	185.8754949	-0.952419148	0.401760628	0.000657412	0.017631495	Down	241638	leucine zipper, putative tumor suppressor family member 3 [Source:MGI Symbol;Acc:MGI:2656976]	Lzts3	protein_coding
ENSMUSG00000037706	5859.530707	-0.291109015	0.121186607	0.002906831	0.038239246	Down	12520	CD81 antigen [Source:MGI Symbol;Acc:MGI:1096398]	Cd81	protein_coding
ENSMUSG00000037738	21.65893754	-1.478920355	0.75600349	0.001241455	0.024468519	Down	330721	NIMA (never in mitosis gene a)-related expressed kinase 5 [Source:MGI Symbol;Acc:MGI:2142824]	Nek5	protein_coding
ENSMUSG00000037740	537.0706156	-0.520520199	0.270396021	0.00318107	0.040219652	Down	99045	mitochondrial ribosomal protein S26 [Source:MGI Symbol;Acc:MGI:1333830]	Mrps26	protein_coding
ENSMUSG00000037772	464.1339236	-0.343244634	0.152541281	0.00336506	0.041809713	Down	19935	mitochondrial ribosomal protein L23 [Source:MGI Symbol;Acc:MGI:1196612]	Mrpl23	protein_coding
ENSMUSG00000037805	3661.622908	-0.381161047	0.182174952	0.003893416	0.045365278	Down	19896	ribosomal protein L10A [Source:MGI Symbol;Acc:MGI:1343877]	Rpl10a	protein_coding
ENSMUSG00000037813	51.82802611	-0.087236755	0.202403544	0.001873346	0.030289224	Down	228846	RIKEN cDNA D630003M21 gene [Source:MGI Symbol;Acc:MGI:3606579]	D630003M21Rik	protein_coding
ENSMUSG00000037820	4435.983148	-0.290742495	0.125812581	0.004562197	0.049137713	Down	21817	transglutaminase 2, C polypeptide [Source:MGI Symbol;Acc:MGI:98731]	Tgm2	protein_coding
ENSMUSG00000037894	981.3215617	-0.505639724	0.174845507	0.000324193	0.011138189	Down	51788	H2A.Z variant histone 1 [Source:MGI Symbol;Acc:MGI:1888388]	H2az1	protein_coding
ENSMUSG00000037904	67.34643523	-0.506049916	0.277434346	0.004064943	0.046324211	Down	74251	ankyrin repeat domain 9 [Source:MGI Symbol;Acc:MGI:1921501]	Ankrd9	protein_coding
ENSMUSG00000037936	1942.816902	0.460364029	0.171649163	0.000672018	0.017734889	Up	20778	scavenger receptor class B, member 1 [Source:MGI Symbol;Acc:MGI:893578]	Scarb1	protein_coding
ENSMUSG00000037960	415.1552468	-0.414422013	0.185542736	0.002655134	0.036638703	Down	68480	caspase recruitment domain family, member 19 [Source:MGI Symbol;Acc:MGI:1915730]	Card19	protein_coding
ENSMUSG00000037966	1362.248053	-0.55938925	0.180660158	0.000145106	0.007509348	Down	18081	ninjurin 1 [Source:MGI Symbol;Acc:MGI:1196617]	Ninj1	protein_coding
ENSMUSG00000037989	86.47220736	-0.071333888	0.190288598	0.004067111	0.046324211	Down	75607	WNK lysine deficient protein kinase 2 [Source:MGI Symbol;Acc:MGI:1922857]	Wnk2	protein_coding
ENSMUSG00000037992	415.7308346	-0.44004642	0.2097823	0.002966429	0.038516848	Down	19401	retinoic acid receptor, alpha [Source:MGI Symbol;Acc:MGI:97856]	Rara	protein_coding
ENSMUSG00000038057	11.17454159	-0.118189092	0.236563185	0.00034029	0.011566513	Down	13168	diazepam binding inhibitor-like 5 [Source:MGI Symbol;Acc:MGI:108039]	Dbil5	protein_coding
ENSMUSG00000038084	2076.577907	0.401071606	0.105836855	1.96E-05	0.002566184	Up	74143	OPA1, mitochondrial dynamin like GTPase [Source:MGI Symbol;Acc:MGI:1921393]	Opa1	protein_coding
ENSMUSG00000038095	1324.053392	0.279477183	0.100516097	0.001236414	0.024468519	Up	243272	strawberry notch 1 [Source:MGI Symbol;Acc:MGI:2384298]	Sbno1	protein_coding
ENSMUSG00000038102	1037.925337	0.208006348	0.081299671	0.003841453	0.045125939	Up	320714	trafficking protein particle complex 11 [Source:MGI Symbol;Acc:MGI:2444585]	Trappc11	protein_coding
ENSMUSG00000038121	937.6241462	0.328945709	0.108733514	0.000445779	0.013942115	Up	108654	family with sequence similarity 210, member A [Source:MGI Symbol;Acc:MGI:1914000]	Fam210a	protein_coding
ENSMUSG00000038127	3479.056786	0.284682201	0.088347511	0.000307884	0.010774635	Up	67501	coiled-coil domain containing 50 [Source:MGI Symbol;Acc:MGI:1914751]	Ccdc50	protein_coding
ENSMUSG00000038174	1240.941375	0.386542743	0.184416578	0.003981073	0.045835427	Up	213056	family with sequence similarity 126, member B [Source:MGI Symbol;Acc:MGI:1098784]	Fam126b	protein_coding
ENSMUSG00000038208	71.5878405	-0.611820773	0.261115934	0.001070956	0.022756833	Down	320655	post-GPI attachment to proteins 3 [Source:MGI Symbol;Acc:MGI:2444461]	Pgap3	protein_coding
ENSMUSG00000038250	527.0302687	0.482206598	0.121649835	7.25E-06	0.001469406	Up	74841	ubiquitin specific peptidase 38 [Source:MGI Symbol;Acc:MGI:1922091]	Usp38	protein_coding
ENSMUSG00000038267	624.1214002	1.263931702	0.279557376	3.09E-07	0.000241407	Up	73102	solute carrier family 22, member 23 [Source:MGI Symbol;Acc:MGI:1920352]	Slc22a23	protein_coding
ENSMUSG00000038312	688.2049652	-0.257166406	0.105082005	0.003682465	0.044069384	Down	108687	ER degradation enhancer, mannosidase alpha-like 2 [Source:MGI Symbol;Acc:MGI:1915540]	Edem2	protein_coding
ENSMUSG00000038324	2713.863573	0.225425567	0.076799328	0.001175755	0.024035335	Up	56407	transient receptor potential cation channel, subfamily C, member 4 associated protein [Source:MGI Symbol;Acc:MGI:1930751]	Trpc4ap	protein_coding
ENSMUSG00000038344	1357.665339	0.807698812	0.201792654	3.74E-06	0.000946988	Up	353170	taxilin gamma [Source:MGI Symbol;Acc:MGI:3590652]	Txlng	protein_coding
ENSMUSG00000038370	55.64933271	-0.080700231	0.196727541	0.003954549	0.045753568	Down	66425	Purkinje cell protein 4-like 1 [Source:MGI Symbol;Acc:MGI:1913675]	Pcp4l1	protein_coding
ENSMUSG00000038387	2911.336472	-0.539890619	0.121072749	6.23E-07	0.000383541	Down	20130	related RAS viral (r-ras) oncogene [Source:MGI Symbol;Acc:MGI:98179]	Rras	protein_coding

ENSMUSG00000038451	167.462184	-0.760268327	0.234607804	6.51E-05	0.004874724	Down	14794	splA/ryanodine receptor domain and SOCS box containing 2 [Source:MGI Symbol;Acc:MGI:1315199]	Spsb2	protein_coding
ENSMUSG00000038453	22.31047115	-0.142445528	0.271624857	0.001100845	0.02321297	Down	56013	SRC kinase signaling inhibitor 1 [Source:MGI Symbol;Acc:MGI:1933179]	Srcin1	protein_coding
ENSMUSG00000038467	2154.295854	-0.329341223	0.142599596	0.003040673	0.038930238	Down	75608	charged multivesicular body protein 4B [Source:MGI Symbol;Acc:MGI:1922858]	Chmp4b	protein_coding
ENSMUSG00000038515	310.5717535	-0.436919279	0.170526881	0.001006674	0.022038737	Down	66790	GH regulated TBC protein 1 [Source:MGI Symbol;Acc:MGI:1914040]	Grtp1	protein_coding
ENSMUSG00000038523	8.66188002	-0.14386965	0.277410394	0.000314923	0.010941659	Down	75480	RIKEN cDNA 1700003F12 gene [Source:MGI Symbol;Acc:MGI:1922730]	1700003F12Rik	protein_coding
ENSMUSG00000038535	360.5077612	0.472539582	0.152671518	0.000188195	0.00835522	Up	235469	zinc finger protein 280D [Source:MGI Symbol;Acc:MGI:2384583]	Zfp280d	protein_coding
ENSMUSG00000038539	2597.448595	-0.397953183	0.196665173	0.004153954	0.046948364	Down	107503	activating transcription factor 5 [Source:MGI Symbol;Acc:MGI:2141857]	Atf5	protein_coding
ENSMUSG00000038552	54.13094955	-0.527338955	0.284776219	0.003553888	0.043008531	Down	64339	fibronectin type III domain containing 4 [Source:MGI Symbol;Acc:MGI:1917195]	Fndc4	protein_coding
ENSMUSG00000038594	512.1209357	0.615280504	0.158772385	8.55E-06	0.001571476	Up	100038725	centrosomal protein 85-like [Source:MGI Symbol;Acc:MGI:3642684]	Cep85l	protein_coding
ENSMUSG00000038618	56.44858207	-0.75353259	0.329128043	0.000964263	0.021479733	Down	66985	Ras association (RalGDS/AF-6) domain family (N-terminal) member 7 [Source:MGI Symbol;Acc:MGI:1914235]	Rassf7	protein_coding
ENSMUSG00000038658	1000.162929	0.350637847	0.108186364	0.000198259	0.008556482	Up	226089	RAB6A GEF complex partner 1 [Source:MGI Symbol;Acc:MGI:1924893]	Ric1	protein_coding
ENSMUSG00000038677	46.29168464	-0.129705554	0.248559693	0.003052305	0.038967523	Down	268935	signal peptide, CUB domain, EGF-like 3 [Source:MGI Symbol;Acc:MGI:3045253]	Scube3	protein_coding
ENSMUSG00000038732	45.98482081	-0.819368126	0.546837377	0.003586236	0.043278431	Down	218121	membrane bound O-acyltransferase domain containing 1 [Source:MGI Symbol;Acc:MGI:2387184]	Mboat1	protein_coding
ENSMUSG00000038775	116.857328	-0.76819786	0.245220337	9.35E-05	0.005856894	Down	22351	villin-like [Source:MGI Symbol;Acc:MGI:1201781]	Vill	protein_coding
ENSMUSG00000038776	1594.69602	-0.645825281	0.191563809	5.41E-05	0.004435779	Down	13849	epoxide hydrolase 1, microsomal [Source:MGI Symbol;Acc:MGI:95405]	Ephx1	protein_coding
ENSMUSG00000038811	99.30883244	-0.784748908	0.466663504	0.002913931	0.038277303	Down	14710	guanine nucleotide binding protein (G protein), gamma transducing activity polypeptide 2 [Source:MGI Symbol;Acc:MGI:893584]	Gngt2	protein_coding
ENSMUSG00000038860	267.8944037	0.303369017	0.131114736	0.003733208	0.044596084	Up	99326	GTPase activating RANGAP domain-like 3 [Source:MGI Symbol;Acc:MGI:2139309]	Garnl3	protein_coding
ENSMUSG00000038932	5.842490652	-0.07504446	0.193652651	0.000620169	0.016991045	Down	277353	transcription factor-like 5 (basic helix-loop-helix) [Source:MGI Symbol;Acc:MGI:2672878]	Tcfl5	protein_coding
ENSMUSG00000038949	995.8446847	0.426354632	0.18494164	0.00200032	0.03146333	Up	226744	consortin, connexin sorting protein [Source:MGI Symbol;Acc:MGI:2445141]	Cnst	protein_coding
ENSMUSG00000038987	26.57149954	-1.045682541	0.603150535	0.002204607	0.03336142	Down	227736	cilia and flagella associated protein 157 [Source:MGI Symbol;Acc:MGI:2447809]	Cfap157	protein_coding
ENSMUSG00000039021	27.32694838	-0.076808254	0.194101081	0.003108962	0.039495505	Down	338348	tetratricopeptide repeat domain 16 [Source:MGI Symbol;Acc:MGI:2443048]	Ttc16	protein_coding
ENSMUSG00000039037	163.391534	1.180224305	0.597754763	0.001357802	0.025674417	Up	26938	ST6 (alpha-N-acetyl-neuraminyl-2,3-beta-galactosyl-1,3)-N-acetylgalactosaminide alpha-2,6-sialyltransferase 5 [Source:MGI Symbol;Acc:MGI:1349471]	St6galnac5	protein_coding
ENSMUSG00000039087	1457.784338	0.465420953	0.213266846	0.002332067	0.034202983	Up	68750	ras responsive element binding protein 1 [Source:MGI Symbol;Acc:MGI:2443664]	Rreb1	protein_coding
ENSMUSG00000039100	2413.715881	0.277821449	0.112837451	0.00299775	0.038725807	Up	223455	membrane associated ring-CH-type finger 6 [Source:MGI Symbol;Acc:MGI:2442773]	Marchf6	protein_coding
ENSMUSG00000039110	22.38514259	-0.080407474	0.197237482	0.001182251	0.024075597	Down	104601	MYCBP associated protein [Source:MGI Symbol;Acc:MGI:2388726]	Mycbpap	protein_coding
ENSMUSG00000039115	359.7445079	-0.395142998	0.191263356	0.003886357	0.045354164	Down	104099	integrin alpha 9 [Source:MGI Symbol;Acc:MGI:104756]	Itga9	protein_coding
ENSMUSG00000039148	902.811941	-0.439534315	0.233715966	0.004377586	0.04814227	Down	20227	squamous cell carcinoma antigen recognized by T cells 1 [Source:MGI Symbol;Acc:MGI:1309453]	Sart1	protein_coding
ENSMUSG00000039166	955.4949102	0.483790405	0.178616345	0.000569459	0.016114087	Up	432442	A kinase (PRKA) anchor protein 7 [Source:MGI Symbol;Acc:MGI:1859150]	Akap7	protein_coding
ENSMUSG00000039167	989.5500076	0.595867073	0.224135479	0.000492977	0.014661533	Up	170757	adhesion G protein-coupled receptor L4 [Source:MGI Symbol;Acc:MGI:2655562]	Adgrl4	protein_coding
ENSMUSG00000039221	530.8734594	-0.802331727	0.34918797	0.000984486	0.021758091	Down	68028	ribosomal protein L22 like 1 [Source:MGI Symbol;Acc:MGI:1915278]	Rpl22l1	protein_coding
ENSMUSG00000039285	1000.711023	0.238339135	0.089851546	0.002470546	0.03526538	Up	27215	5-azacytidine induced gene 2 [Source:MGI Symbol;Acc:MGI:1351332]	Azi2	protein_coding
ENSMUSG00000039361	4728.740266	0.42731922	0.104453731	6.88E-06	0.001444279	Up	233489	phosphatidylinositol binding clathrin assembly protein [Source:MGI Symbol;Acc:MGI:2385902]	Picalm	protein_coding
ENSMUSG00000039476	47.34557432	-0.756068839	0.379166984	0.001837502	0.030071858	Down	20204	paired related homeobox 2 [Source:MGI Symbol;Acc:MGI:98218]	Prrx2	protein_coding
ENSMUSG00000039492	20.35455324	-0.990367144	0.699254435	0.003498467	0.042788555	Down	381580	coiled-coil domain containing 27 [Source:MGI Symbol;Acc:MGI:2685881]	Ccdc27	protein_coding
ENSMUSG00000039509	436.7530095	0.215927387	0.080502079	0.002450599	0.035134082	Up	234865	nucleoporin 133 [Source:MGI Symbol;Acc:MGI:2442620]	Nup133	protein_coding
ENSMUSG00000039577	36.50409799	-0.96042695	0.448299111	0.001115128	0.023364734	Down	260305	nephronophthisis 4 (juvenile) homolog (human) [Source:MGI Symbol;Acc:MGI:2384210]	Nphp4	protein_coding
ENSMUSG00000039629	308.7295448	0.445420458	0.220795404	0.003518494	0.042911678	Up	320609	striatin interacting protein 2 [Source:MGI Symbol;Acc:MGI:2444363]	Strip2	protein_coding
ENSMUSG00000039704	726.6555673	0.383683451	0.132394467	0.000490231	0.014636612	Up	320506	LMBR1 domain containing 2 [Source:MGI Symbol;Acc:MGI:2444173]	Lmbrd2	protein_coding
ENSMUSG00000039747	103.8018537	-0.688414649	0.252783557	0.000355831	0.011906857	Down	269717	ORAI calcium release-activated calcium modulator 2 [Source:MGI Symbol;Acc:MGI:2443195]	Orai2	protein_coding
ENSMUSG00000039781	162.8095721	-0.582042959	0.22610972	0.000629601	0.01711734	Down	12009	centrosomal protein 131 [Source:MGI Symbol;Acc:MGI:107440]	Cep131	protein_coding
ENSMUSG00000039787	435.9126731	-0.452863129	0.186881629	0.001444631	0.026307269	Down	99151	cerebral endothelial cell adhesion molecule [Source:MGI Symbol;Acc:MGI:2139134]	Cercam	protein_coding
ENSMUSG00000039841	256.6182368	0.47662019	0.165006117	0.000351228	0.011844845	Up	627049	zinc finger protein 800 [Source:MGI Symbol;Acc:MGI:1889334]	Zfp800	protein_coding
ENSMUSG00000039929	448.9850274	0.49858522	0.185022514	0.000574575	0.016152764	Up	207932	URB1 ribosome biogenesis 1 homolog (S. cerevisiae) [Source:MGI Symbol;Acc:MGI:2146468]	Urb1	protein_coding
ENSMUSG00000039943	1113.395085	0.449607284	0.170138825	0.000805658	0.019582378	Up	18798	phospholipase C, beta 4 [Source:MGI Symbol;Acc:MGI:107464]	Plcb4	protein_coding
ENSMUSG00000039963	32.41394991	-0.060385992	0.183927687	0.003920241	0.045585372	Down	207607	coiled-coil domain containing 40 [Source:MGI Symbol;Acc:MGI:2443893]	Ccdc40	protein_coding
ENSMUSG00000039968	641.2474331	0.365551863	0.105828361	8.41E-05	0.005465984	Up	242860	round spermatid basic protein 1-like [Source:MGI Symbol;Acc:MGI:3036237]	Rsbn1l	protein_coding
ENSMUSG00000040021	1121.033086	0.32321546	0.099491576	0.000218254	0.009173591	Up	16798	large tumor suppressor [Source:MGI Symbol;Acc:MGI:1333883]	Lats1	protein_coding
ENSMUSG00000040026	112.794857	-0.138342444	0.264643225	0.001136904	0.023576735	Down	20210	serum amyloid A 3 [Source:MGI Symbol;Acc:MGI:98223]	Saa3	protein_coding
ENSMUSG00000040028	1061.871245	0.305123461	0.11312071	0.001427048	0.026134238	Up	15568	ELAV (embryonic lethal, abnormal vision)-like 1 (Hu antigen R) [Source:MGI Symbol;Acc:MGI:1100851]	Elavl1	protein_coding
ENSMUSG00000040029	1107.958527	0.240533145	0.091765369	0.002743077	0.037046589	Up	320727	importin 8 [Source:MGI Symbol;Acc:MGI:2444611]	Ipo8	protein_coding

ENSMUSG00000040037	781.0481251	0.458260372	0.168225383	0.000592948	0.016507716	Up	320840	neuronal growth regulator 1 [Source:MGI Symbol;Acc:MGI:2444846]	Negr1	protein_coding
ENSMUSG00000040044	891.0880343	0.453194545	0.146794391	0.000195328	0.008500208	Up	50793	origin recognition complex, subunit 3 [Source:MGI Symbol;Acc:MGI:1354944]	Orc3	protein_coding
ENSMUSG00000040105	767.9185174	0.385648422	0.133135135	0.000475452	0.014362153	Up	74411	phospholipid phosphatase 6 [Source:MGI Symbol;Acc:MGI:1921661]	Plpp6	protein_coding
ENSMUSG00000040123	909.8420539	0.295618236	0.119666438	0.002665221	0.036638703	Up	219105	zinc finger, MYM-type 5 [Source:MGI Symbol;Acc:MGI:3041170]	Zmym5	protein_coding
ENSMUSG00000040128	1500.925328	-0.46303596	0.121007278	1.36E-05	0.002132486	Down	108767	proline-rich nuclear receptor coactivator 1 [Source:MGI Symbol;Acc:MGI:1917838]	Pnrc1	protein_coding
ENSMUSG00000040147	1499.660788	0.432037028	0.152952171	0.000493454	0.014661533	Up	109731	monoamine oxidase B [Source:MGI Symbol;Acc:MGI:96916]	Maob	protein_coding
ENSMUSG00000040181	3892.912339	0.544534693	0.174111894	0.000146648	0.00753397	Up	14261	flavin containing monooxygenase 1 [Source:MGI Symbol;Acc:MGI:1310002]	Fmo1	protein_coding
ENSMUSG00000040212	773.751419	-0.452249167	0.186223308	0.001238259	0.024468519	Down	13732	epithelial membrane protein 3 [Source:MGI Symbol;Acc:MGI:1098729]	Emp3	protein_coding
ENSMUSG00000040220	163.7343943	-0.469653159	0.22063871	0.002496717	0.035419704	Down	104346	growth arrest specific 8 [Source:MGI Symbol;Acc:MGI:1202386]	Gas8	protein_coding
ENSMUSG00000040276	6.428693896	-0.057514321	0.182534777	0.002761609	0.037133201	Down	23969	protein kinase C and casein kinase substrate in neurons 1 [Source:MGI Symbol;Acc:MGI:1345181]	Pacsin1	protein_coding
ENSMUSG00000040283	1356.524374	0.733215432	0.310028181	0.000839136	0.019809354	Up	237754	butyrophilin-like 9 [Source:MGI Symbol;Acc:MGI:2442439]	Btnl9	protein_coding
ENSMUSG00000040350	62.42309425	-1.841354915	0.569986314	4.28E-05	0.003960507	Down	94089	tripartite motif-containing 7 [Source:MGI Symbol;Acc:MGI:2137353]	Trim7	protein_coding
ENSMUSG00000040396	564.6620634	0.315929422	0.065463352	2.72E-07	0.000234218	Up	68904	abhydrolase domain containing 13 [Source:MGI Symbol;Acc:MGI:1916154]	Abhd13	protein_coding
ENSMUSG00000040423	1195.983412	0.28492118	0.086928358	0.000228732	0.009224534	Up	381305	RING CCCH (C3H) domains 1 [Source:MGI Symbol;Acc:MGI:2685397]	Rc3h1	protein_coding
ENSMUSG00000040435	268.2212392	-0.371130572	0.177068405	0.004255117	0.047293134	Down	17872	protein phosphatase 1, regulatory subunit 15A [Source:MGI Symbol;Acc:MGI:1927072]	Ppp1r15a	protein_coding
ENSMUSG00000040447	515.1139133	-0.805671677	0.274032603	0.000162397	0.007749885	Down	216892	spinster homolog 2 [Source:MGI Symbol;Acc:MGI:2384936]	Spns2	protein_coding
ENSMUSG00000040455	448.4213341	0.42638972	0.125425798	7.93E-05	0.005295104	Up	77593	ubiquitin specific petidase 45 [Source:MGI Symbol;Acc:MGI:101850]	Usp45	protein_coding
ENSMUSG00000040462	3251.885799	-0.412590205	0.17356135	0.001372597	0.025851328	Down	216440	amplified in osteosarcoma [Source:MGI Symbol;Acc:MGI:1924301]	Os9	protein_coding
ENSMUSG00000040466	785.2894714	-0.391796292	0.192343108	0.004206282	0.04700834	Down	233016	biliverdin reductase B (flavin reductase (NADPH)) [Source:MGI Symbol;Acc:MGI:2385271]	Blvrb	protein_coding
ENSMUSG00000040616	65.13044753	-1.136685686	0.386963443	0.000132144	0.007086499	Down	214359	transmembrane protein 51 [Source:MGI Symbol;Acc:MGI:2384874]	Tmem51	protein_coding
ENSMUSG00000040651	689.7823575	0.35557792	0.147872748	0.002255039	0.033762886	Up	218850	transcription activation suppressor [Source:MGI Symbol;Acc:MGI:1921694]	Tasor	protein_coding
ENSMUSG00000040690	764.6674843	-1.188081082	0.351079483	3.00E-05	0.003210041	Down	107581	collagen, type XVI, alpha 1 [Source:MGI Symbol;Acc:MGI:1095396]	Col16a1	protein_coding
ENSMUSG00000040767	147.1230945	-0.819828524	0.304037032	0.000320892	0.011082018	Down	78372	small nuclear ribonucleoprotein 25 (U11/U12) [Source:MGI Symbol;Acc:MGI:1925622]	Snrnp25	protein_coding
ENSMUSG00000040774	1025.617758	0.343404514	0.147736596	0.003014986	0.038803026	Up	99712	choline/ethanolaminephosphotransferase 1 [Source:MGI Symbol;Acc:MGI:2139793]	Cept1	protein_coding
ENSMUSG00000040794	18.57238775	-0.167883823	0.31420564	0.004421726	0.048228949	Down	67445	C1q and tumor necrosis factor related protein 4 [Source:MGI Symbol;Acc:MGI:1914695]	C1qtnf4	protein_coding
ENSMUSG00000040812	18.22678135	-1.264554864	0.839927584	0.002703864	0.036812084	Down	271813	ATP/GTP binding protein-like 2 [Source:MGI Symbol;Acc:MGI:2443254]	Agbl2	protein_coding
ENSMUSG00000040818	687.1543566	0.267109748	0.105942956	0.002880759	0.038099856	Up	211922	DENN/MADD domain containing 6A [Source:MGI Symbol;Acc:MGI:2442980]	Dennd6a	protein_coding
ENSMUSG00000040824	617.9713991	-0.526875364	0.238453814	0.001751614	0.029221259	Down	107686	small nuclear ribonucleoprotein D2 [Source:MGI Symbol;Acc:MGI:98345]	Snrpd2	protein_coding
ENSMUSG00000040857	265.5067232	-0.505243088	0.270331901	0.003680131	0.044069384	Down	13875	Ets2 repressor factor [Source:MGI Symbol;Acc:MGI:109637]	Erf	protein_coding
ENSMUSG00000040860	190.2947197	-1.384995726	0.468074047	0.000109601	0.006430038	Down	230872	ciliary rootlet coiled-coil, rootletin [Source:MGI Symbol;Acc:MGI:3529431]	Crocc	protein_coding
ENSMUSG00000040883	2051.374075	-0.395471692	0.191248918	0.003817004	0.045022918	Down	235043	transmembrane protein 205 [Source:MGI Symbol;Acc:MGI:3045495]	Tmem205	protein_coding
ENSMUSG00000040907	678.5194751	0.863899842	0.3452765	0.000512377	0.01501664	Up	232975	ATPase, Na+/K+ transporting, alpha 3 polypeptide [Source:MGI Symbol;Acc:MGI:88107]	Atp1a3	protein_coding
ENSMUSG00000040936	57.24972776	-1.134873644	0.547461985	0.001126023	0.023450356	Down	209012	unc-51-like kinase 4 [Source:MGI Symbol;Acc:MGI:1921622]	Ulk4	protein_coding
ENSMUSG00000040938	67.55022519	-1.53960322	0.543819837	0.000154238	0.007637906	Down	216867	solute carrier family 16 (monocarboxylic acid transporters), member 11 [Source:MGI Symbol;Acc:MGI:2663709]	Slc16a11	protein_coding
ENSMUSG00000040945	1238.437364	-0.259349321	0.108512839	0.004075116	0.046354104	Down	108911	regulator of chromosome condensation 2 [Source:MGI Symbol;Acc:MGI:1919784]	Rcc2	protein_coding
ENSMUSG00000040964	328.5989824	-0.622144256	0.184745126	5.35E-05	0.004409981	Down	72754	Rho guanine nucleotide exchange factor (GEF) 10-like [Source:MGI Symbol;Acc:MGI:1920004]	Arhgef10l	protein_coding
ENSMUSG00000041046	16.0910662	-0.095224205	0.209214467	0.002180214	0.033161195	Down	56089	receptor (calcitonin) activity modifying protein 3 [Source:MGI Symbol;Acc:MGI:1860292]	Ramp3	protein_coding
ENSMUSG00000041078	50.12475136	-1.926316626	0.712125705	0.000212245	0.009008922	Down	14803	glutamate receptor, ionotropic, delta 1 [Source:MGI Symbol;Acc:MGI:95812]	Grid1	protein_coding
ENSMUSG00000041132	628.2759119	0.476327987	0.162065137	0.000302535	0.010741943	Up	100637	NEDD4 binding protein 2-like 1 [Source:MGI Symbol;Acc:MGI:2140872]	N4bp2l1	protein_coding
ENSMUSG00000041134	370.613101	0.905005153	0.286237705	7.72E-05	0.005239632	Up	224405	cysteine and tyrosine-rich protein 1 [Source:MGI Symbol;Acc:MGI:2152187]	Cyyr1	protein_coding
ENSMUSG00000041193	22.37498539	-0.080586573	0.197401698	0.001122429	0.023417446	Down	18784	phospholipase A2, group V [Source:MGI Symbol;Acc:MGI:101899]	Pla2g5	protein_coding
ENSMUSG00000041203	807.2199336	-0.577432815	0.254051195	0.001321338	0.025272596	Down	68544	telomerase RNA component interacting RNase [Source:MGI Symbol;Acc:MGI:1922833]	Trir	protein_coding
ENSMUSG00000041220	1864.592269	1.600182259	0.352140569	2.51E-07	0.000233309	Up	170439	ELOVL family member 6, elongation of long chain fatty acids (yeast) [Source:MGI Symbol;Acc:MGI:2156528]	Elovl6	protein_coding
ENSMUSG00000041231	510.2960333	0.242159612	0.100665811	0.004615844	0.049498962	Up	79560	ubiquitin-like domain containing CTD phosphatase 1 [Source:MGI Symbol;Acc:MGI:1933105]	Ublcp1	protein_coding
ENSMUSG00000041297	1097.785703	0.255023171	0.10728264	0.004544293	0.049102004	Up	69562	cyclin-dependent kinase 13 [Source:MGI Symbol;Acc:MGI:1916812]	Cdk13	protein_coding
ENSMUSG00000041319	182.5103439	-0.42345123	0.185400067	0.002066556	0.032199783	Down	386612	THO complex 6 [Source:MGI Symbol;Acc:MGI:2677480]	Thoc6	protein_coding
ENSMUSG00000041328	1057.488118	0.447746209	0.168016932	0.000730261	0.018561334	Up	74737	PCF11 cleavage and polyadenylation factor subunit [Source:MGI Symbol;Acc:MGI:1919579]	Pcf11	protein_coding
ENSMUSG00000041343	102.7716725	-0.714971225	0.271655393	0.000439399	0.013868433	Down	73845	ankyrin repeat domain 42 [Source:MGI Symbol;Acc:MGI:1921095]	Ankrd42	protein_coding
ENSMUSG00000041351	74.97834215	-0.197146343	0.408130693	0.002353854	0.034434609	Down	110351	Rap1 GTPase-activating protein [Source:MGI Symbol;Acc:MGI:109338]	Rap1gap	protein_coding
ENSMUSG00000041408	1700.7956	0.34768386	0.090233807	2.18E-05	0.002702701	Up	218914	WAPL cohesin release factor [Source:MGI Symbol;Acc:MGI:2675859]	Wapl	protein_coding
ENSMUSG00000041420	185.0359137	-0.405431652	0.180398184	0.002481205	0.035286651	Down	17537	Meis homeobox 3 [Source:MGI Symbol;Acc:MGI:108519]	Meis3	protein_coding
ENSMUSG00000041453	4966.918856	-0.392591038	0.184158703	0.00345168	0.042578956	Down	19933	ribosomal protein L21 [Source:MGI Symbol;Acc:MGI:1278340]	Rpl21	protein_coding

ENSMUSG00000041536	8.539216639	-0.126134257	0.244189298	0.002271468	0.033800769	Down	74069	serine (or cysteine) peptidase inhibitor, clade A, member 3A [Source:MGI Symbol;Acc:MGI:1921319]	Serpina3a	protein_coding
ENSMUSG00000041560	1491.073071	-0.422978626	0.163239148	0.000796472	0.019496604	Down	68077	NOP53 ribosome biogenesis factor [Source:MGI Symbol;Acc:MGI:2154441]	Nop53	protein_coding
ENSMUSG00000041571	4814.945197	-0.524640494	0.171553606	0.000185546	0.008348612	Down	20364	selenoprotein W [Source:MGI Symbol;Acc:MGI:1100878]	Selenow	protein_coding
ENSMUSG00000041609	526.4868743	-0.318082012	0.143737686	0.004290857	0.047461063	Down	75665	BICD family like cargo adaptor 1 [Source:MGI Symbol;Acc:MGI:1922915]	Bicdl1	protein_coding
ENSMUSG00000041633	899.2526057	0.818268691	0.186646583	7.44E-07	0.000413505	Up	207474	potassium channel tetramerisation domain containing 12b [Source:MGI Symbol;Acc:MGI:2444667]	Kctd12b	protein_coding
ENSMUSG00000041684	219.0969836	0.322124226	0.137094378	0.003045337	0.038931965	Up	246229	basic, immunoglobulin-like variable motif containing [Source:MGI Symbol;Acc:MGI:2179809]	Bivm	protein_coding
ENSMUSG00000041685	1589.121148	0.374932426	0.114215273	0.000161767	0.007749885	Up	218503	FCH domain only 2 [Source:MGI Symbol;Acc:MGI:3505790]	Fcho2	protein_coding
ENSMUSG00000041712	709.3102674	0.268259356	0.097617775	0.001420207	0.026099657	Up	66622	ubiquitin protein ligase E3 component n-recognin 7 (putative) [Source:MGI Symbol;Acc:MGI:1913872]	Ubr7	protein_coding
ENSMUSG00000041763	1194.689956	0.342354636	0.113383429	0.000387518	0.012696012	Up	22019	tripeptidyl peptidase II [Source:MGI Symbol;Acc:MGI:102724]	Tpp2	protein_coding
ENSMUSG00000041775	453.5225607	0.413078417	0.142496609	0.000437102	0.013846661	Up	69546	mitogen-activated protein kinase 1 interacting protein 1 [Source:MGI Symbol;Acc:MGI:1916796]	Mapk1ip1	protein_coding
ENSMUSG00000041846	770.1672258	0.407874349	0.135145735	0.000321547	0.011082444	Up	68734	protein phosphatase 4 regulatory subunit 3A [Source:MGI Symbol;Acc:MGI:1915984]	Ppp4r3a	protein_coding
ENSMUSG00000041879	2200.743229	0.271726455	0.091321718	0.000779445	0.019243812	Up	226432	importin 9 [Source:MGI Symbol;Acc:MGI:1918944]	Ipo9	protein_coding
ENSMUSG00000041935	2199.330655	0.441089436	0.152196529	0.000385665	0.012681007	Up	106064	RAB7A interacting MON1-CCZ1 complex subunit 1 [Source:MGI Symbol;Acc:MGI:2146232]	Rimoc1	protein_coding
ENSMUSG00000041997	1029.747344	0.343344101	0.10455533	0.000177919	0.008185484	Up	228012	tousled-like kinase 1 [Source:MGI Symbol;Acc:MGI:2441683]	Tlk1	protein_coding
ENSMUSG00000042010	5106.682042	0.848716366	0.401705172	0.00120136	0.024267616	Up	100705	acetyl-Coenzyme A carboxylase beta [Source:MGI Symbol;Acc:MGI:2140940]	Acacb	protein_coding
ENSMUSG00000042116	425.3078362	-0.469879461	0.157963321	0.000277948	0.01021295	Down	246228	von Willebrand factor A domain containing 1 [Source:MGI Symbol;Acc:MGI:2179729]	Vwa1	protein_coding
ENSMUSG00000042138	278.6416475	0.473493845	0.129555767	2.60E-05	0.002951933	Up	235184	Myb/SANT-like DNA-binding domain containing 2 [Source:MGI Symbol;Acc:MGI:2384579]	Msantd2	protein_coding
ENSMUSG00000042197	490.4999593	0.297703163	0.111337227	0.001521144	0.027108458	Up	98403	zinc finger protein 451 [Source:MGI Symbol;Acc:MGI:2137896]	Zfp451	protein_coding
ENSMUSG00000042216	30.85522998	-1.367793289	0.506350465	0.00023651	0.009412881	Down	52850	small G protein signaling modulator 1 [Source:MGI Symbol;Acc:MGI:107320]	Sgsm1	protein_coding
ENSMUSG00000042275	51.82895503	-0.68666269	0.255881723	0.000398136	0.012945423	Down	105083	pelota mRNA surveillance and ribosome rescue factor [Source:MGI Symbol;Acc:MGI:2145154]	Pelo	protein_coding
ENSMUSG00000042298	444.5364151	0.310791776	0.06753423	9.08E-07	0.000466086	Up	72795	tetratricopeptide repeat domain 19 [Source:MGI Symbol;Acc:MGI:1920045]	Ttc19	protein_coding
ENSMUSG00000042312	764.1764803	-0.630415311	0.293907449	0.001567344	0.027507171	Down	20196	S100 calcium binding protein A13 [Source:MGI Symbol;Acc:MGI:109581]	S100a13	protein_coding
ENSMUSG00000042380	301.4064893	-0.397789897	0.150849011	0.000960171	0.021461264	Down	80284	small integral membrane protein 12 [Source:MGI Symbol;Acc:MGI:1933141]	Smim12	protein_coding
ENSMUSG00000042410	950.1711492	0.260594176	0.086115536	0.00072367	0.018535693	Up	228061	alkylglycerone phosphate synthase [Source:MGI Symbol;Acc:MGI:2443065]	Agps	protein_coding
ENSMUSG00000042419	145.2544497	-0.517438132	0.191844059	0.000546675	0.015688767	Down	18038	nuclear factor of kappa light polypeptide gene enhancer in B cells inhibitor like 1 [Source:MGI Symbol;Acc:MGI:1340031]	Nfkbil1	protein_coding
ENSMUSG00000042428	98.9567726	-1.159514944	0.423169162	0.000227989	0.009224534	Down	17309	mannoside acetylglucosaminyltransferase 3 [Source:MGI Symbol;Acc:MGI:104532]	Mgat3	protein_coding
ENSMUSG00000042436	96.69009512	-1.568358394	0.521030661	9.08E-05	0.005793096	Down	76293	microfibrillar-associated protein 4 [Source:MGI Symbol;Acc:MGI:1342276]	Mfap4	protein_coding
ENSMUSG00000042485	428.3336451	-0.732261819	0.235643525	0.00010369	0.006269806	Down	66175	musculoskeletal, embryonic nuclear protein 1 [Source:MGI Symbol;Acc:MGI:1913425]	Mustn1	protein_coding
ENSMUSG00000042541	668.9301343	-0.537905556	0.262167444	0.002311505	0.034019775	Down	20422	SEM1, 26S proteasome complex subunit [Source:MGI Symbol;Acc:MGI:109238]	Sem1	protein_coding
ENSMUSG00000042557	797.1909478	0.277374622	0.108484074	0.002398038	0.03472722	Up	20466	transcriptional regulator, SIN3A (yeast) [Source:MGI Symbol;Acc:MGI:107157]	Sin3a	protein_coding
ENSMUSG00000042558	402.5616421	-0.403169444	0.121398828	0.000107203	0.006425415	Down	100206	ADP-ribosylserine hydrolase [Source:MGI Symbol;Acc:MGI:2140364]	Adprs	protein_coding
ENSMUSG00000042589	51.31397295	-1.395987085	0.706026894	0.001241426	0.024468519	Down	13048	cut-like homeobox 2 [Source:MGI Symbol;Acc:MGI:107321]	Cux2	protein_coding
ENSMUSG00000042595	212.5182504	0.359463606	0.114053637	0.000248217	0.009737966	Up	245622	family with sequence similarity 199, X-linked [Source:MGI Symbol;Acc:MGI:2384304]	Fam199x	protein_coding
ENSMUSG00000042684	104.2180853	-0.845931917	0.277309693	0.000113044	0.006537211	Down	74091	N-acetylneuraminate pyruvate lyase [Source:MGI Symbol;Acc:MGI:1921341]	Npl	protein_coding
ENSMUSG00000042734	76.7211484	-1.115271371	0.453448327	0.000513468	0.015023066	Down	69480	tetratricopeptide repeat domain 9 [Source:MGI Symbol;Acc:MGI:1916730]	Ttc9	protein_coding
ENSMUSG00000042750	6.233987045	-0.054448103	0.180877382	0.004555706	0.049112217	Down	12069	brain expressed X-linked 2 [Source:MGI Symbol;Acc:MGI:1338017]	Bex2	protein_coding
ENSMUSG00000042759	238.7533209	-0.770878799	0.291454539	0.000405843	0.013131231	Down	171504	apolipoprotein B receptor [Source:MGI Symbol;Acc:MGI:2176230]	Apobr	protein_coding
ENSMUSG00000042772	934.3292755	0.215702926	0.084965845	0.004014328	0.045941738	Up	226517	SMG7 nonsense mediated mRNA decay factor [Source:MGI Symbol;Acc:MGI:2682334]	Smg7	protein_coding
ENSMUSG00000043059	214.2788238	-0.343708042	0.163095338	0.004495024	0.048662455	Down	101023	zinc finger protein 513 [Source:MGI Symbol;Acc:MGI:2141255]	Zfp513	protein_coding
ENSMUSG00000043155	28.4830949	-0.822159477	0.41742721	0.001822505	0.029911646	Down	242642	4-hydroxyphenylpyruvate dioxygenase-like [Source:MGI Symbol;Acc:MGI:2444646]	Hpdl	protein_coding
ENSMUSG00000043207	1042.479699	0.387108417	0.121164055	0.000188428	0.00835522	Up	230709	zinc metallopeptidase, STE24 [Source:MGI Symbol;Acc:MGI:1890508]	Zmpste24	protein_coding
ENSMUSG00000043230	6.384605724	-0.1676511	0.319860594	0.002421452	0.034942197	Down	241128	family with sequence similarity 124, member B [Source:MGI Symbol;Acc:MGI:3026880]	Fam124b	protein_coding
ENSMUSG00000043439	9.572916252	-1.241436578	0.614438569	0.001526367	0.027173425	Down	103551	elongin BC and polycomb repressive complex 2 associated protein [Source:MGI Symbol;Acc:MGI:2143991]	Epop	protein_coding
ENSMUSG00000043448	41.87629077	-0.674131817	0.313151741	0.001499738	0.026974929	Down	118454	gap junction protein, gamma 2 [Source:MGI Symbol;Acc:MGI:2153060]	Gjc2	protein_coding
ENSMUSG00000043541	24.16517899	-0.111319135	0.225605512	0.002384295	0.034692551	Down	320662	cancer susceptibility candidate 1 [Source:MGI Symbol;Acc:MGI:2444480]	Casc1	protein_coding
ENSMUSG00000043614	18.0596587	-0.185418429	0.361469161	0.003548789	0.043007234	Down	194309	vacuolar protein sorting 37D [Source:MGI Symbol;Acc:MGI:2159402]	Vps37d	protein_coding
ENSMUSG00000043998	939.3343839	-0.31923776	0.101369619	0.000290454	0.010493486	Down	217664	mannoside acetylglucosaminyltransferase 2 [Source:MGI Symbol;Acc:MGI:2384966]	Mgat2	protein_coding
ENSMUSG00000044134	148.6505799	-0.499853011	0.181407784	0.000472526	0.014353791	Down	231717	PH domain containing endocytic trafficking adaptor 1 [Source:MGI Symbol;Acc:MGI:2442708]	Pheta1	protein_coding
ENSMUSG00000044148	615.861392	0.362848452	0.117393597	0.00030525	0.010757394	Up	69155	RIKEN cDNA 1810030O07 gene [Source:MGI Symbol;Acc:MGI:1916405]	1810030O07Rik	protein_coding
ENSMUSG00000044206	96.63825893	-1.116005698	0.727336172	0.002695346	0.036812084	Down	278180	V-set and immunoglobulin domain containing 4 [Source:MGI Symbol;Acc:MGI:2679720]	Vsig4	protein_coding

ENSMUSG00000044221	1672.018348	0.246733906	0.098364458	0.00326425	0.041018087	Up	231413	G-rich RNA sequence binding factor 1 [Source:MGI Symbol;Acc:MGI:106479]	Grsf1	protein_coding
ENSMUSG00000044244	84.45752683	-1.288942067	0.409776564	6.43E-05	0.004869134	Down	213208	interleukin 20 receptor beta [Source:MGI Symbol;Acc:MGI:2143266]	Il20rb	protein_coding
ENSMUSG00000044252	1353.836009	0.440633653	0.14710104	0.000293914	0.010552138	Up	64291	oxysterol binding protein-like 1A [Source:MGI Symbol;Acc:MGI:1927551]	Osbpl1a	protein_coding
ENSMUSG00000044303	21.20116454	-1.267573862	0.569267031	0.000828894	0.019736989	Down	12578	cyclin dependent kinase inhibitor 2A [Source:MGI Symbol;Acc:MGI:104738]	Cdkn2a	protein_coding
ENSMUSG00000044308	2832.661059	0.334785636	0.150309211	0.003774571	0.044767499	Up	68795	ubiquitin protein ligase E3 component n-recognin 3 [Source:MGI Symbol;Acc:MGI:1861100]	Ubr3	protein_coding
ENSMUSG00000044433	132.120758	-0.671893092	0.248046967	0.000406136	0.013131231	Down	69697	calmodulin regulated spectrin-associated protein family, member 3 [Source:MGI Symbol;Acc:MGI:1916947]	Camsap3	protein_coding
ENSMUSG00000044501	191.0976049	0.415566579	0.191586015	0.00288934	0.038184051	Up	224598	zinc finger protein 758 [Source:MGI Symbol;Acc:MGI:2385044]	Zfp758	protein_coding
ENSMUSG00000044550	23.55137424	-1.570718528	0.596442127	0.000270052	0.010102419	Down	594844	transcription elongation factor A (SII)-like 3 [Source:MGI Symbol;Acc:MGI:1913354]	Tceal3	protein_coding
ENSMUSG00000044709	628.1397296	-0.44178993	0.182944003	0.00141093	0.026088583	Down	69731	gem nuclear organelle associated protein 7 [Source:MGI Symbol;Acc:MGI:1916981]	Gemin7	protein_coding
ENSMUSG00000044724	15.14618255	1.181271056	0.735034679	0.00257277	0.036024921	Up	269053	G protein-coupled receptor 152 [Source:MGI Symbol;Acc:MGI:2685519]	Gpr152	protein_coding
ENSMUSG00000044854	75.63456179	-0.549312163	0.298603158	0.003462639	0.042634311	Down	73363	RIKEN cDNA 1700056E22 gene [Source:MGI Symbol;Acc:MGI:1920613]	1700056E22Rik	protein_coding
ENSMUSG00000044948	163.0319306	-1.03419486	0.428133339	0.000553899	0.015829765	Down	100048534	cilia and flagella associated protein 43 [Source:MGI Symbol;Acc:MGI:1289258]	Cfap43	protein_coding
ENSMUSG00000044952	365.1755972	0.485951264	0.223618032	0.002162455	0.03297839	Up	622320	potassium channel tetramerisation domain containing 21 [Source:MGI Symbol;Acc:MGI:3643121]	Kctd21	protein_coding
ENSMUSG00000044968	1038.430012	0.256401786	0.101596505	0.003196451	0.040384485	Up	242864	N-acyl phosphatidylethanolamine phospholipase D [Source:MGI Symbol;Acc:MGI:2140885]	Napepld	protein_coding
ENSMUSG00000045064	37.50509044	-0.799641787	0.428160284	0.002169249	0.033052757	Down	72350	zinc finger, C2HC-type containing 1C [Source:MGI Symbol;Acc:MGI:1919600]	Zc2hc1c	protein_coding
ENSMUSG00000045140	112.3993741	0.338793657	0.148985103	0.003320655	0.041437258	Up	70325	phosphatidylinositol glycan anchor biosynthesis, class W [Source:MGI Symbol;Acc:MGI:1917575]	Pigw	protein_coding
ENSMUSG00000045176	189.1292199	-0.605357394	0.265253011	0.001237378	0.024468519	Down	71923	BLOC-1 related complex subunit 6 [Source:MGI Symbol;Acc:MGI:1919173]	Borcs6	protein_coding
ENSMUSG00000045210	809.4302881	0.324599705	0.108195128	0.000458099	0.014147708	Up	70675	valosin containing protein (p97)/p47 complex interacting protein 1 [Source:MGI Symbol;Acc:MGI:1917925]	Vcpip1	protein_coding
ENSMUSG00000045275	55.455065	-1.001689725	0.498132781	0.001378207	0.025872156	Down	385668	Leber congenital amaurosis 5-like [Source:MGI Symbol;Acc:MGI:3041157]	Lca5l	protein_coding
ENSMUSG00000045467	25.39038837	-1.475897596	0.510973858	0.000142338	0.007455645	Down	269954	tubulin tyrosine ligase-like family, member 13 [Source:MGI Symbol;Acc:MGI:1920845]	Ttll13	protein_coding
ENSMUSG00000045502	418.6953434	0.987019945	0.305008969	5.58E-05	0.004471085	Up	80885	hydroxycarboxylic acid receptor 2 [Source:MGI Symbol;Acc:MGI:1933383]	Hcar2	protein_coding
ENSMUSG00000045555	128.2432384	-0.523774401	0.229939475	0.001520036	0.027108458	Down	327747	methyltransferase like 24 [Source:MGI Symbol;Acc:MGI:3045338]	Mettl24	protein_coding
ENSMUSG00000045636	2970.717238	0.744110051	0.156673081	1.45E-07	0.000178701	Up	102103	mitochondrial tumor suppressor 1 [Source:MGI Symbol;Acc:MGI:2142572]	Mtus1	protein_coding
ENSMUSG00000045659	171.0187094	-0.587897844	0.322826862	0.003256088	0.040971021	Down	233765	pleckstrin homology domain containing, family A member 7 [Source:MGI Symbol;Acc:MGI:2445094]	Plekha7	protein_coding
ENSMUSG00000045790	215.9965723	-0.335679096	0.142016628	0.002695207	0.036812084	Down	100503884	coiled-coil domain containing 149 [Source:MGI Symbol;Acc:MGI:2685293]	Ccdc149	protein_coding
ENSMUSG00000045802	56.97725727	0.674806586	0.322407302	0.001685258	0.028725282	Up	245525	heat shock transcription factor 3 [Source:MGI Symbol;Acc:MGI:3045337]	Hsf3	protein_coding
ENSMUSG00000045948	421.7099432	-0.606174441	0.237682309	0.000611097	0.016903753	Down	24030	mitochondrial ribosomal protein S12 [Source:MGI Symbol;Acc:MGI:1346333]	Mrps12	protein_coding
ENSMUSG00000046056	581.1638855	-1.409682601	0.280437382	2.92E-08	6.29E-05	Down	282619	suprabasin [Source:MGI Symbol;Acc:MGI:2446326]	Sbsn	protein_coding
ENSMUSG00000046062	1734.764271	0.564655569	0.129049874	9.89E-07	0.000478129	Up	108954	protein phosphatase 1, regulatory subunit 15B [Source:MGI Symbol;Acc:MGI:2444211]	Ppp1r15b	protein_coding
ENSMUSG00000046096	808.4401675	0.217492131	0.074594752	0.001297571	0.025124767	Up	233812	modulator of smoothened [Source:MGI Symbol;Acc:MGI:2446240]	Mosmo	protein_coding
ENSMUSG00000046157	240.8739524	-0.609074844	0.360958162	0.003804111	0.044993994	Down	268567	transmembrane protein 229B [Source:MGI Symbol;Acc:MGI:2444389]	Tmem229b	protein_coding
ENSMUSG00000046229	1057.024255	-0.648241874	0.357532103	0.00287375	0.03803636	Down	19018	SCAN domain-containing 1 [Source:MGI Symbol;Acc:MGI:1343132]	Scand1	protein_coding
ENSMUSG00000046432	80.15822827	-1.278355206	0.445327331	0.000148346	0.00753397	Down	12070	brain expressed X-linked 3 [Source:MGI Symbol;Acc:MGI:1338016]	Bex3	protein_coding
ENSMUSG00000046447	721.5452356	-0.496244747	0.208674897	0.001309988	0.025189625	Down	66259	calcium/calmodulin-dependent protein kinase II inhibitor 1 [Source:MGI Symbol;Acc:MGI:1913509]	Camk2n1	protein_coding
ENSMUSG00000046491	76.19886855	-0.626625666	0.263114023	0.000940499	0.021297795	Down	69183	C1q and tumor necrosis factor related protein 2 [Source:MGI Symbol;Acc:MGI:1916433]	C1qtnf2	protein_coding
ENSMUSG00000046532	1629.914828	0.428634654	0.196969913	0.002720159	0.036881584	Up	11835	androgen receptor [Source:MGI Symbol;Acc:MGI:88064]	Ar	protein_coding
ENSMUSG00000046546	517.9390814	-0.470662251	0.232279215	0.003040319	0.038930238	Down	224093	family with sequence similarity 43, member A [Source:MGI Symbol;Acc:MGI:2676309]	Fam43a	protein_coding
ENSMUSG00000046721	32.1520401	-0.962108002	0.357074224	0.000291122	0.010495604	Down	NA	ribosomal protein L14, pseudogene 1 [Source:MGI Symbol;Acc:MGI:3710579]	Rpl14-ps1	processed_pseudogene
ENSMUSG00000046722	1122.561034	-0.343560371	0.142623794	0.001969755	0.031227954	Down	57912	CDC42 small effector 1 [Source:MGI Symbol;Acc:MGI:1889510]	Cdc42se1	protein_coding
ENSMUSG00000046727	691.8893097	-0.636833353	0.281125325	0.001263222	0.024669378	Down	66060	cysteine-rich transmembrane module containing 1 [Source:MGI Symbol;Acc:MGI:1913310]	Cystm1	protein_coding
ENSMUSG00000046764	468.8749422	0.467645031	0.218533914	0.002616629	0.036337993	Up	208079	RIKEN cDNA A530053G22 gene [Source:MGI Symbol;Acc:MGI:2443347]	A530053G22Rik	lncRNA
ENSMUSG00000046785	1018.764476	0.410680874	0.186818558	0.002762419	0.037133201	Up	77781	EPM2A (laforin) interacting protein 1 [Source:MGI Symbol;Acc:MGI:1925031]	Epm2aip1	protein_coding
ENSMUSG00000046818	44.44662002	-1.869316127	0.697799599	0.000220132	0.009207591	Down	73284	DNA-damage-inducible transcript 4-like [Source:MGI Symbol;Acc:MGI:1920534]	Ddit4l	protein_coding
ENSMUSG00000046836	1057.705751	0.314020138	0.063753936	1.68E-07	0.000181415	Up	71678	BRO1 domain and CAAX motif containing [Source:MGI Symbol;Acc:MGI:1918928]	Brox	protein_coding
ENSMUSG00000046873	350.5178466	0.390057492	0.086856674	1.03E-06	0.000478129	Up	270669	membrane-bound transcription factor peptidase, site 2 [Source:MGI Symbol;Acc:MGI:2444506]	Mbtps2	protein_coding
ENSMUSG00000046985	1067.647024	0.528973866	0.171477401	0.000173925	0.008122649	Up	231225	transmembrane anterior posterior transformation 1 [Source:MGI Symbol;Acc:MGI:2683537]	Tapt1	protein_coding
ENSMUSG00000047022	96.86328236	0.452992792	0.164774853	0.00057971	0.016244133	Up	73490	mirror-image polydactyly 1 [Source:MGI Symbol;Acc:MGI:1920740]	Mipol1	protein_coding
ENSMUSG00000047126	7737.164751	0.349560233	0.099511322	7.32E-05	0.005101093	Up	67300	clathrin, heavy polypeptide (Hc) [Source:MGI Symbol;Acc:MGI:2388633]	Cltc	protein_coding
ENSMUSG00000047139	386.217116	-1.253750322	0.447487712	0.000180502	0.008185824	Down	12484	CD24a antigen [Source:MGI Symbol;Acc:MGI:88323]	Cd24a	protein_coding
ENSMUSG00000047141	343.0229629	0.455568267	0.181689285	0.001112545	0.023364734	Up	72020	zinc finger protein 654 [Source:MGI Symbol;Acc:MGI:1919270]	Zfp654	protein_coding

ENSMUSG00000047146	16.64887315	-0.997008602	0.583592776	0.002503902	0.035468996	Down	52463	tet methylcytosine dioxygenase 1 [Source:MGI Symbol;Acc:MGI:1098693]	Tet1	protein_coding
ENSMUSG00000047213	1576.99422	0.358403892	0.094778286	2.75E-05	0.003061902	Up	229096	YTH N6-methyladenosine RNA binding protein 3 [Source:MGI Symbol;Acc:MGI:1918850]	Ythdf3	protein_coding
ENSMUSG00000047238	145.2334292	-0.522152472	0.20850344	0.000840744	0.019820172	Down	75625	MAGE family member H1 [Source:MGI Symbol;Acc:MGI:1922875]	Mageh1	protein_coding
ENSMUSG00000047242	359.0072143	0.443075478	0.227773807	0.003827768	0.045022918	Up	407786	TATA-box binding protein associated factor 9B [Source:MGI Symbol;Acc:MGI:3039562]	Taf9b	protein_coding
ENSMUSG00000047361	17.2248052	-0.05858941	0.182984238	0.003932264	0.045632798	Down	381260	predicted gene 973 [Source:MGI Symbol;Acc:MGI:2685819]	Gm973	protein_coding
ENSMUSG00000047379	798.3928389	-0.355095035	0.140063421	0.001548664	0.027385104	Down	108902	beta-1,4-glucuronyltransferase 1 [Source:MGI Symbol;Acc:MGI:1919680]	B4gat1	protein_coding
ENSMUSG00000047409	2719.299002	0.364205159	0.159820612	0.002665184	0.036638703	Up	69274	CTD (carboxy-terminal domain, RNA polymerase II, polypeptide A) small phosphatase-like [Source:MGI Symbol;Acc:MGI:1916524]	Ctdspl	protein_coding
ENSMUSG00000047412	610.1161091	0.34501136	0.103440724	0.000134721	0.007165599	Up	235132	zinc finger and BTB domain containing 44 [Source:MGI Symbol;Acc:MGI:1925123]	Zbtb44	protein_coding
ENSMUSG00000047446	3619.120983	0.359864305	0.170843682	0.004651986	0.04967018	Up	11861	ADP-ribosylation factor-like 4A [Source:MGI Symbol;Acc:MGI:99437]	Arl4a	protein_coding
ENSMUSG00000047454	900.9122516	0.408924995	0.123073497	0.000108944	0.006430038	Up	268566	gephyrin [Source:MGI Symbol;Acc:MGI:109602]	Gphn	protein_coding
ENSMUSG00000047496	101.6697115	0.73717559	0.316559829	0.000902657	0.02074065	Up	320311	ring finger protein 152 [Source:MGI Symbol;Acc:MGI:2443787]	Rnf152	protein_coding
ENSMUSG00000047497	693.8713943	0.46930324	0.224681012	0.003042165	0.038930238	Up	239337	a disintegrin-like and metallopeptidase (reprolysin type) with thrombospondin type 1 motif, 12 [Source:MGI Symbol;Acc:MGI:2146046]	Adamts12	protein_coding
ENSMUSG00000047547	1329.468021	-0.498571356	0.232256067	0.002111441	0.032569253	Down	74325	clathrin, light polypeptide (Lcb) [Source:MGI Symbol;Acc:MGI:1921575]	Cltb	protein_coding
ENSMUSG00000047654	15.25911954	-1.45127103	0.687097565	0.000984909	0.021758091	Down	83984	testis-specific serine kinase 6 [Source:MGI Symbol;Acc:MGI:2148775]	Tssk6	protein_coding
ENSMUSG00000047671	23.33668169	-0.121295336	0.243151789	1.83E-05	0.002550282	Down	242646	dynein light chain Tctex-type 4 [Source:MGI Symbol;Acc:MGI:3045358]	Dynlt4	protein_coding
ENSMUSG00000047878	217.7233536	-0.383705852	0.16541104	0.002200516	0.033352241	Down	239559	alpha 1,4-galactosyltransferase [Source:MGI Symbol;Acc:MGI:3512453]	A4galt	protein_coding
ENSMUSG00000047879	1293.718298	0.339201824	0.152707676	0.003623375	0.043597946	Up	59025	ubiquitin specific peptidase 14 [Source:MGI Symbol;Acc:MGI:1928898]	Usp14	protein_coding
ENSMUSG00000047963	328.680957	-0.595151477	0.218522406	0.000410613	0.013251122	Down	52331	starch binding domain 1 [Source:MGI Symbol;Acc:MGI:1261768]	Stbd1	protein_coding
ENSMUSG00000048029	17.48988602	-0.068715506	0.189020863	0.001686879	0.028725282	Down	226265	enolase 4 [Source:MGI Symbol;Acc:MGI:2441717]	Eno4	protein_coding
ENSMUSG00000048058	292.3642639	0.341584647	0.160314083	0.004402973	0.048144948	Up	241576	low density lipoprotein receptor class A domain containing 3 [Source:MGI Symbol;Acc:MGI:2138856]	Ldlrad3	protein_coding
ENSMUSG00000048170	825.6634079	0.352438599	0.098377745	5.34E-05	0.004409981	Up	210711	minichromosome maintenance complex binding protein [Source:MGI Symbol;Acc:MGI:1920977]	Mcmbp	protein_coding
ENSMUSG00000048234	648.2116568	-0.4104884	0.149026374	0.000671975	0.017734889	Down	67702	ring finger protein 149 [Source:MGI Symbol;Acc:MGI:2677438]	Rnf149	protein_coding
ENSMUSG00000048371	403.5603284	0.54181588	0.16737223	9.25E-05	0.005837253	Up	382051	pyruvate dehydrogenase phosphatase catalytic subunit 2 [Source:MGI Symbol;Acc:MGI:1918878]	Pdp2	protein_coding
ENSMUSG00000048416	24.51659691	-0.113559265	0.228411227	0.002103168	0.03255949	Down	17349	myeloid leukemia factor 1 [Source:MGI Symbol;Acc:MGI:1341819]	Mlf1	protein_coding
ENSMUSG00000048490	3595.288206	0.617044514	0.219564558	0.000281609	0.010303527	Up	268903	nuclear receptor interacting protein 1 [Source:MGI Symbol;Acc:MGI:1315213]	Nrip1	protein_coding
ENSMUSG00000048644	41.32954282	-1.656595427	0.451600868	9.53E-06	0.001692953	Down	330695	cortexin 1 [Source:MGI Symbol;Acc:MGI:88566]	Ctxn1	protein_coding
ENSMUSG00000048647	209.5351222	0.400346502	0.195002779	0.003886431	0.045354164	Up	241624	exonuclease 3'-5' domain containing 1 [Source:MGI Symbol;Acc:MGI:3045306]	Exd1	protein_coding
ENSMUSG00000048799	803.0514233	0.361831706	0.143952073	0.00161982	0.028167874	Up	225523	centrosomal protein 120 [Source:MGI Symbol;Acc:MGI:2147298]	Cep120	protein_coding
ENSMUSG00000048915	497.661686	0.395311029	0.154410896	0.001230135	0.024468519	Up	13640	ephrin A5 [Source:MGI Symbol;Acc:MGI:107444]	Efna5	protein_coding
ENSMUSG00000049076	1564.630036	0.289537044	0.118025305	0.002937818	0.038383182	Up	78618	ArfGAP with coiled-coil, ankyrin repeat and PH domains 2 [Source:MGI Symbol;Acc:MGI:1925868]	Acap2	protein_coding
ENSMUSG00000049090	1357.072492	0.374779738	0.106692779	6.81E-05	0.004991657	Up	225791	zinc binding alcohol dehydrogenase, domain containing 2 [Source:MGI Symbol;Acc:MGI:2444835]	Zadh2	protein_coding
ENSMUSG00000049115	204.2186332	0.41014554	0.186226087	0.002728964	0.036914178	Up	11607	angiotensin II receptor, type 1a [Source:MGI Symbol;Acc:MGI:87964]	Agtr1a	protein_coding
ENSMUSG00000049134	8.454334786	-2.169887713	0.845901633	0.000311516	0.010845171	Down	18175	nebulin-related anchoring protein [Source:MGI Symbol;Acc:MGI:1098765]	Nrap	protein_coding
ENSMUSG00000049241	1859.904881	1.17152779	0.3570862	4.47E-05	0.004062525	Up	243270	hydrocarboxylic acid receptor 1 [Source:MGI Symbol;Acc:MGI:2441671]	Hcar1	protein_coding
ENSMUSG00000049353	6.738458267	-0.071957816	0.190617596	0.004168856	0.04696359	Down	74023	retinal degeneration 3 [Source:MGI Symbol;Acc:MGI:1921273]	Rd3	protein_coding
ENSMUSG00000049421	632.4554101	0.262127566	0.110429222	0.00438271	0.048144948	Up	26466	zinc finger protein 260 [Source:MGI Symbol;Acc:MGI:1347071]	Zfp260	protein_coding
ENSMUSG00000049502	870.3449862	0.461677933	0.161953879	0.000421249	0.013519063	Up	209200	deltex 3-like, E3 ubiquitin ligase [Source:MGI Symbol;Acc:MGI:2656973]	Dtx3l	protein_coding
ENSMUSG00000049517	3828.066161	-0.794393896	0.194448006	2.60E-06	0.000864464	Down	66475	ribosomal protein S23 [Source:MGI Symbol;Acc:MGI:1913725]	Rps23	protein_coding
ENSMUSG00000049571	36.28187398	-0.095687358	0.210193189	0.001202608	0.024267616	Down	212124	cilia and flagella associated protein 46 [Source:MGI Symbol;Acc:MGI:2444387]	Cfap46	protein_coding
ENSMUSG00000049760	806.9968416	-0.543894913	0.263770559	0.002221797	0.033556725	Down	224904	mitochondrial contact site and cristae organizing system subunit 13 [Source:MGI Symbol;Acc:MGI:2442174]	Micos13	protein_coding
ENSMUSG00000049866	297.7609897	-0.743085768	0.418461369	0.00267074	0.036638703	Down	320982	ADP-ribosylation factor-like 4C [Source:MGI Symbol;Acc:MGI:2445172]	Arl4c	protein_coding
ENSMUSG00000050029	817.0837103	0.237941358	0.080050622	0.000936359	0.021259924	Up	72065	RAP2C, member of RAS oncogene family [Source:MGI Symbol;Acc:MGI:1919315]	Rap2c	protein_coding
ENSMUSG00000050052	1757.45011	0.369982743	0.157489394	0.002370884	0.034595638	Up	72148	testis development related protein [Source:MGI Symbol;Acc:MGI:1919398]	Tdrp	protein_coding
ENSMUSG00000050103	999.334368	0.413712442	0.183993079	0.002393223	0.03472722	Up	319660	alkylglycerol monooxygenase [Source:MGI Symbol;Acc:MGI:2442495]	Agmo	protein_coding
ENSMUSG00000050122	12.22944738	-0.076988544	0.194144615	0.003350193	0.041715223	Down	70853	von Willebrand factor A domain containing 3B [Source:MGI Symbol;Acc:MGI:1918103]	Vwa3b	protein_coding
ENSMUSG00000050212	269.0336423	-0.724765879	0.259982425	0.0002644	0.010014062	Down	230752	eva-1 homolog B (C. elegans) [Source:MGI Symbol;Acc:MGI:1922063]	Eva1b	protein_coding
ENSMUSG00000050240	49.75836504	0.537440155	0.298204314	0.003861856	0.045255836	Up	58180	hypermethylated in cancer 2 [Source:MGI Symbol;Acc:MGI:1929869]	Hic2	protein_coding
ENSMUSG00000050248	503.601472	-0.459290913	0.131861631	4.48E-05	0.004062525	Down	68525	EvC ciliary complex subunit 2 [Source:MGI Symbol;Acc:MGI:1915775]	Evc2	protein_coding
ENSMUSG00000050299	75.75162559	-0.561155252	0.313059984	0.003497409	0.042788555	Down	NA	predicted gene 9843 [Source:MGI Symbol;Acc:MGI:3708621]	Gm9843	processed_pseudogene
ENSMUSG00000050312	326.73785	0.342821864	0.097299512	7.45E-05	0.005101815	Up	106338	NOL1/NOP2/Sun domain family member 3 [Source:MGI Symbol;Acc:MGI:2146565]	Nsun3	protein_coding

ENSMUSG00000050565	2252.081807	0.265929796	0.069193604	3.54E-05	0.003506698	Up	240832	torsin A interacting protein 2 [Source:MGI Symbol;Acc:MGI:3582695]	Tor1aip2	protein_coding
ENSMUSG00000050628	348.5332999	-0.702966992	0.247505092	0.000226366	0.009224534	Down	319370	UBA-like domain containing 2 [Source:MGI Symbol;Acc:MGI:1914635]	Ubald2	protein_coding
ENSMUSG00000050697	1546.098001	0.40527552	0.139903784	0.000469278	0.014338768	Up	105787	protein kinase, AMP-activated, alpha 1 catalytic subunit [Source:MGI Symbol;Acc:MGI:2145955]	Prkaa1	protein_coding
ENSMUSG00000050705	522.6224438	0.327199621	0.120637356	0.001129149	0.023453446	Up	69662	RIKEN cDNA 2310061I04 gene [Source:MGI Symbol;Acc:MGI:1916912]	2310061I04Rik	protein_coding
ENSMUSG00000050732	603.3392459	-0.537776179	0.269745834	0.00267248	0.036638703	Down	22320	vesicle-associated membrane protein 8 [Source:MGI Symbol;Acc:MGI:1336882]	Vamp8	protein_coding
ENSMUSG00000050777	261.0538698	-1.075680782	0.313589845	2.63E-05	0.002956124	Down	170706	transmembrane protein 37 [Source:MGI Symbol;Acc:MGI:2157899]	Tmem37	protein_coding
ENSMUSG00000050812	2642.8666	0.353825663	0.097387404	4.60E-05	0.004104998	Up	230249	Ecm29 proteasome adaptor and scaffold [Source:MGI Symbol;Acc:MGI:2140220]	Ecpas	protein_coding
ENSMUSG00000050821	81.51809637	-0.479123842	0.247849232	0.003543341	0.043004611	Down	78408	family with sequence similarity 131, member A [Source:MGI Symbol;Acc:MGI:1925658]	Fam131a	protein_coding
ENSMUSG00000050919	306.8731581	0.510239392	0.260449169	0.002990256	0.038681479	Up	238803	zinc finger protein 366 [Source:MGI Symbol;Acc:MGI:2178429]	Zfp366	protein_coding
ENSMUSG00000051652	246.324289	-0.430884075	0.207528858	0.003110273	0.039495505	Down	237387	leucine rich repeat containing 3 [Source:MGI Symbol;Acc:MGI:2447899]	Lrrc3	protein_coding
ENSMUSG00000051851	451.8662252	-0.547768319	0.200522381	0.000435342	0.013841802	Down	72865	retrotransposon Gag like 8C [Source:MGI Symbol;Acc:MGI:1920115]	Rtl8c	protein_coding
ENSMUSG00000051950	673.6077995	0.300222309	0.088697394	0.000153707	0.007637906	Up	381694	beta-3-glucosyltransferase [Source:MGI Symbol;Acc:MGI:2685903]	B3glct	protein_coding
ENSMUSG00000051951	40.19800096	-1.162456003	0.49825031	0.000658625	0.017631495	Down	497097	X-linked Kx blood group related 4 [Source:MGI Symbol;Acc:MGI:3528744]	Xkr4	protein_coding
ENSMUSG00000051977	159.5009107	0.421240218	0.169277099	0.001295748	0.025117695	Up	213389	PR domain containing 9 [Source:MGI Symbol;Acc:MGI:2384854]	Prdm9	protein_coding
ENSMUSG00000052155	425.9990713	0.478167631	0.140237991	6.41E-05	0.004869134	Up	11480	activin receptor IIA [Source:MGI Symbol;Acc:MGI:102806]	Acvr2a	protein_coding
ENSMUSG00000052253	495.33333	-0.546952501	0.12801283	1.55E-06	0.000667176	Down	52521	zinc finger protein 622 [Source:MGI Symbol;Acc:MGI:1289282]	Zfp622	protein_coding
ENSMUSG00000052337	4616.324325	0.401379079	0.110859051	4.75E-05	0.004134689	Up	76614	inner membrane protein, mitochondrial [Source:MGI Symbol;Acc:MGI:1923864]	Immt	protein_coding
ENSMUSG00000052392	223.6418313	0.711506874	0.284423538	0.000614375	0.016964332	Up	171282	acyl-CoA thioesterase 4 [Source:MGI Symbol;Acc:MGI:2159621]	Acot4	protein_coding
ENSMUSG00000052415	14.29723172	-1.117419155	0.453469823	0.000531771	0.015375846	Down	99681	trichohyalin [Source:MGI Symbol;Acc:MGI:2177944]	Tchh	protein_coding
ENSMUSG00000052566	123.6518622	-0.705125423	0.332593619	0.001423645	0.026099657	Down	170833	hook microtubule tethering protein 2 [Source:MGI Symbol;Acc:MGI:2181664]	Hook2	protein_coding
ENSMUSG00000052861	60.22663302	-0.071274454	0.190322549	0.003575784	0.043212822	Down	330355	dynein, axonemal, heavy chain 6 [Source:MGI Symbol;Acc:MGI:107744]	Dnah6	protein_coding
ENSMUSG00000052917	459.9565669	0.341340248	0.133224139	0.001528293	0.027179639	Up	66315	SUMO1/sentrin specific peptidase 7 [Source:MGI Symbol;Acc:MGI:1913565]	Senp7	protein_coding
ENSMUSG00000053062	1147.086201	0.414230914	0.172453676	0.001785338	0.029521437	Up	67374	junction adhesion molecule 2 [Source:MGI Symbol;Acc:MGI:1933820]	Jam2	protein_coding
ENSMUSG00000053286	591.5497828	0.423828697	0.12495629	8.00E-05	0.005299588	Up	98685	tRNA methyltransferase 1 like [Source:MGI Symbol;Acc:MGI:1916185]	Trmt1l	protein_coding
ENSMUSG00000053477	1658.516508	0.229887308	0.089321411	0.003302459	0.041299908	Up	21413	transcription factor 4 [Source:MGI Symbol;Acc:MGI:98506]	Tcf4	protein_coding
ENSMUSG00000053510	3071.954737	0.212372853	0.079493137	0.002780859	0.037264807	Up	230598	nardilysin, N-arginine dibasic convertase, NRD convertase 1 [Source:MGI Symbol;Acc:MGI:1201386]	Nrd1	protein_coding
ENSMUSG00000053553	102.8471047	-0.780284567	0.192598383	2.99E-06	0.000921546	Down	73212	RIKEN cDNA 3110082I17 gene [Source:MGI Symbol;Acc:MGI:1920462]	3110082I17Rik	protein_coding
ENSMUSG00000053646	314.9034723	-0.864627773	0.482260712	0.002227478	0.033613075	Down	235611	plexin B1 [Source:MGI Symbol;Acc:MGI:2154238]	Plxnb1	protein_coding
ENSMUSG00000053714	165.2958631	0.8141798	0.358392511	0.000948153	0.021358851	Up	654804	RIKEN cDNA 4732471J01 gene [Source:MGI Symbol;Acc:MGI:3603586]	4732471J01Rik	lncRNA
ENSMUSG00000053746	74.10084885	-0.994372055	0.243387856	2.28E-06	0.000834238	Down	329384	peptidyl-tRNA hydrolase 1 homolog [Source:MGI Symbol;Acc:MGI:1913779]	Ptrh1	protein_coding
ENSMUSG00000053774	825.5970877	0.30375123	0.110875658	0.001210772	0.024318448	Up	224111	UBX domain protein 7 [Source:MGI Symbol;Acc:MGI:2146388]	Ubxn7	protein_coding
ENSMUSG00000053819	2229.459624	0.335802082	0.120167364	0.000868299	0.02024816	Up	108058	calcium/calmodulin-dependent protein kinase II, delta [Source:MGI Symbol;Acc:MGI:1341265]	Camk2d	protein_coding
ENSMUSG00000053907	4759.181309	0.492420089	0.143781931	6.03E-05	0.00467818	Up	232087	methionine adenosyltransferase II, alpha [Source:MGI Symbol;Acc:MGI:2443731]	Mat2a	protein_coding
ENSMUSG00000053965	106.4579779	0.530383331	0.210563794	0.000821118	0.019680555	Up	242202	phosphodiesterase 5A, cGMP-specific [Source:MGI Symbol;Acc:MGI:2651499]	Pde5a	protein_coding
ENSMUSG00000054065	47.29164584	-1.477955102	0.415526394	1.50E-05	0.002229165	Down	56460	plakophilin 3 [Source:MGI Symbol;Acc:MGI:1891830]	Pkp3	protein_coding
ENSMUSG00000054091	845.4592264	-0.424100264	0.214230972	0.004124899	0.046766041	Down	67704	RIKEN cDNA 1810037I17 gene [Source:MGI Symbol;Acc:MGI:1914954]	1810037I17Rik	protein_coding
ENSMUSG00000054204	172.4883952	-0.914864289	0.299135449	0.000108626	0.006430038	Down	100294583	ALK and LTK ligand 2 [Source:MGI Symbol;Acc:MGI:3697448]	Alkal2	protein_coding
ENSMUSG00000054381	81.58972005	-0.451014057	0.232170222	0.003871165	0.045320504	Down	269997	zinc finger protein 747 [Source:MGI Symbol;Acc:MGI:2443581]	Zfp747	protein_coding
ENSMUSG00000054418	11.23668314	-1.482098848	0.587269592	0.000449472	0.013990982	Down	NA	RIKEN cDNA 2900041M22 gene [Source:MGI Symbol;Acc:MGI:1925653]	2900041M22Rik	lncRNA
ENSMUSG00000054428	319.1124755	-1.019727669	0.278799278	1.20E-05	0.00193348	Down	11983	ATPase inhibitory factor 1 [Source:MGI Symbol;Acc:MGI:1196457]	Atpif1	protein_coding
ENSMUSG00000054452	5083.765785	-0.452969129	0.236816856	0.004193551	0.04696359	Down	14797	TLE family member 5, transcriptional modulator [Source:MGI Symbol;Acc:MGI:95806]	Tle5	protein_coding
ENSMUSG00000054517	290.2924055	0.480133867	0.216720551	0.002029113	0.031835195	Up	338364	tripartite motif-containing 65 [Source:MGI Symbol;Acc:MGI:2442815]	Trim65	protein_coding
ENSMUSG00000054555	433.8391657	0.546967952	0.272701708	0.002665537	0.036638703	Up	11489	a disintegrin and metallopeptidase domain 12 (meltrin alpha) [Source:MGI Symbol;Acc:MGI:105378]	Adam12	protein_coding
ENSMUSG00000054604	1560.963078	0.271342118	0.082888151	0.000270694	0.010102419	Up	106143	CGG triplet repeat binding protein 1 [Source:MGI Symbol;Acc:MGI:2146370]	Cggbp1	protein_coding
ENSMUSG00000054737	185.6552223	0.472487124	0.190678715	0.001115835	0.023364734	Up	319535	zinc finger protein 182 [Source:MGI Symbol;Acc:MGI:2442220]	Zfp182	protein_coding
ENSMUSG00000054871	150.0070247	-0.884052223	0.380883194	0.000798668	0.019522616	Down	72309	transmembrane protein 158 [Source:MGI Symbol;Acc:MGI:1919559]	Tmem158	protein_coding
ENSMUSG00000055044	745.5709217	-0.428876443	0.163877907	0.000812625	0.019613515	Down	54132	PDZ and LIM domain 1 (elfin) [Source:MGI Symbol;Acc:MGI:1860611]	Pdlim1	protein_coding
ENSMUSG00000055320	1979.251581	0.479027397	0.201977888	0.001600819	0.027921974	Up	21676	TEA domain family member 1 [Source:MGI Symbol;Acc:MGI:101876]	Tead1	protein_coding
ENSMUSG00000055371	779.0926102	0.194838273	0.061737464	0.000678598	0.017881166	Up	56324	signal transducing adaptor molecule (SH3 domain and ITAM motif) 2 [Source:MGI Symbol;Acc:MGI:1929100]	Stam2	protein_coding
ENSMUSG00000055401	497.5566135	-0.286960248	0.110167554	0.001833632	0.030037054	Down	50762	F-box protein 6 [Source:MGI Symbol;Acc:MGI:1354743]	Fbxo6	protein_coding
ENSMUSG00000055436	2819.559069	0.445796124	0.140699717	0.000155427	0.007652757	Up	69207	serine and arginine-rich splicing factor 11 [Source:MGI Symbol;Acc:MGI:1916457]	Srsf11	protein_coding
ENSMUSG00000055531	1123.478788	0.268567864	0.100604226	0.001875133	0.030289224	Up	432508	cleavage and polyadenylation specific factor 6 [Source:MGI Symbol;Acc:MGI:1913948]	Cpsf6	protein_coding

ENSMUSG00000055629	11.62266142	-0.10299465	0.217132988	0.001583725	0.027693405	Down	330671	beta-1,4-N-acetyl-galactosaminyl transferase 4 [Source:MGI Symbol;Acc:MGI:2652891]	B4galnt4	protein_coding
ENSMUSG00000055704	9.944467619	-0.96955765	0.692627518	0.004155692	0.046948364	Down	NA	predicted gene 9978 [Source:MGI Symbol;Acc:MGI:3641806]	Gm9978	TEC
ENSMUSG00000055737	27244.42233	0.369149355	0.106608797	9.19E-05	0.005821151	Up	14600	growth hormone receptor [Source:MGI Symbol;Acc:MGI:95708]	Ghr	protein_coding
ENSMUSG00000055745	186.943316	-0.634321078	0.207348331	0.000141692	0.007452872	Down	223732	retrotransposon Gag like 6 [Source:MGI Symbol;Acc:MGI:2675858]	Rtl6	protein_coding
ENSMUSG00000055782	14163.34939	0.453350805	0.115651854	1.15E-05	0.00193348	Up	26874	ATP-binding cassette, sub-family D (ALD), member 2 [Source:MGI Symbol;Acc:MGI:1349467]	Abcd2	protein_coding
ENSMUSG00000055809	39.10709312	-1.020156904	0.593950022	0.002244934	0.033722017	Down	436022	dynein, axonemal assembly factor 3 [Source:MGI Symbol;Acc:MGI:3588207]	Dnaaf3	protein_coding
ENSMUSG00000055923	327.4995732	0.328285517	0.137540804	0.00258496	0.036128648	Up	231326	aminoadipate-semialdehyde dehydrogenase [Source:MGI Symbol;Acc:MGI:2442517]	Aasdh	protein_coding
ENSMUSG00000056162	59.1169867	-1.115491243	0.503432711	0.000852268	0.020037017	Down	338403	carnosine dipeptidase 1 (metallopeptidase M20 family) [Source:MGI Symbol;Acc:MGI:2451097]	Cndp1	protein_coding
ENSMUSG00000056174	85.09234607	-1.382025832	0.473098229	0.000124453	0.006874031	Down	329941	collagen, type VIII, alpha 2 [Source:MGI Symbol;Acc:MGI:88464]	Col8a2	protein_coding
ENSMUSG00000056204	430.5253894	-0.323916672	0.109832376	0.000566374	0.016114087	Down	66522	pyroglutamyl-peptidase I [Source:MGI Symbol;Acc:MGI:1913772]	Pgpep1	protein_coding
ENSMUSG00000056211	823.0340146	0.335713731	0.126399821	0.001276041	0.024791451	Up	226412	R3H domain containing 1 [Source:MGI Symbol;Acc:MGI:2448514]	R3hdm1	protein_coding
ENSMUSG00000056492	4015.405557	0.457566868	0.194999611	0.001625094	0.028224608	Up	224792	adhesion G protein-coupled receptor F5 [Source:MGI Symbol;Acc:MGI:2182928]	Adgrf5	protein_coding
ENSMUSG00000056501	1397.731674	-1.226107841	0.334543495	1.04E-05	0.001821577	Down	12608	CCAAT/enhancer binding protein (C/EBP), beta [Source:MGI Symbol;Acc:MGI:88373]	Cebpb	protein_coding
ENSMUSG00000056537	972.5839662	0.329954495	0.084086412	1.66E-05	0.002387315	Up	19820	ring finger protein, LIM domain interacting [Source:MGI Symbol;Acc:MGI:1342291]	Rlim	protein_coding
ENSMUSG00000056579	1347.388473	0.471773313	0.126156339	1.97E-05	0.002566184	Up	NA	taurine upregulated gene 1 [Source:MGI Symbol;Acc:MGI:2144114]	Tug1	protein_coding
ENSMUSG00000056596	119.0209984	-0.612241214	0.220051346	0.000342449	0.011616987	Down	69539	TMF1-regulated nuclear protein 1 [Source:MGI Symbol;Acc:MGI:1916789]	Trnp1	protein_coding
ENSMUSG00000056598	44.43694705	-1.498216637	0.721662257	0.000959224	0.021461264	Down	74665	dynein regulatory complex subunit 3 [Source:MGI Symbol;Acc:MGI:1921915]	Drc3	protein_coding
ENSMUSG00000056612	1294.520736	-0.564819693	0.222250951	0.000725333	0.018535693	Down	18938	protein phosphatase 1, regulatory inhibitor subunit 14B [Source:MGI Symbol;Acc:MGI:107682]	Ppp1r14b	protein_coding
ENSMUSG00000056629	689.4683697	-0.757775222	0.386288961	0.001788797	0.029527144	Down	14227	FK506 binding protein 2 [Source:MGI Symbol;Acc:MGI:95542]	Fkbp2	protein_coding
ENSMUSG00000056708	431.8098583	-0.76513963	0.411400215	0.002233909	0.033651183	Down	15939	immediate early response 5 [Source:MGI Symbol;Acc:MGI:1337072]	Ier5	protein_coding
ENSMUSG00000056832	55.40923153	-0.804067327	0.439071189	0.002251002	0.03374121	Down	264134	tetratricopeptide repeat domain 26 [Source:MGI Symbol;Acc:MGI:2444853]	Ttc26	protein_coding
ENSMUSG00000056870	536.9047105	1.352339866	0.315816574	8.44E-07	0.000454425	Up	70676	GULP, engulfment adaptor PTB domain containing 1 [Source:MGI Symbol;Acc:MGI:1920407]	Gulp1	protein_coding
ENSMUSG00000057068	21.7221945	-0.166302761	0.330618886	0.000167615	0.007913736	Down	384198	family with sequence similarity 47, member E [Source:MGI Symbol;Acc:MGI:2686227]	Fam47e	protein_coding
ENSMUSG00000057098	3007.695078	0.440112707	0.147332047	0.000301086	0.010720273	Up	13591	early B cell factor 1 [Source:MGI Symbol;Acc:MGI:95275]	Ebf1	protein_coding
ENSMUSG00000057176	69.73031871	-1.313355771	0.411744662	5.64E-05	0.00449019	Down	233899	coiled-coil domain containing 189 [Source:MGI Symbol;Acc:MGI:2685012]	Ccdc189	protein_coding
ENSMUSG00000057411	760.8646683	-0.522331937	0.199755159	0.000648239	0.017454853	Down	214917	adenine nucleotide translocase lysine methyltransferase [Source:MGI Symbol;Acc:MGI:2384888]	Antkmt	protein_coding
ENSMUSG00000057465	12.29189846	0.026075652	0.171096835	0.00238077	0.034681162	Up	20209	serum amyloid A 2 [Source:MGI Symbol;Acc:MGI:98222]	Saa2	protein_coding
ENSMUSG00000057667	258.7667819	-0.324211312	0.12567733	0.001643021	0.028305152	Down	232946	biogenesis of lysosomal organelles complex-1, subunit 3 [Source:MGI Symbol;Acc:MGI:2678952]	Bloc1s3	protein_coding
ENSMUSG00000057706	42.08892859	-0.833363509	0.354337049	0.000784584	0.019315327	Down	108797	mex3 RNA binding family member B [Source:MGI Symbol;Acc:MGI:1918252]	Mex3b	protein_coding
ENSMUSG00000057716	79.6643086	0.594334773	0.34944429	0.003973757	0.045835427	Up	434008	transmembrane protein 178B [Source:MGI Symbol;Acc:MGI:3647581]	Tmem178b	protein_coding
ENSMUSG00000057841	3998.718959	-0.55583841	0.205194861	0.00047091	0.014350473	Down	19951	ribosomal protein L32 [Source:MGI Symbol;Acc:MGI:98038]	Rpl32	protein_coding
ENSMUSG00000057863	3003.764321	-0.628313629	0.217826856	0.000247404	0.009734066	Down	54217	ribosomal protein L36 [Source:MGI Symbol;Acc:MGI:1860603]	Rpl36	protein_coding
ENSMUSG00000057880	1507.13561	-0.527980039	0.232108129	0.001488632	0.026877103	Down	268860	4-aminobutyrate aminotransferase [Source:MGI Symbol;Acc:MGI:2443582]	Abat	protein_coding
ENSMUSG00000057969	324.5956789	-0.950878992	0.25707321	1.17E-05	0.00193348	Down	20347	sema domain, immunoglobulin domain (Ig), short basic domain, secreted, (semaphorin) 3B [Source:MGI Symbol;Acc:MGI:107561]	Sema3b	protein_coding
ENSMUSG00000058135	10279.54952	-0.430965282	0.14314764	0.00026771	0.010095082	Down	14862	glutathione S-transferase, mu 1 [Source:MGI Symbol;Acc:MGI:95860]	Gstm1	protein_coding
ENSMUSG00000058254	1624.327202	0.354126291	0.104780562	0.000126147	0.006945362	Up	21912	tetraspanin 7 [Source:MGI Symbol;Acc:MGI:1298407]	Tspan7	protein_coding
ENSMUSG00000058258	448.6330303	0.562446701	0.154099375	2.20E-05	0.002705768	Up	319554	isopentenyl-diphosphate delta isomerase [Source:MGI Symbol;Acc:MGI:2442264]	Idi1	protein_coding
ENSMUSG00000058355	1601.522818	0.295783144	0.115339349	0.002095843	0.032538438	Up	24015	ATP-binding cassette, sub-family E (OABP), member 1 [Source:MGI Symbol;Acc:MGI:1195458]	Abce1	protein_coding
ENSMUSG00000058446	3898.477918	0.314759014	0.10709136	0.000641398	0.017324783	Up	387524	zinc and ring finger 2 [Source:MGI Symbol;Acc:MGI:1196246]	Znrf2	protein_coding
ENSMUSG00000058569	1372.36001	-0.568646675	0.171169128	7.12E-05	0.005090915	Down	67511	transmembrane p24 trafficking protein 9 [Source:MGI Symbol;Acc:MGI:1914761]	Tmed9	protein_coding
ENSMUSG00000058656	92.54863699	0.449093336	0.240955353	0.004627709	0.049564519	Up	320679	sterile alpha motif domain containing 12 [Source:MGI Symbol;Acc:MGI:2444518]	Samd12	protein_coding
ENSMUSG00000058706	761.0927498	0.300106828	0.114513565	0.001726822	0.029034732	Up	68364	RIKEN cDNA 0610030E20 gene [Source:MGI Symbol;Acc:MGI:1915614]	0610030E20Rik	protein_coding
ENSMUSG00000058709	1060.535689	-0.391120416	0.125141931	0.000230673	0.009266171	Down	112406	egl-9 family hypoxia-inducible factor 2 [Source:MGI Symbol;Acc:MGI:1932287]	Egln2	protein_coding
ENSMUSG00000058743	147.0387074	-1.439031302	0.58936752	0.00044158	0.013886414	Down	211480	potassium inwardly-rectifying channel, subfamily J, member 14 [Source:MGI Symbol;Acc:MGI:2384820]	Kcnj14	protein_coding
ENSMUSG00000058833	1076.196818	-0.447211193	0.204477546	0.002432923	0.034967948	Down	66462	required for excision 1-B domain containing [Source:MGI Symbol;Acc:MGI:1913712]	Rex1bd	protein_coding
ENSMUSG00000058914	37.7122521	-1.116730734	0.402288129	0.000217081	0.009168997	Down	81799	C1q and tumor necrosis factor related protein 3 [Source:MGI Symbol;Acc:MGI:1932136]	C1qtnf3	protein_coding
ENSMUSG00000059027	340.3383639	0.476378052	0.231260438	0.002867067	0.037986425	Up	NA	RIKEN cDNA 9630013D21 gene [Source:MGI Symbol;Acc:MGI:2442649]	9630013D21Rik	lncRNA
ENSMUSG00000059201	37111.74757	-0.873026951	0.60720853	0.003596978	0.043377689	Down	16846	leptin [Source:MGI Symbol;Acc:MGI:104663]	Lep	protein_coding
ENSMUSG00000059278	282.4929855	-0.62652468	0.361040177	0.003508789	0.042853972	Down	78304	N(alpha)-acetyltransferase 38, NatC auxiliary subunit [Source:MGI Symbol;Acc:MGI:1925554]	Naa38	protein_coding
ENSMUSG00000059291	5121.582696	-0.580093049	0.211810431	0.000440575	0.013880126	Down	67025	ribosomal protein L11 [Source:MGI Symbol;Acc:MGI:1914275]	Rpl11	protein_coding
ENSMUSG00000059412	194.6811062	-1.53377163	0.298507682	1.27E-08	3.65E-05	Down	11936	FXYD domain-containing ion transport regulator 2 [Source:MGI Symbol;Acc:MGI:1195260]	Fxyd2	protein_coding

ENSMUSG00000059475	369.7101643	0.413129374	0.15498336	0.00083359	0.019759637	Up	235028	zinc finger protein 426 [Source:MGI Symbol;Acc:MGI:1920248]	Zfp426	protein_coding
ENSMUSG00000059479	98.94297335	-0.518530662	0.223914872	0.0014148	0.026099657	Down	232984	UDP-GlcNAc:betaGal beta-1,3-N-acetylglucosaminyltransferase 8 [Source:MGI Symbol;Acc:MGI:2385269]	B3gnt8	protein_coding
ENSMUSG00000059555	327.6609995	-0.406656709	0.149760452	0.000725268	0.018535693	Down	227612	torsin family 4, member A [Source:MGI Symbol;Acc:MGI:2442720]	Tor4a	protein_coding
ENSMUSG00000059714	1974.819548	-0.485411747	0.177185556	0.000518648	0.015123297	Down	14251	flotillin 1 [Source:MGI Symbol;Acc:MGI:1100500]	Flot1	protein_coding
ENSMUSG00000059791	102.6603931	-0.468288173	0.246881645	0.004044864	0.046192933	Down	106582	nurim (nuclear envelope membrane protein) [Source:MGI Symbol;Acc:MGI:2146855]	Nrm	protein_coding
ENSMUSG00000059854	61.50209507	-0.105905403	0.220438666	0.001395645	0.02591718	Down	244653	HYDIN, axonemal central pair apparatus protein [Source:MGI Symbol;Acc:MGI:2389007]	Hydin	protein_coding
ENSMUSG00000059890	2099.597682	0.314762499	0.095100846	0.000178704	0.008185824	Up	140630	ubiquitination factor E4A [Source:MGI Symbol;Acc:MGI:2154580]	Ube4a	protein_coding
ENSMUSG00000059970	368.3038487	-0.843742815	0.258152771	5.32E-05	0.004409981	Down	15512	heat shock protein 2 [Source:MGI Symbol;Acc:MGI:96243]	Hspa2	protein_coding
ENSMUSG00000059994	11.19220358	-0.07216108	0.190909794	0.003226878	0.040682063	Down	229499	Fc receptor-like 1 [Source:MGI Symbol;Acc:MGI:2442862]	Fcrl1	protein_coding
ENSMUSG00000060002	29379.76215	0.424626904	0.120455312	4.99E-05	0.004254642	Up	212862	choline phosphotransferase 1 [Source:MGI Symbol;Acc:MGI:2384841]	Chpt1	protein_coding
ENSMUSG00000060032	794.7309115	-1.246470851	0.2925535	9.20E-07	0.000466086	Down	232440	H2J.A histone [Source:MGI Symbol;Acc:MGI:3606192]	H2aj	protein_coding
ENSMUSG00000060063	307.6946428	-0.548562546	0.325589952	0.004443818	0.048407284	Down	11690	arachidonate 5-lipoxygenase activating protein [Source:MGI Symbol;Acc:MGI:107505]	Alox5ap	protein_coding
ENSMUSG00000060176	31.15645168	-1.050087763	0.800049739	0.003625675	0.043597946	Down	75050	kinesin family member 27 [Source:MGI Symbol;Acc:MGI:1922300]	Kif27	protein_coding
ENSMUSG00000060224	237.3242533	-0.427303122	0.187115264	0.002048339	0.031973755	Down	74580	pyridine nucleotide-disulphide oxidoreductase domain 2 [Source:MGI Symbol;Acc:MGI:1921830]	Pyroxd2	protein_coding
ENSMUSG00000060429	3104.630377	0.642102032	0.1577473	3.43E-06	0.000946988	Up	20649	syntrophin, basic 1 [Source:MGI Symbol;Acc:MGI:101781]	Sntb1	protein_coding
ENSMUSG00000060512	15.83375764	-0.182251659	0.374093473	0.000467452	0.014308361	Down	76261	RIKEN cDNA 0610040J01 gene [Source:MGI Symbol;Acc:MGI:1923511]	0610040J01Rik	protein_coding
ENSMUSG00000060572	79.5361572	-0.625532282	0.344809008	0.00297483	0.038516848	Down	17150	microfibrillar-associated protein 2 [Source:MGI Symbol;Acc:MGI:99559]	Mfap2	protein_coding
ENSMUSG00000060591	1350.974804	-0.650845185	0.116692617	1.89E-09	1.01E-05	Down	80876	interferon induced transmembrane protein 2 [Source:MGI Symbol;Acc:MGI:1933382]	Ifitm2	protein_coding
ENSMUSG00000060601	1334.058514	-0.264673444	0.111092952	0.004387851	0.048144948	Down	22260	nuclear receptor subfamily 1, group H, member 2 [Source:MGI Symbol;Acc:MGI:1352463]	Nr1h2	protein_coding
ENSMUSG00000060613	13.97249576	2.26570857	1.626652815	0.002391385	0.03472722	Up	226105	cytochrome P450, family 2, subfamily c, polypeptide 70 [Source:MGI Symbol;Acc:MGI:2385878]	Cyp2c70	protein_coding
ENSMUSG00000060657	1390.742619	0.306843143	0.109191533	0.001059969	0.022737013	Up	223989	meiosis regulator and mRNA stability 1 [Source:MGI Symbol;Acc:MGI:2444505]	Marf1	protein_coding
ENSMUSG00000060716	41.59649999	-0.10154051	0.21434231	0.004493681	0.048662455	Down	211945	pleckstrin homology domain containing, family H (with MyTH4 domain) member 1 [Source:MGI Symbol;Acc:MGI:2144989]	Plekhh1	protein_coding
ENSMUSG00000060743	2223.7398	-0.466696153	0.17042007	0.000603169	0.016711276	Down	15078	H3.3 histone A [Source:MGI Symbol;Acc:MGI:1097686]	H3f3a	protein_coding
ENSMUSG00000060791	198.6564936	-0.666785862	0.34340482	0.00214242	0.032776559	Down	63986	glia maturation factor, gamma [Source:MGI Symbol;Acc:MGI:1927135]	Gmfg	protein_coding
ENSMUSG00000060950	215.3712009	-0.522019192	0.201762696	0.000715922	0.018469289	Down	328162	tRNA methyltransferase 61A [Source:MGI Symbol;Acc:MGI:2443487]	Trmt61a	protein_coding
ENSMUSG00000060962	90.59834955	-1.392178784	0.437058605	5.51E-05	0.004447279	Down	73712	dermokine [Source:MGI Symbol;Acc:MGI:1920962]	Dmkn	protein_coding
ENSMUSG00000060981	511.5354449	-0.945037523	0.245888617	5.72E-06	0.001281	Down	69386	H4 clustered histone 8 [Source:MGI Symbol;Acc:MGI:2448427]	H4c8	protein_coding
ENSMUSG00000061046	157.7441145	-0.44041548	0.198138692	0.002251633	0.03374121	Down	68977	hydroxyacylglutathione hydrolase-like [Source:MGI Symbol;Acc:MGI:1919877]	Haghl	protein_coding
ENSMUSG00000061048	126.6022912	-0.670734469	0.324790126	0.001748325	0.02919465	Down	12560	cadherin 3 [Source:MGI Symbol;Acc:MGI:88356]	Cdh3	protein_coding
ENSMUSG00000061086	172.4201013	-0.80968593	0.492568455	0.002966329	0.038516848	Down	17896	myosin, light polypeptide 4 [Source:MGI Symbol;Acc:MGI:97267]	Myl4	protein_coding
ENSMUSG00000061273	1228.62128	0.464775871	0.137505823	7.30E-05	0.005101093	Up	236792	membrane magnesium transporter 1 [Source:MGI Symbol;Acc:MGI:2384305]	Mmgt1	protein_coding
ENSMUSG00000061313	1755.077507	0.566615228	0.175770072	9.43E-05	0.005856894	Up	72108	DDHD domain containing 2 [Source:MGI Symbol;Acc:MGI:1919358]	Ddhd2	protein_coding
ENSMUSG00000061397	150.3514696	-0.835858217	0.382609009	0.001101852	0.02321297	Down	223917	keratin 79 [Source:MGI Symbol;Acc:MGI:2385030]	Krt79	protein_coding
ENSMUSG00000061518	1500.12686	-0.535513796	0.301719635	0.003886877	0.045354164	Down	12859	cytochrome c oxidase subunit 5B [Source:MGI Symbol;Acc:MGI:88475]	Cox5b	protein_coding
ENSMUSG00000061535	216.762072	-0.315228333	0.132256804	0.002935896	0.038383182	Down	109323	C1q and tumor necrosis factor related protein 7 [Source:MGI Symbol;Acc:MGI:1925911]	C1qtnf7	protein_coding
ENSMUSG00000061650	605.0414036	-0.444090929	0.153855984	0.000390633	0.012773761	Down	192191	mediator complex subunit 9 [Source:MGI Symbol;Acc:MGI:2183151]	Med9	protein_coding
ENSMUSG00000061718	76.59167691	-0.053584522	0.180865127	0.000718218	0.018500824	Down	19049	protein phosphatase 1, regulatory inhibitor subunit 1B [Source:MGI Symbol;Acc:MGI:94860]	Ppp1r1b	protein_coding
ENSMUSG00000061787	3401.331564	-0.467931041	0.164811233	0.000396967	0.012931806	Down	20068	ribosomal protein S17 [Source:MGI Symbol;Acc:MGI:1309526]	Rps17	protein_coding
ENSMUSG00000062012	93.5335789	-0.494638027	0.237315732	0.002518625	0.035547476	Down	22654	zinc finger protein 13 [Source:MGI Symbol;Acc:MGI:99159]	Zfp13	protein_coding
ENSMUSG00000062075	196.2878606	-0.45830084	0.167919293	0.000596783	0.016587689	Down	16907	lamin B2 [Source:MGI Symbol;Acc:MGI:96796]	Lmnb2	protein_coding
ENSMUSG00000062078	5013.075055	0.376507101	0.09364296	8.74E-06	0.001585952	Up	19317	quaking, KH domain containing RNA binding [Source:MGI Symbol;Acc:MGI:97837]	Qki	protein_coding
ENSMUSG00000062157	24.71695711	-1.176014282	0.48440297	0.000522485	0.015209427	Down	242700	interferon lambda receptor 1 [Source:MGI Symbol;Acc:MGI:2429859]	Ifnlr1	protein_coding
ENSMUSG00000062661	78.05754934	-0.600592097	0.265641705	0.001312471	0.025189625	Down	14299	neuronal calcium sensor 1 [Source:MGI Symbol;Acc:MGI:109166]	Ncs1	protein_coding
ENSMUSG00000062859	19.84289735	-0.0899834	0.206513846	2.41E-05	0.002792968	Down	21463	t-complex protein 11 [Source:MGI Symbol;Acc:MGI:98544]	Tcp11	protein_coding
ENSMUSG00000062929	1874.753602	0.296578198	0.128719772	0.004049003	0.046199963	Up	12632	cofilin 2, muscle [Source:MGI Symbol;Acc:MGI:101763]	Cfl2	protein_coding
ENSMUSG00000062949	641.9515094	0.560738516	0.16027899	3.72E-05	0.003584747	Up	320940	ATPase, class VI, type 11C [Source:MGI Symbol;Acc:MGI:1859661]	Atp11c	protein_coding
ENSMUSG00000062997	2545.674483	-0.908284184	0.313539642	0.00015415	0.007637906	Down	66489	ribosomal protein L35 [Source:MGI Symbol;Acc:MGI:1913739]	Rpl35	protein_coding
ENSMUSG00000063019	388.2346476	-0.419974468	0.172663776	0.001222447	0.024468519	Down	69161	mannosidase, beta A, lysosomal-like [Source:MGI Symbol;Acc:MGI:1916411]	Manbal	protein_coding
ENSMUSG00000063316	3541.210045	-0.558021869	0.151407462	1.68E-05	0.002391284	Down	19942	ribosomal protein L27 [Source:MGI Symbol;Acc:MGI:98036]	Rpl27	protein_coding
ENSMUSG00000063358	2930.807663	0.22555904	0.063298605	0.000131687	0.007086499	Up	26413	mitogen-activated protein kinase 1 [Source:MGI Symbol;Acc:MGI:1346858]	Mapk1	protein_coding
ENSMUSG00000063439	121.6973437	-0.394525292	0.177812812	0.002663032	0.036638703	Down	232987	B9 protein domain 2 [Source:MGI Symbol;Acc:MGI:2387643]	B9d2	protein_coding
ENSMUSG00000063457	4719.930781	-0.668104741	0.251037985	0.000423944	0.013554397	Down	20054	ribosomal protein S15 [Source:MGI Symbol;Acc:MGI:98117]	Rps15	protein_coding
ENSMUSG00000063529	5.440701127	-0.105013259	0.218702381	0.002268783	0.033800769	Down	380842	stathmin domain containing 1 [Source:MGI Symbol;Acc:MGI:2686420]	Stmnd1	protein_coding
ENSMUSG00000063568	384.9816634	0.281711846	0.123280813	0.004633132	0.049578114	Up	231986	JAZF zinc finger 1 [Source:MGI Symbol;Acc:MGI:2141450]	Jazf1	protein_coding
ENSMUSG00000063605	240.5756205	-0.735950025	0.192483017	7.84E-06	0.001518562	Down	234582	coiled-coil domain containing 102A [Source:MGI Symbol;Acc:MGI:2686927]	Ccdc102a	protein_coding
ENSMUSG00000063884	817.707349	0.368172506	0.095551148	1.85E-05	0.002550282	Up	69956	pentatricopeptide repeat domain 3 [Source:MGI Symbol;Acc:MGI:1917206]	Ptcd3	protein_coding

ENSMUSG00000063904	809.3632439	-0.40125838	0.139503639	0.000471326	0.014350473	Down	75221	dipeptidylpeptidase 3 [Source:MGI Symbol;Acc:MGI:1922471]	Dpp3	protein_coding
ENSMUSG00000064341	70477.38633	0.555914344	0.156747607	3.16E-05	0.003266187	Up	17716	mitochondrially encoded NADH dehydrogenase 1 [Source:MGI Symbol;Acc:MGI:101787]	mt-Nd1	protein_coding
ENSMUSG00000064345	65081.00168	0.800269452	0.218831209	1.95E-05	0.002566184	Up	17717	mitochondrially encoded NADH dehydrogenase 2 [Source:MGI Symbol;Acc:MGI:102500]	mt-Nd2	protein_coding
ENSMUSG00000064351	313672.5894	0.72535305	0.201258847	1.93E-05	0.002566184	Up	17708	mitochondrially encoded cytochrome c oxidase I [Source:MGI Symbol;Acc:MGI:102504]	mt-Co1	protein_coding
ENSMUSG00000064354	1009.834142	0.553936687	0.19728993	0.000362325	0.01207727	Up	17709	mitochondrially encoded cytochrome c oxidase II [Source:MGI Symbol;Acc:MGI:102503]	mt-Co2	protein_coding
ENSMUSG00000064357	1155.704389	0.835633993	0.215137371	5.79E-06	0.001281	Up	17705	mitochondrially encoded ATP synthase 6 [Source:MGI Symbol;Acc:MGI:99927]	mt-Atp6	protein_coding
ENSMUSG00000064360	329.2004569	0.718790246	0.303130191	0.000854805	0.020060187	Up	17718	mitochondrially encoded NADH dehydrogenase 3 [Source:MGI Symbol;Acc:MGI:102499]	mt-Nd3	protein_coding
ENSMUSG00000064363	73409.62086	0.743504734	0.189547564	5.46E-06	0.001275029	Up	17719	mitochondrially encoded NADH dehydrogenase 4 [Source:MGI Symbol;Acc:MGI:102498]	mt-Nd4	protein_coding
ENSMUSG00000064366	45.05699138	0.889619815	0.352238534	0.000484221	0.01451232	Up	NA	mitochondrially encoded tRNA leucine 2 [Source:MGI Symbol;Acc:MGI:102481]	mt-Tl2	Mt_tRNA
ENSMUSG00000064367	97638.1055	0.897789766	0.267017921	3.89E-05	0.003705074	Up	17721	mitochondrially encoded NADH dehydrogenase 5 [Source:MGI Symbol;Acc:MGI:102496]	mt-Nd5	protein_coding
ENSMUSG00000064368	32708.49003	0.980181909	0.266905215	1.20E-05	0.00193348	Up	17722	mitochondrially encoded NADH dehydrogenase 6 [Source:MGI Symbol;Acc:MGI:102495]	mt-Nd6	protein_coding
ENSMUSG00000064370	165241.1753	0.745654619	0.17409376	1.19E-06	0.000537988	Up	17711	mitochondrially encoded cytochrome b [Source:MGI Symbol;Acc:MGI:102501]	mt-Cytb	protein_coding
ENSMUSG00000065947	222.5507846	0.91529396	0.292813667	8.30E-05	0.005437165	Up	17720	mitochondrially encoded NADH dehydrogenase 4L [Source:MGI Symbol;Acc:MGI:102497]	mt-Nd4l	protein_coding
ENSMUSG00000066154	121.9893906	1.938956164	2.06723198	0.00325714	0.040971021	Up	17842	major urinary protein 3 [Source:MGI Symbol;Acc:MGI:97235]	Mup3	protein_coding
ENSMUSG00000066232	1907.3301	0.296051008	0.128238385	0.004251688	0.047293134	Up	233726	importin 7 [Source:MGI Symbol;Acc:MGI:2152414]	Ipo7	protein_coding
ENSMUSG00000066235	269.5491691	-0.321944763	0.142392642	0.00379272	0.044920786	Down	215494	protein O-linked mannose beta 1,4-N-acetylglucosaminyltransferase 2 [Source:MGI Symbol;Acc:MGI:2143424]	Pomgnt2	protein_coding
ENSMUSG00000066315	21.34770138	-0.930050833	0.508167868	0.002134315	0.032697518	Down	NA	predicted gene 12918 [Source:MGI Symbol;Acc:MGI:3652005]	Gm12918	processed_pseudogene
ENSMUSG00000066324	1636.382418	0.242762768	0.091813414	0.002481717	0.035286651	Up	242291	3'(2'), 5'-bisphosphate nucleotidase 2 [Source:MGI Symbol;Acc:MGI:1915720]	Bpnt2	protein_coding
ENSMUSG00000066357	963.4291808	-0.40853885	0.191134983	0.003457143	0.042615836	Down	83669	WD repeat domain 6 [Source:MGI Symbol;Acc:MGI:1930140]	Wdr6	protein_coding
ENSMUSG00000066366	147.2459189	1.025756638	0.435106563	0.000661642	0.017650281	Up	20700	serine (or cysteine) peptidase inhibitor, clade A, member 1A [Source:MGI Symbol;Acc:MGI:891971]	Serpina1a	protein_coding
ENSMUSG00000066613	231.4720277	0.312040105	0.13925902	0.004179789	0.04696359	Up	69504	zinc finger protein 932 [Source:MGI Symbol;Acc:MGI:1916754]	Zfp932	protein_coding
ENSMUSG00000066705	205.9281371	-0.7803464	0.281345812	0.000270115	0.010102419	Down	59095	FXYD domain-containing ion transport regulator 6 [Source:MGI Symbol;Acc:MGI:1890226]	Fxyd6	protein_coding
ENSMUSG00000066877	482.0713488	-0.319576887	0.102553835	0.00030824	0.010774635	Down	17974	non-catalytic region of tyrosine kinase adaptor protein 2 [Source:MGI Symbol;Acc:MGI:1306821]	Nck2	protein_coding
ENSMUSG00000066894	185.7670171	-0.413095709	0.171345637	0.001709904	0.028849913	Down	231668	V-set and immunoglobulin domain containing 10 [Source:MGI Symbol;Acc:MGI:2448533]	Vsig10	protein_coding
ENSMUSG00000067071	194.4351578	-0.78377427	0.22543075	2.84E-05	0.003106886	Down	55927	hairy and enhancer of split 6 [Source:MGI Symbol;Acc:MGI:1859852]	Hes6	protein_coding
ENSMUSG00000067235	214.9093256	1.784581329	0.465682248	5.16E-06	0.001244765	Up	15007	histocompatibility 2, Q region locus 10 [Source:MGI Symbol;Acc:MGI:95929]	H2-Q10	protein_coding
ENSMUSG00000067336	2122.752491	0.47441581	0.122559832	1.09E-05	0.001900639	Up	12168	bone morphogenetic protein receptor, type II (serine/threonine kinase) [Source:MGI Symbol;Acc:MGI:1095407]	Bmpr2	protein_coding
ENSMUSG00000067736	248.1487446	0.809753039	0.291075536	0.000260951	0.009990243	Up	NA	predicted gene 10222 [Source:MGI Symbol;Acc:MGI:3642643]	Gm10222	unprocessed_pseudogene
ENSMUSG00000067787	2660.508817	0.460429698	0.172840882	0.000689409	0.018000888	Up	53619	bladder cancer associated protein [Source:MGI Symbol;Acc:MGI:1858907]	Blcap	protein_coding
ENSMUSG00000067847	603.8319965	-0.938474226	0.274088205	2.93E-05	0.003152842	Down	67067	reactive oxygen species modulator 1 [Source:MGI Symbol;Acc:MGI:1914317]	Romo1	protein_coding
ENSMUSG00000068290	1603.824046	-0.476037662	0.245207299	0.003542105	0.043004611	Down	77006	DDRGK domain containing 1 [Source:MGI Symbol;Acc:MGI:1924256]	Ddrgk1	protein_coding
ENSMUSG00000068335	198.5756948	-0.642920113	0.258274218	0.000697968	0.018169318	Down	13448	docking protein 1 [Source:MGI Symbol;Acc:MGI:893587]	Dok1	protein_coding
ENSMUSG00000068566	4391.732366	0.376537741	0.089321506	3.71E-06	0.000946988	Up	50918	myeloid-associated differentiation marker [Source:MGI Symbol;Acc:MGI:1355332]	Myadm	protein_coding
ENSMUSG00000068587	25.7321563	-1.712363624	0.766342676	0.000645259	0.017401805	Down	232714	maltase-glucoamylase [Source:MGI Symbol;Acc:MGI:1203495]	Mgam	protein_coding
ENSMUSG00000068758	327.5305334	-0.50407776	0.251947994	0.002895488	0.038235978	Down	16188	interleukin 3 receptor, alpha chain [Source:MGI Symbol;Acc:MGI:96553]	Il3ra	protein_coding
ENSMUSG00000068762	205.2395929	-1.339576109	0.335599688	2.86E-06	0.000921546	Down	14867	glutathione S-transferase, mu 6 [Source:MGI Symbol;Acc:MGI:1309467]	Gstm6	protein_coding
ENSMUSG00000069237	2156.950945	0.229045938	0.080935736	0.001501129	0.026974929	Up	97863	family with sequence similarity 8, member A1 [Source:MGI Symbol;Acc:MGI:2145496]	Fam8a1	protein_coding
ENSMUSG00000069515	1146.067842	-0.716253215	0.317470603	0.001067106	0.022737013	Down	17110	lysozyme 1 [Source:MGI Symbol;Acc:MGI:96902]	Lyz1	protein_coding
ENSMUSG00000069539	730.2910681	0.303506757	0.099671569	0.000474549	0.014362153	Up	213326	SCY1-like 2 (S. cerevisiae) [Source:MGI Symbol;Acc:MGI:1289172]	Scyl2	protein_coding
ENSMUSG00000070003	674.4707176	-0.661440093	0.295400628	0.001246169	0.024468519	Down	76900	single stranded DNA binding protein 4 [Source:MGI Symbol;Acc:MGI:1924150]	Ssbp4	protein_coding
ENSMUSG00000070343	39.70514928	-0.816188418	0.32104607	0.000503023	0.014833024	Down	NA	predicted gene 10288 [Source:MGI Symbol;Acc:MGI:3704227]	Gm10288	processed_pseudogene
ENSMUSG00000070427	106.7757595	0.432312114	0.227577863	0.004583682	0.049276724	Up	16068	interleukin 18 binding protein [Source:MGI Symbol;Acc:MGI:1333800]	Il18bp	protein_coding
ENSMUSG00000070473	58.60373945	-1.22878296	0.419963161	0.000130181	0.00705474	Down	12739	claudin 3 [Source:MGI Symbol;Acc:MGI:1329044]	Cldn3	protein_coding
ENSMUSG00000070544	1704.640309	0.228520107	0.084926426	0.002427829	0.034953036	Up	21969	topoisomerase (DNA) I [Source:MGI Symbol;Acc:MGI:98788]	Top1	protein_coding
ENSMUSG00000070610	15.0420968	-0.834788461	0.421648082	0.001858371	0.030184084	Down	NA	predicted gene 13127 [Source:MGI Symbol;Acc:MGI:3651260]	Gm13127	processed_pseudogene
ENSMUSG00000070803	32.27722519	-0.112700215	0.228827288	0.0008208	0.019680555	Down	56222	Cbp/p300-interacting transactivator, with Glu/Asp-rich carboxy-terminal domain, 4 [Source:MGI Symbol;Acc:MGI:1861694]	Cited4	protein_coding
ENSMUSG00000070868	30.49348776	-0.991183262	0.5295295	0.0018648	0.030231513	Down	195564	selection and upkeep of intraepithelial T cells 3 [Source:MGI Symbol;Acc:MGI:3045331]	Skint3	protein_coding
ENSMUSG00000070871	349.1377359	0.307999305	0.11584486	0.001498464	0.026974929	Up	227210	cyclin Y-like 1 [Source:MGI Symbol;Acc:MGI:2138614]	Ccnyl1	protein_coding
ENSMUSG00000070972	357.1918874	0.287547782	0.120732011	0.00346921	0.042649262	Up	72429	DnaJ heat shock protein family (Hsp40) member C25 [Source:MGI Symbol;Acc:MGI:1919679]	Dnajc25	protein_coding
ENSMUSG00000071178	99.9370677	1.507652513	1.012162984	0.00250684	0.035468996	Up	20701	serine (or cysteine) preptidase inhibitor, clade A, member 1B [Source:MGI Symbol;Acc:MGI:891970]	Serpina1b	protein_coding

ENSMUSG00000071398	27.49193719	-0.086930759	0.203046282	0.000319309	0.011049498	Down	73667	RIKEN cDNA 2410004P03 gene [Source:MGI Symbol;Acc:MGI:1920917]	2410004P03Rik	protein_coding
ENSMUSG00000071415	3876.952547	-0.68491659	0.190393916	2.08E-05	0.002600071	Down	65019	ribosomal protein L23 [Source:MGI Symbol;Acc:MGI:1929455]	Rpl23	protein_coding
ENSMUSG00000071547	106.8913331	-0.574369323	0.221915774	0.000624642	0.017069631	Down	70021	5'-nucleotidase domain containing 2 [Source:MGI Symbol;Acc:MGI:1917271]	Nt5dc2	protein_coding
ENSMUSG00000071632	215.4537565	-0.373509312	0.157122022	0.002124036	0.032652559	Down	72307	RIKEN cDNA 2510002D24 gene [Source:MGI Symbol;Acc:MGI:1919557]	2510002D24Rik	protein_coding
ENSMUSG00000071637	962.5908996	-1.083065635	0.289781521	8.57E-06	0.001571476	Down	12609	CCAAT/enhancer binding protein (C/EBP), delta [Source:MGI Symbol;Acc:MGI:103573]	Cebpd	protein_coding
ENSMUSG00000071654	339.9544183	-0.699555381	0.247435708	0.00025475	0.009843284	Down	107197	ubiquinol-cytochrome c reductase complex assembly factor 3 [Source:MGI Symbol;Acc:MGI:2147553]	Uqcc3	protein_coding
ENSMUSG00000071661	31.23862631	-0.630428671	0.330273258	0.002589646	0.036162167	Down	75291	zinc finger and BTB domain containing 3 [Source:MGI Symbol;Acc:MGI:1922541]	Zbtb3	protein_coding
ENSMUSG00000072769	94.50458856	0.890983574	0.309175734	0.00017743	0.008185484	Up	NA	predicted gene 10419 [Source:MGI Symbol;Acc:MGI:3642823]	Gm10419	lncRNA
ENSMUSG00000072847	62.84168671	0.452819939	0.180356913	0.001108893	0.023332795	Up	NA	RIKEN cDNA A530017D24 gene [Source:MGI Symbol;Acc:MGI:2144320]	A530017D24Rik	lncRNA
ENSMUSG00000072849	119.6798998	2.150719624	2.060422017	0.002928879	0.038324503	Up	20704	serine (or cysteine) peptidase inhibitor, clade A, member 1E [Source:MGI Symbol;Acc:MGI:891967]	Serpina1e	protein_coding
ENSMUSG00000072875	171.5846329	-0.560196966	0.224907264	0.00081104	0.019613515	Down	14761	G protein-coupled receptor 27 [Source:MGI Symbol;Acc:MGI:1202299]	Gpr27	protein_coding
ENSMUSG00000072949	51.84049102	-2.344243102	0.539733114	5.37E-07	0.000370312	Down	26897	acyl-CoA thioesterase 1 [Source:MGI Symbol;Acc:MGI:1349396]	Acot1	protein_coding
ENSMUSG00000072966	28.38206285	-1.180301155	0.775292779	0.002696221	0.036812084	Down	245607	G protein-coupled receptor associated sorting protein 2 [Source:MGI Symbol;Acc:MGI:2442071]	Gprasp2	protein_coding
ENSMUSG00000073062	110.9043583	0.436320892	0.178525304	0.001386869	0.02591718	Up	668166	zinc finger, X-linked, duplicated B [Source:MGI Symbol;Acc:MGI:3694898]	Zxdb	protein_coding
ENSMUSG00000073077	13.41721134	-1.476517012	0.740308129	0.001235104	0.024468519	Down	636104	cilia and flagella associated protein 47 [Source:MGI Symbol;Acc:MGI:3781475]	Cfap47	protein_coding
ENSMUSG00000073079	41.87926295	-1.070927019	0.311357572	2.64E-05	0.002956124	Down	24067	signal recognition particle 54A [Source:MGI Symbol;Acc:MGI:1346087]	Srp54a	protein_coding
ENSMUSG00000073418	25602.30423	-0.78822577	0.191083482	2.18E-06	0.000834238	Down	12268	complement component 4B (Chido blood group) [Source:MGI Symbol;Acc:MGI:88228]	C4b	protein_coding
ENSMUSG00000073490	869.7396252	0.589186257	0.30111185	0.002773397	0.03719373	Up	226691	interferon activated gene 207 [Source:MGI Symbol;Acc:MGI:2138302]	Ifi207	protein_coding
ENSMUSG00000073600	64.55300522	-1.442404081	0.637170682	0.000663702	0.017650586	Down	381148	proline rich basic protein 1 [Source:MGI Symbol;Acc:MGI:2686460]	Prob1	protein_coding
ENSMUSG00000073616	495.6547421	-0.655202483	0.291302842	0.001205969	0.024306965	Down	66915	COP9 signalosome subunit 9 [Source:MGI Symbol;Acc:MGI:1914165]	Cops9	protein_coding
ENSMUSG00000073650	28.04719459	-0.135405002	0.260289283	0.000961591	0.021465148	Down	241112	ciliogenesis associated TTC17 interacting protein [Source:MGI Symbol;Acc:MGI:2685062]	Catip	protein_coding
ENSMUSG00000073664	1334.878455	0.334586488	0.137303929	0.002281335	0.033800769	Up	269198	neurobeachin like 1 [Source:MGI Symbol;Acc:MGI:2444343]	Nbeal1	protein_coding
ENSMUSG00000073680	351.2553523	0.628023818	0.395223483	0.004287205	0.047461063	Up	320587	transmembrane protein 88B [Source:MGI Symbol;Acc:MGI:2444329]	Tmem88b	protein_coding
ENSMUSG00000073702	3619.818939	-0.37825234	0.147892283	0.001248329	0.024473786	Down	114641	ribosomal protein L31 [Source:MGI Symbol;Acc:MGI:2149632]	Rpl31	protein_coding
ENSMUSG00000074227	506.2338182	-1.293740673	0.26614063	5.39E-08	9.29E-05	Down	20733	serine protease inhibitor, Kunitz type 2 [Source:MGI Symbol;Acc:MGI:1338031]	Spint2	protein_coding
ENSMUSG00000074247	1215.72928	-0.312143216	0.117919437	0.001518097	0.027108458	Down	66498	DET1 and DDB1 associated 1 [Source:MGI Symbol;Acc:MGI:1913748]	Dda1	protein_coding
ENSMUSG00000074277	27.62876217	-0.649508454	0.377389582	0.003485583	0.042721947	Down	232970	pleckstrin homology like domain, family B, member 3 [Source:MGI Symbol;Acc:MGI:3642959]	Phldb3	protein_coding
ENSMUSG00000074457	1323.925935	-0.433322075	0.213639199	0.003479565	0.042717186	Down	67860	S100 calcium binding protein A16 [Source:MGI Symbol;Acc:MGI:1915110]	S100a16	protein_coding
ENSMUSG00000074578	180.0159001	-0.504955205	0.228166408	0.001842083	0.030090232	Down	68949	zinc finger, NFX1-type containing 1, antisense RNA 1 [Source:MGI Symbol;Acc:MGI:1916199]	Zfas1	lncRNA
ENSMUSG00000074698	1799.310467	0.219532215	0.048445698	2.29E-06	0.000834238	Up	12995	casein kinase 2, alpha 1 polypeptide [Source:MGI Symbol;Acc:MGI:88543]	Csnk2a1	protein_coding
ENSMUSG00000074738	135.5924145	-0.814015912	0.323182238	0.000501021	0.014809759	Down	230991	fibronectin type III domain containing 10 [Source:MGI Symbol;Acc:MGI:2444790]	Fndc10	protein_coding
ENSMUSG00000074884	1530.274067	-0.857111042	0.333255008	0.000422299	0.013526918	Down	378702	small EDRK-rich factor 2 [Source:MGI Symbol;Acc:MGI:1337041]	Serf2	protein_coding
ENSMUSG00000074994	640.6373461	0.464342903	0.180690565	0.000882683	0.020528033	Up	99003	glutamine and serine rich 1 [Source:MGI Symbol;Acc:MGI:2138986]	Qser1	protein_coding
ENSMUSG00000075225	52.77308304	-0.133235944	0.254979894	0.002001852	0.03146333	Down	75973	coiled-coil domain containing 162 [Source:MGI Symbol;Acc:MGI:1923223]	Ccdc162	protein_coding
ENSMUSG00000075324	112.8536916	1.155154951	0.286169124	2.54E-06	0.000864464	Up	60344	fidgetin [Source:MGI Symbol;Acc:MGI:1890647]	Fign	protein_coding
ENSMUSG00000075376	624.7534263	0.34177607	0.121394412	0.000727704	0.018535693	Up	319817	ring finger and CCCH-type zinc finger domains 2 [Source:MGI Symbol;Acc:MGI:2442789]	Rc3h2	protein_coding
ENSMUSG00000075467	678.2932968	-0.424948176	0.195859304	0.002671059	0.036638703	Down	52838	DNL-type zinc finger [Source:MGI Symbol;Acc:MGI:106559]	Dnlz	protein_coding
ENSMUSG00000075470	1341.011569	0.257167405	0.094588666	0.001779445	0.029514117	Up	380959	asparagine-linked glycosylation 10B (alpha-1,2-glucosyltransferase) [Source:MGI Symbol;Acc:MGI:2146159]	Alg10b	protein_coding
ENSMUSG00000075569	25.49914309	-0.141297589	0.265878283	0.003291532	0.041223093	Down	18861	radial spoke head 10 homolog B (Chlamydomonas) [Source:MGI Symbol;Acc:MGI:1922386]	Rsph10b	protein_coding
ENSMUSG00000075702	468.4515893	-0.597321299	0.211184629	0.000298996	0.010690053	Down	114679	selenoprotein M [Source:MGI Symbol;Acc:MGI:2149786]	Selenom	protein_coding
ENSMUSG00000076433	18.76092198	-0.990329207	0.381740254	0.000396516	0.012931806	Down	58251	CEP295 N-terminal like [Source:MGI Symbol;Acc:MGI:1929713]	Cep295nl	protein_coding
ENSMUSG00000076594	11.01529628	-0.003264648	0.168200489	5.69E-06	0.001281	Down	NA	immunoglobulin kappa variable 6-13 [Source:MGI Symbol;Acc:MGI:1330829]	Igkv6-13	IG_V_gene
ENSMUSG00000078185	482.9184324	0.550357672	0.172094651	0.000109587	0.006430038	Up	12663	choroideremia-like [Source:MGI Symbol;Acc:MGI:101913]	Chml	protein_coding
ENSMUSG00000078350	868.3941702	-0.609890652	0.20765733	0.000225463	0.009224534	Down	68859	small integral membrane protein 1 [Source:MGI Symbol;Acc:MGI:1916109]	Smim1	protein_coding
ENSMUSG00000078439	47.97516911	-1.849971557	0.526561941	1.64E-05	0.002373852	Down	72273	small integral membrane protein 24 [Source:MGI Symbol;Acc:MGI:1919523]	Smim24	protein_coding
ENSMUSG00000078451	33.24811166	-0.127962141	0.248064403	0.001403537	0.026007694	Down	73075	peptidylprolyl isomerase (cyclophilin)-like 6 [Source:MGI Symbol;Acc:MGI:1920325]	Ppil6	protein_coding
ENSMUSG00000078453	222.7305333	-0.498520452	0.237809474	0.002423021	0.034942197	Down	73112	ABRA C-terminal like [Source:MGI Symbol;Acc:MGI:1920362]	Abracl	protein_coding
ENSMUSG00000078580	64.90904172	-0.481060931	0.227984984	0.002542903	0.035772933	Down	101604	RIKEN cDNA E430018J23 gene [Source:MGI Symbol;Acc:MGI:2141981]	E430018J23Rik	protein_coding
ENSMUSG00000078713	430.3456715	-0.432245316	0.193070774	0.00205804	0.032096114	Down	68512	translocase of outer mitochondrial membrane 5 [Source:MGI Symbol;Acc:MGI:1915762]	Tomm5	protein_coding
ENSMUSG00000078952	11.12164783	1.00457946	0.678640815	0.003557546	0.043022595	Up	NA	long non-coding RNA, embryonic stem cells expressed 1 [Source:MGI Symbol;Acc:MGI:3780541]	Lncenc1	lncRNA
ENSMUSG00000079056	136.0406781	-0.592497369	0.301754299	0.002476128	0.03526538	Down	56461	Kv channel interacting protein 3, calsenilin [Source:MGI Symbol;Acc:MGI:1929258]	Kcnip3	protein_coding

ENSMUSG00000079235	7.615361314	-0.046405054	0.177588889	0.001359943	0.025674417	Down	100502861	coiled-coil domain containing 13 [Source:MGI Symbol;Acc:MGI:1920144]	Ccdc13	protein_coding
ENSMUSG00000079427	152.8809642	-0.473220685	0.208131995	0.001797142	0.029636503	Down	100039707	5, 10-methenyltetrahydrofolate synthetase-like [Source:MGI Symbol;Acc:MGI:3780550]	Mthfsl	protein_coding
ENSMUSG00000079478	226.1300248	-0.515728899	0.241302254	0.002113094	0.032569253	Down	56390	zinc ribbon domain containing 2 [Source:MGI Symbol;Acc:MGI:1913482]	Znrd2	protein_coding
ENSMUSG00000079480	116.8456461	-0.776299081	0.387665915	0.001690394	0.02875672	Down	69713	peptidyl-prolyl cis/trans isomerase, NIMA-interacting, 4 (parvulin) [Source:MGI Symbol;Acc:MGI:1916963]	Pin4	protein_coding
ENSMUSG00000079523	1437.099347	-0.895665314	0.536436187	0.002621025	0.036337993	Down	19240	thymosin, beta 10 [Source:MGI Symbol;Acc:MGI:109146]	Tmsb10	protein_coding
ENSMUSG00000079614	1192.043828	0.308781344	0.094353026	0.000224834	0.009224534	Up	72124	SEH1-like (S. cerevisiae [Source:MGI Symbol;Acc:MGI:1919374]	Seh1l	protein_coding
ENSMUSG00000080268	329.5451554	-0.333183513	0.128250596	0.001504211	0.026977079	Down	107392	breast cancer metastasis-suppressor 1 [Source:MGI Symbol;Acc:MGI:2388804]	Brms1	protein_coding
ENSMUSG00000081058	146.5917255	-1.177376872	0.341384112	2.49E-05	0.002844843	Down	97114	H3 clustered histone 15 [Source:MGI Symbol;Acc:MGI:2448357]	H3c15	protein_coding
ENSMUSG00000083282	1318.957897	-0.351822127	0.128885225	0.000930054	0.021144612	Down	56464	cathepsin F [Source:MGI Symbol;Acc:MGI:1861434]	Ctsf	protein_coding
ENSMUSG00000083563	165.9520682	0.951799121	0.386400446	0.000537033	0.015450228	Up	NA	predicted gene 13340 [Source:MGI Symbol;Acc:MGI:3650227]	Gm13340	unprocessed_pseudogene
ENSMUSG00000084166	6.293067459	-2.242763396	0.747567859	0.000147979	0.00753397	Down	NA	predicted gene 6451 [Source:MGI Symbol;Acc:MGI:3648305]	Gm6451	processed_pseudogene
ENSMUSG00000084834	20.44440429	-0.177330091	0.350696004	0.001200091	0.024267616	Down	68306	RIKEN cDNA 4930565N06 gene [Source:MGI Symbol;Acc:MGI:1915556]	4930565N06Rik	lncRNA
ENSMUSG00000085042	29.80718872	-1.681037868	0.510626938	3.72E-05	0.003584747	Down	NA	abhydrolase domain containing 11, opposite strand [Source:MGI Symbol;Acc:MGI:1917062]	Abhd11os	transcribed_unitary_pseudogene
ENSMUSG00000085519	36.65218474	-1.93595124	0.70658837	0.000188655	0.00835522	Down	NA	predicted gene 13703 [Source:MGI Symbol;Acc:MGI:3651307]	Gm13703	lncRNA
ENSMUSG00000085566	163.1084878	0.368751264	0.180492755	0.004588642	0.049299293	Up	NA	RIKEN cDNA A730017L22 gene [Source:MGI Symbol;Acc:MGI:3584452]	A730017L22Rik	lncRNA
ENSMUSG00000085786	10.08220777	-0.191323169	0.380144134	0.003249406	0.040933485	Down	NA	predicted gene 15987 [Source:MGI Symbol;Acc:MGI:3801849]	Gm15987	lncRNA
ENSMUSG00000085793	390.2109479	0.318521917	0.128357625	0.002171891	0.03306378	Up	217708	lin-52 DREAM MuvB core complex component [Source:MGI Symbol;Acc:MGI:3045391]	Lin52	protein_coding
ENSMUSG00000086058	33.66482777	0.714584186	0.37558597	0.002321026	0.034099096	Up	100038761	unc-45 myosin chaperone B, opposite strand [Source:MGI Symbol;Acc:MGI:3705119]	Unc45bos	lncRNA
ENSMUSG00000086247	44.29113887	0.791203849	0.223526381	2.33E-05	0.002728029	Up	100504007	predicted gene 15787 [Source:MGI Symbol;Acc:MGI:3783229]	Gm15787	lncRNA
ENSMUSG00000086742	14.91343821	-1.574302124	0.604891206	0.000306843	0.010769517	Down	NA	predicted gene 16201 [Source:MGI Symbol;Acc:MGI:3802153]	Gm16201	lncRNA
ENSMUSG00000086841	144.1950466	-1.527155719	0.383356835	2.91E-06	0.000921546	Down	NA	RIKEN cDNA 2410006H16 gene [Source:MGI Symbol;Acc:MGI:1916471]	2410006H16Rik	lncRNA
ENSMUSG00000087006	63.10763124	-0.672475481	0.462238672	0.004680588	0.049882854	Down	620695	predicted gene 13889 [Source:MGI Symbol;Acc:MGI:3652053]	Gm13889	protein_coding
ENSMUSG00000087013	6.214856431	-0.115165806	0.229662954	0.00275427	0.037110507	Down	NA	RIKEN cDNA 2610027K06 gene [Source:MGI Symbol;Acc:MGI:1917159]	2610027K06Rik	lncRNA
ENSMUSG00000087150	29.80180261	-1.206698355	0.405648595	0.000121523	0.0068438	Down	NA	cDNA sequence BC064078 [Source:MGI Symbol;Acc:MGI:3040692]	BC064078	cessed_pseudogene
ENSMUSG00000087336	13.38591322	-1.249646232	0.546457473	0.000765692	0.019013207	Down	NA	predicted gene 15860 [Source:MGI Symbol;Acc:MGI:3801778]	Gm15860	lncRNA
ENSMUSG00000087370	478.1806976	0.312263884	0.102365043	0.000443922	0.013919129	Up	621976	transmembrane protein 170B [Source:MGI Symbol;Acc:MGI:3647046]	Tmem170b	protein_coding
ENSMUSG00000087445	94.14072229	0.956522748	0.240966491	3.64E-06	0.000946988	Up	NA	predicted gene 14286 [Source:MGI Symbol;Acc:MGI:3650190]	Gm14286	lncRNA
ENSMUSG00000087478	23.895938	0.725919805	0.333029832	0.001312614	0.025189625	Up	75060	RIKEN cDNA 4930506C21 gene [Source:MGI Symbol;Acc:MGI:1922310]	4930506C21Rik	lncRNA
ENSMUSG00000087500	52.42428158	0.537997992	0.205495973	0.000638945	0.017312795	Up	NA	predicted gene 12426 [Source:MGI Symbol;Acc:MGI:3650989]	Gm12426	lncRNA
ENSMUSG00000087651	16.78870216	-1.143833527	0.600424385	0.001682708	0.028711005	Down	69784	RIKEN cDNA 1500009L16 gene [Source:MGI Symbol;Acc:MGI:1917034]	1500009L16Rik	protein_coding
ENSMUSG00000089762	154.2712453	-0.783686864	0.342329538	0.00095719	0.021461264	Down	72500	immediate early response 5-like [Source:MGI Symbol;Acc:MGI:1919750]	Ier5l	protein_coding
ENSMUSG00000089783	571.8533997	0.581431115	0.361609817	0.004664581	0.049743023	Up	NA	predicted gene 454 [Source:MGI Symbol;Acc:MGI:2685300]	Gm454	cessed_pseudogene
ENSMUSG00000089875	90.77858345	0.418837377	0.160581223	0.000970466	0.021551591	Up	NA	ethanol decreased 2 [Source:MGI Symbol;Acc:MGI:1261417]	Etohd2	lncRNA
ENSMUSG00000090093	89.41258948	0.619448371	0.218367727	0.000292897	0.010537577	Up	100043761	predicted gene 14399 [Source:MGI Symbol;Acc:MGI:3650082]	Gm14399	protein_coding
ENSMUSG00000090100	252.3785178	0.451933492	0.177945445	0.001027934	0.022338446	Up	140810	tau tubulin kinase 2 [Source:MGI Symbol;Acc:MGI:2155779]	Ttbk2	protein_coding
ENSMUSG00000090336	6.701495954	-0.070343082	0.189861124	0.002400406	0.03473233	Down	403185	CFAP97 domain containing 2 [Source:MGI Symbol;Acc:MGI:2685952]	Cfap97d2	protein_coding
ENSMUSG00000090841	6879.35264	-0.479304477	0.227038273	0.002706495	0.036812084	Down	17904	myosin, light polypeptide 6, alkali, smooth muscle and non-muscle [Source:MGI Symbol;Acc:MGI:109318]	Myl6	protein_coding
ENSMUSG00000091243	1577.519223	-0.623479371	0.331388995	0.002549151	0.03583158	Down	73569	vestigial like family member 3 [Source:MGI Symbol;Acc:MGI:1920819]	Vgll3	protein_coding
ENSMUSG00000091498	404.2673716	0.492525742	0.232438723	0.002405107	0.034771146	Up	NA	mitochondrial pyruvate carrier 1, pseudogene [Source:MGI Symbol;Acc:MGI:3781628]	Mpc1-ps	processed_pseudogene
ENSMUSG00000091955	43.64247983	-0.878513232	0.425617834	0.001397502	0.025923738	Down	NA	predicted pseudogene 9844 [Source:MGI Symbol;Acc:MGI:3704288]	Gm9844	protein_coding
ENSMUSG00000092203	121.7081507	-0.550821135	0.201102066	0.000436354	0.013846661	Down	68763	RIKEN cDNA 1110038B12 gene [Source:MGI Symbol;Acc:MGI:1916013]	1110038B12Rik	lncRNA
ENSMUSG00000092981	200.812975	0.855369424	0.249315525	3.14E-05	0.003266187	Up	100628593	microRNA 5125 [Source:MGI Symbol;Acc:MGI:4950450]	Mir5125	miRNA
ENSMUSG00000093661	1018.789249	0.35572594	0.100292528	6.27E-05	0.004826458	Up	66892	eukaryotic translation initiation factor 4E member 3 [Source:MGI Symbol;Acc:MGI:1914142]	Eif4e3	protein_coding
ENSMUSG00000093674	5246.082403	-0.601573737	0.279312268	0.001641171	0.028305152	Down	67945	ribosomal protein L41 [Source:MGI Symbol;Acc:MGI:1915195]	Rpl41	protein_coding
ENSMUSG00000095079	257.7796702	-0.050108121	0.178955513	0.004289907	0.047461063	Down	NA	immunoglobulin heavy constant alpha [Source:MGI Symbol;Acc:MGI:96444]	Igha	IG_C_gene
ENSMUSG00000095442	63.53429538	-0.014775217	0.169126137	0.000109248	0.006430038	Down	NA	immunoglobulin heavy variable 1-4 [Source:MGI Symbol;Acc:MGI:4439618]	Ighv1-4	IG_V_gene
ENSMUSG00000095526	6.808431986	-1.193756968	0.649738225	0.002281639	0.033800769	Down	NA	predicted gene 10243 [Source:MGI Symbol;Acc:MGI:3704266]	Gm10243	processed_pseudogene
ENSMUSG00000095742	148.1644844	-1.33369188	0.457421078	0.000129028	0.007036523	Down	NA			protein_coding
ENSMUSG00000096210	1711.945771	-0.389702216	0.138097893	0.000572334	0.016152764	Down	14958	H1.0 linker histone [Source:MGI Symbol;Acc:MGI:95893]	H1f0	protein_coding
ENSMUSG00000096422	56.45642447	-0.025122827	0.170916811	0.001855941	0.030173051	Down	NA	immunoglobulin kappa variable 12-44 [Source:MGI Symbol;Acc:MGI:4439775]	Igkv12-44	IG_V_gene
ENSMUSG00000096472	180.9205322	-0.496245276	0.235619765	0.002556981	0.035883103	Down	12581	cyclin dependent kinase inhibitor 2D [Source:MGI Symbol;Acc:MGI:105387]	Cdkn2d	protein_coding
ENSMUSG00000096544	17.78582784	-0.739364142	0.439045739	0.003227086	0.040682063	Down	NA	predicted pseudogene 4617 [Source:MGI Symbol;Acc:MGI:3704225]	Gm4617	processed_pseudogene

ENSMUSG00000097075	21.59887875	-1.26893104	0.67069303	0.001537	0.02725013	Down	381922	CDIP transferase, opposite strand [Source:MGI Symbol;Acc:MGI:2443610]	Cdiptos	protein_coding
ENSMUSG00000097124	1320.777038	0.454907071	0.238584623	0.004306294	0.047601259	Up	NA	RIKEN cDNA A530020G20 gene [Source:MGI Symbol;Acc:MGI:2442825]	A530020G20Rik	lncRNA
ENSMUSG00000097339	68.28985552	0.491651099	0.259202815	0.003742202	0.044598452	Up	NA	predicted gene, 26671 [Source:MGI Symbol;Acc:MGI:5477165]	Gm26671	lncRNA
ENSMUSG00000097392	160.9532763	0.502842118	0.17980041	0.000429565	0.013683362	Up	100042165	THO complex subunit 2-like [Source:MGI Symbol;Acc:MGI:3040669]	Thoc2l	protein_coding
ENSMUSG00000098188	2238.413763	0.56012687	0.182380248	0.000158115	0.007675767	Up	268301	sosondowah ankyrin repeat domain family member C [Source:MGI Symbol;Acc:MGI:3606051]	Sowahc	protein_coding
ENSMUSG00000099083	1431.313605	0.445514523	0.182646404	0.001388359	0.02591718	Up	223922	activating transcription factor 7 [Source:MGI Symbol;Acc:MGI:2443472]	Atf7	protein_coding
ENSMUSG00000099719	191.7375107	-1.249769956	0.488350641	0.000355164	0.011906857	Down	NA	predicted gene 28802 [Source:MGI Symbol;Acc:MGI:5579508]	Gm28802	unprocessed_pseudogene
ENSMUSG00000100131	652.3909746	0.807213644	0.298975519	0.000324457	0.011138189	Up	NA	predicted gene 28439 [Source:MGI Symbol;Acc:MGI:5579145]	Gm28439	unprocessed_pseudogene
ENSMUSG00000100774	14.35017973	1.389576969	0.590683813	0.00059083	0.016475355	Up	NA	predicted gene 7329 [Source:MGI Symbol;Acc:MGI:3646271]	Gm7329	processed_pseudogene
ENSMUSG00000100862	1521.107309	0.852275409	0.248803003	3.24E-05	0.003305157	Up	NA	predicted gene 10925 [Source:MGI Symbol;Acc:MGI:3809095]	Gm10925	unprocessed_pseudogene
ENSMUSG00000100954	28.66614978	-1.35007272	0.430769413	7.23E-05	0.005101093	Down	NA	predicted gene 10138 [Source:MGI Symbol;Acc:MGI:3708522]	Gm10138	lncRNA
ENSMUSG00000101111	984.9907015	0.69881563	0.311594952	0.001141163	0.023636606	Up	NA	predicted gene 28437 [Source:MGI Symbol;Acc:MGI:5579143]	Gm28437	unprocessed_pseudogene
ENSMUSG00000101249	915.1458117	0.691670315	0.23362383	0.000179957	0.008185824	Up	NA	predicted gene 29216 [Source:MGI Symbol;Acc:MGI:5579922]	Gm29216	unprocessed_pseudogene
ENSMUSG00000101335	27.76161665	0.748524742	0.377461273	0.001893277	0.030378816	Up	NA	predicted gene 28229 [Source:MGI Symbol;Acc:MGI:5578935]	Gm28229	lncRNA
ENSMUSG00000102018	42.59304191	-0.837266635	0.401450462	0.00136012	0.025674417	Down	432964	IQ motif and ankyrin repeat containing 1 [Source:MGI Symbol;Acc:MGI:3588184]	Iqank1	protein_coding
ENSMUSG00000102059	188.5797237	0.552539983	0.187821167	0.000238937	0.009465746	Up	NA	predicted gene, 20257 [Source:MGI Symbol;Acc:MGI:5012442]	Gm20257	cessed_pseudogene
ENSMUSG00000102752	249.4113657	-0.638671112	0.370708322	0.003394251	0.042142025	Down	665574	predicted gene 7694 [Source:MGI Symbol;Acc:MGI:3649135]	Gm7694	protein_coding
ENSMUSG00000103041	149.4390736	0.620948122	0.199750873	0.000124181	0.006874031	Up	NA	predicted gene, 37305 [Source:MGI Symbol;Acc:MGI:5610533]	Gm37305	lncRNA
ENSMUSG00000103149	69.67529741	2.058013356	0.512180178	2.32E-06	0.000834238	Up	NA	EST AA914427 [Source:MGI Symbol;Acc:MGI:1858439]	AA914427	TEC
ENSMUSG00000103313	12.47151825	-1.172937918	0.76909561	0.003096922	0.039386907	Down	NA	predicted gene, 38357 [Source:MGI Symbol;Acc:MGI:5611585]	Gm38357	TEC
ENSMUSG00000103411	12.36142525	1.261097998	0.570561424	0.000884625	0.020545475	Up	NA	predicted gene, 18300 [Source:MGI Symbol;Acc:MGI:5010485]	Gm18300	processed_pseudogene
ENSMUSG00000103509	14.19249224	-1.599024626	0.589131194	0.000255339	0.009843978	Down	NA	predicted gene, 38372 [Source:MGI Symbol;Acc:MGI:5611600]	Gm38372	TEC
ENSMUSG00000104348	67.19665672	0.595096518	0.245069003	0.000892303	0.020651151	Up	NA	predicted gene, 37691 [Source:MGI Symbol;Acc:MGI:5610919]	Gm37691	TEC
ENSMUSG00000104399	57.33737229	0.568872506	0.296213196	0.002857626	0.037968751	Up	NA	predicted gene, 37963 [Source:MGI Symbol;Acc:MGI:5611191]	Gm37963	lncRNA
ENSMUSG00000104453	249.5057396	0.338812434	0.157468701	0.004396611	0.048144948	Up	NA	predicted gene, 37829 [Source:MGI Symbol;Acc:MGI:5611057]	Gm37829	TEC
ENSMUSG00000105107	5.335484191	-0.124773218	0.241009103	0.003525004	0.042960679	Down	NA	predicted gene 43412 [Source:MGI Symbol;Acc:MGI:5663549]	Gm43412	TEC
ENSMUSG00000106743	20.91170726	0.912810069	0.605838875	0.003402529	0.042214386	Up	NA	predicted gene 42847 [Source:MGI Symbol;Acc:MGI:5662984]	Gm42847	TEC
ENSMUSG00000106990	122.9604786	0.533615753	0.26966581	0.002790878	0.037370006	Up	NA	predicted gene 42547 [Source:MGI Symbol;Acc:MGI:5662684]	Gm42547	TEC
ENSMUSG00000107096	66.48986812	0.535165981	0.236761349	0.001558637	0.027492314	Up	NA	predicted gene 43597 [Source:MGI Symbol;Acc:MGI:5663734]	Gm43597	lncRNA
ENSMUSG00000107741	119.2360013	0.403478814	0.1902784	0.003355675	0.0417232	Up	NA	predicted gene 2011 [Source:MGI Symbol;Acc:MGI:3780180]	Gm2011	cessed_pseudogene
ENSMUSG00000107838	105.0661812	0.417181643	0.19767584	0.003210417	0.040531226	Up	NA	predicted gene 45769 [Source:MGI Symbol;Acc:MGI:5804884]	Gm45769	TEC
ENSMUSG00000108425	65.5092611	0.82701678	0.391283402	0.001329435	0.025315087	Up	NA	predicted gene 44706 [Source:MGI Symbol;Acc:MGI:5753282]	Gm44706	lncRNA
ENSMUSG00000108452	18.515091	-1.125502157	0.760179121	0.003026811	0.0388775	Down	NA	RIKEN cDNA 4930413G21 gene [Source:MGI Symbol;Acc:MGI:1921201]	4930413G21Rik	TEC
ENSMUSG00000108461	14.64246867	-0.882167302	0.681895958	0.004592969	0.04931504	Down	NA	expressed sequence AV356131 [Source:MGI Symbol;Acc:MGI:2142184]	AV356131	lncRNA
ENSMUSG00000108859	9.38023479	-1.387819719	0.681437754	0.001246641	0.024468519	Down	NA	predicted gene 44776 [Source:MGI Symbol;Acc:MGI:5753352]	Gm44776	processed_pseudogene
ENSMUSG00000109311	12.07422179	-0.103254629	0.217288292	0.001778258	0.029514117	Down	101521	expressed sequence AI314278 [Source:MGI Symbol;Acc:MGI:2141898]	AI314278	lncRNA
ENSMUSG00000109498	12.30329563	1.450867322	0.472178061	8.97E-05	0.005761676	Up	NA	predicted gene 45222 [Source:MGI Symbol;Acc:MGI:5753798]	Gm45222	TEC
ENSMUSG00000109559	46.12391526	0.646838723	0.319343722	0.002002859	0.03146333	Up	NA	predicted gene, 34280 [Source:MGI Symbol;Acc:MGI:5593439]	Gm34280	lncRNA
ENSMUSG00000109572	26.77721601	-1.082150046	0.489161579	0.000895289	0.020653966	Down	100862066	cilia and flagella associated protein 99 [Source:MGI Symbol;Acc:MGI:5434801]	Cfap99	protein_coding
ENSMUSG00000109876	5.369629459	-0.268379317	1.66434331	0.001884178	0.030289224	Down	NA	predicted gene 45449 [Source:MGI Symbol;Acc:MGI:5791285]	Gm45449	lncRNA
ENSMUSG00000110332	32.04536756	-0.088345141	0.203437631	0.001640595	0.028305152	Down	NA	predicted gene, 19935 [Source:MGI Symbol;Acc:MGI:5012120]	Gm19935	protein_coding
ENSMUSG00000111840	84.746239	1.561285692	0.482525356	4.56E-05	0.00409291	Up	NA	predicted gene, 48832 [Source:MGI Symbol;Acc:MGI:6098557]	Gm48832	lncRNA
ENSMUSG00000112327	44.39915737	-0.141502367	0.265732981	0.003548349	0.043007234	Down	NA	predicted gene, 36827 [Source:MGI Symbol;Acc:MGI:5595986]	Gm36827	lncRNA
ENSMUSG00000112547	16.95637074	1.488216442	0.385746912	5.20E-06	0.001244765	Up	NA	predicted gene, 47096 [Source:MGI Symbol;Acc:MGI:6095829]	Gm47096	lncRNA
ENSMUSG00000112657	8.865517528	-0.05119856	0.179516397	0.002705808	0.036812084	Down	NA	cDNA sequence BC106175 [Source:MGI Symbol;Acc:MGI:3628444]	BC106175	transcribed_processed_pseudogene
ENSMUSG00000114025	70.53341594	0.644459916	0.290565052	0.001382405	0.02591718	Up	NA	predicted gene, 49331 [Source:MGI Symbol;Acc:MGI:6121516]	Gm49331	unprocessed_pseudogene
ENSMUSG00000116564	395.4312592	0.348139585	0.124487349	0.000792134	0.019445637	Up	67045	RIO kinase 2 [Source:MGI Symbol;Acc:MGI:1914295]	Riok2	protein_coding
ENSMUSG00000116908	37.86094932	-1.40788169	0.572437682	0.000449777	0.013990982	Down	NA	predicted gene, 49599 [Source:MGI Symbol;Acc:MGI:6215007]	Gm49599	TEC
