# Supplementary material for: The effect of aldafermin expressing-Escherichia coli Nissle 1917 along with dietary change on visceral adipose tissue in MASLD mouse model
Source: Int J Obes (Lond). 2025 Apr 10;49(7):1334–44. doi: 10.1038/s41366-025-01774-w (PMC12283412; doi:10.1038/s41366-025-01774-w)
Supplement: Supplementary file 3 — Supplementary table 4 [file 41366_2025_1774_MOESM3_ESM.pdf]

# Supplementary table 4. DEGs observed in eVAT when EcNA was compared to CTRL

**EcNA vs CTRL eVAT**

| ENSEMBL | baseMean | og2FoldChang | lfcSE | pvalue | padj | UD | entrezgene_id | description | external_gene_name | gene_biotype |
|---|---|---|---|---|---|---|---|---|---|---|
| ENSMUSG00000000149 | 1582.09149 | -0.3455472 | 0.1145752 | 0.0004614 | 0.00789467 | Down | 14673 | guanine nucleotide binding protein, alpha 12 [Source:MGI Symbol;Acc:MGI:95767] | Gna12 | protein_coding |
| ENSMUSG00000000154 | 204.816837 | -0.4367936 | 0.19224905 | 0.00276695 | 0.0256852 | Down | 18400 | solute carrier family 22 (organic cation transporter), member 18 [Source:MGI Symbol;Acc:MGI:1336884] | Slc22a18 | protein_coding |
| ENSMUSG00000000168 | 2696.69462 | 0.37012231 | 0.12110935 | 0.00047958 | 0.00814082 | Up | 235339 | dihydrolipoamide S-acetyltransferase (E2 component of pyruvate dehydrogenase complex) [Source:MGI Symbol;Acc:MGI:2385311] | Dlat | protein_coding |
| ENSMUSG00000000253 | 184.915793 | -0.5019699 | 0.26437158 | 0.00472789 | 0.036383 | Down | 66355 | guanosine monophosphate reductase [Source:MGI Symbol;Acc:MGI:1913605] | Gmpr | protein_coding |
| ENSMUSG00000000263 | 17.4678469 | -1.2105204 | 0.88389277 | 0.00363818 | 0.03060877 | Down | 14654 | glycine receptor, alpha 1 subunit [Source:MGI Symbol;Acc:MGI:95747] | Glra1 | protein_coding |
| ENSMUSG00000000275 | 1125.55507 | 0.3228694 | 0.13169575 | 0.00339281 | 0.02924946 | Up | 217069 | tripartite motif-containing 25 [Source:MGI Symbol;Acc:MGI:102749] | Trim25 | protein_coding |
| ENSMUSG00000000276 | 466.573201 | 0.37726945 | 0.15968649 | 0.00299728 | 0.02702045 | Up | 56077 | diacylglycerol kinase, epsilon [Source:MGI Symbol;Acc:MGI:1889276] | Dgke | protein_coding |
| ENSMUSG00000000374 | 1536.36999 | 0.29932462 | 0.10803547 | 0.00153578 | 0.01748044 | Up | 216131 | trafficking protein particle complex 10 [Source:MGI Symbol;Acc:MGI:1336209] | Trappc10 | protein_coding |
| ENSMUSG00000000378 | 879.075041 | -0.2933468 | 0.10515673 | 0.00149234 | 0.01723036 | Down | 216527 | cerebral cavernous malformation 2 [Source:MGI Symbol;Acc:MGI:2384924] | Ccm2 | protein_coding |
| ENSMUSG00000000579 | 74.174073 | -0.5413171 | 0.23157026 | 0.00169971 | 0.01864406 | Down | 100040563 | dynein light chain Tctex-type 1C [Source:MGI Symbol;Acc:MGI:3807476] | Dynlt1c | protein_coding |
| ENSMUSG00000000594 | 4258.03205 | -0.2310228 | 0.08593385 | 0.00344819 | 0.02951308 | Down | 14667 | GM2 ganglioside activator protein [Source:MGI Symbol;Acc:MGI:95762] | Gm2a | protein_coding |
| ENSMUSG00000000682 | 287.918929 | -0.7673897 | 0.62820514 | 0.00590306 | 0.04202246 | Down | 23833 | CD52 antigen [Source:MGI Symbol;Acc:MGI:1346088] | Cd52 | protein_coding |
| ENSMUSG00000000708 | 1822.71656 | 0.30056099 | 0.12143174 | 0.00357303 | 0.03024692 | Up | 18519 | K(lysine) acetyltransferase 2B [Source:MGI Symbol;Acc:MGI:1343094] | Kat2b | protein_coding |
| ENSMUSG00000000738 | 1677.55543 | -0.2972046 | 0.08919035 | 0.0002386 | 0.00528083 | Down | 234847 | SPG7, paraplegin matrix AAA peptidase subunit [Source:MGI Symbol;Acc:MGI:2385906] | Spg7 | protein_coding |
| ENSMUSG00000000740 | 7628.80707 | -0.7993954 | 0.20030732 | 4.36E-06 | 0.00037622 | Down | 270106 | ribosomal protein L13 [Source:MGI Symbol;Acc:MGI:105922] | Rpl13 | protein_coding |
| ENSMUSG00000000743 | 1689.19089 | -0.3080097 | 0.09803717 | 0.00044196 | 0.00769517 | Down | 234852 | charged multivesicular body protein 1A [Source:MGI Symbol;Acc:MGI:1920159] | Chmp1a | protein_coding |
| ENSMUSG00000000753 | 3377.51101 | -0.9809574 | 0.30469745 | 6.51E-05 | 0.00221232 | Down | 20317 | serine (or cysteine) peptidase inhibitor, clade F, member 1 [Source:MGI Symbol;Acc:MGI:108080] | Serpinf1 | protein_coding |
| ENSMUSG00000000787 | 4394.17562 | 0.28824536 | 0.09357252 | 0.00054959 | 0.00892046 | Up | 13205 | DEAD box helicase 3, X-linked [Source:MGI Symbol;Acc:MGI:103064] | Ddx3x | protein_coding |
| ENSMUSG00000001025 | 2967.8932 | -0.8875309 | 0.24869154 | 2.05E-05 | 0.00109543 | Down | 20200 | S100 calcium binding protein A6 (calcyclin) [Source:MGI Symbol;Acc:MGI:1339467] | S100a6 | protein_coding |
| ENSMUSG00000001052 | 1617.9432 | 0.48897396 | 0.17259586 | 0.00051897 | 0.00864686 | Up | 99683 | Sec24 related gene family, member B (S. cerevisiae) [Source:MGI Symbol;Acc:MGI:2139764] | Sec24b | protein_coding |
| ENSMUSG00000001056 | 452.462455 | -0.8158042 | 0.22240085 | 1.66E-05 | 0.00095687 | Down | 52530 | NHP2 ribonucleoprotein [Source:MGI Symbol;Acc:MGI:1098547] | Nhp2 | protein_coding |
| ENSMUSG00000001082 | 383.964744 | -0.2619757 | 0.11103402 | 0.00631543 | 0.04410755 | Down | 68294 | major facilitator superfamily domain containing 10 [Source:MGI Symbol;Acc:MGI:1915544] | Mfsd10 | protein_coding |
| ENSMUSG00000001105 | 629.80551 | -0.4571586 | 0.12131603 | 2.29E-05 | 0.00117239 | Down | 55978 | intraflagellar transport 20 [Source:MGI Symbol;Acc:MGI:1915585] | Ift20 | protein_coding |
| ENSMUSG00000001119 | 10095.5149 | -0.8672204 | 0.24500641 | 2.33E-05 | 0.00118724 | Down | 12833 | collagen, type VI, alpha 1 [Source:MGI Symbol;Acc:MGI:88459] | Col6a1 | protein_coding |
| ENSMUSG00000001131 | 75.0584083 | -1.0402439 | 0.35468989 | 0.00015086 | 0.00389592 | Down | 21857 | tissue inhibitor of metalloproteinase 1 [Source:MGI Symbol;Acc:MGI:98752] | Timp1 | protein_coding |
| ENSMUSG00000001156 | 251.761141 | 0.37336199 | 0.15486014 | 0.00269387 | 0.02525209 | Up | 17119 | MAX dimerization protein 1 [Source:MGI Symbol;Acc:MGI:96908] | Mxd1 | protein_coding |
| ENSMUSG00000001166 | 56.0021711 | -0.4213401 | 0.22742656 | 0.00713144 | 0.04801088 | Down | 114643 | 2'-5' oligoadenylate synthetase 1C [Source:MGI Symbol;Acc:MGI:2149633] | Oas1c | protein_coding |
| ENSMUSG00000001280 | 1454.92361 | 0.36284586 | 0.15954599 | 0.00400726 | 0.03264694 | Up | 20683 | trans-acting transcription factor 1 [Source:MGI Symbol;Acc:MGI:98372] | Sp1 | protein_coding |
| ENSMUSG00000001289 | 1382.83812 | -0.4235294 | 0.2019548 | 0.00436982 | 0.03446687 | Down | 56612 | prefoldin 5 [Source:MGI Symbol;Acc:MGI:1928753] | Pfdn5 | protein_coding |
| ENSMUSG00000001313 | 143.029009 | -0.8053432 | 0.24025956 | 4.98E-05 | 0.00187013 | Down | 11858 | Rho family GTPase 2 [Source:MGI Symbol;Acc:MGI:1338755] | Rnd2 | protein_coding |
| ENSMUSG00000001333 | 60.0275572 | -0.7976978 | 0.32564661 | 0.00072452 | 0.01090021 | Down | 68828 | syncoilin [Source:MGI Symbol;Acc:MGI:1916078] | Sync | protein_coding |
| ENSMUSG00000001383 | 846.300779 | -0.5136053 | 0.15213982 | 8.07E-05 | 0.00254484 | Down | 66492 | zinc finger, matrin type 2 [Source:MGI Symbol;Acc:MGI:1913742] | Zmat2 | protein_coding |
| ENSMUSG00000001418 | 1472.09344 | -0.2735933 | 0.10300486 | 0.00249715 | 0.02401966 | Down | 56700 | glycosylated lysosomal membrane protein [Source:MGI Symbol;Acc:MGI:1913318] | Glmp | protein_coding |
| ENSMUSG00000001435 | 6009.17886 | -0.7698359 | 0.22355986 | 3.71E-05 | 0.00157811 | Down | 12822 | collagen, type XVIII, alpha 1 [Source:MGI Symbol;Acc:MGI:88451] | Col18a1 | protein_coding |
| ENSMUSG00000001506 | 7171.21934 | -0.5958898 | 0.22902324 | 0.00068835 | 0.01048989 | Down | 12842 | collagen, type I, alpha 1 [Source:MGI Symbol;Acc:MGI:88467] | Col1a1 | protein_coding |
| ENSMUSG00000001524 | 263.997544 | -0.3455686 | 0.14561897 | 0.00353805 | 0.03009197 | Down | 14885 | general transcription factor II H, polypeptide 4 [Source:MGI Symbol;Acc:MGI:1338799] | Gtf2h4 | protein_coding |
| ENSMUSG00000001552 | 5243.24415 | -0.4000869 | 0.1250995 | 0.00023591 | 0.00524985 | Down | 16480 | junction plakoglobin [Source:MGI Symbol;Acc:MGI:96650] | Jup | protein_coding |
| ENSMUSG00000001642 | 2497.0358 | -0.3215085 | 0.14690027 | 0.00642138 | 0.04461736 | Down | 11677 | aldo-keto reductase family 1, member B3 (aldose reductase) [Source:MGI Symbol;Acc:MGI:1353494] | Akr1b3 | protein_coding |
| ENSMUSG00000001707 | 248.276293 | -0.4017444 | 0.17784666 | 0.00340967 | 0.02932366 | Down | 66143 | eukaryotic translation elongation factor 1 epsilon 1 [Source:MGI Symbol;Acc:MGI:1913393] | Eef1e1 | protein_coding |
| ENSMUSG00000001751 | 988.532984 | -0.3416288 | 0.10175411 | 0.00017363 | 0.00426713 | Down | 27419 | alpha-N-acetylglucosaminidase (Sanfilippo disease IIIB) [Source:MGI Symbol;Acc:MGI:1351641] | Naglu | protein_coding |
| ENSMUSG00000001768 | 1533.18618 | 0.28899699 | 0.11080759 | 0.00261167 | 0.02470975 | Up | 408057 | Ras and Rab interactor 2 [Source:MGI Symbol;Acc:MGI:1921280] | Rin2 | protein_coding |
| ENSMUSG00000001783 | 1732.5647 | -0.242751 | 0.0903685 | 0.00236927 | 0.02320131 | Down | 28088 | RNA 2',3'-cyclic phosphate and 5'-OH ligase [Source:MGI Symbol;Acc:MGI:106379] | Rtcb | protein_coding |
| ENSMUSG00000001794 | 7233.44431 | -0.3488562 | 0.12251738 | 0.00124249 | 0.01524486 | Down | 12336 | calpain, small subunit 1 [Source:MGI Symbol;Acc:MGI:88266] | Capns1 | protein_coding |
| ENSMUSG00000001999 | 626.49435 | -0.5434275 | 0.24226651 | 0.00193044 | 0.02029777 | Down | 109778 | biliverdin reductase A [Source:MGI Symbol;Acc:MGI:88170] | Blvra | protein_coding |
| ENSMUSG00000002014 | 1232.96561 | -0.4540803 | 0.12891915 | 5.74E-05 | 0.00202121 | Down | 20832 | signal sequence receptor, delta [Source:MGI Symbol;Acc:MGI:1099464] | Ssr4 | protein_coding |
| ENSMUSG00000002043 | 301.758848 | -0.9934738 | 0.20452108 | 7.17E-08 | 2.20E-05 | Down | 67091 | trafficking protein particle complex 6A [Source:MGI Symbol;Acc:MGI:1914341] | Trappc6a | protein_coding |
| ENSMUSG00000002064 | 593.384077 | -0.365754 | 0.18268742 | 0.00693002 | 0.04714328 | Down | 20316 | stromal cell derived factor 2 [Source:MGI Symbol;Acc:MGI:108019] | Sdf2 | protein_coding |
| ENSMUSG00000002083 | 269.950217 | -0.6704573 | 0.394282 | 0.00407926 | 0.03296898 | Down | 170770 | BCL2 binding component 3 [Source:MGI Symbol;Acc:MGI:2181667] | Bbc3 | protein_coding |
| ENSMUSG00000002102 | 1995.81928 | -0.397157 | 0.1568971 | 0.00172515 | 0.01881773 | Down | 19182 | proteasome (prosome, macropain) 26S subunit, ATPase 3 [Source:MGI Symbol;Acc:MGI:1098754] | Psmc3 | protein_coding |
| ENSMUSG00000002105 | 817.168033 | -0.3171799 | 0.11539464 | 0.00143434 | 0.01677936 | Down | 68427 | solute carrier family 39 (metal ion transporter), member 13 [Source:MGI Symbol;Acc:MGI:1915677] | Slc39a13 | protein_coding |
| ENSMUSG00000002109 | 120.703775 | 0.39008418 | 0.16952489 | 0.00329663 | 0.02878681 | Up | 107986 | damage specific DNA binding protein 2 [Source:MGI Symbol;Acc:MGI:1355314] | Ddb2 | protein_coding |

| | | | | | | | | | | |
|---|---|---|---|---|---|---|---|---|---|---|
| ENSMUSG00000002222 | 2339.63898 | 0.20747104 | 0.06946549 | 0.00143237 | 0.01677936 | Up | 68477 | required for meiotic nuclear division 5 homolog A [Source:MGI Symbol;Acc:MGI:1915727] | Rmnd5a | protein_coding |
| ENSMUSG00000002233 | 1581.47539 | -0.8664152 | 0.23117554 | 1.12E-05 | 0.00074835 | Down | 11853 | ras homolog family member C [Source:MGI Symbol;Acc:MGI:106028] | Rhoc | protein_coding |
| ENSMUSG00000002274 | 29.9321473 | -1.7946888 | 0.70839473 | 0.000352 | 0.00677512 | Down | 70083 | meteorin, glial cell differentiation regulator [Source:MGI Symbol;Acc:MGI:1917333] | Metrn | protein_coding |
| ENSMUSG00000002289 | 11762.857 | -0.6035031 | 0.15629445 | 1.03E-05 | 0.00071093 | Down | 57875 | angiopoietin-like 4 [Source:MGI Symbol;Acc:MGI:1888999] | Angptl4 | protein_coding |
| ENSMUSG00000002307 | 907.517659 | -0.4211643 | 0.17349554 | 0.00192612 | 0.0202654 | Down | 13163 | Fas death domain-associated protein [Source:MGI Symbol;Acc:MGI:1197015] | Daxx | protein_coding |
| ENSMUSG00000002308 | 999.329464 | -0.3852172 | 0.18127266 | 0.0048659 | 0.03712226 | Down | 54219 | CD320 antigen [Source:MGI Symbol;Acc:MGI:1860083] | Cd320 | protein_coding |
| ENSMUSG00000002319 | 675.605228 | -0.3503429 | 0.120004 | 0.00071685 | 0.01086301 | Down | 75751 | importin 4 [Source:MGI Symbol;Acc:MGI:1923001] | Ipo4 | protein_coding |
| ENSMUSG00000002329 | 602.38179 | -0.4635272 | 0.16987405 | 0.00080413 | 0.01163158 | Down | 67881 | magnesium-dependent phosphatase 1 [Source:MGI Symbol;Acc:MGI:1915131] | Mdp1 | protein_coding |
| ENSMUSG00000002332 | 821.894176 | -0.4131351 | 0.13613701 | 0.0003401 | 0.00663257 | Down | 52585 | dehydrogenase/reductase (SDR family) member 1 [Source:MGI Symbol;Acc:MGI:1196314] | Dhrs1 | protein_coding |
| ENSMUSG00000002343 | 207.010725 | -0.5471271 | 0.15893683 | 5.71E-05 | 0.00201842 | Down | 76813 | armadillo repeat containing 6 [Source:MGI Symbol;Acc:MGI:1924063] | Armc6 | protein_coding |
| ENSMUSG00000002379 | 890.286664 | -0.7029568 | 0.28013897 | 0.0007358 | 0.01099993 | Down | 69875 | NADH:ubiquinone oxidoreductase subunit A11 [Source:MGI Symbol;Acc:MGI:1917125] | Ndufa11 | protein_coding |
| ENSMUSG00000002393 | 428.058825 | -0.6743047 | 0.23898115 | 0.00030671 | 0.00617693 | Down | 13864 | nuclear receptor subfamily 2, group F, member 6 [Source:MGI Symbol;Acc:MGI:1352453] | Nr2f6 | protein_coding |
| ENSMUSG00000002395 | 1105.14161 | -0.3692317 | 0.1571403 | 0.00319667 | 0.02819979 | Down | 67023 | unconventional SNARE in the ER 1 homolog (S. cerevisiae) [Source:MGI Symbol;Acc:MGI:1914273] | Use1 | protein_coding |
| ENSMUSG00000002413 | 780.568078 | 0.32294702 | 0.15215121 | 0.00724385 | 0.04852633 | Up | 109880 | Braf transforming gene [Source:MGI Symbol;Acc:MGI:88190] | Braf | protein_coding |
| ENSMUSG00000002524 | 1559.60641 | -0.3136401 | 0.09707498 | 0.00031978 | 0.00636881 | Down | 67959 | poly-U binding splicing factor 60 [Source:MGI Symbol;Acc:MGI:1915209] | Puf60 | protein_coding |
| ENSMUSG00000002580 | 235.655204 | -1.0783057 | 0.39818261 | 0.00027686 | 0.00577363 | Down | 103742 | migration and invasion enhancer 1 [Source:MGI Symbol;Acc:MGI:1913678] | Mien1 | protein_coding |
| ENSMUSG00000002658 | 571.367428 | -0.5962944 | 0.21058108 | 0.00038035 | 0.00710202 | Down | 98053 | general transcription factor IIF, polypeptide 1 [Source:MGI Symbol;Acc:MGI:1923848] | Gtf2f1 | protein_coding |
| ENSMUSG00000002661 | 117.283375 | -0.4383717 | 0.20301861 | 0.00356298 | 0.03019333 | Down | 66400 | alkB homolog 7 [Source:MGI Symbol;Acc:MGI:1913650] | Alkbh7 | protein_coding |
| ENSMUSG00000002718 | 960.642564 | 0.29228804 | 0.0997162 | 0.00105275 | 0.01373732 | Up | 110750 | chromosome segregation 1-like (S. cerevisiae) [Source:MGI Symbol;Acc:MGI:1339951] | Cse1l | protein_coding |
| ENSMUSG00000002732 | 569.527091 | -0.4128014 | 0.15197048 | 0.00096721 | 0.01312646 | Down | 14231 | FK506 binding protein 7 [Source:MGI Symbol;Acc:MGI:1336879] | Fkbp7 | protein_coding |
| ENSMUSG00000002767 | 651.049199 | -0.4714157 | 0.17205237 | 0.00072381 | 0.01090021 | Down | 27398 | mitochondrial ribosomal protein L2 [Source:MGI Symbol;Acc:MGI:1351622] | Mrpl2 | protein_coding |
| ENSMUSG00000002768 | 493.150274 | -0.5158602 | 0.25572006 | 0.00351253 | 0.02996906 | Down | 17256 | male enhanced antigen 1 [Source:MGI Symbol;Acc:MGI:96957] | Mea1 | protein_coding |
| ENSMUSG00000002804 | 126.083124 | -0.6197694 | 0.19207524 | 0.00010392 | 0.00298801 | Down | 66174 | nudix (nucleoside diphosphate linked moiety X)-type motif 14 [Source:MGI Symbol;Acc:MGI:1913424] | Nudt14 | protein_coding |
| ENSMUSG00000002808 | 517.540702 | -0.5849919 | 0.18277146 | 0.00012117 | 0.00333059 | Down | 105298 | ependymin related protein 1 (zebrafish) [Source:MGI Symbol;Acc:MGI:2145369] | Epdr1 | protein_coding |
| ENSMUSG00000002885 | 1845.35111 | -0.3430662 | 0.13757744 | 0.00238818 | 0.02330085 | Down | 26364 | adhesion G protein-coupled receptor E5 [Source:MGI Symbol;Acc:MGI:1347095] | Adgre5 | protein_coding |
| ENSMUSG00000002963 | 476.200881 | -0.3772593 | 0.12424789 | 0.00044162 | 0.00769517 | Down | 59047 | polynucleotide kinase 3'- phosphatase [Source:MGI Symbol;Acc:MGI:1891698] | Pnkp | protein_coding |
| ENSMUSG00000002980 | 1669.91175 | -0.3577036 | 0.1595674 | 0.00402982 | 0.03274858 | Down | 57278 | basal cell adhesion molecule [Source:MGI Symbol;Acc:MGI:1929940] | Bcam | protein_coding |
| ENSMUSG00000002983 | 425.971617 | -0.4447808 | 0.12014128 | 3.44E-05 | 0.00149515 | Down | 19698 | avian reticuloendotheliosis viral (v-rel) oncogene related B [Source:MGI Symbol;Acc:MGI:103289] | Relb | protein_coding |
| ENSMUSG00000002985 | 66291.1668 | -0.7214533 | 0.22602807 | 9.41E-05 | 0.00280835 | Down | 11816 | apolipoprotein E [Source:MGI Symbol;Acc:MGI:88057] | Apoe | protein_coding |
| ENSMUSG00000003037 | 950.431525 | -0.2238119 | 0.09151359 | 0.00635517 | 0.04430891 | Down | 17274 | RAB8A, member RAS oncogene family [Source:MGI Symbol;Acc:MGI:96960] | Rab8a | protein_coding |
| ENSMUSG00000003039 | 1273.43077 | -0.2463021 | 0.10331967 | 0.00537609 | 0.03959432 | Down | 67922 | family with sequence similarity 32, member A [Source:MGI Symbol;Acc:MGI:1915172] | Fam32a | protein_coding |
| ENSMUSG00000003062 | 411.009405 | -0.4413855 | 0.15534073 | 0.00058772 | 0.00939442 | Down | 76205 | STARD3 N-terminal like [Source:MGI Symbol;Acc:MGI:1923455] | Stard3nl | protein_coding |
| ENSMUSG00000003068 | 1983.73945 | -0.4065184 | 0.13896428 | 0.00054317 | 0.0088592 | Down | 20869 | serine/threonine kinase 11 [Source:MGI Symbol;Acc:MGI:1341870] | Stk11 | protein_coding |
| ENSMUSG00000003199 | 818.284666 | -0.4169477 | 0.13378336 | 0.00031902 | 0.00636668 | Down | 68047 | MPN domain containing [Source:MGI Symbol;Acc:MGI:1915297] | Mpnd | protein_coding |
| ENSMUSG00000003269 | 662.579896 | -0.3386771 | 0.15963872 | 0.00654827 | 0.04515136 | Down | 19158 | cytohesin 2 [Source:MGI Symbol;Acc:MGI:1334255] | Cyth2 | protein_coding |
| ENSMUSG00000003299 | 1021.40299 | -0.3519428 | 0.13574016 | 0.00190434 | 0.02010138 | Down | 66163 | mitochondrial ribosomal protein L4 [Source:MGI Symbol;Acc:MGI:2137210] | Mrpl4 | protein_coding |
| ENSMUSG00000003345 | 2273.65546 | -0.5621793 | 0.08882943 | 2.27E-11 | 4.27E-08 | Down | 103236 | casein kinase 1, gamma 2 [Source:MGI Symbol;Acc:MGI:1920014] | Csnk1g2 | protein_coding |
| ENSMUSG00000003346 | 1387.39258 | -0.7830557 | 0.1650829 | 1.48E-07 | 3.81E-05 | Down | 216169 | abhydrolase domain containing 17A [Source:MGI Symbol;Acc:MGI:106388] | Abhd17a | protein_coding |
| ENSMUSG00000003363 | 1249.39162 | -0.6046469 | 0.12781747 | 2.33E-07 | 5.41E-05 | Down | 18807 | phospholipase D family, member 3 [Source:MGI Symbol;Acc:MGI:1333782] | Pld3 | protein_coding |
| ENSMUSG00000003380 | 3362.01694 | -0.8311256 | 0.13638085 | 8.98E-11 | 1.46E-07 | Down | 14470 | Rab acceptor 1 (prenylated) [Source:MGI Symbol;Acc:MGI:1201692] | Rabac1 | protein_coding |
| ENSMUSG00000003420 | 5590.38672 | -0.3226429 | 0.13620968 | 0.00352788 | 0.03004887 | Down | 14132 | Fc fragment of IgG receptor and transporter [Source:MGI Symbol;Acc:MGI:103017] | Fcgrt | protein_coding |
| ENSMUSG00000003421 | 662.426697 | -0.3356732 | 0.12442562 | 0.00123352 | 0.01521525 | Down | 66394 | nitric oxide synthase interacting protein [Source:MGI Symbol;Acc:MGI:1913644] | Nosip | protein_coding |
| ENSMUSG00000003423 | 471.345077 | -0.3634717 | 0.13753306 | 0.00143469 | 0.01677936 | Down | 68845 | PIH1 domain containing 1 [Source:MGI Symbol;Acc:MGI:1916095] | Pih1d1 | protein_coding |
| ENSMUSG00000003429 | 3111.0603 | -0.370057 | 0.13374788 | 0.00105048 | 0.01373732 | Down | 27207 | ribosomal protein S11 [Source:MGI Symbol;Acc:MGI:1351329] | Rps11 | protein_coding |
| ENSMUSG00000003444 | 195.904074 | -0.4287191 | 0.14968002 | 0.0006004 | 0.00951179 | Down | 67224 | mediator complex subunit 29 [Source:MGI Symbol;Acc:MGI:1914474] | Med29 | protein_coding |
| ENSMUSG00000003518 | 2924.64837 | -0.3844445 | 0.15178647 | 0.00181715 | 0.01951063 | Down | 72349 | dual specificity phosphatase 3 (vaccinia virus phosphatase VH1-related) [Source:MGI Symbol;Acc:MGI:1919599] | Dusp3 | protein_coding |
| ENSMUSG00000003559 | 199.824086 | -0.4096517 | 0.20791444 | 0.00638373 | 0.04443173 | Down | 57344 | arsenite methyltransferase [Source:MGI Symbol;Acc:MGI:1929882] | As3mt | protein_coding |
| ENSMUSG00000003585 | 19.5461257 | -0.2028232 | 0.37786085 | 0.00603862 | 0.0426696 | Down | 67815 | SEC14-like lipid binding 2 [Source:MGI Symbol;Acc:MGI:1915065] | Sec14l2 | protein_coding |
| ENSMUSG00000003617 | 3848.93414 | 0.66030649 | 0.24066281 | 0.00042484 | 0.00751546 | Up | 12870 | ceruloplasmin [Source:MGI Symbol;Acc:MGI:88476] | Cp | protein_coding |
| ENSMUSG00000003809 | 819.611313 | -0.2789274 | 0.11921752 | 0.00573653 | 0.04134421 | Down | 270076 | glutaryl-Coenzyme A dehydrogenase [Source:MGI Symbol;Acc:MGI:104541] | Gcdh | protein_coding |
| ENSMUSG00000003863 | 125.484486 | -0.6905395 | 0.22860658 | 0.00018857 | 0.00452448 | Down | 76787 | protein tyrosine phosphatase, receptor type, f polypeptide (PTPRF), interacting protein (liprin), alpha 3 [Source:MGI Symbol;Acc:MGI:1924037] | Ppfia3 | protein_coding |
| ENSMUSG00000003873 | 368.627261 | -0.7725414 | 0.22967057 | 4.97E-05 | 0.00187013 | Down | 12028 | BCL2-associated X protein [Source:MGI Symbol;Acc:MGI:99702] | Bax | protein_coding |
| ENSMUSG00000003949 | 263.595234 | 1.01102618 | 0.30822861 | 5.10E-05 | 0.00189042 | Up | 217082 | hepatic leukemia factor [Source:MGI Symbol;Acc:MGI:96108] | Hlf | protein_coding |

| | | | | | | | | | | |
|---|---|---|---|---|---|---|---|---|---|---|
| ENSMUSG00000003955 | 566.355152 | -0.427746 | 0.14514644 | 0.00045644 | 0.00785873 | Down | 70186 | family with sequence similarity 162, member A [Source:MGI Symbol;Acc:MGI:1917436] | Fam162a | protein_coding |
| ENSMUSG00000003970 | 6465.67535 | -0.5910922 | 0.1797489 | 9.40E-05 | 0.00280835 | Down | 26961 | ribosomal protein L8 [Source:MGI Symbol;Acc:MGI:1350927] | Rpl8 | protein_coding |
| ENSMUSG00000004044 | 33565.0238 | -0.469457 | 0.15158804 | 0.00023796 | 0.00527371 | Down | 19285 | caveolae associated 1 [Source:MGI Symbol;Acc:MGI:1277968] | Cavin1 | protein_coding |
| ENSMUSG00000004110 | 258.432435 | -1.6869607 | 0.4667662 | 1.23E-05 | 0.00078933 | Down | 12290 | calcium channel, voltage-dependent, R type, alpha 1E subunit [Source:MGI Symbol;Acc:MGI:106217] | Cacna1e | protein_coding |
| ENSMUSG00000004285 | 827.327776 | -0.5824633 | 0.15146712 | 1.16E-05 | 0.00075871 | Down | 66144 | ATPase, H+ transporting, lysosomal V1 subunit F [Source:MGI Symbol;Acc:MGI:1913394] | Atp6v1f | protein_coding |
| ENSMUSG00000004360 | 390.272917 | 0.7033852 | 0.29568726 | 0.00101534 | 0.01342082 | Up | 212448 | RIKEN cDNA 9330159F19 gene [Source:MGI Symbol;Acc:MGI:3036239] | 9330159F19Rik | protein_coding |
| ENSMUSG00000004364 | 2175.80126 | 0.23156238 | 0.09004737 | 0.00421433 | 0.0337749 | Up | 26554 | cullin 3 [Source:MGI Symbol;Acc:MGI:1347360] | Cul3 | protein_coding |
| ENSMUSG00000004383 | 862.178525 | -0.5563108 | 0.18320824 | 0.00023333 | 0.00520675 | Down | 16795 | LARGE xylosyl- and glucuronyltransferase 1 [Source:MGI Symbol;Acc:MGI:1342270] | Large1 | protein_coding |
| ENSMUSG00000004393 | 651.285751 | -0.3227081 | 0.12668888 | 0.00254531 | 0.02429407 | Down | 52513 | DEAD box helicase 56 [Source:MGI Symbol;Acc:MGI:1277172] | Ddx56 | protein_coding |
| ENSMUSG00000004394 | 1500.24761 | -0.2800718 | 0.08630736 | 0.00034446 | 0.00667501 | Down | 103694 | transmembrane p24 trafficking protein 4 [Source:MGI Symbol;Acc:MGI:1915070] | Tmed4 | protein_coding |
| ENSMUSG00000004558 | 5153.73354 | -0.3602925 | 0.17161354 | 0.00600113 | 0.04254817 | Down | 29811 | N-myc downstream regulated gene 2 [Source:MGI Symbol;Acc:MGI:1352498] | Ndrg2 | protein_coding |
| ENSMUSG00000004610 | 464.853618 | -1.0505541 | 0.33792222 | 8.61E-05 | 0.00264502 | Down | 110826 | electron transferring flavoprotein, beta polypeptide [Source:MGI Symbol;Acc:MGI:106098] | Etfb | protein_coding |
| ENSMUSG00000004665 | 925.52444 | -1.0389386 | 0.16123484 | 7.26E-12 | 1.96E-08 | Down | 12798 | calponin 2 [Source:MGI Symbol;Acc:MGI:105093] | Cnn2 | protein_coding |
| ENSMUSG00000004667 | 684.892415 | -0.514125 | 0.18392124 | 0.00052516 | 0.00873207 | Down | 66420 | polymerase (RNA) II (DNA directed) polypeptide E [Source:MGI Symbol;Acc:MGI:1913670] | Polr2e | protein_coding |
| ENSMUSG00000004814 | 316.804031 | -0.7220941 | 0.22907456 | 0.00010758 | 0.00305533 | Down | 56221 | chemokine (C-C motif) ligand 24 [Source:MGI Symbol;Acc:MGI:1928953] | Ccl24 | protein_coding |
| ENSMUSG00000004846 | 2724.0418 | -0.47481 | 0.14941701 | 0.00017031 | 0.00421008 | Down | 26433 | procollagen-lysine, 2-oxoglutarate 5-dioxygenase 3 [Source:MGI Symbol;Acc:MGI:1347008] | Plod3 | protein_coding |
| ENSMUSG00000004849 | 1099.77078 | -0.5642355 | 0.20383207 | 0.00049886 | 0.00837668 | Down | 11769 | adaptor protein complex AP-1, sigma 1 [Source:MGI Symbol;Acc:MGI:1098244] | Ap1s1 | protein_coding |
| ENSMUSG00000004865 | 812.766252 | 0.27027488 | 0.10375091 | 0.00298537 | 0.02697293 | Up | 20815 | serine/arginine-rich protein specific kinase 1 [Source:MGI Symbol;Acc:MGI:106908] | Srpk1 | protein_coding |
| ENSMUSG00000004896 | 305.620746 | 0.35025189 | 0.12747064 | 0.00123903 | 0.01523671 | Up | 229503 | ribosomal RNA adenine dimethylase domain containing 1 [Source:MGI Symbol;Acc:MGI:2387197] | Rrnad1 | protein_coding |
| ENSMUSG00000004929 | 420.128618 | -0.4983617 | 0.12470882 | 8.10E-06 | 0.00059029 | Down | 50492 | thimet oligopeptidase 1 [Source:MGI Symbol;Acc:MGI:1354165] | Thop1 | protein_coding |
| ENSMUSG00000004931 | 580.333416 | -0.2840683 | 0.12875263 | 0.00755768 | 0.04988808 | Down | 57267 | amyloid beta (A4) precursor protein-binding, family A, member 3 [Source:MGI Symbol;Acc:MGI:1888527] | Apba3 | protein_coding |
| ENSMUSG00000004951 | 2389.22076 | -0.7708158 | 0.20722497 | 1.58E-05 | 0.00092534 | Down | 15507 | heat shock protein 1 [Source:MGI Symbol;Acc:MGI:96240] | Hspb1 | protein_coding |
| ENSMUSG00000004996 | 448.429187 | -0.3217743 | 0.13957668 | 0.00444184 | 0.03482517 | Down | 67873 | methylthioribose-1-phosphate isomerase 1 [Source:MGI Symbol;Acc:MGI:1915123] | Mri1 | protein_coding |
| ENSMUSG00000005034 | 1498.53033 | 0.31739402 | 0.10466021 | 0.00061697 | 0.00966009 | Up | 18749 | protein kinase, cAMP dependent, catalytic, beta [Source:MGI Symbol;Acc:MGI:97594] | Prkacb | protein_coding |
| ENSMUSG00000005054 | 1013.99933 | -0.5883116 | 0.22740656 | 0.0007606 | 0.01122243 | Down | 13014 | cystatin B [Source:MGI Symbol;Acc:MGI:109514] | Cstb | protein_coding |
| ENSMUSG00000005161 | 2731.69597 | -0.4645836 | 0.16378833 | 0.00054188 | 0.008856 | Down | 21672 | peroxiredoxin 2 [Source:MGI Symbol;Acc:MGI:109486] | Prdx2 | protein_coding |
| ENSMUSG00000005225 | 220.512252 | 0.34463903 | 0.14996453 | 0.00437154 | 0.03446687 | Up | 231999 | pleckstrin homology domain containing, family A (phosphoinositide binding specific) member 8 [Source:MGI Symbol;Acc:MGI:2681164] | Plekha8 | protein_coding |
| ENSMUSG00000005233 | 49.5657844 | -1.193917 | 0.66574721 | 0.00195257 | 0.02046808 | Down | 66442 | SPC25, NDC80 kinetochore complex component, homolog (S. cerevisiae) [Source:MGI Symbol;Acc:MGI:1913692] | Spc25 | protein_coding |
| ENSMUSG00000005371 | 1159.33274 | 0.36165087 | 0.09666731 | 4.04E-05 | 0.00164666 | Up | 225055 | F-box protein 11 [Source:MGI Symbol;Acc:MGI:2147134] | Fbxo11 | protein_coding |
| ENSMUSG00000005373 | 1823.7262 | 0.58554955 | 0.22495508 | 0.00063987 | 0.00991866 | Up | 58805 | MLX interacting protein-like [Source:MGI Symbol;Acc:MGI:1927999] | Mlxipl | protein_coding |
| ENSMUSG00000005506 | 1980.49827 | 0.33868557 | 0.12335924 | 0.00134227 | 0.01614199 | Up | 13046 | CUGBP, Elav-like family member 1 [Source:MGI Symbol;Acc:MGI:1342295] | Celf1 | protein_coding |
| ENSMUSG00000005682 | 505.358427 | 0.40882616 | 0.16011324 | 0.0016164 | 0.01802229 | Up | 103135 | PAN2 poly(A) specific ribonuclease subunit [Source:MGI Symbol;Acc:MGI:1918984] | Pan2 | protein_coding |
| ENSMUSG00000005683 | 9318.13171 | 0.38102581 | 0.12678516 | 0.00061024 | 0.00960103 | Up | 12974 | citrate synthase [Source:MGI Symbol;Acc:MGI:88529] | Cs | protein_coding |
| ENSMUSG00000005698 | 956.059529 | 0.33874072 | 0.08153796 | 7.78E-06 | 0.00057186 | Up | 13018 | CCCTC-binding factor [Source:MGI Symbol;Acc:MGI:109447] | Ctcf | protein_coding |
| ENSMUSG00000005699 | 117.890468 | -0.3657063 | 0.17079803 | 0.00518301 | 0.0386052 | Down | 56513 | par-6 family cell polarity regulator alpha [Source:MGI Symbol;Acc:MGI:1927223] | Pard6a | protein_coding |
| ENSMUSG00000005716 | 7.69577917 | -0.1734153 | 0.34040122 | 3.39E-05 | 0.00149515 | Down | 19293 | parvalbumin [Source:MGI Symbol;Acc:MGI:97821] | Pvalb | protein_coding |
| ENSMUSG00000005779 | 2173.31484 | -0.3636932 | 0.1560829 | 0.00391628 | 0.03216375 | Down | 19172 | proteasome (prosome, macropain) subunit, beta type 4 [Source:MGI Symbol;Acc:MGI:1098257] | Psmb4 | protein_coding |
| ENSMUSG00000005802 | 614.176166 | 0.339584 | 0.12145986 | 0.00114302 | 0.01448198 | Up | 22785 | solute carrier family 30 (zinc transporter), member 4 [Source:MGI Symbol;Acc:MGI:1345282] | Slc30a4 | protein_coding |
| ENSMUSG00000005873 | 9254.82744 | -0.3908311 | 0.14134974 | 0.00119051 | 0.01486541 | Down | 13476 | receptor accessory protein 5 [Source:MGI Symbol;Acc:MGI:1270152] | Reep5 | protein_coding |
| ENSMUSG00000005881 | 1846.53109 | -0.2528463 | 0.09127179 | 0.00201314 | 0.02088341 | Down | 66366 | ERGIC and golgi 3 [Source:MGI Symbol;Acc:MGI:1913616] | Ergic3 | protein_coding |
| ENSMUSG00000005897 | 334.737966 | 0.30662336 | 0.09803292 | 0.0004886 | 0.00825092 | Up | 22025 | nuclear receptor subfamily 2, group C, member 1 [Source:MGI Symbol;Acc:MGI:1352465] | Nr2c1 | protein_coding |
| ENSMUSG00000005899 | 636.303197 | 0.2244064 | 0.07957298 | 0.00205987 | 0.02117888 | Up | 77626 | sphingomyelin phosphodiesterase 4 [Source:MGI Symbol;Acc:MGI:1924876] | Smpd4 | protein_coding |
| ENSMUSG00000005907 | 423.589781 | 0.31810603 | 0.11753361 | 0.00169735 | 0.01863067 | Up | 71382 | peroxisomal biogenesis factor 1 [Source:MGI Symbol;Acc:MGI:1918632] | Pex1 | protein_coding |
| ENSMUSG00000005947 | 128.012744 | 0.53376439 | 0.21779878 | 0.00122588 | 0.0151441 | Up | 16407 | integrin alpha E, epithelial-associated [Source:MGI Symbol;Acc:MGI:1298377] | Itgae | protein_coding |
| ENSMUSG00000005949 | 384.033022 | 0.34362269 | 0.13019094 | 0.00183207 | 0.01959309 | Up | 83429 | cystinosis, nephropathic [Source:MGI Symbol;Acc:MGI:1932872] | Ctns | protein_coding |
| ENSMUSG00000005958 | 113.936829 | -0.6369382 | 0.44606386 | 0.00630748 | 0.04410755 | Down | 13845 | Eph receptor B3 [Source:MGI Symbol;Acc:MGI:104770] | Ephb3 | protein_coding |
| ENSMUSG00000005982 | 886.241279 | 0.28162749 | 0.098023 | 0.00125246 | 0.01530269 | Up | 74763 | N(alpha)-acetyltransferase 60, NatF catalytic subunit [Source:MGI Symbol;Acc:MGI:1922013] | Naa60 | protein_coding |
| ENSMUSG00000006095 | 765.734936 | -0.6204426 | 0.14908766 | 2.75E-06 | 0.00028616 | Down | 66411 | tubulin folding cofactor B [Source:MGI Symbol;Acc:MGI:1913661] | Tbcb | protein_coding |
| ENSMUSG00000006205 | 6368.43205 | -0.7101865 | 0.15761471 | 5.18E-07 | 9.05E-05 | Down | 56213 | HtrA serine peptidase 1 [Source:MGI Symbol;Acc:MGI:1929076] | Htra1 | protein_coding |
| ENSMUSG00000006221 | 1298.19778 | -1.4105887 | 0.80215879 | 0.00183037 | 0.01958787 | Down | 29818 | heat shock protein family, member 7 (cardiovascular) [Source:MGI Symbol;Acc:MGI:1352494] | Hspb7 | protein_coding |
| ENSMUSG00000006262 | 841.662208 | 0.27743436 | 0.09060195 | 0.00072467 | 0.01090021 | Up | 68473 | MOB kinase activator 1B [Source:MGI Symbol;Acc:MGI:1915723] | Mob1b | protein_coding |
| ENSMUSG00000006299 | 2362.88262 | -0.4758583 | 0.19972593 | 0.00180367 | 0.01940435 | Down | 227290 | angio-associated migratory protein [Source:MGI Symbol;Acc:MGI:107809] | Aamp | protein_coding |
| ENSMUSG00000006304 | 3109.28256 | -0.3703437 | 0.11435982 | 0.00019599 | 0.00460768 | Down | 76709 | actin related protein 2/3 complex, subunit 2 [Source:MGI Symbol;Acc:MGI:1923959] | Arpc2 | protein_coding |
| ENSMUSG00000006315 | 820.989967 | -0.3521874 | 0.12267985 | 0.00085182 | 0.01206436 | Down | 69804 | transmembrane protein 147 [Source:MGI Symbol;Acc:MGI:1915011] | Tmem147 | protein_coding |
| ENSMUSG00000006333 | 6805.56675 | -0.7921178 | 0.21063049 | 1.12E-05 | 0.00074835 | Down | 76846 | ribosomal protein S9 [Source:MGI Symbol;Acc:MGI:1924096] | Rps9 | protein_coding |

| | | | | | | | | | | |
|---|---|---|---|---|---|---|---|---|---|---|
| ENSMUSG00000006335 | 180.890812 | -0.7809435 | 0.2731394 | 0.00025575 | 0.00549552 | Down | 69714 | TCF3 (E2A) fusion partner [Source:MGI Symbol;Acc:MGI:1916964] | Tfpt | protein_coding |
| ENSMUSG00000006356 | 1492.15214 | -0.9170003 | 0.22611412 | 2.92E-06 | 0.00029825 | Down | 68337 | cysteine rich protein 2 [Source:MGI Symbol;Acc:MGI:1915587] | Crip2 | protein_coding |
| ENSMUSG00000006360 | 2404.66275 | -1.605251 | 0.32729572 | 4.21E-08 | 1.51E-05 | Down | 12925 | cysteine-rich protein 1 (intestinal) [Source:MGI Symbol;Acc:MGI:88501] | Crip1 | protein_coding |
| ENSMUSG00000006378 | 55.5455538 | -0.5179028 | 0.27269767 | 0.00444579 | 0.0348257 | Down | 26912 | glycine C-acetyltransferase (2-amino-3-ketobutyrate-coenzyme A ligase) [Source:MGI Symbol;Acc:MGI:1349389] | Gcat | protein_coding |
| ENSMUSG00000006390 | 881.997699 | -0.2434269 | 0.10381832 | 0.00711239 | 0.0479133 | Down | 54325 | elongation of very long chain fatty acids (FEN1/Elo2, SUR4/Elo3, yeast)-like 1 [Source:MGI Symbol;Acc:MGI:1858959] | Elovl1 | protein_coding |
| ENSMUSG00000006395 | 334.795544 | -0.7491776 | 0.14029016 | 7.49E-09 | 5.07E-06 | Down | NA | hydroxypyruvate isomerase (putative) [Source:MGI Symbol;Acc:MGI:1915430] | Hyi | protein_coding |
| ENSMUSG00000006442 | 816.10012 | -0.4119295 | 0.13941581 | 0.0004741 | 0.00805782 | Down | 20810 | spermidine synthase [Source:MGI Symbol;Acc:MGI:102690] | Srm | protein_coding |
| ENSMUSG00000006494 | 987.984899 | 0.85751096 | 0.19723008 | 8.01E-07 | 0.0001251 | Up | 228026 | pyruvate dehydrogenase kinase, isoenzyme 1 [Source:MGI Symbol;Acc:MGI:1926119] | Pdk1 | protein_coding |
| ENSMUSG00000006519 | 747.508243 | -0.7721388 | 0.26681382 | 0.00022103 | 0.00500777 | Down | 13057 | cytochrome b-245, alpha polypeptide [Source:MGI Symbol;Acc:MGI:1316658] | Cyba | protein_coding |
| ENSMUSG00000006527 | 530.840359 | 0.24550238 | 0.08746038 | 0.00207069 | 0.02122294 | Up | 54650 | Scm-like with four mbt domains 1 [Source:MGI Symbol;Acc:MGI:1859609] | Sfmbt1 | protein_coding |
| ENSMUSG00000006542 | 265.736875 | -0.7858836 | 0.37881572 | 0.00180337 | 0.01940435 | Down | 241113 | protein kinase, AMP-activated, gamma 3 non-catalytic subunit [Source:MGI Symbol;Acc:MGI:1891343] | Prkag3 | protein_coding |
| ENSMUSG00000006641 | 836.529605 | 0.79651971 | 0.41210087 | 0.00225943 | 0.02245945 | Up | 330064 | solute carrier family 5 (sodium-dependent vitamin transporter), member 6 [Source:MGI Symbol;Acc:MGI:2660847] | Slc5a6 | protein_coding |
| ENSMUSG00000006715 | 59.2478325 | -0.5998372 | 0.32898448 | 0.00401606 | 0.03268583 | Down | 57441 | geminin [Source:MGI Symbol;Acc:MGI:1927344] | Gmnn | protein_coding |
| ENSMUSG00000006728 | 867.821809 | -0.2973904 | 0.12125798 | 0.00404068 | 0.03280398 | Down | 12567 | cyclin-dependent kinase 4 [Source:MGI Symbol;Acc:MGI:88357] | Cdk4 | protein_coding |
| ENSMUSG00000006764 | 132.159079 | -0.1430807 | 0.28804286 | 2.97E-06 | 0.00030199 | Down | 216343 | tryptophan hydroxylase 2 [Source:MGI Symbol;Acc:MGI:2651811] | Tph2 | protein_coding |
| ENSMUSG00000007036 | 1704.05764 | -0.222968 | 0.08603393 | 0.00424706 | 0.03388677 | Down | 193742 | abhydrolase domain containing 16A [Source:MGI Symbol;Acc:MGI:99476] | Abhd16a | protein_coding |
| ENSMUSG00000007039 | 448.760502 | -0.5142322 | 0.14889645 | 6.32E-05 | 0.00215991 | Down | 51793 | dimethylarginine dimethylaminohydrolase 2 [Source:MGI Symbol;Acc:MGI:1859016] | Ddah2 | protein_coding |
| ENSMUSG00000007041 | 1003.88128 | -0.3957955 | 0.19317719 | 0.00546166 | 0.0400925 | Down | 114584 | chloride intracellular channel 1 [Source:MGI Symbol;Acc:MGI:2148924] | Clic1 | protein_coding |
| ENSMUSG00000007107 | 49.70435 | -2.5194161 | 0.66111546 | 5.15E-06 | 0.00042911 | Down | 27222 | ATPase, Na+/K+ transporting, alpha 4 polypeptide [Source:MGI Symbol;Acc:MGI:1351335] | Atp1a4 | protein_coding |
| ENSMUSG00000007122 | 24.6437602 | -1.7001163 | 0.81654692 | 0.00099627 | 0.01330743 | Down | 12372 | calsequestrin 1 [Source:MGI Symbol;Acc:MGI:1309468] | Casq1 | protein_coding |
| ENSMUSG00000007415 | 1706.11728 | -0.3692909 | 0.12661933 | 0.00054518 | 0.00888303 | Down | 67210 | GATA zinc finger domain containing 1 [Source:MGI Symbol;Acc:MGI:1914460] | Gatad1 | protein_coding |
| ENSMUSG00000007613 | 762.851185 | 0.42346026 | 0.13203755 | 0.00020665 | 0.00475491 | Up | 21812 | transforming growth factor, beta receptor I [Source:MGI Symbol;Acc:MGI:98728] | Tgfbr1 | protein_coding |
| ENSMUSG00000007617 | 218.620288 | 0.39450014 | 0.19273428 | 0.00563187 | 0.04076902 | Up | 26556 | homer scaffolding protein 1 [Source:MGI Symbol;Acc:MGI:1347345] | Homer1 | protein_coding |
| ENSMUSG00000007721 | 551.131494 | -0.8972283 | 0.2723087 | 5.29E-05 | 0.00193072 | Down | 234388 | coiled-coil domain containing 124 [Source:MGI Symbol;Acc:MGI:1916403] | Ccdc124 | protein_coding |
| ENSMUSG00000007836 | 2654.93548 | -0.7630773 | 0.2068734 | 1.51E-05 | 0.00090128 | Down | 77134 | heterogeneous nuclear ribonucleoprotein A0 [Source:MGI Symbol;Acc:MGI:1924384] | Hnrnpa0 | protein_coding |
| ENSMUSG00000007837 | 345.585262 | -0.3723636 | 0.17933941 | 0.00613718 | 0.04319693 | Down | 65116 | proline-rich Gla (G-carboxyglutamic acid) polypeptide 2 [Source:MGI Symbol;Acc:MGI:1929596] | Prrg2 | protein_coding |
| ENSMUSG00000007867 | 213.186655 | -0.5242172 | 0.1663987 | 0.0001724 | 0.00424338 | Down | 76411 | intraflagellar transport 43 [Source:MGI Symbol;Acc:MGI:1923661] | Ift43 | protein_coding |
| ENSMUSG00000007891 | 19904.7767 | -0.4939039 | 0.24313204 | 0.0036873 | 0.03089471 | Down | 13033 | cathepsin D [Source:MGI Symbol;Acc:MGI:88562] | Ctsd | protein_coding |
| ENSMUSG00000007892 | 11159.2846 | -0.9071014 | 0.20166654 | 4.10E-07 | 7.65E-05 | Down | 56040 | ribosomal protein, large, P1 [Source:MGI Symbol;Acc:MGI:1927099] | Rplp1 | protein_coding |
| ENSMUSG00000007987 | 311.983182 | -0.5155716 | 0.14235791 | 3.44E-05 | 0.00149515 | Down | 67286 | intraflagellar transport 22 [Source:MGI Symbol;Acc:MGI:1914536] | Ift22 | protein_coding |
| ENSMUSG00000008036 | 1139.99942 | -0.54039 | 0.2128129 | 0.00114533 | 0.01448198 | Down | 232910 | adaptor-related protein complex 2, sigma 1 subunit [Source:MGI Symbol;Acc:MGI:2141861] | Ap2s1 | protein_coding |
| ENSMUSG00000008090 | 5840.59654 | -0.2977877 | 0.12131661 | 0.00376374 | 0.03130672 | Down | 116701 | fibroblast growth factor receptor-like 1 [Source:MGI Symbol;Acc:MGI:2150920] | Fgfrl1 | protein_coding |
| ENSMUSG00000008140 | 1939.16026 | -0.3845402 | 0.12251597 | 0.0002962 | 0.00608313 | Down | 69683 | ER membrane protein complex subunit 10 [Source:MGI Symbol;Acc:MGI:1916933] | Emc10 | protein_coding |
| ENSMUSG00000008200 | 814.15443 | 0.5245119 | 0.12021481 | 1.49E-06 | 0.00018768 | Up | 55935 | formin binding protein 4 [Source:MGI Symbol;Acc:MGI:1860513] | Fnbp4 | protein_coding |
| ENSMUSG00000008307 | 46.0012868 | 0.78403701 | 0.38101194 | 0.00183474 | 0.01960874 | Up | 77036 | RIKEN cDNA 1700109H08 gene [Source:MGI Symbol;Acc:MGI:1924286] | 1700109H08Rik | protein_coding |
| ENSMUSG00000008668 | 4988.4058 | -0.5722653 | 0.16585275 | 5.27E-05 | 0.00193072 | Down | 20084 | ribosomal protein S18 [Source:MGI Symbol;Acc:MGI:98146] | Rps18 | protein_coding |
| ENSMUSG00000008690 | 1481.17049 | -0.2813585 | 0.07763434 | 9.89E-05 | 0.002905 | Down | 52683 | non-SMC condensin II complex, subunit H2 [Source:MGI Symbol;Acc:MGI:1289164] | Ncaph2 | protein_coding |
| ENSMUSG00000008822 | 148.231897 | -1.2061359 | 0.23726863 | 1.98E-08 | 9.20E-06 | Down | 66204 | acylphosphatase 1, erythrocyte (common) type [Source:MGI Symbol;Acc:MGI:1913454] | Acyp1 | protein_coding |
| ENSMUSG00000008958 | 416.766121 | -0.3896269 | 0.18964489 | 0.00560795 | 0.04067018 | Down | 21427 | vacuolar protein sorting 72 [Source:MGI Symbol;Acc:MGI:1202305] | Vps72 | protein_coding |
| ENSMUSG00000009013 | 1083.08908 | -0.5834724 | 0.17340125 | 7.31E-05 | 0.00237067 | Down | 56455 | dynein light chain LC8-type 1 [Source:MGI Symbol;Acc:MGI:1861457] | Dynll1 | protein_coding |
| ENSMUSG00000009076 | 161.641113 | -0.8275126 | 0.22984066 | 2.02E-05 | 0.00109259 | Down | 67178 | zinc finger, matrin type 5 [Source:MGI Symbol;Acc:MGI:1914428] | Zmat5 | protein_coding |
| ENSMUSG00000009114 | 8.11631478 | 0.04067733 | 0.20424426 | 0.00742666 | 0.04940461 | Up | 76964 | RIKEN cDNA 2610028H24 gene [Source:MGI Symbol;Acc:MGI:1924214] | 2610028H24Rik | protein_coding |
| ENSMUSG00000009281 | 4472.09587 | -0.9884203 | 0.19193383 | 2.16E-08 | 9.39E-06 | Down | 71660 | retinoic acid receptor responder (tazarotene induced) 2 [Source:MGI Symbol;Acc:MGI:1918910] | Rarres2 | protein_coding |
| ENSMUSG00000009293 | 1117.09334 | -0.2559013 | 0.09940782 | 0.00348293 | 0.0297634 | Down | 22213 | ubiquitin-conjugating enzyme E2G 2 [Source:MGI Symbol;Acc:MGI:1343188] | Ube2g2 | protein_coding |
| ENSMUSG00000009630 | 1124.37656 | -0.4896209 | 0.14022587 | 5.56E-05 | 0.00199124 | Down | 19053 | protein phosphatase 2 (formerly 2A), catalytic subunit, beta isoform [Source:MGI Symbol;Acc:MGI:1321161] | Ppp2cb | protein_coding |
| ENSMUSG00000009687 | 732.368458 | -0.4153959 | 0.19457997 | 0.00419327 | 0.0336513 | Down | 18301 | FXYD domain-containing ion transport regulator 5 [Source:MGI Symbol;Acc:MGI:1201785] | Fxyd5 | protein_coding |
| ENSMUSG00000009741 | 1123.32847 | 0.2986387 | 0.10205498 | 0.00097304 | 0.01318349 | Up | 22221 | upstream binding protein 1 [Source:MGI Symbol;Acc:MGI:104889] | Ubp1 | protein_coding |
| ENSMUSG00000009894 | 696.469484 | -0.2333979 | 0.09587885 | 0.00581774 | 0.04168913 | Down | 67826 | synaptosomal-associated protein, 47 [Source:MGI Symbol;Acc:MGI:1915076] | Snap47 | protein_coding |
| ENSMUSG00000010095 | 2281.38875 | -0.4596899 | 0.20070583 | 0.0023724 | 0.02321049 | Down | 17254 | solute carrier family 3 (activators of dibasic and neutral amino acid transport), member 2 [Source:MGI Symbol;Acc:MGI:96955] | Slc3a2 | protein_coding |
| ENSMUSG00000010362 | 402.681669 | -0.4362462 | 0.18424217 | 0.00219512 | 0.02206662 | Down | 66599 | RAD52 motif 1 [Source:MGI Symbol;Acc:MGI:1913849] | Rdm1 | protein_coding |

| | | | | | | | | | | |
|---|---|---|---|---|---|---|---|---|---|---|
| ENSMUSG00000010376 | 1183.13855 | -0.7869957 | 0.22785626 | 3.42E-05 | 0.00149515 | Down | 18002 | neural precursor cell expressed, developmentally down-regulated gene 8 [Source:MGI Symbol;Acc:MGI:97301] | Nedd8 | protein_coding |
| ENSMUSG00000010392 | 1081.54434 | 0.23567588 | 0.0942627 | 0.00497707 | 0.03772865 | Up | 53334 | golgi SNAP receptor complex member 1 [Source:MGI Symbol;Acc:MGI:1858260] | Gosr1 | protein_coding |
| ENSMUSG00000010607 | 296.699234 | -0.6845761 | 0.23052137 | 0.00020843 | 0.00477778 | Down | 66268 | phosphatidylinositol glycan anchor biosynthesis, class Y-like [Source:MGI Symbol;Acc:MGI:1913518] | Pigyl | protein_coding |
| ENSMUSG00000010608 | 1740.37229 | 0.36124326 | 0.11665811 | 0.00041708 | 0.00743736 | Up | 67039 | RNA binding motif protein 25 [Source:MGI Symbol;Acc:MGI:1914289] | Rbm25 | protein_coding |
| ENSMUSG00000010609 | 972.645893 | -0.3628972 | 0.15933097 | 0.00380135 | 0.03149054 | Down | 19165 | presenilin 2 [Source:MGI Symbol;Acc:MGI:109284] | Psen2 | protein_coding |
| ENSMUSG00000010651 | 1409.17131 | -0.7868583 | 0.23923444 | 6.23E-05 | 0.00214587 | Down | 235674 | acetyl-Coenzyme A acyltransferase 1B [Source:MGI Symbol;Acc:MGI:3605455] | Acaa1b | protein_coding |
| ENSMUSG00000010797 | 10.7161335 | -2.0919623 | 0.66665522 | 7.65E-05 | 0.00245632 | Down | 22413 | wingless-type MMTV integration site family, member 2 [Source:MGI Symbol;Acc:MGI:98954] | Wnt2 | protein_coding |
| ENSMUSG00000011096 | 1215.01876 | -0.4528106 | 0.14430056 | 0.00020629 | 0.00475336 | Down | 67605 | AKT1 substrate 1 (proline-rich) [Source:MGI Symbol;Acc:MGI:1914855] | Akt1s1 | protein_coding |
| ENSMUSG00000011179 | 1874.51852 | -0.2646932 | 0.11059752 | 0.00498804 | 0.03777653 | Down | 18263 | ornithine decarboxylase, structural 1 [Source:MGI Symbol;Acc:MGI:97402] | Odc1 | protein_coding |
| ENSMUSG00000011382 | 2803.98927 | 0.27125804 | 0.11825533 | 0.00678189 | 0.04636861 | Up | 71755 | dihydrodiol dehydrogenase (dimeric) [Source:MGI Symbol;Acc:MGI:1919005] | Dhdh | protein_coding |
| ENSMUSG00000011658 | 214.876202 | -0.3896804 | 0.16013781 | 0.00235896 | 0.02317822 | Down | 70300 | fuzzy planar cell polarity protein [Source:MGI Symbol;Acc:MGI:1917550] | Fuz | protein_coding |
| ENSMUSG00000011884 | 2106.64922 | -0.4802087 | 0.13417467 | 3.96E-05 | 0.0016295 | Down | 56356 | glycolipid transfer protein [Source:MGI Symbol;Acc:MGI:1929253] | Gltp | protein_coding |
| ENSMUSG00000011960 | 713.498269 | 0.39909824 | 0.10339673 | 2.05E-05 | 0.00109543 | Up | 12455 | cyclin T1 [Source:MGI Symbol;Acc:MGI:1328363] | Ccnt1 | protein_coding |
| ENSMUSG00000012405 | 3069.44325 | -0.5627699 | 0.16889464 | 7.95E-05 | 0.00251852 | Down | 66480 | ribosomal protein L15 [Source:MGI Symbol;Acc:MGI:1913730] | Rpl15 | protein_coding |
| ENSMUSG00000012640 | 334.689404 | 0.44891558 | 0.15111296 | 0.0003943 | 0.00722135 | Up | 69930 | zinc finger protein 715 [Source:MGI Symbol;Acc:MGI:1917180] | Zfp715 | protein_coding |
| ENSMUSG00000013160 | 2705.47724 | -0.301383 | 0.13322141 | 0.00577979 | 0.04150871 | Down | 11972 | ATPase, H+ transporting, lysosomal V0 subunit D1 [Source:MGI Symbol;Acc:MGI:1201778] | Atp6v0d1 | protein_coding |
| ENSMUSG00000013646 | 703.036088 | -0.3704059 | 0.15742291 | 0.00298998 | 0.02699954 | Down | 79566 | SH3 binding domain protein 5 like [Source:MGI Symbol;Acc:MGI:1933124] | Sh3bp5l | protein_coding |
| ENSMUSG00000013663 | 3750.27016 | 0.36338101 | 0.15049417 | 0.0027557 | 0.02563937 | Up | 19211 | phosphatase and tensin homolog [Source:MGI Symbol;Acc:MGI:109583] | Pten | protein_coding |
| ENSMUSG00000013698 | 3234.62514 | -0.6007037 | 0.18989415 | 0.0001581 | 0.00404202 | Down | 18611 | proliferation and apoptosis adaptor protein 15A [Source:MGI Symbol;Acc:MGI:104799] | Pea15a | protein_coding |
| ENSMUSG00000013858 | 2652.82642 | -0.5351145 | 0.17424441 | 0.00020852 | 0.00477778 | Down | 216157 | transmembrane protein 259 [Source:MGI Symbol;Acc:MGI:2177957] | Tmem259 | protein_coding |
| ENSMUSG00000013997 | 687.708317 | -0.3604718 | 0.12927426 | 0.00099847 | 0.01330743 | Down | 27045 | nitrilase 1 [Source:MGI Symbol;Acc:MGI:1350916] | Nit1 | protein_coding |
| ENSMUSG00000014177 | 664.241267 | -0.2989321 | 0.12934966 | 0.00532087 | 0.03941521 | Down | 67510 | trans-golgi network vesicle protein 23B [Source:MGI Symbol;Acc:MGI:1914760] | Tvp23b | protein_coding |
| ENSMUSG00000014294 | 1494.97221 | -1.0852018 | 0.29266192 | 1.06E-05 | 0.00072429 | Down | 17991 | NADH:ubiquinone oxidoreductase subunit A2 [Source:MGI Symbol;Acc:MGI:1343103] | Ndufa2 | protein_coding |
| ENSMUSG00000014418 | 1108.40694 | 0.2407412 | 0.08018267 | 0.0011064 | 0.01418587 | Up | 246694 | HPS5, biogenesis of lysosomal organelles complex 2 subunit 2 [Source:MGI Symbol;Acc:MGI:2180307] | Hps5 | protein_coding |
| ENSMUSG00000014496 | 520.556777 | 0.35196959 | 0.1062592 | 0.00019302 | 0.00456711 | Up | 105522 | ankyrin repeat domain 28 [Source:MGI Symbol;Acc:MGI:2145661] | Ankrd28 | protein_coding |
| ENSMUSG00000014551 | 423.186346 | -0.4402378 | 0.15550836 | 0.00059228 | 0.00943301 | Down | 64658 | mitochondrial ribosomal protein S25 [Source:MGI Symbol;Acc:MGI:1928140] | Mrps25 | protein_coding |
| ENSMUSG00000014633 | 249.170811 | -0.9209365 | 0.2363998 | 5.60E-06 | 0.00044805 | Down | 66531 | COX assembly mitochondrial protein 2 [Source:MGI Symbol;Acc:MGI:1913781] | Cmc2 | protein_coding |
| ENSMUSG00000014776 | 784.551916 | -0.7845587 | 0.15868914 | 5.48E-08 | 1.78E-05 | Down | 78688 | nucleolar protein 3 (apoptosis repressor with CARD domain) [Source:MGI Symbol;Acc:MGI:1925938] | Nol3 | protein_coding |
| ENSMUSG00000014813 | 61.4874571 | 0.70539801 | 0.45665595 | 0.00484828 | 0.03708114 | Up | 20855 | stanniocalcin 1 [Source:MGI Symbol;Acc:MGI:109131] | Stc1 | protein_coding |
| ENSMUSG00000014846 | 1394.64687 | -0.7438412 | 0.22259329 | 5.55E-05 | 0.00199124 | Down | 67971 | tubulin polymerization-promoting protein family member 3 [Source:MGI Symbol;Acc:MGI:1915221] | Tppp3 | protein_coding |
| ENSMUSG00000014856 | 473.709031 | -0.4454056 | 0.18748317 | 0.00204711 | 0.02110103 | Down | 66320 | transmembrane protein 208 [Source:MGI Symbol;Acc:MGI:1913570] | Tmem208 | protein_coding |
| ENSMUSG00000014867 | 4843.44201 | -0.1904577 | 0.07549595 | 0.00618312 | 0.04348262 | Down | 20932 | surfeit gene 4 [Source:MGI Symbol;Acc:MGI:98445] | Surf4 | protein_coding |
| ENSMUSG00000015013 | 491.524801 | -0.2973455 | 0.13827483 | 0.00757438 | 0.04993745 | Down | 59005 | trafficking protein particle complex 2-like [Source:MGI Symbol;Acc:MGI:1916295] | Trappc2l | protein_coding |
| ENSMUSG00000015092 | 1673.2253 | -0.7635981 | 0.32009844 | 0.00088325 | 0.01238632 | Down | 59022 | endothelial differentiation-related factor 1 [Source:MGI Symbol;Acc:MGI:1891227] | Edf1 | protein_coding |
| ENSMUSG00000015094 | 2745.82467 | -0.5739308 | 0.10952061 | 1.39E-08 | 7.81E-06 | Down | 18146 | neural proliferation, differentiation and control 1 [Source:MGI Symbol;Acc:MGI:1099802] | Npdc1 | protein_coding |
| ENSMUSG00000015126 | 247.227202 | -0.5411898 | 0.1447154 | 2.15E-05 | 0.00112222 | Down | 68327 | TSR3 20S rRNA accumulation [Source:MGI Symbol;Acc:MGI:1915577] | Tsr3 | protein_coding |
| ENSMUSG00000015214 | 711.1992 | 0.32642159 | 0.13563517 | 0.0036288 | 0.03059148 | Up | 53332 | myotubularin related protein 1 [Source:MGI Symbol;Acc:MGI:1858271] | Mtmr1 | protein_coding |
| ENSMUSG00000015222 | 16.0636787 | 0.85437832 | 0.83353026 | 0.00642615 | 0.04461897 | Up | 17756 | microtubule-associated protein 2 [Source:MGI Symbol;Acc:MGI:97175] | Map2 | protein_coding |
| ENSMUSG00000015247 | 567.347609 | -0.2910072 | 0.09936261 | 0.0009153 | 0.0126653 | Down | 66536 | nipsnap homolog 3B [Source:MGI Symbol;Acc:MGI:1913786] | Nipsnap3b | protein_coding |
| ENSMUSG00000015289 | 395.475972 | -0.576564 | 0.22095098 | 0.00081839 | 0.01174443 | Down | 66192 | L antigen family, member 3 [Source:MGI Symbol;Acc:MGI:1913442] | Lage3 | protein_coding |
| ENSMUSG00000015312 | 183.510133 | -0.9151129 | 0.35757719 | 0.0004686 | 0.00798777 | Down | 17873 | growth arrest and DNA-damage-inducible 45 beta [Source:MGI Symbol;Acc:MGI:107776] | Gadd45b | protein_coding |
| ENSMUSG00000015335 | 160.762196 | -0.5051674 | 0.19531295 | 0.00094379 | 0.01296022 | Down | 66220 | zinc finger, DHHC domain containing 12 [Source:MGI Symbol;Acc:MGI:1913470] | Zdhhc12 | protein_coding |
| ENSMUSG00000015337 | 305.097496 | -0.6575516 | 0.16150403 | 3.94E-06 | 0.00036162 | Down | 13804 | endonuclease G [Source:MGI Symbol;Acc:MGI:1261433] | Endog | protein_coding |
| ENSMUSG00000015354 | 1221.2043 | -1.2440262 | 0.28927739 | 8.36E-07 | 0.00012935 | Down | 76477 | procollagen C-endopeptidase enhancer 2 [Source:MGI Symbol;Acc:MGI:1923727] | Pcolce2 | protein_coding |
| ENSMUSG00000015474 | 385.407456 | -0.3052382 | 0.13250214 | 0.00505006 | 0.03798203 | Down | 54397 | palmitoyl-protein thioesterase 2 [Source:MGI Symbol;Acc:MGI:1860075] | Ppt2 | protein_coding |
| ENSMUSG00000015478 | 440.411637 | -0.4445019 | 0.24095141 | 0.00613022 | 0.04316666 | Down | 54197 | ring finger protein 5 [Source:MGI Symbol;Acc:MGI:1860076] | Rnf5 | protein_coding |
| ENSMUSG00000015488 | 530.464125 | -0.2169991 | 0.07418464 | 0.00156628 | 0.01770086 | Down | 381356 | calcium channel flower domain containing 1 [Source:MGI Symbol;Acc:MGI:1924317] | Cacfd1 | protein_coding |
| ENSMUSG00000015522 | 1503.40853 | 0.28184165 | 0.08847566 | 0.00047205 | 0.00803813 | Up | 11863 | aryl hydrocarbon receptor nuclear translocator [Source:MGI Symbol;Acc:MGI:88071] | Arnt | protein_coding |
| ENSMUSG00000015597 | 635.519033 | 0.36868086 | 0.12892896 | 0.00080495 | 0.01163158 | Up | 57908 | zinc finger protein 318 [Source:MGI Symbol;Acc:MGI:1889348] | Zfp318 | protein_coding |
| ENSMUSG00000015697 | 610.233634 | 0.29181178 | 0.10989669 | 0.00227994 | 0.02258388 | Up | 84505 | SET domain, bifurcated 1 [Source:MGI Symbol;Acc:MGI:1934229] | Setdb1 | protein_coding |
| ENSMUSG00000015745 | 448.736286 | -0.4667291 | 0.25886113 | 0.00631118 | 0.04410755 | Down | 67220 | pleckstrin homology domain containing, family O member 1 [Source:MGI Symbol;Acc:MGI:1914470] | Plekho1 | protein_coding |
| ENSMUSG00000015749 | 1152.66968 | 0.25068382 | 0.0912479 | 0.00224135 | 0.02233787 | Up | 66471 | acidic (leucine-rich) nuclear phosphoprotein 32 family, member E [Source:MGI Symbol;Acc:MGI:1913721] | Anp32e | protein_coding |
| ENSMUSG00000015757 | 543.367084 | 0.43376221 | 0.13852056 | 0.00025348 | 0.00549267 | Up | 67418 | peptidylprolyl isomerase (cyclophilin)-like 4 [Source:MGI Symbol;Acc:MGI:1914668] | Ppil4 | protein_coding |

| | | | | | | | | | | |
|---|---|---|---|---|---|---|---|---|---|---|
| ENSMUSG00000015837 | 7721.42985 | -0.2604298 | 0.11188649 | 0.00533044 | 0.0394522 | Down | 18412 | sequestosome 1 [Source:MGI Symbol;Acc:MGI:107931] | Sqstm1 | protein_coding |
| ENSMUSG00000015850 | 872.677954 | -0.3482235 | 0.17084444 | 0.00755464 | 0.04988808 | Down | 229595 | ADAMTS-like 4 [Source:MGI Symbol;Acc:MGI:2389008] | Adamtsl4 | protein_coding |
| ENSMUSG00000015882 | 180.773766 | 0.46891678 | 0.22118853 | 0.00344405 | 0.02949322 | Up | 209707 | ligand dependent nuclear receptor corepressor-like [Source:MGI Symbol;Acc:MGI:2651932] | Lcorl | protein_coding |
| ENSMUSG00000015943 | 263.653255 | -1.098999 | 0.20189355 | 3.36E-09 | 3.04E-06 | Down | 69168 | bolA-like 1 (E. coli) [Source:MGI Symbol;Acc:MGI:1916418] | Bola1 | protein_coding |
| ENSMUSG00000015957 | 226.090702 | -0.5506648 | 0.25534036 | 0.00236065 | 0.02317822 | Down | 22411 | wingless-type MMTV integration site family, member 11 [Source:MGI Symbol;Acc:MGI:101948] | Wnt11 | protein_coding |
| ENSMUSG00000016024 | 4763.9712 | -0.8353473 | 0.29373097 | 0.00024864 | 0.00543629 | Down | 16803 | lipopolysaccharide binding protein [Source:MGI Symbol;Acc:MGI:1098776] | Lbp | protein_coding |
| ENSMUSG00000016181 | 230.367271 | 0.39419987 | 0.14408313 | 0.00103059 | 0.01354527 | Up | 215193 | UTP25 small subunit processome component [Source:MGI Symbol;Acc:MGI:2138080] | Utp25 | protein_coding |
| ENSMUSG00000016252 | 1843.73464 | -0.9409053 | 0.27574201 | 3.43E-05 | 0.00149515 | Down | 67126 | ATP synthase, H+ transporting, mitochondrial F1 complex, epsilon subunit [Source:MGI Symbol;Acc:MGI:1855697] | Atp5e | protein_coding |
| ENSMUSG00000016256 | 3259.72298 | -0.6866069 | 0.19403412 | 3.25E-05 | 0.00145985 | Down | 64138 | cathepsin Z [Source:MGI Symbol;Acc:MGI:1891190] | Ctsz | protein_coding |
| ENSMUSG00000016344 | 699.611828 | -0.3833861 | 0.16276129 | 0.00280343 | 0.02590538 | Down | 66496 | pancreatic progenitor cell differentiation and proliferation factor [Source:MGI Symbol;Acc:MGI:1913746] | Ppdpf | protein_coding |
| ENSMUSG00000016409 | 414.128337 | 0.30196438 | 0.13509205 | 0.00632156 | 0.04411245 | Up | 67050 | NFKB activating protein [Source:MGI Symbol;Acc:MGI:1914300] | Nkap | protein_coding |
| ENSMUSG00000016493 | 74.7805455 | 0.59488576 | 0.27438068 | 0.00209024 | 0.02134251 | Up | 17221 | CD46 antigen, complement regulatory protein [Source:MGI Symbol;Acc:MGI:1203290] | Cd46 | protein_coding |
| ENSMUSG00000016528 | 3150.04495 | -0.4106624 | 0.13514509 | 0.00031991 | 0.00636881 | Down | 17164 | MAP kinase-activated protein kinase 2 [Source:MGI Symbol;Acc:MGI:109298] | Mapkapk2 | protein_coding |
| ENSMUSG00000017167 | 283.935029 | 0.38728285 | 0.17199988 | 0.00370232 | 0.03100213 | Up | 53321 | contactin associated protein-like 1 [Source:MGI Symbol;Acc:MGI:1858201] | Cntnap1 | protein_coding |
| ENSMUSG00000017176 | 287.429095 | -0.2919532 | 0.12905486 | 0.00595163 | 0.04229406 | Down | 68106 | 5'-nucleotidase, cytosolic IIIB [Source:MGI Symbol;Acc:MGI:1915356] | Nt5c3b | protein_coding |
| ENSMUSG00000017188 | 824.209275 | -0.5678998 | 0.16667027 | 6.44E-05 | 0.00219898 | Down | 52469 | cytochrome C oxidase assembly factor 3 [Source:MGI Symbol;Acc:MGI:1098757] | Coa3 | protein_coding |
| ENSMUSG00000017299 | 277.10255 | -0.4496296 | 0.18302977 | 0.00165329 | 0.01831478 | Down | 76233 | deoxynucleotidyltransferase, terminal, interacting protein 1 [Source:MGI Symbol;Acc:MGI:1923483] | Dnttip1 | protein_coding |
| ENSMUSG00000017309 | 1910.26232 | 0.44759303 | 0.22817037 | 0.00490409 | 0.03733222 | Up | 52685 | CD300 molecule like family member G [Source:MGI Symbol;Acc:MGI:1289168] | Cd300lg | protein_coding |
| ENSMUSG00000017418 | 551.992478 | 0.40175449 | 0.15918647 | 0.00166654 | 0.01839198 | Up | 75869 | ADP-ribosylation factor-like 5B [Source:MGI Symbol;Acc:MGI:1923119] | Arl5b | protein_coding |
| ENSMUSG00000017421 | 2759.15622 | 0.2762427 | 0.10687019 | 0.00312059 | 0.02782324 | Up | 22680 | zinc finger protein 207 [Source:MGI Symbol;Acc:MGI:1340045] | Zfp207 | protein_coding |
| ENSMUSG00000017493 | 19505.5561 | -0.5346286 | 0.09504558 | 2.83E-09 | 3.04E-06 | Down | 16010 | insulin-like growth factor binding protein 4 [Source:MGI Symbol;Acc:MGI:96439] | Igfbp4 | protein_coding |
| ENSMUSG00000017548 | 786.235481 | 0.42729314 | 0.11925833 | 5.37E-05 | 0.00194658 | Up | 52615 | SUZ12 polycomb repressive complex 2 subunit [Source:MGI Symbol;Acc:MGI:1261758] | Suz12 | protein_coding |
| ENSMUSG00000017715 | 807.675979 | -0.3622214 | 0.10775615 | 0.00015944 | 0.00405324 | Down | 74451 | phosphatidylglycerophosphate synthase 1 [Source:MGI Symbol;Acc:MGI:1921701] | Pgs1 | protein_coding |
| ENSMUSG00000017747 | 451.049958 | -0.3313482 | 0.10696461 | 0.0003854 | 0.00714005 | Down | 80860 | GH3 domain containing [Source:MGI Symbol;Acc:MGI:1931556] | Ghdc | protein_coding |
| ENSMUSG00000017754 | 4383.64675 | -0.4495016 | 0.16457353 | 0.00077808 | 0.01139694 | Down | 18830 | phospholipid transfer protein [Source:MGI Symbol;Acc:MGI:103151] | Pltp | protein_coding |
| ENSMUSG00000017760 | 3383.44003 | -0.2907889 | 0.08086578 | 0.00010087 | 0.00291573 | Down | 19025 | cathepsin A [Source:MGI Symbol;Acc:MGI:97748] | Ctsa | protein_coding |
| ENSMUSG00000017765 | 1312.14324 | -0.3652962 | 0.0950953 | 2.42E-05 | 0.00120579 | Down | 20498 | solute carrier family 12, member 4 [Source:MGI Symbol;Acc:MGI:1309465] | Slc12a4 | protein_coding |
| ENSMUSG00000017837 | 773.520869 | -0.2842416 | 0.10531701 | 0.00206241 | 0.02119153 | Down | 71966 | NFKB inhibitor interacting Ras-like protein 2 [Source:MGI Symbol;Acc:MGI:1919216] | Nkiras2 | protein_coding |
| ENSMUSG00000017897 | 345.252086 | -0.5646963 | 0.18213795 | 0.0001782 | 0.00435971 | Down | 14049 | EYA transcriptional coactivator and phosphatase 2 [Source:MGI Symbol;Acc:MGI:109341] | Eya2 | protein_coding |
| ENSMUSG00000018040 | 757.050901 | -0.3288376 | 0.1213204 | 0.00143212 | 0.01677936 | Down | 74778 | ribosomal RNA processing 7 homolog A [Source:MGI Symbol;Acc:MGI:1922028] | Rrp7a | protein_coding |
| ENSMUSG00000018042 | 5890.98108 | -0.4350062 | 0.09824957 | 2.16E-06 | 0.00024589 | Down | 109754 | cytochrome b5 reductase 3 [Source:MGI Symbol;Acc:MGI:94893] | Cyb5r3 | protein_coding |
| ENSMUSG00000018102 | 2494.70865 | -0.5234635 | 0.22952082 | 0.00215052 | 0.02184818 | Down | 68024 | H2B clustered histone 4 [Source:MGI Symbol;Acc:MGI:1915274] | H2bc4 | protein_coding |
| ENSMUSG00000018160 | 677.027937 | 0.26063642 | 0.09728587 | 0.00251852 | 0.02413899 | Up | 19014 | mediator complex subunit 1 [Source:MGI Symbol;Acc:MGI:1100846] | Med1 | protein_coding |
| ENSMUSG00000018199 | 582.087602 | 0.42394304 | 0.16196199 | 0.00120402 | 0.01500733 | Up | 20822 | Ro60, Y RNA binding protein [Source:MGI Symbol;Acc:MGI:106652] | Ro60 | protein_coding |
| ENSMUSG00000018286 | 1527.2827 | -0.5080233 | 0.17612177 | 0.00040536 | 0.00734114 | Down | 19175 | proteasome (prosome, macropain) subunit, beta type 6 [Source:MGI Symbol;Acc:MGI:104880] | Psmb6 | protein_coding |
| ENSMUSG00000018293 | 4261.84005 | -0.5978102 | 0.2042812 | 0.0002901 | 0.00598739 | Down | 18643 | profilin 1 [Source:MGI Symbol;Acc:MGI:97549] | Pfn1 | protein_coding |
| ENSMUSG00000018340 | 12410.3395 | -0.5400208 | 0.1038474 | 2.29E-08 | 9.39E-06 | Down | 11749 | annexin A6 [Source:MGI Symbol;Acc:MGI:88255] | Anxa6 | protein_coding |
| ENSMUSG00000018372 | 245.979552 | 0.30370297 | 0.14108247 | 0.00756756 | 0.04993295 | Up | 320162 | centrosomal protein 95 [Source:MGI Symbol;Acc:MGI:2443502] | Cep95 | protein_coding |
| ENSMUSG00000018377 | 1011.12477 | 0.3316489 | 0.13514432 | 0.00323198 | 0.0283803 | Up | 22344 | vascular endothelial zinc finger 1 [Source:MGI Symbol;Acc:MGI:1313291] | Vezf1 | protein_coding |
| ENSMUSG00000018395 | 385.866746 | -0.2579005 | 0.10795266 | 0.00561451 | 0.0406814 | Down | 16568 | kinesin family member 3A [Source:MGI Symbol;Acc:MGI:107689] | Kif3a | protein_coding |
| ENSMUSG00000018405 | 194.639372 | -0.4116853 | 0.17097215 | 0.00224955 | 0.02237842 | Down | 217038 | mitochondrial rRNA methyltransferase 1 [Source:MGI Symbol;Acc:MGI:2443470] | Mrm1 | protein_coding |
| ENSMUSG00000018417 | 749.015178 | 0.56318192 | 0.22560006 | 0.00100299 | 0.01334051 | Up | 17912 | myosin IB [Source:MGI Symbol;Acc:MGI:107752] | Myo1b | protein_coding |
| ENSMUSG00000018567 | 4875.84904 | -0.30651 | 0.11824178 | 0.00246974 | 0.0238673 | Down | 56486 | gamma-aminobutyric acid receptor associated protein [Source:MGI Symbol;Acc:MGI:1861742] | Gabarap | protein_coding |
| ENSMUSG00000018585 | 1235.99085 | -0.5748206 | 0.21416272 | 0.00059918 | 0.00950549 | Down | 11927 | antioxidant 1 copper chaperone [Source:MGI Symbol;Acc:MGI:1333855] | Atox1 | protein_coding |
| ENSMUSG00000018593 | 54821.9039 | -0.761042 | 0.23247528 | 6.74E-05 | 0.00225926 | Down | 20692 | secreted acidic cysteine rich glycoprotein [Source:MGI Symbol;Acc:MGI:98373] | Sparc | protein_coding |
| ENSMUSG00000018669 | 1116.4221 | -0.5043238 | 0.14173197 | 4.40E-05 | 0.00173708 | Down | 80280 | CDK5 regulatory subunit associated protein 3 [Source:MGI Symbol;Acc:MGI:1933126] | Cdk5rap3 | protein_coding |
| ENSMUSG00000018672 | 1090.44966 | -0.3915488 | 0.12172089 | 0.0002237 | 0.00504031 | Down | 56358 | coatomer protein complex, subunit zeta 2 [Source:MGI Symbol;Acc:MGI:1929008] | Copz2 | protein_coding |
| ENSMUSG00000018740 | 782.70228 | 0.36876302 | 0.14287546 | 0.00190434 | 0.02010138 | Up | 71998 | solute carrier family 25, member 35 [Source:MGI Symbol;Acc:MGI:1919248] | Slc25a35 | protein_coding |
| ENSMUSG00000018765 | 1524.01817 | -0.2853746 | 0.12362156 | 0.00574067 | 0.04135158 | Down | 23879 | FMR1 autosomal homolog 2 [Source:MGI Symbol;Acc:MGI:1346074] | Fxr2 | protein_coding |
| ENSMUSG00000018796 | 23240.9808 | 0.44671623 | 0.18119036 | 0.00168206 | 0.01852162 | Up | 14081 | acyl-CoA synthetase long-chain family member 1 [Source:MGI Symbol;Acc:MGI:102797] | Acsl1 | protein_coding |
| ENSMUSG00000018819 | 1485.8336 | -0.4324358 | 0.23609535 | 0.00722934 | 0.04848376 | Down | 16985 | lymphocyte specific 1 [Source:MGI Symbol;Acc:MGI:96832] | Lsp1 | protein_coding |
| ENSMUSG00000018822 | 3621.89426 | -2.6379675 | 0.52696368 | 2.37E-08 | 9.39E-06 | Down | 54612 | secreted frizzled-related sequence protein 5 [Source:MGI Symbol;Acc:MGI:1860298] | Sfrp5 | protein_coding |
| ENSMUSG00000018846 | 11872.8466 | 0.74573811 | 0.15976478 | 2.36E-07 | 5.41E-05 | Up | 211347 | pantothenate kinase 3 [Source:MGI Symbol;Acc:MGI:2387464] | Pank3 | protein_coding |

| | | | | | | | | | | |
|---|---|---|---|---|---|---|---|---|---|---|
| ENSMUSG00000018865 | 16.6523687 | -1.757021 | 0.79865131 | 0.00081055 | 0.01167356 | Down | 29859 | sulfotransferase family 4A, member 1 [Source:MGI Symbol;Acc:MGI:1888971] | Sult4a1 | protein_coding |
| ENSMUSG00000018906 | 457.725104 | -0.2498792 | 0.08453274 | 0.00106878 | 0.01387877 | Down | 18452 | procollagen-proline, 2-oxoglutarate 4-dioxygenase (proline 4-hydroxylase), alpha II polypeptide [Source:MGI Symbol;Acc:MGI:894286] | P4ha2 | protein_coding |
| ENSMUSG00000018965 | 2068.56068 | -0.2592561 | 0.09695385 | 0.00220915 | 0.02215289 | Down | 22629 | tyrosine 3-monooxygenase/tryptophan 5-monooxygenase activation protein, eta polypeptide [Source:MGI Symbol;Acc:MGI:109194] | Ywhah | protein_coding |
| ENSMUSG00000019054 | 1167.78508 | -0.6432432 | 0.22673858 | 0.00035655 | 0.00683838 | Down | 66437 | fission, mitochondrial 1 [Source:MGI Symbol;Acc:MGI:1913687] | Fis1 | protein_coding |
| ENSMUSG00000019055 | 1960.50159 | -0.3088321 | 0.10680535 | 0.00100516 | 0.01334051 | Down | 18822 | procollagen-lysine, 2-oxoglutarate 5-dioxygenase 1 [Source:MGI Symbol;Acc:MGI:99907] | Plod1 | protein_coding |
| ENSMUSG00000019158 | 603.739825 | -1.3669942 | 0.26862754 | 1.83E-08 | 9.20E-06 | Down | 69094 | transmembrane protein 160 [Source:MGI Symbol;Acc:MGI:1916344] | Tmem160 | protein_coding |
| ENSMUSG00000019188 | 2255.0314 | -0.298106 | 0.07861393 | 4.27E-05 | 0.00169601 | Down | 14950 | histocompatibility 13 [Source:MGI Symbol;Acc:MGI:95886] | H13 | protein_coding |
| ENSMUSG00000019194 | 781.365656 | -0.5915673 | 0.18757142 | 0.00013953 | 0.00367367 | Down | 20266 | sodium channel, voltage-gated, type I, beta [Source:MGI Symbol;Acc:MGI:98247] | Scn1b | protein_coding |
| ENSMUSG00000019254 | 1823.03718 | -0.3759154 | 0.11391566 | 0.00015875 | 0.00404733 | Down | 232807 | protein phosphatase 1, regulatory subunit 12C [Source:MGI Symbol;Acc:MGI:1924258] | Ppp1r12c | protein_coding |
| ENSMUSG00000019261 | 602.526518 | -0.3043322 | 0.12492062 | 0.00362661 | 0.03059148 | Down | 270058 | microtubule-associated protein 1S [Source:MGI Symbol;Acc:MGI:2443304] | Map1s | protein_coding |
| ENSMUSG00000019362 | 821.515832 | -0.7519553 | 0.26922285 | 0.00033363 | 0.00656146 | Down | 101966 | DNA segment, Chr 8, ERATO Doi 738, expressed [Source:MGI Symbol;Acc:MGI:1289231] | D8Ertd738e | protein_coding |
| ENSMUSG00000019370 | 3378.30995 | -0.4559246 | 0.10187194 | 1.12E-06 | 0.0001581 | Down | 12315 | calmodulin 3 [Source:MGI Symbol;Acc:MGI:103249] | Calm3 | protein_coding |
| ENSMUSG00000019428 | 3579.61739 | -0.6377079 | 0.15718396 | 4.32E-06 | 0.00037622 | Down | 14232 | FK506 binding protein 8 [Source:MGI Symbol;Acc:MGI:1341070] | Fkbp8 | protein_coding |
| ENSMUSG00000019433 | 431.708491 | -0.7868676 | 0.23034303 | 3.96E-05 | 0.0016295 | Down | 67903 | GIPC PDZ domain containing family, member 1 [Source:MGI Symbol;Acc:MGI:1926252] | Gipc1 | protein_coding |
| ENSMUSG00000019461 | 850.619962 | -0.3038988 | 0.07940532 | 3.88E-05 | 0.00161517 | Down | 70310 | phospholipid scramblase 3 [Source:MGI Symbol;Acc:MGI:1917560] | Plscr3 | protein_coding |
| ENSMUSG00000019471 | 1974.98719 | -0.4485905 | 0.18113823 | 0.00154298 | 0.01751621 | Down | 12539 | cell division cycle 37 [Source:MGI Symbol;Acc:MGI:109531] | Cdc37 | protein_coding |
| ENSMUSG00000019494 | 1477.2639 | -0.3107396 | 0.14487608 | 0.00710362 | 0.0479133 | Down | 26893 | COP9 signalosome subunit 6 [Source:MGI Symbol;Acc:MGI:1349439] | Cops6 | protein_coding |
| ENSMUSG00000019505 | 10257.8511 | -0.4278549 | 0.18327811 | 0.0024962 | 0.02401966 | Down | 22187 | ubiquitin B [Source:MGI Symbol;Acc:MGI:98888] | Ubb | protein_coding |
| ENSMUSG00000019539 | 881.685187 | -0.6575007 | 0.2333348 | 0.00033835 | 0.00660925 | Down | 52377 | reticulocalbin 3, EF-hand calcium binding domain [Source:MGI Symbol;Acc:MGI:1277122] | Rcn3 | protein_coding |
| ENSMUSG00000019578 | 347.953476 | -0.4426361 | 0.16763473 | 0.00105281 | 0.01373732 | Down | 66530 | UBX domain protein 6 [Source:MGI Symbol;Acc:MGI:1913780] | Ubxn6 | protein_coding |
| ENSMUSG00000019579 | 651.164079 | -0.5747897 | 0.16926101 | 6.63E-05 | 0.00224542 | Down | 28106 | myeloid derived growth factor [Source:MGI Symbol;Acc:MGI:2156020] | Mydgf | protein_coding |
| ENSMUSG00000019647 | 409.712844 | 0.46290682 | 0.24712112 | 0.00585927 | 0.04180232 | Up | 20358 | sema domain, transmembrane domain (TM), and cytoplasmic domain, (semaphorin) 6A [Source:MGI Symbol;Acc:MGI:1203727] | Sema6a | protein_coding |
| ENSMUSG00000019659 | 261.348017 | -0.8924321 | 0.28033593 | 7.95E-05 | 0.00251852 | Down | 72654 | coiled-coil domain containing 12 [Source:MGI Symbol;Acc:MGI:1919904] | Ccdc12 | protein_coding |
| ENSMUSG00000019689 | 213.960077 | -0.8400775 | 0.25466582 | 5.61E-05 | 0.00199528 | Down | 66117 | formation of mitochondrial complex V assembly factor 1 [Source:MGI Symbol;Acc:MGI:1913367] | Fmc1 | protein_coding |
| ENSMUSG00000019699 | 440.20511 | 0.37007465 | 0.16795365 | 0.00466538 | 0.03603857 | Up | 23797 | thymoma viral proto-oncogene 3 [Source:MGI Symbol;Acc:MGI:1345147] | Akt3 | protein_coding |
| ENSMUSG00000019710 | 849.619527 | -0.3310561 | 0.14234167 | 0.00423314 | 0.03384218 | Down | 67707 | mitochondrial ribosomal protein L24 [Source:MGI Symbol;Acc:MGI:1914957] | Mrpl24 | protein_coding |
| ENSMUSG00000019715 | 758.887561 | -0.1571214 | 0.06032109 | 0.00466399 | 0.03603857 | Down | 74412 | GLE1 RNA export mediator (yeast) [Source:MGI Symbol;Acc:MGI:1921662] | Gle1 | protein_coding |
| ENSMUSG00000019779 | 75.0049183 | 0.5787095 | 0.37408466 | 0.00646222 | 0.04472893 | Up | 14302 | fyn-related kinase [Source:MGI Symbol;Acc:MGI:103265] | Frk | protein_coding |
| ENSMUSG00000019787 | 53.6206415 | -0.1203011 | 0.25776573 | 4.65E-05 | 0.0018168 | Down | 76757 | triadin [Source:MGI Symbol;Acc:MGI:1924007] | Trdn | protein_coding |
| ENSMUSG00000019791 | 420.522036 | -0.4782781 | 0.16199925 | 0.00037782 | 0.00706289 | Down | 66847 | histidine triad nucleotide binding protein 3 [Source:MGI Symbol;Acc:MGI:1914097] | Hint3 | protein_coding |
| ENSMUSG00000019872 | 2330.39046 | -0.29612 | 0.13176141 | 0.00747464 | 0.0496425 | Down | 57319 | sphingomyelin phosphodiesterase, acid-like 3A [Source:MGI Symbol;Acc:MGI:1931437] | Smpdl3a | protein_coding |
| ENSMUSG00000019873 | 2149.0839 | 0.39078132 | 0.11969644 | 0.0001989 | 0.0046492 | Up | 28193 | receptor accessory protein 3 [Source:MGI Symbol;Acc:MGI:88930] | Reep3 | protein_coding |
| ENSMUSG00000019878 | 296.882944 | 0.41059409 | 0.13399022 | 0.00034984 | 0.00674958 | Up | 15500 | heat shock factor 2 [Source:MGI Symbol;Acc:MGI:96239] | Hsf2 | protein_coding |
| ENSMUSG00000019883 | 678.670075 | 0.34034793 | 0.15073675 | 0.00486737 | 0.03712226 | Up | 52665 | enoyl Coenzyme A hydratase domain containing 1 [Source:MGI Symbol;Acc:MGI:1277169] | Echdc1 | protein_coding |
| ENSMUSG00000019907 | 1376.47542 | 0.38021386 | 0.09881343 | 2.35E-05 | 0.00119254 | Up | 17931 | protein phosphatase 1, regulatory subunit 12A [Source:MGI Symbol;Acc:MGI:1309528] | Ppp1r12a | protein_coding |
| ENSMUSG00000019966 | 1208.93996 | 0.48926772 | 0.17604164 | 0.0006125 | 0.0096222 | Up | 17311 | kit ligand [Source:MGI Symbol;Acc:MGI:96974] | Kitl | protein_coding |
| ENSMUSG00000019984 | 524.804324 | 0.34700654 | 0.12640304 | 0.00130244 | 0.01574263 | Up | 70208 | mediator complex subunit 23 [Source:MGI Symbol;Acc:MGI:1917458] | Med23 | protein_coding |
| ENSMUSG00000020017 | 40.3412555 | -1.0729905 | 0.48996051 | 0.00102351 | 0.0134631 | Down | 15109 | histidine ammonia lyase [Source:MGI Symbol;Acc:MGI:96010] | Hal | protein_coding |
| ENSMUSG00000020044 | 3797.00523 | 0.64726651 | 0.23060728 | 0.00036004 | 0.00685683 | Up | 21859 | tissue inhibitor of metalloproteinase 3 [Source:MGI Symbol;Acc:MGI:98754] | Timp3 | protein_coding |
| ENSMUSG00000020063 | 411.557024 | 0.49092854 | 0.15403548 | 0.00016193 | 0.00408764 | Up | 93759 | sirtuin 1 [Source:MGI Symbol;Acc:MGI:2135607] | Sirt1 | protein_coding |
| ENSMUSG00000020070 | 287.990049 | 0.45112194 | 0.15381002 | 0.00043239 | 0.00759277 | Up | 70432 | RUN and FYVE domain-containing 2 [Source:MGI Symbol;Acc:MGI:1917682] | Rufy2 | protein_coding |
| ENSMUSG00000020074 | 1172.1776 | 0.33060792 | 0.142829 | 0.00436572 | 0.03446687 | Up | 67500 | cell division cycle and apoptosis regulator 1 [Source:MGI Symbol;Acc:MGI:1914750] | Ccar1 | protein_coding |
| ENSMUSG00000020083 | 52.0779005 | -0.9833962 | 0.45799045 | 0.0011863 | 0.0148328 | Down | 69894 | family with sequence similarity 241, member B [Source:MGI Symbol;Acc:MGI:1917144] | Fam241b | protein_coding |
| ENSMUSG00000020086 | 135.199301 | -0.4784087 | 0.18535183 | 0.00109418 | 0.0140848 | Down | 404634 | macroH2A.2 histone [Source:MGI Symbol;Acc:MGI:3037658] | Macroh2a2 | protein_coding |
| ENSMUSG00000020087 | 1305.99967 | -0.4995074 | 0.158447 | 0.00018229 | 0.00442652 | Down | 71767 | trypsin domain containing 1 [Source:MGI Symbol;Acc:MGI:1919017] | Tysnd1 | protein_coding |
| ENSMUSG00000020088 | 5242.47182 | -0.3331741 | 0.08442732 | 2.05E-05 | 0.00109543 | Down | 20224 | secretion associated Ras related GTPase 1A [Source:MGI Symbol;Acc:MGI:98230] | Sar1a | protein_coding |
| ENSMUSG00000020097 | 2686.99329 | 0.32273612 | 0.14417195 | 0.00563413 | 0.04076902 | Up | 20397 | sphingosine phosphate lyase 1 [Source:MGI Symbol;Acc:MGI:1261415] | Sgpl1 | protein_coding |
| ENSMUSG00000020098 | 287.226663 | -1.2031183 | 0.31603798 | 5.81E-06 | 0.00045849 | Down | 13180 | pterin 4 alpha carbinolamine dehydratase/dimerization cofactor of hepatocyte nuclear factor 1 alpha (TCF1) 1 [Source:MGI Symbol;Acc:MGI:94873] | Pcbd1 | protein_coding |
| ENSMUSG00000020102 | 917.676671 | 0.43770112 | 0.15839133 | 0.00076329 | 0.01124169 | Up | 20503 | solute carrier family 16 (monocarboxylic acid transporters), member 7 [Source:MGI Symbol;Acc:MGI:1330284] | Slc16a7 | protein_coding |
| ENSMUSG00000020107 | 1043.7186 | -0.526289 | 0.12638834 | 2.91E-06 | 0.00029825 | Down | 52717 | anaphase promoting complex subunit 16 [Source:MGI Symbol;Acc:MGI:1289325] | Anapc16 | protein_coding |
| ENSMUSG00000020128 | 1170.46865 | 0.36198491 | 0.11104992 | 0.00023259 | 0.00519722 | Up | 245944 | VPS54 GARP complex subunit [Source:MGI Symbol;Acc:MGI:2178798] | Vps54 | protein_coding |
| ENSMUSG00000020131 | 110.397451 | 0.5368458 | 0.23466347 | 0.00185361 | 0.01974549 | Up | 18551 | proprotein convertase subtilisin/kexin type 4 [Source:MGI Symbol;Acc:MGI:97514] | Pcsk4 | protein_coding |

| | | | | | | | | | | |
|---|---|---|---|---|---|---|---|---|---|---|
| ENSMUSG00000020133 | 679.939783 | -0.4092623 | 0.16608632 | 0.00199901 | 0.0208033 | Down | 66374 | RIKEN cDNA 2310011J03 gene [Source:MGI Symbol;Acc:MGI:1913624] | 2310011J03Rik | protein_coding |
| ENSMUSG00000020142 | 180.119641 | -0.7000284 | 0.2559152 | 0.00040127 | 0.0073079 | Down | 55963 | solute carrier family 1 (glutamate/neutral amino acid transporter), member 4 [Source:MGI Symbol;Acc:MGI:2135601] | Slc1a4 | protein_coding |
| ENSMUSG00000020150 | 972.333763 | -0.9255522 | 0.22348107 | 2.01E-06 | 0.00023289 | Down | 14431 | guanidinoacetate methyltransferase [Source:MGI Symbol;Acc:MGI:1098221] | Gamt | protein_coding |
| ENSMUSG00000020160 | 412.091263 | 0.3750597 | 0.18006162 | 0.00582744 | 0.0417218 | Up | 17268 | Meis homeobox 1 [Source:MGI Symbol;Acc:MGI:104717] | Meis1 | protein_coding |
| ENSMUSG00000020163 | 1218.22987 | -1.2559511 | 0.40171818 | 6.75E-05 | 0.00225926 | Down | 66594 | ubiquinol-cytochrome c reductase, complex III subunit XI [Source:MGI Symbol;Acc:MGI:1913844] | Uqcr11 | protein_coding |
| ENSMUSG00000020166 | 871.386314 | 0.2399996 | 0.07881067 | 0.00094963 | 0.01301841 | Up | 72068 | CCR4-NOT transcription complex, subunit 2 [Source:MGI Symbol;Acc:MGI:1919318] | Cnot2 | protein_coding |
| ENSMUSG00000020170 | 741.97547 | 0.37999427 | 0.1427283 | 0.00139494 | 0.01655278 | Up | 327826 | fibroblast growth factor receptor substrate 2 [Source:MGI Symbol;Acc:MGI:1100860] | Frs2 | protein_coding |
| ENSMUSG00000020183 | 42.0800454 | 0.51553414 | 0.3137287 | 0.00700103 | 0.04748716 | Up | 70574 | carboxypeptidase M [Source:MGI Symbol;Acc:MGI:1917824] | Cpm | protein_coding |
| ENSMUSG00000020219 | 863.99182 | -1.0388862 | 0.28652952 | 1.44E-05 | 0.00088122 | Down | 30055 | translocase of inner mitochondrial membrane 13 [Source:MGI Symbol;Acc:MGI:1353432] | Timm13 | protein_coding |
| ENSMUSG00000020230 | 441.234331 | -0.5412983 | 0.10379578 | 1.66E-08 | 8.72E-06 | Down | 15468 | protein arginine N-methyltransferase 2 [Source:MGI Symbol;Acc:MGI:1316652] | Prmt2 | protein_coding |
| ENSMUSG00000020241 | 9051.32725 | -0.7762389 | 0.26438714 | 0.00017126 | 0.00422167 | Down | 12834 | collagen, type VI, alpha 2 [Source:MGI Symbol;Acc:MGI:88460] | Col6a2 | protein_coding |
| ENSMUSG00000020260 | 868.078579 | -0.3769874 | 0.10015424 | 3.21E-05 | 0.00145985 | Down | 80294 | protein O-fucosyltransferase 2 [Source:MGI Symbol;Acc:MGI:1916863] | Pofut2 | protein_coding |
| ENSMUSG00000020264 | 3194.25837 | 0.43679176 | 0.16175888 | 0.00106072 | 0.01379615 | Up | 246049 | solute carrier family 36 (proton/amino acid symporter), member 2 [Source:MGI Symbol;Acc:MGI:1891430] | Slc36a2 | protein_coding |
| ENSMUSG00000020307 | 1041.19455 | -0.4574039 | 0.25096419 | 0.0063392 | 0.04421651 | Down | 216150 | cell division cycle 34 [Source:MGI Symbol;Acc:MGI:102657] | Cdc34 | protein_coding |
| ENSMUSG00000020308 | 413.551955 | -0.9835228 | 0.22580709 | 7.50E-07 | 0.00011943 | Down | 110012 | tubulin polyglutamylase complex subunit 1 [Source:MGI Symbol;Acc:MGI:106618] | Tpgs1 | protein_coding |
| ENSMUSG00000020325 | 180.200537 | -1.3258923 | 0.26761181 | 3.46E-08 | 1.31E-05 | Down | 83554 | follistatin-like 3 [Source:MGI Symbol;Acc:MGI:1890391] | Fstl3 | protein_coding |
| ENSMUSG00000020328 | 472.222362 | -0.4461324 | 0.19362152 | 0.00230573 | 0.02279766 | Down | 52653 | NudC domain containing 2 [Source:MGI Symbol;Acc:MGI:1277103] | Nudcd2 | protein_coding |
| ENSMUSG00000020331 | 84.1903156 | -0.5069359 | 0.28394185 | 0.00584704 | 0.04177802 | Down | 15166 | hyperpolarization-activated, cyclic nucleotide-gated K+ 2 [Source:MGI Symbol;Acc:MGI:1298210] | Hcn2 | protein_coding |
| ENSMUSG00000020346 | 1573.49064 | -0.3299043 | 0.13359051 | 0.00282239 | 0.02600668 | Down | 17308 | mannoside acetylglucosaminyltransferase 1 [Source:MGI Symbol;Acc:MGI:96973] | Mgat1 | protein_coding |
| ENSMUSG00000020354 | 1053.75409 | -0.4501887 | 0.15317829 | 0.00044041 | 0.00769298 | Down | 24052 | sarcoglycan, delta (dystrophin-associated glycoprotein) [Source:MGI Symbol;Acc:MGI:1346525] | Sgcd | protein_coding |
| ENSMUSG00000020358 | 2142.09805 | -0.6386212 | 0.2306758 | 0.00038564 | 0.00714005 | Down | 15384 | heterogeneous nuclear ribonucleoprotein A/B [Source:MGI Symbol;Acc:MGI:1330294] | Hnrnpab | protein_coding |
| ENSMUSG00000020362 | 916.742955 | 0.40539883 | 0.14397325 | 0.00076618 | 0.01127411 | Up | 104625 | CCR4-NOT transcription complex, subunit 6 [Source:MGI Symbol;Acc:MGI:2144529] | Cnot6 | protein_coding |
| ENSMUSG00000020364 | 92.9647781 | 0.50033482 | 0.24910451 | 0.00383355 | 0.03167651 | Up | 21408 | zinc finger protein 354A [Source:MGI Symbol;Acc:MGI:103172] | Zfp354a | protein_coding |
| ENSMUSG00000020375 | 487.513691 | -0.213111 | 0.07765087 | 0.00289775 | 0.02650557 | Down | 216724 | RUN and FYVE domain containing 1 [Source:MGI Symbol;Acc:MGI:2429762] | Rufy1 | protein_coding |
| ENSMUSG00000020377 | 581.998494 | -0.8252506 | 0.25386001 | 6.80E-05 | 0.00225926 | Down | 17001 | leukotriene C4 synthase [Source:MGI Symbol;Acc:MGI:107498] | Ltc4s | protein_coding |
| ENSMUSG00000020380 | 1091.92546 | -0.4253026 | 0.19264965 | 0.00333243 | 0.0289494 | Down | 19360 | RAD50 double strand break repair protein [Source:MGI Symbol;Acc:MGI:109292] | Rad50 | protein_coding |
| ENSMUSG00000020385 | 955.856459 | 0.3694539 | 0.16271942 | 0.00394481 | 0.03232446 | Up | 12750 | CDC like kinase 4 [Source:MGI Symbol;Acc:MGI:1098551] | Clk4 | protein_coding |
| ENSMUSG00000020388 | 283.012742 | -0.7893556 | 0.20220377 | 6.24E-06 | 0.00047968 | Down | 30794 | PDZ and LIM domain 4 [Source:MGI Symbol;Acc:MGI:1353470] | Pdlim4 | protein_coding |
| ENSMUSG00000020400 | 824.939364 | -0.626826 | 0.13744974 | 4.98E-07 | 8.80E-05 | Down | 57783 | TNFAIP3 interacting protein 1 [Source:MGI Symbol;Acc:MGI:1926194] | Tnip1 | protein_coding |
| ENSMUSG00000020415 | 463.797062 | -0.5101742 | 0.1834386 | 0.00052041 | 0.008662 | Down | 30939 | pituitary tumor-transforming gene 1 [Source:MGI Symbol;Acc:MGI:1353578] | Pttg1 | protein_coding |
| ENSMUSG00000020424 | 312.252972 | -0.5733005 | 0.24681302 | 0.00152523 | 0.01741052 | Down | 71962 | cytosolic arginine sensor for mTORC1 subunit 1 [Source:MGI Symbol;Acc:MGI:1919212] | Castor1 | protein_coding |
| ENSMUSG00000020440 | 1323.9728 | -0.5106439 | 0.15282154 | 9.44E-05 | 0.00280835 | Down | 11844 | ADP-ribosylation factor 5 [Source:MGI Symbol;Acc:MGI:99434] | Arf5 | protein_coding |
| ENSMUSG00000020441 | 193.940332 | -0.4848549 | 0.18997402 | 0.0011513 | 0.01453259 | Down | 67862 | RIKEN cDNA 2310033P09 gene [Source:MGI Symbol;Acc:MGI:1915112] | 2310033P09Rik | protein_coding |
| ENSMUSG00000020444 | 605.199616 | -0.5757938 | 0.16349811 | 4.09E-05 | 0.00165324 | Down | 14923 | guanylate kinase 1 [Source:MGI Symbol;Acc:MGI:95871] | Guk1 | protein_coding |
| ENSMUSG00000020448 | 579.892723 | 0.2657854 | 0.09123937 | 0.00116392 | 0.01464591 | Up | 193670 | ring finger protein 185 [Source:MGI Symbol;Acc:MGI:1922078] | Rnf185 | protein_coding |
| ENSMUSG00000020464 | 561.727351 | 0.38844326 | 0.13300305 | 0.00061052 | 0.00960103 | Up | 71701 | polyribonucleotide nucleotidyltransferase 1 [Source:MGI Symbol;Acc:MGI:1918951] | Pnpt1 | protein_coding |
| ENSMUSG00000020476 | 1067.05802 | -0.3209873 | 0.14656077 | 0.00638048 | 0.04443173 | Down | 13169 | drebrin-like [Source:MGI Symbol;Acc:MGI:700006] | Dbnl | protein_coding |
| ENSMUSG00000020477 | 648.874685 | -0.6605186 | 0.20338251 | 8.78E-05 | 0.00267477 | Down | 64660 | mitochondrial ribosomal protein S24 [Source:MGI Symbol;Acc:MGI:1928142] | Mrps24 | protein_coding |
| ENSMUSG00000020485 | 571.654381 | -0.584986 | 0.16865865 | 4.70E-05 | 0.00183193 | Down | 20922 | SPT4A, DSIF elongation factor subunit [Source:MGI Symbol;Acc:MGI:107416] | Supt4a | protein_coding |
| ENSMUSG00000020496 | 2748.0613 | -0.395193 | 0.1252898 | 0.00028181 | 0.00584669 | Down | 108660 | ring finger protein 187 [Source:MGI Symbol;Acc:MGI:1914224] | Rnf187 | protein_coding |
| ENSMUSG00000020514 | 206.9465 | -0.4216541 | 0.20742898 | 0.00498039 | 0.03773619 | Down | 216767 | mitochondrial ribosomal protein L22 [Source:MGI Symbol;Acc:MGI:1333794] | Mrpl22 | protein_coding |
| ENSMUSG00000020519 | 438.197871 | -0.4718523 | 0.16959489 | 0.00063125 | 0.00981306 | Down | 50724 | SAP30-like [Source:MGI Symbol;Acc:MGI:1354709] | Sap30l | protein_coding |
| ENSMUSG00000020532 | 20863.902 | 1.08635482 | 0.35491423 | 0.00010317 | 0.00297701 | Up | 107476 | acetyl-Coenzyme A carboxylase alpha [Source:MGI Symbol;Acc:MGI:108451] | Acaca | protein_coding |
| ENSMUSG00000020544 | 61.2360481 | -0.7103573 | 0.60615559 | 0.00684931 | 0.04673877 | Down | 69802 | cytochrome c oxidase assembly protein 11, copper chaperone [Source:MGI Symbol;Acc:MGI:1917052] | Cox11 | protein_coding |
| ENSMUSG00000020571 | 3533.13516 | -0.3741029 | 0.13209481 | 0.000649 | 0.01003147 | Down | 71853 | protein disulfide isomerase associated 6 [Source:MGI Symbol;Acc:MGI:1919103] | Pdia6 | protein_coding |
| ENSMUSG00000020572 | 2592.97402 | 0.3738684 | 0.1763013 | 0.00537825 | 0.03959432 | Up | 59027 | nicotinamide phosphoribosyltransferase [Source:MGI Symbol;Acc:MGI:1929865] | Nampt | protein_coding |
| ENSMUSG00000020590 | 1053.80605 | 0.21735844 | 0.07343668 | 0.00130584 | 0.01577198 | Up | 217463 | sorting nexin 13 [Source:MGI Symbol;Acc:MGI:2661416] | Snx13 | protein_coding |
| ENSMUSG00000020592 | 393.651546 | -0.4696043 | 0.23841032 | 0.00463648 | 0.0359007 | Down | 20969 | syndecan 1 [Source:MGI Symbol;Acc:MGI:1349162] | Sdc1 | protein_coding |
| ENSMUSG00000020594 | 2832.67752 | 0.24389041 | 0.10325905 | 0.00708202 | 0.04783037 | Up | 80913 | pumilio RNA-binding family member 2 [Source:MGI Symbol;Acc:MGI:1931751] | Pum2 | protein_coding |
| ENSMUSG00000020611 | 1201.67739 | 0.21025101 | 0.08397527 | 0.00543323 | 0.03993618 | Up | 14674 | guanine nucleotide binding protein, alpha 13 [Source:MGI Symbol;Acc:MGI:95768] | Gna13 | protein_coding |
| ENSMUSG00000020623 | 97.2491751 | -0.6568147 | 0.31528986 | 0.00207448 | 0.02122768 | Down | 26399 | mitogen-activated protein kinase kinase 6 [Source:MGI Symbol;Acc:MGI:1346870] | Map2k6 | protein_coding |
| ENSMUSG00000020640 | 1301.36663 | 0.33902653 | 0.11959892 | 0.00100768 | 0.01335224 | Up | 20403 | intersectin 2 [Source:MGI Symbol;Acc:MGI:1338049] | Itsn2 | protein_coding |
| ENSMUSG00000020642 | 959.436125 | 0.24358537 | 0.09609641 | 0.00427676 | 0.03407355 | Up | 108089 | ring finger protein 144A [Source:MGI Symbol;Acc:MGI:1344401] | Rnf144a | protein_coding |

| | | | | | | | | | | |
|---|---|---|---|---|---|---|---|---|---|---|
| ENSMUSG00000020659 | 149.065391 | 0.48724375 | 0.16086407 | 0.00028788 | 0.00594986 | Up | 104836 | Casitas B-lineage lymphoma-like 1 [Source:MGI Symbol;Acc:MGI:2144842] | Cbll1 | protein_coding |
| ENSMUSG00000020687 | 1633.3709 | 0.39905394 | 0.10851846 | 4.21E-05 | 0.00168414 | Up | 217232 | cell division cycle 27 [Source:MGI Symbol;Acc:MGI:102685] | Cdc27 | protein_coding |
| ENSMUSG00000020694 | 736.309895 | 0.29429423 | 0.08249869 | 0.00010518 | 0.00301348 | Up | 24086 | tousled-like kinase 2 (Arabidopsis) [Source:MGI Symbol;Acc:MGI:1346023] | Tlk2 | protein_coding |
| ENSMUSG00000020695 | 928.878388 | -2.0011546 | 0.63117514 | 4.93E-05 | 0.00187013 | Down | 17534 | mannose receptor, C type 2 [Source:MGI Symbol;Acc:MGI:107818] | Mrc2 | protein_coding |
| ENSMUSG00000020706 | 871.755794 | -0.3329373 | 0.14229933 | 0.0038412 | 0.03171305 | Down | 56095 | FtsJ RNA methyltransferase homolog 3 (E. coli) [Source:MGI Symbol;Acc:MGI:1860295] | Ftsj3 | protein_coding |
| ENSMUSG00000020723 | 8.4803244 | 0.18723193 | 0.34994224 | 0.00379598 | 0.03146211 | Up | 54377 | calcium channel, voltage-dependent, gamma subunit 4 [Source:MGI Symbol;Acc:MGI:1859167] | Cacng4 | protein_coding |
| ENSMUSG00000020736 | 459.60143 | -0.6956791 | 0.22694764 | 0.0001508 | 0.00389592 | Down | 50773 | 5',3'-nucleotidase, cytosolic [Source:MGI Symbol;Acc:MGI:1354954] | Nt5c | protein_coding |
| ENSMUSG00000020737 | 1592.90654 | -0.45336 | 0.16655773 | 0.00077 | 0.01130978 | Down | 15374 | Jupiter microtubule associated homolog 1 [Source:MGI Symbol;Acc:MGI:1096361] | Jpt1 | protein_coding |
| ENSMUSG00000020743 | 1373.6908 | -0.2927758 | 0.1259799 | 0.005332 | 0.0394522 | Down | 69674 | MIF4G domain containing [Source:MGI Symbol;Acc:MGI:1916924] | Mif4gd | protein_coding |
| ENSMUSG00000020755 | 560.771971 | -0.2461054 | 0.08093631 | 0.00081168 | 0.01167918 | Down | 57230 | SAP30 binding protein [Source:MGI Symbol;Acc:MGI:1927479] | Sap30bp | protein_coding |
| ENSMUSG00000020783 | 493.899891 | 0.22684998 | 0.07519075 | 0.00113656 | 0.01442449 | Up | 66874 | nuclear cap binding subunit 3 [Source:MGI Symbol;Acc:MGI:1914124] | Ncbp3 | protein_coding |
| ENSMUSG00000020801 | 130.216601 | -0.3867216 | 0.16803115 | 0.0031367 | 0.0278751 | Down | 67279 | mediator complex subunit 31 [Source:MGI Symbol;Acc:MGI:1914529] | Med31 | protein_coding |
| ENSMUSG00000020808 | 18.5570939 | -2.0264132 | 0.65685552 | 7.44E-05 | 0.00240273 | Down | 109212 | PICALM interacting mitotic regulator [Source:MGI Symbol;Acc:MGI:1924434] | Pimreg | protein_coding |
| ENSMUSG00000020836 | 218.18626 | 0.64124383 | 0.39898717 | 0.00510573 | 0.03827231 | Up | 216961 | coronin 6 [Source:MGI Symbol;Acc:MGI:2183448] | Coro6 | protein_coding |
| ENSMUSG00000020841 | 3112.40315 | 0.27797209 | 0.11226266 | 0.00407913 | 0.03296898 | Up | 12874 | carboxypeptidase D [Source:MGI Symbol;Acc:MGI:107265] | Cpd | protein_coding |
| ENSMUSG00000020843 | 417.547802 | -0.3277706 | 0.13087959 | 0.00272058 | 0.02542912 | Down | 56322 | translocase of inner mitochondrial membrane 22 [Source:MGI Symbol;Acc:MGI:1929742] | Timm22 | protein_coding |
| ENSMUSG00000020844 | 410.502081 | -0.5124057 | 0.15261149 | 6.52E-05 | 0.00221232 | Down | 18230 | nucleoredoxin [Source:MGI Symbol;Acc:MGI:109331] | Nxn | protein_coding |
| ENSMUSG00000020863 | 1467.93056 | 0.34005717 | 0.15014563 | 0.00478625 | 0.03671039 | Up | 67684 | LUC7-like 3 (S. cerevisiae) [Source:MGI Symbol;Acc:MGI:1914934] | Luc7l3 | protein_coding |
| ENSMUSG00000020877 | 90.2206851 | -0.6332844 | 0.19843996 | 0.00011217 | 0.00313098 | Down | 217140 | secernin 2 [Source:MGI Symbol;Acc:MGI:1343092] | Scrn2 | protein_coding |
| ENSMUSG00000020894 | 1445.01416 | 0.25525033 | 0.10779807 | 0.0061955 | 0.04351315 | Up | 22318 | vesicle-associated membrane protein 2 [Source:MGI Symbol;Acc:MGI:1313277] | Vamp2 | protein_coding |
| ENSMUSG00000020917 | 23919.501 | 1.24270494 | 0.46677247 | 0.00030476 | 0.00615863 | Up | 104112 | ATP citrate lyase [Source:MGI Symbol;Acc:MGI:103251] | Acly | protein_coding |
| ENSMUSG00000020962 | 928.898844 | 0.3850458 | 0.09712653 | 1.55E-05 | 0.00090777 | Up | 83602 | general transcription factor II A, 1 [Source:MGI Symbol;Acc:MGI:1933277] | Gtf2a1 | protein_coding |
| ENSMUSG00000020973 | 12.5437859 | -0.9905394 | 0.43357771 | 0.00099358 | 0.01330743 | Down | 109065 | dynein, axonemal assembly factor 2 [Source:MGI Symbol;Acc:MGI:1923566] | Dnaaf2 | protein_coding |
| ENSMUSG00000021000 | 2256.61803 | 0.30514096 | 0.13275028 | 0.00528187 | 0.03921572 | Up | 338320 | MIA SH3 domain ER export factor 2 [Source:MGI Symbol;Acc:MGI:2159614] | Mia2 | protein_coding |
| ENSMUSG00000021091 | 9429.9895 | -0.4030385 | 0.16912654 | 0.00239918 | 0.02338012 | Down | 20716 | serine (or cysteine) peptidase inhibitor, clade A, member 3N [Source:MGI Symbol;Acc:MGI:105045] | Serpina3n | protein_coding |
| ENSMUSG00000021102 | 1786.45592 | -0.7518798 | 0.20871413 | 2.13E-05 | 0.00111477 | Down | 73046 | glutaredoxin 5 [Source:MGI Symbol;Acc:MGI:1920296] | Glrx5 | protein_coding |
| ENSMUSG00000021120 | 113.931805 | 0.40464593 | 0.16551756 | 0.00208164 | 0.02128141 | Up | 110417 | phosphatidylinositol glycan anchor biosynthesis, class H [Source:MGI Symbol;Acc:MGI:99463] | Pigh | protein_coding |
| ENSMUSG00000021124 | 1315.91336 | -0.4324216 | 0.15543169 | 0.00065234 | 0.0100543 | Down | 53612 | vesicle transport through interaction with t-SNAREs 1B [Source:MGI Symbol;Acc:MGI:1855688] | Vti1b | protein_coding |
| ENSMUSG00000021147 | 446.694977 | 0.30274439 | 0.10105308 | 0.00072337 | 0.01090021 | Up | 207615 | WD repeat domain 37 [Source:MGI Symbol;Acc:MGI:1920393] | Wdr37 | protein_coding |
| ENSMUSG00000021156 | 1962.55474 | 0.26631299 | 0.09943397 | 0.00253949 | 0.02428132 | Up | 66505 | zinc finger, MYND domain containing 11 [Source:MGI Symbol;Acc:MGI:1913755] | Zmynd11 | protein_coding |
| ENSMUSG00000021189 | 667.999021 | 0.39776846 | 0.11180479 | 6.71E-05 | 0.00225926 | Up | 110616 | ataxin 3 [Source:MGI Symbol;Acc:MGI:1099442] | Atxn3 | protein_coding |
| ENSMUSG00000021209 | 172.580719 | 0.46854971 | 0.26314946 | 0.00662314 | 0.04553236 | Up | 74521 | protein phosphatase 4, regulatory subunit 4 [Source:MGI Symbol;Acc:MGI:1921771] | Ppp4r4 | protein_coding |
| ENSMUSG00000021213 | 324.896717 | -0.4020676 | 0.19300774 | 0.00496868 | 0.03770026 | Down | 27384 | aldo-keto reductase family 1, member C13 [Source:MGI Symbol;Acc:MGI:1351662] | Akr1c13 | protein_coding |
| ENSMUSG00000021222 | 406.175962 | -0.2723924 | 0.107938 | 0.00365536 | 0.03073564 | Down | 73828 | DDB1 and CUL4 associated factor 4 [Source:MGI Symbol;Acc:MGI:1921078] | Dcaf4 | protein_coding |
| ENSMUSG00000021224 | 649.80827 | 0.266259 | 0.10252314 | 0.00314758 | 0.02792596 | Up | 18222 | NUMB endocytic adaptor protein [Source:MGI Symbol;Acc:MGI:107423] | Numb | protein_coding |
| ENSMUSG00000021241 | 733.540006 | -0.3464763 | 0.12833073 | 0.00155404 | 0.01760485 | Down | 74316 | iron-sulfur cluster assembly 2 [Source:MGI Symbol;Acc:MGI:1921566] | Isca2 | protein_coding |
| ENSMUSG00000021242 | 15157.0447 | -0.6851904 | 0.20650166 | 5.83E-05 | 0.00203978 | Down | 67963 | NPC intracellular cholesterol transporter 2 [Source:MGI Symbol;Acc:MGI:1915213] | Npc2 | protein_coding |
| ENSMUSG00000021286 | 779.66743 | -0.3559525 | 0.11563385 | 0.00048255 | 0.00818272 | Down | 68520 | zinc finger, FYVE domain containing 21 [Source:MGI Symbol;Acc:MGI:1915770] | Zfyve21 | protein_coding |
| ENSMUSG00000021288 | 1402.66368 | -0.3433679 | 0.10873867 | 0.00032828 | 0.00649556 | Down | 16593 | kinesin light chain 1 [Source:MGI Symbol;Acc:MGI:107978] | Klc1 | protein_coding |
| ENSMUSG00000021311 | 646.740463 | 0.33619036 | 0.13239283 | 0.00237756 | 0.02322516 | Up | 238505 | 5-methyltetrahydrofolate-homocysteine methyltransferase [Source:MGI Symbol;Acc:MGI:894292] | Mtr | protein_coding |
| ENSMUSG00000021319 | 238.032637 | -1.2768497 | 0.35765945 | 1.61E-05 | 0.0009316 | Down | 20379 | secreted frizzled-related protein 4 [Source:MGI Symbol;Acc:MGI:892010] | Sfrp4 | protein_coding |
| ENSMUSG00000021327 | 1108.04767 | 0.64076785 | 0.18268577 | 3.89E-05 | 0.00161546 | Up | 72739 | zinc finger with KRAB and SCAN domains 3 [Source:MGI Symbol;Acc:MGI:1919989] | Zkscan3 | protein_coding |
| ENSMUSG00000021339 | 738.889928 | 0.26937644 | 0.08026599 | 0.00025831 | 0.00553592 | Up | 380836 | MRS2 magnesium transporter [Source:MGI Symbol;Acc:MGI:2685748] | Mrs2 | protein_coding |
| ENSMUSG00000021385 | 188.243074 | 0.36709633 | 0.15685636 | 0.00335631 | 0.02904812 | Up | 75678 | inositol 1,3,4,5,6-pentakisphosphate 2-kinase [Source:MGI Symbol;Acc:MGI:1922928] | Ippk | protein_coding |
| ENSMUSG00000021411 | 694.972853 | -0.9766097 | 0.1755413 | 1.64E-09 | 1.91E-06 | Down | 66895 | PX domain containing 1 [Source:MGI Symbol;Acc:MGI:1914145] | Pxdc1 | protein_coding |
| ENSMUSG00000021413 | 1829.79076 | 0.38509283 | 0.15028804 | 0.00172829 | 0.01881773 | Up | 19134 | pre-mRNA processing factor 4B [Source:MGI Symbol;Acc:MGI:109584] | Prpf4b | protein_coding |
| ENSMUSG00000021431 | 462.009514 | 0.32253728 | 0.12441616 | 0.00224328 | 0.0223434 | Up | 67797 | small nuclear ribonucleoprotein 48 (U11/U12) [Source:MGI Symbol;Acc:MGI:1915047] | Snrnp48 | protein_coding |
| ENSMUSG00000021470 | 689.911787 | 0.33021611 | 0.12924884 | 0.00254807 | 0.02430618 | Up | 76251 | excision repair cross-complementing rodent repair deficiency, complementation group 6 like 2 [Source:MGI Symbol;Acc:MGI:1923501] | Ercc6l2 | protein_coding |
| ENSMUSG00000021474 | 4043.96976 | 0.52708258 | 0.30611574 | 0.00586478 | 0.04181297 | Up | 14057 | sideroflexin 1 [Source:MGI Symbol;Acc:MGI:2137677] | Sfxn1 | protein_coding |
| ENSMUSG00000021477 | 9409.74606 | -0.5080101 | 0.18084013 | 0.00041891 | 0.00745369 | Down | 13039 | cathepsin L [Source:MGI Symbol;Acc:MGI:88564] | Ctsl | protein_coding |
| ENSMUSG00000021486 | 2471.20152 | -0.5428277 | 0.15431241 | 4.43E-05 | 0.00173708 | Down | 66494 | PRELI domain containing 1 [Source:MGI Symbol;Acc:MGI:1913744] | Prelid1 | protein_coding |
| ENSMUSG00000021493 | 954.436064 | -0.6737323 | 0.23357469 | 0.00027185 | 0.00570575 | Down | 67399 | PDZ and LIM domain 7 [Source:MGI Symbol;Acc:MGI:1914649] | Pdlim7 | protein_coding |
| ENSMUSG00000021496 | 181.73283 | -1.0098829 | 0.27964286 | 1.54E-05 | 0.00090777 | Down | 72562 | pterin 4 alpha carbinolamine dehydratase/dimerization cofactor of hepatocyte nuclear factor 1 alpha (TCF1) 2 [Source:MGI Symbol;Acc:MGI:1919812] | Pcbd2 | protein_coding |
| ENSMUSG00000021501 | 487.632959 | -0.4411247 | 0.11116817 | 1.05E-05 | 0.00072256 | Down | 12328 | calcium modulating ligand [Source:MGI Symbol;Acc:MGI:104728] | Caml | protein_coding |

| | | | | | | | | | | |
|---|---|---|---|---|---|---|---|---|---|---|
| ENSMUSG00000021510 | 194.190701 | 0.35556891 | 0.16090861 | 0.00490904 | 0.03735242 | Up | 212281 | zinc finger protein 729a [Source:MGI Symbol;Acc:MGI:3036250] | Zfp729a | protein_coding |
| ENSMUSG00000021540 | 1437.91039 | 0.29256984 | 0.07381448 | 2.36E-05 | 0.00119254 | Up | 17129 | SMAD family member 5 [Source:MGI Symbol;Acc:MGI:1328787] | Smad5 | protein_coding |
| ENSMUSG00000021550 | 1157.25015 | -0.4955749 | 0.17064467 | 0.0004138 | 0.00741144 | Down | 70153 | RIKEN cDNA 2210016F16 gene [Source:MGI Symbol;Acc:MGI:1917403] | 2210016F16Rik | protein_coding |
| ENSMUSG00000021576 | 940.900746 | -0.2759828 | 0.12320576 | 0.00736846 | 0.04911805 | Down | 18570 | programmed cell death 6 [Source:MGI Symbol;Acc:MGI:109283] | Pdcd6 | protein_coding |
| ENSMUSG00000021606 | 525.630693 | -0.5465513 | 0.26115668 | 0.00287728 | 0.02637436 | Down | 407785 | NADH:ubiquinone oxidoreductase core subunit S6 [Source:MGI Symbol;Acc:MGI:107932] | Ndufs6 | protein_coding |
| ENSMUSG00000021690 | 704.958842 | 0.3405829 | 0.13355405 | 0.00223931 | 0.02233125 | Up | 57748 | junction-mediating and regulatory protein [Source:MGI Symbol;Acc:MGI:1913096] | Jmy | protein_coding |
| ENSMUSG00000021693 | 561.152035 | 0.26486809 | 0.10524924 | 0.00380888 | 0.03153684 | Up | 16563 | kinesin family member 2A [Source:MGI Symbol;Acc:MGI:108390] | Kif2a | protein_coding |
| ENSMUSG00000021709 | 1788.68944 | 0.34878154 | 0.11447813 | 0.00058227 | 0.00932832 | Up | 59079 | Erbb2 interacting protein [Source:MGI Symbol;Acc:MGI:1890169] | Erbin | protein_coding |
| ENSMUSG00000021713 | 182.166312 | 0.47472889 | 0.13825579 | 7.52E-05 | 0.0024251 | Up | 238831 | peptidylprolyl isomerase domain and WD repeat containing 1 [Source:MGI Symbol;Acc:MGI:2443069] | Ppwd1 | protein_coding |
| ENSMUSG00000021719 | 645.925358 | 0.86763622 | 0.24221962 | 2.06E-05 | 0.00109543 | Up | 52882 | regulator of G-protein signalling 7 binding protein [Source:MGI Symbol;Acc:MGI:106334] | Rgs7bp | protein_coding |
| ENSMUSG00000021738 | 715.518598 | 0.33324834 | 0.11964909 | 0.0012195 | 0.01508817 | Up | 246103 | ataxin 7 [Source:MGI Symbol;Acc:MGI:2179277] | Atxn7 | protein_coding |
| ENSMUSG00000021748 | 4715.44371 | 0.57171988 | 0.21510988 | 0.00066072 | 0.01013548 | Up | 68263 | pyruvate dehydrogenase (lipoamide) beta [Source:MGI Symbol;Acc:MGI:1915513] | Pdhb | protein_coding |
| ENSMUSG00000021752 | 316.748449 | 0.50163119 | 0.15186291 | 0.0001095 | 0.00308292 | Up | 71393 | potassium channel tetramerisation domain containing 6 [Source:MGI Symbol;Acc:MGI:1918643] | Kctd6 | protein_coding |
| ENSMUSG00000021770 | 623.028873 | 0.38868515 | 0.09984001 | 1.85E-05 | 0.00102462 | Up | 67630 | sterile alpha motif domain containing 8 [Source:MGI Symbol;Acc:MGI:1914880] | Samd8 | protein_coding |
| ENSMUSG00000021779 | 564.968275 | 0.54003726 | 0.15102178 | 3.73E-05 | 0.00158342 | Up | 21834 | thyroid hormone receptor beta [Source:MGI Symbol;Acc:MGI:98743] | Thrb | protein_coding |
| ENSMUSG00000021840 | 858.25303 | 0.34133496 | 0.10879423 | 0.00038775 | 0.00714987 | Up | 218975 | mitogen-activated protein kinase 1 interacting protein 1-like [Source:MGI Symbol;Acc:MGI:2444022] | Mapk1ip1l | protein_coding |
| ENSMUSG00000021850 | 41.9256303 | 0.0392667 | 0.20387774 | 0.00445815 | 0.03486891 | Up | 67082 | coiled-coil domain containing 198 [Source:MGI Symbol;Acc:MGI:1914332] | ccdc198 | protein_coding |
| ENSMUSG00000021870 | 1687.05445 | 0.38308205 | 0.14608336 | 0.00152012 | 0.01741052 | Up | 83997 | sarcolemma associated protein [Source:MGI Symbol;Acc:MGI:1933549] | Slmap | protein_coding |
| ENSMUSG00000021945 | 883.522759 | 0.36348602 | 0.15067149 | 0.00307573 | 0.02751393 | Up | 76007 | zinc finger, MYM-type 2 [Source:MGI Symbol;Acc:MGI:1923257] | Zmym2 | protein_coding |
| ENSMUSG00000021957 | 18459.6006 | 0.70647737 | 0.34232009 | 0.00203787 | 0.02105932 | Up | 21881 | transketolase [Source:MGI Symbol;Acc:MGI:105992] | Tkt | protein_coding |
| ENSMUSG00000021962 | 391.985022 | 0.36237819 | 0.11413013 | 0.0002995 | 0.00610744 | Up | 75901 | decapping mRNA 1A [Source:MGI Symbol;Acc:MGI:1923151] | Dcp1a | protein_coding |
| ENSMUSG00000021963 | 1466.24202 | -0.3981653 | 0.15207534 | 0.00120625 | 0.01501193 | Down | 20220 | Sin3-associated polypeptide 18 [Source:MGI Symbol;Acc:MGI:1277978] | Sap18 | protein_coding |
| ENSMUSG00000021967 | 429.000686 | -0.4846995 | 0.15703658 | 0.00024252 | 0.00533125 | Down | 67840 | mitochondrial ribosomal protein L57 [Source:MGI Symbol;Acc:MGI:1915090] | Mrpl57 | protein_coding |
| ENSMUSG00000022018 | 7242.81954 | -0.3363127 | 0.16214354 | 0.00729195 | 0.048748 | Down | 66214 | regulator of cell cycle [Source:MGI Symbol;Acc:MGI:1913464] | Rgcc | protein_coding |
| ENSMUSG00000022020 | 244.443805 | 0.34603996 | 0.15301416 | 0.00461039 | 0.03576683 | Up | 66897 | N(alpha)-acetyltransferase 16, NatA auxiliary subunit [Source:MGI Symbol;Acc:MGI:1914147] | Naa16 | protein_coding |
| ENSMUSG00000022031 | 1343.81595 | -0.3372 | 0.10155325 | 0.00019013 | 0.00454793 | Down | 74195 | elongator acetyltransferase complex subunit 3 [Source:MGI Symbol;Acc:MGI:1921445] | Elp3 | protein_coding |
| ENSMUSG00000022053 | 828.217456 | 0.42936758 | 0.23300784 | 0.0069433 | 0.04721385 | Up | 13592 | early B cell factor 2 [Source:MGI Symbol;Acc:MGI:894332] | Ebf2 | protein_coding |
| ENSMUSG00000022056 | 29.6293181 | 0.00743319 | 0.19841486 | 3.88E-06 | 0.00036162 | Up | 11500 | a disintegrin and metallopeptidase domain 7 [Source:MGI Symbol;Acc:MGI:107247] | Adam7 | protein_coding |
| ENSMUSG00000022089 | 261.091949 | -0.3802742 | 0.15299149 | 0.00187205 | 0.01982492 | Down | 57784 | bridging integrator 3 [Source:MGI Symbol;Acc:MGI:1929883] | Bin3 | protein_coding |
| ENSMUSG00000022100 | 1231.64702 | 0.35218239 | 0.11748997 | 0.00057024 | 0.00917181 | Up | 65246 | exportin 7 [Source:MGI Symbol;Acc:MGI:1929705] | Xpo7 | protein_coding |
| ENSMUSG00000022102 | 416.054547 | -0.6360929 | 0.32406905 | 0.00277789 | 0.02571326 | Down | 13449 | docking protein 2 [Source:MGI Symbol;Acc:MGI:1332623] | Dok2 | protein_coding |
| ENSMUSG00000022105 | 1883.46875 | 0.25586308 | 0.10921453 | 0.00677041 | 0.04630959 | Up | 19645 | RB transcriptional corepressor 1 [Source:MGI Symbol;Acc:MGI:97874] | Rb1 | protein_coding |
| ENSMUSG00000022108 | 31588.7774 | -0.3781687 | 0.10906154 | 0.00010038 | 0.00291573 | Down | 16432 | integral membrane protein 2B [Source:MGI Symbol;Acc:MGI:1309517] | Itm2b | protein_coding |
| ENSMUSG00000022119 | 727.427023 | 0.38464735 | 0.12302868 | 0.00032687 | 0.00647569 | Up | 74213 | RNA binding motif protein 26 [Source:MGI Symbol;Acc:MGI:1921463] | Rbm26 | protein_coding |
| ENSMUSG00000022151 | 441.905918 | -0.3325138 | 0.13807716 | 0.00336156 | 0.02907806 | Down | 67515 | tetratricopeptide repeat domain 33 [Source:MGI Symbol;Acc:MGI:1914765] | Ttc33 | protein_coding |
| ENSMUSG00000022160 | 230.918861 | -0.3543545 | 0.10358427 | 0.00012253 | 0.00335107 | Down | 56335 | methyltransferase like 3 [Source:MGI Symbol;Acc:MGI:1927165] | Mettl3 | protein_coding |
| ENSMUSG00000022174 | 1567.12453 | -0.6420529 | 0.15726902 | 3.93E-06 | 0.00036162 | Down | 13135 | defender against cell death 1 [Source:MGI Symbol;Acc:MGI:101912] | Dad1 | protein_coding |
| ENSMUSG00000022175 | 2000.99479 | -0.2599214 | 0.09934972 | 0.00291791 | 0.02656911 | Down | 65107 | low-density lipoprotein receptor-related protein 10 [Source:MGI Symbol;Acc:MGI:1929480] | Lrp10 | protein_coding |
| ENSMUSG00000022195 | 395.1229 | 0.32183848 | 0.11863599 | 0.0016179 | 0.01802662 | Up | 77877 | RIKEN cDNA 6030458C11 gene [Source:MGI Symbol;Acc:MGI:1925127] | 6030458C11Rik | protein_coding |
| ENSMUSG00000022199 | 484.185913 | -0.4069707 | 0.17347104 | 0.00265995 | 0.02497739 | Down | 59049 | solute carrier family 22 (organic cation transporter), member 17 [Source:MGI Symbol;Acc:MGI:1926225] | Slc22a17 | protein_coding |
| ENSMUSG00000022201 | 2323.29722 | 0.30452053 | 0.11807688 | 0.00260375 | 0.02466347 | Up | 22763 | zinc finger RNA binding protein [Source:MGI Symbol;Acc:MGI:1341890] | Zfr | protein_coding |
| ENSMUSG00000022206 | 21457.0503 | -0.5435139 | 0.27320894 | 0.00372955 | 0.03113389 | Down | 18162 | natriuretic peptide receptor 3 [Source:MGI Symbol;Acc:MGI:97373] | Npr3 | protein_coding |
| ENSMUSG00000022210 | 1125.16547 | -0.3414618 | 0.14399146 | 0.0035172 | 0.02997743 | Down | 28200 | dehydrogenase/reductase (SDR family) member 4 [Source:MGI Symbol;Acc:MGI:90169] | Dhrs4 | protein_coding |
| ENSMUSG00000022217 | 715.280351 | -0.834756 | 0.18376239 | 2.71E-07 | 5.99E-05 | Down | 85308 | ER membrane protein complex subunit 9 [Source:MGI Symbol;Acc:MGI:1934682] | Emc9 | protein_coding |
| ENSMUSG00000022228 | 1298.81972 | 0.49781221 | 0.14725907 | 8.22E-05 | 0.00258765 | Up | 432731 | zinc finger and SCAN domain containing 26 [Source:MGI Symbol;Acc:MGI:3531417] | Zscan26 | protein_coding |
| ENSMUSG00000022305 | 319.28061 | 0.40697309 | 0.16623778 | 0.00207014 | 0.02122294 | Up | 239393 | low density lipoprotein-related protein 12 [Source:MGI Symbol;Acc:MGI:2443132] | Lrp12 | protein_coding |
| ENSMUSG00000022307 | 1673.82926 | 0.2034863 | 0.0820602 | 0.00612039 | 0.04311613 | Up | 170719 | oxidation resistance 1 [Source:MGI Symbol;Acc:MGI:2179326] | Oxr1 | protein_coding |
| ENSMUSG00000022309 | 267.644716 | 0.88363384 | 0.50413043 | 0.0028191 | 0.02599109 | Up | 11600 | angiopoietin 1 [Source:MGI Symbol;Acc:MGI:108448] | Angpt1 | protein_coding |
| ENSMUSG00000022370 | 685.824205 | -0.3706474 | 0.17695381 | 0.00559991 | 0.04066318 | Down | 68537 | mitochondrial ribosomal protein L13 [Source:MGI Symbol;Acc:MGI:2137218] | Mrpl13 | protein_coding |
| ENSMUSG00000022383 | 117.122909 | 0.66140852 | 0.33789405 | 0.00265889 | 0.02497739 | Up | 19013 | peroxisome proliferator activated receptor alpha [Source:MGI Symbol;Acc:MGI:104740] | Ppara | protein_coding |
| ENSMUSG00000022385 | 41.5307622 | -1.1972798 | 0.82900152 | 0.00317613 | 0.02811787 | Down | 29870 | G two S phase expressed protein 1 [Source:MGI Symbol;Acc:MGI:1352755] | Gtse1 | protein_coding |
| ENSMUSG00000022389 | 2494.12719 | 0.69042929 | 0.27436943 | 0.00075165 | 0.01114106 | Up | 21685 | thyrotroph embryonic factor [Source:MGI Symbol;Acc:MGI:98663] | Tef | protein_coding |
| ENSMUSG00000022401 | 917.321103 | 0.37725764 | 0.13635944 | 0.00101975 | 0.01344594 | Up | 321003 | X-prolyl aminopeptidase 3, mitochondrial [Source:MGI Symbol;Acc:MGI:2445217] | Xpnpep3 | protein_coding |

| | | | | | | | | | | |
|---|---|---|---|---|---|---|---|---|---|---|
| ENSMUSG00000022420 | 283.753526 | -0.3364974 | 0.13565436 | 0.00281797 | 0.02599109 | Down | 54152 | dynein, axonemal, light chain 4 [Source:MGI Symbol;Acc:MGI:1859217] | Dnal4 | protein_coding |
| ENSMUSG00000022428 | 179.301064 | -0.4342025 | 0.18780804 | 0.00259345 | 0.02458028 | Down | 73739 | chibby family member 1, beta catenin antagonist [Source:MGI Symbol;Acc:MGI:1920989] | Cby1 | protein_coding |
| ENSMUSG00000022437 | 1704.73646 | -0.396762 | 0.14328304 | 0.00090299 | 0.01253761 | Down | 68653 | SAMM50 sorting and assembly machinery component [Source:MGI Symbol;Acc:MGI:1915903] | Samm50 | protein_coding |
| ENSMUSG00000022452 | 1225.64903 | -0.3999802 | 0.16328574 | 0.00218299 | 0.02196533 | Down | 69029 | single-pass membrane protein with aspartate rich tail 1 [Source:MGI Symbol;Acc:MGI:1916279] | Smdt1 | protein_coding |
| ENSMUSG00000022453 | 982.446479 | -0.2702776 | 0.12045598 | 0.00750375 | 0.04969361 | Down | 17939 | N-acetyl galactosaminidase, alpha [Source:MGI Symbol;Acc:MGI:1261422] | Naga | protein_coding |
| ENSMUSG00000022476 | 349.666579 | -0.6057226 | 0.10582766 | 1.07E-09 | 1.58E-06 | Down | 78929 | polymerase (RNA) III (DNA directed) polypeptide H [Source:MGI Symbol;Acc:MGI:1926179] | Polr3h | protein_coding |
| ENSMUSG00000022485 | 603.7918 | -0.398927 | 0.16501538 | 0.00215831 | 0.02185892 | Down | 15424 | homeobox C5 [Source:MGI Symbol;Acc:MGI:96196] | Hoxc5 | protein_coding |
| ENSMUSG00000022508 | 1362.25773 | 1.01132687 | 0.40184046 | 0.00049852 | 0.00837668 | Up | 12053 | B cell leukemia/lymphoma 6 [Source:MGI Symbol;Acc:MGI:107187] | Bcl6 | protein_coding |
| ENSMUSG00000022516 | 405.760098 | -0.4157202 | 0.13430038 | 0.00034165 | 0.00664584 | Down | 66911 | nudix (nucleoside diphosphate linked moiety X)-type motif 16-like 1 [Source:MGI Symbol;Acc:MGI:1914161] | Nudt16l1 | protein_coding |
| ENSMUSG00000022529 | 377.863211 | 0.38742774 | 0.1191504 | 0.00020811 | 0.00477778 | Up | 74120 | zinc finger protein 263 [Source:MGI Symbol;Acc:MGI:1921370] | Zfp263 | protein_coding |
| ENSMUSG00000022553 | 1084.72758 | -0.2626413 | 0.10271502 | 0.00356967 | 0.03023422 | Down | 68877 | MAF1 homolog, negative regulator of RNA polymerase III [Source:MGI Symbol;Acc:MGI:1916127] | Maf1 | protein_coding |
| ENSMUSG00000022554 | 149.503357 | -0.7146185 | 0.18924269 | 1.20E-05 | 0.00077825 | Down | 59053 | HGH1 homolog [Source:MGI Symbol;Acc:MGI:1930628] | Hgh1 | protein_coding |
| ENSMUSG00000022559 | 477.857614 | -0.2803745 | 0.09551647 | 0.00108762 | 0.01403495 | Down | 30840 | F-box and leucine-rich repeat protein 6 [Source:MGI Symbol;Acc:MGI:1354705] | Fbxl6 | protein_coding |
| ENSMUSG00000022560 | 279.735559 | -0.4099594 | 0.11410741 | 5.28E-05 | 0.00193072 | Down | 52710 | solute carrier protein 52, member 2 [Source:MGI Symbol;Acc:MGI:1289288] | Slc52a2 | protein_coding |
| ENSMUSG00000022561 | 677.729468 | -0.2186247 | 0.08921488 | 0.00530644 | 0.03935283 | Down | 14731 | GPI anchor attachment protein 1 [Source:MGI Symbol;Acc:MGI:1202392] | Gpaa1 | protein_coding |
| ENSMUSG00000022564 | 11046.2577 | -0.2829049 | 0.09815654 | 0.00121566 | 0.01506361 | Down | 66168 | glutamate receptor, ionotropic, N-methyl D-aspartate-associated protein 1 (glutamate binding) [Source:MGI Symbol;Acc:MGI:1913418] | Grina | protein_coding |
| ENSMUSG00000022601 | 277.916696 | 0.31964452 | 0.13516151 | 0.00385763 | 0.03182689 | Up | 271377 | zinc finger and BTB domain containing 11 [Source:MGI Symbol;Acc:MGI:2443876] | Zbtb11 | protein_coding |
| ENSMUSG00000022671 | 325.519967 | -0.6638248 | 0.19293883 | 4.41E-05 | 0.00173708 | Down | 72083 | mitotic spindle organizing protein 2 [Source:MGI Symbol;Acc:MGI:1922845] | Mzt2 | protein_coding |
| ENSMUSG00000022704 | 218.02784 | 0.34557283 | 0.14339014 | 0.00328765 | 0.02876027 | Up | 106248 | queuine tRNA-ribosyltransferase accessory subunit 2 [Source:MGI Symbol;Acc:MGI:1922194] | Qtrt2 | protein_coding |
| ENSMUSG00000022706 | 502.239913 | -0.2778737 | 0.10570326 | 0.00264373 | 0.02487182 | Down | 18100 | mitochondrial ribosomal protein L40 [Source:MGI Symbol;Acc:MGI:1332635] | Mrpl40 | protein_coding |
| ENSMUSG00000022707 | 2264.86667 | 0.75207127 | 0.18458098 | 3.56E-06 | 0.00034867 | Up | 74185 | glucan (1,4-alpha-), branching enzyme 1 [Source:MGI Symbol;Acc:MGI:1921435] | Gbe1 | protein_coding |
| ENSMUSG00000022710 | 1880.85336 | 0.30504479 | 0.07895873 | 3.54E-05 | 0.00152861 | Up | 252870 | ubiquitin specific peptidase 7 [Source:MGI Symbol;Acc:MGI:2182061] | Usp7 | protein_coding |
| ENSMUSG00000022769 | 282.567534 | -1.0223233 | 0.2873706 | 1.92E-05 | 0.00105458 | Down | 64136 | stromal cell-derived factor 2-like 1 [Source:MGI Symbol;Acc:MGI:2149842] | Sdf2l1 | protein_coding |
| ENSMUSG00000022781 | 2495.6448 | 0.2465341 | 0.06460237 | 5.47E-05 | 0.00197501 | Up | 224105 | p21 (RAC1) activated kinase 2 [Source:MGI Symbol;Acc:MGI:1339984] | Pak2 | protein_coding |
| ENSMUSG00000022789 | 1641.46941 | 0.33613974 | 0.12166832 | 0.00129755 | 0.01570691 | Up | 74006 | dynamin 1-like [Source:MGI Symbol;Acc:MGI:1921256] | Dnm1l | protein_coding |
| ENSMUSG00000022811 | 1117.60455 | 0.36981064 | 0.12472323 | 0.00055608 | 0.00900355 | Up | 22661 | zinc finger protein 148 [Source:MGI Symbol;Acc:MGI:1332234] | Zfp148 | protein_coding |
| ENSMUSG00000022812 | 1925.46536 | 0.25036253 | 0.0942399 | 0.00255401 | 0.02434848 | Up | 56637 | glycogen synthase kinase 3 beta [Source:MGI Symbol;Acc:MGI:1861437] | Gsk3b | protein_coding |
| ENSMUSG00000022817 | 5658.50638 | -0.4577972 | 0.16966583 | 0.00087523 | 0.01232073 | Down | 16419 | integrin beta 5 [Source:MGI Symbol;Acc:MGI:96614] | Itgb5 | protein_coding |
| ENSMUSG00000022820 | 1022.49952 | -0.5985033 | 0.14643645 | 3.99E-06 | 0.00036162 | Down | 68194 | NADH:ubiquinone oxidoreductase subunit B4 [Source:MGI Symbol;Acc:MGI:1915444] | Ndufb4 | protein_coding |
| ENSMUSG00000022837 | 176.342315 | 0.41916493 | 0.15784556 | 0.00112685 | 0.01433253 | Up | 320299 | IQ calmodulin-binding motif containing 1 [Source:MGI Symbol;Acc:MGI:2443764] | Iqcb1 | protein_coding |
| ENSMUSG00000022848 | 1011.28766 | -0.2283591 | 0.07756545 | 0.00134855 | 0.01617956 | Down | 224132 | solute carrier family 49 member 4 [Source:MGI Symbol;Acc:MGI:2387188] | Slc49a4 | protein_coding |
| ENSMUSG00000022856 | 185.154874 | -0.4823368 | 0.17777457 | 0.00073604 | 0.01099993 | Down | 66664 | transmembrane protein 41a [Source:MGI Symbol;Acc:MGI:1913914] | Tmem41a | protein_coding |
| ENSMUSG00000022887 | 132.607372 | 1.02292792 | 0.44631291 | 0.00085899 | 0.0121448 | Up | 17174 | mannan-binding lectin serine peptidase 1 [Source:MGI Symbol;Acc:MGI:88492] | Masp1 | protein_coding |
| ENSMUSG00000022892 | 18669.3418 | -0.3638259 | 0.12734622 | 0.00082115 | 0.01177368 | Down | 11820 | amyloid beta (A4) precursor protein [Source:MGI Symbol;Acc:MGI:88059] | App | protein_coding |
| ENSMUSG00000022895 | 967.061091 | 0.37939614 | 0.19212478 | 0.00708402 | 0.04783037 | Up | 23872 | E26 avian leukemia oncogene 2, 3' domain [Source:MGI Symbol;Acc:MGI:95456] | Ets2 | protein_coding |
| ENSMUSG00000022897 | 815.149368 | 0.33246013 | 0.08889367 | 4.59E-05 | 0.00179583 | Up | 13548 | dual-specificity tyrosine-(Y)-phosphorylation regulated kinase 1a [Source:MGI Symbol;Acc:MGI:1330299] | Dyrk1a | protein_coding |
| ENSMUSG00000022951 | 639.499775 | -0.2432414 | 0.07957352 | 0.00090951 | 0.01260666 | Down | 54720 | regulator of calcineurin 1 [Source:MGI Symbol;Acc:MGI:1890564] | Rcan1 | protein_coding |
| ENSMUSG00000022974 | 903.837633 | 0.38207115 | 0.14594922 | 0.00158798 | 0.01782774 | Up | 67367 | PAX3 and PAX7 binding protein 1 [Source:MGI Symbol;Acc:MGI:1914617] | Paxbp1 | protein_coding |
| ENSMUSG00000022992 | 691.897504 | 0.3352195 | 0.09958904 | 0.00017949 | 0.00438461 | Up | 69612 | KAT8 regulatory NSL complex subunit 2 [Source:MGI Symbol;Acc:MGI:1916862] | Kansl2 | protein_coding |
| ENSMUSG00000023010 | 12443.6101 | -0.357177 | 0.09389852 | 3.30E-05 | 0.00147348 | Down | 110213 | transmembrane BAX inhibitor motif containing 6 [Source:MGI Symbol;Acc:MGI:99682] | Tmbim6 | protein_coding |
| ENSMUSG00000023020 | 754.745941 | -0.6904391 | 0.1942881 | 2.79E-05 | 0.00134946 | Down | 66379 | cytochrome c oxidase assembly protein 14 [Source:MGI Symbol;Acc:MGI:1913629] | Cox14 | protein_coding |
| ENSMUSG00000023025 | 1140.39522 | 0.50669464 | 0.12892806 | 1.09E-05 | 0.00073608 | Up | 207214 | La ribonucleoprotein domain family, member 4 [Source:MGI Symbol;Acc:MGI:2443114] | Larp4 | protein_coding |
| ENSMUSG00000023046 | 4600.57819 | -0.646985 | 0.28297634 | 0.00154861 | 0.01756789 | Down | 16012 | insulin-like growth factor binding protein 6 [Source:MGI Symbol;Acc:MGI:96441] | Igfbp6 | protein_coding |
| ENSMUSG00000023048 | 1044.35906 | -0.5734408 | 0.18294671 | 0.00016815 | 0.00418952 | Down | 66151 | proline rich 13 [Source:MGI Symbol;Acc:MGI:1913401] | Prr13 | protein_coding |
| ENSMUSG00000023064 | 13165.6407 | -1.5254553 | 0.2411625 | 1.50E-11 | 3.48E-08 | Down | 20618 | synuclein, gamma [Source:MGI Symbol;Acc:MGI:1298397] | Sncg | protein_coding |
| ENSMUSG00000023066 | 115.489474 | 0.37350803 | 0.1853934 | 0.00684503 | 0.04673877 | Up | 246102 | rotatin [Source:MGI Symbol;Acc:MGI:2179288] | Rttn | protein_coding |
| ENSMUSG00000023067 | 1536.86555 | -1.0225558 | 0.48800282 | 0.00126311 | 0.01538171 | Down | 12575 | cyclin-dependent kinase inhibitor 1A (P21) [Source:MGI Symbol;Acc:MGI:104556] | Cdkn1a | protein_coding |
| ENSMUSG00000023094 | 341.255574 | -0.479158 | 0.15625833 | 0.00025288 | 0.00549267 | Down | 76467 | methionine sulfoxide reductase B2 [Source:MGI Symbol;Acc:MGI:1923717] | Msrb2 | protein_coding |
| ENSMUSG00000023143 | 394.691954 | -0.3125684 | 0.13520479 | 0.00510769 | 0.03827231 | Down | 27426 | N-acetylglucosamine-1-phosphodiester alpha-N-acetylglucosaminidase [Source:MGI Symbol;Acc:MGI:1351598] | Nagpa | protein_coding |
| ENSMUSG00000023175 | 7255.3169 | -0.3420795 | 0.1313081 | 0.00130081 | 0.01573465 | Down | 12215 | basigin [Source:MGI Symbol;Acc:MGI:88208] | Bsg | protein_coding |
| ENSMUSG00000023206 | 583.917297 | 0.82547165 | 0.21235147 | 6.49E-06 | 0.00049007 | Up | 16169 | interleukin 15 receptor, alpha chain [Source:MGI Symbol;Acc:MGI:104644] | Il15ra | protein_coding |
| ENSMUSG00000023262 | 132.250675 | -0.3998011 | 0.17590437 | 0.00280756 | 0.02591412 | Down | 109652 | aminoacylase 1 [Source:MGI Symbol;Acc:MGI:87913] | Acy1 | protein_coding |

| | | | | | | | | | | |
|---|---|---|---|---|---|---|---|---|---|---|
| ENSMUSG00000023267 | 70.3766381 | -1.1498302 | 0.40372661 | 0.00019344 | 0.00456744 | Down | 14409 | gamma-aminobutyric acid (GABA) C receptor, subunit rho 2 [Source:MGI Symbol;Acc:MGI:95626] | Gabrr2 | protein_coding |
| ENSMUSG00000023277 | 521.130677 | -0.4624101 | 0.18616689 | 0.00148141 | 0.01711625 | Down | 23999 | twinfilin actin binding protein 2 [Source:MGI Symbol;Acc:MGI:1346078] | Twf2 | protein_coding |
| ENSMUSG00000023341 | 29.4834537 | 0.66877459 | 0.42595649 | 0.00512864 | 0.03837619 | Up | 17858 | MX dynamin-like GTPase 2 [Source:MGI Symbol;Acc:MGI:97244] | Mx2 | protein_coding |
| ENSMUSG00000023367 | 1010.04154 | -0.5158202 | 0.2485227 | 0.00314578 | 0.02792596 | Down | 66058 | transmembrane protein 176A [Source:MGI Symbol;Acc:MGI:1913308] | Tmem176a | protein_coding |
| ENSMUSG00000023456 | 7059.64355 | -0.6162708 | 0.23924662 | 0.00072219 | 0.01090021 | Down | 21991 | triosephosphate isomerase 1 [Source:MGI Symbol;Acc:MGI:98797] | Tpi1 | protein_coding |
| ENSMUSG00000023707 | 363.177314 | -0.5158055 | 0.18079654 | 0.00045747 | 0.00786417 | Down | 66627 | 2-oxoglutarate and iron-dependent oxygenase domain containing 2 [Source:MGI Symbol;Acc:MGI:1913877] | Ogfod2 | protein_coding |
| ENSMUSG00000023806 | 365.626291 | -0.3570434 | 0.1343257 | 0.00151796 | 0.01741052 | Down | 100037282 | radial spoke 3B homolog (Chlamydomonas) [Source:MGI Symbol;Acc:MGI:3630308] | Rsph3b | protein_coding |
| ENSMUSG00000023852 | 659.536439 | 0.38616387 | 0.13295236 | 0.00065168 | 0.01005376 | Up | 12648 | chromodomain helicase DNA binding protein 1 [Source:MGI Symbol;Acc:MGI:88393] | Chd1 | protein_coding |
| ENSMUSG00000023861 | 1784.97846 | 0.59157695 | 0.23301331 | 0.00089239 | 0.0124223 | Up | 55951 | mitochondrial pyruvate carrier 1 [Source:MGI Symbol;Acc:MGI:1915240] | Mpc1 | protein_coding |
| ENSMUSG00000023904 | 1249.59768 | -0.6609411 | 0.27545491 | 0.00102248 | 0.01346043 | Down | 353502 | host cell factor C1 regulator 1 (XPO1-dependent) [Source:MGI Symbol;Acc:MGI:2663619] | Hcfc1r1 | protein_coding |
| ENSMUSG00000023905 | 270.220134 | -0.900442 | 0.31786269 | 0.00023984 | 0.00530105 | Down | 27279 | tumor necrosis factor receptor superfamily, member 12a [Source:MGI Symbol;Acc:MGI:1351484] | Tnfrsf12a | protein_coding |
| ENSMUSG00000023912 | 84.4193343 | 0.56991272 | 0.26000829 | 0.00211381 | 0.02154256 | Up | 74011 | solute carrier family 25, member 27 [Source:MGI Symbol;Acc:MGI:1921261] | Slc25a27 | protein_coding |
| ENSMUSG00000023923 | 762.193268 | 0.3038168 | 0.11632543 | 0.00236441 | 0.02319431 | Up | 72238 | TBC1 domain family, member 5 [Source:MGI Symbol;Acc:MGI:1919488] | Tbc1d5 | protein_coding |
| ENSMUSG00000023939 | 411.427092 | -0.8696702 | 0.23788978 | 1.45E-05 | 0.00088154 | Down | 68463 | mitochondrial ribosomal protein L14 [Source:MGI Symbol;Acc:MGI:1333864] | Mrpl14 | protein_coding |
| ENSMUSG00000023961 | 756.816331 | 0.33371348 | 0.1208945 | 0.00131111 | 0.0158239 | Up | 224794 | ectonucleotide pyrophosphatase/phosphodiesterase 4 [Source:MGI Symbol;Acc:MGI:2682634] | Enpp4 | protein_coding |
| ENSMUSG00000023973 | 815.57577 | -0.3415958 | 0.11750818 | 0.00080116 | 0.01161263 | Down | 72029 | canopy FGF signaling regulator 3 [Source:MGI Symbol;Acc:MGI:1919279] | Cnpy3 | protein_coding |
| ENSMUSG00000024006 | 2059.75973 | 0.36200678 | 0.08270936 | 2.67E-06 | 0.0002824 | Up | 106504 | serine/threonine kinase 38 [Source:MGI Symbol;Acc:MGI:2442572] | Stk38 | protein_coding |
| ENSMUSG00000024012 | 3717.23136 | -0.6050245 | 0.14105597 | 1.64E-06 | 0.00019877 | Down | 56462 | mitochondrial carrier 1 [Source:MGI Symbol;Acc:MGI:1929261] | Mtch1 | protein_coding |
| ENSMUSG00000024030 | 859.632341 | -0.4456988 | 0.23871098 | 0.00610914 | 0.04307424 | Down | 11307 | ATP binding cassette subfamily G member 1 [Source:MGI Symbol;Acc:MGI:107704] | Abcg1 | protein_coding |
| ENSMUSG00000024038 | 1656.4842 | -0.4945152 | 0.19671685 | 0.00124145 | 0.0152436 | Down | 78330 | NADH:ubiquinone oxidoreductase core subunit V3 [Source:MGI Symbol;Acc:MGI:1890894] | Ndufv3 | protein_coding |
| ENSMUSG00000024039 | 155.198924 | -0.6516338 | 0.3017091 | 0.00182377 | 0.01955503 | Down | 12411 | cystathionine beta-synthase [Source:MGI Symbol;Acc:MGI:88285] | Cbs | protein_coding |
| ENSMUSG00000024045 | 1131.52379 | 0.45445109 | 0.10182597 | 1.22E-06 | 0.0001654 | Up | 56399 | A kinase (PRKA) anchor protein 8 [Source:MGI Symbol;Acc:MGI:1928488] | Akap8 | protein_coding |
| ENSMUSG00000024048 | 2294.37844 | -0.7621463 | 0.19100574 | 4.54E-06 | 0.00038448 | Down | 67268 | myosin, light chain 12A, regulatory, non-sarcomeric [Source:MGI Symbol;Acc:MGI:1914518] | Myl12a | protein_coding |
| ENSMUSG00000024053 | 2604.64498 | -0.6205435 | 0.24675071 | 0.00088299 | 0.01238632 | Down | 246707 | elastin microfibril interfacer 2 [Source:MGI Symbol;Acc:MGI:2389136] | Emilin2 | protein_coding |
| ENSMUSG00000024096 | 1645.48134 | -0.1679962 | 0.05078567 | 0.00060579 | 0.00958229 | Down | 19765 | ralA binding protein 1 [Source:MGI Symbol;Acc:MGI:108466] | Ralbp1 | protein_coding |
| ENSMUSG00000024101 | 642.568109 | -0.3942994 | 0.10532739 | 3.12E-05 | 0.00144258 | Down | 68767 | WASH complex subunit 1 [Source:MGI Symbol;Acc:MGI:1916017] | Washc1 | protein_coding |
| ENSMUSG00000024150 | 2447.13943 | -0.3449964 | 0.14356641 | 0.0031242 | 0.02784016 | Down | 193813 | multiple coagulation factor deficiency 2 [Source:MGI Symbol;Acc:MGI:2183439] | Mcfd2 | protein_coding |
| ENSMUSG00000024165 | 965.50824 | -0.8223824 | 0.22268767 | 1.39E-05 | 0.00086056 | Down | 52009 | Jupiter microtubule associated homolog 2 [Source:MGI Symbol;Acc:MGI:1196260] | Jpt2 | protein_coding |
| ENSMUSG00000024177 | 63.0668329 | -0.9739907 | 0.32138037 | 0.00011901 | 0.00328649 | Down | 56520 | NME/NM23 nucleoside diphosphate kinase 4 [Source:MGI Symbol;Acc:MGI:1931148] | Nme4 | protein_coding |
| ENSMUSG00000024181 | 741.923934 | -0.6031997 | 0.18935303 | 0.00012467 | 0.00338386 | Down | 68611 | mitochondrial ribosomal protein L28 [Source:MGI Symbol;Acc:MGI:1915861] | Mrpl28 | protein_coding |
| ENSMUSG00000024191 | 274.875437 | -0.2563798 | 0.11285393 | 0.00749253 | 0.04964955 | Down | 224630 | BCL2/adenovirus E1B interacting protein 1 [Source:MGI Symbol;Acc:MGI:109328] | Bnip1 | protein_coding |
| ENSMUSG00000024197 | 1284.48603 | -0.3540123 | 0.14139817 | 0.00234274 | 0.02305135 | Down | 66905 | perilipin 3 [Source:MGI Symbol;Acc:MGI:1914155] | Plin3 | protein_coding |
| ENSMUSG00000024208 | 351.71285 | -0.7248025 | 0.17893765 | 4.01E-06 | 0.00036162 | Down | 67267 | ubiquinol-cytochrome c reductase complex assembly factor 2 [Source:MGI Symbol;Acc:MGI:1914517] | Uqcc2 | protein_coding |
| ENSMUSG00000024213 | 754.628069 | -0.4615721 | 0.19178757 | 0.00180365 | 0.01940435 | Down | 56409 | nudix (nucleotide diphosphate linked moiety X)-type motif 3 [Source:MGI Symbol;Acc:MGI:1928484] | Nudt3 | protein_coding |
| ENSMUSG00000024217 | 434.162986 | -0.348393 | 0.16406593 | 0.00594576 | 0.04227086 | Down | 20630 | U1 small nuclear ribonucleoprotein C [Source:MGI Symbol;Acc:MGI:109489] | Snrpc | protein_coding |
| ENSMUSG00000024228 | 624.372707 | 0.36328312 | 0.14672955 | 0.00236134 | 0.02317822 | Up | 67993 | nudix (nucleoside diphosphate linked moiety X)-type motif 12 [Source:MGI Symbol;Acc:MGI:1915243] | Nudt12 | protein_coding |
| ENSMUSG00000024234 | 642.760083 | 0.2866966 | 0.11222013 | 0.00311918 | 0.02782324 | Up | 67440 | mitochondrial poly(A) polymerase [Source:MGI Symbol;Acc:MGI:1914690] | Mtpap | protein_coding |
| ENSMUSG00000024238 | 965.907092 | 0.37321629 | 0.132526 | 0.00089654 | 0.01245871 | Up | 21417 | zinc finger E-box binding homeobox 1 [Source:MGI Symbol;Acc:MGI:1344313] | Zeb1 | protein_coding |
| ENSMUSG00000024240 | 572.195028 | 0.45093344 | 0.14431864 | 0.00024045 | 0.00530727 | Up | 13831 | enhancer of polycomb homolog 1 [Source:MGI Symbol;Acc:MGI:1278322] | Epc1 | protein_coding |
| ENSMUSG00000024241 | 715.023284 | 0.5518062 | 0.14289106 | 1.23E-05 | 0.00078933 | Up | 20662 | SOS Ras/Rac guanine nucleotide exchange factor 1 [Source:MGI Symbol;Acc:MGI:98354] | Sos1 | protein_coding |
| ENSMUSG00000024242 | 1370.98625 | 0.26996875 | 0.10601926 | 0.00363637 | 0.03060877 | Up | 225028 | mitogen-activated protein kinase kinase kinase kinase 3 [Source:MGI Symbol;Acc:MGI:2154405] | Map4k3 | protein_coding |
| ENSMUSG00000024248 | 2072.6683 | -0.4327376 | 0.15892405 | 0.00091437 | 0.01266315 | Down | 20463 | cytochrome c oxidase subunit 7A2 like [Source:MGI Symbol;Acc:MGI:106015] | Cox7a2l | protein_coding |
| ENSMUSG00000024258 | 338.658064 | -0.5881536 | 0.15315367 | 1.16E-05 | 0.00075871 | Down | 69241 | polymerase (RNA) II (DNA directed) polypeptide D [Source:MGI Symbol;Acc:MGI:1916491] | Polr2d | protein_coding |
| ENSMUSG00000024276 | 647.427854 | 0.4064855 | 0.17706029 | 0.00293652 | 0.02662691 | Up | 69256 | zinc finger protein 397 [Source:MGI Symbol;Acc:MGI:1916506] | Zfp397 | protein_coding |
| ENSMUSG00000024283 | 1560.01708 | 0.29801786 | 0.10636115 | 0.00142813 | 0.01677936 | Up | 225131 | WW domain containing adaptor with coiled-coil [Source:MGI Symbol;Acc:MGI:2387357] | Wac | protein_coding |
| ENSMUSG00000024292 | 16.7364644 | -1.0311864 | 0.58110388 | 0.00235276 | 0.02313599 | Down | 64385 | cytochrome P450, family 4, subfamily f, polypeptide 14 [Source:MGI Symbol;Acc:MGI:1927669] | Cyp4f14 | protein_coding |
| ENSMUSG00000024294 | 1651.71133 | 0.35158687 | 0.0923233 | 3.24E-05 | 0.00145985 | Up | 225164 | MIB E3 ubiquitin protein ligase 1 [Source:MGI Symbol;Acc:MGI:2443157] | Mib1 | protein_coding |
| ENSMUSG00000024308 | 5952.38009 | -0.394264 | 0.14831366 | 0.00109658 | 0.01410443 | Down | 21356 | TAP binding protein [Source:MGI Symbol;Acc:MGI:1201689] | Tapbp | protein_coding |
| ENSMUSG00000024309 | 330.746714 | -0.7542437 | 0.35870538 | 0.00171453 | 0.01877503 | Down | 14976 | prefoldin subunit 6 [Source:MGI Symbol;Acc:MGI:95908] | Pfdn6 | protein_coding |
| ENSMUSG00000024317 | 348.260015 | 0.4182991 | 0.11313542 | 3.56E-05 | 0.00153347 | Up | 56515 | ring finger protein 138 [Source:MGI Symbol;Acc:MGI:1929211] | Rnf138 | protein_coding |

| | | | | | | | | | | |
|---|---|---|---|---|---|---|---|---|---|---|
| ENSMUSG00000024338 | 433.368882 | -0.5476307 | 0.32360041 | 0.00556365 | 0.04054803 | Down | 16913 | proteasome (prosome, macropain) subunit, beta type 8 (large multifunctional peptidase 7) [Source:MGI Symbol;Acc:MGI:1346527] | Psmb8 | protein_coding |
| ENSMUSG00000024357 | 1011.33842 | -0.4327891 | 0.14131049 | 0.00029881 | 0.00610744 | Down | 81500 | endoplasmic reticulum chaperone SIL1 homolog (S. cerevisiae) [Source:MGI Symbol;Acc:MGI:1932040] | Sil1 | protein_coding |
| ENSMUSG00000024369 | 649.342202 | -0.36737 | 0.14011497 | 0.00150707 | 0.01732646 | Down | 27632 | negative elongation factor complex member E, Rdbp [Source:MGI Symbol;Acc:MGI:102744] | Nelfe | protein_coding |
| ENSMUSG00000024414 | 618.266671 | -0.4704601 | 0.17870397 | 0.00098775 | 0.01327212 | Down | 94064 | mitochondrial ribosomal protein L27 [Source:MGI Symbol;Acc:MGI:2137224] | Mrpl27 | protein_coding |
| ENSMUSG00000024422 | 767.572079 | -0.2390263 | 0.08696322 | 0.00240834 | 0.02344123 | Down | 69192 | DEAH (Asp-Glu-Ala-His) box polypeptide 16 [Source:MGI Symbol;Acc:MGI:1916442] | Dhx16 | protein_coding |
| ENSMUSG00000024429 | 826.32719 | -0.4365375 | 0.1404357 | 0.00025364 | 0.00549267 | Down | 14670 | guanine nucleotide binding protein-like 1 [Source:MGI Symbol;Acc:MGI:95764] | Gnl1 | protein_coding |
| ENSMUSG00000024431 | 4066.29443 | 0.30342413 | 0.13869996 | 0.00698473 | 0.04739641 | Up | 14815 | nuclear receptor subfamily 3, group C, member 1 [Source:MGI Symbol;Acc:MGI:95824] | Nr3c1 | protein_coding |
| ENSMUSG00000024446 | 156.287944 | -0.6998221 | 0.25543262 | 0.00039124 | 0.00719432 | Down | 67676 | ribonuclease P 21 subunit [Source:MGI Symbol;Acc:MGI:1914926] | Rpp21 | protein_coding |
| ENSMUSG00000024491 | 533.197301 | 0.46076707 | 0.1226277 | 2.42E-05 | 0.00120579 | Up | 225432 | RNA binding motif protein 27 [Source:MGI Symbol;Acc:MGI:2147194] | Rbm27 | protein_coding |
| ENSMUSG00000024533 | 704.766914 | 0.36020989 | 0.15585208 | 0.00376225 | 0.03130672 | Up | 68166 | spire type actin nucleation factor 1 [Source:MGI Symbol;Acc:MGI:1915416] | Spire1 | protein_coding |
| ENSMUSG00000024538 | 989.736439 | -0.6124898 | 0.25795581 | 0.00136966 | 0.0163561 | Down | 19038 | peptidylprolyl isomerase C [Source:MGI Symbol;Acc:MGI:97751] | Ppic | protein_coding |
| ENSMUSG00000024570 | 505.214548 | -0.3441163 | 0.16665299 | 0.007114 | 0.0479133 | Down | 68731 | ribosome binding factor A [Source:MGI Symbol;Acc:MGI:1915981] | Rbfa | protein_coding |
| ENSMUSG00000024580 | 242.380698 | 0.27728325 | 0.11019378 | 0.00361252 | 0.03050175 | Up | 17714 | GrpE-like 2, mitochondrial [Source:MGI Symbol;Acc:MGI:1334416] | Grpel2 | protein_coding |
| ENSMUSG00000024589 | 757.756815 | -0.3892237 | 0.17823449 | 0.00424116 | 0.03387298 | Down | 83814 | neural precursor cell expressed, developmentally down-regulated gene 4-like [Source:MGI Symbol;Acc:MGI:1933754] | Nedd4l | protein_coding |
| ENSMUSG00000024608 | 6545.53833 | -0.4525519 | 0.19129278 | 0.00204295 | 0.02108493 | Down | 20044 | ribosomal protein S14 [Source:MGI Symbol;Acc:MGI:98107] | Rps14 | protein_coding |
| ENSMUSG00000024639 | 2176.97284 | 0.33715061 | 0.11117539 | 0.00056131 | 0.00906407 | Up | 14682 | guanine nucleotide binding protein, alpha q polypeptide [Source:MGI Symbol;Acc:MGI:95776] | Gnaq | protein_coding |
| ENSMUSG00000024644 | 1801.34899 | -0.3467032 | 0.16120928 | 0.0062709 | 0.04392872 | Down | 66054 | CNDP dipeptidase 2 (metallopeptidase M20 family) [Source:MGI Symbol;Acc:MGI:1913304] | Cndp2 | protein_coding |
| ENSMUSG00000024646 | 7065.91212 | -0.3166538 | 0.11704769 | 0.00161087 | 0.01798528 | Down | 109672 | cytochrome b5 type A (microsomal) [Source:MGI Symbol;Acc:MGI:1926952] | Cyb5a | protein_coding |
| ENSMUSG00000024659 | 10172.3038 | -0.7419956 | 0.21944212 | 4.81E-05 | 0.0018569 | Down | 16952 | annexin A1 [Source:MGI Symbol;Acc:MGI:96819] | Anxa1 | protein_coding |
| ENSMUSG00000024664 | 12022.4531 | -0.4493782 | 0.18682943 | 0.00201152 | 0.02088341 | Down | 60527 | fatty acid desaturase 3 [Source:MGI Symbol;Acc:MGI:1928740] | Fads3 | protein_coding |
| ENSMUSG00000024725 | 1444.47614 | -0.4588343 | 0.2041323 | 0.0026327 | 0.02480871 | Down | 20409 | osteoclast stimulating factor 1 [Source:MGI Symbol;Acc:MGI:700012] | Ostf1 | protein_coding |
| ENSMUSG00000024736 | 203.951803 | -0.5504973 | 0.31929327 | 0.00530397 | 0.03935283 | Down | 98170 | transmembrane protein 132A [Source:MGI Symbol;Acc:MGI:2147810] | Tmem132a | protein_coding |
| ENSMUSG00000024754 | 192.73775 | 0.50358409 | 0.23066825 | 0.00263282 | 0.02480871 | Up | 83921 | cell migration inducing hyaluronidase 2 [Source:MGI Symbol;Acc:MGI:1890373] | Cemip2 | protein_coding |
| ENSMUSG00000024759 | 5837.58526 | 0.31163449 | 0.09474508 | 0.00026857 | 0.00569075 | Up | 109168 | atlastin GTPase 3 [Source:MGI Symbol;Acc:MGI:1924270] | Atl3 | protein_coding |
| ENSMUSG00000024767 | 869.135994 | -0.2896557 | 0.1168213 | 0.0041408 | 0.03333364 | Down | 107260 | OTU domain, ubiquitin aldehyde binding 1 [Source:MGI Symbol;Acc:MGI:2147616] | Otub1 | protein_coding |
| ENSMUSG00000024768 | 69.7999996 | -1.4142515 | 0.80284384 | 0.0018249 | 0.01955503 | Down | 67717 | lipase, gastric [Source:MGI Symbol;Acc:MGI:1914967] | Lipf | protein_coding |
| ENSMUSG00000024780 | 1258.70551 | 0.37104711 | 0.12037575 | 0.00039193 | 0.00719432 | Up | 67072 | cell division cycle 37-like 1 [Source:MGI Symbol;Acc:MGI:1914322] | Cdc37l1 | protein_coding |
| ENSMUSG00000024790 | 208.393128 | -1.1128306 | 0.17787156 | 2.37E-11 | 4.27E-08 | Down | 66406 | SAC3 domain containing 1 [Source:MGI Symbol;Acc:MGI:1913656] | Sac3d1 | protein_coding |
| ENSMUSG00000024791 | 9.67455548 | -1.2724746 | 0.68528237 | 0.00196035 | 0.02050607 | Down | 67849 | cell division cycle associated 5 [Source:MGI Symbol;Acc:MGI:1915099] | Cdca5 | protein_coding |
| ENSMUSG00000024811 | 3022.70558 | 0.27958648 | 0.1079143 | 0.00293298 | 0.02661802 | Up | 74493 | tankyrase, TRF1-interacting ankyrin-related ADP-ribose polymerase 2 [Source:MGI Symbol;Acc:MGI:1921743] | Tnks2 | protein_coding |
| ENSMUSG00000024816 | 601.270306 | -0.2665314 | 0.08572341 | 0.00064175 | 0.00993832 | Down | 67457 | FERM domain containing 8 [Source:MGI Symbol;Acc:MGI:1914707] | Frmd8 | protein_coding |
| ENSMUSG00000024844 | 527.337713 | -0.5274013 | 0.19728197 | 0.00073931 | 0.01101595 | Down | 23825 | BAF nuclear assembly factor 1 [Source:MGI Symbol;Acc:MGI:1346330] | Banf1 | protein_coding |
| ENSMUSG00000024847 | 1080.81263 | -0.3846594 | 0.14332352 | 0.00120465 | 0.01500733 | Down | 11632 | aryl-hydrocarbon receptor-interacting protein [Source:MGI Symbol;Acc:MGI:109622] | Aip | protein_coding |
| ENSMUSG00000024854 | 857.85414 | -0.5737424 | 0.15911054 | 2.96E-05 | 0.00140942 | Down | 69745 | polymerase (DNA-directed), delta 4 [Source:MGI Symbol;Acc:MGI:1916995] | Pold4 | protein_coding |
| ENSMUSG00000024856 | 1140.16564 | -0.3372894 | 0.14062654 | 0.00340311 | 0.02929847 | Down | 52004 | CDK2-associated protein 2 [Source:MGI Symbol;Acc:MGI:1098779] | Cdk2ap2 | protein_coding |
| ENSMUSG00000024875 | 1040.29488 | -0.3205868 | 0.10899375 | 0.00079941 | 0.01161263 | Down | 68090 | Yip1 interacting factor homolog A (S. cerevisiae) [Source:MGI Symbol;Acc:MGI:1915340] | Yif1a | protein_coding |
| ENSMUSG00000024885 | 347.19927 | -0.8937818 | 0.26936354 | 5.00E-05 | 0.00187013 | Down | 67689 | aldehyde dehydrogenase 3 family, member B1 [Source:MGI Symbol;Acc:MGI:1914939] | Aldh3b1 | protein_coding |
| ENSMUSG00000024887 | 731.854646 | 0.4212592 | 0.15339134 | 0.00087457 | 0.01232073 | Up | 54447 | N-acylsphingosine amidohydrolase 2 [Source:MGI Symbol;Acc:MGI:1859310] | Asah2 | protein_coding |
| ENSMUSG00000024896 | 646.308099 | -0.2764703 | 0.09876913 | 0.00157975 | 0.01778474 | Down | 17330 | multiple inositol polyphosphate histidine phosphatase 1 [Source:MGI Symbol;Acc:MGI:1336159] | Minpp1 | protein_coding |
| ENSMUSG00000024902 | 653.806807 | -0.5153739 | 0.19736135 | 0.00089095 | 0.0124223 | Down | 66419 | mitochondrial ribosomal protein L11 [Source:MGI Symbol;Acc:MGI:2137215] | Mrpl11 | protein_coding |
| ENSMUSG00000024908 | 1784.21703 | 0.3714173 | 0.10330046 | 6.52E-05 | 0.00221232 | Up | 52036 | protein phosphatase 6, regulatory subunit 3 [Source:MGI Symbol;Acc:MGI:1921807] | Ppp6r3 | protein_coding |
| ENSMUSG00000024925 | 267.407906 | -0.5630884 | 0.30754146 | 0.00432139 | 0.03426111 | Down | 68209 | ribonuclease H2, subunit C [Source:MGI Symbol;Acc:MGI:1915459] | Rnaseh2c | protein_coding |
| ENSMUSG00000024940 | 9764.24669 | -0.3748249 | 0.16108968 | 0.00329971 | 0.02878827 | Down | 16998 | latent transforming growth factor beta binding protein 3 [Source:MGI Symbol;Acc:MGI:1101355] | Ltbp3 | protein_coding |
| ENSMUSG00000024944 | 633.986176 | -0.356141 | 0.15953357 | 0.00457522 | 0.03556782 | Down | 56327 | ADP-ribosylation factor-like 2 [Source:MGI Symbol;Acc:MGI:1928393] | Arl2 | protein_coding |
| ENSMUSG00000024953 | 3985.65838 | -0.8937665 | 0.24434309 | 1.66E-05 | 0.00095798 | Down | 54683 | peroxiredoxin 5 [Source:MGI Symbol;Acc:MGI:1859821] | Prdx5 | protein_coding |
| ENSMUSG00000024959 | 426.172195 | -0.6177049 | 0.15583374 | 6.79E-06 | 0.00050796 | Down | 12015 | BCL2-associated agonist of cell death [Source:MGI Symbol;Acc:MGI:1096330] | Bad | protein_coding |
| ENSMUSG00000024972 | 5275.43333 | 0.44793086 | 0.19628374 | 0.00251866 | 0.02413899 | Up | 56072 | lectin, galactose binding, soluble 12 [Source:MGI Symbol;Acc:MGI:1929094] | Lgals12 | protein_coding |
| ENSMUSG00000024976 | 1038.47394 | 0.3100178 | 0.12688015 | 0.00358752 | 0.03034741 | Up | 56392 | Shoc2, leucine rich repeat scaffold protein [Source:MGI Symbol;Acc:MGI:1927197] | Shoc2 | protein_coding |
| ENSMUSG00000024981 | 1204.12464 | -0.2315318 | 0.08891237 | 0.00408784 | 0.03298905 | Down | 433256 | acyl-CoA synthetase long-chain family member 5 [Source:MGI Symbol;Acc:MGI:1919129] | Acsl5 | protein_coding |
| ENSMUSG00000025035 | 377.023491 | -0.6949944 | 0.26842196 | 0.00059359 | 0.00944449 | Down | 56350 | ADP-ribosylation factor-like 3 [Source:MGI Symbol;Acc:MGI:1929699] | Arl3 | protein_coding |
| ENSMUSG00000025037 | 1086.59294 | -0.4973527 | 0.2209973 | 0.00228338 | 0.02259048 | Down | 17161 | monoamine oxidase A [Source:MGI Symbol;Acc:MGI:96915] | Maoa | protein_coding |

| | | | | | | | | | | |
|---|---|---|---|---|---|---|---|---|---|---|
| ENSMUSG00000025094 | 34.4046722 | 0.65582445 | 0.37185431 | 0.00396168 | 0.0324056 | Up | 214084 | solute carrier family 18 (vesicular monoamine), member 2 [Source:MGI Symbol;Acc:MGI:106677] | Slc18a2 | protein_coding |
| ENSMUSG00000025102 | 309.587902 | -0.37622 | 0.15039758 | 0.00210329 | 0.02146226 | Down | 67290 | RIKEN cDNA 3110040N11 gene [Source:MGI Symbol;Acc:MGI:1914540] | 3110040N11Rik | protein_coding |
| ENSMUSG00000025104 | 319.59687 | 0.43179399 | 0.15143311 | 0.00061305 | 0.0096222 | Up | 29877 | HDGF like 3 [Source:MGI Symbol;Acc:MGI:1352760] | Hdgfl3 | protein_coding |
| ENSMUSG00000025130 | 8554.00419 | -0.3145766 | 0.09548266 | 0.00026036 | 0.00556603 | Down | 18453 | prolyl 4-hydroxylase, beta polypeptide [Source:MGI Symbol;Acc:MGI:97464] | P4hb | protein_coding |
| ENSMUSG00000025132 | 6590.91289 | -0.3586681 | 0.11608094 | 0.00046168 | 0.00789467 | Down | 192662 | Rho GDP dissociation inhibitor (GDI) alpha [Source:MGI Symbol;Acc:MGI:2178103] | Arhgdia | protein_coding |
| ENSMUSG00000025135 | 774.842338 | -0.5820832 | 0.15759116 | 1.84E-05 | 0.00102462 | Down | 66156 | anaphase promoting complex subunit 11 [Source:MGI Symbol;Acc:MGI:1913406] | Anapc11 | protein_coding |
| ENSMUSG00000025140 | 65.9781704 | -0.7337883 | 0.23533093 | 0.00012213 | 0.00334573 | Down | 209027 | pyrroline-5-carboxylate reductase 1 [Source:MGI Symbol;Acc:MGI:2384795] | Pycr1 | protein_coding |
| ENSMUSG00000025153 | 179956.449 | 0.8451132 | 0.36680215 | 0.00099697 | 0.01330743 | Up | 14104 | fatty acid synthase [Source:MGI Symbol;Acc:MGI:95485] | Fasn | protein_coding |
| ENSMUSG00000025171 | 393.444829 | -0.5228632 | 0.22301001 | 0.00168017 | 0.01851726 | Down | 226122 | ubiquitin domain containing 1 [Source:MGI Symbol;Acc:MGI:2385092] | Ubtd1 | protein_coding |
| ENSMUSG00000025188 | 508.407011 | -0.2903501 | 0.11873169 | 0.0040211 | 0.03269408 | Down | 192236 | HPS1, biogenesis of lysosomal organelles complex 3 subunit 1 [Source:MGI Symbol;Acc:MGI:2177763] | Hps1 | protein_coding |
| ENSMUSG00000025199 | 1060.7056 | 0.3742116 | 0.10614214 | 8.42E-05 | 0.00261938 | Up | 12675 | conserved helix-loop-helix ubiquitous kinase [Source:MGI Symbol;Acc:MGI:99484] | Chuk | protein_coding |
| ENSMUSG00000025208 | 608.275628 | -0.3187901 | 0.13629124 | 0.00434341 | 0.03438536 | Down | 94067 | mitochondrial ribosomal protein L43 [Source:MGI Symbol;Acc:MGI:2137229] | Mrpl43 | protein_coding |
| ENSMUSG00000025218 | 230.979097 | -0.4500807 | 0.14200721 | 0.00020888 | 0.00477922 | Down | 56626 | polymerase (DNA directed), lambda [Source:MGI Symbol;Acc:MGI:1889000] | Poll | protein_coding |
| ENSMUSG00000025225 | 682.248441 | -0.3553935 | 0.14349034 | 0.00249265 | 0.02401966 | Down | 18034 | nuclear factor of kappa light polypeptide gene enhancer in B cells 2, p49/p100 [Source:MGI Symbol;Acc:MGI:1099800] | Nfkb2 | protein_coding |
| ENSMUSG00000025226 | 96.0025508 | -0.7043792 | 0.27630889 | 0.00065479 | 0.0100634 | Down | 68431 | F-box and leucine-rich repeat protein 15 [Source:MGI Symbol;Acc:MGI:1915681] | Fbxl15 | protein_coding |
| ENSMUSG00000025228 | 3260.59875 | -0.2257885 | 0.09015682 | 0.00523737 | 0.03893868 | Down | 54130 | ARP1 actin-related protein 1A, centractin alpha [Source:MGI Symbol;Acc:MGI:1858964] | Actr1a | protein_coding |
| ENSMUSG00000025234 | 2220.12108 | 0.26151966 | 0.10378676 | 0.00429041 | 0.0341161 | Up | 23806 | ariadne RBR E3 ubiquitin protein ligase 1 [Source:MGI Symbol;Acc:MGI:1344363] | Arih1 | protein_coding |
| ENSMUSG00000025268 | 612.071624 | -0.4456183 | 0.11939292 | 3.24E-05 | 0.00145985 | Down | 80884 | MAGE family member D2 [Source:MGI Symbol;Acc:MGI:1933391] | Maged2 | protein_coding |
| ENSMUSG00000025280 | 344.226933 | 0.3309852 | 0.13642963 | 0.00319518 | 0.02819979 | Up | 218832 | polymerase (RNA) III (DNA directed) polypeptide A [Source:MGI Symbol;Acc:MGI:2681836] | Polr3a | protein_coding |
| ENSMUSG00000025323 | 115.883857 | 0.53362876 | 0.18245983 | 0.00034729 | 0.00670832 | Up | 20688 | trans-acting transcription factor 4 [Source:MGI Symbol;Acc:MGI:107595] | Sp4 | protein_coding |
| ENSMUSG00000025326 | 1367.77617 | 0.28031253 | 0.11949728 | 0.00543545 | 0.03993618 | Up | 22215 | ubiquitin protein ligase E3A [Source:MGI Symbol;Acc:MGI:105098] | Ube3a | protein_coding |
| ENSMUSG00000025337 | 1808.46666 | -0.2528441 | 0.09527657 | 0.00287853 | 0.02637436 | Down | 66711 | SBDS ribosome maturation factor [Source:MGI Symbol;Acc:MGI:1913961] | Sbds | protein_coding |
| ENSMUSG00000025348 | 4568.67439 | -0.3908881 | 0.20473 | 0.00744969 | 0.04953752 | Down | 16404 | integrin alpha 7 [Source:MGI Symbol;Acc:MGI:102700] | Itga7 | protein_coding |
| ENSMUSG00000025357 | 404.002794 | 0.38453778 | 0.12723916 | 0.00044775 | 0.00775086 | Up | 13139 | diacylglycerol kinase, alpha [Source:MGI Symbol;Acc:MGI:102952] | Dgka | protein_coding |
| ENSMUSG00000025362 | 4022.79868 | -0.751153 | 0.24201705 | 0.00011917 | 0.00328649 | Down | 27370 | ribosomal protein S26 [Source:MGI Symbol;Acc:MGI:1351628] | Rps26 | protein_coding |
| ENSMUSG00000025366 | 8975.76026 | -0.3381158 | 0.10515644 | 0.00026931 | 0.00569518 | Down | 23943 | extended synaptotagmin-like protein 1 [Source:MGI Symbol;Acc:MGI:1344426] | Esyt1 | protein_coding |
| ENSMUSG00000025371 | 362.607844 | -0.5742988 | 0.22036055 | 0.00073736 | 0.01100029 | Down | 208092 | charged multivesicular body protein 6 [Source:MGI Symbol;Acc:MGI:3583942] | Chmp6 | protein_coding |
| ENSMUSG00000025403 | 429.711059 | -0.3637532 | 0.10966691 | 0.00017806 | 0.00435971 | Down | 108037 | serine hydroxymethyltransferase 2 (mitochondrial) [Source:MGI Symbol;Acc:MGI:1277989] | Shmt2 | protein_coding |
| ENSMUSG00000025437 | 699.573567 | 0.42634547 | 0.18621768 | 0.00282913 | 0.02605398 | Up | 170822 | ubiquitin specific peptidase 33 [Source:MGI Symbol;Acc:MGI:2159711] | Usp33 | protein_coding |
| ENSMUSG00000025485 | 1040.61164 | -0.302901 | 0.10869976 | 0.00140308 | 0.01661296 | Down | 101489 | RIC8 guanine nucleotide exchange factor A [Source:MGI Symbol;Acc:MGI:2141866] | Ric8a | protein_coding |
| ENSMUSG00000025499 | 638.902372 | -0.4369735 | 0.14739305 | 0.00042118 | 0.0074724 | Down | 15461 | Harvey rat sarcoma virus oncogene [Source:MGI Symbol;Acc:MGI:96224] | Hras | protein_coding |
| ENSMUSG00000025508 | 6083.34874 | -0.6552427 | 0.18791663 | 3.83E-05 | 0.00161218 | Down | 67186 | ribosomal protein, large P2 [Source:MGI Symbol;Acc:MGI:1914436] | Rplp2 | protein_coding |
| ENSMUSG00000025510 | 7259.24621 | -0.4277462 | 0.09826593 | 2.19E-06 | 0.00024723 | Down | 12476 | CD151 antigen [Source:MGI Symbol;Acc:MGI:1096360] | Cd151 | protein_coding |
| ENSMUSG00000025512 | 675.783074 | -0.5702471 | 0.14646865 | 9.98E-06 | 0.00069605 | Down | 68038 | chitinase domain containing 1 [Source:MGI Symbol;Acc:MGI:1915288] | Chid1 | protein_coding |
| ENSMUSG00000025534 | 1794.10058 | -0.4192771 | 0.15081383 | 0.00079207 | 0.01154006 | Down | 110006 | glucuronidase, beta [Source:MGI Symbol;Acc:MGI:95872] | Gusb | protein_coding |
| ENSMUSG00000025558 | 2138.31989 | 0.55717572 | 0.21234085 | 0.00073982 | 0.01101595 | Up | 105445 | dedicator of cytokinesis 9 [Source:MGI Symbol;Acc:MGI:106321] | Dock9 | protein_coding |
| ENSMUSG00000025572 | 486.005675 | -0.4020369 | 0.17474768 | 0.0030478 | 0.02732424 | Down | 217353 | transmembrane channel-like gene family 6 [Source:MGI Symbol;Acc:MGI:1098686] | Tmc6 | protein_coding |
| ENSMUSG00000025578 | 123.877537 | -0.624197 | 0.30808725 | 0.00255811 | 0.02435899 | Down | 30951 | chromobox 8 [Source:MGI Symbol;Acc:MGI:1353589] | Cbx8 | protein_coding |
| ENSMUSG00000025586 | 887.487023 | 0.29153717 | 0.12485632 | 0.00535815 | 0.03955973 | Up | 12877 | cytoplasmic polyadenylation element binding protein 1 [Source:MGI Symbol;Acc:MGI:108442] | Cpeb1 | protein_coding |
| ENSMUSG00000025609 | 1530.84116 | 0.30991198 | 0.0957895 | 0.00030477 | 0.00615863 | Up | 27418 | muskelin 1, intracellular mediator containing kelch motifs [Source:MGI Symbol;Acc:MGI:1351638] | Mkln1 | protein_coding |
| ENSMUSG00000025666 | 171.786841 | 0.50358937 | 0.20089578 | 0.0012383 | 0.01523671 | Up | 192216 | transmembrane protein 47 [Source:MGI Symbol;Acc:MGI:2177570] | Tmem47 | protein_coding |
| ENSMUSG00000025724 | 759.620478 | -0.3855262 | 0.12128106 | 0.0002504 | 0.00545876 | Down | 56529 | SEC11 homolog A, signal peptidase complex subunit [Source:MGI Symbol;Acc:MGI:1929464] | Sec11a | protein_coding |
| ENSMUSG00000025758 | 142.795165 | 0.31949236 | 0.14993484 | 0.00687128 | 0.04686141 | Up | 20873 | polo like kinase 4 [Source:MGI Symbol;Acc:MGI:101783] | Plk4 | protein_coding |
| ENSMUSG00000025764 | 1546.89115 | 0.3262276 | 0.14602018 | 0.00548558 | 0.0401592 | Up | 269424 | jade family PHD finger 1 [Source:MGI Symbol;Acc:MGI:1925835] | Jade1 | protein_coding |
| ENSMUSG00000025784 | 2872.19356 | -0.7039511 | 0.18422732 | 9.98E-06 | 0.00069605 | Down | 21922 | C-type lectin domain family 3, member b [Source:MGI Symbol;Acc:MGI:104540] | Clec3b | protein_coding |
| ENSMUSG00000025794 | 5168.40389 | -0.3792246 | 0.1701201 | 0.00438396 | 0.03448783 | Down | 67115 | ribosomal protein L14 [Source:MGI Symbol;Acc:MGI:1914365] | Rpl14 | protein_coding |
| ENSMUSG00000025816 | 418.591603 | 0.43526173 | 0.15672201 | 0.00073223 | 0.01097336 | Up | 57743 | Sec61, alpha subunit 2 (S. cerevisiae) [Source:MGI Symbol;Acc:MGI:1931071] | Sec61a2 | protein_coding |
| ENSMUSG00000025825 | 2731.69085 | -0.5198318 | 0.1290369 | 6.56E-06 | 0.00049323 | Down | 66383 | iron-sulfur cluster assembly enzyme [Source:MGI Symbol;Acc:MGI:1913633] | Iscu | protein_coding |
| ENSMUSG00000025854 | 2027.70139 | -0.5330134 | 0.34130266 | 0.00710788 | 0.0479133 | Down | 80752 | FAM20C, golgi associated secretory pathway kinase [Source:MGI Symbol;Acc:MGI:2136853] | Fam20c | protein_coding |
| ENSMUSG00000025856 | 284.809506 | -0.6334226 | 0.19000615 | 6.11E-05 | 0.00211041 | Down | 18590 | platelet derived growth factor, alpha [Source:MGI Symbol;Acc:MGI:97527] | Pdgfa | protein_coding |
| ENSMUSG00000025858 | 884.899339 | -0.4431527 | 0.09223424 | 1.37E-07 | 3.65E-05 | Down | 67604 | golgi to ER traffic protein 4 [Source:MGI Symbol;Acc:MGI:1914854] | Get4 | protein_coding |
| ENSMUSG00000025860 | 2065.08561 | 0.44095952 | 0.12377838 | 6.15E-05 | 0.00212136 | Up | 11798 | X-linked inhibitor of apoptosis [Source:MGI Symbol;Acc:MGI:107572] | Xiap | protein_coding |
| ENSMUSG00000025862 | 2024.45001 | 0.32545307 | 0.12810739 | 0.00256947 | 0.02445285 | Up | 20843 | stromal antigen 2 [Source:MGI Symbol;Acc:MGI:1098583] | Stag2 | protein_coding |

| | | | | | | | | | | |
|---|---|---|---|---|---|---|---|---|---|---|
| ENSMUSG00000025868 | 874.013651 | -1.0191924 | 0.31608331 | 5.94E-05 | 0.00206241 | Down | 67044 | HIG1 domain family, member 2A [Source:MGI Symbol;Acc:MGI:1914294] | Higd2a | protein_coding |
| ENSMUSG00000025869 | 332.903122 | -0.5080856 | 0.20497048 | 0.00125686 | 0.01532864 | Down | 28126 | NOP16 nucleolar protein [Source:MGI Symbol;Acc:MGI:107862] | Nop16 | protein_coding |
| ENSMUSG00000025875 | 1748.97085 | -1.0494411 | 0.2407155 | 6.60E-07 | 0.00010939 | Down | 74257 | tetraspanin 17 [Source:MGI Symbol;Acc:MGI:1921507] | Tspan17 | protein_coding |
| ENSMUSG00000025898 | 420.193323 | 0.32029182 | 0.11352563 | 0.00118385 | 0.01482781 | Up | 244672 | CWF19-like 2, cell cycle control (S. pombe) [Source:MGI Symbol;Acc:MGI:1918023] | Cwf19l2 | protein_coding |
| ENSMUSG00000025907 | 1115.29786 | 0.31778946 | 0.14426538 | 0.00607727 | 0.04288673 | Up | 12421 | RB1-inducible coiled-coil 1 [Source:MGI Symbol;Acc:MGI:1341850] | Rb1cc1 | protein_coding |
| ENSMUSG00000025915 | 435.924093 | 0.37746882 | 0.14242009 | 0.00142582 | 0.01677221 | Up | 170755 | serum/glucocorticoid regulated kinase 3 [Source:MGI Symbol;Acc:MGI:2182368] | Sgk3 | protein_coding |
| ENSMUSG00000025917 | 1125.77298 | -0.4343566 | 0.16298846 | 0.00095495 | 0.01305827 | Down | 26754 | COP9 signalosome subunit 5 [Source:MGI Symbol;Acc:MGI:1349415] | Cops5 | protein_coding |
| ENSMUSG00000025937 | 1441.6371 | 0.46688105 | 0.13922629 | 0.00011055 | 0.00310699 | Up | 212442 | lactamase, beta 2 [Source:MGI Symbol;Acc:MGI:2442551] | Lactb2 | protein_coding |
| ENSMUSG00000025958 | 791.411259 | 0.36217502 | 0.09026294 | 1.30E-05 | 0.00081719 | Up | 12912 | cAMP responsive element binding protein 1 [Source:MGI Symbol;Acc:MGI:88494] | Creb1 | protein_coding |
| ENSMUSG00000025959 | 288.740982 | 0.52572578 | 0.25727757 | 0.00330486 | 0.02881777 | Up | 93691 | Kruppel-like factor 7 (ubiquitous) [Source:MGI Symbol;Acc:MGI:1935151] | Klf7 | protein_coding |
| ENSMUSG00000025967 | 3303.30789 | -0.4052734 | 0.19479322 | 0.00506031 | 0.03804015 | Down | 55949 | eukaryotic translation elongation factor 1 beta 2 [Source:MGI Symbol;Acc:MGI:1929520] | Eef1b2 | protein_coding |
| ENSMUSG00000025986 | 628.029071 | 0.37768946 | 0.14501239 | 0.0015974 | 0.01788111 | Up | 227059 | solute carrier family 39 (zinc transporter), member 10 [Source:MGI Symbol;Acc:MGI:1914515] | Slc39a10 | protein_coding |
| ENSMUSG00000026017 | 197.607915 | 0.52444729 | 0.16879614 | 0.00019824 | 0.00464036 | Up | 241066 | calcium response factor [Source:MGI Symbol;Acc:MGI:2182269] | Carf | protein_coding |
| ENSMUSG00000026032 | 1048.23826 | -0.4922798 | 0.23575984 | 0.00328727 | 0.02876027 | Down | 66495 | NADH:ubiquinone oxidoreductase subunit B3 [Source:MGI Symbol;Acc:MGI:1913745] | Ndufb3 | protein_coding |
| ENSMUSG00000026034 | 2452.9078 | 0.40321908 | 0.1730345 | 0.00302445 | 0.0271599 | Up | 12747 | CDC-like kinase 1 [Source:MGI Symbol;Acc:MGI:107403] | Clk1 | protein_coding |
| ENSMUSG00000026042 | 3403.87079 | -0.4037152 | 0.21345584 | 0.00754404 | 0.04987912 | Down | 12832 | collagen, type V, alpha 2 [Source:MGI Symbol;Acc:MGI:88458] | Col5a2 | protein_coding |
| ENSMUSG00000026078 | 532.631982 | -0.485669 | 0.21902539 | 0.00263931 | 0.0248554 | Down | 68833 | phosducin-like 3 [Source:MGI Symbol;Acc:MGI:1916083] | Pdcl3 | protein_coding |
| ENSMUSG00000026082 | 272.528998 | 0.6917154 | 0.17019567 | 3.77E-06 | 0.00036065 | Up | 56210 | REV1, DNA directed polymerase [Source:MGI Symbol;Acc:MGI:1929074] | Rev1 | protein_coding |
| ENSMUSG00000026100 | 12.4551726 | -3.1989014 | 0.95087966 | 3.16E-05 | 0.00144822 | Down | 17700 | myostatin [Source:MGI Symbol;Acc:MGI:95691] | Mstn | protein_coding |
| ENSMUSG00000026102 | 412.012187 | 0.38532476 | 0.15825213 | 0.00237463 | 0.02321049 | Up | 16329 | inositol polyphosphate-1-phosphatase [Source:MGI Symbol;Acc:MGI:104848] | Inpp1 | protein_coding |
| ENSMUSG00000026111 | 772.50287 | -0.3563006 | 0.13717087 | 0.00179811 | 0.01938311 | Down | 67387 | unc-50 homolog [Source:MGI Symbol;Acc:MGI:1914637] | Unc50 | protein_coding |
| ENSMUSG00000026154 | 270.621942 | -0.7108361 | 0.30062813 | 0.00099477 | 0.01330743 | Down | 68002 | succinate dehydrogenase complex assembly factor 4 [Source:MGI Symbol;Acc:MGI:1915252] | Sdhaf4 | protein_coding |
| ENSMUSG00000026156 | 25.7261067 | 0.80184709 | 0.33527631 | 0.00089006 | 0.01242185 | Up | 280645 | beta-1,3-glucuronyltransferase 2 (glucuronosyltransferase S) [Source:MGI Symbol;Acc:MGI:2389490] | B3gat2 | protein_coding |
| ENSMUSG00000026174 | 415.86166 | 0.23265677 | 0.08746835 | 0.00319531 | 0.02819979 | Up | 58184 | CCR4-NOT transcription complex, subunit 9 [Source:MGI Symbol;Acc:MGI:1928902] | Cnot9 | protein_coding |
| ENSMUSG00000026223 | 2678.76295 | -0.7152944 | 0.14859 | 1.07E-07 | 2.99E-05 | Down | 64294 | integral membrane protein 2C [Source:MGI Symbol;Acc:MGI:1927594] | Itm2c | protein_coding |
| ENSMUSG00000026229 | 2433.76134 | 0.37519429 | 0.10137604 | 4.42E-05 | 0.00173708 | Up | 70247 | proteasome (prosome, macropain) 26S subunit, non-ATPase, 1 [Source:MGI Symbol;Acc:MGI:1917497] | Psmd1 | protein_coding |
| ENSMUSG00000026235 | 64.9739072 | 0.6570029 | 0.27534093 | 0.00111764 | 0.01427358 | Up | 13838 | Eph receptor A4 [Source:MGI Symbol;Acc:MGI:98277] | Epha4 | protein_coding |
| ENSMUSG00000026239 | 219.414652 | -0.3501463 | 0.12905086 | 0.00133997 | 0.0161362 | Down | 18582 | phosphodiesterase 6D, cGMP-specific, rod, delta [Source:MGI Symbol;Acc:MGI:1270843] | Pde6d | protein_coding |
| ENSMUSG00000026278 | 495.629639 | -0.5902174 | 0.31619994 | 0.00371351 | 0.0310638 | Down | 51800 | BCL2-related ovarian killer [Source:MGI Symbol;Acc:MGI:1858494] | Bok | protein_coding |
| ENSMUSG00000026281 | 561.478837 | -0.306373 | 0.10356007 | 0.00082895 | 0.01184377 | Down | 21915 | deoxythymidylate kinase [Source:MGI Symbol;Acc:MGI:108396] | Dtymk | protein_coding |
| ENSMUSG00000026335 | 4163.90192 | -0.6422143 | 0.20522761 | 0.00013497 | 0.00361195 | Down | 18484 | peptidylglycine alpha-amidating monooxygenase [Source:MGI Symbol;Acc:MGI:97475] | Pam | protein_coding |
| ENSMUSG00000026349 | 981.972316 | 0.55165923 | 0.11374941 | 1.44E-07 | 3.78E-05 | Up | 72949 | cyclin T2 [Source:MGI Symbol;Acc:MGI:1920199] | Ccnt2 | protein_coding |
| ENSMUSG00000026384 | 912.095903 | 0.45985172 | 0.11760791 | 1.32E-05 | 0.0008203 | Up | 19258 | protein tyrosine phosphatase, non-receptor type 4 [Source:MGI Symbol;Acc:MGI:1099792] | Ptpn4 | protein_coding |
| ENSMUSG00000026398 | 34.5847752 | 0.68394735 | 0.37046225 | 0.00325867 | 0.0285683 | Up | 26424 | nuclear receptor subfamily 5, group A, member 2 [Source:MGI Symbol;Acc:MGI:1346834] | Nr5a2 | protein_coding |
| ENSMUSG00000026399 | 1325.37758 | -0.3699456 | 0.1562974 | 0.00301468 | 0.02708706 | Down | 13136 | CD55 molecule, decay accelerating factor for complement [Source:MGI Symbol;Acc:MGI:104850] | Cd55 | protein_coding |
| ENSMUSG00000026409 | 219.665157 | 0.40813301 | 0.18745755 | 0.0039292 | 0.03225355 | Up | 18640 | 6-phosphofructo-2-kinase/fructose-2,6-biphosphatase 2 [Source:MGI Symbol;Acc:MGI:107815] | Pfkfb2 | protein_coding |
| ENSMUSG00000026411 | 431.021036 | -0.3627553 | 0.12076484 | 0.00058408 | 0.00934817 | Down | 66241 | transmembrane protein 9 [Source:MGI Symbol;Acc:MGI:1913491] | Tmem9 | protein_coding |
| ENSMUSG00000026413 | 25.2783142 | -0.1230254 | 0.25533012 | 0.00639576 | 0.0444964 | Down | 18772 | plakophilin 1 [Source:MGI Symbol;Acc:MGI:1328359] | Pkp1 | protein_coding |
| ENSMUSG00000026421 | 2846.61692 | -0.8768549 | 0.09669866 | 8.36E-21 | 1.36E-16 | Down | 13007 | cysteine and glycine-rich protein 1 [Source:MGI Symbol;Acc:MGI:88549] | Csrp1 | protein_coding |
| ENSMUSG00000026433 | 433.289034 | -0.8769 | 0.22073538 | 4.34E-06 | 0.00037622 | Down | 226422 | RAB29, member RAS oncogene family [Source:MGI Symbol;Acc:MGI:2385107] | Rab29 | protein_coding |
| ENSMUSG00000026434 | 1712.53513 | 0.39102856 | 0.08101032 | 2.73E-07 | 5.99E-05 | Up | 98415 | nuclear casein kinase and cyclin-dependent kinase substrate 1 [Source:MGI Symbol;Acc:MGI:1934811] | Nucks1 | protein_coding |
| ENSMUSG00000026443 | 182.344305 | -0.4339103 | 0.21207615 | 0.00463564 | 0.0359007 | Down | 16980 | leucine rich repeat protein 2, neuronal [Source:MGI Symbol;Acc:MGI:106037] | Lrrn2 | protein_coding |
| ENSMUSG00000026456 | 821.844544 | -0.3356423 | 0.1455351 | 0.00437229 | 0.03446687 | Down | 72017 | cytochrome b5 reductase 1 [Source:MGI Symbol;Acc:MGI:1919267] | Cyb5r1 | protein_coding |
| ENSMUSG00000026466 | 1877.56661 | 0.25214957 | 0.10343321 | 0.00530761 | 0.03935283 | Up | 208263 | torsin A interacting protein 1 [Source:MGI Symbol;Acc:MGI:3582693] | Tor1aip1 | protein_coding |
| ENSMUSG00000026471 | 910.707736 | 0.30294169 | 0.13136013 | 0.0049765 | 0.03772865 | Up | 15064 | major histocompatibility complex, class I-related [Source:MGI Symbol;Acc:MGI:1195463] | Mr1 | protein_coding |
| ENSMUSG00000026491 | 1017.63253 | 0.37256785 | 0.13402622 | 0.00100394 | 0.01334051 | Up | 226747 | AT hook containing transcription factor 1 [Source:MGI Symbol;Acc:MGI:1915033] | Ahctf1 | protein_coding |
| ENSMUSG00000026516 | 693.011166 | 0.26480585 | 0.09076678 | 0.0012808 | 0.01556217 | Up | 67459 | nuclear VCP-like [Source:MGI Symbol;Acc:MGI:1914709] | Nvl | protein_coding |
| ENSMUSG00000026520 | 506.463616 | -0.5505242 | 0.15339318 | 3.23E-05 | 0.00145985 | Down | 69051 | pyrroline-5-carboxylate reductase family, member 2 [Source:MGI Symbol;Acc:MGI:1277956] | Pycr2 | protein_coding |
| ENSMUSG00000026567 | 43.3817057 | 1.31650197 | 0.50735679 | 0.00035084 | 0.00676081 | Up | 271639 | adenylate cyclase 10 [Source:MGI Symbol;Acc:MGI:2660854] | Adcy10 | protein_coding |
| ENSMUSG00000026571 | 1094.81998 | 0.3757735 | 0.08823114 | 4.12E-06 | 0.00036814 | Up | 74106 | DDB1 and CUL4 associated factor 6 [Source:MGI Symbol;Acc:MGI:1921356] | Dcaf6 | protein_coding |
| ENSMUSG00000026574 | 6625.68504 | -0.4115651 | 0.141816 | 0.00051204 | 0.00854899 | Down | 56429 | dermatopontin [Source:MGI Symbol;Acc:MGI:1928392] | Dpt | protein_coding |
| ENSMUSG00000026622 | 57.0634697 | -0.8670444 | 0.3816778 | 0.00101195 | 0.01339787 | Down | 18005 | NIMA (never in mitosis gene a)-related expressed kinase 2 [Source:MGI Symbol;Acc:MGI:109359] | Nek2 | protein_coding |
| ENSMUSG00000026630 | 99.4406417 | -0.9383534 | 0.40810362 | 0.00088098 | 0.01238019 | Down | 381319 | basic leucine zipper transcription factor, ATF-like 3 [Source:MGI Symbol;Acc:MGI:1925491] | Batf3 | protein_coding |
| ENSMUSG00000026639 | 142.730934 | -0.9329047 | 0.31562568 | 0.00015267 | 0.00393057 | Down | 16780 | laminin, beta 3 [Source:MGI Symbol;Acc:MGI:99915] | Lamb3 | protein_coding |
| ENSMUSG00000026641 | 483.942092 | -0.3457993 | 0.14929483 | 0.00381135 | 0.03154118 | Down | 22278 | upstream transcription factor 1 [Source:MGI Symbol;Acc:MGI:99542] | Usf1 | protein_coding |

| | | | | | | | | | | |
|---|---|---|---|---|---|---|---|---|---|---|
| ENSMUSG00000026692 | 23.9708489 | 0.94283711 | 0.46566503 | 0.00164876 | 0.01829511 | Up | 226564 | flavin containing monooxygenase 4 [Source:MGI Symbol;Acc:MGI:2429497] | Fmo4 | protein_coding |
| ENSMUSG00000026728 | 36679.5171 | -0.7939977 | 0.14953884 | 7.38E-09 | 5.07E-06 | Down | 22352 | vimentin [Source:MGI Symbol;Acc:MGI:98932] | Vim | protein_coding |
| ENSMUSG00000026739 | 1030.65605 | 0.31423689 | 0.11449429 | 0.00158196 | 0.01778474 | Up | 12151 | Bmi1 polycomb ring finger oncogene [Source:MGI Symbol;Acc:MGI:88174] | Bmi1 | protein_coding |
| ENSMUSG00000026743 | 595.173912 | 0.50700595 | 0.15846363 | 0.00015617 | 0.00400779 | Up | 17354 | myeloid/lymphoid or mixed-lineage leukemia; translocated to, 10 [Source:MGI Symbol;Acc:MGI:1329038] | Mllt10 | protein_coding |
| ENSMUSG00000026749 | 1235.36081 | -0.4238283 | 0.19049041 | 0.00321745 | 0.02832928 | Down | 59126 | NIMA (never in mitosis gene a)-related expressed kinase 6 [Source:MGI Symbol;Acc:MGI:1891638] | Nek6 | protein_coding |
| ENSMUSG00000026754 | 503.527392 | 0.33406559 | 0.10456084 | 0.00033247 | 0.00655467 | Up | 76899 | golgi autoantigen, golgin subfamily a, 1 [Source:MGI Symbol;Acc:MGI:1924149] | Golga1 | protein_coding |
| ENSMUSG00000026764 | 59.3869634 | 0.6455945 | 0.24420803 | 0.00058871 | 0.00939442 | Up | 16574 | kinesin family member 5C [Source:MGI Symbol;Acc:MGI:1098269] | Kif5c | protein_coding |
| ENSMUSG00000026770 | 101.880418 | 0.56518614 | 0.26649823 | 0.00249814 | 0.02401966 | Up | 16184 | interleukin 2 receptor, alpha chain [Source:MGI Symbol;Acc:MGI:96549] | Il2ra | protein_coding |
| ENSMUSG00000026811 | 516.517953 | -0.4277174 | 0.15470324 | 0.0008019 | 0.01161263 | Down | 50935 | ST6 (alpha-N-acetyl-neuraminyl-2,3-beta-galactosyl-1,3)-N-acetylgalactosaminide alpha-2,6-sialyltransferase 6 [Source:MGI Symbol;Acc:MGI:1355316] | St6galnac6 | protein_coding |
| ENSMUSG00000026817 | 269.222361 | -0.6433897 | 0.24489014 | 0.00059581 | 0.00946125 | Down | 11636 | adenylate kinase 1 [Source:MGI Symbol;Acc:MGI:87977] | Ak1 | protein_coding |
| ENSMUSG00000026827 | 1768.49094 | 0.82884136 | 0.32112306 | 0.00049219 | 0.00829418 | Up | 14571 | glycerol phosphate dehydrogenase 2, mitochondrial [Source:MGI Symbol;Acc:MGI:99778] | Gpd2 | protein_coding |
| ENSMUSG00000026837 | 5785.91413 | -0.5578278 | 0.28141603 | 0.00333629 | 0.02895618 | Down | 12831 | collagen, type V, alpha 1 [Source:MGI Symbol;Acc:MGI:88457] | Col5a1 | protein_coding |
| ENSMUSG00000026839 | 158.182695 | -0.4580563 | 0.18408499 | 0.00146101 | 0.01698185 | Down | 76654 | uridine phosphorylase 2 [Source:MGI Symbol;Acc:MGI:1923904] | Upp2 | protein_coding |
| ENSMUSG00000026851 | 576.817612 | -0.5283347 | 0.19958944 | 0.00079408 | 0.01155904 | Down | 227707 | cDNA sequence BC005624 [Source:MGI Symbol;Acc:MGI:2385132] | BC005624 | protein_coding |
| ENSMUSG00000026857 | 278.636985 | -0.416312 | 0.13270308 | 0.00021357 | 0.00486595 | Down | 66617 | N-terminal Xaa-Pro-Lys N-methyltransferase 1 [Source:MGI Symbol;Acc:MGI:1913867] | Ntmt1 | protein_coding |
| ENSMUSG00000026878 | 2950.478 | 0.19333403 | 0.06447294 | 0.0015842 | 0.01779759 | Up | 68365 | RAB14, member RAS oncogene family [Source:MGI Symbol;Acc:MGI:1915615] | Rab14 | protein_coding |
| ENSMUSG00000026895 | 1184.69827 | -0.4212949 | 0.19548377 | 0.00394778 | 0.03232446 | Down | 68375 | NADH:ubiquinone oxidoreductase subunit A8 [Source:MGI Symbol;Acc:MGI:1915625] | Ndufa8 | protein_coding |
| ENSMUSG00000026928 | 77.8657338 | -0.5941806 | 0.27404749 | 0.00207401 | 0.02122768 | Down | 332579 | caspase recruitment domain family, member 9 [Source:MGI Symbol;Acc:MGI:2685628] | Card9 | protein_coding |
| ENSMUSG00000026939 | 181.641088 | -0.4602321 | 0.18980659 | 0.00174479 | 0.01894659 | Down | 51875 | transmembrane protein 141 [Source:MGI Symbol;Acc:MGI:1098773] | Tmem141 | protein_coding |
| ENSMUSG00000026965 | 1914.10603 | -0.2239611 | 0.0625428 | 0.00014436 | 0.00375216 | Down | 99152 | anaphase promoting complex subunit 2 [Source:MGI Symbol;Acc:MGI:2139135] | Anapc2 | protein_coding |
| ENSMUSG00000026966 | 428.452068 | -0.4860301 | 0.16625555 | 0.00038295 | 0.00711788 | Down | 68475 | SS nuclear autoantigen 1 [Source:MGI Symbol;Acc:MGI:1915725] | Ssna1 | protein_coding |
| ENSMUSG00000026974 | 234.983421 | -0.4122709 | 0.14242467 | 0.0005904 | 0.00941222 | Down | 67187 | zinc finger, MYND domain containing 19 [Source:MGI Symbol;Acc:MGI:1914437] | Zmynd19 | protein_coding |
| ENSMUSG00000026977 | 1213.32676 | 0.32954334 | 0.11424967 | 0.00093989 | 0.01292775 | Up | 57438 | membrane associated ring-CH-type finger 7 [Source:MGI Symbol;Acc:MGI:1931053] | Marchf7 | protein_coding |
| ENSMUSG00000027001 | 189.722148 | -0.2847337 | 0.12412958 | 0.0060031 | 0.04254817 | Down | 68082 | dual specificity phosphatase 19 [Source:MGI Symbol;Acc:MGI:1915332] | Dusp19 | protein_coding |
| ENSMUSG00000027086 | 264.406035 | 0.45342621 | 0.14209078 | 0.00017004 | 0.00421008 | Up | 320720 | FAST kinase domains 1 [Source:MGI Symbol;Acc:MGI:2444596] | Fastkd1 | protein_coding |
| ENSMUSG00000027109 | 1732.41068 | 0.27669738 | 0.09706288 | 0.00134319 | 0.01614199 | Up | 20687 | trans-acting transcription factor 3 [Source:MGI Symbol;Acc:MGI:1277166] | Sp3 | protein_coding |
| ENSMUSG00000027122 | 349.234772 | 0.30260343 | 0.08930867 | 0.0002018 | 0.00469853 | Up | 212772 | ADP-ribosylation factor-like 14 effector protein [Source:MGI Symbol;Acc:MGI:1926020] | Arl14ep | protein_coding |
| ENSMUSG00000027131 | 783.793938 | -0.4190377 | 0.12913852 | 0.00017053 | 0.00421008 | Down | 68032 | ER membrane protein complex subunit 4 [Source:MGI Symbol;Acc:MGI:1915282] | Emc4 | protein_coding |
| ENSMUSG00000027133 | 516.471276 | -0.7712853 | 0.2129153 | 2.25E-05 | 0.00115443 | Down | 66181 | NOP10 ribonucleoprotein [Source:MGI Symbol;Acc:MGI:1913431] | Nop10 | protein_coding |
| ENSMUSG00000027163 | 431.66118 | -0.6538476 | 0.17175645 | 1.14E-05 | 0.0007528 | Down | 76501 | COMM domain containing 9 [Source:MGI Symbol;Acc:MGI:1923751] | Commd9 | protein_coding |
| ENSMUSG00000027164 | 530.65132 | 0.34696239 | 0.14342254 | 0.00308694 | 0.0275838 | Up | 22034 | TNF receptor-associated factor 6 [Source:MGI Symbol;Acc:MGI:108072] | Traf6 | protein_coding |
| ENSMUSG00000027165 | 463.4152 | -0.3967798 | 0.17877469 | 0.00338477 | 0.02923208 | Down | 68170 | intraflagellar transport associated protein [Source:MGI Symbol;Acc:MGI:1915420] | Iftap | protein_coding |
| ENSMUSG00000027166 | 227.871914 | -0.4733366 | 0.16068271 | 0.00037056 | 0.00697533 | Down | 99349 | DnaJ heat shock protein family (Hsp40) member C24 [Source:MGI Symbol;Acc:MGI:1919522] | Dnajc24 | protein_coding |
| ENSMUSG00000027180 | 1586.64599 | 0.25665196 | 0.08743707 | 0.00117111 | 0.01471355 | Up | 57443 | F-box protein 3 [Source:MGI Symbol;Acc:MGI:1929084] | Fbxo3 | protein_coding |
| ENSMUSG00000027189 | 2967.12951 | 0.32087479 | 0.13548949 | 0.0041289 | 0.03325433 | Up | 80985 | tripartite motif-containing 44 [Source:MGI Symbol;Acc:MGI:1931835] | Trim44 | protein_coding |
| ENSMUSG00000027203 | 193.409378 | -0.4161603 | 0.17241631 | 0.00227052 | 0.02254208 | Down | 110074 | deoxyuridine triphosphatase [Source:MGI Symbol;Acc:MGI:1346051] | Dut | protein_coding |
| ENSMUSG00000027224 | 99.4910553 | -1.374057 | 0.51469397 | 0.00027124 | 0.00570043 | Down | 213696 | dual oxidase maturation factor 1 [Source:MGI Symbol;Acc:MGI:2384861] | Duoxa1 | protein_coding |
| ENSMUSG00000027230 | 594.015323 | -0.6099498 | 0.26157316 | 0.00136366 | 0.01630477 | Down | 26427 | cAMP responsive element binding protein 3-like 1 [Source:MGI Symbol;Acc:MGI:1347062] | Creb3l1 | protein_coding |
| ENSMUSG00000027245 | 32.126809 | 0.61349102 | 0.35651234 | 0.00468532 | 0.03610755 | Up | 67693 | huntingtin interacting protein K [Source:MGI Symbol;Acc:MGI:1914943] | Hypk | protein_coding |
| ENSMUSG00000027254 | 930.853857 | -0.9455841 | 0.51428252 | 0.00217025 | 0.02191828 | Down | 17754 | microtubule-associated protein 1 A [Source:MGI Symbol;Acc:MGI:1306776] | Map1a | protein_coding |
| ENSMUSG00000027276 | 1107.58142 | 0.2936059 | 0.13500527 | 0.00755598 | 0.04988808 | Up | 16449 | jagged 1 [Source:MGI Symbol;Acc:MGI:1095416] | Jag1 | protein_coding |
| ENSMUSG00000027284 | 288.940855 | 0.27809131 | 0.1195843 | 0.0059134 | 0.04207764 | Up | 68968 | congenital dyserythropoietic anemia, type I (human) [Source:MGI Symbol;Acc:MGI:1916218] | Cdan1 | protein_coding |
| ENSMUSG00000027312 | 1660.13358 | 0.32902691 | 0.13585311 | 0.00349473 | 0.02984854 | Up | 11990 | attractin [Source:MGI Symbol;Acc:MGI:1341628] | Atrn | protein_coding |
| ENSMUSG00000027335 | 180.186303 | -0.8490707 | 0.28019735 | 0.00013518 | 0.00361195 | Down | 11550 | adrenergic receptor, alpha 1d [Source:MGI Symbol;Acc:MGI:106673] | Adra1d | protein_coding |
| ENSMUSG00000027341 | 517.001871 | -0.2392089 | 0.10061395 | 0.00696405 | 0.04730581 | Down | 70612 | transmembrane protein 230 [Source:MGI Symbol;Acc:MGI:1917862] | Tmem230 | protein_coding |
| ENSMUSG00000027342 | 877.564352 | -0.3484566 | 0.13745053 | 0.00221812 | 0.02221538 | Down | 18538 | proliferating cell nuclear antigen [Source:MGI Symbol;Acc:MGI:97503] | Pcna | protein_coding |
| ENSMUSG00000027346 | 968.282549 | 0.41972472 | 0.11609 | 4.89E-05 | 0.00186708 | Up | 74182 | glycerophosphocholine phosphodiesterase 1 [Source:MGI Symbol;Acc:MGI:104898] | Gpcpd1 | protein_coding |
| ENSMUSG00000027356 | 48.6986915 | 0.03714389 | 0.20327203 | 0.00537567 | 0.03959432 | Up | 241639 | fermitin family member 1 [Source:MGI Symbol;Acc:MGI:2443583] | Fermt1 | protein_coding |
| ENSMUSG00000027365 | 1832.09437 | 0.45662826 | 0.14005054 | 0.00016071 | 0.00407555 | Up | 58800 | transient receptor potential cation channel, subfamily M, member 7 [Source:MGI Symbol;Acc:MGI:1929996] | Trpm7 | protein_coding |
| ENSMUSG00000027380 | 13.5938705 | -0.1471025 | 0.28296216 | 0.00563034 | 0.04076902 | Down | 74121 | acyl-Coenzyme A oxidase-like [Source:MGI Symbol;Acc:MGI:1921371] | Acoxl | protein_coding |
| ENSMUSG00000027381 | 229.599937 | 0.39514641 | 0.18713361 | 0.00491416 | 0.03735638 | Up | 12125 | BCL2-like 11 (apoptosis facilitator) [Source:MGI Symbol;Acc:MGI:1197519] | Bcl2l11 | protein_coding |

| | | | | | | | | | | |
|---|---|---|---|---|---|---|---|---|---|---|
| ENSMUSG00000027404 | 1304.67188 | -0.4528649 | 0.14786698 | 0.00026995 | 0.00569518 | Down | 20638 | small nuclear ribonucleoprotein B [Source:MGI Symbol;Acc:MGI:98342] | Snrpb | protein_coding |
| ENSMUSG00000027411 | 634.631268 | -0.209795 | 0.08151355 | 0.0047748 | 0.03665717 | Down | 80743 | VSP16 CORVET/HOPS core subunit [Source:MGI Symbol;Acc:MGI:2136772] | Vps16 | protein_coding |
| ENSMUSG00000027422 | 5934.21667 | -0.4096585 | 0.16101744 | 0.00146405 | 0.01698816 | Down | 81910 | ribosome binding protein 1 [Source:MGI Symbol;Acc:MGI:1932395] | Rrbp1 | protein_coding |
| ENSMUSG00000027447 | 15309.6699 | -0.7664381 | 0.16405027 | 3.02E-07 | 6.46E-05 | Down | 13010 | cystatin C [Source:MGI Symbol;Acc:MGI:102519] | Cst3 | protein_coding |
| ENSMUSG00000027455 | 1155.30476 | -0.4706481 | 0.19395335 | 0.00201019 | 0.02088341 | Down | 386649 | NSFL1 (p97) cofactor (p47) [Source:MGI Symbol;Acc:MGI:3042273] | Nsfl1c | protein_coding |
| ENSMUSG00000027459 | 94.1155804 | -0.8914189 | 0.25264032 | 2.48E-05 | 0.00122687 | Down | 73847 | family with sequence similarity 110, member A [Source:MGI Symbol;Acc:MGI:1921097] | Fam110a | protein_coding |
| ENSMUSG00000027472 | 1175.77096 | -0.6944494 | 0.18066771 | 1.02E-05 | 0.00070901 | Down | 68559 | p53 and DNA damage regulated 1 [Source:MGI Symbol;Acc:MGI:1915809] | Pdrg1 | protein_coding |
| ENSMUSG00000027490 | 233.053507 | -0.8431644 | 0.22542123 | 1.11E-05 | 0.0007425 | Down | 13555 | E2F transcription factor 1 [Source:MGI Symbol;Acc:MGI:101941] | E2f1 | protein_coding |
| ENSMUSG00000027499 | 650.95574 | -0.357551 | 0.15603525 | 0.00390281 | 0.03210187 | Down | 18767 | protein kinase inhibitor, alpha [Source:MGI Symbol;Acc:MGI:104747] | Pkia | protein_coding |
| ENSMUSG00000027510 | 220.829688 | 0.47313996 | 0.2233739 | 0.00339398 | 0.02924946 | Up | 56190 | RNA binding motif protein 38 [Source:MGI Symbol;Acc:MGI:1889294] | Rbm38 | protein_coding |
| ENSMUSG00000027546 | 5635.42034 | -0.5064319 | 0.14202624 | 4.02E-05 | 0.00164117 | Down | 11981 | ATPase, class II, type 9A [Source:MGI Symbol;Acc:MGI:1330826] | Atp9a | protein_coding |
| ENSMUSG00000027583 | 361.116261 | 0.53448734 | 0.16686815 | 0.00014141 | 0.00369317 | Up | 72147 | zinc finger and BTB domain containing 46 [Source:MGI Symbol;Acc:MGI:1919397] | Zbtb46 | protein_coding |
| ENSMUSG00000027589 | 1056.1669 | 0.27710352 | 0.11873762 | 0.00619329 | 0.04351315 | Up | 245867 | protein-L-isoaspartate (D-aspartate) O-methyltransferase domain containing 2 [Source:MGI Symbol;Acc:MGI:1923927] | Pcmtd2 | protein_coding |
| ENSMUSG00000027593 | 1661.10187 | -0.2588754 | 0.11283687 | 0.00689412 | 0.04695804 | Down | 19383 | hnRNP-associated with lethal yellow [Source:MGI Symbol;Acc:MGI:97850] | Raly | protein_coding |
| ENSMUSG00000027602 | 2995.55512 | -0.9277556 | 0.29371166 | 7.97E-05 | 0.00251969 | Down | 66734 | microtubule-associated protein 1 light chain 3 alpha [Source:MGI Symbol;Acc:MGI:1915661] | Map1lc3a | protein_coding |
| ENSMUSG00000027605 | 5592.1322 | 0.71369468 | 0.33469179 | 0.00152454 | 0.01741052 | Up | 60525 | acyl-CoA synthetase short-chain family member 2 [Source:MGI Symbol;Acc:MGI:1890410] | Acss2 | protein_coding |
| ENSMUSG00000027613 | 768.267645 | -0.4095604 | 0.15961916 | 0.00152221 | 0.01741052 | Down | 16418 | eukaryotic translation initiation factor 6 [Source:MGI Symbol;Acc:MGI:1196288] | Eif6 | protein_coding |
| ENSMUSG00000027630 | 767.78831 | 0.52828072 | 0.11436769 | 4.86E-07 | 8.78E-05 | Up | 81004 | transducin (beta)-like 1X-linked receptor 1 [Source:MGI Symbol;Acc:MGI:2441730] | Tbl1xr1 | protein_coding |
| ENSMUSG00000027641 | 309.649829 | 0.27288418 | 0.11662814 | 0.00585303 | 0.04177802 | Up | 19650 | RB transcriptional corepressor like 1 [Source:MGI Symbol;Acc:MGI:103300] | Rbl1 | protein_coding |
| ENSMUSG00000027642 | 5692.81688 | -0.2226024 | 0.08630517 | 0.0042905 | 0.0341161 | Down | 20014 | ribophorin II [Source:MGI Symbol;Acc:MGI:98085] | Rpn2 | protein_coding |
| ENSMUSG00000027649 | 417.12332 | -0.3127984 | 0.11592259 | 0.00170746 | 0.01871642 | Down | 66642 | catenin, beta like 1 [Source:MGI Symbol;Acc:MGI:1913892] | Ctnnbl1 | protein_coding |
| ENSMUSG00000027712 | 10641.2925 | -0.745324 | 0.15560145 | 1.32E-07 | 3.59E-05 | Down | 11747 | annexin A5 [Source:MGI Symbol;Acc:MGI:106008] | Anxa5 | protein_coding |
| ENSMUSG00000027763 | 5184.44999 | 0.32481488 | 0.09997288 | 0.00026278 | 0.00560946 | Up | 56758 | muscleblind like splicing factor 1 [Source:MGI Symbol;Acc:MGI:1928482] | Mbnl1 | protein_coding |
| ENSMUSG00000027770 | 881.796239 | 0.39105213 | 0.07649769 | 6.30E-08 | 2.00E-05 | Up | 72162 | DEAH (Asp-Glu-Ala-His) box polypeptide 36 [Source:MGI Symbol;Acc:MGI:1919412] | Dhx36 | protein_coding |
| ENSMUSG00000027792 | 4440.33489 | 0.51039508 | 0.1500748 | 8.47E-05 | 0.00262438 | Up | 12038 | butyrylcholinesterase [Source:MGI Symbol;Acc:MGI:894278] | Bche | protein_coding |
| ENSMUSG00000027796 | 44.6584331 | 0.59647481 | 0.36127033 | 0.00535481 | 0.03955841 | Up | 55994 | SMAD family member 9 [Source:MGI Symbol;Acc:MGI:1859993] | Smad9 | protein_coding |
| ENSMUSG00000027811 | 57.2562051 | -0.5491527 | 0.21610939 | 0.00094743 | 0.01299921 | Down | 75939 | RIKEN cDNA 4930579G24 gene [Source:MGI Symbol;Acc:MGI:1923189] | 4930579G24Rik | protein_coding |
| ENSMUSG00000027829 | 1278.49538 | 0.46146515 | 0.1688514 | 0.00077874 | 0.01139694 | Up | 56706 | cyclin L1 [Source:MGI Symbol;Acc:MGI:1922664] | Ccnl1 | protein_coding |
| ENSMUSG00000027848 | 439.59309 | -0.7001876 | 0.43253026 | 0.00429457 | 0.03413175 | Down | 99543 | olfactomedin-like 3 [Source:MGI Symbol;Acc:MGI:1914877] | Olfml3 | protein_coding |
| ENSMUSG00000027879 | 1245.58703 | -0.4177615 | 0.13795988 | 0.0003576 | 0.00685057 | Down | 20333 | SEC22 homolog B, vesicle trafficking protein [Source:MGI Symbol;Acc:MGI:1338759] | Sec22b | protein_coding |
| ENSMUSG00000027881 | 1862.65476 | 0.33576002 | 0.1197188 | 0.00108448 | 0.01401546 | Up | 66921 | PRP38 pre-mRNA processing factor 38 (yeast) domain containing B [Source:MGI Symbol;Acc:MGI:1914171] | Prpf38b | protein_coding |
| ENSMUSG00000027890 | 706.482436 | -0.5464446 | 0.13148824 | 3.34E-06 | 0.00033259 | Down | 14865 | glutathione S-transferase, mu 4 [Source:MGI Symbol;Acc:MGI:95862] | Gstm4 | protein_coding |
| ENSMUSG00000027907 | 7375.9785 | -0.649724 | 0.14950424 | 1.20E-06 | 0.00016433 | Down | 20195 | S100 calcium binding protein A11 [Source:MGI Symbol;Acc:MGI:1338798] | S100a11 | protein_coding |
| ENSMUSG00000027932 | 154.321217 | -0.4463391 | 0.16931925 | 0.00108772 | 0.01403495 | Down | 26568 | solute carrier family 27 (fatty acid transporter), member 3 [Source:MGI Symbol;Acc:MGI:1347358] | Slc27a3 | protein_coding |
| ENSMUSG00000027935 | 309.395477 | -0.5235509 | 0.18421219 | 0.00044536 | 0.00774495 | Down | 68328 | RAB13, member RAS oncogene family [Source:MGI Symbol;Acc:MGI:1927232] | Rab13 | protein_coding |
| ENSMUSG00000027937 | 985.571793 | -0.3641373 | 0.18124365 | 0.00654676 | 0.04515136 | Down | 23922 | jumping translocation breakpoint [Source:MGI Symbol;Acc:MGI:1346082] | Jtb | protein_coding |
| ENSMUSG00000027957 | 802.903399 | 0.34872262 | 0.15388711 | 0.00451266 | 0.03515976 | Up | 229782 | solute carrier family 35 (UDP-N-acetylglucosamine (UDP-GlcNAc) transporter), member 3 [Source:MGI Symbol;Acc:MGI:1917648] | Slc35a3 | protein_coding |
| ENSMUSG00000027994 | 203.974179 | -0.5313929 | 0.26995166 | 0.00377465 | 0.03136535 | Down | 66815 | mitochondrial calcium uniporter dominant negative beta subunit [Source:MGI Symbol;Acc:MGI:1914065] | Mcub | protein_coding |
| ENSMUSG00000027996 | 631.429566 | -0.7392347 | 0.37363014 | 0.00214938 | 0.02184818 | Down | 20319 | secreted frizzled-related protein 2 [Source:MGI Symbol;Acc:MGI:108078] | Sfrp2 | protein_coding |
| ENSMUSG00000027999 | 1624.88172 | -0.35436 | 0.14133154 | 0.00227463 | 0.02254506 | Down | 66350 | phospholipase A2, group XIIA [Source:MGI Symbol;Acc:MGI:1913600] | Pla2g12a | protein_coding |
| ENSMUSG00000028019 | 672.267827 | -0.2701376 | 0.10463993 | 0.00322128 | 0.02834759 | Down | 54635 | platelet-derived growth factor, C polypeptide [Source:MGI Symbol;Acc:MGI:1859631] | Pdgfc | protein_coding |
| ENSMUSG00000028041 | 4096.48108 | -0.4302779 | 0.09820388 | 2.36E-06 | 0.00025754 | Down | 11490 | a disintegrin and metallopeptidase domain 15 (metargidin) [Source:MGI Symbol;Acc:MGI:1333882] | Adam15 | protein_coding |
| ENSMUSG00000028048 | 909.74282 | -0.2934131 | 0.10958753 | 0.0021083 | 0.02149991 | Down | 14466 | glucosidase, beta, acid [Source:MGI Symbol;Acc:MGI:95665] | Gba | protein_coding |
| ENSMUSG00000028049 | 775.62167 | -0.317327 | 0.13224228 | 0.00381842 | 0.03156757 | Down | 24045 | secretory carrier membrane protein 3 [Source:MGI Symbol;Acc:MGI:1346346] | Scamp3 | protein_coding |
| ENSMUSG00000028060 | 1104.67884 | 0.38070142 | 0.10360171 | 5.18E-05 | 0.00191161 | Up | 74200 | KH domain containing 4, pre-mRNA splicing factor [Source:MGI Symbol;Acc:MGI:1921450] | Khdc4 | protein_coding |
| ENSMUSG00000028062 | 836.840352 | -0.8504658 | 0.2786488 | 0.00012865 | 0.00346504 | Down | 83409 | late endosomal/lysosomal adaptor, MAPK and MTOR activator 2 [Source:MGI Symbol;Acc:MGI:1932697] | Lamtor2 | protein_coding |
| ENSMUSG00000028063 | 2948.20377 | -0.5815075 | 0.13231847 | 1.10E-06 | 0.00015687 | Down | 16905 | lamin A [Source:MGI Symbol;Acc:MGI:96794] | Lmna | protein_coding |
| ENSMUSG00000028070 | 717.205209 | -0.4881203 | 0.15709554 | 0.00020576 | 0.00475336 | Down | 246703 | NAD(P)HX epimerase [Source:MGI Symbol;Acc:MGI:2180167] | Naxe | protein_coding |
| ENSMUSG00000028076 | 7170.00679 | -0.469809 | 0.23580898 | 0.00439037 | 0.03452155 | Down | 12479 | CD1d1 antigen [Source:MGI Symbol;Acc:MGI:107674] | Cd1d1 | protein_coding |
| ENSMUSG00000028098 | 607.890776 | 0.30756085 | 0.09777961 | 0.00044589 | 0.00774495 | Up | 67845 | ring finger protein 115 [Source:MGI Symbol;Acc:MGI:1915095] | Rnf115 | protein_coding |
| ENSMUSG00000028106 | 702.24651 | 0.38058235 | 0.1084549 | 8.87E-05 | 0.00268763 | Up | 75137 | regulation of nuclear pre-mRNA domain containing 2 [Source:MGI Symbol;Acc:MGI:1922387] | Rprd2 | protein_coding |
| ENSMUSG00000028127 | 2233.84918 | 0.21995686 | 0.07230082 | 0.00110436 | 0.01418212 | Up | 19299 | ATP-binding cassette, sub-family D (ALD), member 3 [Source:MGI Symbol;Acc:MGI:1349216] | Abcd3 | protein_coding |
| ENSMUSG00000028133 | 44.1897014 | 1.06285044 | 0.66086833 | 0.002903 | 0.02651452 | Up | 66568 | RWD domain containing 3 [Source:MGI Symbol;Acc:MGI:1920420] | Rwdd3 | protein_coding |

| | | | | | | | | | | |
|---|---|---|---|---|---|---|---|---|---|---|
| ENSMUSG00000028136 | 1084.91251 | 0.30715128 | 0.08363783 | 6.77E-05 | 0.00225926 | Up | 76742 | sorting nexin family member 27 [Source:MGI Symbol;Acc:MGI:1923992] | Snx27 | protein_coding |
| ENSMUSG00000028150 | 220.181511 | 0.79853297 | 0.3441488 | 0.00100393 | 0.01334051 | Up | 19885 | RAR-related orphan receptor gamma [Source:MGI Symbol;Acc:MGI:104856] | Rorc | protein_coding |
| ENSMUSG00000028179 | 201.263628 | -0.5380633 | 0.21757695 | 0.00117265 | 0.01472154 | Down | 107869 | cystathionase (cystathionine gamma-lyase) [Source:MGI Symbol;Acc:MGI:1339968] | Cth | protein_coding |
| ENSMUSG00000028233 | 488.705717 | 0.35394885 | 0.12922586 | 0.00121255 | 0.01504799 | Up | 116940 | trimethylguanosine synthase 1 [Source:MGI Symbol;Acc:MGI:2151797] | Tgs1 | protein_coding |
| ENSMUSG00000028234 | 5191.39598 | -0.671384 | 0.19619204 | 4.71E-05 | 0.00183193 | Down | 67427 | ribosomal protein S20 [Source:MGI Symbol;Acc:MGI:1914677] | Rps20 | protein_coding |
| ENSMUSG00000028251 | 163.296468 | -0.4819821 | 0.22571966 | 0.00311359 | 0.02779139 | Down | 77032 | thiosulfate sulfurtransferase (rhodanese)-like domain containing 3 [Source:MGI Symbol;Acc:MGI:1924282] | Tstd3 | protein_coding |
| ENSMUSG00000028264 | 132.782865 | -0.7786595 | 0.334065 | 0.0009751 | 0.01320038 | Down | 67652 | sperm acrosome associated 1 [Source:MGI Symbol;Acc:MGI:1914902] | Spaca1 | protein_coding |
| ENSMUSG00000028273 | 1800.76139 | 0.20340751 | 0.07990037 | 0.00556028 | 0.04054164 | Up | 56376 | PDZ and LIM domain 5 [Source:MGI Symbol;Acc:MGI:1927489] | Pdlim5 | protein_coding |
| ENSMUSG00000028278 | 23.8291966 | 0.17929067 | 0.3337057 | 0.00437306 | 0.03446687 | Up | 52187 | Ras-related GTP binding D [Source:MGI Symbol;Acc:MGI:1098604] | Rragd | protein_coding |
| ENSMUSG00000028282 | 257.407575 | 0.43867985 | 0.14335066 | 0.00030786 | 0.00618692 | Up | 26885 | caspase 8 associated protein 2 [Source:MGI Symbol;Acc:MGI:1349399] | Casp8ap2 | protein_coding |
| ENSMUSG00000028334 | 432.765476 | -0.8176916 | 0.1871717 | 6.84E-07 | 0.00011158 | Down | 94181 | N-acetylneuraminic acid synthase (sialic acid synthase) [Source:MGI Symbol;Acc:MGI:2149820] | Nans | protein_coding |
| ENSMUSG00000028345 | 337.376609 | 0.28624113 | 0.10557354 | 0.00198306 | 0.0207036 | Up | 269536 | testis expressed gene 10 [Source:MGI Symbol;Acc:MGI:1344413] | Tex10 | protein_coding |
| ENSMUSG00000028367 | 2133.16667 | -0.4040037 | 0.17448074 | 0.00288311 | 0.02640145 | Down | 22166 | thioredoxin 1 [Source:MGI Symbol;Acc:MGI:98874] | Txn1 | protein_coding |
| ENSMUSG00000028378 | 206.236508 | -0.7319447 | 0.27241135 | 0.00043084 | 0.00757464 | Down | 67103 | prostaglandin reductase 1 [Source:MGI Symbol;Acc:MGI:1914353] | Ptgr1 | protein_coding |
| ENSMUSG00000028381 | 585.414133 | 0.34833969 | 0.15860492 | 0.00504894 | 0.03798203 | Up | 22234 | UDP-glucose ceramide glucosyltransferase [Source:MGI Symbol;Acc:MGI:1332243] | Ugcg | protein_coding |
| ENSMUSG00000028382 | 2205.23374 | 0.31760895 | 0.12302495 | 0.00236908 | 0.02320131 | Up | 230257 | polypyrimidine tract binding protein 3 [Source:MGI Symbol;Acc:MGI:1923334] | Ptbp3 | protein_coding |
| ENSMUSG00000028394 | 785.511177 | -0.559786 | 0.12721764 | 1.18E-06 | 0.00016368 | Down | 59001 | polymerase (DNA directed), epsilon 3 (p17 subunit) [Source:MGI Symbol;Acc:MGI:1933378] | Pole3 | protein_coding |
| ENSMUSG00000028398 | 576.925913 | -0.4000005 | 0.15351193 | 0.00140605 | 0.01663216 | Down | 66928 | distal membrane arm assembly complex 1 [Source:MGI Symbol;Acc:MGI:1914178] | Dmac1 | protein_coding |
| ENSMUSG00000028419 | 1454.50197 | -0.3406305 | 0.15728665 | 0.00578819 | 0.04153228 | Down | 76959 | charged multivesicular body protein 5 [Source:MGI Symbol;Acc:MGI:1924209] | Chmp5 | protein_coding |
| ENSMUSG00000028445 | 110.061458 | -1.5591707 | 0.29155684 | 4.45E-09 | 3.64E-06 | Down | 69638 | energy homeostasis associated [Source:MGI Symbol;Acc:MGI:1916888] | Enho | protein_coding |
| ENSMUSG00000028461 | 190.975178 | -0.3498943 | 0.17260164 | 0.00731871 | 0.04888499 | Down | 622404 | coiled-coil domain containing 107 [Source:MGI Symbol;Acc:MGI:1913423] | Ccdc107 | protein_coding |
| ENSMUSG00000028466 | 793.50992 | -0.5539677 | 0.14481164 | 1.25E-05 | 0.00079322 | Down | 12913 | cAMP responsive element binding protein 3 [Source:MGI Symbol;Acc:MGI:99946] | Creb3 | protein_coding |
| ENSMUSG00000028478 | 3006.26759 | -0.4080328 | 0.16046765 | 0.00163001 | 0.01812427 | Down | 12757 | clathrin, light polypeptide (Lca) [Source:MGI Symbol;Acc:MGI:894297] | Clta | protein_coding |
| ENSMUSG00000028518 | 343.169968 | 0.5927777 | 0.17652482 | 6.88E-05 | 0.00226099 | Up | 108079 | protein kinase, AMP-activated, alpha 2 catalytic subunit [Source:MGI Symbol;Acc:MGI:1336173] | Prkaa2 | protein_coding |
| ENSMUSG00000028521 | 221.65558 | 0.39435378 | 0.15044566 | 0.00142147 | 0.01674536 | Up | 242585 | solute carrier family 35 (UDP-glucuronic acid/UDP-N-acetylgalactosamine dual transporter), member D1 [Source:MGI Symbol;Acc:MGI:2140361] | Slc35d1 | protein_coding |
| ENSMUSG00000028522 | 1060.0009 | 0.29313162 | 0.10497389 | 0.00153509 | 0.01748044 | Up | 71148 | MEIR1 treanscription regulator [Source:MGI Symbol;Acc:MGI:1918398] | Mier1 | protein_coding |
| ENSMUSG00000028538 | 1319.21964 | -0.4225196 | 0.15826961 | 0.00107421 | 0.01392696 | Down | 20441 | ST3 beta-galactoside alpha-2,3-sialyltransferase 3 [Source:MGI Symbol;Acc:MGI:1316659] | St3gal3 | protein_coding |
| ENSMUSG00000028541 | 219.854294 | -0.5408698 | 0.29024627 | 0.00431891 | 0.03425813 | Down | 53418 | UDP-Gal:betaGlcNAc beta 1,4- galactosyltransferase, polypeptide 2 [Source:MGI Symbol;Acc:MGI:1858493] | B4galt2 | protein_coding |
| ENSMUSG00000028558 | 15.0488769 | -1.9290371 | 0.64750992 | 0.00011248 | 0.00313422 | Down | 108802 | calreticulin 4 [Source:MGI Symbol;Acc:MGI:2140435] | Calr4 | protein_coding |
| ENSMUSG00000028565 | 1636.25673 | 0.33966178 | 0.16258561 | 0.00702508 | 0.0475708 | Up | 18027 | nuclear factor I/A [Source:MGI Symbol;Acc:MGI:108056] | Nfia | protein_coding |
| ENSMUSG00000028582 | 698.676695 | -0.2630588 | 0.0893435 | 0.00092012 | 0.01271037 | Down | 319965 | coiled-coil and C2 domain containing 1B [Source:MGI Symbol;Acc:MGI:2443076] | Cc2d1b | protein_coding |
| ENSMUSG00000028597 | 360.337495 | -0.7155213 | 0.15659076 | 3.84E-07 | 7.34E-05 | Down | 67305 | glutathione peroxidase 7 [Source:MGI Symbol;Acc:MGI:1914555] | Gpx7 | protein_coding |
| ENSMUSG00000028608 | 647.193032 | -0.2930834 | 0.12497639 | 0.00481851 | 0.03690556 | Down | 74098 | CXXC motif containing zinc binding protein [Source:MGI Symbol;Acc:MGI:1921348] | Czib | protein_coding |
| ENSMUSG00000028630 | 825.694668 | 0.29241115 | 0.09784129 | 0.0008355 | 0.01191802 | Up | 69181 | dual-specificity tyrosine-(Y)-phosphorylation regulated kinase 2 [Source:MGI Symbol;Acc:MGI:1330301] | Dyrk2 | protein_coding |
| ENSMUSG00000028641 | 862.272933 | -0.438521 | 0.14517737 | 0.00034474 | 0.00667501 | Down | 56401 | prolyl 3-hydroxylase 1 [Source:MGI Symbol;Acc:MGI:1888921] | P3h1 | protein_coding |
| ENSMUSG00000028643 | 247.450888 | -0.7650436 | 0.16600185 | 3.01E-07 | 6.46E-05 | Down | 69216 | small vasohibin binding protein [Source:MGI Symbol;Acc:MGI:1916466] | Svbp | protein_coding |
| ENSMUSG00000028664 | 405.780058 | -0.498877 | 0.26540794 | 0.00499545 | 0.03781504 | Down | 13844 | Eph receptor B2 [Source:MGI Symbol;Acc:MGI:99611] | Ephb2 | protein_coding |
| ENSMUSG00000028669 | 542.865134 | -0.2971086 | 0.13393577 | 0.00691088 | 0.04703281 | Down | 66193 | PITH (C-terminal proteasome-interacting domain of thioredoxin-like) domain containing 1 [Source:MGI Symbol;Acc:MGI:1913443] | Pithd1 | protein_coding |
| ENSMUSG00000028670 | 739.135701 | -0.520923 | 0.16724438 | 0.00019556 | 0.00460413 | Down | 26394 | lysophospholipase 2 [Source:MGI Symbol;Acc:MGI:1347000] | Lypla2 | protein_coding |
| ENSMUSG00000028672 | 824.947026 | -0.2830599 | 0.12920079 | 0.00741081 | 0.04931935 | Down | 15356 | 3-hydroxy-3-methylglutaryl-Coenzyme A lyase [Source:MGI Symbol;Acc:MGI:96158] | Hmgcl | protein_coding |
| ENSMUSG00000028673 | 2349.68327 | -0.3443507 | 0.11098221 | 0.00045667 | 0.00785873 | Down | 71665 | fucosidase, alpha-L- 1, tissue [Source:MGI Symbol;Acc:MGI:95593] | Fuca1 | protein_coding |
| ENSMUSG00000028692 | 7768.12445 | -0.3596476 | 0.11711395 | 0.00037522 | 0.00703871 | Down | 58810 | aldo-keto reductase family 1, member A1 (aldehyde reductase) [Source:MGI Symbol;Acc:MGI:1929955] | Akr1a1 | protein_coding |
| ENSMUSG00000028743 | 708.032018 | -0.4338654 | 0.22956436 | 0.00575991 | 0.04143785 | Down | 110198 | aldo-keto reductase family 7, member A5 (aflatoxin aldehyde reductase) [Source:MGI Symbol;Acc:MGI:107796] | Akr7a5 | protein_coding |
| ENSMUSG00000028751 | 176.716444 | -1.1164853 | 0.43434033 | 0.00039766 | 0.00725598 | Down | 26970 | phospholipase A2, group IIE [Source:MGI Symbol;Acc:MGI:1349660] | Pla2g2e | protein_coding |
| ENSMUSG00000028756 | 4355.50124 | -0.5368195 | 0.15425931 | 4.90E-05 | 0.00186708 | Down | 68943 | PTEN induced putative kinase 1 [Source:MGI Symbol;Acc:MGI:1916193] | Pink1 | protein_coding |
| ENSMUSG00000028757 | 2612.26934 | -0.3019498 | 0.09378088 | 0.00043036 | 0.0075744 | Down | 13200 | dolichyl-di-phosphooligosaccharide-protein glycotransferase [Source:MGI Symbol;Acc:MGI:1194508] | Ddost | protein_coding |
| ENSMUSG00000028759 | 2922.17672 | 0.16995606 | 0.06626338 | 0.0058533 | 0.04177802 | Up | 15441 | heterochromatin protein 1, binding protein 3 [Source:MGI Symbol;Acc:MGI:109369] | Hp1bp3 | protein_coding |
| ENSMUSG00000028772 | 657.389842 | -0.2946939 | 0.1140723 | 0.00265672 | 0.02497591 | Down | 619605 | zinc finger, CCHC domain containing 17 [Source:MGI Symbol;Acc:MGI:1919955] | Zcchc17 | protein_coding |
| ENSMUSG00000028779 | 1651.38018 | -0.3167222 | 0.11139701 | 0.00111228 | 0.01424975 | Down | 67898 | penta-EF hand domain containing 1 [Source:MGI Symbol;Acc:MGI:1915148] | Pef1 | protein_coding |
| ENSMUSG00000028785 | 41.0803679 | 1.2213238 | 0.7695564 | 0.00268668 | 0.02519925 | Up | 15444 | hippocalcin [Source:MGI Symbol;Acc:MGI:1336200] | Hpca | protein_coding |
| ENSMUSG00000028789 | 453.451519 | -0.455549 | 0.24050229 | 0.00548417 | 0.0401592 | Down | 242669 | antizyme inhibitor 2 [Source:MGI Symbol;Acc:MGI:2442093] | Azin2 | protein_coding |
| ENSMUSG00000028793 | 981.34805 | -0.388656 | 0.12935496 | 0.00046134 | 0.00789467 | Down | 75234 | ring finger protein 19B [Source:MGI Symbol;Acc:MGI:1922484] | Rnf19b | protein_coding |

| | | | | | | | | | | |
|---|---|---|---|---|---|---|---|---|---|---|
| ENSMUSG00000028821 | 1067.38315 | -0.390937 | 0.14217625 | 0.00098332 | 0.01325648 | Down | 68592 | SYF2 homolog, RNA splicing factor (S. cerevisiae) [Source:MGI Symbol;Acc:MGI:1915842] | Syf2 | protein_coding |
| ENSMUSG00000028822 | 2238.77438 | -0.3633684 | 0.14409981 | 0.00208467 | 0.02129902 | Down | 71817 | transmembrane protein 50A [Source:MGI Symbol;Acc:MGI:1919067] | Tmem50a | protein_coding |
| ENSMUSG00000028843 | 854.467777 | -0.5267212 | 0.20982203 | 0.00113222 | 0.0143807 | Down | 73723 | SH3 domain binding glutamic acid-rich protein-like 3 [Source:MGI Symbol;Acc:MGI:1920973] | Sh3bgrl3 | protein_coding |
| ENSMUSG00000028849 | 3901.54891 | -0.5310212 | 0.21480513 | 0.00121889 | 0.01508817 | Down | 245877 | MAP7 domain containing 1 [Source:MGI Symbol;Acc:MGI:2384297] | Map7d1 | protein_coding |
| ENSMUSG00000028851 | 1163.63111 | -0.4745557 | 0.18361307 | 0.00114554 | 0.01448198 | Down | 18221 | nudC nuclear distribution protein [Source:MGI Symbol;Acc:MGI:106014] | Nudc | protein_coding |
| ENSMUSG00000028898 | 484.785497 | -0.4520175 | 0.21184308 | 0.00351513 | 0.02997548 | Down | 71787 | tRNA selenocysteine 1 associated protein 1 [Source:MGI Symbol;Acc:MGI:1919037] | Trnau1ap | protein_coding |
| ENSMUSG00000028910 | 1215.8308 | -0.4115211 | 0.1623723 | 0.00160922 | 0.01797919 | Down | 26922 | mitochondrial trans-2-enoyl-CoA reductase [Source:MGI Symbol;Acc:MGI:1349441] | Mecr | protein_coding |
| ENSMUSG00000028937 | 549.703384 | -0.4324242 | 0.12653756 | 8.65E-05 | 0.00264502 | Down | 70025 | acyl-CoA thioesterase 7 [Source:MGI Symbol;Acc:MGI:1917275] | Acot7 | protein_coding |
| ENSMUSG00000028957 | 1040.37174 | 1.10720336 | 0.35604649 | 8.61E-05 | 0.00264502 | Up | 18628 | period circadian clock 3 [Source:MGI Symbol;Acc:MGI:1277134] | Per3 | protein_coding |
| ENSMUSG00000028958 | 105.61853 | -0.4050976 | 0.19509418 | 0.00491235 | 0.03735638 | Down | 64295 | transmembrane and ubiquitin-like domain containing 1 [Source:MGI Symbol;Acc:MGI:1923764] | Tmub1 | protein_coding |
| ENSMUSG00000028998 | 384.950259 | -0.5950832 | 0.30990213 | 0.00334237 | 0.02895829 | Down | 66169 | translocase of outer mitochondrial membrane 7 [Source:MGI Symbol;Acc:MGI:1913419] | Tomm7 | protein_coding |
| ENSMUSG00000029029 | 145.750524 | -0.4014784 | 0.17276754 | 0.002915 | 0.02655961 | Down | 59002 | WD repeat containing, antisense to Trp73 [Source:MGI Symbol;Acc:MGI:1891749] | Wrap73 | protein_coding |
| ENSMUSG00000029030 | 2759.22054 | -0.5045481 | 0.09670378 | 2.30E-08 | 9.39E-06 | Down | 67808 | transformation related protein 63 regulated like [Source:MGI Symbol;Acc:MGI:1915058] | Tprgl | protein_coding |
| ENSMUSG00000029059 | 1481.11287 | -0.7740123 | 0.33251328 | 0.00098676 | 0.01327032 | Down | 66469 | peroxiredoxin like 2B [Source:MGI Symbol;Acc:MGI:1913719] | Prxl2b | protein_coding |
| ENSMUSG00000029066 | 872.987141 | -0.5282011 | 0.19174737 | 0.00056242 | 0.00907293 | Down | 66448 | mitochondrial ribosomal protein L20 [Source:MGI Symbol;Acc:MGI:2137221] | Mrpl20 | protein_coding |
| ENSMUSG00000029068 | 3989.25162 | 0.32173566 | 0.11262705 | 0.00103234 | 0.01355327 | Up | 56036 | cyclin L2 [Source:MGI Symbol;Acc:MGI:1927119] | Ccnl2 | protein_coding |
| ENSMUSG00000029070 | 3011.97743 | -0.6250592 | 0.17729497 | 3.71E-05 | 0.00157811 | Down | 74761 | matrix-remodelling associated 8 [Source:MGI Symbol;Acc:MGI:1922011] | Mxra8 | protein_coding |
| ENSMUSG00000029094 | 2146.0946 | 0.38336205 | 0.16865811 | 0.0037368 | 0.03117835 | Up | 70292 | actin filament associated protein 1 [Source:MGI Symbol;Acc:MGI:1917542] | Afap1 | protein_coding |
| ENSMUSG00000029096 | 11328.1998 | -0.4858833 | 0.20789657 | 0.0022349 | 0.02230091 | Down | 78558 | HtrA serine peptidase 3 [Source:MGI Symbol;Acc:MGI:1925808] | Htra3 | protein_coding |
| ENSMUSG00000029119 | 1715.83402 | -0.4097767 | 0.15691866 | 0.00128604 | 0.01560249 | Down | 17160 | mannosidase 2, alpha B2 [Source:MGI Symbol;Acc:MGI:1195262] | Man2b2 | protein_coding |
| ENSMUSG00000029145 | 411.644309 | -0.4065107 | 0.14807481 | 0.00093866 | 0.01292255 | Down | 13667 | eukaryotic translation initiation factor 2B, subunit 4 delta [Source:MGI Symbol;Acc:MGI:95300] | Eif2b4 | protein_coding |
| ENSMUSG00000029146 | 2010.30835 | -0.3079578 | 0.10415019 | 0.00081057 | 0.01167356 | Down | 266781 | sorting nexin 17 [Source:MGI Symbol;Acc:MGI:2387801] | Snx17 | protein_coding |
| ENSMUSG00000029169 | 1909.67672 | 0.29088034 | 0.09380457 | 0.00057199 | 0.00918173 | Up | 13204 | DEAH (Asp-Glu-Ala-His) box polypeptide 15 [Source:MGI Symbol;Acc:MGI:1099786] | Dhx15 | protein_coding |
| ENSMUSG00000029178 | 1602.41115 | 0.40263215 | 0.12469175 | 0.00022027 | 0.00499772 | Up | 16599 | Kruppel-like factor 3 (basic) [Source:MGI Symbol;Acc:MGI:1342773] | Klf3 | protein_coding |
| ENSMUSG00000029185 | 943.766935 | -0.36778 | 0.11680901 | 0.00031409 | 0.0062915 | Down | 68303 | family with sequence similarity 114, member A1 [Source:MGI Symbol;Acc:MGI:1915553] | Fam114a1 | protein_coding |
| ENSMUSG00000029207 | 671.119514 | 0.41811334 | 0.16487403 | 0.00158122 | 0.01778474 | Up | 11787 | amyloid beta (A4) precursor protein-binding, family B, member 2 [Source:MGI Symbol;Acc:MGI:108405] | Apbb2 | protein_coding |
| ENSMUSG00000029223 | 133.324628 | -0.5398263 | 0.22207363 | 0.00128521 | 0.01560249 | Down | 22223 | ubiquitin carboxy-terminal hydrolase L1 [Source:MGI Symbol;Acc:MGI:103149] | Uchl1 | protein_coding |
| ENSMUSG00000029287 | 4222.2641 | 0.44383207 | 0.14969588 | 0.0004402 | 0.00769298 | Up | 21814 | transforming growth factor, beta receptor III [Source:MGI Symbol;Acc:MGI:104637] | Tgfbr3 | protein_coding |
| ENSMUSG00000029290 | 545.314344 | 0.27517896 | 0.12195985 | 0.00706338 | 0.04775566 | Up | 54367 | zinc finger protein 326 [Source:MGI Symbol;Acc:MGI:1927246] | Zfp326 | protein_coding |
| ENSMUSG00000029291 | 437.518394 | 0.37359348 | 0.1018948 | 4.92E-05 | 0.00187013 | Up | 52822 | RUN and FYVE domain containing 3 [Source:MGI Symbol;Acc:MGI:106484] | Rufy3 | protein_coding |
| ENSMUSG00000029310 | 820.122049 | -0.5166548 | 0.1830925 | 0.00053021 | 0.00877232 | Down | 74167 | nudix (nucleoside diphosphate linked moiety X)-type motif 9 [Source:MGI Symbol;Acc:MGI:1921417] | Nudt9 | protein_coding |
| ENSMUSG00000029314 | 1825.40633 | 0.45253309 | 0.19448556 | 0.00245084 | 0.02374113 | Up | 231510 | glycerol-3-phosphate acyltransferase 3 [Source:MGI Symbol;Acc:MGI:3603816] | Gpat3 | protein_coding |
| ENSMUSG00000029319 | 1578.72505 | -0.3821056 | 0.11764917 | 0.00018821 | 0.00452448 | Down | 71883 | coenzyme Q2 4-hydroxybenzoate polyprenyltransferase [Source:MGI Symbol;Acc:MGI:1919133] | Coq2 | protein_coding |
| ENSMUSG00000029335 | 1077.66541 | -1.3831275 | 0.35776322 | 5.21E-06 | 0.00043202 | Down | 110075 | bone morphogenetic protein 3 [Source:MGI Symbol;Acc:MGI:88179] | Bmp3 | protein_coding |
| ENSMUSG00000029344 | 1039.59535 | -0.3660772 | 0.12060677 | 0.00043381 | 0.00760215 | Down | 22022 | protein-tyrosine sulfotransferase 2 [Source:MGI Symbol;Acc:MGI:1309516] | Tpst2 | protein_coding |
| ENSMUSG00000029361 | 43.0283918 | -0.7217107 | 0.34216651 | 0.0017797 | 0.01922291 | Down | 18125 | nitric oxide synthase 1, neuronal [Source:MGI Symbol;Acc:MGI:97360] | Nos1 | protein_coding |
| ENSMUSG00000029364 | 1114.0822 | -0.2528554 | 0.07762264 | 0.00044172 | 0.00769517 | Down | 59043 | WD repeat and SOCS box-containing 2 [Source:MGI Symbol;Acc:MGI:2144041] | Wsb2 | protein_coding |
| ENSMUSG00000029370 | 32.0033739 | 1.33768011 | 0.67853272 | 0.00139293 | 0.01655278 | Up | 73246 | Ras association (RalGDS/AF-6) domain family member 6 [Source:MGI Symbol;Acc:MGI:1920496] | Rassf6 | protein_coding |
| ENSMUSG00000029402 | 76.3383286 | -0.5627523 | 0.3733531 | 0.0069606 | 0.04730581 | Down | 76167 | small nuclear ribonucleoprotein 35 (U11/U12) [Source:MGI Symbol;Acc:MGI:1923417] | Snrnp35 | protein_coding |
| ENSMUSG00000029404 | 494.121081 | -0.4820358 | 0.196947 | 0.00163407 | 0.01815692 | Down | 65105 | ADP-ribosylation factor-like 6 interacting protein 4 [Source:MGI Symbol;Acc:MGI:1929500] | Arl6ip4 | protein_coding |
| ENSMUSG00000029413 | 2229.82027 | -0.4089001 | 0.11430214 | 6.26E-05 | 0.00215125 | Down | 67111 | N-acylethanolamine acid amidase [Source:MGI Symbol;Acc:MGI:1914361] | Naaa | protein_coding |
| ENSMUSG00000029415 | 426.544004 | 0.27124036 | 0.10710503 | 0.00363838 | 0.03060877 | Up | 231452 | SDA1 domain containing 1 [Source:MGI Symbol;Acc:MGI:2140779] | Sdad1 | protein_coding |
| ENSMUSG00000029440 | 352.35501 | -0.4021116 | 0.17588495 | 0.00312843 | 0.02784593 | Down | 67151 | proteasome (prosome, macropain) 26S subunit, non-ATPase, 9 [Source:MGI Symbol;Acc:MGI:1914401] | Psmd9 | protein_coding |
| ENSMUSG00000029465 | 1734.90237 | -0.3168675 | 0.14741619 | 0.00685042 | 0.04673877 | Down | 56378 | actin related protein 2/3 complex, subunit 3 [Source:MGI Symbol;Acc:MGI:1928375] | Arpc3 | protein_coding |
| ENSMUSG00000029470 | 956.360566 | -0.2643211 | 0.09140049 | 0.00118699 | 0.0148328 | Down | 18438 | purinergic receptor P2X, ligand-gated ion channel 4 [Source:MGI Symbol;Acc:MGI:1338859] | P2rx4 | protein_coding |
| ENSMUSG00000029484 | 1950.3334 | -0.780181 | 0.16490492 | 2.14E-07 | 5.19E-05 | Down | 11745 | annexin A3 [Source:MGI Symbol;Acc:MGI:1201378] | Anxa3 | protein_coding |
| ENSMUSG00000029490 | 283.231177 | -0.5993242 | 0.24223155 | 0.00099439 | 0.01330743 | Down | 243197 | major facilitator superfamily domain containing 7A [Source:MGI Symbol;Acc:MGI:2442629] | Mfsd7a | protein_coding |
| ENSMUSG00000029501 | 886.760149 | 0.16672367 | 0.06684429 | 0.00745958 | 0.04956276 | Up | 71782 | ankyrin repeat and LEM domain containing 2 [Source:MGI Symbol;Acc:MGI:1261856] | Ankle2 | protein_coding |
| ENSMUSG00000029535 | 549.585203 | -0.5643315 | 0.18026029 | 0.00016864 | 0.00419526 | Down | 69076 | TP53 regulated inhibitor of apoptosis 1 [Source:MGI Symbol;Acc:MGI:1916326] | Triap1 | protein_coding |
| ENSMUSG00000029538 | 539.776965 | -0.5328533 | 0.17615612 | 0.00024747 | 0.005418 | Down | 108014 | serine and arginine-rich splicing factor 9 [Source:MGI Symbol;Acc:MGI:104896] | Srsf9 | protein_coding |
| ENSMUSG00000029560 | 521.107074 | -0.311311 | 0.13587766 | 0.00517024 | 0.03852779 | Down | 231834 | sorting nexin 8 [Source:MGI Symbol;Acc:MGI:2443816] | Snx8 | protein_coding |

| | | | | | | | | | | |
|---|---|---|---|---|---|---|---|---|---|---|
| ENSMUSG00000029570 | 679.785232 | -0.5383309 | 0.20965078 | 0.00093761 | 0.01291901 | Down | 16848 | LFNG O-fucosylpeptide 3-beta-N-acetylglucosaminyltransferase [Source:MGI Symbol;Acc:MGI:1095413] | Lfng | protein_coding |
| ENSMUSG00000029594 | 238.564611 | -0.4644389 | 0.11776591 | 1.07E-05 | 0.00072429 | Down | 74111 | RNA binding motif protein 19 [Source:MGI Symbol;Acc:MGI:1921361] | Rbm19 | protein_coding |
| ENSMUSG00000029596 | 271.964853 | -0.9364884 | 0.29794207 | 8.44E-05 | 0.00262016 | Down | 257635 | serine dehydratase-like [Source:MGI Symbol;Acc:MGI:2182607] | Sdsl | protein_coding |
| ENSMUSG00000029610 | 201.001545 | -0.2987587 | 0.12170262 | 0.00367246 | 0.03084754 | Down | 231872 | aminoacyl tRNA synthetase complex-interacting multifunctional protein 2 [Source:MGI Symbol;Acc:MGI:2385237] | Aimp2 | protein_coding |
| ENSMUSG00000029614 | 8199.90589 | -0.3090545 | 0.14146504 | 0.00514903 | 0.03841138 | Down | 19988 | ribosomal protein L6 [Source:MGI Symbol;Acc:MGI:108057] | Rpl6 | protein_coding |
| ENSMUSG00000029616 | 1235.29338 | -0.6793942 | 0.17962398 | 1.29E-05 | 0.00081719 | Down | 67397 | endoplasmic reticulum protein 29 [Source:MGI Symbol;Acc:MGI:1914647] | Erp29 | protein_coding |
| ENSMUSG00000029622 | 3877.36194 | -0.5318789 | 0.15953369 | 8.75E-05 | 0.00267235 | Down | 11867 | actin related protein 2/3 complex, subunit 1B [Source:MGI Symbol;Acc:MGI:1343142] | Arpc1b | protein_coding |
| ENSMUSG00000029647 | 739.458032 | 0.49994947 | 0.09995295 | 7.56E-08 | 2.28E-05 | Up | 72587 | PAN3 poly(A) specific ribonuclease subunit [Source:MGI Symbol;Acc:MGI:1919837] | Pan3 | protein_coding |
| ENSMUSG00000029648 | 1424.77099 | 0.64682567 | 0.23656211 | 0.00044625 | 0.00774495 | Up | 14254 | FMS-like tyrosine kinase 1 [Source:MGI Symbol;Acc:MGI:95558] | Flt1 | protein_coding |
| ENSMUSG00000029649 | 1538.63224 | -0.4121258 | 0.1821773 | 0.00325382 | 0.02854116 | Down | 66537 | proteasome maturation protein [Source:MGI Symbol;Acc:MGI:1913787] | Pomp | protein_coding |
| ENSMUSG00000029655 | 916.563204 | 0.26340489 | 0.09745002 | 0.00234191 | 0.02305135 | Up | 381695 | NEDD4 binding protein 2-like 2 [Source:MGI Symbol;Acc:MGI:2687207] | N4bp2l2 | protein_coding |
| ENSMUSG00000029658 | 40.8321986 | -0.1370456 | 0.27010337 | 0.00702819 | 0.04757208 | Down | 381693 | WD40 repeat domain 95 [Source:MGI Symbol;Acc:MGI:1923042] | Wdr95 | protein_coding |
| ENSMUSG00000029664 | 106.30743 | -1.2626155 | 0.39233749 | 5.50E-05 | 0.00197972 | Down | 21789 | tissue factor pathway inhibitor 2 [Source:MGI Symbol;Acc:MGI:108543] | Tfpi2 | protein_coding |
| ENSMUSG00000029670 | 308.535391 | 0.4594363 | 0.17923856 | 0.00123995 | 0.01523671 | Up | 71777 | inhibitor of growth family, member 3 [Source:MGI Symbol;Acc:MGI:1919027] | Ing3 | protein_coding |
| ENSMUSG00000029681 | 841.511642 | -0.3504409 | 0.16312967 | 0.00567999 | 0.04100955 | Down | 12054 | B cell CLL/lymphoma 7B [Source:MGI Symbol;Acc:MGI:1332238] | Bcl7b | protein_coding |
| ENSMUSG00000029684 | 1281.25257 | 0.38615396 | 0.08210414 | 4.98E-07 | 8.80E-05 | Up | 73178 | WASP like actin nucleation promoting factor [Source:MGI Symbol;Acc:MGI:1920428] | Wasl | protein_coding |
| ENSMUSG00000029701 | 2131.87686 | -0.369746 | 0.18578971 | 0.00609716 | 0.04300844 | Down | 68272 | RNA binding motif protein 28 [Source:MGI Symbol;Acc:MGI:2655711] | Rbm28 | protein_coding |
| ENSMUSG00000029713 | 4233.32052 | -0.3258259 | 0.14876304 | 0.00624063 | 0.04375442 | Down | 14693 | guanine nucleotide binding protein (G protein), beta 2 [Source:MGI Symbol;Acc:MGI:95784] | Gnb2 | protein_coding |
| ENSMUSG00000029716 | 251.282418 | -1.7274377 | 0.60428377 | 0.00014164 | 0.00369325 | Down | 50765 | transferrin receptor 2 [Source:MGI Symbol;Acc:MGI:1354956] | Tfr2 | protein_coding |
| ENSMUSG00000029718 | 1061.84234 | -0.4626798 | 0.17973639 | 0.00115976 | 0.01462098 | Down | 18542 | procollagen C-endopeptidase enhancer protein [Source:MGI Symbol;Acc:MGI:105099] | Pcolce | protein_coding |
| ENSMUSG00000029722 | 1244.53286 | -0.3792955 | 0.16335998 | 0.00290348 | 0.02651452 | Down | 231801 | ArfGAP with FG repeats 2 [Source:MGI Symbol;Acc:MGI:2443267] | Agfg2 | protein_coding |
| ENSMUSG00000029725 | 212.60121 | -0.8043466 | 0.22417167 | 2.16E-05 | 0.00112295 | Down | 69871 | protein phosphatase 1, regulatory subunit 35 [Source:MGI Symbol;Acc:MGI:1922853] | Ppp1r35 | protein_coding |
| ENSMUSG00000029729 | 419.249095 | 0.51208976 | 0.10644997 | 1.85E-07 | 4.70E-05 | Up | 74570 | zinc finger with KRAB and SCAN domains 1 [Source:MGI Symbol;Acc:MGI:1921820] | Zkscan1 | protein_coding |
| ENSMUSG00000029781 | 5536.60421 | -0.2192762 | 0.06606902 | 0.00040753 | 0.00737228 | Down | 27055 | FK506 binding protein 9 [Source:MGI Symbol;Acc:MGI:1350921] | Fkbp9 | protein_coding |
| ENSMUSG00000029802 | 437.874299 | 0.49095556 | 0.18169127 | 0.00076003 | 0.01122243 | Up | 26357 | ATP binding cassette subfamily G member 2 (Junior blood group) [Source:MGI Symbol;Acc:MGI:1347061] | Abcg2 | protein_coding |
| ENSMUSG00000029810 | 2129.08767 | -0.5935335 | 0.20930645 | 0.0003823 | 0.00711394 | Down | 65963 | transmembrane protein 176B [Source:MGI Symbol;Acc:MGI:1916348] | Tmem176b | protein_coding |
| ENSMUSG00000029815 | 401.17135 | -0.4573391 | 0.19190486 | 0.00194932 | 0.02046808 | Down | 75593 | mitochondrial assembly of ribosomal large subunit 1 [Source:MGI Symbol;Acc:MGI:1922843] | Malsu1 | protein_coding |
| ENSMUSG00000029823 | 2966.20094 | 0.59996305 | 0.11324822 | 1.18E-08 | 6.87E-06 | Up | 192196 | LUC7-like 2 (S. cerevisiae) [Source:MGI Symbol;Acc:MGI:2183260] | Luc7l2 | protein_coding |
| ENSMUSG00000029863 | 304.083322 | 0.38178431 | 0.14686264 | 0.00155158 | 0.01758923 | Up | 12366 | caspase 2 [Source:MGI Symbol;Acc:MGI:97295] | Casp2 | protein_coding |
| ENSMUSG00000029920 | 462.168878 | 0.41571301 | 0.15106615 | 0.00083804 | 0.01191802 | Up | 13990 | SWI/SNF-related, matrix-associated actin-dependent regulator of chromatin, subfamily a, containing DEAD/H box 1 [Source:MGI Symbol;Acc:MGI:95453] | Smarcad1 | protein_coding |
| ENSMUSG00000029993 | 725.591696 | -0.3305546 | 0.15842322 | 0.00727271 | 0.04864656 | Down | 56748 | NFU1 iron-sulfur cluster scaffold [Source:MGI Symbol;Acc:MGI:1913290] | Nfu1 | protein_coding |
| ENSMUSG00000030002 | 1792.88152 | 0.3147243 | 0.11893645 | 0.00199289 | 0.02076619 | Up | 72102 | dual specificity phosphatase 11 (RNA/RNP complex 1-interacting) [Source:MGI Symbol;Acc:MGI:1919352] | Dusp11 | protein_coding |
| ENSMUSG00000030016 | 1231.32737 | 0.32759173 | 0.15138524 | 0.00659297 | 0.04538255 | Up | 18139 | zinc finger protein 638 [Source:MGI Symbol;Acc:MGI:1203484] | Zfp638 | protein_coding |
| ENSMUSG00000030034 | 229.465352 | -1.0903356 | 0.29099737 | 9.19E-06 | 0.000655 | Down | 70020 | INO80 complex subunit B [Source:MGI Symbol;Acc:MGI:1917270] | Ino80b | protein_coding |
| ENSMUSG00000030035 | 807.210105 | -0.3156081 | 0.13015085 | 0.00410276 | 0.03307662 | Down | 22377 | WW domain binding protein 1 [Source:MGI Symbol;Acc:MGI:104710] | Wbp1 | protein_coding |
| ENSMUSG00000030036 | 403.347421 | -0.4043466 | 0.15828726 | 0.00157644 | 0.0177719 | Down | 57377 | mannosyl-oligosaccharide glucosidase [Source:MGI Symbol;Acc:MGI:1929872] | Mogs | protein_coding |
| ENSMUSG00000030079 | 370.297644 | -0.4971352 | 0.16769475 | 0.00033577 | 0.00657966 | Down | 56505 | RuvB-like protein 1 [Source:MGI Symbol;Acc:MGI:1928760] | Ruvbl1 | protein_coding |
| ENSMUSG00000030086 | 917.002132 | -0.4165488 | 0.14884217 | 0.00077588 | 0.01137555 | Down | 66098 | coiled-coil-helix-coiled-coil-helix domain containing 6 [Source:MGI Symbol;Acc:MGI:1913348] | Chchd6 | protein_coding |
| ENSMUSG00000030089 | 209.496315 | -0.4465583 | 0.21944174 | 0.00451782 | 0.03518314 | Down | 71699 | solute carrier family 41, member 3 [Source:MGI Symbol;Acc:MGI:1918949] | Slc41a3 | protein_coding |
| ENSMUSG00000030096 | 1598.65715 | 0.47888009 | 0.20061887 | 0.0018428 | 0.01966901 | Up | 21366 | solute carrier family 6 (neurotransmitter transporter, taurine), member 6 [Source:MGI Symbol;Acc:MGI:98488] | Slc6a6 | protein_coding |
| ENSMUSG00000030101 | 1081.75635 | -0.2891073 | 0.10029616 | 0.00125379 | 0.01530269 | Down | 58911 | sulfatase modifying factor 1 [Source:MGI Symbol;Acc:MGI:1889844] | Sumf1 | protein_coding |
| ENSMUSG00000030108 | 1950.85332 | -0.3748912 | 0.16774312 | 0.00398961 | 0.03253576 | Down | 14412 | solute carrier family 6 (neurotransmitter transporter, GABA), member 13 [Source:MGI Symbol;Acc:MGI:95629] | Slc6a13 | protein_coding |
| ENSMUSG00000030116 | 1306.1739 | -0.4267576 | 0.18177819 | 0.00254474 | 0.02429407 | Down | 50530 | microfibrillar associated protein 5 [Source:MGI Symbol;Acc:MGI:1354387] | Mfap5 | protein_coding |
| ENSMUSG00000030120 | 3354.97041 | -0.3788367 | 0.16642918 | 0.00403629 | 0.03278479 | Down | 30853 | myeloid leukemia factor 2 [Source:MGI Symbol;Acc:MGI:1353554] | Mlf2 | protein_coding |
| ENSMUSG00000030122 | 8818.32083 | -0.5379035 | 0.2472033 | 0.00226046 | 0.02245945 | Down | 69202 | parathymosin [Source:MGI Symbol;Acc:MGI:1916452] | Ptms | protein_coding |
| ENSMUSG00000030177 | 226.80748 | 0.36346236 | 0.1823641 | 0.0075849 | 0.04998649 | Up | 67200 | coiled-coil domain containing 77 [Source:MGI Symbol;Acc:MGI:1914450] | Ccdc77 | protein_coding |
| ENSMUSG00000030189 | 4496.69013 | -0.3114201 | 0.12300009 | 0.00272866 | 0.02546069 | Down | 56449 | Y box protein 3 [Source:MGI Symbol;Acc:MGI:2137670] | Ybx3 | protein_coding |
| ENSMUSG00000030199 | 390.002368 | 0.32937918 | 0.14584366 | 0.00513988 | 0.03840728 | Up | 14011 | ets variant 6 [Source:MGI Symbol;Acc:MGI:109336] | Etv6 | protein_coding |
| ENSMUSG00000030203 | 474.021144 | 0.4320855 | 0.15736465 | 0.00084914 | 0.01203683 | Up | 70686 | dual specificity phosphatase 16 [Source:MGI Symbol;Acc:MGI:1917936] | Dusp16 | protein_coding |
| ENSMUSG00000030249 | 646.025811 | 0.43793987 | 0.22392031 | 0.00560448 | 0.04066318 | Up | 20928 | ATP-binding cassette, sub-family C (CFTR/MRP), member 9 [Source:MGI Symbol;Acc:MGI:1352630] | Abcc9 | protein_coding |

| | | | | | | | | | | |
|---|---|---|---|---|---|---|---|---|---|---|
| ENSMUSG00000030272 | 1665.11201 | -0.4310925 | 0.10353076 | 4.58E-06 | 0.00038572 | Down | 52163 | calcium/calmodulin-dependent protein kinase I [Source:MGI Symbol;Acc:MGI:1098535] | Camk1 | protein_coding |
| ENSMUSG00000030275 | 2150.8265 | 0.37308269 | 0.10782181 | 0.00011777 | 0.00326479 | Up | 75320 | ethanolamine kinase 1 [Source:MGI Symbol;Acc:MGI:1922570] | Etnk1 | protein_coding |
| ENSMUSG00000030278 | 71085.6573 | -0.3576041 | 0.12692873 | 0.00095446 | 0.01305827 | Down | 14311 | cell death-inducing DFFA-like effector c [Source:MGI Symbol;Acc:MGI:95585] | Cidec | protein_coding |
| ENSMUSG00000030281 | 841.796353 | -0.5817197 | 0.1254957 | 3.63E-07 | 7.26E-05 | Down | 171095 | interleukin 17 receptor C [Source:MGI Symbol;Acc:MGI:2159336] | Il17rc | protein_coding |
| ENSMUSG00000030284 | 1722.25873 | -0.5303306 | 0.15970881 | 9.24E-05 | 0.00278006 | Down | 171508 | cysteine-rich with EGF-like domains 1 [Source:MGI Symbol;Acc:MGI:2152539] | Creld1 | protein_coding |
| ENSMUSG00000030301 | 752.657903 | -0.2983654 | 0.08491943 | 0.00012883 | 0.00346504 | Down | 67015 | coiled-coil domain containing 91 [Source:MGI Symbol;Acc:MGI:1914265] | Ccdc91 | protein_coding |
| ENSMUSG00000030304 | 560.253019 | 0.29900827 | 0.11847285 | 0.00320575 | 0.02825683 | Up | 67456 | ERGIC and golgi 2 [Source:MGI Symbol;Acc:MGI:1914706] | Ergic2 | protein_coding |
| ENSMUSG00000030306 | 569.857324 | 0.4354965 | 0.20798774 | 0.00409073 | 0.032996 | Up | 387314 | transmembrane and tetratricopeptide repeat containing 1 [Source:MGI Symbol;Acc:MGI:3039590] | Tmtc1 | protein_coding |
| ENSMUSG00000030313 | 427.995743 | 0.38819885 | 0.1956305 | 0.00658682 | 0.04537869 | Up | 320560 | DENN/MADD domain containing 5B [Source:MGI Symbol;Acc:MGI:2444273] | Dennd5b | protein_coding |
| ENSMUSG00000030317 | 8692.84642 | -0.4017315 | 0.1776253 | 0.00334052 | 0.02895775 | Down | 110595 | tissue inhibitor of metalloproteinase 4 [Source:MGI Symbol;Acc:MGI:109125] | Timp4 | protein_coding |
| ENSMUSG00000030327 | 577.690614 | 0.23355223 | 0.09539504 | 0.0056935 | 0.04107057 | Up | 67602 | NECAP endocytosis associated 1 [Source:MGI Symbol;Acc:MGI:1914852] | Necap1 | protein_coding |
| ENSMUSG00000030341 | 3800.96295 | -0.2491747 | 0.09510648 | 0.00342007 | 0.02938078 | Down | 21937 | tumor necrosis factor receptor superfamily, member 1a [Source:MGI Symbol;Acc:MGI:1314884] | Tnfrsf1a | protein_coding |
| ENSMUSG00000030401 | 700.884548 | -1.0629024 | 0.21418134 | 3.82E-08 | 1.41E-05 | Down | 20167 | reticulon 2 (Z-band associated protein) [Source:MGI Symbol;Acc:MGI:107612] | Rtn2 | protein_coding |
| ENSMUSG00000030403 | 878.356186 | -0.5780042 | 0.29328663 | 0.00319754 | 0.02819979 | Down | 22323 | vasodilator-stimulated phosphoprotein [Source:MGI Symbol;Acc:MGI:109268] | Vasp | protein_coding |
| ENSMUSG00000030431 | 199.530833 | -0.5952817 | 0.30396793 | 0.00310112 | 0.02769524 | Down | 664968 | transmembrane protein 238 [Source:MGI Symbol;Acc:MGI:1922935] | Tmem238 | protein_coding |
| ENSMUSG00000030432 | 3944.8632 | -0.7397577 | 0.28454448 | 0.00060892 | 0.00960103 | Down | 19943 | ribosomal protein L28 [Source:MGI Symbol;Acc:MGI:101839] | Rpl28 | protein_coding |
| ENSMUSG00000030509 | 292.964927 | 0.23044146 | 0.09411991 | 0.00600156 | 0.04254817 | Up | 117589 | ankyrin repeat and SOCS box-containing 7 [Source:MGI Symbol;Acc:MGI:2152835] | Asb7 | protein_coding |
| ENSMUSG00000030538 | 465.463185 | -0.4792871 | 0.17387672 | 0.00082457 | 0.01179143 | Down | 23991 | calcium and integrin binding 1 (calmyrin) [Source:MGI Symbol;Acc:MGI:1344418] | Cib1 | protein_coding |
| ENSMUSG00000030541 | 2596.49261 | -0.4462165 | 0.10265891 | 1.62E-06 | 0.00019783 | Down | 269951 | isocitrate dehydrogenase 2 (NADP+), mitochondrial [Source:MGI Symbol;Acc:MGI:96414] | Idh2 | protein_coding |
| ENSMUSG00000030557 | 1566.88548 | 0.44442677 | 0.12770447 | 8.63E-05 | 0.00264502 | Up | 17258 | myocyte enhancer factor 2A [Source:MGI Symbol;Acc:MGI:99532] | Mef2a | protein_coding |
| ENSMUSG00000030559 | 120.082546 | -0.4619334 | 0.24839423 | 0.00600752 | 0.04255949 | Down | 72433 | RAB38, member RAS oncogene family [Source:MGI Symbol;Acc:MGI:1919683] | Rab38 | protein_coding |
| ENSMUSG00000030579 | 533.152336 | -0.5725201 | 0.3339173 | 0.00504703 | 0.03798203 | Down | 22177 | TYRO protein tyrosine kinase binding protein [Source:MGI Symbol;Acc:MGI:1277211] | Tyrobp | protein_coding |
| ENSMUSG00000030588 | 534.934526 | -0.6730992 | 0.14811587 | 4.71E-07 | 8.59E-05 | Down | 77254 | Yip1 interacting factor homolog B (S. cerevisiae) [Source:MGI Symbol;Acc:MGI:1924504] | Yif1b | protein_coding |
| ENSMUSG00000030591 | 2481.90262 | -0.3763959 | 0.12699916 | 0.00053768 | 0.00880853 | Down | 57296 | proteasome (prosome, macropain) 26S subunit, non-ATPase, 8 [Source:MGI Symbol;Acc:MGI:1888669] | Psmd8 | protein_coding |
| ENSMUSG00000030595 | 391.619904 | -0.6727205 | 0.13434917 | 4.27E-08 | 1.51E-05 | Down | 18036 | nuclear factor of kappa light polypeptide gene enhancer in B cells inhibitor, beta [Source:MGI Symbol;Acc:MGI:104752] | Nfkbib | protein_coding |
| ENSMUSG00000030604 | 171.195366 | 0.29480053 | 0.12260208 | 0.00443607 | 0.03479673 | Up | 71163 | zinc finger protein 626 [Source:MGI Symbol;Acc:MGI:1918413] | Zfp626 | protein_coding |
| ENSMUSG00000030605 | 9204.76412 | -0.953268 | 0.24370456 | 5.45E-06 | 0.00044782 | Down | 17304 | milk fat globule EGF and factor V/VIII domain containing [Source:MGI Symbol;Acc:MGI:102768] | Mfge8 | protein_coding |
| ENSMUSG00000030606 | 37.8551319 | -1.5829508 | 0.46563541 | 2.83E-05 | 0.00135817 | Down | 67666 | hyaluronan and proteoglycan link protein 3 [Source:MGI Symbol;Acc:MGI:1914916] | Hapln3 | protein_coding |
| ENSMUSG00000030609 | 456.146261 | -0.5443222 | 0.20998918 | 0.00080551 | 0.01163158 | Down | 68048 | apoptosis enhancing nuclease [Source:MGI Symbol;Acc:MGI:1915298] | Aen | protein_coding |
| ENSMUSG00000030611 | 258.602211 | -0.534339 | 0.20144657 | 0.00072874 | 0.0109412 | Down | 67994 | mitochondrial ribosomal protein S11 [Source:MGI Symbol;Acc:MGI:1915244] | Mrps11 | protein_coding |
| ENSMUSG00000030647 | 1092.59875 | -0.7906812 | 0.25836945 | 0.00013032 | 0.00349914 | Down | 68197 | NADH:ubiquinone oxidoreductase subunit C2 [Source:MGI Symbol;Acc:MGI:1344370] | Ndufc2 | protein_coding |
| ENSMUSG00000030681 | 1778.8659 | -0.6272549 | 0.13525281 | 3.55E-07 | 7.21E-05 | Down | 78388 | major vault protein [Source:MGI Symbol;Acc:MGI:1925638] | Mvp | protein_coding |
| ENSMUSG00000030682 | 1066.01801 | -0.2977438 | 0.08845356 | 0.00022697 | 0.00509977 | Down | 52858 | CDP-diacylglycerol--inositol 3-phosphatidyltransferase (phosphatidylinositol synthase) [Source:MGI Symbol;Acc:MGI:105491] | Cdipt | protein_coding |
| ENSMUSG00000030685 | 201.984831 | -0.3281689 | 0.12427328 | 0.0018648 | 0.0197925 | Down | 233877 | potassium channel tetramerisation domain containing 13 [Source:MGI Symbol;Acc:MGI:1923739] | Kctd13 | protein_coding |
| ENSMUSG00000030697 | 733.031497 | -0.437085 | 0.17090521 | 0.00137492 | 0.01638712 | Down | 56420 | protein phosphatase 4, catalytic subunit [Source:MGI Symbol;Acc:MGI:1891763] | Ppp4c | protein_coding |
| ENSMUSG00000030708 | 231.354741 | -0.6049003 | 0.26991873 | 0.00168285 | 0.01852162 | Down | 69387 | DnaJ heat shock protein family (Hsp40) member B13 [Source:MGI Symbol;Acc:MGI:1916637] | Dnajb13 | protein_coding |
| ENSMUSG00000030744 | 6019.51324 | -0.4715845 | 0.19930125 | 0.0019961 | 0.02078636 | Down | 27050 | ribosomal protein S3 [Source:MGI Symbol;Acc:MGI:1350917] | Rps3 | protein_coding |
| ENSMUSG00000030750 | 408.887276 | -0.4171942 | 0.18832151 | 0.0034622 | 0.02961744 | Down | 67711 | NSE1 homolog, SMC5-SMC6 complex component [Source:MGI Symbol;Acc:MGI:1914961] | Nsmce1 | protein_coding |
| ENSMUSG00000030779 | 1106.95053 | 0.37897461 | 0.12930436 | 0.00062108 | 0.00971065 | Up | 19647 | retinoblastoma binding protein 6, ubiquitin ligase [Source:MGI Symbol;Acc:MGI:894835] | Rbbp6 | protein_coding |
| ENSMUSG00000030814 | 250.779751 | -0.5268913 | 0.26339316 | 0.00352929 | 0.03004887 | Down | 12055 | B cell CLL/lymphoma 7C [Source:MGI Symbol;Acc:MGI:1332237] | Bcl7c | protein_coding |
| ENSMUSG00000030842 | 2014.35667 | -0.376275 | 0.150259 | 0.00204482 | 0.02109082 | Down | 66508 | late endosomal/lysosomal adaptor, MAPK and MTOR activator 1 [Source:MGI Symbol;Acc:MGI:1913758] | Lamtor1 | protein_coding |
| ENSMUSG00000030846 | 1015.55241 | 0.23790244 | 0.07779428 | 0.00096501 | 0.01311446 | Up | 21843 | Tia1 cytotoxic granule-associated RNA binding protein-like 1 [Source:MGI Symbol;Acc:MGI:107913] | Tial1 | protein_coding |
| ENSMUSG00000030878 | 1817.64544 | -0.3688144 | 0.15686371 | 0.00314715 | 0.02792596 | Down | 12585 | cerebellar degeneration-related 2 [Source:MGI Symbol;Acc:MGI:1100885] | Cdr2 | protein_coding |
| ENSMUSG00000030884 | 3202.65295 | 0.37993067 | 0.17252185 | 0.00436203 | 0.03446687 | Up | 67003 | ubiquinol cytochrome c reductase core protein 2 [Source:MGI Symbol;Acc:MGI:1914253] | Uqcrc2 | protein_coding |
| ENSMUSG00000030889 | 158.327822 | 0.86277325 | 0.56094769 | 0.00378304 | 0.03139658 | Up | 233813 | von Willebrand factor A domain containing 3A [Source:MGI Symbol;Acc:MGI:3041229] | Vwa3a | protein_coding |
| ENSMUSG00000030922 | 122.622762 | -0.3387448 | 0.15516156 | 0.00568855 | 0.04105306 | Down | 73919 | LYR motif containing 1 [Source:MGI Symbol;Acc:MGI:1921169] | Lyrm1 | protein_coding |
| ENSMUSG00000030929 | 161.165859 | 0.44137833 | 0.20967884 | 0.00401994 | 0.03269408 | Up | 71151 | exoribonuclease 2 [Source:MGI Symbol;Acc:MGI:1918401] | Eri2 | protein_coding |
| ENSMUSG00000030946 | 289.72294 | -0.2864619 | 0.10353894 | 0.00139966 | 0.01658455 | Down | 76429 | phospholysine phosphohistidine inorganic pyrophosphate phosphatase [Source:MGI Symbol;Acc:MGI:1923679] | Lhpp | protein_coding |
| ENSMUSG00000031012 | 696.967216 | 0.34286934 | 0.10379063 | 0.0002284 | 0.00511766 | Up | 12361 | calcium/calmodulin-dependent serine protein kinase (MAGUK family) [Source:MGI Symbol;Acc:MGI:1309489] | Cask | protein_coding |
| ENSMUSG00000031023 | 235.097915 | -0.4739738 | 0.20217673 | 0.00198433 | 0.0207036 | Down | 57373 | A kinase (PRKA) interacting protein 1 [Source:MGI Symbol;Acc:MGI:3041226] | Akip1 | protein_coding |

| | | | | | | | | | | |
|---|---|---|---|---|---|---|---|---|---|---|
| ENSMUSG00000031059 | 1407.55903 | -0.5487521 | 0.19756753 | 0.00054741 | 0.00891044 | Down | 104130 | NADH:ubiquinone oxidoreductase subunit B11 [Source:MGI Symbol;Acc:MGI:1349919] | Ndufb11 | protein_coding |
| ENSMUSG00000031072 | 398.774822 | -0.4835611 | 0.13885976 | 6.30E-05 | 0.00215944 | Down | 72284 | ABCE maturation factor [Source:MGI Symbol;Acc:MGI:1919534] | LTO1 | protein_coding |
| ENSMUSG00000031095 | 838.181906 | 0.35324686 | 0.14915183 | 0.00338698 | 0.02923564 | Up | 72584 | cullin 4B [Source:MGI Symbol;Acc:MGI:1919834] | Cul4b | protein_coding |
| ENSMUSG00000031134 | 704.552741 | 0.40958322 | 0.13442334 | 0.00039278 | 0.00720168 | Up | 19655 | RNA binding motif protein, X chromosome [Source:MGI Symbol;Acc:MGI:1343044] | Rbmx | protein_coding |
| ENSMUSG00000031142 | 10.224175 | -0.7690907 | 0.69947792 | 0.00715546 | 0.04813269 | Down | 54652 | calcium channel, voltage-dependent, alpha 1F subunit [Source:MGI Symbol;Acc:MGI:1859639] | Cacna1f | protein_coding |
| ENSMUSG00000031144 | 511.916065 | -1.7051182 | 0.34030556 | 2.62E-08 | 1.01E-05 | Down | 20977 | synaptophysin [Source:MGI Symbol;Acc:MGI:98467] | Syp | protein_coding |
| ENSMUSG00000031149 | 91.2847552 | -0.5325104 | 0.24779739 | 0.00252076 | 0.02414493 | Down | 54637 | PRA1 domain family 2 [Source:MGI Symbol;Acc:MGI:1859607] | Praf2 | protein_coding |
| ENSMUSG00000031156 | 515.532289 | -0.2432488 | 0.10357767 | 0.00676178 | 0.04627006 | Down | 22232 | solute carrier family 35 (UDP-galactose transporter), member A2 [Source:MGI Symbol;Acc:MGI:1345297] | Slc35a2 | protein_coding |
| ENSMUSG00000031157 | 439.665584 | -0.3312042 | 0.13973461 | 0.00374104 | 0.0311977 | Down | 54633 | polyglutamine binding protein 1 [Source:MGI Symbol;Acc:MGI:1859638] | Pqbp1 | protein_coding |
| ENSMUSG00000031204 | 68.3356542 | -0.6995174 | 0.44635081 | 0.00445676 | 0.03486891 | Down | 70392 | ankyrin repeat and SOCS box-containing 12 [Source:MGI Symbol;Acc:MGI:1917642] | Asb12 | protein_coding |
| ENSMUSG00000031230 | 15.0812845 | -1.1804068 | 0.64168424 | 0.00195728 | 0.02048935 | Down | 80903 | fibroblast growth factor 16 [Source:MGI Symbol;Acc:MGI:1931627] | Fgf16 | protein_coding |
| ENSMUSG00000031253 | 986.517373 | -0.7304961 | 0.22264919 | 6.87E-05 | 0.00226099 | Down | 68792 | sushi-repeat-containing protein, X-linked 2 [Source:MGI Symbol;Acc:MGI:1916042] | Srpx2 | protein_coding |
| ENSMUSG00000031256 | 603.675365 | 0.36807897 | 0.11821993 | 0.00036328 | 0.00689422 | Up | 108062 | cleavage stimulation factor, 3' pre-RNA subunit 2 [Source:MGI Symbol;Acc:MGI:1343054] | Cstf2 | protein_coding |
| ENSMUSG00000031266 | 158.427284 | -0.379775 | 0.15999184 | 0.00276599 | 0.0256852 | Down | 11605 | galactosidase, alpha [Source:MGI Symbol;Acc:MGI:1347344] | Gla | protein_coding |
| ENSMUSG00000031278 | 778.898641 | 0.26667329 | 0.11510454 | 0.00627502 | 0.04393255 | Up | 50790 | acyl-CoA synthetase long-chain family member 4 [Source:MGI Symbol;Acc:MGI:1354713] | Acsl4 | protein_coding |
| ENSMUSG00000031289 | 24.8787336 | -2.8492863 | 0.84549879 | 2.46E-05 | 0.00122074 | Down | 16165 | interleukin 13 receptor, alpha 2 [Source:MGI Symbol;Acc:MGI:1277954] | Il13ra2 | protein_coding |
| ENSMUSG00000031299 | 5251.83735 | 0.50966333 | 0.1792139 | 0.00047419 | 0.00805782 | Up | 18597 | pyruvate dehydrogenase E1 alpha 1 [Source:MGI Symbol;Acc:MGI:97532] | Pdha1 | protein_coding |
| ENSMUSG00000031333 | 400.913951 | 0.43228345 | 0.11864731 | 4.11E-05 | 0.00165511 | Up | 11306 | ATP-binding cassette, sub-family B (MDR/TAP), member 7 [Source:MGI Symbol;Acc:MGI:109533] | Abcb7 | protein_coding |
| ENSMUSG00000031355 | 162.606166 | 0.31716803 | 0.13991396 | 0.00537571 | 0.03959432 | Up | 11856 | Rho GTPase activating protein 6 [Source:MGI Symbol;Acc:MGI:1196332] | Arhgap6 | protein_coding |
| ENSMUSG00000031373 | 9195.45934 | 0.40149138 | 0.20284518 | 0.00625482 | 0.043835 | Up | 56078 | carbonic anhydrase 5b, mitochondrial [Source:MGI Symbol;Acc:MGI:1926249] | Car5b | protein_coding |
| ENSMUSG00000031383 | 69.9315892 | -1.2947607 | 0.40966258 | 5.88E-05 | 0.00204593 | Down | 75590 | dual specificity phosphatase 9 [Source:MGI Symbol;Acc:MGI:2387107] | Dusp9 | protein_coding |
| ENSMUSG00000031429 | 309.836576 | -0.3965261 | 0.16923502 | 0.00262209 | 0.02475068 | Down | 53380 | proteasome (prosome, macropain) 26S subunit, non-ATPase, 10 [Source:MGI Symbol;Acc:MGI:1858898] | Psmd10 | protein_coding |
| ENSMUSG00000031432 | 1617.22709 | -0.4279148 | 0.19738286 | 0.00375346 | 0.03126921 | Down | 19139 | phosphoribosyl pyrophosphate synthetase 1 [Source:MGI Symbol;Acc:MGI:97775] | Prps1 | protein_coding |
| ENSMUSG00000031433 | 102.700216 | 0.49775264 | 0.22426806 | 0.00249631 | 0.02401966 | Up | 237073 | RNA binding motif protein 41 [Source:MGI Symbol;Acc:MGI:2444923] | Rbm41 | protein_coding |
| ENSMUSG00000031490 | 4636.93137 | -0.5886161 | 0.17201752 | 5.64E-05 | 0.00200213 | Down | 13685 | eukaryotic translation initiation factor 4E binding protein 1 [Source:MGI Symbol;Acc:MGI:103267] | Eif4ebp1 | protein_coding |
| ENSMUSG00000031505 | 883.27275 | -0.3940136 | 0.1138733 | 9.41E-05 | 0.00280835 | Down | 69225 | NAD(P)HX dehydratase [Source:MGI Symbol;Acc:MGI:1913353] | Naxd | protein_coding |
| ENSMUSG00000031516 | 695.667936 | -0.2742267 | 0.11406175 | 0.00510245 | 0.03827231 | Down | 22428 | dynactin 6 [Source:MGI Symbol;Acc:MGI:1343154] | Dctn6 | protein_coding |
| ENSMUSG00000031521 | 643.720052 | -0.3283137 | 0.14041795 | 0.00428563 | 0.03411078 | Down | 11593 | aspartylglucosaminidase [Source:MGI Symbol;Acc:MGI:104873] | Aga | protein_coding |
| ENSMUSG00000031523 | 8406.85905 | 0.34352414 | 0.16306054 | 0.0068936 | 0.04695804 | Up | 50768 | deleted in liver cancer 1 [Source:MGI Symbol;Acc:MGI:1354949] | Dlc1 | protein_coding |
| ENSMUSG00000031532 | 2802.71749 | -0.4606204 | 0.11867942 | 1.29E-05 | 0.0008158 | Down | 67887 | store-operated calcium entry-associated regulatory factor [Source:MGI Symbol;Acc:MGI:1915137] | Saraf | protein_coding |
| ENSMUSG00000031540 | 1087.27096 | 0.49335122 | 0.19626766 | 0.00123626 | 0.0152312 | Up | 244349 | K(lysine) acetyltransferase 6A [Source:MGI Symbol;Acc:MGI:2442415] | Kat6a | protein_coding |
| ENSMUSG00000031556 | 1172.39344 | -0.5032937 | 0.11631391 | 1.65E-06 | 0.00019877 | Down | 69742 | TM2 domain containing 2 [Source:MGI Symbol;Acc:MGI:1916992] | Tm2d2 | protein_coding |
| ENSMUSG00000031577 | 245.797986 | 0.2448971 | 0.09931141 | 0.00516407 | 0.03849946 | Up | 234138 | TELO2 interacting protein 2 [Source:MGI Symbol;Acc:MGI:2384576] | Tti2 | protein_coding |
| ENSMUSG00000031592 | 1280.41193 | 0.41353689 | 0.17250581 | 0.00230794 | 0.02280563 | Up | 18536 | pericentriolar material 1 [Source:MGI Symbol;Acc:MGI:1277958] | Pcm1 | protein_coding |
| ENSMUSG00000031616 | 295.14685 | 0.57167962 | 0.24941197 | 0.00172758 | 0.01881773 | Up | 13617 | endothelin receptor type A [Source:MGI Symbol;Acc:MGI:105923] | Ednra | protein_coding |
| ENSMUSG00000031622 | 1252.40734 | -0.2872302 | 0.11460016 | 0.00355515 | 0.03014273 | Down | 20467 | transcriptional regulator, SIN3B (yeast) [Source:MGI Symbol;Acc:MGI:107158] | Sin3b | |
| ENSMUSG00000031633 | 4417.28823 | -0.3434513 | 0.14418549 | 0.00333803 | 0.02895618 | Down | 11739 | solute carrier family 25 (mitochondrial carrier, adenine nucleotide translocator), member 4 [Source:MGI Symbol;Acc:MGI:1353495] | Slc25a4 | protein_coding |
| ENSMUSG00000031661 | 632.394912 | -1.1119539 | 0.36824545 | 0.00011296 | 0.0031423 | Down | 93960 | naked cuticle 1 [Source:MGI Symbol;Acc:MGI:2135954] | Nkd1 | protein_coding |
| ENSMUSG00000031662 | 138.866917 | -0.7019567 | 0.42180486 | 0.00394613 | 0.03232446 | Down | 71607 | sorting nexin 20 [Source:MGI Symbol;Acc:MGI:1918857] | Snx20 | protein_coding |
| ENSMUSG00000031671 | 261.109958 | -0.3462509 | 0.10146379 | 0.00013989 | 0.00367613 | Down | 66083 | SET domain containing 6 [Source:MGI Symbol;Acc:MGI:1913333] | Setd6 | protein_coding |
| ENSMUSG00000031722 | 38997.0952 | -0.9457257 | 0.3417137 | 0.00026869 | 0.00569075 | Down | 15439 | haptoglobin [Source:MGI Symbol;Acc:MGI:96211] | Hp | protein_coding |
| ENSMUSG00000031731 | 1458.27221 | 0.27977576 | 0.10570553 | 0.0024655 | 0.02384055 | Up | 11765 | adaptor protein complex AP-1, gamma 1 subunit [Source:MGI Symbol;Acc:MGI:101919] | Ap1g1 | protein_coding |
| ENSMUSG00000031732 | 417.46261 | 0.42775573 | 0.11508819 | 3.13E-05 | 0.00144359 | Up | 244650 | PH domain and leucine rich repeat protein phosphatase 2 [Source:MGI Symbol;Acc:MGI:2444928] | Phlpp2 | protein_coding |
| ENSMUSG00000031750 | 485.242966 | -0.3637694 | 0.13386491 | 0.00115134 | 0.01453259 | Down | 76527 | interleukin 34 [Source:MGI Symbol;Acc:MGI:1923777] | Il34 | protein_coding |
| ENSMUSG00000031799 | 4893.94536 | -0.594161 | 0.17485083 | 5.87E-05 | 0.00204593 | Down | 326618 | tropomyosin 4 [Source:MGI Symbol;Acc:MGI:2449202] | Tpm4 | protein_coding |
| ENSMUSG00000031807 | 1454.84539 | -0.386871 | 0.16521061 | 0.00297321 | 0.02687799 | Down | 66171 | 6-phosphogluconolactonase [Source:MGI Symbol;Acc:MGI:1913421] | Pgls | protein_coding |
| ENSMUSG00000031813 | 331.545223 | -0.4620751 | 0.1723091 | 0.0008918 | 0.0124223 | Down | 73711 | multivesicular body subunit 12A [Source:MGI Symbol;Acc:MGI:1920961] | Mvb12a | protein_coding |
| ENSMUSG00000031821 | 132.133311 | -0.7512274 | 0.21930915 | 4.08E-05 | 0.00165266 | Down | 272551 | GINS complex subunit 2 (Psf2 homolog) [Source:MGI Symbol;Acc:MGI:1921019] | Gins2 | protein_coding |
| ENSMUSG00000031823 | 1013.0367 | -0.2267562 | 0.08848049 | 0.00462797 | 0.03588607 | Down | 102193 | zinc finger, DHHC domain containing 7 [Source:MGI Symbol;Acc:MGI:2142662] | Zdhhc7 | protein_coding |
| ENSMUSG00000031827 | 1156.5876 | -0.6390007 | 0.26677698 | 0.00116014 | 0.01462098 | Down | 72042 | coactosin-like 1 (Dictyostelium) [Source:MGI Symbol;Acc:MGI:1919292] | Cotl1 | protein_coding |
| ENSMUSG00000031839 | 2376.66023 | -0.3400702 | 0.1268701 | 0.00147873 | 0.01710968 | Down | 68196 | heat shock factor binding protein 1 [Source:MGI Symbol;Acc:MGI:1915446] | Hsbp1 | protein_coding |
| ENSMUSG00000031848 | 593.407712 | -0.5207554 | 0.22038118 | 0.00162685 | 0.01810151 | Down | 50783 | LSM4 homolog, U6 small nuclear RNA and mRNA degradation associated [Source:MGI Symbol;Acc:MGI:1354692] | Lsm4 | protein_coding |

| | | | | | | | | | | |
|---|---|---|---|---|---|---|---|---|---|---|
| ENSMUSG00000031851 | 333.270643 | -0.4522399 | 0.13942009 | 0.00016344 | 0.00410687 | Down | 66566 | nucleoside-triphosphatase, cancer-related [Source:MGI Symbol;Acc:MGI:1913816] | Ntpcr | protein_coding |
| ENSMUSG00000031858 | 1100.60411 | 0.34479914 | 0.14445293 | 0.00408163 | 0.03297173 | Up | 74549 | MAU2 sister chromatid cohesion factor [Source:MGI Symbol;Acc:MGI:1921799] | Mau2 | protein_coding |
| ENSMUSG00000031879 | 383.704739 | -0.7037725 | 0.16660108 | 1.86E-06 | 0.00021781 | Down | 68523 | cytosolic iron-sulfur assembly component 2B [Source:MGI Symbol;Acc:MGI:1915773] | Ciao2b | protein_coding |
| ENSMUSG00000031880 | 83.3798953 | -1.0754535 | 0.66013532 | 0.00274833 | 0.02560015 | Down | 56437 | Ras-related associated with diabetes [Source:MGI Symbol;Acc:MGI:1930943] | Rrad | protein_coding |
| ENSMUSG00000031887 | 248.296556 | -0.7146222 | 0.18711736 | 9.58E-06 | 0.00067695 | Down | 71609 | TNFRSF1A-associated via death domain [Source:MGI Symbol;Acc:MGI:109200] | Tradd | protein_coding |
| ENSMUSG00000031891 | 22.6606495 | -0.7371694 | 0.69894224 | 0.0072356 | 0.04849105 | Down | 15484 | hydroxysteroid 11-beta dehydrogenase 2 [Source:MGI Symbol;Acc:MGI:104720] | Hsd11b2 | protein_coding |
| ENSMUSG00000031897 | 797.355499 | -0.7314782 | 0.24960768 | 0.00021243 | 0.00484683 | Down | 19171 | proteasome (prosome, macropain) subunit, beta type 10 [Source:MGI Symbol;Acc:MGI:1096380] | Psmb10 | protein_coding |
| ENSMUSG00000031916 | 559.43399 | -0.2590948 | 0.10044719 | 0.00347375 | 0.02970053 | Down | 97484 | component of oligomeric golgi complex 8 [Source:MGI Symbol;Acc:MGI:2142885] | Cog8 | protein_coding |
| ENSMUSG00000031918 | 998.348114 | 0.19481679 | 0.0678405 | 0.00216857 | 0.02191828 | Up | 77116 | myotubularin related protein 2 [Source:MGI Symbol;Acc:MGI:1924366] | Mtmr2 | protein_coding |
| ENSMUSG00000031939 | 414.200064 | 0.50177634 | 0.12510548 | 7.35E-06 | 0.0005429 | Up | 75316 | TATA-box binding protein associated factor, RNA polymerase I, D [Source:MGI Symbol;Acc:MGI:1922566] | Taf1d | protein_coding |
| ENSMUSG00000031950 | 884.637506 | -0.3345099 | 0.14753439 | 0.00457598 | 0.03556782 | Down | 93739 | gamma-aminobutyric acid (GABA) A receptor-associated protein-like 2 [Source:MGI Symbol;Acc:MGI:1890602] | Gabarapl2 | protein_coding |
| ENSMUSG00000031972 | 1466.38697 | -1.6220182 | 0.54811775 | 0.00010695 | 0.00304801 | Down | 11459 | actin alpha 1, skeletal muscle [Source:MGI Symbol;Acc:MGI:87902] | Acta1 | protein_coding |
| ENSMUSG00000031974 | 567.696826 | 0.31356138 | 0.13581919 | 0.00511514 | 0.03829281 | Up | 56199 | ATP-binding cassette, sub-family B (MDR/TAP), member 10 [Source:MGI Symbol;Acc:MGI:1860508] | Abcb10 | protein_coding |
| ENSMUSG00000031986 | 501.372135 | 0.3210375 | 0.1070092 | 0.000669 | 0.01023337 | Up | 244666 | SprT-like N-terminal domain [Source:MGI Symbol;Acc:MGI:2685351] | Sprtn | protein_coding |
| ENSMUSG00000032009 | 1190.53441 | -0.3651746 | 0.17722422 | 0.00644318 | 0.04467328 | Down | 75747 | sestrin 3 [Source:MGI Symbol;Acc:MGI:1922997] | Sesn3 | protein_coding |
| ENSMUSG00000032011 | 402.840501 | -0.7660039 | 0.21264314 | 1.95E-05 | 0.00107201 | Down | 21838 | thymus cell antigen 1, theta [Source:MGI Symbol;Acc:MGI:98747] | Thy1 | protein_coding |
| ENSMUSG00000032014 | 776.502713 | -0.6306944 | 0.16516812 | 1.17E-05 | 0.00076318 | Down | 102644 | out at first homolog [Source:MGI Symbol;Acc:MGI:94852] | Oaf | protein_coding |
| ENSMUSG00000032030 | 852.943449 | 0.27358716 | 0.08528195 | 0.00042984 | 0.00757341 | Up | 75717 | cullin 5 [Source:MGI Symbol;Acc:MGI:1922967] | Cul5 | protein_coding |
| ENSMUSG00000032035 | 963.294173 | 0.41605647 | 0.179548 | 0.0027624 | 0.0256852 | Up | 23871 | E26 avian leukemia oncogene 1, 5' domain [Source:MGI Symbol;Acc:MGI:95455] | Ets1 | protein_coding |
| ENSMUSG00000032040 | 200.587165 | -0.4814179 | 0.14744459 | 0.00013807 | 0.00364704 | Down | 69305 | decapping enzyme, scavenger [Source:MGI Symbol;Acc:MGI:1916555] | Dcps | protein_coding |
| ENSMUSG00000032046 | 2153.42701 | -0.2912618 | 0.10132027 | 0.00112221 | 0.0143189 | Down | 76192 | abhydrolase domain containing 12 [Source:MGI Symbol;Acc:MGI:1923442] | Abhd12 | protein_coding |
| ENSMUSG00000032051 | 911.686653 | -0.3441276 | 0.16435702 | 0.00661378 | 0.04549394 | Down | 14148 | ferredoxin 1 [Source:MGI Symbol;Acc:MGI:103224] | Fdx1 | protein_coding |
| ENSMUSG00000032060 | 2681.19353 | -0.8884202 | 0.22820547 | 5.79E-06 | 0.00045849 | Down | 12955 | crystallin, alpha B [Source:MGI Symbol;Acc:MGI:88516] | Cryab | protein_coding |
| ENSMUSG00000032085 | 19.0279545 | -0.7171876 | 0.54649815 | 0.00622233 | 0.04364497 | Down | 21345 | transgelin [Source:MGI Symbol;Acc:MGI:106012] | Tagln | protein_coding |
| ENSMUSG00000032097 | 4311.89242 | 0.28181591 | 0.08943677 | 0.00053699 | 0.00880853 | Up | 13209 | DEAD (Asp-Glu-Ala-Asp) box polypeptide 6 [Source:MGI Symbol;Acc:MGI:104976] | Ddx6 | protein_coding |
| ENSMUSG00000032112 | 1023.72413 | -0.6575157 | 0.20452039 | 9.80E-05 | 0.00288364 | Down | 60409 | trafficking protein particle complex 4 [Source:MGI Symbol;Acc:MGI:1926211] | Trappc4 | protein_coding |
| ENSMUSG00000032119 | 348.295432 | 0.30909009 | 0.10276005 | 0.00070537 | 0.01070914 | Up | 102423 | histone H4 transcription factor [Source:MGI Symbol;Acc:MGI:2429620] | Hinfp | protein_coding |
| ENSMUSG00000032171 | 631.104367 | -0.6106403 | 0.19146025 | 0.00012491 | 0.00338386 | Down | 23988 | peptidyl-prolyl cis/trans isomerase, NIMA-interacting 1 [Source:MGI Symbol;Acc:MGI:1346036] | Pin1 | protein_coding |
| ENSMUSG00000032180 | 369.020724 | -0.5601198 | 0.14602151 | 1.31E-05 | 0.00081991 | Down | 17083 | transmembrane p24 trafficking protein 1 [Source:MGI Symbol;Acc:MGI:106201] | Tmed1 | protein_coding |
| ENSMUSG00000032217 | 743.230068 | 0.41638465 | 0.10644524 | 1.55E-05 | 0.00090777 | Up | 93836 | ring finger 111 [Source:MGI Symbol;Acc:MGI:1934919] | Rnf111 | protein_coding |
| ENSMUSG00000032228 | 1471.08439 | 0.27782386 | 0.11245642 | 0.00423747 | 0.03386017 | Up | 21406 | transcription factor 12 [Source:MGI Symbol;Acc:MGI:101877] | Tcf12 | protein_coding |
| ENSMUSG00000032231 | 16046.3637 | -0.7854461 | 0.18061863 | 9.52E-07 | 0.00014191 | Down | 12306 | annexin A2 [Source:MGI Symbol;Acc:MGI:88246] | Anxa2 | protein_coding |
| ENSMUSG00000032253 | 884.808524 | 0.33919203 | 0.14849439 | 0.00451094 | 0.03515976 | Up | 83946 | pleckstrin homology domain interacting protein [Source:MGI Symbol;Acc:MGI:1932404] | Phip | protein_coding |
| ENSMUSG00000032271 | 3503.70923 | -0.4802095 | 0.18788551 | 0.00111456 | 0.01424975 | Down | 18113 | nicotinamide N-methyltransferase [Source:MGI Symbol;Acc:MGI:1099443] | Nnmt | protein_coding |
| ENSMUSG00000032288 | 606.868875 | -0.7983628 | 0.21159052 | 1.16E-05 | 0.00075871 | Down | 102462 | IMP3, U3 small nucleolar ribonucleoprotein [Source:MGI Symbol;Acc:MGI:1916119] | Imp3 | protein_coding |
| ENSMUSG00000032293 | 1311.19589 | 0.36687749 | 0.12105491 | 0.00046146 | 0.00789467 | Up | 64602 | iron responsive element binding protein 2 [Source:MGI Symbol;Acc:MGI:1928268] | Ireb2 | protein_coding |
| ENSMUSG00000032309 | 1287.82333 | -0.2204045 | 0.08109996 | 0.00312999 | 0.02784593 | Down | 71999 | F-box protein 22 [Source:MGI Symbol;Acc:MGI:1926014] | Fbxo22 | protein_coding |
| ENSMUSG00000032312 | 1210.26954 | -0.3974109 | 0.15864328 | 0.00186831 | 0.01979822 | Down | 12988 | c-src tyrosine kinase [Source:MGI Symbol;Acc:MGI:88537] | Csk | protein_coding |
| ENSMUSG00000032315 | 11.2901366 | 0.19769553 | 0.39430734 | 0.00023556 | 0.00524931 | Up | 13076 | cytochrome P450, family 1, subfamily a, polypeptide 1 [Source:MGI Symbol;Acc:MGI:88588] | Cyp1a1 | protein_coding |
| ENSMUSG00000032316 | 853.32072 | 0.29334923 | 0.10032306 | 0.00096054 | 0.01311446 | Up | 102414 | CDC-like kinase 3 [Source:MGI Symbol;Acc:MGI:1098670] | Clk3 | protein_coding |
| ENSMUSG00000032320 | 1232.1179 | -0.3855407 | 0.11115264 | 9.46E-05 | 0.00280835 | Down | 26611 | reticulocalbin 2 [Source:MGI Symbol;Acc:MGI:1349765] | Rcn2 | protein_coding |
| ENSMUSG00000032324 | 4335.4169 | -0.2650115 | 0.09768104 | 0.0018535 | 0.01974549 | Down | 56434 | tetraspanin 3 [Source:MGI Symbol;Acc:MGI:1928098] | Tspan3 | protein_coding |
| ENSMUSG00000032332 | 268.6976 | -1.408849 | 0.43010035 | 4.16E-05 | 0.00167251 | Down | 12816 | collagen, type XII, alpha 1 [Source:MGI Symbol;Acc:MGI:88448] | Col12a1 | protein_coding |
| ENSMUSG00000032334 | 1955.64549 | -0.4865916 | 0.1603783 | 0.00027878 | 0.00579132 | Down | 16949 | lysyl oxidase-like 1 [Source:MGI Symbol;Acc:MGI:106096] | Loxl1 | protein_coding |
| ENSMUSG00000032353 | 1298.1608 | -0.5383165 | 0.14938771 | 3.31E-05 | 0.00147348 | Down | 66111 | transmembrane p24 trafficking protein 3 [Source:MGI Symbol;Acc:MGI:1913361] | Tmed3 | protein_coding |
| ENSMUSG00000032366 | 1738.72062 | -0.397456 | 0.11608797 | 8.85E-05 | 0.00268763 | Down | 22003 | tropomyosin 1, alpha [Source:MGI Symbol;Acc:MGI:98809] | Tpm1 | protein_coding |
| ENSMUSG00000032373 | 32.2640246 | -1.9979027 | 0.74501341 | 0.00022732 | 0.00510059 | Down | 76459 | carbonic anhydrase 12 [Source:MGI Symbol;Acc:MGI:1923709] | Car12 | protein_coding |
| ENSMUSG00000032377 | 648.774711 | 0.28631252 | 0.11462813 | 0.00367067 | 0.03084754 | Up | 235527 | phospholipid scramblase 4 [Source:MGI Symbol;Acc:MGI:2143267] | Plscr4 | protein_coding |
| ENSMUSG00000032401 | 948.378795 | -0.9530888 | 0.37877048 | 0.00048448 | 0.00819824 | Down | 235435 | lactase-like [Source:MGI Symbol;Acc:MGI:2183549] | Lctl | protein_coding |
| ENSMUSG00000032407 | 1082.6473 | 0.37798089 | 0.11549414 | 0.00020262 | 0.0047067 | Up | 67958 | U2 snRNP-associated SURP domain containing [Source:MGI Symbol;Acc:MGI:1915208] | U2surp | protein_coding |
| ENSMUSG00000032413 | 949.656957 | 0.41391301 | 0.15234435 | 0.0009868 | 0.01327032 | Up | 114713 | RAS p21 protein activator 2 [Source:MGI Symbol;Acc:MGI:2149960] | Rasa2 | protein_coding |
| ENSMUSG00000032418 | 19071.9235 | 0.97251222 | 0.28040722 | 2.84E-05 | 0.00136194 | Up | 17436 | malic enzyme 1, NADP(+)-dependent, cytosolic [Source:MGI Symbol;Acc:MGI:97043] | Me1 | protein_coding |
| ENSMUSG00000032423 | 2300.64182 | 0.29719267 | 0.09730617 | 0.0006607 | 0.01013548 | Up | 56403 | synaptotagmin binding, cytoplasmic RNA interacting protein [Source:MGI Symbol;Acc:MGI:1891690] | Syncrip | protein_coding |
| ENSMUSG00000032431 | 1868.10613 | -0.4546155 | 0.08677156 | 2.32E-08 | 9.39E-06 | Down | 56693 | cartilage associated protein [Source:MGI Symbol;Acc:MGI:1891221] | Crtap | protein_coding |
| ENSMUSG00000032449 | 1486.5013 | 0.30583904 | 0.08982655 | 0.00018849 | 0.00452448 | Up | 192287 | solute carrier family 25, member 36 [Source:MGI Symbol;Acc:MGI:1924909] | Slc25a36 | protein_coding |
| ENSMUSG00000032462 | 1566.26305 | 0.41901949 | 0.19151175 | 0.00363136 | 0.03059721 | Up | 74769 | phosphatidylinositol-4,5-bisphosphate 3-kinase catalytic subunit beta [Source:MGI Symbol;Acc:MGI:1922019] | Pik3cb | protein_coding |
| ENSMUSG00000032485 | 907.915542 | -0.2148611 | 0.0841898 | 0.00501871 | 0.0379028 | Down | 235623 | SREBF chaperone [Source:MGI Symbol;Acc:MGI:2135958] | Scap | protein_coding |

| | | | | | | | | | | |
|---|---|---|---|---|---|---|---|---|---|---|
| ENSMUSG00000032492 | 194.066653 | 0.71355304 | 0.37467428 | 0.00269776 | 0.02527404 | Up | 19228 | parathyroid hormone 1 receptor [Source:MGI Symbol;Acc:MGI:97801] | Pth1r | protein_coding |
| ENSMUSG00000032500 | 133.790879 | -0.7673496 | 0.57543272 | 0.0054249 | 0.03989477 | Down | 245038 | doublecortin-like kinase 3 [Source:MGI Symbol;Acc:MGI:3039580] | Dclk3 | protein_coding |
| ENSMUSG00000032519 | 346.304221 | -0.2749253 | 0.10394484 | 0.00262192 | 0.02475068 | Down | 208638 | solute carrier family 25, member 38 [Source:MGI Symbol;Acc:MGI:2384782] | Slc25a38 | protein_coding |
| ENSMUSG00000032526 | 269.014612 | -0.4038211 | 0.16497085 | 0.00205716 | 0.02117778 | Down | 26901 | SS18, nBAF chromatin remodeling complex subunit like 2 [Source:MGI Symbol;Acc:MGI:1349474] | Ss18l2 | protein_coding |
| ENSMUSG00000032553 | 499.556479 | -0.2720693 | 0.11369848 | 0.00525854 | 0.03907821 | Down | 20818 | signal recognition particle receptor, B subunit [Source:MGI Symbol;Acc:MGI:102964] | Srprb | protein_coding |
| ENSMUSG00000032562 | 6688.84436 | -0.3700257 | 0.09146118 | 1.48E-05 | 0.00089149 | Down | 14678 | guanine nucleotide binding protein (G protein), alpha inhibiting 2 [Source:MGI Symbol;Acc:MGI:95772] | Gnai2 | protein_coding |
| ENSMUSG00000032575 | 701.807022 | -0.55279 | 0.23888185 | 0.00180589 | 0.01941545 | Down | 74840 | mesencephalic astrocyte-derived neurotrophic factor [Source:MGI Symbol;Acc:MGI:1922090] | Manf | protein_coding |
| ENSMUSG00000032580 | 1962.56922 | 0.39205208 | 0.11959354 | 0.00018388 | 0.00444761 | Up | 83486 | RNA binding motif protein 5 [Source:MGI Symbol;Acc:MGI:1933204] | Rbm5 | protein_coding |
| ENSMUSG00000032621 | 997.53878 | 0.31012748 | 0.13097079 | 0.00424526 | 0.03388677 | Up | 218543 | splicing regulatory glutamine/lysine-rich protein 1 [Source:MGI Symbol;Acc:MGI:2145245] | Srek1 | protein_coding |
| ENSMUSG00000032625 | 343.122056 | 0.43243741 | 0.15526605 | 0.00074126 | 0.01102724 | Up | 330267 | thrombospondin, type I, domain containing 7A [Source:MGI Symbol;Acc:MGI:2685683] | Thsd7a | protein_coding |
| ENSMUSG00000032656 | 169.204023 | -0.3532661 | 0.1539989 | 0.00401535 | 0.03268583 | Down | 320253 | membrane associated ring-CH-type finger 3 [Source:MGI Symbol;Acc:MGI:2443667] | Marchf3 | protein_coding |
| ENSMUSG00000032667 | 2031.00601 | -0.3053254 | 0.13135804 | 0.00534509 | 0.03950454 | Down | 330260 | paraoxonase 2 [Source:MGI Symbol;Acc:MGI:106687] | Pon2 | protein_coding |
| ENSMUSG00000032679 | 3278.32338 | -0.6107512 | 0.16548676 | 1.98E-05 | 0.0010735 | Down | 12509 | CD59a antigen [Source:MGI Symbol;Acc:MGI:109177] | Cd59a | protein_coding |
| ENSMUSG00000032715 | 147.065519 | -0.8721046 | 0.29265228 | 0.00016784 | 0.00418819 | Down | 228775 | tribbles pseudokinase 3 [Source:MGI Symbol;Acc:MGI:1345675] | Trib3 | protein_coding |
| ENSMUSG00000032727 | 406.070624 | 0.39929461 | 0.13173861 | 0.0003859 | 0.00714005 | Up | 218613 | MIER family member 3 [Source:MGI Symbol;Acc:MGI:2442317] | Mier3 | protein_coding |
| ENSMUSG00000032763 | 562.806141 | -0.4037788 | 0.12108271 | 0.00013609 | 0.00361827 | Down | 216136 | ilvB (bacterial acetolactate synthase)-like [Source:MGI Symbol;Acc:MGI:1351911] | Ilvbl | protein_coding |
| ENSMUSG00000032766 | 348.909106 | -0.4392636 | 0.21688177 | 0.00469724 | 0.03618142 | Down | 66066 | guanine nucleotide binding protein (G protein), gamma 11 [Source:MGI Symbol;Acc:MGI:1913316] | Gng11 | protein_coding |
| ENSMUSG00000032845 | 14.7512502 | -0.2191467 | 0.47780201 | 3.85E-05 | 0.00161379 | Down | 225638 | alpha-kinase 2 [Source:MGI Symbol;Acc:MGI:2449492] | Alpk2 | protein_coding |
| ENSMUSG00000032849 | 289.815697 | 0.38063177 | 0.14769411 | 0.00169049 | 0.01859315 | Up | 239273 | ATP-binding cassette, sub-family C (CFTR/MRP), member 4 [Source:MGI Symbol;Acc:MGI:2443111] | Abcc4 | protein_coding |
| ENSMUSG00000032959 | 2520.40611 | -0.5820254 | 0.17256643 | 6.86E-05 | 0.00226099 | Down | 23980 | phosphatidylethanolamine binding protein 1 [Source:MGI Symbol;Acc:MGI:1344408] | Pebp1 | protein_coding |
| ENSMUSG00000032966 | 6339.32386 | -0.2578583 | 0.11019041 | 0.0062138 | 0.04360395 | Down | 14225 | FK506 binding protein 1a [Source:MGI Symbol;Acc:MGI:95541] | Fkbp1a | protein_coding |
| ENSMUSG00000032977 | 278.164333 | -0.3590583 | 0.1555723 | 0.0030355 | 0.02724405 | Down | 108707 | family with sequence similarity 207, member A [Source:MGI Symbol;Acc:MGI:1916334] | Fam207a | protein_coding |
| ENSMUSG00000032997 | 1223.66847 | -0.7167733 | 0.1573048 | 4.16E-07 | 7.68E-05 | Down | 74241 | chondroitin polymerizing factor [Source:MGI Symbol;Acc:MGI:106576] | Chpf | protein_coding |
| ENSMUSG00000032998 | 821.036451 | 0.34750274 | 0.13223829 | 0.00178467 | 0.01925102 | Up | 230700 | forkhead box J3 [Source:MGI Symbol;Acc:MGI:2443432] | Foxj3 | protein_coding |
| ENSMUSG00000033020 | 446.276563 | -0.6922394 | 0.15908193 | 1.14E-06 | 0.00016035 | Down | 69833 | polymerase (RNA) II (DNA directed) polypeptide F [Source:MGI Symbol;Acc:MGI:1349393] | Polr2f | protein_coding |
| ENSMUSG00000033021 | 781.765934 | -0.2398872 | 0.0865885 | 0.00215418 | 0.02185892 | Down | 69080 | GDP-mannose pyrophosphorylase A [Source:MGI Symbol;Acc:MGI:1916330] | Gmppa | protein_coding |
| ENSMUSG00000033054 | 276.952926 | 0.42335667 | 0.15651505 | 0.00096447 | 0.01311446 | Up | 244879 | nuclear protein in the AT region [Source:MGI Symbol;Acc:MGI:107605] | Npat | protein_coding |
| ENSMUSG00000033075 | 473.539056 | 0.40774312 | 0.12145919 | 0.00013236 | 0.00354809 | Up | 223870 | SUMO1/sentrin specific peptidase 1 [Source:MGI Symbol;Acc:MGI:2445054] | Senp1 | protein_coding |
| ENSMUSG00000033082 | 100.511032 | 0.58185666 | 0.21772812 | 0.00062688 | 0.00977314 | Up | 243653 | C-type lectin domain family 1, member a [Source:MGI Symbol;Acc:MGI:2444151] | Clec1a | protein_coding |
| ENSMUSG00000033099 | 355.755713 | -0.5815427 | 0.17228887 | 6.93E-05 | 0.00227458 | Down | 97961 | nucleolar protein 12 [Source:MGI Symbol;Acc:MGI:2146285] | Nol12 | protein_coding |
| ENSMUSG00000033152 | 151.67431 | -0.7199202 | 0.25836604 | 0.00033408 | 0.00656251 | Down | 319655 | podocalyxin-like 2 [Source:MGI Symbol;Acc:MGI:2442488] | Podxl2 | protein_coding |
| ENSMUSG00000033166 | 281.443547 | 0.37904738 | 0.1407685 | 0.00118184 | 0.01481407 | Up | 72662 | DIS3 homolog, exosome endoribonuclease and 3'-5' exoribonuclease [Source:MGI Symbol;Acc:MGI:1919912] | Dis3 | protein_coding |
| ENSMUSG00000033208 | 39.2904934 | -1.780146 | 0.48227759 | 9.68E-06 | 0.00068061 | Down | 20203 | S100 protein, beta polypeptide, neural [Source:MGI Symbol;Acc:MGI:98217] | S100b | protein_coding |
| ENSMUSG00000033256 | 117.52104 | -0.5485344 | 0.26154925 | 0.00274008 | 0.02555255 | Down | 435684 | Src homology 2 domain containing F [Source:MGI Symbol;Acc:MGI:3613669] | Shf | protein_coding |
| ENSMUSG00000033257 | 449.716328 | 0.39586365 | 0.19394228 | 0.00566966 | 0.04095318 | Up | 67534 | tubulin tyrosine ligase-like family, member 4 [Source:MGI Symbol;Acc:MGI:1914784] | Ttll4 | protein_coding |
| ENSMUSG00000033307 | 701.612928 | -0.6100091 | 0.20867223 | 0.0003005 | 0.00611735 | Down | 17319 | macrophage migration inhibitory factor (glycosylation-inhibiting factor) [Source:MGI Symbol;Acc:MGI:96982] | Mif | protein_coding |
| ENSMUSG00000033313 | 118.7882 | -0.7888363 | 0.1914123 | 2.59E-06 | 0.00027895 | Down | 50788 | F-box and leucine-rich repeat protein 8 [Source:MGI Symbol;Acc:MGI:1354697] | Fbxl8 | protein_coding |
| ENSMUSG00000033319 | 559.353763 | 0.30370667 | 0.12278643 | 0.00343137 | 0.02943118 | Up | 240263 | fem 1 homolog c [Source:MGI Symbol;Acc:MGI:2444737] | Fem1c | protein_coding |
| ENSMUSG00000033352 | 854.038506 | 0.2492132 | 0.0982716 | 0.00405613 | 0.03286198 | Up | 26398 | mitogen-activated protein kinase kinase 4 [Source:MGI Symbol;Acc:MGI:1346869] | Map2k4 | protein_coding |
| ENSMUSG00000033377 | 3842.86592 | -0.3881229 | 0.13048655 | 0.00049915 | 0.00837668 | Down | 114301 | palmdelphin [Source:MGI Symbol;Acc:MGI:2148896] | Palmd | protein_coding |
| ENSMUSG00000033379 | 1499.87812 | -0.3771781 | 0.16814398 | 0.00391428 | 0.03216375 | Down | 114143 | ATPase, H+ transporting, lysosomal V0 subunit B [Source:MGI Symbol;Acc:MGI:1890510] | Atp6v0b | protein_coding |
| ENSMUSG00000033400 | 1183.18618 | 0.35217808 | 0.11670994 | 0.0005302 | 0.00877232 | Up | 77559 | amylo-1,6-glucosidase, 4-alpha-glucanotransferase [Source:MGI Symbol;Acc:MGI:1924809] | Agl | protein_coding |
| ENSMUSG00000033411 | 372.915082 | 0.39187445 | 0.16189079 | 0.0023694 | 0.02320131 | Up | 329506 | CTD (carboxy-terminal domain, RNA polymerase II, polypeptide A) small phosphatase like 2 [Source:MGI Symbol;Acc:MGI:1196405] | Ctdspl2 | protein_coding |
| ENSMUSG00000033416 | 854.934709 | 0.4013619 | 0.17610823 | 0.00329777 | 0.02878681 | Up | 68778 | guanylyl cyclase domain containing 1 [Source:MGI Symbol;Acc:MGI:1916028] | Gucd1 | protein_coding |
| ENSMUSG00000033423 | 936.18849 | -0.2382361 | 0.06732482 | 0.00016699 | 0.00417349 | Down | 140546 | exoribonuclease 3 [Source:MGI Symbol;Acc:MGI:2153887] | Eri3 | protein_coding |
| ENSMUSG00000033429 | 395.316598 | -0.4577332 | 0.18104589 | 0.00146141 | 0.01698185 | Down | 73724 | methylmalonyl CoA epimerase [Source:MGI Symbol;Acc:MGI:1920974] | Mcee | protein_coding |
| ENSMUSG00000033439 | 116.356993 | 0.39718077 | 0.19866308 | 0.00627685 | 0.04393255 | Up | 229780 | tRNA methyltransferase 13 [Source:MGI Symbol;Acc:MGI:1925219] | Trmt13 | protein_coding |
| ENSMUSG00000033460 | 240.746169 | -0.3631562 | 0.13082181 | 0.00107036 | 0.01388819 | Down | 78248 | armadillo repeat containing, X-linked 1 [Source:MGI Symbol;Acc:MGI:1925498] | Armcx1 | protein_coding |
| ENSMUSG00000033467 | 577.348242 | -1.0350919 | 0.25009093 | 2.07E-06 | 0.00023819 | Down | 57914 | cytokine receptor-like factor 2 [Source:MGI Symbol;Acc:MGI:1889506] | Crlf2 | protein_coding |
| ENSMUSG00000033488 | 777.178243 | -0.376549 | 0.14487238 | 0.0015936 | 0.01786615 | Down | 226527 | crystallin zeta like 2 [Source:MGI Symbol;Acc:MGI:2448516] | Cryzl2 | protein_coding |
| ENSMUSG00000033585 | 713.124545 | -0.8081017 | 0.18719419 | 1.02E-06 | 0.00014754 | Down | 17984 | necdin, MAGE family member [Source:MGI Symbol;Acc:MGI:97290] | Ndn | protein_coding |
| ENSMUSG00000033589 | 311.336311 | -0.5109433 | 0.16814519 | 0.00024194 | 0.0053257 | Down | 72549 | receptor accessory protein 4 [Source:MGI Symbol;Acc:MGI:1919799] | Reep4 | protein_coding |
| ENSMUSG00000033596 | 592.406839 | 0.2051179 | 0.07751613 | 0.00391537 | 0.03216375 | Up | 234736 | ring finger and WD repeat domain 3 [Source:MGI Symbol;Acc:MGI:2384584] | Rfwd3 | protein_coding |

| | | | | | | | | | | |
|---|---|---|---|---|---|---|---|---|---|---|
| ENSMUSG00000033632 | 195.880411 | 0.43026253 | 0.16740319 | 0.00137318 | 0.0163783 | Up | 225289 | expressed sequence AW554918 [Source:MGI Symbol;Acc:MGI:2147376] | AW554918 | protein_coding |
| ENSMUSG00000033685 | 4125.43469 | -0.2392812 | 0.08483053 | 0.00204097 | 0.02107786 | Down | 22228 | uncoupling protein 2 (mitochondrial, proton carrier) [Source:MGI Symbol;Acc:MGI:109354] | Ucp2 | protein_coding |
| ENSMUSG00000033701 | 603.284727 | -0.6458932 | 0.22875902 | 0.00034112 | 0.00664443 | Down | 72482 | acyl-Coenzyme A binding domain containing 6 [Source:MGI Symbol;Acc:MGI:1919732] | Acbd6 | protein_coding |
| ENSMUSG00000033713 | 1185.41801 | 0.42521499 | 0.12058665 | 6.76E-05 | 0.00225926 | Up | 71375 | forkhead box N3 [Source:MGI Symbol;Acc:MGI:1918625] | Foxn3 | protein_coding |
| ENSMUSG00000033735 | 906.1373 | -0.643993 | 0.14708309 | 8.91E-07 | 0.0001353 | Down | 20751 | sepiapterin reductase [Source:MGI Symbol;Acc:MGI:103078] | Spr | protein_coding |
| ENSMUSG00000033751 | 336.876278 | -0.6088091 | 0.2680715 | 0.00153899 | 0.01748314 | Down | 102060 | growth arrest and DNA-damage-inducible, gamma interacting protein 1 [Source:MGI Symbol;Acc:MGI:1914947] | Gadd45gip1 | protein_coding |
| ENSMUSG00000033760 | 311.506252 | 0.47234988 | 0.14406903 | 0.00013806 | 0.00364704 | Up | 66704 | RNA binding motif protein 4B [Source:MGI Symbol;Acc:MGI:1913954] | Rbm4b | protein_coding |
| ENSMUSG00000033767 | 329.463255 | 0.284906 | 0.09752256 | 0.00105817 | 0.01378505 | Up | 229473 | transmembrane 131 like [Source:MGI Symbol;Acc:MGI:2443399] | Tmem131l | protein_coding |
| ENSMUSG00000033768 | 95.8273839 | -0.5835178 | 0.36613353 | 0.00586591 | 0.04181297 | Down | 18190 | neurexin II [Source:MGI Symbol;Acc:MGI:1096362] | Nrxn2 | protein_coding |
| ENSMUSG00000033808 | 600.219601 | 0.35107836 | 0.08699128 | 1.23E-05 | 0.00078933 | Up | 211499 | transmembrane protein 87A [Source:MGI Symbol;Acc:MGI:2441844] | Tmem87a | protein_coding |
| ENSMUSG00000033819 | 822.211038 | -0.3838297 | 0.10793601 | 6.76E-05 | 0.00225926 | Down | 73062 | protein phosphatase 1, regulatory subunit 16A [Source:MGI Symbol;Acc:MGI:1920312] | Ppp1r16a | protein_coding |
| ENSMUSG00000033845 | 413.30982 | -0.3527516 | 0.16428053 | 0.0056525 | 0.0408655 | Down | 27395 | mitochondrial ribosomal protein L15 [Source:MGI Symbol;Acc:MGI:1351639] | Mrpl15 | protein_coding |
| ENSMUSG00000033871 | 91.6837807 | 1.30380747 | 0.27653299 | 1.28E-07 | 3.53E-05 | Up | 170826 | peroxisome proliferative activated receptor, gamma, coactivator 1 beta [Source:MGI Symbol;Acc:MGI:2444934] | Ppargc1b | protein_coding |
| ENSMUSG00000033883 | 297.892919 | 0.3592822 | 0.14853432 | 0.00287733 | 0.02637436 | Up | 241944 | zinc finger protein 267 [Source:MGI Symbol;Acc:MGI:1098769] | Zfp267 | protein_coding |
| ENSMUSG00000033909 | 569.537167 | 0.35366115 | 0.14428667 | 0.00272631 | 0.02545336 | Up | 72344 | ubiquitin specific peptidase 36 [Source:MGI Symbol;Acc:MGI:1919594] | Usp36 | protein_coding |
| ENSMUSG00000033916 | 1604.60802 | -0.4326508 | 0.16787778 | 0.00134343 | 0.01614199 | Down | 68953 | charged multivesicular body protein 2A [Source:MGI Symbol;Acc:MGI:1916203] | Chmp2a | protein_coding |
| ENSMUSG00000033938 | 1173.795 | -0.5056843 | 0.21312639 | 0.00166033 | 0.01836086 | Down | 66916 | NADH:ubiquinone oxidoreductase subunit B7 [Source:MGI Symbol;Acc:MGI:1914166] | Ndufb7 | protein_coding |
| ENSMUSG00000033940 | 1812.45504 | -0.4862346 | 0.1759881 | 0.00062943 | 0.00979801 | Down | 101314 | BRICK1, SCAR/WAVE actin-nucleating complex subunit [Source:MGI Symbol;Acc:MGI:1915406] | Brk1 | protein_coding |
| ENSMUSG00000033972 | 154.269595 | 0.41195442 | 0.16420044 | 0.00173874 | 0.01890613 | Up | 319615 | zinc finger protein 944 [Source:MGI Symbol;Acc:MGI:2442394] | Zfp944 | protein_coding |
| ENSMUSG00000033998 | 46.2411027 | 0.03808558 | 0.20360175 | 0.00192468 | 0.02026339 | Up | 16525 | potassium channel, subfamily K, member 1 [Source:MGI Symbol;Acc:MGI:109322] | Kcnk1 | protein_coding |
| ENSMUSG00000034006 | 239.143805 | -0.4863243 | 0.20687389 | 0.00202996 | 0.02101764 | Down | 66943 | solute carrier family 66 member 2 [Source:MGI Symbol;Acc:MGI:1914193] | Slc66a2 | protein_coding |
| ENSMUSG00000034021 | 704.535631 | 0.35865135 | 0.13065252 | 0.00131701 | 0.01588327 | Up | 100710 | PDS5 cohesin associated factor B [Source:MGI Symbol;Acc:MGI:2140945] | Pds5b | protein_coding |
| ENSMUSG00000034042 | 904.110974 | 0.39393414 | 0.0942306 | 5.54E-06 | 0.00044782 | Up | 77110 | GC-rich promoter binding protein 1-like 1 [Source:MGI Symbol;Acc:MGI:1924360] | Gpbp1l1 | protein_coding |
| ENSMUSG00000034108 | 366.643907 | -0.6296567 | 0.2089165 | 0.00020946 | 0.00478577 | Down | 12460 | copper chaperone for superoxide dismutase [Source:MGI Symbol;Acc:MGI:1333783] | Ccs | protein_coding |
| ENSMUSG00000034157 | 587.399404 | 0.46460321 | 0.11512526 | 8.09E-06 | 0.00059029 | Up | 217732 | CLOCK interacting protein, circadian [Source:MGI Symbol;Acc:MGI:1919185] | Cipc | protein_coding |
| ENSMUSG00000034158 | 4759.04697 | 0.39048638 | 0.11900141 | 0.00018725 | 0.00451991 | Up | 320184 | leucine rich repeat containing 58 [Source:MGI Symbol;Acc:MGI:2443542] | Lrrc58 | protein_coding |
| ENSMUSG00000034165 | 2277.542 | -0.2419332 | 0.10691973 | 0.00737991 | 0.04917416 | Down | 12445 | cyclin D3 [Source:MGI Symbol;Acc:MGI:88315] | Ccnd3 | protein_coding |
| ENSMUSG00000034201 | 1380.93298 | -0.4834856 | 0.13407913 | 3.74E-05 | 0.00158342 | Down | 78926 | growth arrest-specific 2 like 1 [Source:MGI Symbol;Acc:MGI:1926176] | Gas2l1 | protein_coding |
| ENSMUSG00000034220 | 1061.41748 | -0.6316962 | 0.36292115 | 0.00388352 | 0.03200803 | Down | 14733 | glypican 1 [Source:MGI Symbol;Acc:MGI:1194891] | Gpc1 | protein_coding |
| ENSMUSG00000034235 | 338.202682 | 0.445454 | 0.25058676 | 0.00706413 | 0.04775566 | Up | 78787 | ubiquitin specific peptidase 54 [Source:MGI Symbol;Acc:MGI:1926037] | Usp54 | protein_coding |
| ENSMUSG00000034259 | 297.254298 | -0.4185549 | 0.1640207 | 0.00149384 | 0.01723537 | Down | 109075 | exosome component 4 [Source:MGI Symbol;Acc:MGI:1923576] | Exosc4 | protein_coding |
| ENSMUSG00000034269 | 1704.94405 | 0.35591849 | 0.15467652 | 0.00394518 | 0.03232446 | Up | 72895 | SET domain containing 5 [Source:MGI Symbol;Acc:MGI:1920145] | Setd5 | protein_coding |
| ENSMUSG00000034297 | 1266.3702 | 0.50655334 | 0.15823504 | 0.00015825 | 0.00404202 | Up | 327987 | mediator complex subunit 13 [Source:MGI Symbol;Acc:MGI:3029632] | Med13 | protein_coding |
| ENSMUSG00000034345 | 1106.58093 | -0.4376413 | 0.16664895 | 0.00112519 | 0.01433253 | Down | 66467 | general transcription factor IIH, polypeptide 5 [Source:MGI Symbol;Acc:MGI:107227] | Gtf2h5 | protein_coding |
| ENSMUSG00000034353 | 72.5015249 | -0.5605852 | 0.35364197 | 0.00618948 | 0.04350849 | Down | 51801 | receptor (calcitonin) activity modifying protein 1 [Source:MGI Symbol;Acc:MGI:1858418] | Ramp1 | protein_coding |
| ENSMUSG00000034412 | 355.646193 | -0.3160299 | 0.14048918 | 0.00560277 | 0.04066318 | Down | 103724 | TBC1 domain family, member 10a [Source:MGI Symbol;Acc:MGI:2144164] | Tbc1d10a | protein_coding |
| ENSMUSG00000034427 | 56.6038613 | 0.03966505 | 0.20399462 | 0.00444769 | 0.0348257 | Up | 217328 | myosin XVB [Source:MGI Symbol;Acc:MGI:2685534] | Myo15b | protein_coding |
| ENSMUSG00000034480 | 735.682039 | 0.45384253 | 0.20345503 | 0.00296881 | 0.02686817 | Up | 54004 | diaphanous related formin 2 [Source:MGI Symbol;Acc:MGI:1858500] | Diaph2 | protein_coding |
| ENSMUSG00000034501 | 372.626103 | -0.2351547 | 0.09680256 | 0.0060262 | 0.0426189 | Down | 67708 | pecanex homolog 4 [Source:MGI Symbol;Acc:MGI:1914958] | Pcnx4 | protein_coding |
| ENSMUSG00000034543 | 863.132935 | 0.36347688 | 0.12928199 | 0.00096552 | 0.01311446 | Up | 74522 | microrchidia 2A [Source:MGI Symbol;Acc:MGI:1921772] | Morc2a | protein_coding |
| ENSMUSG00000034557 | 564.096524 | 0.43226871 | 0.13311567 | 0.00018179 | 0.00442085 | Up | 230597 | zinc finger, FYVE domain containing 9 [Source:MGI Symbol;Acc:MGI:2652838] | Zfyve9 | protein_coding |
| ENSMUSG00000034610 | 483.095084 | 0.3814921 | 0.15460096 | 0.00220602 | 0.02213517 | Up | 230594 | terminal uridylyl transferase 4 [Source:MGI Symbol;Acc:MGI:2445126] | Tut4 | protein_coding |
| ENSMUSG00000034621 | 927.650428 | 0.36050001 | 0.16047225 | 0.00442055 | 0.03470851 | Up | 237943 | G patch domain containing 8 [Source:MGI Symbol;Acc:MGI:1918667] | Gpatch8 | protein_coding |
| ENSMUSG00000034640 | 1394.1125 | 0.33457965 | 0.15749846 | 0.0067296 | 0.04606923 | Up | 99929 | TCDD-inducible poly(ADP-ribose) polymerase [Source:MGI Symbol;Acc:MGI:2159210] | Tiparp | protein_coding |
| ENSMUSG00000034647 | 646.979853 | 0.39869655 | 0.18732516 | 0.0046326 | 0.0359007 | Up | 106585 | ankyrin repeat domain 12 [Source:MGI Symbol;Acc:MGI:1914357] | Ankrd12 | protein_coding |
| ENSMUSG00000034659 | 3415.29306 | -0.4129099 | 0.12899981 | 0.00020188 | 0.00469853 | Down | 68539 | transmembrane protein 109 [Source:MGI Symbol;Acc:MGI:1915789] | Tmem109 | protein_coding |
| ENSMUSG00000034667 | 1346.12953 | 0.17076944 | 0.06750978 | 0.00667552 | 0.04577618 | Up | 73192 | exportin, tRNA (nuclear export receptor for tRNAs) [Source:MGI Symbol;Acc:MGI:1920442] | Xpot | protein_coding |
| ENSMUSG00000034675 | 970.391558 | -0.4919215 | 0.16745091 | 0.00038129 | 0.0071115 | Down | 56320 | drebrin 1 [Source:MGI Symbol;Acc:MGI:1931838] | Dbn1 | protein_coding |
| ENSMUSG00000034685 | 137.617978 | -1.0226639 | 0.18742494 | 3.14E-09 | 3.04E-06 | Down | 217219 | family with sequence similarity 171, member A2 [Source:MGI Symbol;Acc:MGI:2448496] | Fam171a2 | protein_coding |
| ENSMUSG00000034724 | 770.451506 | 0.37893846 | 0.11328913 | 0.00016205 | 0.00408764 | Up | 231464 | CCR4-NOT transcription complex, subunit 6-like [Source:MGI Symbol;Acc:MGI:2443154] | Cnot6l | protein_coding |
| ENSMUSG00000034744 | 824.925704 | -0.674445 | 0.15038513 | 6.08E-07 | 0.00010294 | Down | 56174 | N-acetylglucosamine kinase [Source:MGI Symbol;Acc:MGI:1860418] | Nagk | protein_coding |
| ENSMUSG00000034761 | 642.680269 | 0.30866713 | 0.11272027 | 0.00157402 | 0.01775686 | Up | 399510 | mitogen-activated protein kinase kinase kinase kinase 5 [Source:MGI Symbol;Acc:MGI:1925503] | Map4k5 | protein_coding |
| ENSMUSG00000034780 | 29.7620123 | 0.77346869 | 0.55736119 | 0.00521098 | 0.0387958 | Up | 26877 | UDP-Gal:betaGlcNAc beta 1,3-galactosyltransferase, polypeptide 1 [Source:MGI Symbol;Acc:MGI:1349403] | B3galt1 | protein_coding |

| | | | | | | | | | | |
|---|---|---|---|---|---|---|---|---|---|---|
| ENSMUSG00000034786 | 183.178201 | -0.6603214 | 0.24020738 | 0.00041314 | 0.00740784 | Down | 106512 | G-protein signalling modulator 3 (AGS3-like, C. elegans) [Source:MGI Symbol;Acc:MGI:2146785] | Gpsm3 | protein_coding |
| ENSMUSG00000034793 | 948.679748 | -0.2985758 | 0.10132641 | 0.00088525 | 0.01238725 | Down | 68401 | glucose 6 phosphatase, catalytic, 3 [Source:MGI Symbol;Acc:MGI:1915651] | G6pc3 | protein_coding |
| ENSMUSG00000034801 | 898.906033 | 0.46614149 | 0.10317205 | 8.99E-07 | 0.0001353 | Up | 20663 | SOS Ras/Rho guanine nucleotide exchange factor 2 [Source:MGI Symbol;Acc:MGI:98355] | Sos2 | protein_coding |
| ENSMUSG00000034807 | 1945.53479 | -0.2282409 | 0.096461 | 0.00748289 | 0.04964493 | Down | 234407 | collagen beta(1-O)galactosyltransferase 1 [Source:MGI Symbol;Acc:MGI:1924348] | Colgalt1 | protein_coding |
| ENSMUSG00000034820 | 868.434506 | 0.24881424 | 0.09247468 | 0.0025577 | 0.02435899 | Up | 269061 | cleavage and polyadenylation specific factor 7 [Source:MGI Symbol;Acc:MGI:1917826] | Cpsf7 | protein_coding |
| ENSMUSG00000034868 | 74.9811648 | -0.4241754 | 0.20031868 | 0.0041759 | 0.03354971 | Down | 67938 | myosin, light chain 12B, regulatory [Source:MGI Symbol;Acc:MGI:107494] | Myl12b | protein_coding |
| ENSMUSG00000034880 | 585.632514 | -0.5541958 | 0.20314427 | 0.00053786 | 0.00880853 | Down | 94065 | mitochondrial ribosomal protein L34 [Source:MGI Symbol;Acc:MGI:2137227] | Mrpl34 | protein_coding |
| ENSMUSG00000034893 | 631.611972 | 0.26013711 | 0.0758784 | 0.00022609 | 0.00508701 | Up | 338337 | component of oligomeric golgi complex 3 [Source:MGI Symbol;Acc:MGI:2450151] | Cog3 | protein_coding |
| ENSMUSG00000034932 | 501.113644 | -0.869803 | 0.23993694 | 1.71E-05 | 0.00097401 | Down | 66047 | mitochondrial ribosomal protein L54 [Source:MGI Symbol;Acc:MGI:1913297] | Mrpl54 | protein_coding |
| ENSMUSG00000034974 | 750.772989 | -0.7865971 | 0.16210258 | 8.60E-08 | 2.54E-05 | Down | 13144 | death-associated protein kinase 3 [Source:MGI Symbol;Acc:MGI:1203520] | Dapk3 | protein_coding |
| ENSMUSG00000034981 | 581.097604 | 1.21184339 | 0.33459183 | 1.35E-05 | 0.00083781 | Up | 231440 | prostate androgen-regulated mucin-like protein 1 [Source:MGI Symbol;Acc:MGI:2443349] | Parm1 | protein_coding |
| ENSMUSG00000034987 | 52.4193209 | 0.69608237 | 0.32760296 | 0.00183888 | 0.01964012 | Up | 15466 | histamine receptor H2 [Source:MGI Symbol;Acc:MGI:108482] | Hrh2 | protein_coding |
| ENSMUSG00000034993 | 2433.84377 | -0.3046122 | 0.10005451 | 0.00068835 | 0.01048989 | Down | 26949 | vesicle amine transport 1 [Source:MGI Symbol;Acc:MGI:1349450] | Vat1 | protein_coding |
| ENSMUSG00000035027 | 1600.43385 | -0.3550591 | 0.14848073 | 0.0030102 | 0.02708091 | Down | 26396 | mitogen-activated protein kinase kinase 2 [Source:MGI Symbol;Acc:MGI:1346867] | Map2k2 | protein_coding |
| ENSMUSG00000035085 | 251.016578 | -0.4660033 | 0.23848079 | 0.00483431 | 0.03700914 | Down | 66330 | RIKEN cDNA 1700020L24 gene [Source:MGI Symbol;Acc:MGI:1913580] | 1700020L24Rik | protein_coding |
| ENSMUSG00000035139 | 380.573405 | 0.29619 | 0.08899768 | 0.00025068 | 0.00545876 | Up | 75420 | SECIS binding protein 2 [Source:MGI Symbol;Acc:MGI:1922670] | Secisbp2 | protein_coding |
| ENSMUSG00000035142 | 218.713601 | -0.399747 | 0.17849849 | 0.00353733 | 0.03009197 | Down | 76826 | nucleotide binding protein-like [Source:MGI Symbol;Acc:MGI:1924076] | Nubpl | protein_coding |
| ENSMUSG00000035150 | 1181.85353 | 0.23738445 | 0.09978257 | 0.00671726 | 0.04600416 | Up | 26905 | eukaryotic translation initiation factor 2, subunit 3, structural gene X-linked [Source:MGI Symbol;Acc:MGI:1349431] | Eif2s3x | protein_coding |
| ENSMUSG00000035158 | 350.927586 | 0.40285241 | 0.18756205 | 0.00435616 | 0.03446687 | Up | 17342 | melanogenesis associated transcription factor [Source:MGI Symbol;Acc:MGI:104554] | Mitf | protein_coding |
| ENSMUSG00000035186 | 208.349103 | -3.0317593 | 0.77934432 | 3.36E-06 | 0.00033259 | Down | 24108 | ubiquitin D [Source:MGI Symbol;Acc:MGI:1344410] | Ubd | protein_coding |
| ENSMUSG00000035199 | 1405.73041 | -0.3757732 | 0.13219863 | 0.00076284 | 0.01124169 | Down | 65106 | ADP-ribosylation factor-like 6 interacting protein 5 [Source:MGI Symbol;Acc:MGI:1929501] | Arl6ip5 | protein_coding |
| ENSMUSG00000035212 | 1183.26505 | -0.3098365 | 0.10224707 | 0.00061725 | 0.00966009 | Down | 230514 | leptin receptor overlapping transcript [Source:MGI Symbol;Acc:MGI:2687005] | Leprot | protein_coding |
| ENSMUSG00000035234 | 183.155156 | 0.50589289 | 0.12788672 | 9.05E-06 | 0.00064754 | Up | 70681 | BRCA1 A complex subunit [Source:MGI Symbol;Acc:MGI:1917931] | Abraxas1 | protein_coding |
| ENSMUSG00000035242 | 5717.77067 | -0.59647 | 0.19588673 | 0.00019302 | 0.00456711 | Down | 18245 | ornithine decarboxylase antizyme 1 [Source:MGI Symbol;Acc:MGI:109433] | Oaz1 | protein_coding |
| ENSMUSG00000035245 | 447.998734 | 0.37623972 | 0.12620359 | 0.00053704 | 0.00880853 | Up | 101351 | EGF domain-specific O-linked N-acetylglucosamine (GlcNAc) transferase [Source:MGI Symbol;Acc:MGI:2141669] | Eogt | protein_coding |
| ENSMUSG00000035268 | 553.286782 | -0.6912379 | 0.17375786 | 5.52E-06 | 0.00044782 | Down | 18769 | protein kinase inhibitor, gamma [Source:MGI Symbol;Acc:MGI:1343086] | Pkig | protein_coding |
| ENSMUSG00000035277 | 345.43168 | -0.6030647 | 0.38879249 | 0.00599359 | 0.04253641 | Down | 11878 | aristaless related homeobox [Source:MGI Symbol;Acc:MGI:1097716] | Arx | protein_coding |
| ENSMUSG00000035278 | 432.163749 | -0.3668863 | 0.15682119 | 0.0032958 | 0.02878681 | Down | 78670 | pleckstrin homology domain containing, family J member 1 [Source:MGI Symbol;Acc:MGI:1925920] | Plekhj1 | protein_coding |
| ENSMUSG00000035283 | 153.784923 | -0.4897073 | 0.25215016 | 0.00440887 | 0.03463347 | Down | 11554 | adrenergic receptor, beta 1 [Source:MGI Symbol;Acc:MGI:87937] | Adrb1 | protein_coding |
| ENSMUSG00000035293 | 243.52068 | 0.51311007 | 0.14453665 | 4.28E-05 | 0.00169601 | Up | 217558 | G2/M-phase specific E3 ubiquitin ligase [Source:MGI Symbol;Acc:MGI:2444298] | G2e3 | protein_coding |
| ENSMUSG00000035372 | 323.461498 | -0.3174041 | 0.13318512 | 0.00406313 | 0.03287128 | Down | 72056 | RIKEN cDNA 1810055G02 gene [Source:MGI Symbol;Acc:MGI:1919306] | 1810055G02Rik | protein_coding |
| ENSMUSG00000035413 | 910.636737 | -0.387216 | 0.18386716 | 0.00484247 | 0.03705413 | Down | 103743 | transmembrane protein 98 [Source:MGI Symbol;Acc:MGI:1923457] | Tmem98 | protein_coding |
| ENSMUSG00000035437 | 1161.51263 | 0.28713413 | 0.10625567 | 0.00201314 | 0.02088341 | Up | 227800 | RAB GTPase activating protein 1 [Source:MGI Symbol;Acc:MGI:2385139] | Rabgap1 | protein_coding |
| ENSMUSG00000035478 | 1671.33707 | -0.5124975 | 0.17539016 | 0.00036166 | 0.00687161 | Down | 17192 | methyl-CpG binding domain protein 3 [Source:MGI Symbol;Acc:MGI:1333812] | Mbd3 | protein_coding |
| ENSMUSG00000035559 | 858.070705 | -0.6837661 | 0.19101069 | 2.53E-05 | 0.00124403 | Down | 234384 | MPV17 mitochondrial membrane protein-like 2 [Source:MGI Symbol;Acc:MGI:2681846] | Mpv17l2 | protein_coding |
| ENSMUSG00000035585 | 1118.63513 | -0.5593223 | 0.19445955 | 0.00035935 | 0.00685683 | Down | 66078 | tRNA splicing endonuclease subunit 34 [Source:MGI Symbol;Acc:MGI:1913328] | Tsen34 | protein_coding |
| ENSMUSG00000035595 | 142.161471 | -0.6691065 | 0.23842308 | 0.00038645 | 0.00714211 | Down | 69770 | family with sequence similarity 174, member C [Source:MGI Symbol;Acc:MGI:1917020] | Fam174c | protein_coding |
| ENSMUSG00000035597 | 823.170023 | 0.61243744 | 0.14659173 | 3.03E-06 | 0.00030559 | Up | 328110 | pre-mRNA processing factor 39 [Source:MGI Symbol;Acc:MGI:104602] | Prpf39 | protein_coding |
| ENSMUSG00000035614 | 516.228135 | 0.35692788 | 0.13907519 | 0.00199203 | 0.02076619 | Up | 328108 | TOG array regulator of axonemal microtubules 1 [Source:MGI Symbol;Acc:MGI:2684313] | Togaram1 | protein_coding |
| ENSMUSG00000035623 | 602.847542 | 0.25713409 | 0.09418852 | 0.00218369 | 0.02196533 | Up | 233532 | remodeling and spacing factor 1 [Source:MGI Symbol;Acc:MGI:2682305] | Rsf1 | protein_coding |
| ENSMUSG00000035642 | 677.287776 | -0.4999577 | 0.1476028 | 7.70E-05 | 0.00246157 | Down | 66273 | adipogenesis associated Mth938 domain containing [Source:MGI Symbol;Acc:MGI:1913523] | Aamdc | protein_coding |
| ENSMUSG00000035649 | 680.16679 | 0.34313139 | 0.15776167 | 0.00566704 | 0.04095243 | Up | 319885 | zinc finger, CCHC domain containing 7 [Source:MGI Symbol;Acc:MGI:2442912] | Zcchc7 | protein_coding |
| ENSMUSG00000035696 | 1098.23574 | 0.36178812 | 0.14853096 | 0.00283591 | 0.02609298 | Up | 73469 | ring finger protein 38 [Source:MGI Symbol;Acc:MGI:1920719] | Rnf38 | protein_coding |
| ENSMUSG00000035725 | 634.689861 | 0.44077279 | 0.14653738 | 0.00036863 | 0.0069552 | Up | 19108 | protein kinase, X-linked [Source:MGI Symbol;Acc:MGI:1309999] | Prkx | protein_coding |
| ENSMUSG00000035726 | 724.700507 | 0.27953629 | 0.10461866 | 0.00232563 | 0.02291075 | Up | 114741 | SPT16, facilitates chromatin remodeling subunit [Source:MGI Symbol;Acc:MGI:1890948] | Supt16 | protein_coding |
| ENSMUSG00000035754 | 978.828914 | -0.3092037 | 0.09790873 | 0.00043432 | 0.00760296 | Down | 216156 | WD repeat domain 18 [Source:MGI Symbol;Acc:MGI:2158400] | Wdr18 | protein_coding |
| ENSMUSG00000035762 | 235.155985 | 0.51316938 | 0.12910714 | 8.42E-06 | 0.00060539 | Up | 72745 | transmembrane protein 161B [Source:MGI Symbol;Acc:MGI:1919995] | Tmem161b | protein_coding |
| ENSMUSG00000035783 | 4303.23063 | -2.0422096 | 0.2940581 | 1.66E-13 | 1.35E-09 | Down | 11475 | actin alpha 2, smooth muscle, aorta [Source:MGI Symbol;Acc:MGI:87909] | Acta2 | protein_coding |
| ENSMUSG00000035798 | 613.085446 | 0.30949683 | 0.0826069 | 4.74E-05 | 0.00183687 | Up | 320150 | zinc finger, DHHC domain containing 17 [Source:MGI Symbol;Acc:MGI:2445110] | Zdhhc17 | protein_coding |
| ENSMUSG00000035799 | 562.168409 | -0.3389764 | 0.1410227 | 0.00327007 | 0.02863734 | Down | 22160 | twist basic helix-loop-helix transcription factor 1 [Source:MGI Symbol;Acc:MGI:98872] | Twist1 | protein_coding |

| | | | | | | | | | | |
|---|---|---|---|---|---|---|---|---|---|---|
| ENSMUSG00000035847 | 1764.46263 | 0.28790671 | 0.11463124 | 0.00354872 | 0.0301354 | Up | 15931 | iduronate 2-sulfatase [Source:MGI Symbol;Acc:MGI:96417] | Ids | protein_coding |
| ENSMUSG00000035910 | 228.483744 | 0.00909874 | 0.19851929 | 1.87E-08 | 9.20E-06 | Up | 195208 | doublecortin domain containing 2a [Source:MGI Symbol;Acc:MGI:2652818] | Dcdc2a | protein_coding |
| ENSMUSG00000035941 | 731.262109 | 0.28000964 | 0.10343294 | 0.00205909 | 0.02117888 | Up | 108837 | inhibitor of Bruton agammaglobulinemia tyrosine kinase [Source:MGI Symbol;Acc:MGI:1918677] | Ibtk | protein_coding |
| ENSMUSG00000035960 | 144.996888 | -0.8321504 | 0.3853982 | 0.00136096 | 0.01629237 | Down | 11792 | apurinic/apyrimidinic endonuclease 1 [Source:MGI Symbol;Acc:MGI:88042] | Apex1 | protein_coding |
| ENSMUSG00000035967 | 537.05683 | 0.45928577 | 0.18254966 | 0.00135522 | 0.01623561 | Up | 236790 | integrator complex subunit 6 like [Source:MGI Symbol;Acc:MGI:2442593] | Ints6l | protein_coding |
| ENSMUSG00000035992 | 801.996398 | 0.56756207 | 0.15825738 | 3.24E-05 | 0.00145985 | Up | 216742 | folliculin interacting protein 1 [Source:MGI Symbol;Acc:MGI:2444668] | Fnip1 | protein_coding |
| ENSMUSG00000036040 | 124.946552 | -1.4963384 | 0.8035876 | 0.00151533 | 0.01739686 | Down | 77794 | ADAMTS-like 2 [Source:MGI Symbol;Acc:MGI:1925044] | Adamtsl2 | protein_coding |
| ENSMUSG00000036053 | 548.405525 | 0.47000104 | 0.15072547 | 0.00023739 | 0.00527371 | Up | 71409 | formin-like 2 [Source:MGI Symbol;Acc:MGI:1918659] | Fmnl2 | protein_coding |
| ENSMUSG00000036106 | 642.844559 | -0.4120609 | 0.16085161 | 0.00146248 | 0.01698215 | Down | 109270 | proline rich 5 (renal) [Source:MGI Symbol;Acc:MGI:1924714] | Prr5 | protein_coding |
| ENSMUSG00000036114 | 185.925067 | -0.4929379 | 0.30354713 | 0.00752584 | 0.0497993 | Down | 69961 | ribonuclease P/MRP 25 subunit-like [Source:MGI Symbol;Acc:MGI:1917211] | Rpp25l | protein_coding |
| ENSMUSG00000036138 | 3275.59151 | -0.3325946 | 0.1245036 | 0.00173311 | 0.01885755 | Down | 113868 | acetyl-Coenzyme A acyltransferase 1A [Source:MGI Symbol;Acc:MGI:2148491] | Acaa1a | protein_coding |
| ENSMUSG00000036181 | 3165.86482 | -0.6475797 | 0.22769596 | 0.00029964 | 0.00610744 | Down | 50708 | H1.2 linker histone, cluster member [Source:MGI Symbol;Acc:MGI:1931526] | H1f2 | protein_coding |
| ENSMUSG00000036197 | 501.892733 | 0.2508839 | 0.09231161 | 0.00237459 | 0.02321049 | Up | 223827 | glucoside xylosyltransferase 1 [Source:MGI Symbol;Acc:MGI:2684933] | Gxylt1 | protein_coding |
| ENSMUSG00000036199 | 1369.03687 | -0.6554456 | 0.19782471 | 7.31E-05 | 0.00237067 | Down | 67184 | NADH:ubiquinone oxidoreductase subunit A13 [Source:MGI Symbol;Acc:MGI:1914434] | Ndufa13 | protein_coding |
| ENSMUSG00000036202 | 367.979533 | 0.43808893 | 0.18493038 | 0.00222456 | 0.02225247 | Up | 51869 | replication timing regulatory factor 1 [Source:MGI Symbol;Acc:MGI:1098622] | Rif1 | protein_coding |
| ENSMUSG00000036241 | 2564.30441 | -0.1726876 | 0.04949866 | 0.0002699 | 0.00569518 | Down | 67615 | ubiquitin-conjugating enzyme E2R 2 [Source:MGI Symbol;Acc:MGI:1914865] | Ube2r2 | protein_coding |
| ENSMUSG00000036256 | 5808.75024 | -0.5003193 | 0.16427181 | 0.0002557 | 0.00549552 | Down | 29817 | insulin-like growth factor binding protein 7 [Source:MGI Symbol;Acc:MGI:1352480] | Igfbp7 | protein_coding |
| ENSMUSG00000036275 | 3262.91753 | -0.3007046 | 0.07799789 | 3.41E-05 | 0.00149515 | Down | 213673 | RIKEN cDNA 9530068E07 gene [Source:MGI Symbol;Acc:MGI:2654705] | 9530068E07Rik | protein_coding |
| ENSMUSG00000036278 | 465.304114 | -0.3868014 | 0.19535529 | 0.00690213 | 0.04699289 | Down | 107227 | mono-ADP ribosylhydrolase 1 [Source:MGI Symbol;Acc:MGI:2147583] | Macrod1 | protein_coding |
| ENSMUSG00000036282 | 681.765457 | 0.30991828 | 0.08907738 | 0.0001379 | 0.00364704 | Up | 70646 | N(alpha)-acetyltransferase 30, NatC catalytic subunit [Source:MGI Symbol;Acc:MGI:1922259] | Naa30 | protein_coding |
| ENSMUSG00000036291 | 469.758353 | 0.36529445 | 0.14280544 | 0.0019516 | 0.02046808 | Up | 74385 | adaptor-related protein complex 5, mu 1 subunit [Source:MGI Symbol;Acc:MGI:1921635] | Ap5m1 | protein_coding |
| ENSMUSG00000036298 | 532.840801 | 0.63989821 | 0.27280074 | 0.00127731 | 0.01553137 | Up | 239606 | solute carrier family 2 (facilitated glucose transporter), member 13 [Source:MGI Symbol;Acc:MGI:2146030] | Slc2a13 | protein_coding |
| ENSMUSG00000036371 | 3563.95461 | -0.3230431 | 0.13915376 | 0.00397101 | 0.03244919 | Down | 66870 | serpine1 mRNA binding protein 1 [Source:MGI Symbol;Acc:MGI:1914120] | Serbp1 | protein_coding |
| ENSMUSG00000036372 | 279.146003 | -0.9836093 | 0.27439045 | 1.78E-05 | 0.00100229 | Down | 69038 | transmembrane protein 258 [Source:MGI Symbol;Acc:MGI:1916288] | Tmem258 | protein_coding |
| ENSMUSG00000036376 | 190.279339 | -0.656667 | 0.18697125 | 3.48E-05 | 0.00150778 | Down | 30946 | activator of basal transcription 1 [Source:MGI Symbol;Acc:MGI:1353636] | Abt1 | protein_coding |
| ENSMUSG00000036390 | 189.730981 | -1.1588236 | 0.33860027 | 2.85E-05 | 0.00136288 | Down | 13197 | growth arrest and DNA-damage-inducible 45 alpha [Source:MGI Symbol;Acc:MGI:107799] | Gadd45a | protein_coding |
| ENSMUSG00000036391 | 790.603628 | 0.49504874 | 0.15106624 | 0.00012369 | 0.00337129 | Up | 77371 | Sec24 related gene family, member A (S. cerevisiae) [Source:MGI Symbol;Acc:MGI:1924621] | Sec24a | protein_coding |
| ENSMUSG00000036430 | 231.273287 | -0.9979729 | 0.22043431 | 3.26E-07 | 6.78E-05 | Down | 72726 | tubulin-specific chaperone C [Source:MGI Symbol;Acc:MGI:1919976] | Tbcc | protein_coding |
| ENSMUSG00000036437 | 38.332424 | 0.59653364 | 0.4296828 | 0.00740649 | 0.04931086 | Up | 18166 | neuropeptide Y receptor Y1 [Source:MGI Symbol;Acc:MGI:104963] | Npy1r | protein_coding |
| ENSMUSG00000036442 | 444.244635 | -0.4187891 | 0.11212894 | 2.99E-05 | 0.00141298 | Down | 59016 | THAP domain containing 11 [Source:MGI Symbol;Acc:MGI:1930964] | Thap11 | protein_coding |
| ENSMUSG00000036513 | 345.599347 | -0.3807441 | 0.16688021 | 0.00371214 | 0.0310638 | Down | 52245 | COMM domain containing 2 [Source:MGI Symbol;Acc:MGI:1098806] | Commd2 | protein_coding |
| ENSMUSG00000036533 | 396.171437 | -0.4637141 | 0.24196743 | 0.00514528 | 0.03841138 | Down | 260409 | CDC42 effector protein (Rho GTPase binding) 3 [Source:MGI Symbol;Acc:MGI:2384718] | Cdc42ep3 | protein_coding |
| ENSMUSG00000036545 | 3772.47315 | -0.4878136 | 0.16742713 | 0.00040331 | 0.00732601 | Down | 216725 | a disintegrin-like and metallopeptidase (reprolysin type) with thrombospondin type 1 motif, 2 [Source:MGI Symbol;Acc:MGI:1347356] | Adamts2 | protein_coding |
| ENSMUSG00000036564 | 180.440914 | -0.4443215 | 0.20144822 | 0.00322772 | 0.02837355 | Down | 234593 | N-myc downstream regulated gene 4 [Source:MGI Symbol;Acc:MGI:2384590] | Ndrg4 | protein_coding |
| ENSMUSG00000036570 | 1429.79152 | -0.5672735 | 0.19985276 | 0.00041018 | 0.00738372 | Down | 56188 | FXYD domain-containing ion transport regulator 1 [Source:MGI Symbol;Acc:MGI:1889273] | Fxyd1 | protein_coding |
| ENSMUSG00000036599 | 1380.65229 | -0.6678453 | 0.18286121 | 2.11E-05 | 0.00111477 | Down | 59031 | carbohydrate sulfotransferase 12 [Source:MGI Symbol;Acc:MGI:1929064] | Chst12 | protein_coding |
| ENSMUSG00000036622 | 1330.87757 | -0.3388958 | 0.1392045 | 0.00304714 | 0.02732424 | Down | 74772 | ATPase type 13A2 [Source:MGI Symbol;Acc:MGI:1922022] | Atp13a2 | protein_coding |
| ENSMUSG00000036639 | 101.28509 | -0.4849275 | 0.26816863 | 0.00583335 | 0.04172976 | Down | 17766 | nudix (nucleoside diphosphate linked moiety X)-type motif 1 [Source:MGI Symbol;Acc:MGI:109280] | Nudt1 | protein_coding |
| ENSMUSG00000036646 | 1588.05449 | -0.270133 | 0.09239367 | 0.00112697 | 0.01433253 | Down | 227619 | mannosidase, alpha, class 1B, member 1 [Source:MGI Symbol;Acc:MGI:2684954] | Man1b1 | protein_coding |
| ENSMUSG00000036678 | 212.736087 | -0.3453736 | 0.16114964 | 0.00584643 | 0.04177802 | Down | 223921 | achalasia, adrenocortical insufficiency, alacrimia [Source:MGI Symbol;Acc:MGI:2443767] | Aaas | protein_coding |
| ENSMUSG00000036748 | 755.991318 | -0.4553513 | 0.14777954 | 0.0002632 | 0.00561107 | Down | 67116 | CUE domain containing 2 [Source:MGI Symbol;Acc:MGI:1914366] | Cuedc2 | protein_coding |
| ENSMUSG00000036752 | 3450.33468 | -0.2861886 | 0.1259605 | 0.00632043 | 0.04411245 | Down | 227613 | tubulin, beta 4B class IVB [Source:MGI Symbol;Acc:MGI:1915472] | Tubb4b | protein_coding |
| ENSMUSG00000036779 | 636.094518 | 0.42932325 | 0.0908348 | 3.90E-07 | 7.37E-05 | Up | 214627 | terminal nucleotidyltransferase 4B [Source:MGI Symbol;Acc:MGI:1917820] | Tent4b | protein_coding |
| ENSMUSG00000036819 | 150.764165 | 0.33096267 | 0.13467909 | 0.00307339 | 0.02750811 | Up | 194952 | jumonji domain containing 4 [Source:MGI Symbol;Acc:MGI:2144404] | Jmjd4 | protein_coding |
| ENSMUSG00000036850 | 481.963373 | -0.7395835 | 0.23565944 | 0.00010871 | 0.00307142 | Down | 107733 | mitochondrial ribosomal protein L41 [Source:MGI Symbol;Acc:MGI:1333816] | Mrpl41 | protein_coding |
| ENSMUSG00000036863 | 508.289827 | 0.4879948 | 0.20697323 | 0.00182755 | 0.01957057 | Up | 214804 | synapse defective 1, Rho GTPase, homolog 2 (C. elegans) [Source:MGI Symbol;Acc:MGI:3036264] | Syde2 | protein_coding |
| ENSMUSG00000036873 | 244.128401 | -0.4102924 | 0.18352328 | 0.00333856 | 0.02895618 | Down | 66421 | RIKEN cDNA 2410004B18 gene [Source:MGI Symbol;Acc:MGI:1913671] | 2410004B18Rik | protein_coding |
| ENSMUSG00000036879 | 1650.06051 | 0.21482494 | 0.08510003 | 0.00553603 | 0.04041924 | Up | 102093 | phosphorylase kinase beta [Source:MGI Symbol;Acc:MGI:97578] | Phkb | protein_coding |
| ENSMUSG00000036880 | 2816.39588 | -0.346417 | 0.15026407 | 0.00405702 | 0.03286198 | Down | 52538 | acetyl-Coenzyme A acyltransferase 2 (mitochondrial 3-oxoacyl-Coenzyme A thiolase) [Source:MGI Symbol;Acc:MGI:1098623] | Acaa2 | protein_coding |
| ENSMUSG00000036887 | 2770.9272 | -0.7630481 | 0.35726958 | 0.00152473 | 0.01741052 | Down | 12259 | complement component 1, q subcomponent, alpha polypeptide [Source:MGI Symbol;Acc:MGI:88223] | C1qa | protein_coding |

| | | | | | | | | | | |
|---|---|---|---|---|---|---|---|---|---|---|
| ENSMUSG00000036916 | 267.943527 | 0.65794734 | 0.18189627 | 2.40E-05 | 0.00120579 | Up | 208968 | zinc finger protein 280C [Source:MGI Symbol;Acc:MGI:2387585] | Zfp280c | protein_coding |
| ENSMUSG00000036966 | 507.958678 | -0.3307692 | 0.12100398 | 0.00143274 | 0.01677936 | Down | 223918 | SPRY domain containing 3 [Source:MGI Symbol;Acc:MGI:2446175] | Spryd3 | protein_coding |
| ENSMUSG00000036968 | 342.905522 | -0.5079112 | 0.15056073 | 8.37E-05 | 0.00261825 | Down | 66455 | canopy FGF signaling regulator 4 [Source:MGI Symbol;Acc:MGI:1913705] | Cnpy4 | protein_coding |
| ENSMUSG00000036990 | 1248.40002 | 0.30466449 | 0.09100184 | 0.00023004 | 0.00514729 | Up | 73945 | OTU domain containing 4 [Source:MGI Symbol;Acc:MGI:1098801] | Otud4 | protein_coding |
| ENSMUSG00000037007 | 160.488615 | 0.54255929 | 0.16579191 | 0.00010835 | 0.0030665 | Up | 56314 | zinc finger protein 113 [Source:MGI Symbol;Acc:MGI:1929116] | Zfp113 | protein_coding |
| ENSMUSG00000037035 | 1337.09599 | -0.6306664 | 0.22135045 | 0.00029799 | 0.00610744 | Down | 16324 | inhibin beta-B [Source:MGI Symbol;Acc:MGI:96571] | Inhbb | protein_coding |
| ENSMUSG00000037049 | 2086.06777 | -0.3033505 | 0.09157623 | 0.0002409 | 0.00530995 | Down | 20597 | sphingomyelin phosphodiesterase 1, acid lysosomal [Source:MGI Symbol;Acc:MGI:98325] | Smpd1 | protein_coding |
| ENSMUSG00000037060 | 5505.54784 | -1.0565068 | 0.2187419 | 9.92E-08 | 2.83E-05 | Down | 109042 | caveolae associated 3 [Source:MGI Symbol;Acc:MGI:1923422] | Cavin3 | protein_coding |
| ENSMUSG00000037086 | 493.090075 | -1.110885 | 0.24661115 | 3.66E-07 | 7.26E-05 | Down | 68800 | proline rich 32 [Source:MGI Symbol;Acc:MGI:1916050] | Prr32 | protein_coding |
| ENSMUSG00000037089 | 781.463623 | -0.3125885 | 0.11435422 | 0.00159145 | 0.01785435 | Down | 73836 | solute carrier family 35, member B2 [Source:MGI Symbol;Acc:MGI:1921086] | Slc35b2 | protein_coding |
| ENSMUSG00000037095 | 5709.9861 | -0.8586603 | 0.27303137 | 9.91E-05 | 0.00290693 | Down | 76905 | leucine-rich alpha-2-glycoprotein 1 [Source:MGI Symbol;Acc:MGI:1924155] | Lrg1 | protein_coding |
| ENSMUSG00000037110 | 2609.99909 | 0.5241172 | 0.25006582 | 0.00315507 | 0.02797717 | Up | 241694 | Ral GTPase activating protein, alpha subunit 2 (catalytic) [Source:MGI Symbol;Acc:MGI:3036245] | Ralgapa2 | protein_coding |
| ENSMUSG00000037119 | 1613.58847 | 0.22811607 | 0.09229102 | 0.00527846 | 0.03920829 | Up | 210998 | family with sequence similarity 91, member A1 [Source:MGI Symbol;Acc:MGI:1277178] | Fam91a1 | protein_coding |
| ENSMUSG00000037151 | 157.522512 | -0.3652914 | 0.17414421 | 0.00547414 | 0.04012973 | Down | 216011 | leucine rich repeat containing 20 [Source:MGI Symbol;Acc:MGI:2387182] | Lrrc20 | protein_coding |
| ENSMUSG00000037152 | 1102.03512 | -0.4395656 | 0.18757434 | 0.00227419 | 0.02254506 | Down | 66377 | NADH:ubiquinone oxidoreductase subunit C1 [Source:MGI Symbol;Acc:MGI:1913627] | Ndufc1 | protein_coding |
| ENSMUSG00000037185 | 168.466782 | -0.969253 | 0.242854 | 3.94E-06 | 0.00036162 | Down | 74127 | keratin 80 [Source:MGI Symbol;Acc:MGI:1921377] | Krt80 | protein_coding |
| ENSMUSG00000037190 | 191.811951 | -0.9366968 | 0.20684701 | 3.76E-07 | 7.27E-05 | Down | 56368 | cytochrome b-561 domain containing 2 [Source:MGI Symbol;Acc:MGI:1929280] | Cyb561d2 | protein_coding |
| ENSMUSG00000037210 | 590.535631 | 0.38859626 | 0.15545636 | 0.00195294 | 0.02046808 | Up | 231128 | family with sequence homology 193, member A [Source:MGI Symbol;Acc:MGI:2447768] | Fam193a | protein_coding |
| ENSMUSG00000037224 | 114.194433 | -0.9058856 | 0.44023329 | 0.00144183 | 0.01685071 | Down | 231125 | zinc finger, FYVE domain containing 28 [Source:MGI Symbol;Acc:MGI:2684992] | Zfyve28 | protein_coding |
| ENSMUSG00000037257 | 853.993063 | -0.2787938 | 0.115506 | 0.00467284 | 0.03606139 | Down | 66939 | alpha- and gamma-adaptin binding protein [Source:MGI Symbol;Acc:MGI:1914189] | Aagab | protein_coding |
| ENSMUSG00000037286 | 1106.37819 | 0.34789882 | 0.14363086 | 0.00300707 | 0.02707859 | Up | 20842 | stromal antigen 1 [Source:MGI Symbol;Acc:MGI:1098658] | Stag1 | protein_coding |
| ENSMUSG00000037296 | 351.652994 | -0.272834 | 0.1151706 | 0.00513973 | 0.03840728 | Down | 67207 | LSM1 homolog, mRNA degradation associated [Source:MGI Symbol;Acc:MGI:1914457] | Lsm1 | protein_coding |
| ENSMUSG00000037343 | 676.808183 | 0.21762761 | 0.08507528 | 0.0046551 | 0.0359934 | Up | 319944 | TATA-box binding protein associated factor 2 [Source:MGI Symbol;Acc:MGI:2443028] | Taf2 | protein_coding |
| ENSMUSG00000037347 | 132.42011 | -0.6216802 | 0.25305481 | 0.00099038 | 0.01329642 | Down | 60322 | carbohydrate (N-acetylglucosamino) sulfotransferase 7 [Source:MGI Symbol;Acc:MGI:1891767] | Chst7 | protein_coding |
| ENSMUSG00000037348 | 774.862154 | -0.9314869 | 0.25704276 | 1.61E-05 | 0.0009316 | Down | 71904 | progestin and adipoQ receptor family member VII [Source:MGI Symbol;Acc:MGI:1919154] | Paqr7 | protein_coding |
| ENSMUSG00000037349 | 170.318377 | -0.700301 | 0.21783761 | 9.19E-05 | 0.00276846 | Down | 68323 | nudix (nucleoside diphosphate linked moiety X)-type motif 22 [Source:MGI Symbol;Acc:MGI:1915573] | Nudt22 | protein_coding |
| ENSMUSG00000037369 | 501.215883 | 0.32802853 | 0.13489778 | 0.00331699 | 0.02887705 | Up | 22289 | lysine (K)-specific demethylase 6A [Source:MGI Symbol;Acc:MGI:1095419] | Kdm6a | protein_coding |
| ENSMUSG00000037375 | 52.8564922 | -0.7468766 | 0.22897987 | 7.43E-05 | 0.00240273 | Down | 226861 | hedgehog acyltransferase [Source:MGI Symbol;Acc:MGI:2444681] | Hhat | protein_coding |
| ENSMUSG00000037379 | 174.691099 | -0.8676647 | 0.38234568 | 0.00102055 | 0.01344594 | Down | 100689 | spondin 2, extracellular matrix protein [Source:MGI Symbol;Acc:MGI:1923724] | Spon2 | protein_coding |
| ENSMUSG00000037400 | 957.157871 | 0.33795375 | 0.15028597 | 0.00494339 | 0.03754341 | Up | 76295 | ATPase, class VI, type 11B [Source:MGI Symbol;Acc:MGI:1923545] | Atp11b | protein_coding |
| ENSMUSG00000037406 | 153.020292 | -0.6628695 | 0.23940391 | 0.00040326 | 0.00732601 | Down | 330723 | HtrA serine peptidase 4 [Source:MGI Symbol;Acc:MGI:3036260] | Htra4 | protein_coding |
| ENSMUSG00000037419 | 664.064098 | -0.2825516 | 0.09917913 | 0.00139489 | 0.01655278 | Down | 71946 | endonuclease domain containing 1 [Source:MGI Symbol;Acc:MGI:1919196] | Endod1 | protein_coding |
| ENSMUSG00000037443 | 564.4431 | 0.59452303 | 0.21911685 | 0.00053691 | 0.00880853 | Up | 70012 | centrosomal protein 85 [Source:MGI Symbol;Acc:MGI:1917262] | Cep85 | protein_coding |
| ENSMUSG00000037499 | 1208.18678 | -0.6862955 | 0.17353133 | 6.16E-06 | 0.00047651 | Down | 66208 | neuron derived neurotrophic factor [Source:MGI Symbol;Acc:MGI:1913458] | Nenf | protein_coding |
| ENSMUSG00000037563 | 3854.85733 | -0.8675089 | 0.19945494 | 8.85E-07 | 0.0001353 | Down | 20055 | ribosomal protein S16 [Source:MGI Symbol;Acc:MGI:98118] | Rps16 | protein_coding |
| ENSMUSG00000037608 | 1447.32862 | 0.48742382 | 0.13092936 | 2.53E-05 | 0.00124403 | Up | 72567 | BCL2-associated transcription factor 1 [Source:MGI Symbol;Acc:MGI:1917580] | Bclaf1 | protein_coding |
| ENSMUSG00000037640 | 206.033975 | 0.45242061 | 0.17827899 | 0.00134797 | 0.01617956 | Up | 22718 | zinc finger protein 60 [Source:MGI Symbol;Acc:MGI:99207] | Zfp60 | protein_coding |
| ENSMUSG00000037649 | 527.775378 | -0.5689013 | 0.29597682 | 0.00355456 | 0.03014273 | Down | 14998 | histocompatibility 2, class II, locus DMa [Source:MGI Symbol;Acc:MGI:95921] | H2-DMa | protein_coding |
| ENSMUSG00000037706 | 5859.53071 | -0.4050749 | 0.12141573 | 0.00010336 | 0.00297723 | Down | 12520 | CD81 antigen [Source:MGI Symbol;Acc:MGI:1096398] | Cd81 | protein_coding |
| ENSMUSG00000037740 | 537.070616 | -0.764495 | 0.25451496 | 0.0001907 | 0.00454899 | Down | 99045 | mitochondrial ribosomal protein S26 [Source:MGI Symbol;Acc:MGI:1333830] | Mrps26 | protein_coding |
| ENSMUSG00000037772 | 464.133924 | -0.4756413 | 0.15124703 | 0.00020344 | 0.0047145 | Down | 19935 | mitochondrial ribosomal protein L23 [Source:MGI Symbol;Acc:MGI:1196612] | Mrpl23 | protein_coding |
| ENSMUSG00000037787 | 746.14418 | -0.3743827 | 0.13460864 | 0.00093526 | 0.01289754 | Down | 68020 | cytochrome c oxidase assembly factor 8 [Source:MGI Symbol;Acc:MGI:1915270] | Coa8 | protein_coding |
| ENSMUSG00000037797 | 13.9217897 | -0.9683898 | 0.51230825 | 0.00206444 | 0.02119901 | Down | 26876 | alcohol dehydrogenase 4 (class II), pi polypeptide [Source:MGI Symbol;Acc:MGI:1349472] | Adh4 | protein_coding |
| ENSMUSG00000037805 | 3661.62291 | -0.5805281 | 0.17814763 | 0.00010036 | 0.00291573 | Down | 19896 | ribosomal protein L10A [Source:MGI Symbol;Acc:MGI:1343877] | Rpl10a | protein_coding |
| ENSMUSG00000037818 | 144.934261 | 0.39965986 | 0.17115463 | 0.00287709 | 0.02637436 | Up | 269423 | abhydrolase domain containing 18 [Source:MGI Symbol;Acc:MGI:1915468] | Abhd18 | protein_coding |
| ENSMUSG00000037820 | 4435.98315 | -0.3194509 | 0.12452329 | 0.00257295 | 0.02445731 | Down | 21817 | transglutaminase 2, C polypeptide [Source:MGI Symbol;Acc:MGI:98731] | Tgm2 | protein_coding |
| ENSMUSG00000037857 | 905.187234 | 0.30290733 | 0.10934106 | 0.00143151 | 0.01677936 | Up | 68564 | nuclear FMR1 interacting protein 2 [Source:MGI Symbol;Acc:MGI:1915814] | Nufip2 | protein_coding |
| ENSMUSG00000037894 | 981.321562 | -0.7322639 | 0.17048409 | 1.30E-06 | 0.00017279 | Down | 51788 | H2A.Z variant histone 1 [Source:MGI Symbol;Acc:MGI:1888388] | H2az1 | protein_coding |
| ENSMUSG00000037896 | 635.055051 | 0.29124299 | 0.09733311 | 0.00080205 | 0.01161263 | Up | 217864 | REST corepressor 1 [Source:MGI Symbol;Acc:MGI:106340] | Rcor1 | protein_coding |
| ENSMUSG00000037940 | 167.549095 | 0.46148658 | 0.23131495 | 0.00460643 | 0.03575318 | Up | 234515 | inositol polyphosphate-4-phosphatase, type II [Source:MGI Symbol;Acc:MGI:2158925] | Inpp4b | protein_coding |
| ENSMUSG00000037966 | 1362.24805 | -0.5577068 | 0.17883159 | 0.00017818 | 0.00435971 | Down | 18081 | ninjurin 1 [Source:MGI Symbol;Acc:MGI:1196617] | Ninj1 | protein_coding |
| ENSMUSG00000037997 | 316.111194 | 0.25507691 | 0.11205326 | 0.0075076 | 0.04969882 | Up | 101187 | poly (ADP-ribose) polymerase family, member 11 [Source:MGI Symbol;Acc:MGI:2141505] | Parp11 | protein_coding |
| ENSMUSG00000038002 | 741.940327 | 0.42302689 | 0.17023616 | 0.00186724 | 0.01979822 | Up | 57354 | cramped chromatin regulator 1 [Source:MGI Symbol;Acc:MGI:1930190] | Cramp1 | protein_coding |

| | | | | | | | | | | |
|---|---|---|---|---|---|---|---|---|---|---|
| ENSMUSG00000038055 | 261.292227 | -0.4706173 | 0.15846712 | 0.00038184 | 0.00711355 | Down | 58239 | dexamethasone-induced transcript [Source:MGI Symbol;Acc:MGI:1926236] | Dexi | protein_coding |
| ENSMUSG00000038069 | 249.247633 | 0.32666793 | 0.14083086 | 0.00444834 | 0.0348257 | Up | 70925 | CDKN2A interacting protein [Source:MGI Symbol;Acc:MGI:1918175] | Cdkn2aip | protein_coding |
| ENSMUSG00000038084 | 2076.57791 | 0.30881108 | 0.10406871 | 0.00088547 | 0.01238725 | Up | 74143 | OPA1, mitochondrial dynamin like GTPase [Source:MGI Symbol;Acc:MGI:1921393] | Opa1 | protein_coding |
| ENSMUSG00000038086 | 33.8823751 | -0.9839846 | 0.42434982 | 0.00083919 | 0.01191802 | Down | 69253 | heat shock protein 2 [Source:MGI Symbol;Acc:MGI:1916503] | Hspb2 | protein_coding |
| ENSMUSG00000038095 | 1324.05339 | 0.35162188 | 0.10083139 | 0.0001116 | 0.00312038 | Up | 243272 | strawberry notch 1 [Source:MGI Symbol;Acc:MGI:2384298] | Sbno1 | protein_coding |
| ENSMUSG00000038121 | 937.624146 | 0.2626667 | 0.10608708 | 0.00437492 | 0.03446687 | Up | 108654 | family with sequence similarity 210, member A [Source:MGI Symbol;Acc:MGI:1914000] | Fam210a | protein_coding |
| ENSMUSG00000038127 | 3479.05679 | 0.29986409 | 0.08787181 | 0.00018883 | 0.00452448 | Up | 67501 | coiled-coil domain containing 50 [Source:MGI Symbol;Acc:MGI:1914751] | Ccdc50 | protein_coding |
| ENSMUSG00000038156 | 245.647104 | 0.59190337 | 0.35072569 | 0.00510594 | 0.03827231 | Up | 233744 | spondin 1, (f-spondin) extracellular matrix protein [Source:MGI Symbol;Acc:MGI:2385287] | Spon1 | protein_coding |
| ENSMUSG00000038173 | 49.6838205 | 1.01811 | 0.39376558 | 0.00042039 | 0.00747173 | Up | 320981 | ectonucleotide pyrophosphatase/phosphodiesterase 6 [Source:MGI Symbol;Acc:MGI:2445171] | Enpp6 | protein_coding |
| ENSMUSG00000038250 | 527.030269 | 0.37736696 | 0.12059297 | 0.00032994 | 0.00651252 | Up | 74841 | ubiquitin specific peptidase 38 [Source:MGI Symbol;Acc:MGI:1922091] | Usp38 | protein_coding |
| ENSMUSG00000038267 | 624.1214 | 0.55141858 | 0.30386324 | 0.00489398 | 0.03727269 | Up | 73102 | solute carrier family 22, member 23 [Source:MGI Symbol;Acc:MGI:1920352] | Slc22a23 | protein_coding |
| ENSMUSG00000038312 | 688.204965 | -0.4134039 | 0.10624778 | 1.71E-05 | 0.00097401 | Down | 108687 | ER degradation enhancer, mannosidase alpha-like 2 [Source:MGI Symbol;Acc:MGI:1915540] | Edem2 | protein_coding |
| ENSMUSG00000038344 | 1357.66534 | 0.52566821 | 0.20497746 | 0.00095345 | 0.01305827 | Up | 353170 | taxilin gamma [Source:MGI Symbol;Acc:MGI:3590652] | Txlng | protein_coding |
| ENSMUSG00000038369 | 975.93599 | 0.34912587 | 0.16921588 | 0.00740619 | 0.04931086 | Up | 56406 | nuclear receptor coactivator 6 [Source:MGI Symbol;Acc:MGI:1929915] | Ncoa6 | protein_coding |
| ENSMUSG00000038374 | 493.32299 | -0.3940594 | 0.2028128 | 0.00668703 | 0.04583579 | Down | 60365 | RNA binding motif protein 8a [Source:MGI Symbol;Acc:MGI:1913129] | Rbm8a | protein_coding |
| ENSMUSG00000038387 | 2911.33647 | -0.8075574 | 0.11943388 | 1.02E-12 | 4.13E-09 | Down | 20130 | related RAS viral (r-ras) oncogene [Source:MGI Symbol;Acc:MGI:98179] | Rras | protein_coding |
| ENSMUSG00000038412 | 782.505895 | -0.731852 | 0.28684293 | 0.00061458 | 0.0096369 | Down | 56295 | HIG1 domain family, member 1A [Source:MGI Symbol;Acc:MGI:1930666] | Higd1a | protein_coding |
| ENSMUSG00000038446 | 622.283635 | 0.31203708 | 0.14529425 | 0.00723153 | 0.04848376 | Up | 71713 | cell division cycle 40 [Source:MGI Symbol;Acc:MGI:1918963] | Cdc40 | protein_coding |
| ENSMUSG00000038451 | 167.462184 | -0.7474219 | 0.23431623 | 9.17E-05 | 0.00276846 | Down | 14794 | splA/ryanodine receptor domain and SOCS box containing 2 [Source:MGI Symbol;Acc:MGI:1315199] | Spsb2 | protein_coding |
| ENSMUSG00000038467 | 2154.29585 | -0.4997371 | 0.14167748 | 4.94E-05 | 0.00187013 | Down | 75608 | charged multivesicular body protein 4B [Source:MGI Symbol;Acc:MGI:1922858] | Chmp4b | protein_coding |
| ENSMUSG00000038473 | 93.1632535 | -0.7630561 | 0.54746191 | 0.00513286 | 0.0383901 | Down | 70729 | nitric oxide synthase 1 (neuronal) adaptor protein [Source:MGI Symbol;Acc:MGI:1917979] | Nos1ap | protein_coding |
| ENSMUSG00000038481 | 529.408755 | 0.24849259 | 0.10313035 | 0.00578361 | 0.04151777 | Up | 78334 | cyclin-dependent kinase 19 [Source:MGI Symbol;Acc:MGI:1925584] | Cdk19 | protein_coding |
| ENSMUSG00000038489 | 620.298974 | -0.7694049 | 0.37339436 | 0.00169675 | 0.01863067 | Down | 66491 | polymerase (RNA) II (DNA directed) polypeptide L [Source:MGI Symbol;Acc:MGI:1913741] | Polr2l | protein_coding |
| ENSMUSG00000038497 | 637.442725 | -0.2191556 | 0.07929756 | 0.00262984 | 0.02480871 | Down | 234076 | transmembrane and coiled-coil domains 3 [Source:MGI Symbol;Acc:MGI:2444946] | Tmco3 | protein_coding |
| ENSMUSG00000038515 | 310.571754 | -0.5251116 | 0.16793394 | 0.00019389 | 0.0045714 | Down | 66790 | GH regulated TBC protein 1 [Source:MGI Symbol;Acc:MGI:1914040] | Grtp1 | protein_coding |
| ENSMUSG00000038518 | 581.347058 | 0.39025414 | 0.09921807 | 1.54E-05 | 0.00090777 | Up | 16468 | jumonji, AT rich interactive domain 2 [Source:MGI Symbol;Acc:MGI:104813] | Jarid2 | protein_coding |
| ENSMUSG00000038520 | 1385.41961 | -0.2765867 | 0.11178165 | 0.00411356 | 0.03314717 | Down | 233204 | TBC1 domain family, member 17 [Source:MGI Symbol;Acc:MGI:2449973] | Tbc1d17 | protein_coding |
| ENSMUSG00000038527 | 318.160944 | -0.3837468 | 0.15607652 | 0.00227155 | 0.02254208 | Down | 232371 | complement component 1, r subcomponent-like [Source:MGI Symbol;Acc:MGI:2660692] | C1rl | protein_coding |
| ENSMUSG00000038535 | 360.507761 | 0.66440584 | 0.15142109 | 9.86E-07 | 0.00014478 | Up | 235469 | zinc finger protein 280D [Source:MGI Symbol;Acc:MGI:2384583] | Zfp280d | protein_coding |
| ENSMUSG00000038539 | 2597.4486 | -0.6518465 | 0.19039359 | 4.89E-05 | 0.00186708 | Down | 107503 | activating transcription factor 5 [Source:MGI Symbol;Acc:MGI:2141857] | Atf5 | protein_coding |
| ENSMUSG00000038550 | 25.6666807 | 0.97606439 | 0.66018058 | 0.00371809 | 0.03108259 | Up | 229599 | circadian associated repressor of transcription [Source:MGI Symbol;Acc:MGI:2684975] | Ciart | protein_coding |
| ENSMUSG00000038594 | 512.120936 | 0.85032938 | 0.15729186 | 4.48E-09 | 3.64E-06 | Up | 100038725 | centrosomal protein 85-like [Source:MGI Symbol;Acc:MGI:3642684] | Cep85l | protein_coding |
| ENSMUSG00000038622 | 278.342753 | -0.5105162 | 0.22645062 | 0.00216443 | 0.02189366 | Down | 69790 | mediator complex subunit 30 [Source:MGI Symbol;Acc:MGI:1917040] | Med30 | protein_coding |
| ENSMUSG00000038633 | 3287.06271 | -0.4269652 | 0.17277036 | 0.00175395 | 0.01901671 | Down | 13244 | delta(4)-desaturase, sphingolipid 1 [Source:MGI Symbol;Acc:MGI:1097711] | Degs1 | protein_coding |
| ENSMUSG00000038658 | 1000.16293 | 0.31429816 | 0.10675378 | 0.00083901 | 0.01191802 | Up | 226089 | RAB6A GEF complex partner 1 [Source:MGI Symbol;Acc:MGI:1924893] | Ric1 | protein_coding |
| ENSMUSG00000038705 | 296.63163 | 0.31571488 | 0.10771268 | 0.00085753 | 0.01213459 | Up | 229004 | glucocorticoid modulatory element binding protein 2 [Source:MGI Symbol;Acc:MGI:2652836] | Gmeb2 | protein_coding |
| ENSMUSG00000038775 | 116.857328 | -0.9593753 | 0.23951848 | 3.66E-06 | 0.00035407 | Down | 22351 | villin-like [Source:MGI Symbol;Acc:MGI:1201781] | Vill | protein_coding |
| ENSMUSG00000038776 | 1594.69602 | -0.8031627 | 0.18829718 | 1.59E-06 | 0.00019629 | Down | 13849 | epoxide hydrolase 1, microsomal [Source:MGI Symbol;Acc:MGI:95405] | Ephx1 | protein_coding |
| ENSMUSG00000038807 | 1061.29643 | -0.6862402 | 0.29201552 | 0.00108889 | 0.01403896 | Down | 380711 | RAP1 GTPase activating protein 2 [Source:MGI Symbol;Acc:MGI:3028623] | Rap1gap2 | protein_coding |
| ENSMUSG00000038860 | 267.894404 | 0.42361964 | 0.13273171 | 0.00021851 | 0.00496458 | Up | 99326 | GTPase activating RANGAP domain-like 3 [Source:MGI Symbol;Acc:MGI:2139309] | Garnl3 | protein_coding |
| ENSMUSG00000038880 | 486.286609 | -0.7319419 | 0.2549641 | 0.00025746 | 0.00552495 | Down | 79044 | mitochondrial ribosomal protein S34 [Source:MGI Symbol;Acc:MGI:1930188] | Mrps34 | protein_coding |
| ENSMUSG00000038888 | 139.375245 | -0.3419801 | 0.14876361 | 0.0042256 | 0.03381519 | Down | 233189 | cytosolic thiouridylase subunit 1 [Source:MGI Symbol;Acc:MGI:2385277] | Ctu1 | protein_coding |
| ENSMUSG00000038909 | 1111.78822 | 0.2526316 | 0.1094043 | 0.00753112 | 0.04981396 | Up | 217127 | K(lysine) acetyltransferase 7 [Source:MGI Symbol;Acc:MGI:2182799] | Kat7 | protein_coding |
| ENSMUSG00000038914 | 1763.60412 | 0.48440031 | 0.20928686 | 0.00207507 | 0.02122768 | Up | 23856 | death inducer-obliterator 1 [Source:MGI Symbol;Acc:MGI:1344352] | Dido1 | protein_coding |
| ENSMUSG00000038949 | 995.844685 | 0.56383575 | 0.18122788 | 0.00018321 | 0.00444223 | Up | 226744 | consortin, connexin sorting protein [Source:MGI Symbol;Acc:MGI:2445141] | Cnst | protein_coding |
| ENSMUSG00000038990 | 311.527439 | 0.27464083 | 0.12188641 | 0.00700731 | 0.04750994 | Up | 252966 | CDK5 and Abl enzyme substrate 2 [Source:MGI Symbol;Acc:MGI:2182335] | Cables2 | protein_coding |
| ENSMUSG00000039016 | 535.279253 | -0.383675 | 0.18040033 | 0.00478404 | 0.03671039 | Down | 30057 | translocase of inner mitochondrial membrane 8B [Source:MGI Symbol;Acc:MGI:1353424] | Timm8b | protein_coding |
| ENSMUSG00000039041 | 1846.16152 | -0.3092832 | 0.1308336 | 0.00419682 | 0.0336513 | Down | 56436 | adhesion regulating molecule 1 [Source:MGI Symbol;Acc:MGI:1929289] | Adrm1 | protein_coding |
| ENSMUSG00000039065 | 397.821167 | -0.2842643 | 0.11045143 | 0.00299347 | 0.02701153 | Down | 68073 | ATP synthase C subunit lysine N-methyltransferase [Source:MGI Symbol;Acc:MGI:1915323] | Atpsckmt | protein_coding |
| ENSMUSG00000039087 | 1457.78434 | 0.42414048 | 0.20750116 | 0.00485333 | 0.03708776 | Up | 68750 | ras responsive element binding protein 1 [Source:MGI Symbol;Acc:MGI:2443664] | Rreb1 | protein_coding |
| ENSMUSG00000039167 | 989.550008 | 0.53357268 | 0.22192388 | 0.0014109 | 0.01664495 | Up | 170757 | adhesion G protein-coupled receptor L4 [Source:MGI Symbol;Acc:MGI:2655562] | Adgrl4 | protein_coding |

| | | | | | | | | | | |
|---|---|---|---|---|---|---|---|---|---|---|
| ENSMUSG00000039168 | 2589.69941 | -0.494158 | 0.18538749 | 0.00080128 | 0.01161263 | Down | 223453 | death-associated protein [Source:MGI Symbol;Acc:MGI:1918190] | Dap | protein_coding |
| ENSMUSG00000039195 | 711.441808 | -0.6118258 | 0.19445274 | 0.00011936 | 0.00328649 | Down | 73737 | bublin coiled coil protein [Source:MGI Symbol;Acc:MGI:1920987] | Bbln | protein_coding |
| ENSMUSG00000039206 | 793.036478 | -0.3605208 | 0.17070023 | 0.00583369 | 0.04172976 | Down | 231871 | diacylglycerol lipase, beta [Source:MGI Symbol;Acc:MGI:2442032] | Daglb | protein_coding |
| ENSMUSG00000039208 | 530.182364 | -0.5852708 | 0.23524896 | 0.00105967 | 0.01379348 | Down | 210029 | meteorin, glial cell differentiation regulator-like [Source:MGI Symbol;Acc:MGI:2384806] | Metrnl | protein_coding |
| ENSMUSG00000039210 | 353.786562 | 0.34977178 | 0.14775957 | 0.0033665 | 0.02910524 | Up | 67769 | G patch domain containing 2 [Source:MGI Symbol;Acc:MGI:1915019] | Gpatch2 | protein_coding |
| ENSMUSG00000039219 | 742.646609 | 0.36830996 | 0.1276457 | 0.00077387 | 0.01135635 | Up | 94246 | AT rich interactive domain 4B (RBP1-like) [Source:MGI Symbol;Acc:MGI:2137512] | Arid4b | protein_coding |
| ENSMUSG00000039221 | 530.873459 | -1.0258242 | 0.33228393 | 9.45E-05 | 0.00280835 | Down | 68028 | ribosomal protein L22 like 1 [Source:MGI Symbol;Acc:MGI:1915278] | Rpl22l1 | protein_coding |
| ENSMUSG00000039304 | 293.636312 | 0.80498206 | 0.27335633 | 0.00019261 | 0.00456711 | Up | 22035 | tumor necrosis factor (ligand) superfamily, member 10 [Source:MGI Symbol;Acc:MGI:107414] | Tnfsf10 | protein_coding |
| ENSMUSG00000039316 | 362.852628 | -0.4252378 | 0.20723103 | 0.00508358 | 0.03819739 | Down | 76438 | raftlin lipid raft linker 1 [Source:MGI Symbol;Acc:MGI:1923688] | Rftn1 | protein_coding |
| ENSMUSG00000039323 | 218.459757 | -1.4340177 | 0.4107827 | 1.98E-05 | 0.0010735 | Down | 16008 | insulin-like growth factor binding protein 2 [Source:MGI Symbol;Acc:MGI:96437] | Igfbp2 | protein_coding |
| ENSMUSG00000039347 | 1564.25887 | -0.2898394 | 0.11177345 | 0.002707 | 0.02534023 | Down | 76252 | ATPase, H+ transporting, lysosomal V0 subunit E2 [Source:MGI Symbol;Acc:MGI:1923502] | Atp6v0e2 | protein_coding |
| ENSMUSG00000039361 | 4728.74027 | 0.39408676 | 0.10351526 | 3.27E-05 | 0.00146413 | Up | 233489 | phosphatidylinositol binding clathrin assembly protein [Source:MGI Symbol;Acc:MGI:2385902] | Picalm | protein_coding |
| ENSMUSG00000039405 | 1153.18029 | -0.5149743 | 0.18667305 | 0.0005883 | 0.00939442 | Down | 76453 | protease, serine 23 [Source:MGI Symbol;Acc:MGI:1923703] | Prss23 | protein_coding |
| ENSMUSG00000039410 | 123.015157 | 0.40259039 | 0.19162564 | 0.00485371 | 0.03708776 | Up | 70673 | PR domain containing 16 [Source:MGI Symbol;Acc:MGI:1917923] | Prdm16 | protein_coding |
| ENSMUSG00000039450 | 472.383695 | -0.821947 | 0.19212245 | 1.34E-06 | 0.00017569 | Down | 67880 | dicarbonyl L-xylulose reductase [Source:MGI Symbol;Acc:MGI:1915130] | Dcxr | protein_coding |
| ENSMUSG00000039461 | 1003.95568 | -0.3605036 | 0.0991001 | 5.80E-05 | 0.00203498 | Down | 102791 | T cell leukemia translocation altered gene [Source:MGI Symbol;Acc:MGI:1918829] | Tcta | protein_coding |
| ENSMUSG00000039474 | 593.478151 | -0.3348971 | 0.13559094 | 0.00261868 | 0.02474716 | Down | 22393 | wolframin ER transmembrane glycoprotein [Source:MGI Symbol;Acc:MGI:1328355] | Wfs1 | protein_coding |
| ENSMUSG00000039476 | 47.3455743 | -1.0079222 | 0.3538171 | 0.00020589 | 0.00475336 | Down | 20204 | paired related homeobox 2 [Source:MGI Symbol;Acc:MGI:98218] | Prrx2 | protein_coding |
| ENSMUSG00000039478 | 190.802963 | 0.39040965 | 0.14983711 | 0.00150413 | 0.01730496 | Up | 78506 | mitochondrial calcium uptake family, member 3 [Source:MGI Symbol;Acc:MGI:1925756] | Micu3 | protein_coding |
| ENSMUSG00000039480 | 362.421631 | 0.27481264 | 0.11146064 | 0.00422436 | 0.03381519 | Up | 319638 | 5'-nucleotidase domain containing 1 [Source:MGI Symbol;Acc:MGI:2442446] | Nt5dc1 | protein_coding |
| ENSMUSG00000039515 | 4439.82617 | -0.2800328 | 0.11707457 | 0.00470642 | 0.03623495 | Down | 110854 | protein phosphatase 2 protein activator [Source:MGI Symbol;Acc:MGI:1346006] | Ptpa | protein_coding |
| ENSMUSG00000039656 | 707.243141 | -0.3968406 | 0.1688462 | 0.00275376 | 0.02563597 | Down | 20182 | retinoid X receptor beta [Source:MGI Symbol;Acc:MGI:98215] | Rxrb | protein_coding |
| ENSMUSG00000039680 | 900.108892 | -0.4482657 | 0.21493684 | 0.00378614 | 0.03139658 | Down | 121022 | mitochondrial ribosomal protein S6 [Source:MGI Symbol;Acc:MGI:2153111] | Mrps6 | protein_coding |
| ENSMUSG00000039704 | 726.655567 | 0.41506047 | 0.1312859 | 0.00025464 | 0.00549552 | Up | 320506 | LMBR1 domain containing 2 [Source:MGI Symbol;Acc:MGI:2444173] | Lmbrd2 | protein_coding |
| ENSMUSG00000039745 | 922.225621 | -0.5704997 | 0.19515812 | 0.00030253 | 0.00614327 | Down | 53415 | HIV-1 Tat interactive protein 2 [Source:MGI Symbol;Acc:MGI:1859271] | Htatip2 | protein_coding |
| ENSMUSG00000039782 | 1309.77219 | 0.43800313 | 0.19408643 | 0.00271585 | 0.0253995 | Up | 231207 | cytoplasmic polyadenylation element binding protein 2 [Source:MGI Symbol;Acc:MGI:2442640] | Cpeb2 | protein_coding |
| ENSMUSG00000039787 | 435.912673 | -0.5259015 | 0.18313237 | 0.0003655 | 0.00692825 | Down | 99151 | cerebral endothelial cell adhesion molecule [Source:MGI Symbol;Acc:MGI:2139134] | Cercam | protein_coding |
| ENSMUSG00000039789 | 154.485721 | 0.45143795 | 0.16596368 | 0.00081468 | 0.01171188 | Up | 71063 | zinc finger protein 597 [Source:MGI Symbol;Acc:MGI:1918313] | Zfp597 | protein_coding |
| ENSMUSG00000039798 | 15.6355827 | -0.8045412 | 0.78010539 | 0.00665876 | 0.04571914 | Down | NA | RIKEN cDNA 2600006K01 gene [Source:MGI Symbol;Acc:MGI:1917569] | 2600006K01Rik | lncRNA |
| ENSMUSG00000039841 | 256.618237 | 0.46692861 | 0.16373808 | 0.00053303 | 0.00879984 | Up | 627049 | zinc finger protein 800 [Source:MGI Symbol;Acc:MGI:1889334] | Zfp800 | protein_coding |
| ENSMUSG00000039929 | 448.985027 | 0.47005745 | 0.18260969 | 0.0011675 | 0.01467958 | Up | 207932 | URB1 ribosome biogenesis 1 homolog (S. cerevisiae) [Source:MGI Symbol;Acc:MGI:2146468] | Urb1 | protein_coding |
| ENSMUSG00000039943 | 1113.39509 | 0.58287577 | 0.16737425 | 5.72E-05 | 0.00201842 | Up | 18798 | phospholipase C, beta 4 [Source:MGI Symbol;Acc:MGI:107464] | Plcb4 | protein_coding |
| ENSMUSG00000039967 | 475.439921 | 0.3621129 | 0.17037467 | 0.00558684 | 0.04062551 | Up | 30046 | zinc finger protein 292 [Source:MGI Symbol;Acc:MGI:1353423] | Zfp292 | protein_coding |
| ENSMUSG00000039968 | 641.247433 | 0.40811177 | 0.10574159 | 2.13E-05 | 0.00111477 | Up | 242860 | round spermatid basic protein 1-like [Source:MGI Symbol;Acc:MGI:3036237] | Rsbn1l | protein_coding |
| ENSMUSG00000040021 | 1121.03309 | 0.36203429 | 0.09930508 | 5.60E-05 | 0.00199528 | Up | 16798 | large tumor suppressor [Source:MGI Symbol;Acc:MGI:1333883] | Lats1 | protein_coding |
| ENSMUSG00000040022 | 572.109617 | 0.33349292 | 0.12247511 | 0.00144998 | 0.01691077 | Up | 74998 | RAB11 family interacting protein 2 (class I) [Source:MGI Symbol;Acc:MGI:1922248] | Rab11fip2 | protein_coding |
| ENSMUSG00000040026 | 112.794857 | -3.0730019 | 0.75515585 | 1.69E-06 | 0.00020213 | Down | 20210 | serum amyloid A 3 [Source:MGI Symbol;Acc:MGI:98223] | Saa3 | protein_coding |
| ENSMUSG00000040029 | 1107.95853 | 0.21560375 | 0.09045201 | 0.00748723 | 0.04964493 | Up | 320727 | importin 8 [Source:MGI Symbol;Acc:MGI:2444611] | Ipo8 | protein_coding |
| ENSMUSG00000040037 | 781.048125 | 0.43804432 | 0.16575195 | 0.00106513 | 0.01384241 | Up | 320840 | neuronal growth regulator 1 [Source:MGI Symbol;Acc:MGI:2444846] | Negr1 | protein_coding |
| ENSMUSG00000040044 | 891.088034 | 0.32993551 | 0.14295808 | 0.00459366 | 0.03568821 | Up | 50793 | origin recognition complex, subunit 3 [Source:MGI Symbol;Acc:MGI:1354944] | Orc3 | protein_coding |
| ENSMUSG00000040105 | 767.918517 | 0.33714909 | 0.13064771 | 0.0021731 | 0.02191828 | Up | 74411 | phospholipid phosphatase 6 [Source:MGI Symbol;Acc:MGI:1921661] | Plpp6 | protein_coding |
| ENSMUSG00000040123 | 909.842054 | 0.35940301 | 0.1196277 | 0.00054268 | 0.0088592 | Up | 219105 | zinc finger, MYM-type 5 [Source:MGI Symbol;Acc:MGI:3041170] | Zmym5 | protein_coding |
| ENSMUSG00000040128 | 1500.92533 | -0.499779 | 0.12019287 | 3.85E-06 | 0.00036162 | Down | 108767 | proline-rich nuclear receptor coactivator 1 [Source:MGI Symbol;Acc:MGI:1917838] | Pnrc1 | protein_coding |
| ENSMUSG00000040152 | 8868.75012 | -1.2349351 | 0.38518992 | 5.20E-05 | 0.00191521 | Down | 21825 | thrombospondin 1 [Source:MGI Symbol;Acc:MGI:98737] | Thbs1 | protein_coding |
| ENSMUSG00000040181 | 3892.91234 | 0.50633445 | 0.17237945 | 0.0003589 | 0.00685683 | Up | 14261 | flavin containing monooxygenase 1 [Source:MGI Symbol;Acc:MGI:1310002] | Fmo1 | protein_coding |
| ENSMUSG00000040205 | 165.347886 | 0.0152959 | 0.19908681 | 0.00110139 | 0.01415518 | Up | 16433 | CUB and zona pellucida-like domains 1 [Source:MGI Symbol;Acc:MGI:1202881] | Cuzd1 | protein_coding |
| ENSMUSG00000040212 | 773.751419 | -0.6013178 | 0.18184355 | 8.58E-05 | 0.00264502 | Down | 13732 | epithelial membrane protein 3 [Source:MGI Symbol;Acc:MGI:1098729] | Emp3 | protein_coding |
| ENSMUSG00000040236 | 687.408425 | -0.4259693 | 0.1256395 | 0.00010511 | 0.00301348 | Down | 66682 | trafficking protein particle complex 5 [Source:MGI Symbol;Acc:MGI:1913932] | Trappc5 | protein_coding |
| ENSMUSG00000040269 | 421.124294 | -0.6111164 | 0.19647359 | 0.00015745 | 0.00403429 | Down | 66230 | mitochondrial ribosomal protein S28 [Source:MGI Symbol;Acc:MGI:1913480] | Mrps28 | protein_coding |
| ENSMUSG00000040283 | 1356.52437 | 0.6027709 | 0.31314452 | 0.0033116 | 0.02886105 | Up | 237754 | butyrophilin-like 9 [Source:MGI Symbol;Acc:MGI:2442439] | Btnl9 | protein_coding |
| ENSMUSG00000040297 | 688.695425 | 0.31451553 | 0.13996336 | 0.00557579 | 0.04058186 | Up | 226551 | SUN domain containing ossification factor [Source:MGI Symbol;Acc:MGI:2138346] | Suco | protein_coding |

| | | | | | | | | | | |
|---|---|---|---|---|---|---|---|---|---|---|
| ENSMUSG00000040325 | 622.970205 | 0.35057477 | 0.17014276 | 0.00696749 | 0.04730581 | Up | 321006 | DDB1 and CUL4 associated factor 1 [Source:MGI Symbol;Acc:MGI:2445220] | Dcaf1 | protein_coding |
| ENSMUSG00000040390 | 493.587818 | -0.3864341 | 0.18286047 | 0.00476323 | 0.03660297 | Down | 269881 | mitogen-activated protein kinase kinase kinase 10 [Source:MGI Symbol;Acc:MGI:1346879] | Map3k10 | protein_coding |
| ENSMUSG00000040396 | 564.662063 | 0.21862844 | 0.06503003 | 0.00034693 | 0.00670832 | Up | 68904 | abhydrolase domain containing 13 [Source:MGI Symbol;Acc:MGI:1916154] | Abhd13 | protein_coding |
| ENSMUSG00000040414 | 790.556647 | -0.3025706 | 0.11625092 | 0.00249071 | 0.02401966 | Down | 246696 | solute carrier family 25, member 28 [Source:MGI Symbol;Acc:MGI:2180509] | Slc25a28 | protein_coding |
| ENSMUSG00000040423 | 1195.98341 | 0.23633696 | 0.08558192 | 0.00232449 | 0.02291075 | Up | 381305 | RING CCCH (C3H) domains 1 [Source:MGI Symbol;Acc:MGI:2685397] | Rc3h1 | protein_coding |
| ENSMUSG00000040430 | 421.546513 | 0.39256519 | 0.15907739 | 0.00217496 | 0.02191828 | Up | 71795 | phosphatidylinositol transfer protein, cytoplasmic 1 [Source:MGI Symbol;Acc:MGI:1919045] | Pitpnc1 | protein_coding |
| ENSMUSG00000040455 | 448.421334 | 0.47881067 | 0.12519071 | 1.86E-05 | 0.00102764 | Up | 77593 | ubiquitin specific petidase 45 [Source:MGI Symbol;Acc:MGI:101850] | Usp45 | protein_coding |
| ENSMUSG00000040462 | 3251.8858 | -0.4717497 | 0.17020664 | 0.00066461 | 0.01017581 | Down | 216440 | amplified in osteosarcoma [Source:MGI Symbol;Acc:MGI:1924301] | Os9 | protein_coding |
| ENSMUSG00000040466 | 785.289471 | -0.4832402 | 0.18802558 | 0.00126114 | 0.01536926 | Down | 233016 | biliverdin reductase B (flavin reductase (NADPH)) [Source:MGI Symbol;Acc:MGI:2385271] | Blvrb | protein_coding |
| ENSMUSG00000040483 | 279.988694 | 0.42497705 | 0.16449496 | 0.00133993 | 0.0161362 | Up | 327959 | XIAP associated factor 1 [Source:MGI Symbol;Acc:MGI:3772572] | Xaf1 | protein_coding |
| ENSMUSG00000040521 | 361.050764 | -0.3808226 | 0.14004356 | 0.00112755 | 0.01433253 | Down | 66399 | Ts translation elongation factor, mitochondrial [Source:MGI Symbol;Acc:MGI:1913649] | Tsfm | protein_coding |
| ENSMUSG00000040557 | 375.11123 | -0.3784233 | 0.12201611 | 0.00036741 | 0.00694027 | Down | 79565 | methyltransferase like 27 [Source:MGI Symbol;Acc:MGI:1933146] | Mettl27 | protein_coding |
| ENSMUSG00000040563 | 207.184764 | -0.3423667 | 0.16217964 | 0.00638241 | 0.04443173 | Down | 235044 | phospholipid phosphatase related 2 [Source:MGI Symbol;Acc:MGI:2384575] | Plppr2 | protein_coding |
| ENSMUSG00000040605 | 283.615483 | -0.3869635 | 0.14132166 | 0.00107727 | 0.01394925 | Down | 56175 | beta-site APP-cleaving enzyme 2 [Source:MGI Symbol;Acc:MGI:1860440] | Bace2 | protein_coding |
| ENSMUSG00000040651 | 689.782357 | 0.43871193 | 0.14672154 | 0.00039193 | 0.00719432 | Up | 218850 | transcription activation suppressor [Source:MGI Symbol;Acc:MGI:1921694] | Tasor | protein_coding |
| ENSMUSG00000040690 | 764.667484 | -1.4311585 | 0.34250964 | 1.53E-06 | 0.00018939 | Down | 107581 | collagen, type XVI, alpha 1 [Source:MGI Symbol;Acc:MGI:1095396] | Col16a1 | protein_coding |
| ENSMUSG00000040731 | 3885.23154 | -0.2816722 | 0.10981524 | 0.0029291 | 0.02661705 | Down | 22384 | eukaryotic translation initiation factor 4H [Source:MGI Symbol;Acc:MGI:1341822] | Eif4h | protein_coding |
| ENSMUSG00000040767 | 147.123094 | -1.399756 | 0.28495396 | 4.57E-08 | 1.58E-05 | Down | 78372 | small nuclear ribonucleoprotein 25 (U11/U12) [Source:MGI Symbol;Acc:MGI:1925622] | Snrnp25 | protein_coding |
| ENSMUSG00000040813 | 1473.154 | -0.5657186 | 0.13429495 | 2.21E-06 | 0.00024802 | Down | 21767 | testis expressed gene 264 [Source:MGI Symbol;Acc:MGI:1096570] | Tex264 | protein_coding |
| ENSMUSG00000040818 | 687.154357 | 0.35446789 | 0.10698706 | 0.00019037 | 0.00454793 | Up | 211922 | DENN/MADD domain containing 6A [Source:MGI Symbol;Acc:MGI:2442980] | Dennd6a | protein_coding |
| ENSMUSG00000040824 | 617.971399 | -0.9531381 | 0.2241475 | 1.23E-06 | 0.0001654 | Down | 107686 | small nuclear ribonucleoprotein D2 [Source:MGI Symbol;Acc:MGI:98345] | Snrpd2 | protein_coding |
| ENSMUSG00000040865 | 729.448437 | 0.46708899 | 0.17201001 | 0.00078695 | 0.01148612 | Up | 227195 | INO80 complex subunit D [Source:MGI Symbol;Acc:MGI:3027003] | Ino80d | protein_coding |
| ENSMUSG00000040883 | 2051.37408 | -0.850393 | 0.18314963 | 2.46E-07 | 5.54E-05 | Down | 235043 | transmembrane protein 205 [Source:MGI Symbol;Acc:MGI:3045495] | Tmem205 | protein_coding |
| ENSMUSG00000040928 | 501.112716 | 0.51637367 | 0.14709055 | 4.97E-05 | 0.00187013 | Up | 74648 | S100P binding protein [Source:MGI Symbol;Acc:MGI:1921898] | S100pbp | protein_coding |
| ENSMUSG00000040943 | 441.75347 | 0.48938954 | 0.21317512 | 0.00215515 | 0.02185892 | Up | 214133 | tet methylcytosine dioxygenase 2 [Source:MGI Symbol;Acc:MGI:2443298] | Tet2 | protein_coding |
| ENSMUSG00000040952 | 5216.91828 | -0.4083952 | 0.1648697 | 0.00189224 | 0.02001266 | Down | 20085 | ribosomal protein S19 [Source:MGI Symbol;Acc:MGI:1333780] | Rps19 | protein_coding |
| ENSMUSG00000040966 | 33.8480044 | -1.2977559 | 0.49993572 | 0.00035613 | 0.00683838 | Down | 20518 | solute carrier family 22 (organic cation transporter), member 2 [Source:MGI Symbol;Acc:MGI:1335072] | Slc22a2 | protein_coding |
| ENSMUSG00000040972 | 17.8250805 | -1.0465793 | 0.64113833 | 0.00283767 | 0.02609298 | Down | 230868 | immunoglobulin superfamily, member 21 [Source:MGI Symbol;Acc:MGI:2681842] | Igsf21 | protein_coding |
| ENSMUSG00000041035 | 211.713462 | -0.4074849 | 0.18397889 | 0.00359715 | 0.03038774 | Down | 226162 | deleted in primary ciliary dyskinesia [Source:MGI Symbol;Acc:MGI:1924407] | Dpcd | protein_coding |
| ENSMUSG00000041078 | 50.1247514 | -2.1121291 | 0.69622187 | 7.82E-05 | 0.00249421 | Down | 14803 | glutamate receptor, ionotropic, delta 1 [Source:MGI Symbol;Acc:MGI:95812] | Grid1 | protein_coding |
| ENSMUSG00000041126 | 499.685057 | -0.3209844 | 0.14518439 | 0.00607412 | 0.04288314 | Down | 77605 | H2A.Z histone variant 2 [Source:MGI Symbol;Acc:MGI:1924855] | H2az2 | protein_coding |
| ENSMUSG00000041134 | 370.613101 | 1.08564746 | 0.28004537 | 5.54E-06 | 0.00044782 | Up | 224405 | cysteine and tyrosine-rich protein 1 [Source:MGI Symbol;Acc:MGI:2152187] | Cyyr1 | protein_coding |
| ENSMUSG00000041203 | 807.219934 | -0.6593578 | 0.24644155 | 0.00052839 | 0.00876789 | Down | 68544 | telomerase RNA component interacting RNase [Source:MGI Symbol;Acc:MGI:1922833] | Trir | protein_coding |
| ENSMUSG00000041220 | 1864.59227 | 1.47053119 | 0.35450205 | 1.49E-06 | 0.00018768 | Up | 170439 | ELOVL family member 6, elongation of long chain fatty acids (yeast) [Source:MGI Symbol;Acc:MGI:2156528] | Elovl6 | protein_coding |
| ENSMUSG00000041235 | 558.114421 | 0.60041347 | 0.22022474 | 0.00050882 | 0.00850397 | Up | 320790 | chromodomain helicase DNA binding protein 7 [Source:MGI Symbol;Acc:MGI:2444748] | Chd7 | protein_coding |
| ENSMUSG00000041297 | 1097.7857 | 0.29671441 | 0.10736167 | 0.001613 | 0.01799671 | Up | 69562 | cyclin-dependent kinase 13 [Source:MGI Symbol;Acc:MGI:1916812] | Cdk13 | protein_coding |
| ENSMUSG00000041319 | 182.510344 | -0.5422709 | 0.18193685 | 0.00027643 | 0.00577363 | Down | 386612 | THO complex 6 [Source:MGI Symbol;Acc:MGI:2677480] | Thoc6 | protein_coding |
| ENSMUSG00000041328 | 1057.48812 | 0.35377217 | 0.1633532 | 0.00536506 | 0.03958014 | Up | 74737 | PCF11 cleavage and polyadenylation factor subunit [Source:MGI Symbol;Acc:MGI:1919579] | Pcf11 | protein_coding |
| ENSMUSG00000041355 | 1385.35425 | -0.377601 | 0.16533146 | 0.00355326 | 0.03014273 | Down | 66256 | signal sequence receptor, beta [Source:MGI Symbol;Acc:MGI:1913506] | Ssr2 | protein_coding |
| ENSMUSG00000041390 | 1486.29822 | -0.3704959 | 0.18007553 | 0.00600994 | 0.04255949 | Down | 16543 | MyoD family inhibitor domain containing [Source:MGI Symbol;Acc:MGI:104611] | Mdfic | protein_coding |
| ENSMUSG00000041408 | 1700.7956 | 0.38711655 | 0.09003649 | 3.45E-06 | 0.00033982 | Up | 218914 | WAPL cohesin release factor [Source:MGI Symbol;Acc:MGI:2675859] | Wapl | protein_coding |
| ENSMUSG00000041415 | 1048.1058 | 0.30558175 | 0.14209993 | 0.00757378 | 0.04993745 | Up | 192119 | dicer 1, ribonuclease type III [Source:MGI Symbol;Acc:MGI:2177178] | Dicer1 | protein_coding |
| ENSMUSG00000041429 | 116.959059 | -0.4780187 | 0.20665031 | 0.00221778 | 0.02221538 | Down | 18207 | nth (endonuclease III)-like 1 (E.coli) [Source:MGI Symbol;Acc:MGI:1313275] | Nthl1 | protein_coding |
| ENSMUSG00000041444 | 553.564048 | 0.41989157 | 0.17620765 | 0.00232439 | 0.02291075 | Up | 330914 | Rho GTPase activating protein 32 [Source:MGI Symbol;Acc:MGI:2450166] | Arhgap32 | protein_coding |
| ENSMUSG00000041477 | 225.723226 | 0.28083212 | 0.11585355 | 0.00468542 | 0.03610755 | Up | 319618 | decapping mRNA 1B [Source:MGI Symbol;Acc:MGI:2442404] | Dcp1b | protein_coding |
| ENSMUSG00000041536 | 8.53921664 | -2.3260792 | 0.89567501 | 0.0003332 | 0.00656101 | Down | 74069 | serine (or cysteine) peptidase inhibitor, clade A, member 3A [Source:MGI Symbol;Acc:MGI:1921319] | Serpina3a | protein_coding |
| ENSMUSG00000041560 | 1491.07307 | -0.5071708 | 0.16047165 | 0.00016949 | 0.00421004 | Down | 68077 | NOP53 ribosome biogenesis factor [Source:MGI Symbol;Acc:MGI:2154441] | Nop53 | protein_coding |
| ENSMUSG00000041571 | 4814.9452 | -0.8872297 | 0.16673724 | 6.56E-09 | 4.85E-06 | Down | 20364 | selenoprotein W [Source:MGI Symbol;Acc:MGI:1100878] | Selenow | protein_coding |
| ENSMUSG00000041577 | 18059.9371 | -0.9931297 | 0.25169306 | 4.35E-06 | 0.00037622 | Down | 116847 | proline arginine-rich end leucine-rich repeat [Source:MGI Symbol;Acc:MGI:2151110] | Prelp | protein_coding |
| ENSMUSG00000041609 | 526.486874 | -0.5241538 | 0.14325593 | 3.24E-05 | 0.00145985 | Down | 75665 | BICD family like cargo adaptor 1 [Source:MGI Symbol;Acc:MGI:1922915] | Bicdl1 | protein_coding |
| ENSMUSG00000041633 | 899.252606 | 0.45364252 | 0.18930815 | 0.00201872 | 0.02091457 | Up | 207474 | potassium channel tetramerisation domain containing 12b [Source:MGI Symbol;Acc:MGI:2444667] | Kctd12b | protein_coding |

| | | | | | | | | | | |
|---|---|---|---|---|---|---|---|---|---|---|
| ENSMUSG00000041653 | 8619.22546 | 0.48627018 | 0.19290262 | 0.00124715 | 0.01526747 | Up | 116939 | patatin-like phospholipase domain containing 3 [Source:MGI Symbol;Acc:MGI:2151796] | Pnpla3 | protein_coding |
| ENSMUSG00000041685 | 1589.12115 | 0.4608267 | 0.11376191 | 7.10E-06 | 0.00052685 | Up | 218503 | FCH domain only 2 [Source:MGI Symbol;Acc:MGI:3505790] | Fcho2 | protein_coding |
| ENSMUSG00000041736 | 7268.71323 | -0.5976264 | 0.16925552 | 3.80E-05 | 0.00160355 | Down | 12257 | translocator protein [Source:MGI Symbol;Acc:MGI:88222] | Tspo | protein_coding |
| ENSMUSG00000041747 | 279.906283 | 0.41105099 | 0.13259713 | 0.00031252 | 0.0062678 | Up | 105372 | UTP15 small subunit processome component [Source:MGI Symbol;Acc:MGI:2145443] | Utp15 | protein_coding |
| ENSMUSG00000041763 | 1194.68996 | 0.29139486 | 0.11110018 | 0.00250029 | 0.02401966 | Up | 22019 | tripeptidyl peptidase II [Source:MGI Symbol;Acc:MGI:102724] | Tpp2 | protein_coding |
| ENSMUSG00000041845 | 186.753711 | -0.6573079 | 0.19879074 | 7.30E-05 | 0.00237067 | Down | 11854 | ras homolog family member D [Source:MGI Symbol;Acc:MGI:108446] | Rhod | protein_coding |
| ENSMUSG00000041846 | 770.167226 | 0.48859747 | 0.13426937 | 4.25E-05 | 0.00169206 | Up | 68734 | protein phosphatase 4 regulatory subunit 3A [Source:MGI Symbol;Acc:MGI:1915984] | Ppp4r3a | protein_coding |
| ENSMUSG00000041881 | 1537.2738 | -0.4829525 | 0.17089262 | 0.00052756 | 0.00876295 | Down | 66416 | NADH:ubiquinone oxidoreductase subunit A7 [Source:MGI Symbol;Acc:MGI:1913666] | Ndufa7 | protein_coding |
| ENSMUSG00000041915 | 551.810659 | 0.22208337 | 0.09162864 | 0.00641431 | 0.04458731 | Up | 225339 | AMME chromosomal region gene 1-like [Source:MGI Symbol;Acc:MGI:2442711] | Ammecr1l | protein_coding |
| ENSMUSG00000041930 | 89.2372744 | -0.667921 | 0.38743661 | 0.00396541 | 0.0324198 | Down | 433940 | family with sequence similarity 222, member A [Source:MGI Symbol;Acc:MGI:3605543] | Fam222a | protein_coding |
| ENSMUSG00000041935 | 2199.33066 | 0.31661284 | 0.14714948 | 0.00696847 | 0.04730581 | Up | 106064 | RAB7A interacting MON1-CCZ1 complex subunit 1 [Source:MGI Symbol;Acc:MGI:2146232] | Rimoc1 | protein_coding |
| ENSMUSG00000041939 | 671.59002 | -0.4500807 | 0.18608013 | 0.00162622 | 0.01810151 | Down | 17855 | mevalonate kinase [Source:MGI Symbol;Acc:MGI:107624] | Mvk | protein_coding |
| ENSMUSG00000041958 | 1354.99371 | -0.4174229 | 0.15106405 | 0.00082309 | 0.01178874 | Down | 276846 | phosphatidylinositol glycan anchor biosynthesis, class S [Source:MGI Symbol;Acc:MGI:2687325] | Pigs | protein_coding |
| ENSMUSG00000041997 | 1029.74734 | 0.39169941 | 0.10430363 | 3.43E-05 | 0.00149515 | Up | 228012 | tousled-like kinase 1 [Source:MGI Symbol;Acc:MGI:2441683] | Tlk1 | protein_coding |
| ENSMUSG00000042010 | 5106.68204 | 0.67900119 | 0.41669828 | 0.00429914 | 0.03415134 | Up | 100705 | acetyl-Coenzyme A carboxylase beta [Source:MGI Symbol;Acc:MGI:2140940] | Acacb | protein_coding |
| ENSMUSG00000042043 | 712.603525 | -0.5312664 | 0.20458728 | 0.0008837 | 0.01238632 | Down | 21371 | tubulin cofactor A [Source:MGI Symbol;Acc:MGI:107549] | Tbca | protein_coding |
| ENSMUSG00000042096 | 83.8877616 | 0.01512121 | 0.19906538 | 4.80E-12 | 1.56E-08 | Up | 13142 | D-amino acid oxidase [Source:MGI Symbol;Acc:MGI:94859] | Dao | protein_coding |
| ENSMUSG00000042116 | 425.307836 | -0.4694116 | 0.15641177 | 0.0003357 | 0.00657966 | Down | 246228 | von Willebrand factor A domain containing 1 [Source:MGI Symbol;Acc:MGI:2179729] | Vwa1 | protein_coding |
| ENSMUSG00000042138 | 278.641647 | 0.51973406 | 0.12980328 | 7.02E-06 | 0.00052284 | Up | 235184 | Myb/SANT-like DNA-binding domain containing 2 [Source:MGI Symbol;Acc:MGI:2384579] | Msantd2 | protein_coding |
| ENSMUSG00000042197 | 490.499959 | 0.35176343 | 0.11164841 | 0.00035296 | 0.00678562 | Up | 98403 | zinc finger protein 451 [Source:MGI Symbol;Acc:MGI:2137896] | Zfp451 | protein_coding |
| ENSMUSG00000042202 | 317.48089 | 0.30677461 | 0.12623907 | 0.00381377 | 0.03154519 | Up | 320541 | solute carrier family 35, member E2 [Source:MGI Symbol;Acc:MGI:2444240] | Slc35e2 | protein_coding |
| ENSMUSG00000042210 | 327.408407 | -0.3218155 | 0.15090608 | 0.00702382 | 0.0475708 | Down | 68644 | abhydrolase domain containing 14A [Source:MGI Symbol;Acc:MGI:1915894] | Abhd14a | protein_coding |
| ENSMUSG00000042211 | 930.744968 | 0.21679958 | 0.08870086 | 0.00665243 | 0.04569502 | Up | 107035 | F-box protein 38 [Source:MGI Symbol;Acc:MGI:2444639] | Fbxo38 | protein_coding |
| ENSMUSG00000042275 | 51.828955 | -0.5702638 | 0.26064431 | 0.00217453 | 0.02191828 | Down | 105083 | pelota mRNA surveillance and ribosome rescue factor [Source:MGI Symbol;Acc:MGI:2145154] | Pelo | protein_coding |
| ENSMUSG00000042298 | 444.536415 | 0.23853234 | 0.06752165 | 0.00016081 | 0.00407555 | Up | 72795 | tetratricopeptide repeat domain 19 [Source:MGI Symbol;Acc:MGI:1920045] | Ttc19 | protein_coding |
| ENSMUSG00000042303 | 289.637044 | -0.2552917 | 0.0947953 | 0.00290362 | 0.02651452 | Down | 105835 | small G protein signaling modulator 3 [Source:MGI Symbol;Acc:MGI:1916329] | Sgsm3 | protein_coding |
| ENSMUSG00000042312 | 764.17648 | -0.622547 | 0.28826089 | 0.00192161 | 0.02024422 | Down | 20196 | S100 calcium binding protein A13 [Source:MGI Symbol;Acc:MGI:109581] | S100a13 | protein_coding |
| ENSMUSG00000042348 | 327.191898 | 0.33861222 | 0.10550173 | 0.00030685 | 0.00617693 | Up | 218639 | ADP-ribosylation factor-like 15 [Source:MGI Symbol;Acc:MGI:2442308] | Arl15 | protein_coding |
| ENSMUSG00000042350 | 825.774151 | 0.35415872 | 0.1433647 | 0.00257727 | 0.02446978 | Up | 68497 | apoptosis resistant E3 ubiquitin protein ligase 1 [Source:MGI Symbol;Acc:MGI:1915747] | Arel1 | protein_coding |
| ENSMUSG00000042380 | 301.406489 | -0.4547591 | 0.14933898 | 0.0003048 | 0.00615863 | Down | 80284 | small integral membrane protein 12 [Source:MGI Symbol;Acc:MGI:1933141] | Smim12 | protein_coding |
| ENSMUSG00000042390 | 1087.36469 | 0.42523978 | 0.10292922 | 6.46E-06 | 0.00049005 | Up | 229542 | GATA zinc finger domain containing 2B [Source:MGI Symbol;Acc:MGI:2443225] | Gatad2b | protein_coding |
| ENSMUSG00000042401 | 401.498967 | -1.0880521 | 0.57058047 | 0.00172212 | 0.01881773 | Down | 72832 | cartilage acidic protein 1 [Source:MGI Symbol;Acc:MGI:1920082] | Crtac1 | protein_coding |
| ENSMUSG00000042419 | 145.25445 | -0.435225 | 0.190354 | 0.00270795 | 0.02534023 | Down | 18038 | nuclear factor of kappa light polypeptide gene enhancer in B cells inhibitor like 1 [Source:MGI Symbol;Acc:MGI:1340031] | Nfkbil1 | protein_coding |
| ENSMUSG00000042444 | 1313.36499 | 0.36675207 | 0.11450874 | 0.00027125 | 0.00570043 | Up | 235461 | MINDY lysine 48 deubiquitinase 2 [Source:MGI Symbol;Acc:MGI:2443086] | Mindy2 | protein_coding |
| ENSMUSG00000042446 | 673.365978 | 0.39497056 | 0.10478386 | 3.04E-05 | 0.00143331 | Up | 67785 | zinc finger, MYM-type 4 [Source:MGI Symbol;Acc:MGI:1915035] | Zmym4 | protein_coding |
| ENSMUSG00000042462 | 157.377983 | -0.5318522 | 0.19537328 | 0.00060074 | 0.00951179 | Down | 66422 | dCTP pyrophosphatase 1 [Source:MGI Symbol;Acc:MGI:1913672] | Dctpp1 | protein_coding |
| ENSMUSG00000042485 | 428.333645 | -1.0306648 | 0.22770608 | 3.74E-07 | 7.27E-05 | Down | 66175 | musculoskeletal, embryonic nuclear protein 1 [Source:MGI Symbol;Acc:MGI:1913425] | Mustn1 | protein_coding |
| ENSMUSG00000042492 | 951.45355 | -0.3114787 | 0.11299842 | 0.00145009 | 0.01691077 | Down | 68449 | TBC1 domain family, member 10b [Source:MGI Symbol;Acc:MGI:1915699] | Tbc1d10b | protein_coding |
| ENSMUSG00000042508 | 567.557865 | 0.39329473 | 0.13274916 | 0.00053497 | 0.00880853 | Up | 23857 | cyclin D binding myb like transcription factor 1 [Source:MGI Symbol;Acc:MGI:1344415] | Dmtf1 | protein_coding |
| ENSMUSG00000042532 | 246.734979 | -0.6452384 | 0.33877563 | 0.00299463 | 0.02701153 | Down | 71146 | golgi autoantigen, golgin subfamily a, 7B [Source:MGI Symbol;Acc:MGI:1918396] | Golga7b | protein_coding |
| ENSMUSG00000042557 | 797.190948 | 0.32790954 | 0.10865035 | 0.00061003 | 0.00960103 | Up | 20466 | transcriptional regulator, SIN3A (yeast) [Source:MGI Symbol;Acc:MGI:107157] | Sin3a | protein_coding |
| ENSMUSG00000042558 | 402.561642 | -0.4154052 | 0.12083919 | 9.49E-05 | 0.00281444 | Down | 100206 | ADP-ribosylserine hydrolase [Source:MGI Symbol;Acc:MGI:2140364] | Adprs | protein_coding |
| ENSMUSG00000042579 | 102.265825 | 0.4772361 | 0.20464059 | 0.00206817 | 0.02122294 | Up | 74034 | RIKEN cDNA 4632404H12 gene [Source:MGI Symbol;Acc:MGI:1921284] | 4632404H12Rik | lncRNA |
| ENSMUSG00000042595 | 212.51825 | 0.48215142 | 0.11595138 | 4.36E-06 | 0.00037622 | Up | 245622 | family with sequence similarity 199, X-linked [Source:MGI Symbol;Acc:MGI:2384304] | Fam199x | protein_coding |
| ENSMUSG00000042599 | 1000.87074 | 0.39372689 | 0.16534756 | 0.00244108 | 0.0237031 | Up | 338523 | lysine (K)-specific demethylase 7A [Source:MGI Symbol;Acc:MGI:2443388] | Kdm7a | protein_coding |
| ENSMUSG00000042622 | 226.359223 | -0.4307634 | 0.23611328 | 0.00703148 | 0.04757453 | Down | 17133 | v-maf musculoaponeurotic fibrosarcoma oncogene family, protein F (avian) [Source:MGI Symbol;Acc:MGI:96910] | Maff | protein_coding |
| ENSMUSG00000042660 | 189.846157 | -0.3208484 | 0.14182338 | 0.0050484 | 0.03798203 | Down | 67936 | WD repeat domain 55 [Source:MGI Symbol;Acc:MGI:1915186] | Wdr55 | protein_coding |
| ENSMUSG00000042662 | 18.9517528 | -0.2015767 | 0.38171409 | 0.00376209 | 0.03130672 | Down | 252864 | dual specificity phosphatase-like 15 [Source:MGI Symbol;Acc:MGI:1934928] | Dusp15 | protein_coding |
| ENSMUSG00000042675 | 1567.89435 | -0.4509756 | 0.14865964 | 0.00029913 | 0.00610744 | Down | 66090 | yippee like 3 [Source:MGI Symbol;Acc:MGI:1913340] | Ypel3 | protein_coding |
| ENSMUSG00000042699 | 2236.74899 | 0.38192705 | 0.12468276 | 0.00041043 | 0.00738372 | Up | 13211 | DEAH (Asp-Glu-Ala-His) box polypeptide 9 [Source:MGI Symbol;Acc:MGI:108177] | Dhx9 | protein_coding |
| ENSMUSG00000042759 | 238.753321 | -0.8367041 | 0.28597507 | 0.00018766 | 0.00452298 | Down | 171504 | apolipoprotein B receptor [Source:MGI Symbol;Acc:MGI:2176230] | Apobr | protein_coding |
| ENSMUSG00000042770 | 2245.71849 | -0.4117005 | 0.1605249 | 0.00148088 | 0.01711625 | Down | 15199 | heme binding protein 1 [Source:MGI Symbol;Acc:MGI:1333880] | Hebp1 | protein_coding |
| ENSMUSG00000042772 | 934.329275 | 0.30140176 | 0.08648529 | 0.00013678 | 0.00363059 | Up | 226517 | SMG7 nonsense mediated mRNA decay factor [Source:MGI Symbol;Acc:MGI:2682334] | Smg7 | protein_coding |

| | | | | | | | | | | |
|---|---|---|---|---|---|---|---|---|---|---|
| ENSMUSG00000042807 | 114.178269 | 0.53146239 | 0.22852754 | 0.00175476 | 0.01901671 | Up | 329152 | HECT, C2 and WW domain containing E3 ubiquitin protein ligase 2 [Source:MGI Symbol;Acc:MGI:2685817] | Hecw2 | protein_coding |
| ENSMUSG00000042831 | 281.992291 | -0.3933549 | 0.15928275 | 0.00222319 | 0.02225243 | Down | 233065 | alkB homolog 6 [Source:MGI Symbol;Acc:MGI:2142037] | Alkbh6 | protein_coding |
| ENSMUSG00000042903 | 918.367291 | -0.3247203 | 0.13057952 | 0.00291231 | 0.02655961 | Down | 54601 | forkhead box O4 [Source:MGI Symbol;Acc:MGI:1891915] | Foxo4 | protein_coding |
| ENSMUSG00000043029 | 21.2149512 | 0.93230546 | 0.59658575 | 0.00354403 | 0.03011126 | Up | 246788 | transient receptor potential cation channel, subfamily V, member 3 [Source:MGI Symbol;Acc:MGI:2181407] | Trpv3 | protein_coding |
| ENSMUSG00000043090 | 293.161593 | 0.35835063 | 0.1332701 | 0.00139659 | 0.01656035 | Up | 330788 | zinc finger protein 866 [Source:MGI Symbol;Acc:MGI:3584369] | Zfp866 | protein_coding |
| ENSMUSG00000043122 | 4481.00077 | 0.37767101 | 0.18731952 | 0.00666369 | 0.04571935 | Up | 319942 | RIKEN cDNA A530016L24 gene [Source:MGI Symbol;Acc:MGI:2443020] | A530016L24Rik | protein_coding |
| ENSMUSG00000043154 | 561.791127 | 0.63071583 | 0.17511706 | 2.70E-05 | 0.00131199 | Up | 235542 | protein phosphatase 2, regulatory subunit B'', alpha [Source:MGI Symbol;Acc:MGI:2442104] | Ppp2r3a | protein_coding |
| ENSMUSG00000043155 | 28.4830949 | -0.6296819 | 0.44668464 | 0.00679649 | 0.04642932 | Down | 242642 | 4-hydroxyphenylpyruvate dioxygenase-like [Source:MGI Symbol;Acc:MGI:2444646] | Hpdl | protein_coding |
| ENSMUSG00000043207 | 1042.4797 | 0.30285655 | 0.11839058 | 0.00283818 | 0.02609298 | Up | 230709 | zinc metallopeptidase, STE24 [Source:MGI Symbol;Acc:MGI:1890508] | Zmpste24 | protein_coding |
| ENSMUSG00000043445 | 685.722914 | -0.6156939 | 0.14371344 | 1.71E-06 | 0.00020222 | Down | 67078 | phosphoglycolate phosphatase [Source:MGI Symbol;Acc:MGI:1914328] | Pgp | protein_coding |
| ENSMUSG00000043866 | 1159.07788 | -0.5185631 | 0.18167607 | 0.0004274 | 0.00754266 | Down | 24075 | TATA-box binding protein associated factor 10 [Source:MGI Symbol;Acc:MGI:1346320] | Taf10 | protein_coding |
| ENSMUSG00000043998 | 939.334384 | -0.4021823 | 0.1011472 | 1.05E-05 | 0.00072217 | Down | 217664 | mannoside acetylglucosaminyltransferase 2 [Source:MGI Symbol;Acc:MGI:2384966] | Mgat2 | protein_coding |
| ENSMUSG00000044037 | 398.865282 | 0.55372753 | 0.26491356 | 0.00277693 | 0.02571326 | Up | 235633 | ALS2 C-terminal like [Source:MGI Symbol;Acc:MGI:2447532] | Als2cl | protein_coding |
| ENSMUSG00000044134 | 148.65058 | -0.4957719 | 0.18016816 | 0.00062534 | 0.00975861 | Down | 231717 | PH domain containing endocytic trafficking adaptor 1 [Source:MGI Symbol;Acc:MGI:2442708] | Pheta1 | protein_coding |
| ENSMUSG00000044147 | 868.627576 | -0.4523334 | 0.14073232 | 0.00016149 | 0.00408621 | Down | 11845 | ADP-ribosylation factor 6 [Source:MGI Symbol;Acc:MGI:99435] | Arf6 | protein_coding |
| ENSMUSG00000044303 | 21.2011645 | -1.2378674 | 0.57334444 | 0.00101861 | 0.01344218 | Down | 12578 | cyclin dependent kinase inhibitor 2A [Source:MGI Symbol;Acc:MGI:104738] | Cdkn2a | protein_coding |
| ENSMUSG00000044308 | 2832.66106 | 0.35140172 | 0.1474657 | 0.00332316 | 0.02890559 | Up | 68795 | ubiquitin protein ligase E3 component n-recognin 3 [Source:MGI Symbol;Acc:MGI:1861100] | Ubr3 | protein_coding |
| ENSMUSG00000044349 | 2446.64339 | 0.64689307 | 0.20914478 | 0.0001266 | 0.00341634 | Up | 319317 | small nucleolar RNA host gene 11 [Source:MGI Symbol;Acc:MGI:2441845] | Snhg11 | protein_coding |
| ENSMUSG00000044477 | 4444.17826 | -0.2250116 | 0.09122739 | 0.00601784 | 0.0425969 | Down | 21769 | zinc finger, AN1-type domain 3 [Source:MGI Symbol;Acc:MGI:1096572] | Zfand3 | protein_coding |
| ENSMUSG00000044496 | 1050.59119 | -0.386875 | 0.12182685 | 0.00024586 | 0.0053899 | Down | 77034 | RIKEN cDNA 2510039O18 gene [Source:MGI Symbol;Acc:MGI:1924284] | 2510039O18Rik | protein_coding |
| ENSMUSG00000044501 | 191.097605 | 0.407594 | 0.18778033 | 0.00405631 | 0.03286198 | Up | 224598 | zinc finger protein 758 [Source:MGI Symbol;Acc:MGI:2385044] | Zfp758 | protein_coding |
| ENSMUSG00000044502 | 863.466128 | -0.435683 | 0.11160955 | 1.43E-05 | 0.00087877 | Down | 69556 | biorientation of chromosomes in cell division 1 [Source:MGI Symbol;Acc:MGI:1916806] | Bod1 | protein_coding |
| ENSMUSG00000044667 | 65.7792399 | -0.2955812 | 0.8164261 | 0.00459961 | 0.03571736 | Down | 229791 | phospholipid phosphatase related 4 [Source:MGI Symbol;Acc:MGI:106530] | Plppr4 | protein_coding |
| ENSMUSG00000044694 | 158.549445 | 0.56495814 | 0.31755777 | 0.00473106 | 0.03639015 | Up | NA | RIKEN cDNA 2010007H06 gene [Source:MGI Symbol;Acc:MGI:1917099] | 2010007H06Rik | lncRNA |
| ENSMUSG00000044709 | 628.13973 | -0.7138032 | 0.17749008 | 4.38E-06 | 0.00037622 | Down | 69731 | gem nuclear organelle associated protein 7 [Source:MGI Symbol;Acc:MGI:1916981] | Gemin7 | protein_coding |
| ENSMUSG00000044791 | 1268.5009 | 0.342513 | 0.15171939 | 0.00479347 | 0.03674841 | Up | 235626 | SET domain containing 2 [Source:MGI Symbol;Acc:MGI:1918177] | Setd2 | protein_coding |
| ENSMUSG00000044807 | 78.3859845 | 0.57681021 | 0.2014721 | 0.00036643 | 0.00693792 | Up | 30944 | zinc finger protein 354C [Source:MGI Symbol;Acc:MGI:1353621] | Zfp354c | protein_coding |
| ENSMUSG00000044813 | 344.31914 | -0.3349181 | 0.13466238 | 0.00277068 | 0.02570516 | Down | 230126 | src homology 2 domain-containing transforming protein B [Source:MGI Symbol;Acc:MGI:98294] | Shb | protein_coding |
| ENSMUSG00000044854 | 75.6345618 | -1.1026629 | 0.2687093 | 2.26E-06 | 0.00024959 | Down | 73363 | RIKEN cDNA 1700056E22 gene [Source:MGI Symbol;Acc:MGI:1920613] | 1700056E22Rik | protein_coding |
| ENSMUSG00000044857 | 863.691903 | -0.3589221 | 0.14909125 | 0.00280557 | 0.02591044 | Down | 224640 | LEM domain containing 2 [Source:MGI Symbol;Acc:MGI:2385045] | Lemd2 | protein_coding |
| ENSMUSG00000044894 | 2573.86165 | -0.5811507 | 0.26258715 | 0.00203454 | 0.02105162 | Down | 22272 | ubiquinol-cytochrome c reductase, complex III subunit VII [Source:MGI Symbol;Acc:MGI:107807] | Uqcrq | protein_coding |
| ENSMUSG00000044950 | 274.939123 | 0.4145128 | 0.17517518 | 0.00249896 | 0.02401966 | Up | 70802 | PWWP domain containing 2A [Source:MGI Symbol;Acc:MGI:1918052] | Pwwp2a | protein_coding |
| ENSMUSG00000045038 | 1019.88294 | 0.471829 | 0.25822025 | 0.00642711 | 0.04461897 | Up | 18754 | protein kinase C, epsilon [Source:MGI Symbol;Acc:MGI:97599] | Prkce | protein_coding |
| ENSMUSG00000045055 | 8.9632845 | -0.8825515 | 0.56405057 | 0.00404991 | 0.03286198 | Down | NA | ribosomal protein SA, pseudogene 2 [Source:MGI Symbol;Acc:MGI:3643356] | Rpsa-ps2 | processed_pseudogene |
| ENSMUSG00000045107 | 132.940242 | -0.8105404 | 0.24444907 | 5.51E-05 | 0.00197972 | Down | 67509 | SAYSVFN motif domain containing 1 [Source:MGI Symbol;Acc:MGI:1914759] | Saysd1 | protein_coding |
| ENSMUSG00000045128 | 5900.53021 | -0.3946973 | 0.19073042 | 0.00554073 | 0.04043535 | Down | 76808 | ribosomal protein L18A [Source:MGI Symbol;Acc:MGI:1924058] | Rpl18a | protein_coding |
| ENSMUSG00000045160 | 841.180712 | -0.3681149 | 0.16209308 | 0.00393395 | 0.03227629 | Down | 78653 | bolA-like 3 (E. coli) [Source:MGI Symbol;Acc:MGI:1925903] | Bola3 | protein_coding |
| ENSMUSG00000045176 | 189.12922 | -0.7511965 | 0.25573626 | 0.00020281 | 0.0047067 | Down | 71923 | BLOC-1 related complex subunit 6 [Source:MGI Symbol;Acc:MGI:1919173] | Borcs6 | protein_coding |
| ENSMUSG00000045210 | 809.430288 | 0.33952949 | 0.10755921 | 0.00037233 | 0.00700065 | Up | 70675 | valosin containing protein (p97)/p47 complex interacting protein 1 [Source:MGI Symbol;Acc:MGI:1917925] | Vcpip1 | protein_coding |
| ENSMUSG00000045503 | 521.349644 | -0.3807886 | 0.15844103 | 0.00264411 | 0.02487182 | Down | 66460 | SYS1 Golgi-localized integral membrane protein homolog (S. cerevisiae) [Source:MGI Symbol;Acc:MGI:1913710] | Sys1 | protein_coding |
| ENSMUSG00000045555 | 128.243238 | -0.6439415 | 0.22369291 | 0.00028641 | 0.00592713 | Down | 327747 | methyltransferase like 24 [Source:MGI Symbol;Acc:MGI:3045338] | Mettl24 | protein_coding |
| ENSMUSG00000045594 | 904.783554 | -0.4163917 | 0.13488502 | 0.0002987 | 0.00610744 | Down | 12091 | galactosidase, beta 1 [Source:MGI Symbol;Acc:MGI:88151] | Glb1 | protein_coding |
| ENSMUSG00000045636 | 2970.71724 | 0.61403024 | 0.15724734 | 1.07E-05 | 0.00072429 | Up | 102103 | mitochondrial tumor suppressor 1 [Source:MGI Symbol;Acc:MGI:2142572] | Mtus1 | protein_coding |
| ENSMUSG00000045752 | 225.661567 | -0.5763557 | 0.27623964 | 0.00259106 | 0.02457199 | Down | 56844 | tumor-suppressing subchromosomal transferable fragment 4 [Source:MGI Symbol;Acc:MGI:1861712] | Tssc4 | protein_coding |
| ENSMUSG00000045763 | 218.240562 | -0.8209621 | 0.31625082 | 0.00048403 | 0.00819824 | Down | 70350 | brain abundant, membrane attached signal protein 1 [Source:MGI Symbol;Acc:MGI:1917600] | Basp1 | protein_coding |
| ENSMUSG00000045767 | 1539.36626 | 0.24265657 | 0.09551783 | 0.00430813 | 0.03418937 | Up | 78521 | RIKEN cDNA B230219D22 gene [Source:MGI Symbol;Acc:MGI:1925771] | B230219D22Rik | protein_coding |
| ENSMUSG00000045948 | 421.709943 | -0.8626466 | 0.22787452 | 9.54E-06 | 0.00067695 | Down | 24030 | mitochondrial ribosomal protein S12 [Source:MGI Symbol;Acc:MGI:1346333] | Mrps12 | protein_coding |
| ENSMUSG00000046034 | 369.578888 | -0.3514528 | 0.15382289 | 0.00405996 | 0.03286198 | Down | 432940 | OTU deubiquitinase with linear linkage specificity [Source:MGI Symbol;Acc:MGI:3577015] | Otulin | protein_coding |
| ENSMUSG00000046056 | 581.163885 | -1.1517134 | 0.28523239 | 2.69E-06 | 0.0002824 | Down | 282619 | suprabasin [Source:MGI Symbol;Acc:MGI:2446326] | Sbsn | protein_coding |
| ENSMUSG00000046058 | 272.254147 | -0.6490762 | 0.17833445 | 2.28E-05 | 0.00116956 | Down | 386655 | EP300 interacting inhibitor of differentiation 2 [Source:MGI Symbol;Acc:MGI:2681174] | Eid2 | protein_coding |
| ENSMUSG00000046062 | 1734.76427 | 0.48579253 | 0.12863121 | 2.12E-05 | 0.00111477 | Up | 108954 | protein phosphatase 1, regulatory subunit 15B [Source:MGI Symbol;Acc:MGI:2444211] | Ppp1r15b | protein_coding |

| | | | | | | | | | | |
|---|---|---|---|---|---|---|---|---|---|---|
| ENSMUSG00000046070 | 1344.92703 | -0.5119154 | 0.28424218 | 0.00533556 | 0.0394522 | Down | 16005 | insulin-like growth factor binding protein, acid labile subunit [Source:MGI Symbol;Acc:MGI:107973] | Igfals | protein_coding |
| ENSMUSG00000046096 | 808.440168 | 0.22935651 | 0.07499241 | 0.00097262 | 0.01318349 | Up | 233812 | modulator of smoothened [Source:MGI Symbol;Acc:MGI:2446240] | Mosmo | protein_coding |
| ENSMUSG00000046139 | 540.185952 | 0.53903098 | 0.18125978 | 0.00029043 | 0.00598739 | Up | 225929 | protein associated with topoisomerase II homolog 1 (yeast) [Source:MGI Symbol;Acc:MGI:2147679] | Patl1 | protein_coding |
| ENSMUSG00000046168 | 11.6374539 | 1.16611849 | 0.60942631 | 0.00189384 | 0.02001659 | Up | 328424 | potassium channel regulator [Source:MGI Symbol;Acc:MGI:2685591] | Kcnrg | protein_coding |
| ENSMUSG00000046229 | 1057.02425 | -1.2473905 | 0.31627263 | 3.81E-06 | 0.00036162 | Down | 19018 | SCAN domain-containing 1 [Source:MGI Symbol;Acc:MGI:1343132] | Scand1 | protein_coding |
| ENSMUSG00000046311 | 349.06362 | 0.48023451 | 0.16403763 | 0.00040362 | 0.00732601 | Up | 22720 | zinc finger protein 62 [Source:MGI Symbol;Acc:MGI:99662] | Zfp62 | protein_coding |
| ENSMUSG00000046351 | 321.907928 | 0.30506235 | 0.10927661 | 0.00142525 | 0.01677221 | Up | 218100 | zinc finger protein 322A [Source:MGI Symbol;Acc:MGI:2442566] | Zfp322a | protein_coding |
| ENSMUSG00000046364 | 4613.88714 | -0.4824805 | 0.18087525 | 0.00083846 | 0.01191802 | Down | 26451 | ribosomal protein L27A [Source:MGI Symbol;Acc:MGI:1347076] | Rpl27a | protein_coding |
| ENSMUSG00000046516 | 127.837998 | 0.67007821 | 0.1890155 | 3.08E-05 | 0.00143738 | Up | 12856 | cytochrome c oxidase assembly protein 17, copper chaperone [Source:MGI Symbol;Acc:MGI:1333806] | Cox17 | protein_coding |
| ENSMUSG00000046718 | 648.264425 | -0.4546376 | 0.25916331 | 0.00749404 | 0.04964955 | Down | 69550 | bone marrow stromal cell antigen 2 [Source:MGI Symbol;Acc:MGI:1916800] | Bst2 | protein_coding |
| ENSMUSG00000046721 | 32.1520401 | -0.834863 | 0.36807258 | 0.00107764 | 0.01394925 | Down | NA | ribosomal protein L14, pseudogene 1 [Source:MGI Symbol;Acc:MGI:3710579] | Rpl14-ps1 | processed_pseudogene |
| ENSMUSG00000046727 | 691.88931 | -0.8784672 | 0.26667084 | 5.37E-05 | 0.00194658 | Down | 66060 | cysteine-rich transmembrane module containing 1 [Source:MGI Symbol;Acc:MGI:1913310] | Cystm1 | protein_coding |
| ENSMUSG00000046785 | 1018.76448 | 0.48055828 | 0.18313571 | 0.00105398 | 0.01374147 | Up | 77781 | EPM2A (laforin) interacting protein 1 [Source:MGI Symbol;Acc:MGI:1925031] | Epm2aip1 | protein_coding |
| ENSMUSG00000046798 | 441.428882 | 0.46465369 | 0.17118948 | 0.00078909 | 0.01150695 | Up | 64945 | claudin 12 [Source:MGI Symbol;Acc:MGI:1929288] | Cldn12 | protein_coding |
| ENSMUSG00000046808 | 573.20243 | 0.35419036 | 0.1321899 | 0.001468 | 0.01700975 | Up | 231287 | ATPase, class V, type 10D [Source:MGI Symbol;Acc:MGI:2450125] | Atp10d | protein_coding |
| ENSMUSG00000046822 | 754.096323 | -0.5327827 | 0.14559886 | 2.80E-05 | 0.00134946 | Down | 106947 | solute carrier family 39 (zinc transporter), member 3 [Source:MGI Symbol;Acc:MGI:2147269] | Slc39a3 | protein_coding |
| ENSMUSG00000046836 | 1057.70575 | 0.23700537 | 0.06338372 | 8.27E-05 | 0.00259338 | Up | 71678 | BRO1 domain and CAAX motif containing [Source:MGI Symbol;Acc:MGI:1918928] | Brox | protein_coding |
| ENSMUSG00000046865 | 456.347729 | -0.4012459 | 0.15684647 | 0.00160743 | 0.01797162 | Down | 14113 | fibrillarin [Source:MGI Symbol;Acc:MGI:95486] | Fbl | protein_coding |
| ENSMUSG00000046873 | 350.517847 | 0.39197212 | 0.08770261 | 1.53E-06 | 0.00018939 | Up | 270669 | membrane-bound transcription factor peptidase, site 2 [Source:MGI Symbol;Acc:MGI:2444506] | Mbtps2 | protein_coding |
| ENSMUSG00000046876 | 576.186455 | 0.44597353 | 0.15932747 | 0.00068588 | 0.01047196 | Up | 20238 | ataxin 1 [Source:MGI Symbol;Acc:MGI:104783] | Atxn1 | protein_coding |
| ENSMUSG00000046985 | 1067.64702 | 0.4790986 | 0.16964554 | 0.00056788 | 0.00914292 | Up | 231225 | transmembrane anterior posterior transformation 1 [Source:MGI Symbol;Acc:MGI:2683537] | Tapt1 | protein_coding |
| ENSMUSG00000047021 | 89.4208203 | 0.0097601 | 0.19856473 | 5.49E-08 | 1.78E-05 | Up | 241116 | cilia and flagella associated protein 65 [Source:MGI Symbol;Acc:MGI:2444274] | Cfap65 | protein_coding |
| ENSMUSG00000047022 | 96.8632824 | 0.3542664 | 0.16200949 | 0.00514812 | 0.03841138 | Up | 73490 | mirror-image polydactyly 1 [Source:MGI Symbol;Acc:MGI:1920740] | Mipol1 | protein_coding |
| ENSMUSG00000047036 | 1376.78189 | 0.32203857 | 0.13858962 | 0.00437493 | 0.03446687 | Up | 235682 | zinc finger protein 445 [Source:MGI Symbol;Acc:MGI:2143340] | Zfp445 | protein_coding |
| ENSMUSG00000047126 | 7737.16475 | 0.36874312 | 0.09877179 | 4.00E-05 | 0.00163748 | Up | 67300 | clathrin, heavy polypeptide (Hc) [Source:MGI Symbol;Acc:MGI:2388633] | Cltc | protein_coding |
| ENSMUSG00000047141 | 343.022963 | 0.57937892 | 0.17895001 | 0.00011134 | 0.0031184 | Up | 72020 | zinc finger protein 654 [Source:MGI Symbol;Acc:MGI:1919270] | Zfp654 | protein_coding |
| ENSMUSG00000047182 | 1415.25922 | -0.4591769 | 0.1552692 | 0.00036151 | 0.00687161 | Down | 16369 | insulin receptor substrate 3 [Source:MGI Symbol;Acc:MGI:1194882] | Irs3 | protein_coding |
| ENSMUSG00000047213 | 1576.99422 | 0.40686643 | 0.09457207 | 3.15E-06 | 0.00031579 | Up | 229096 | YTH N6-methyladenosine RNA binding protein 3 [Source:MGI Symbol;Acc:MGI:1918850] | Ythdf3 | protein_coding |
| ENSMUSG00000047238 | 145.233429 | -0.4033575 | 0.20580101 | 0.00640115 | 0.04451487 | Down | 75625 | MAGE family member H1 [Source:MGI Symbol;Acc:MGI:1922875] | Mageh1 | protein_coding |
| ENSMUSG00000047246 | 399.035097 | -0.4215076 | 0.19418393 | 0.00372781 | 0.03113389 | Down | 319179 | H2B clustered histone 6 [Source:MGI Symbol;Acc:MGI:2448380] | H2bc6 | protein_coding |
| ENSMUSG00000047379 | 798.392839 | -0.4732007 | 0.13899067 | 6.86E-05 | 0.00226099 | Down | 108902 | beta-1,4-glucuronyltransferase 1 [Source:MGI Symbol;Acc:MGI:1919680] | B4gat1 | protein_coding |
| ENSMUSG00000047412 | 610.116109 | 0.43511851 | 0.10383371 | 4.04E-06 | 0.00036296 | Up | 235132 | zinc finger and BTB domain containing 44 [Source:MGI Symbol;Acc:MGI:1925123] | Zbtb44 | protein_coding |
| ENSMUSG00000047423 | 619.315612 | -0.2863754 | 0.11223014 | 0.0030852 | 0.0275834 | Down | 107242 | expressed sequence AI837181 [Source:MGI Symbol;Acc:MGI:2147598] | AI837181 | protein_coding |
| ENSMUSG00000047446 | 3619.12098 | 0.53379251 | 0.16824695 | 0.00016499 | 0.00412988 | Up | 11861 | ADP-ribosylation factor-like 4A [Source:MGI Symbol;Acc:MGI:99437] | Arl4a | protein_coding |
| ENSMUSG00000047454 | 900.912252 | 0.33733529 | 0.12109038 | 0.00124435 | 0.01525618 | Up | 268566 | gephyrin [Source:MGI Symbol;Acc:MGI:109602] | Gphn | protein_coding |
| ENSMUSG00000047459 | 2310.69712 | -0.2977974 | 0.13663432 | 0.00725446 | 0.04855734 | Down | 67068 | dynein light chain roadblock-type 1 [Source:MGI Symbol;Acc:MGI:1914318] | Dynlrb1 | protein_coding |
| ENSMUSG00000047466 | 241.314216 | 0.44121452 | 0.19115805 | 0.00252507 | 0.02417189 | Up | 212163 | RIKEN cDNA 8030462N17 gene [Source:MGI Symbol;Acc:MGI:2444951] | 8030462N17Rik | protein_coding |
| ENSMUSG00000047547 | 1329.46802 | -0.8399605 | 0.22001124 | 8.36E-06 | 0.00060539 | Down | 74325 | clathrin, light polypeptide (Lcb) [Source:MGI Symbol;Acc:MGI:1921575] | Cltb | protein_coding |
| ENSMUSG00000047557 | 203.82349 | -0.4189393 | 0.19631195 | 0.00399878 | 0.0325942 | Down | 17035 | latexin [Source:MGI Symbol;Acc:MGI:107633] | Lxn | protein_coding |
| ENSMUSG00000047613 | 259.042321 | -0.3939658 | 0.16678704 | 0.00261445 | 0.02472158 | Down | 97159 | RIKEN cDNA A430005L14 gene [Source:MGI Symbol;Acc:MGI:2140680] | A430005L14Rik | protein_coding |
| ENSMUSG00000047648 | 275.481611 | 0.3213752 | 0.15142381 | 0.00727378 | 0.04864656 | Up | 71865 | F-box protein 30 [Source:MGI Symbol;Acc:MGI:1919115] | Fbxo30 | protein_coding |
| ENSMUSG00000047649 | 227.230236 | -0.3335943 | 0.15254231 | 0.00577308 | 0.04148535 | Down | 70333 | CD3E antigen, epsilon polypeptide associated protein [Source:MGI Symbol;Acc:MGI:1917583] | Cd3eap | protein_coding |
| ENSMUSG00000047675 | 6262.26503 | -0.4390689 | 0.16734822 | 0.00122545 | 0.0151441 | Down | 20116 | ribosomal protein S8 [Source:MGI Symbol;Acc:MGI:98166] | Rps8 | protein_coding |
| ENSMUSG00000047694 | 770.868359 | 0.29063222 | 0.0908872 | 0.00042134 | 0.0074724 | Up | 77929 | Yip1 domain family, member 6 [Source:MGI Symbol;Acc:MGI:1925179] | Yipf6 | protein_coding |
| ENSMUSG00000047721 | 336.259058 | -0.5758927 | 0.25325554 | 0.00172422 | 0.01881773 | Down | 66162 | bolA-like 2 (E. coli) [Source:MGI Symbol;Acc:MGI:1913412] | Bola2 | protein_coding |
| ENSMUSG00000047731 | 2205.91826 | -0.225514 | 0.09046076 | 0.00559684 | 0.04066223 | Down | 226178 | WW domain binding protein 1 like [Source:MGI Symbol;Acc:MGI:107577] | Wbp1l | protein_coding |
| ENSMUSG00000047843 | 1281.51497 | -0.4231777 | 0.15100091 | 0.00071819 | 0.01086315 | Down | 55950 | brain protein I3 [Source:MGI Symbol;Acc:MGI:1933174] | Bri3 | protein_coding |
| ENSMUSG00000047909 | 648.541389 | 0.41314316 | 0.14189395 | 0.00055645 | 0.00900355 | Up | 320816 | ankyrin repeat domain 16 [Source:MGI Symbol;Acc:MGI:2444796] | Ankrd16 | protein_coding |
| ENSMUSG00000047963 | 328.680957 | -0.5176483 | 0.21734957 | 0.00156687 | 0.01770086 | Down | 52331 | starch binding domain 1 [Source:MGI Symbol;Acc:MGI:1261768] | Stbd1 | protein_coding |
| ENSMUSG00000048047 | 381.943064 | 0.39766024 | 0.1317337 | 0.00041018 | 0.00738372 | Up | 56805 | zinc finger and BTB domain containing 33 [Source:MGI Symbol;Acc:MGI:1927290] | Zbtb33 | protein_coding |
| ENSMUSG00000048058 | 292.364264 | 0.84430236 | 0.1594238 | 8.32E-09 | 5.41E-06 | Up | 241576 | low density lipoprotein receptor class A domain containing 3 [Source:MGI Symbol;Acc:MGI:2138856] | Ldlrad3 | protein_coding |
| ENSMUSG00000048076 | 5471.02927 | -0.2870967 | 0.07882941 | 9.64E-05 | 0.00284772 | Down | 11840 | ADP-ribosylation factor 1 [Source:MGI Symbol;Acc:MGI:99431] | Arf1 | protein_coding |
| ENSMUSG00000048100 | 478.702486 | -0.4785211 | 0.21418147 | 0.00254505 | 0.02429407 | Down | 99730 | TATA-box binding protein associated factor 13 [Source:MGI Symbol;Acc:MGI:1913500] | Taf13 | protein_coding |

| | | | | | | | | | | |
|---|---|---|---|---|---|---|---|---|---|---|
| ENSMUSG00000048109 | 372.133738 | 0.33195426 | 0.12313077 | 0.00156374 | 0.01769928 | Up | 229700 | RNA binding motif protein 15 [Source:MGI Symbol;Acc:MGI:2443205] | Rbm15 | protein_coding |
| ENSMUSG00000048118 | 517.208145 | 0.27891188 | 0.11949012 | 0.0058858 | 0.04191793 | Up | 238247 | AT rich interactive domain 4A (RBP1-like) [Source:MGI Symbol;Acc:MGI:2444354] | Arid4a | protein_coding |
| ENSMUSG00000048163 | 449.755618 | -0.4709378 | 0.24544125 | 0.00509854 | 0.03827231 | Down | 20345 | selectin, platelet (p-selectin) ligand [Source:MGI Symbol;Acc:MGI:106689] | Selplg | protein_coding |
| ENSMUSG00000048170 | 825.663408 | 0.2587906 | 0.09627023 | 0.00257268 | 0.02445731 | Up | 210711 | minichromosome maintenance complex binding protein [Source:MGI Symbol;Acc:MGI:1920977] | Mcmbp | protein_coding |
| ENSMUSG00000048200 | 221.907564 | -0.4918839 | 0.26278382 | 0.0047381 | 0.03642708 | Down | 213573 | calcium release activated channel regulator 2B [Source:MGI Symbol;Acc:MGI:2446129] | Cracr2b | protein_coding |
| ENSMUSG00000048271 | 1210.80249 | 0.38255116 | 0.14543713 | 0.0014461 | 0.01688844 | Up | 381626 | RNA binding motif protein 33 [Source:MGI Symbol;Acc:MGI:1919670] | Rbm33 | protein_coding |
| ENSMUSG00000048277 | 1084.67285 | -0.2841857 | 0.11664796 | 0.00426102 | 0.03396482 | Down | 20973 | synaptogyrin 2 [Source:MGI Symbol;Acc:MGI:1328324] | Syngr2 | protein_coding |
| ENSMUSG00000048371 | 403.560328 | 0.45887341 | 0.16634672 | 0.00072439 | 0.01090021 | Up | 382051 | pyruvate dehydrogenase phosphatase catalytic subunit 2 [Source:MGI Symbol;Acc:MGI:1918878] | Pdp2 | protein_coding |
| ENSMUSG00000048490 | 3595.28821 | 0.91608607 | 0.21186372 | 6.87E-07 | 0.00011158 | Up | 268903 | nuclear receptor interacting protein 1 [Source:MGI Symbol;Acc:MGI:1315213] | Nrip1 | protein_coding |
| ENSMUSG00000048546 | 1163.6759 | 0.3857883 | 0.17265019 | 0.00389829 | 0.03208093 | Up | 57259 | transducer of ERBB2, 2 [Source:MGI Symbol;Acc:MGI:1888525] | Tob2 | protein_coding |
| ENSMUSG00000048706 | 361.692023 | -0.5169869 | 0.1703641 | 0.00025573 | 0.00549552 | Down | 52829 | leucine rich adaptor protein 1-like [Source:MGI Symbol;Acc:MGI:106510] | Lurap1l | protein_coding |
| ENSMUSG00000048758 | 4974.40611 | -0.3428234 | 0.14293767 | 0.00318329 | 0.02816589 | Down | 19944 | ribosomal protein L29 [Source:MGI Symbol;Acc:MGI:99687] | Rpl29 | protein_coding |
| ENSMUSG00000048826 | 263.847124 | -0.5210463 | 0.28539428 | 0.00503281 | 0.03795638 | Down | 240025 | dishevelled-binding antagonist of beta-catenin 2 [Source:MGI Symbol;Acc:MGI:1920347] | Dact2 | protein_coding |
| ENSMUSG00000048915 | 497.661686 | 0.38616969 | 0.15200118 | 0.00176543 | 0.01910683 | Up | 13640 | ephrin A5 [Source:MGI Symbol;Acc:MGI:107444] | Efna5 | protein_coding |
| ENSMUSG00000049076 | 1564.63004 | 0.39424254 | 0.11839193 | 0.00015895 | 0.00404733 | Up | 78618 | ArfGAP with coiled-coil, ankyrin repeat and PH domains 2 [Source:MGI Symbol;Acc:MGI:1925868] | Acap2 | protein_coding |
| ENSMUSG00000049090 | 1357.07249 | 0.37134445 | 0.10590808 | 0.00010071 | 0.00291573 | Up | 225791 | zinc binding alcohol dehydrogenase, domain containing 2 [Source:MGI Symbol;Acc:MGI:2444835] | Zadh2 | protein_coding |
| ENSMUSG00000049112 | 2444.5373 | -0.9080962 | 0.48727349 | 0.00215829 | 0.02185892 | Down | 18430 | oxytocin receptor [Source:MGI Symbol;Acc:MGI:109147] | Oxtr | protein_coding |
| ENSMUSG00000049124 | 56.8330101 | -1.0332801 | 0.26298841 | 4.54E-06 | 0.00038448 | Down | NA | predicted gene 8186 [Source:MGI Symbol;Acc:MGI:3643110] | Gm8186 | processed_pseudogene |
| ENSMUSG00000049164 | 479.985581 | 0.30885419 | 0.13815129 | 0.00650842 | 0.04495296 | Up | 72672 | zinc finger protein 518A [Source:MGI Symbol;Acc:MGI:1919922] | Zfp518a | protein_coding |
| ENSMUSG00000049288 | 1463.52643 | -0.321286 | 0.11102469 | 0.00096547 | 0.01311446 | Down | 280411 | Lix1-like [Source:MGI Symbol;Acc:MGI:3036267] | Lix1l | protein_coding |
| ENSMUSG00000049321 | 117.815276 | 0.41577398 | 0.15184668 | 0.00090749 | 0.01258937 | Up | 22678 | zinc finger protein 2 [Source:MGI Symbol;Acc:MGI:99167] | Zfp2 | protein_coding |
| ENSMUSG00000049517 | 3828.06616 | -0.8248732 | 0.1934234 | 1.31E-06 | 0.0001735 | Down | 66475 | ribosomal protein S23 [Source:MGI Symbol;Acc:MGI:1913725] | Rps23 | protein_coding |
| ENSMUSG00000049577 | 223.006246 | -0.5058696 | 0.20712281 | 0.00138401 | 0.01647125 | Down | 22761 | zinc finger protein, multitype 1 [Source:MGI Symbol;Acc:MGI:1095400] | Zfpm1 | protein_coding |
| ENSMUSG00000049606 | 479.80218 | 0.34727301 | 0.1440783 | 0.00316614 | 0.02804469 | Up | 52397 | zinc finger protein 644 [Source:MGI Symbol;Acc:MGI:1277212] | Zfp644 | protein_coding |
| ENSMUSG00000049760 | 806.996842 | -0.4681039 | 0.25826008 | 0.00662748 | 0.04554291 | Down | 224904 | mitochondrial contact site and cristae organizing system subunit 13 [Source:MGI Symbol;Acc:MGI:2442174] | Micos13 | protein_coding |
| ENSMUSG00000049775 | 13863.6873 | -0.641895 | 0.27098108 | 0.00129334 | 0.0156676 | Down | 19241 | thymosin, beta 4, X chromosome [Source:MGI Symbol;Acc:MGI:99510] | Tmsb4x | protein_coding |
| ENSMUSG00000049800 | 408.147994 | 0.35089564 | 0.15581384 | 0.00464294 | 0.03593354 | Up | 58172 | SERTA domain containing 2 [Source:MGI Symbol;Acc:MGI:1931026] | Sertad2 | protein_coding |
| ENSMUSG00000049804 | 138.31173 | 0.51977388 | 0.32533269 | 0.00731121 | 0.04885669 | Up | 100503043 | armadillo repeat containing, X-linked 4 [Source:MGI Symbol;Acc:MGI:2147887] | Armcx4 | protein_coding |
| ENSMUSG00000049807 | 1906.19482 | -0.4709511 | 0.26480595 | 0.00630683 | 0.04410755 | Down | 58996 | Rho GTPase activating protein 23 [Source:MGI Symbol;Acc:MGI:3697726] | Arhgap23 | protein_coding |
| ENSMUSG00000049932 | 233.793209 | -0.5143944 | 0.18061432 | 0.00044802 | 0.00775086 | Down | 15270 | H2A.X variant histone [Source:MGI Symbol;Acc:MGI:102688] | H2ax | protein_coding |
| ENSMUSG00000049960 | 415.905712 | -0.3748925 | 0.17093316 | 0.00449602 | 0.03509281 | Down | 66242 | mitochondrial ribosomal protein S16 [Source:MGI Symbol;Acc:MGI:1913492] | Mrps16 | protein_coding |
| ENSMUSG00000050064 | 341.840799 | 0.39987435 | 0.16780854 | 0.00245582 | 0.02377525 | Up | 242109 | zinc finger protein 697 [Source:MGI Symbol;Acc:MGI:2139736] | Zfp697 | protein_coding |
| ENSMUSG00000050089 | 9.71409857 | 0.00343815 | 0.19821744 | 0.0040859 | 0.03298905 | Up | 11643 | A kinase (PRKA) anchor protein 4 [Source:MGI Symbol;Acc:MGI:102794] | Akap4 | protein_coding |
| ENSMUSG00000050148 | 1551.91672 | -0.2675082 | 0.11111971 | 0.00511155 | 0.03828357 | Down | 54609 | ubiquilin 2 [Source:MGI Symbol;Acc:MGI:1860283] | Ubqln2 | protein_coding |
| ENSMUSG00000050212 | 269.033642 | -0.8982851 | 0.25234824 | 2.16E-05 | 0.00112295 | Down | 230752 | eva-1 homolog B (C. elegans) [Source:MGI Symbol;Acc:MGI:1922063] | Eva1b | protein_coding |
| ENSMUSG00000050213 | 231.405821 | -0.2660614 | 0.11443388 | 0.00647579 | 0.0447803 | Down | 76793 | Smad nuclear interacting protein 1 [Source:MGI Symbol;Acc:MGI:2156003] | Snip1 | protein_coding |
| ENSMUSG00000050222 | 203.500861 | -0.7208623 | 0.25077613 | 0.00025336 | 0.00549267 | Down | 239114 | interleukin 17D [Source:MGI Symbol;Acc:MGI:2446510] | Il17d | protein_coding |
| ENSMUSG00000050240 | 49.758365 | 0.62920664 | 0.29064811 | 0.00195749 | 0.02048935 | Up | 58180 | hypermethylated in cancer 2 [Source:MGI Symbol;Acc:MGI:1929869] | Hic2 | protein_coding |
| ENSMUSG00000050248 | 503.601472 | -0.3151194 | 0.12936059 | 0.00342714 | 0.02941857 | Down | 68525 | EvC ciliary complex subunit 2 [Source:MGI Symbol;Acc:MGI:1915775] | Evc2 | protein_coding |
| ENSMUSG00000050288 | 282.655417 | -0.5730244 | 0.31017043 | 0.0038981 | 0.03208093 | Down | 57265 | frizzled class receptor 2 [Source:MGI Symbol;Acc:MGI:1888513] | Fzd2 | protein_coding |
| ENSMUSG00000050299 | 75.7516256 | -0.6371407 | 0.30068814 | 0.00214183 | 0.02178712 | Down | NA | predicted gene 9843 [Source:MGI Symbol;Acc:MGI:3708621] | Gm9843 | processed_pseudogene |
| ENSMUSG00000050312 | 326.73785 | 0.26140067 | 0.0959113 | 0.00228245 | 0.02259048 | Up | 106338 | NOL1/NOP2/Sun domain family member 3 [Source:MGI Symbol;Acc:MGI:2146565] | Nsun3 | protein_coding |
| ENSMUSG00000050315 | 3309.90967 | -0.6430815 | 0.34610062 | 0.00331646 | 0.02887705 | Down | 118449 | synaptopodin 2 [Source:MGI Symbol;Acc:MGI:2153070] | Synpo2 | protein_coding |
| ENSMUSG00000050335 | 2658.79539 | -0.9176922 | 0.28029033 | 6.78E-05 | 0.00225926 | Down | 16854 | lectin, galactose binding, soluble 3 [Source:MGI Symbol;Acc:MGI:96778] | Lgals3 | protein_coding |
| ENSMUSG00000050379 | 243.033509 | 0.36017267 | 0.15877365 | 0.00416375 | 0.03348522 | Up | 56526 | septin 6 [Source:MGI Symbol;Acc:MGI:1888939] | Septin6 | protein_coding |
| ENSMUSG00000050410 | 87.0508107 | -0.4596142 | 0.22633965 | 0.00430561 | 0.03418606 | Down | 106795 | transcription factor 19 [Source:MGI Symbol;Acc:MGI:103180] | Tcf19 | protein_coding |
| ENSMUSG00000050552 | 702.753331 | -0.5344422 | 0.25775055 | 0.00304951 | 0.02732451 | Down | 66096 | late endosomal/lysosomal adaptor, MAPK and MTOR activator 4 [Source:MGI Symbol;Acc:MGI:1913346] | Lamtor4 | protein_coding |
| ENSMUSG00000050628 | 348.5333 | -0.6732698 | 0.24613889 | 0.00041493 | 0.00741539 | Down | 319370 | UBA-like domain containing 2 [Source:MGI Symbol;Acc:MGI:1914635] | Ubald2 | protein_coding |
| ENSMUSG00000050666 | 247.084668 | -0.6368963 | 0.26338042 | 0.00103569 | 0.01356836 | Down | 320736 | V-set and transmembrane domain containing 4 [Source:MGI Symbol;Acc:MGI:2444633] | Vstm4 | protein_coding |
| ENSMUSG00000050668 | 336.346107 | -0.3200074 | 0.1372115 | 0.00437175 | 0.03446687 | Down | 53951 | G patch domain containing 11 [Source:MGI Symbol;Acc:MGI:1858435] | Gpatch11 | protein_coding |
| ENSMUSG00000050708 | 19968.8011 | -0.441297 | 0.16380351 | 0.00089502 | 0.01244833 | Down | 14325 | ferritin light polypeptide 1 [Source:MGI Symbol;Acc:MGI:95589] | Ftl1 | protein_coding |
| ENSMUSG00000050714 | 162.373265 | 0.46480242 | 0.22173286 | 0.00367817 | 0.0308795 | Up | 320633 | zinc finger and BTB domain containing 26 [Source:MGI Symbol;Acc:MGI:2444402] | Zbtb26 | protein_coding |
| ENSMUSG00000050730 | 325.395804 | 0.42213959 | 0.1676128 | 0.00158015 | 0.01778474 | Up | 71544 | Rho GTPase activating protein 42 [Source:MGI Symbol;Acc:MGI:1918794] | Arhgap42 | protein_coding |
| ENSMUSG00000050732 | 603.339246 | -0.800135 | 0.25373323 | 9.72E-05 | 0.00286488 | Down | 22320 | vesicle-associated membrane protein 8 [Source:MGI Symbol;Acc:MGI:1336882] | Vamp8 | protein_coding |
| ENSMUSG00000050777 | 261.05387 | -0.9725328 | 0.31632236 | 0.00010613 | 0.00303015 | Down | 170706 | transmembrane protein 37 [Source:MGI Symbol;Acc:MGI:2157899] | Tmem37 | protein_coding |

| | | | | | | | | | | |
|---|---|---|---|---|---|---|---|---|---|---|
| ENSMUSG00000050812 | 2642.8666 | 0.33327023 | 0.09643125 | 0.00014612 | 0.0037919 | Up | 230249 | Ecm29 proteasome adaptor and scaffold [Source:MGI Symbol;Acc:MGI:2140220] | Ecpas | protein_coding |
| ENSMUSG00000050890 | 166.473873 | 0.52630902 | 0.1467057 | 3.59E-05 | 0.00154093 | Up | 230809 | PDLIM1 interacting kinase 1 like [Source:MGI Symbol;Acc:MGI:2385213] | Pdik1l | protein_coding |
| ENSMUSG00000051146 | 118.692854 | -0.9302041 | 0.30238715 | 0.00010547 | 0.00301644 | Down | 73047 | calcium/calmodulin-dependent protein kinase II inhibitor 2 [Source:MGI Symbol;Acc:MGI:1920297] | Camk2n2 | protein_coding |
| ENSMUSG00000051185 | 629.395781 | -0.5686699 | 0.15270258 | 1.98E-05 | 0.0010735 | Down | 67698 | family with sequence similarity 174, member A [Source:MGI Symbol;Acc:MGI:1914948] | Fam174a | protein_coding |
| ENSMUSG00000051234 | 929.645597 | -0.3389323 | 0.12472975 | 0.001364 | 0.01630477 | Down | 19823 | ring finger protein 7 [Source:MGI Symbol;Acc:MGI:1337096] | Rnf7 | protein_coding |
| ENSMUSG00000051243 | 212.264944 | -0.6697372 | 0.35955543 | 0.00312856 | 0.02784593 | Down | 320563 | immunoglobulin superfamily containing leucine-rich repeat 2 [Source:MGI Symbol;Acc:MGI:2444277] | Islr2 | protein_coding |
| ENSMUSG00000051285 | 2460.96123 | 0.2519903 | 0.10478157 | 0.00576227 | 0.04143785 | Up | 319263 | protein-L-isoaspartate (D-aspartate) O-methyltransferase domain containing 1 [Source:MGI Symbol;Acc:MGI:2441773] | Pcmtd1 | protein_coding |
| ENSMUSG00000051314 | 869.104754 | -0.4724891 | 0.16673677 | 0.0005483 | 0.00891604 | Down | 233079 | free fatty acid receptor 2 [Source:MGI Symbol;Acc:MGI:2441731] | Ffar2 | protein_coding |
| ENSMUSG00000051319 | 579.802581 | -0.7721626 | 0.24158066 | 8.39E-05 | 0.00261825 | Down | 67885 | mitoregulin [Source:MGI Symbol;Acc:MGI:1915135] | Mtln | protein_coding |
| ENSMUSG00000051331 | 153.366488 | 0.67199969 | 0.38722637 | 0.00389514 | 0.03208093 | Up | 12288 | calcium channel, voltage-dependent, L type, alpha 1C subunit [Source:MGI Symbol;Acc:MGI:103013] | Cacna1c | protein_coding |
| ENSMUSG00000051413 | 223.297171 | 0.33409283 | 0.12983314 | 0.00217945 | 0.02194987 | Up | 54711 | pleiomorphic adenoma gene-like 2 [Source:MGI Symbol;Acc:MGI:1933165] | Plagl2 | protein_coding |
| ENSMUSG00000051439 | 539.961598 | -0.5122629 | 0.1983568 | 0.00105228 | 0.01373732 | Down | 12475 | CD14 antigen [Source:MGI Symbol;Acc:MGI:88318] | Cd14 | protein_coding |
| ENSMUSG00000051483 | 929.570912 | -0.3414005 | 0.15360614 | 0.00504214 | 0.03798203 | Down | 12408 | carbonyl reductase 1 [Source:MGI Symbol;Acc:MGI:88284] | Cbr1 | protein_coding |
| ENSMUSG00000051579 | 713.780829 | -0.3678958 | 0.16665548 | 0.00448543 | 0.03503162 | Down | 66684 | transcription elongation factor A (SII)-like 8 [Source:MGI Symbol;Acc:MGI:1913934] | Tceal8 | protein_coding |
| ENSMUSG00000051596 | 36.0783865 | -0.1394781 | 0.2763977 | 0.00222621 | 0.02225522 | Down | 21906 | otopetrin 1 [Source:MGI Symbol;Acc:MGI:2388363] | Otop1 | protein_coding |
| ENSMUSG00000051627 | 265.498583 | -0.4856187 | 0.21833857 | 0.00257651 | 0.02446978 | Down | 50709 | H1.4 linker histone, cluster member [Source:MGI Symbol;Acc:MGI:1931527] | H1f4 | protein_coding |
| ENSMUSG00000051652 | 246.324289 | -0.5919624 | 0.20179981 | 0.00027754 | 0.0057803 | Down | 237387 | leucine rich repeat containing 3 [Source:MGI Symbol;Acc:MGI:2447899] | Lrrc3 | protein_coding |
| ENSMUSG00000051671 | 224.299301 | -0.4617982 | 0.16236846 | 0.00053761 | 0.00880853 | Down | 67892 | cytochrome c oxidase assembly factor 6 [Source:MGI Symbol;Acc:MGI:1915142] | Coa6 | protein_coding |
| ENSMUSG00000051695 | 2762.96648 | -0.3127268 | 0.14659725 | 0.00745875 | 0.04956276 | Down | 23983 | poly(rC) binding protein 1 [Source:MGI Symbol;Acc:MGI:1345635] | Pcbp1 | protein_coding |
| ENSMUSG00000051748 | 355.310833 | -1.3020171 | 0.56636066 | 0.00069281 | 0.01052821 | Down | 66107 | WAP four-disulfide core domain 21 [Source:MGI Symbol;Acc:MGI:1913357] | Wfdc21 | protein_coding |
| ENSMUSG00000051851 | 451.866225 | -0.6919218 | 0.19580591 | 3.20E-05 | 0.00145985 | Down | 72865 | retrotransposon Gag like 8C [Source:MGI Symbol;Acc:MGI:1920115] | Rtl8c | protein_coding |
| ENSMUSG00000051950 | 673.6078 | 0.35170889 | 0.08916454 | 1.78E-05 | 0.00100306 | Up | 381694 | beta-3-glucosyltransferase [Source:MGI Symbol;Acc:MGI:2685903] | B3glct | protein_coding |
| ENSMUSG00000051951 | 40.198001 | -1.2305275 | 0.4895085 | 0.00044884 | 0.00775676 | Down | 497097 | X-linked Kx blood group related 4 [Source:MGI Symbol;Acc:MGI:3528744] | Xkr4 | protein_coding |
| ENSMUSG00000052144 | 1007.82652 | 0.24757102 | 0.10160009 | 0.00551338 | 0.0402901 | Up | 232314 | protein phosphatase 4, regulatory subunit 2 [Source:MGI Symbol;Acc:MGI:3027896] | Ppp4r2 | protein_coding |
| ENSMUSG00000052155 | 425.999071 | 0.43246457 | 0.13926168 | 0.00025392 | 0.00549267 | Up | 11480 | activin receptor IIA [Source:MGI Symbol;Acc:MGI:102806] | Acvr2a | protein_coding |
| ENSMUSG00000052253 | 495.33333 | -0.6857398 | 0.12694213 | 5.62E-09 | 4.35E-06 | Down | 52521 | zinc finger protein 622 [Source:MGI Symbol;Acc:MGI:1289282] | Zfp622 | protein_coding |
| ENSMUSG00000052392 | 223.641831 | 1.07768893 | 0.27128398 | 3.77E-06 | 0.00036065 | Up | 171282 | acyl-CoA thioesterase 4 [Source:MGI Symbol;Acc:MGI:2159621] | Acot4 | protein_coding |
| ENSMUSG00000052435 | 11.9346258 | -0.326169 | 1.09899553 | 0.00611573 | 0.04310193 | Down | 110794 | CCAAT/enhancer binding protein (C/EBP), epsilon [Source:MGI Symbol;Acc:MGI:103572] | Cebpe | protein_coding |
| ENSMUSG00000052446 | 187.946373 | 0.43481374 | 0.1760892 | 0.00172807 | 0.01881773 | Up | 234413 | zinc finger protein 961 [Source:MGI Symbol;Acc:MGI:3583954] | Zfp961 | protein_coding |
| ENSMUSG00000052477 | 72.8565607 | -0.740304 | 0.78877593 | 0.00710879 | 0.0479133 | Down | 620078 | RIKEN cDNA C130026I21 gene [Source:MGI Symbol;Acc:MGI:3612702] | C130026I21Rik | protein_coding |
| ENSMUSG00000052563 | 184.875518 | 0.38640024 | 0.18108766 | 0.00489004 | 0.03727269 | Up | NA | RIKEN cDNA D930048N14 gene [Source:MGI Symbol;Acc:MGI:2144709] | D930048N14Rik | protein_coding |
| ENSMUSG00000052605 | 228.280579 | -0.4470225 | 0.19072538 | 0.00224488 | 0.0223456 | Down | 67441 | isochorismatase domain containing 2b [Source:MGI Symbol;Acc:MGI:1914691] | Isoc2b | protein_coding |
| ENSMUSG00000052812 | 429.960919 | 0.38267716 | 0.13156869 | 0.00065468 | 0.0100634 | Up | 320817 | ATPase family, AAA domain containing 2B [Source:MGI Symbol;Acc:MGI:2444798] | Atad2b | protein_coding |
| ENSMUSG00000052917 | 459.956567 | 0.37652319 | 0.13239817 | 0.00082365 | 0.01178874 | Up | 66315 | SUMO1/sentrin specific peptidase 7 [Source:MGI Symbol;Acc:MGI:1913565] | Senp7 | protein_coding |
| ENSMUSG00000052926 | 287.838577 | -0.3259256 | 0.14950084 | 0.00571738 | 0.04122453 | Down | 69724 | ribonuclease H2, large subunit [Source:MGI Symbol;Acc:MGI:1916974] | Rnaseh2a | protein_coding |
| ENSMUSG00000053062 | 1147.0862 | 0.63385002 | 0.16865254 | 1.49E-05 | 0.00089149 | Up | 67374 | junction adhesion molecule 2 [Source:MGI Symbol;Acc:MGI:1933820] | Jam2 | protein_coding |
| ENSMUSG00000053173 | 7.79408082 | -1.1941314 | 0.68047572 | 0.00246008 | 0.02380222 | Down | NA | ribosomal protein L18, pseudogene 2 [Source:MGI Symbol;Acc:MGI:3646997] | Rpl18-ps2 | processed_pseudogene |
| ENSMUSG00000053182 | 23.1609871 | 0.05867449 | 0.21078567 | 0.0050031 | 0.03785529 | Up | 208166 | predicted gene 609 [Source:MGI Symbol;Acc:MGI:2685455] | Gm609 | protein_coding |
| ENSMUSG00000053329 | 1841.43561 | -0.3934159 | 0.18683326 | 0.00501489 | 0.0379028 | Down | 28295 | glutamine amidotransferase like class 1 domain containing 3A [Source:MGI Symbol;Acc:MGI:1351861] | Gatd3a | protein_coding |
| ENSMUSG00000053398 | 11422.3735 | -0.4613519 | 0.18456835 | 0.00141937 | 0.0167327 | Down | 236539 | 3-phosphoglycerate dehydrogenase [Source:MGI Symbol;Acc:MGI:1355330] | Phgdh | protein_coding |
| ENSMUSG00000053470 | 707.351466 | 0.34853869 | 0.13919179 | 0.00242936 | 0.02361755 | Up | 104263 | lysine (K)-specific demethylase 3A [Source:MGI Symbol;Acc:MGI:98847] | Kdm3a | protein_coding |
| ENSMUSG00000053477 | 1658.51651 | 0.29285377 | 0.09007733 | 0.00035962 | 0.00685683 | Up | 21413 | transcription factor 4 [Source:MGI Symbol;Acc:MGI:98506] | Tcf4 | protein_coding |
| ENSMUSG00000053553 | 102.847105 | -0.4864991 | 0.19877926 | 0.00145857 | 0.01698185 | Down | 73212 | RIKEN cDNA 3110082I17 gene [Source:MGI Symbol;Acc:MGI:1920462] | 3110082I17Rik | protein_coding |
| ENSMUSG00000053565 | 1561.20914 | -0.484176 | 0.16146149 | 0.00028494 | 0.00590411 | Down | 73830 | eukaryotic translation initiation factor 3, subunit K [Source:MGI Symbol;Acc:MGI:1921080] | Eif3k | protein_coding |
| ENSMUSG00000053702 | 26.8254401 | 0.97358705 | 0.48545913 | 0.00165504 | 0.01831478 | Up | 74103 | nebulette [Source:MGI Symbol;Acc:MGI:1921353] | Nebl | protein_coding |
| ENSMUSG00000053714 | 165.295863 | 0.65930247 | 0.36802193 | 0.0036831 | 0.03089471 | Up | 654804 | RIKEN cDNA 4732471J01 gene [Source:MGI Symbol;Acc:MGI:3603586] | 4732471J01Rik | lncRNA |
| ENSMUSG00000053746 | 74.1008489 | -1.1719539 | 0.24057909 | 6.41E-08 | 2.00E-05 | Down | 329384 | peptidyl-tRNA hydrolase 1 homolog [Source:MGI Symbol;Acc:MGI:1913779] | Ptrh1 | protein_coding |
| ENSMUSG00000053774 | 825.597088 | 0.3080644 | 0.10989833 | 0.00135033 | 0.01618906 | Up | 224111 | UBX domain protein 7 [Source:MGI Symbol;Acc:MGI:2146388] | Ubxn7 | protein_coding |
| ENSMUSG00000053801 | 257.28966 | -0.3852783 | 0.19264691 | 0.00617088 | 0.0434153 | Down | 101612 | glutamate-rich WD repeat containing 1 [Source:MGI Symbol;Acc:MGI:2141989] | Grwd1 | protein_coding |
| ENSMUSG00000053846 | 19.5267742 | 1.70984868 | 0.60510649 | 0.00016988 | 0.00421008 | Up | 16891 | lipase, endothelial [Source:MGI Symbol;Acc:MGI:1341803] | Lipg | protein_coding |
| ENSMUSG00000053886 | 239.142707 | -0.5559631 | 0.33315327 | 0.00577398 | 0.04148535 | Down | 72281 | SH2 domain containing 4A [Source:MGI Symbol;Acc:MGI:1919531] | Sh2d4a | protein_coding |
| ENSMUSG00000053907 | 4759.18131 | 0.60005449 | 0.14214117 | 2.36E-06 | 0.00025754 | Up | 232087 | methionine adenosyltransferase II, alpha [Source:MGI Symbol;Acc:MGI:2443731] | Mat2a | protein_coding |
| ENSMUSG00000053929 | 1958.66268 | -0.280948 | 0.07778476 | 9.97E-05 | 0.00291573 | Down | 54151 | cysteine and histidine rich 1 [Source:MGI Symbol;Acc:MGI:1859320] | Cyhr1 | protein_coding |
| ENSMUSG00000054000 | 106.051825 | -0.6362929 | 0.3115222 | 0.00240105 | 0.02338434 | Down | 69136 | tumor suppressor candidate 1 [Source:MGI Symbol;Acc:MGI:2684283] | Tusc1 | protein_coding |

| | | | | | | | | | | |
|---|---|---|---|---|---|---|---|---|---|---|
| ENSMUSG00000054013 | 1569.24873 | 0.50022056 | 0.25107999 | 0.00398935 | 0.03253576 | Up | 104885 | transmembrane protein 179 [Source:MGI Symbol;Acc:MGI:2144891] | Tmem179 | protein_coding |
| ENSMUSG00000054091 | 845.459226 | -1.0362243 | 0.20084466 | 1.49E-08 | 8.05E-06 | Down | 67704 | RIKEN cDNA 1810037I17 gene [Source:MGI Symbol;Acc:MGI:1914954] | 1810037I17Rik | protein_coding |
| ENSMUSG00000054204 | 172.488395 | -0.9248024 | 0.29805808 | 0.0001005 | 0.00291573 | Down | 100294583 | ALK and LTK ligand 2 [Source:MGI Symbol;Acc:MGI:3697448] | Alkal2 | protein_coding |
| ENSMUSG00000054263 | 1365.58935 | 0.23723416 | 0.08943517 | 0.00322369 | 0.02835345 | Up | 16880 | LIF receptor alpha [Source:MGI Symbol;Acc:MGI:96788] | Lifr | protein_coding |
| ENSMUSG00000054387 | 2124.33398 | 0.32214654 | 0.14153719 | 0.00508739 | 0.03820836 | Up | 17248 | transformed mouse 3T3 cell double minute 4 [Source:MGI Symbol;Acc:MGI:107934] | Mdm4 | protein_coding |
| ENSMUSG00000054404 | 2301.10819 | 0.39622628 | 0.1554719 | 0.00174657 | 0.0189533 | Up | 327978 | schlafen 5 [Source:MGI Symbol;Acc:MGI:1329004] | Slfn5 | protein_coding |
| ENSMUSG00000054418 | 11.2366831 | -1.3487193 | 0.61055375 | 0.00100648 | 0.01334711 | Down | NA | RIKEN cDNA 2900041M22 gene [Source:MGI Symbol;Acc:MGI:1925653] | 2900041M22Rik | lncRNA |
| ENSMUSG00000054428 | 319.112476 | -0.9062291 | 0.28155849 | 6.85E-05 | 0.00226099 | Down | 11983 | ATPase inhibitory factor 1 [Source:MGI Symbol;Acc:MGI:1196457] | Atpif1 | protein_coding |
| ENSMUSG00000054452 | 5083.76579 | -0.6707846 | 0.22628384 | 0.00021756 | 0.00494989 | Down | 14797 | TLE family member 5, transcriptional modulator [Source:MGI Symbol;Acc:MGI:95806] | Tle5 | protein_coding |
| ENSMUSG00000054455 | 2879.93013 | -0.2966705 | 0.10268039 | 0.00099373 | 0.01330743 | Down | 56491 | vesicle-associated membrane protein, associated protein B and C [Source:MGI Symbol;Acc:MGI:1928744] | Vapb | protein_coding |
| ENSMUSG00000054509 | 743.384331 | 0.40068846 | 0.17389855 | 0.00313221 | 0.02785044 | Up | 328417 | poly (ADP-ribose) polymerase family, member 4 [Source:MGI Symbol;Acc:MGI:2685589] | Parp4 | protein_coding |
| ENSMUSG00000054517 | 290.292405 | 0.50069993 | 0.21248338 | 0.00176768 | 0.01911844 | Up | 338364 | tripartite motif-containing 65 [Source:MGI Symbol;Acc:MGI:2442815] | Trim65 | protein_coding |
| ENSMUSG00000054589 | 20.9692602 | -1.0029014 | 0.45842645 | 0.00111489 | 0.01424975 | Down | 225609 | predicted gene 9949 [Source:MGI Symbol;Acc:MGI:3647947] | Gm9949 | lncRNA |
| ENSMUSG00000054604 | 1560.96308 | 0.25100748 | 0.0821461 | 0.00088606 | 0.01238725 | Up | 106143 | CGG triplet repeat binding protein 1 [Source:MGI Symbol;Acc:MGI:2146370] | Cggbp1 | protein_coding |
| ENSMUSG00000054612 | 251.779841 | -0.8544386 | 0.26675752 | 7.67E-05 | 0.00245645 | Down | 17314 | O-6-methylguanine-DNA methyltransferase [Source:MGI Symbol;Acc:MGI:96977] | Mgmt | protein_coding |
| ENSMUSG00000054716 | 525.120134 | -0.6173405 | 0.21392012 | 0.00031525 | 0.006307 | Down | 244216 | zinc finger protein 771 [Source:MGI Symbol;Acc:MGI:2442050] | Zfp771 | protein_coding |
| ENSMUSG00000054737 | 185.655222 | 0.50711809 | 0.18855409 | 0.00073742 | 0.01100029 | Up | 319535 | zinc finger protein 182 [Source:MGI Symbol;Acc:MGI:2442220] | Zfp182 | protein_coding |
| ENSMUSG00000054836 | 79.8082408 | -0.4008213 | 0.21049113 | 0.00715388 | 0.04813269 | Down | 72341 | elongator acetyltransferase complex subunit 6 [Source:MGI Symbol;Acc:MGI:1919349] | Elp6 | protein_coding |
| ENSMUSG00000054871 | 150.007025 | -0.6355332 | 0.40528064 | 0.00531449 | 0.03938593 | Down | 72309 | transmembrane protein 158 [Source:MGI Symbol;Acc:MGI:1919559] | Tmem158 | protein_coding |
| ENSMUSG00000054920 | 2445.00911 | -0.2713445 | 0.10170552 | 0.00244772 | 0.02373925 | Down | 71778 | kelch-like 5 [Source:MGI Symbol;Acc:MGI:1919028] | Klhl5 | protein_coding |
| ENSMUSG00000054966 | 99.9561144 | -0.5967129 | 0.2295083 | 0.00069183 | 0.01052324 | Down | 74071 | lamin tail domain containing 1 [Source:MGI Symbol;Acc:MGI:1921321] | Lmntd1 | protein_coding |
| ENSMUSG00000055044 | 745.570922 | -0.598159 | 0.16066006 | 1.84E-05 | 0.00102462 | Down | 54132 | PDZ and LIM domain 1 (elfin) [Source:MGI Symbol;Acc:MGI:1860611] | Pdlim1 | protein_coding |
| ENSMUSG00000055184 | 17.6485915 | -1.1958377 | 0.46056213 | 0.00039797 | 0.00725598 | Down | 108900 | family with sequence similarity 72, member A [Source:MGI Symbol;Acc:MGI:1919669] | Fam72a | protein_coding |
| ENSMUSG00000055302 | 3584.63699 | -0.4638191 | 0.15960941 | 0.0003385 | 0.00660925 | Down | 67568 | Morf4 family associated protein 1 [Source:MGI Symbol;Acc:MGI:1914818] | Mrfap1 | protein_coding |
| ENSMUSG00000055320 | 1979.25158 | 0.4867005 | 0.19770718 | 0.00145902 | 0.01698185 | Up | 21676 | TEA domain family member 1 [Source:MGI Symbol;Acc:MGI:101876] | Tead1 | protein_coding |
| ENSMUSG00000055401 | 497.556613 | -0.3454218 | 0.1102903 | 0.00040874 | 0.00737813 | Down | 50762 | F-box protein 6 [Source:MGI Symbol;Acc:MGI:1354743] | Fbxo6 | protein_coding |
| ENSMUSG00000055436 | 2819.55907 | 0.46745265 | 0.13919248 | 0.00010947 | 0.00308292 | Up | 69207 | serine and arginine-rich splicing factor 11 [Source:MGI Symbol;Acc:MGI:1916457] | Srsf11 | protein_coding |
| ENSMUSG00000055531 | 1123.47879 | 0.42089124 | 0.10171353 | 5.54E-06 | 0.00044782 | Up | 432508 | cleavage and polyadenylation specific factor 6 [Source:MGI Symbol;Acc:MGI:1913948] | Cpsf6 | protein_coding |
| ENSMUSG00000055681 | 1616.60645 | -0.4613648 | 0.14996027 | 0.00026574 | 0.00565053 | Down | 59042 | coatomer protein complex, subunit epsilon [Source:MGI Symbol;Acc:MGI:1891702] | Cope | protein_coding |
| ENSMUSG00000055760 | 80.751902 | -0.4761143 | 0.24730353 | 0.00492653 | 0.03743288 | Down | 67242 | gem nuclear organelle associated protein 6 [Source:MGI Symbol;Acc:MGI:1914492] | Gemin6 | protein_coding |
| ENSMUSG00000055762 | 1549.01518 | -0.4317356 | 0.16431683 | 0.00112295 | 0.0143189 | Down | 66656 | eukaryotic translation elongation factor 1 delta (guanine nucleotide exchange protein) [Source:MGI Symbol;Acc:MGI:1913906] | Eef1d | protein_coding |
| ENSMUSG00000055817 | 1107.12661 | -0.2194735 | 0.08770104 | 0.00574264 | 0.04135158 | Down | 116871 | metastasis associated 3 [Source:MGI Symbol;Acc:MGI:2151172] | Mta3 | protein_coding |
| ENSMUSG00000055839 | 2017.75446 | -0.5478188 | 0.15390346 | 3.88E-05 | 0.00161517 | Down | 67673 | elongin B [Source:MGI Symbol;Acc:MGI:1914923] | Elob | protein_coding |
| ENSMUSG00000055866 | 496.933769 | 0.91797662 | 0.41107623 | 0.00105092 | 0.01373732 | Up | 18627 | period circadian clock 2 [Source:MGI Symbol;Acc:MGI:1195265] | Per2 | protein_coding |
| ENSMUSG00000055912 | 287.234026 | -0.2887659 | 0.12428476 | 0.00558845 | 0.04062551 | Down | 232086 | transmembrane protein 150A [Source:MGI Symbol;Acc:MGI:2385244] | Tmem150a | protein_coding |
| ENSMUSG00000055972 | 39.4231307 | 0.54971892 | 0.31276009 | 0.00523455 | 0.03893554 | Up | NA | RIKEN cDNA 2810407A14 gene [Source:MGI Symbol;Acc:MGI:1917461] | 2810407A14Rik | lncRNA |
| ENSMUSG00000056091 | 378.266313 | -0.3840546 | 0.15491979 | 0.00212535 | 0.02163304 | Down | 20454 | ST3 beta-galactoside alpha-2,3-sialyltransferase 5 [Source:MGI Symbol;Acc:MGI:1339963] | St3gal5 | protein_coding |
| ENSMUSG00000056116 | 2180.67536 | -0.5092775 | 0.13893156 | 2.58E-05 | 0.00125997 | Down | 15051 | histocompatibility 2, T region locus 22 [Source:MGI Symbol;Acc:MGI:95956] | H2-T22 | protein_coding |
| ENSMUSG00000056121 | 1196.85533 | -0.3388151 | 0.14226982 | 0.00332383 | 0.02890559 | Down | 225020 | fasciculation and elongation protein zeta 2 (zygin II) [Source:MGI Symbol;Acc:MGI:2675856] | Fez2 | protein_coding |
| ENSMUSG00000056201 | 6963.35994 | -0.5412679 | 0.19893758 | 0.00059417 | 0.0094445 | Down | 12631 | cofilin 1, non-muscle [Source:MGI Symbol;Acc:MGI:101757] | Cfl1 | protein_coding |
| ENSMUSG00000056211 | 823.034015 | 0.30080283 | 0.12379541 | 0.00432443 | 0.03426847 | Up | 226412 | R3H domain containing 1 [Source:MGI Symbol;Acc:MGI:2448514] | R3hdm1 | protein_coding |
| ENSMUSG00000056214 | 221.070436 | 0.71601567 | 0.2917843 | 0.00081816 | 0.01174443 | Up | 93737 | par-6 family cell polarity regulator gamma [Source:MGI Symbol;Acc:MGI:2135606] | Pard6g | protein_coding |
| ENSMUSG00000056268 | 334.477009 | 0.37199698 | 0.13950808 | 0.00143039 | 0.01677936 | Up | 329260 | DENN/MADD domain containing 1B [Source:MGI Symbol;Acc:MGI:2447812] | Dennd1b | protein_coding |
| ENSMUSG00000056380 | 9.6525563 | -0.0702059 | 0.21722153 | 4.88E-05 | 0.00186708 | Down | 14765 | G-protein-coupled receptor 50 [Source:MGI Symbol;Acc:MGI:1333877] | Gpr50 | protein_coding |
| ENSMUSG00000056486 | 207.790666 | 0.34720306 | 0.16412794 | 0.00631273 | 0.04410755 | Up | 108699 | chimerin 1 [Source:MGI Symbol;Acc:MGI:1915674] | Chn1 | protein_coding |
| ENSMUSG00000056492 | 4015.40556 | 0.54628157 | 0.19065134 | 0.00039507 | 0.00722738 | Up | 224792 | adhesion G protein-coupled receptor F5 [Source:MGI Symbol;Acc:MGI:2182928] | Adgrf5 | protein_coding |
| ENSMUSG00000056501 | 1397.73167 | -1.5000561 | 0.32704949 | 2.09E-07 | 5.14E-05 | Down | 12608 | CCAAT/enhancer binding protein (C/EBP), beta [Source:MGI Symbol;Acc:MGI:88373] | Cebpb | protein_coding |
| ENSMUSG00000056536 | 496.061519 | 0.3131028 | 0.11573557 | 0.00167164 | 0.01843575 | Up | 27392 | phosphatidylinositol glycan anchor biosynthesis, class N [Source:MGI Symbol;Acc:MGI:1351629] | Pign | protein_coding |
| ENSMUSG00000056537 | 972.583966 | 0.38252378 | 0.08434626 | 1.20E-06 | 0.00016433 | Up | 19820 | ring finger protein, LIM domain interacting [Source:MGI Symbol;Acc:MGI:1342291] | Rlim | protein_coding |
| ENSMUSG00000056579 | 1347.38847 | 0.46271357 | 0.12531361 | 3.11E-05 | 0.00144258 | Up | NA | taurine upregulated gene 1 [Source:MGI Symbol;Acc:MGI:2144114] | Tug1 | protein_coding |
| ENSMUSG00000056596 | 119.020998 | -0.7100563 | 0.21629754 | 7.19E-05 | 0.00235058 | Down | 69539 | TMF1-regulated nuclear protein 1 [Source:MGI Symbol;Acc:MGI:1916789] | Trnp1 | protein_coding |
| ENSMUSG00000056612 | 1294.52074 | -0.7378986 | 0.21521856 | 4.22E-05 | 0.00168414 | Down | 18938 | protein phosphatase 1, regulatory inhibitor subunit 14B [Source:MGI Symbol;Acc:MGI:107682] | Ppp1r14b | protein_coding |
| ENSMUSG00000056629 | 689.46837 | -0.9934166 | 0.36079646 | 0.0002604 | 0.00556603 | Down | 114841036 | FK506 binding protein 2 [Source:MGI Symbol;Acc:MGI:95542] | Fkbp2 | protein_coding |

| | | | | | | | | | | |
|---|---|---|---|---|---|---|---|---|---|---|
| ENSMUSG00000056708 | 431.809858 | -0.9952721 | 0.38053784 | 0.00038494 | 0.00714005 | Down | 15939 | immediate early response 5 [Source:MGI Symbol;Acc:MGI:1337072] | Ier5 | protein_coding |
| ENSMUSG00000056737 | 1014.54916 | -0.7171841 | 0.26712423 | 0.00044714 | 0.00775086 | Down | 12332 | capping protein (actin filament), gelsolin-like [Source:MGI Symbol;Acc:MGI:1098259] | Capg | protein_coding |
| ENSMUSG00000056851 | 2307.27662 | 0.28895089 | 0.09546646 | 0.0006906 | 0.01051431 | Up | 18521 | poly(rC) binding protein 2 [Source:MGI Symbol;Acc:MGI:108202] | Pcbp2 | protein_coding |
| ENSMUSG00000056870 | 536.90471 | 0.67604131 | 0.34560146 | 0.00268101 | 0.0251606 | Up | 70676 | GULP, engulfment adaptor PTB domain containing 1 [Source:MGI Symbol;Acc:MGI:1920407] | Gulp1 | protein_coding |
| ENSMUSG00000056941 | 612.53782 | -0.3206209 | 0.10888688 | 0.00078223 | 0.01143766 | Down | 99311 | COMM domain containing 7 [Source:MGI Symbol;Acc:MGI:1914197] | Commd7 | protein_coding |
| ENSMUSG00000057036 | 47.087876 | -0.5112768 | 0.29117929 | 0.00584836 | 0.04177802 | Down | NA | predicted gene 7536 [Source:MGI Symbol;Acc:MGI:3645137] | Gm7536 | processed_pseudogene |
| ENSMUSG00000057130 | 369.55447 | -0.361504 | 0.16962422 | 0.00558929 | 0.04062551 | Down | 27366 | thioredoxin-like 4A [Source:MGI Symbol;Acc:MGI:1351613] | Txnl4a | protein_coding |
| ENSMUSG00000057134 | 520.742782 | -0.2554175 | 0.11080209 | 0.00717256 | 0.04816792 | Down | 211488 | 2-aminoethanethiol (cysteamine) dioxygenase [Source:MGI Symbol;Acc:MGI:2685083] | Ado | protein_coding |
| ENSMUSG00000057181 | 634.132713 | 0.33414064 | 0.11352724 | 0.00075109 | 0.01114106 | Up | 70591 | RIKEN cDNA 5730455P16 gene [Source:MGI Symbol;Acc:MGI:1917841] | 5730455P16Rik | protein_coding |
| ENSMUSG00000057234 | 69.9381016 | 0.4217668 | 0.22471967 | 0.00688674 | 0.04694719 | Up | 76894 | methyltransferase like 15 [Source:MGI Symbol;Acc:MGI:1924144] | Mettl15 | protein_coding |
| ENSMUSG00000057342 | 547.905131 | -0.3964855 | 0.14395449 | 0.00083868 | 0.01191802 | Down | 56632 | sphingosine kinase 2 [Source:MGI Symbol;Acc:MGI:1861380] | Sphk2 | protein_coding |
| ENSMUSG00000057411 | 760.864668 | -0.7925136 | 0.19324009 | 2.68E-06 | 0.0002824 | Down | 214917 | adenine nucleotide translocase lysine methyltransferase [Source:MGI Symbol;Acc:MGI:2384888] | Antkmt | protein_coding |
| ENSMUSG00000057594 | 237.840449 | -0.3977608 | 0.13809288 | 0.00065296 | 0.0100543 | Down | 70317 | ADP-ribosylation factor-like 16 [Source:MGI Symbol;Acc:MGI:1917567] | Arl16 | protein_coding |
| ENSMUSG00000057667 | 258.766782 | -0.4711609 | 0.12564508 | 2.20E-05 | 0.00113645 | Down | 232946 | biogenesis of lysosomal organelles complex-1, subunit 3 [Source:MGI Symbol;Acc:MGI:2678952] | Bloc1s3 | protein_coding |
| ENSMUSG00000057715 | 69.5247231 | 0.78693757 | 0.39121167 | 0.00200164 | 0.02081728 | Up | 320492 | RIKEN cDNA A830018L16 gene [Source:MGI Symbol;Acc:MGI:2444149] | A830018L16Rik | protein_coding |
| ENSMUSG00000057729 | 132.335755 | -0.7823715 | 0.27760264 | 0.00027815 | 0.00578569 | Down | 19152 | proteinase 3 [Source:MGI Symbol;Acc:MGI:893580] | Prtn3 | protein_coding |
| ENSMUSG00000057841 | 3998.71896 | -0.7947617 | 0.1986337 | 4.37E-06 | 0.00037622 | Down | 19951 | ribosomal protein L32 [Source:MGI Symbol;Acc:MGI:98038] | Rpl32 | protein_coding |
| ENSMUSG00000057863 | 3003.76432 | -0.6468842 | 0.21491701 | 0.00019778 | 0.00464036 | Down | 54217 | ribosomal protein L36 [Source:MGI Symbol;Acc:MGI:1860603] | Rpl36 | protein_coding |
| ENSMUSG00000057963 | 1220.18269 | -0.4006662 | 0.21369702 | 0.00732146 | 0.04888499 | Down | 217837 | inositol 1,3,4-triphosphate 5/6 kinase [Source:MGI Symbol;Acc:MGI:2446159] | Itpk1 | protein_coding |
| ENSMUSG00000058056 | 1554.37072 | -0.5624226 | 0.31330932 | 0.0050161 | 0.0379028 | Down | 72333 | palladin, cytoskeletal associated protein [Source:MGI Symbol;Acc:MGI:1919583] | Palld | protein_coding |
| ENSMUSG00000058135 | 10279.5495 | -0.6263583 | 0.14102163 | 7.78E-07 | 0.0001227 | Down | 14862 | glutathione S-transferase, mu 1 [Source:MGI Symbol;Acc:MGI:95860] | Gstm1 | protein_coding |
| ENSMUSG00000058254 | 1624.3272 | 0.26166869 | 0.1021417 | 0.00368548 | 0.03089471 | Up | 21912 | tetraspanin 7 [Source:MGI Symbol;Acc:MGI:1298407] | Tspan7 | protein_coding |
| ENSMUSG00000058258 | 448.63303 | 0.5829918 | 0.15343449 | 1.46E-05 | 0.00088781 | Up | 319554 | isopentenyl-diphosphate delta isomerase [Source:MGI Symbol;Acc:MGI:2442264] | Idi1 | protein_coding |
| ENSMUSG00000058267 | 762.395672 | -0.521693 | 0.1726567 | 0.00025566 | 0.00549552 | Down | 64659 | mitochondrial ribosomal protein S14 [Source:MGI Symbol;Acc:MGI:1928141] | Mrps14 | protein_coding |
| ENSMUSG00000058402 | 97.1389724 | 0.39853775 | 0.20953779 | 0.00736554 | 0.04911805 | Up | 233058 | zinc finger protein 420 [Source:MGI Symbol;Acc:MGI:2444666] | Zfp420 | protein_coding |
| ENSMUSG00000058542 | 22.8524214 | -1.0484697 | 0.41085013 | 0.00046013 | 0.00789467 | Down | NA | predicted gene 15590 [Source:MGI Symbol;Acc:MGI:3831433] | Gm15590 | processed_pseudogene |
| ENSMUSG00000058569 | 1372.36001 | -0.6308142 | 0.16909249 | 1.49E-05 | 0.00089149 | Down | 67511 | transmembrane p24 trafficking protein 9 [Source:MGI Symbol;Acc:MGI:1914761] | Tmed9 | protein_coding |
| ENSMUSG00000058603 | 24.7117892 | -1.1126502 | 0.4641759 | 0.00063299 | 0.00983066 | Down | NA | ribosomal protein L28, pseudogene 1 [Source:MGI Symbol;Acc:MGI:3705349] | Rpl28-ps1 | processed_pseudogene |
| ENSMUSG00000058624 | 3075.48139 | 0.31699744 | 0.1487119 | 0.00722972 | 0.04848376 | Up | 14544 | guanine deaminase [Source:MGI Symbol;Acc:MGI:95678] | Gda | protein_coding |
| ENSMUSG00000058656 | 92.548637 | 0.72674794 | 0.23348598 | 0.00012478 | 0.00338386 | Up | 320679 | sterile alpha motif domain containing 12 [Source:MGI Symbol;Acc:MGI:2444518] | Samd12 | protein_coding |
| ENSMUSG00000058672 | 1129.70096 | -0.5016215 | 0.2436963 | 0.0033728 | 0.02914423 | Down | 22151 | tubulin, beta 2A class IIA [Source:MGI Symbol;Acc:MGI:107861] | Tubb2a | protein_coding |
| ENSMUSG00000058706 | 761.09275 | 0.39262761 | 0.11488065 | 0.000117 | 0.00324901 | Up | 68364 | RIKEN cDNA 0610030E20 gene [Source:MGI Symbol;Acc:MGI:1915614] | 0610030E20Rik | protein_coding |
| ENSMUSG00000058709 | 1060.53569 | -0.3861371 | 0.12375438 | 0.00030522 | 0.00615946 | Down | 112406 | egl-9 family hypoxia-inducible factor 2 [Source:MGI Symbol;Acc:MGI:1932287] | Egln2 | protein_coding |
| ENSMUSG00000058743 | 147.038707 | -2.0595789 | 0.54591509 | 6.16E-06 | 0.00047651 | Down | 211480 | potassium inwardly-rectifying channel, subfamily J, member 14 [Source:MGI Symbol;Acc:MGI:2384820] | Kcnj14 | protein_coding |
| ENSMUSG00000058833 | 1076.19682 | -0.8598343 | 0.19478242 | 7.48E-07 | 0.00011943 | Down | 66462 | required for excision 1-B domain containing [Source:MGI Symbol;Acc:MGI:1913712] | Rex1bd | protein_coding |
| ENSMUSG00000059027 | 340.338364 | 0.66628303 | 0.22312056 | 0.00020616 | 0.00475336 | Up | NA | RIKEN cDNA 9630013D21 gene [Source:MGI Symbol;Acc:MGI:2442649] | 9630013D21Rik | lncRNA |
| ENSMUSG00000059070 | 4063.31459 | -0.625835 | 0.18378601 | 5.38E-05 | 0.00194658 | Down | 19899 | ribosomal protein L18 [Source:MGI Symbol;Acc:MGI:98003] | Rpl18 | protein_coding |
| ENSMUSG00000059146 | 127.512415 | 0.65009116 | 0.27679066 | 0.00121241 | 0.01504799 | Up | 18213 | neurotrophic tyrosine kinase, receptor, type 3 [Source:MGI Symbol;Acc:MGI:97385] | Ntrk3 | protein_coding |
| ENSMUSG00000059149 | 376.928079 | 0.5026123 | 0.19448773 | 0.00097709 | 0.01321633 | Up | 213006 | major facilitator superfamily domain containing 4A [Source:MGI Symbol;Acc:MGI:2442786] | Mfsd4a | protein_coding |
| ENSMUSG00000059201 | 37111.7476 | -1.2601404 | 0.51386668 | 0.00048831 | 0.00825092 | Down | 16846 | leptin [Source:MGI Symbol;Acc:MGI:104663] | Lep | protein_coding |
| ENSMUSG00000059248 | 1743.25451 | -0.7129537 | 0.30961073 | 0.00118602 | 0.0148328 | Down | 53860 | septin 9 [Source:MGI Symbol;Acc:MGI:1858222] | Septin9 | protein_coding |
| ENSMUSG00000059278 | 282.492985 | -0.5688183 | 0.35491589 | 0.00602458 | 0.0426189 | Down | 78304 | N(alpha)-acetyltransferase 38, NatC auxiliary subunit [Source:MGI Symbol;Acc:MGI:1925554] | Naa38 | protein_coding |
| ENSMUSG00000059291 | 5121.5827 | -0.7239207 | 0.20611567 | 3.10E-05 | 0.00144162 | Down | 67025 | ribosomal protein L11 [Source:MGI Symbol;Acc:MGI:1914275] | Rpl11 | protein_coding |
| ENSMUSG00000059326 | 971.784697 | -0.4286483 | 0.20420722 | 0.00428191 | 0.03409789 | Down | 12982 | colony stimulating factor 2 receptor, alpha, low-affinity (granulocyte-macrophage) [Source:MGI Symbol;Acc:MGI:1339754] | Csf2ra | protein_coding |
| ENSMUSG00000059355 | 838.034323 | -0.451436 | 0.19838838 | 0.00250974 | 0.02409617 | Down | 414077 | WD repeat domain 83 opposite strand [Source:MGI Symbol;Acc:MGI:3041257] | Wdr83os | protein_coding |
| ENSMUSG00000059412 | 194.681106 | -0.8633367 | 0.31823323 | 0.00032919 | 0.00650564 | Down | 11936 | FXYD domain-containing ion transport regulator 2 [Source:MGI Symbol;Acc:MGI:1195260] | Fxyd2 | protein_coding |
| ENSMUSG00000059474 | 431.02093 | 0.49075434 | 0.13398778 | 3.05E-05 | 0.00143394 | Up | 103537 | mbt domain containing 1 [Source:MGI Symbol;Acc:MGI:2143977] | Mbtd1 | protein_coding |
| ENSMUSG00000059486 | 635.586742 | 0.26498685 | 0.11042663 | 0.00533558 | 0.0394522 | Up | 210973 | kelch repeat and BTB (POZ) domain containing 2 [Source:MGI Symbol;Acc:MGI:2384811] | Kbtbd2 | protein_coding |
| ENSMUSG00000059518 | 552.294764 | -0.6227109 | 0.15574867 | 5.71E-06 | 0.00045502 | Down | 70103 | zinc finger, HIT domain containing 1 [Source:MGI Symbol;Acc:MGI:1917353] | Znhit1 | protein_coding |
| ENSMUSG00000059588 | 316.031308 | 0.56553506 | 0.21403025 | 0.00070821 | 0.01074215 | Up | 54598 | calcitonin receptor-like [Source:MGI Symbol;Acc:MGI:1926944] | Calcrl | protein_coding |
| ENSMUSG00000059714 | 1974.81955 | -0.6346084 | 0.17340546 | 2.11E-05 | 0.00111477 | Down | 14251 | flotillin 1 [Source:MGI Symbol;Acc:MGI:1100500] | Flot1 | protein_coding |
| ENSMUSG00000059742 | 26.3307794 | 0.65432858 | 0.378981 | 0.00423233 | 0.03384218 | Up | 170738 | potassium voltage-gated channel, subfamily H (eag-related), member 7 [Source:MGI Symbol;Acc:MGI:2159566] | Kcnh7 | protein_coding |
| ENSMUSG00000059791 | 102.660393 | -0.5241961 | 0.24013009 | 0.00251233 | 0.02410678 | Down | 106582 | nurim (nuclear envelope membrane protein) [Source:MGI Symbol;Acc:MGI:2146855] | Nrm | protein_coding |
| ENSMUSG00000060019 | 10.0581967 | -1.2589094 | 0.54775604 | 0.00080959 | 0.01167356 | Down | NA | predicted pseudogene 10073 [Source:MGI Symbol;Acc:MGI:3641908] | Gm10073 | processed_pseudogene |

| | | | | | | | | | | |
|---|---|---|---|---|---|---|---|---|---|---|
| ENSMUSG00000060032 | 794.730912 | -1.4876886 | 0.28822353 | 1.14E-08 | 6.87E-06 | Down | 232440 | H2J.A histone [Source:MGI Symbol;Acc:MGI:3606192] | H2aj | protein_coding |
| ENSMUSG00000060063 | 307.694643 | -0.80944 | 0.29956259 | 0.00036701 | 0.00694027 | Down | 11690 | arachidonate 5-lipoxygenase activating protein [Source:MGI Symbol;Acc:MGI:107505] | Alox5ap | protein_coding |
| ENSMUSG00000060147 | 3396.38502 | -0.5020953 | 0.15357162 | 0.00012498 | 0.00338386 | Down | 20719 | serine (or cysteine) peptidase inhibitor, clade B, member 6a [Source:MGI Symbol;Acc:MGI:103123] | Serpinb6a | protein_coding |
| ENSMUSG00000060288 | 114.019852 | -0.4037842 | 0.15239013 | 0.00123152 | 0.01520221 | Down | 66101 | peptidyl prolyl isomerase H [Source:MGI Symbol;Acc:MGI:106499] | Ppih | protein_coding |
| ENSMUSG00000060427 | 134.10992 | 0.34440076 | 0.15864291 | 0.00561232 | 0.0406814 | Up | 234362 | zinc finger protein 868 [Source:MGI Symbol;Acc:MGI:2142546] | Zfp868 | protein_coding |
| ENSMUSG00000060429 | 3104.63038 | 0.459501 | 0.1577367 | 0.00050741 | 0.00849783 | Up | 20649 | syntrophin, basic 1 [Source:MGI Symbol;Acc:MGI:101781] | Sntb1 | protein_coding |
| ENSMUSG00000060445 | 55.7498663 | 0.69665605 | 0.36213073 | 0.00286423 | 0.0263027 | Up | 320558 | synaptonemal complex protein 2 [Source:MGI Symbol;Acc:MGI:1933281] | Sycp2 | protein_coding |
| ENSMUSG00000060510 | 801.571543 | 0.46981782 | 0.12371146 | 1.98E-05 | 0.0010735 | Up | 77519 | zinc finger protein 266 [Source:MGI Symbol;Acc:MGI:1924769] | Zfp266 | protein_coding |
| ENSMUSG00000060538 | 406.47397 | -0.362707 | 0.11711711 | 0.00041201 | 0.00739578 | Down | 68742 | transmembrane protein 219 [Source:MGI Symbol;Acc:MGI:1915992] | Tmem219 | protein_coding |
| ENSMUSG00000060579 | 85.6449441 | -0.5775703 | 0.2439362 | 0.00140777 | 0.01663216 | Down | 14198 | fragile histidine triad gene [Source:MGI Symbol;Acc:MGI:1277947] | Fhit | protein_coding |
| ENSMUSG00000060591 | 1350.9748 | -0.782767 | 0.11609437 | 8.10E-13 | 4.13E-09 | Down | 80876 | interferon induced transmembrane protein 2 [Source:MGI Symbol;Acc:MGI:1933382] | Ifitm2 | protein_coding |
| ENSMUSG00000060601 | 1334.05851 | -0.3341565 | 0.1113786 | 0.00062968 | 0.00979801 | Down | 22260 | nuclear receptor subfamily 1, group H, member 2 [Source:MGI Symbol;Acc:MGI:1352463] | Nr1h2 | protein_coding |
| ENSMUSG00000060657 | 1390.74262 | 0.2957136 | 0.10770424 | 0.00171512 | 0.01877503 | Up | 223989 | meiosis regulator and mRNA stability 1 [Source:MGI Symbol;Acc:MGI:2444505] | Marf1 | protein_coding |
| ENSMUSG00000060678 | 20.647524 | -0.8894126 | 0.59041916 | 0.00397787 | 0.03248895 | Down | 319155 | H4 clustered histone 3 [Source:MGI Symbol;Acc:MGI:2448421] | H4c3 | protein_coding |
| ENSMUSG00000060680 | 7.68137656 | -2.909117 | 1.00928336 | 0.00014246 | 0.00370879 | Down | NA | predicted gene 8894 [Source:MGI Symbol;Acc:MGI:3643084] | Gm8894 | processed_pseudogene |
| ENSMUSG00000060703 | 2365.98587 | -0.3822048 | 0.15647349 | 0.00231542 | 0.02285171 | Down | 66205 | CD302 antigen [Source:MGI Symbol;Acc:MGI:1913455] | Cd302 | protein_coding |
| ENSMUSG00000060708 | 290.532389 | -0.6014324 | 0.17116396 | 3.92E-05 | 0.00161915 | Down | 117197 | biogenesis of lysosomal organelles complex-1, subunit 4, cappuccino [Source:MGI Symbol;Acc:MGI:1929230] | Bloc1s4 | protein_coding |
| ENSMUSG00000060743 | 2223.7398 | -0.5645753 | 0.16737996 | 7.08E-05 | 0.00231999 | Down | 15078 | H3.3 histone A [Source:MGI Symbol;Acc:MGI:1097686] | H3f3a | protein_coding |
| ENSMUSG00000060791 | 198.656494 | -0.5409996 | 0.34553024 | 0.00670717 | 0.04595441 | Down | 63986 | glia maturation factor, gamma [Source:MGI Symbol;Acc:MGI:1927135] | Gmfg | protein_coding |
| ENSMUSG00000060802 | 31794.5601 | -0.411897 | 0.2099675 | 0.00603112 | 0.0426351 | Down | 12010 | beta-2 microglobulin [Source:MGI Symbol;Acc:MGI:88127] | B2m | protein_coding |
| ENSMUSG00000060860 | 569.180904 | -0.4222522 | 0.17896754 | 0.00244666 | 0.02373925 | Down | 77891 | ubiquitin-conjugating enzyme E2S [Source:MGI Symbol;Acc:MGI:1925141] | Ube2s | protein_coding |
| ENSMUSG00000060878 | 14.4697908 | 1.23778022 | 0.45684545 | 0.00029157 | 0.0060033 | Up | 258405 | olfactory receptor 1420 [Source:MGI Symbol;Acc:MGI:3031254] | Olfr1420 | protein_coding |
| ENSMUSG00000060950 | 215.371201 | -0.5503047 | 0.19901884 | 0.00051264 | 0.00855014 | Down | 328162 | tRNA methyltransferase 61A [Source:MGI Symbol;Acc:MGI:2443487] | Trmt61a | protein_coding |
| ENSMUSG00000060981 | 511.535445 | -1.3341157 | 0.23932122 | 1.29E-09 | 1.61E-06 | Down | 69386 | H4 clustered histone 8 [Source:MGI Symbol;Acc:MGI:2448427] | H4c8 | protein_coding |
| ENSMUSG00000061024 | 532.669493 | -0.3650167 | 0.13077561 | 0.00098251 | 0.01325648 | Down | 59014 | ribosome biogenesis regulator 1 [Source:MGI Symbol;Acc:MGI:1929721] | Rrs1 | protein_coding |
| ENSMUSG00000061046 | 157.744114 | -0.4056065 | 0.19408442 | 0.00477209 | 0.03665371 | Down | 68977 | hydroxyacylglutathione hydrolase-like [Source:MGI Symbol;Acc:MGI:1919877] | Haghl | protein_coding |
| ENSMUSG00000061086 | 172.420101 | -1.0967368 | 0.4405117 | 0.00049829 | 0.00837668 | Down | 17896 | myosin, light polypeptide 4 [Source:MGI Symbol;Acc:MGI:97267] | Myl4 | protein_coding |
| ENSMUSG00000061100 | 8341.45325 | -0.5954829 | 0.36877141 | 0.00546688 | 0.04011264 | Down | 57262 | resistin like alpha [Source:MGI Symbol;Acc:MGI:1888504] | Retnla | protein_coding |
| ENSMUSG00000061111 | 938.394191 | -0.5362236 | 0.17202801 | 0.00018398 | 0.00444761 | Down | 192173 | MAPK regulated corepressor interacting protein 1 [Source:MGI Symbol;Acc:MGI:2384752] | Mcrip1 | protein_coding |
| ENSMUSG00000061175 | 341.40662 | 0.36942463 | 0.14451953 | 0.00186533 | 0.0197925 | Up | 329679 | folliculin interacting protein 2 [Source:MGI Symbol;Acc:MGI:2683054] | Fnip2 | protein_coding |
| ENSMUSG00000061232 | 17200.298 | -0.3999364 | 0.20094431 | 0.0060587 | 0.04279286 | Down | 14972 | histocompatibility 2, K1, K region [Source:MGI Symbol;Acc:MGI:95904] | H2-K1 | protein_coding |
| ENSMUSG00000061273 | 1228.62128 | 0.29120391 | 0.13288617 | 0.00748474 | 0.04964493 | Up | 236792 | membrane magnesium transporter 1 [Source:MGI Symbol;Acc:MGI:2384305] | Mmgt1 | protein_coding |
| ENSMUSG00000061286 | 593.04925 | -0.6022208 | 0.17618318 | 5.77E-05 | 0.00203037 | Down | 27998 | exosome component 5 [Source:MGI Symbol;Acc:MGI:107889] | Exosc5 | protein_coding |
| ENSMUSG00000061313 | 1755.07751 | 0.49073332 | 0.17459409 | 0.00057125 | 0.00917906 | Up | 72108 | DDHD domain containing 2 [Source:MGI Symbol;Acc:MGI:1919358] | Ddhd2 | protein_coding |
| ENSMUSG00000061397 | 150.35147 | -0.9049528 | 0.37163318 | 0.00066305 | 0.01016156 | Down | 223917 | keratin 79 [Source:MGI Symbol;Acc:MGI:2385030] | Krt79 | protein_coding |
| ENSMUSG00000061479 | 681.91711 | -0.3214662 | 0.10853573 | 0.00075593 | 0.01119418 | Down | 53607 | small nuclear ribonucleoprotein polypeptide A [Source:MGI Symbol;Acc:MGI:1855690] | Snrpa | protein_coding |
| ENSMUSG00000061518 | 1500.12686 | -0.6803019 | 0.28441591 | 0.00098563 | 0.01327032 | Down | 12859 | cytochrome c oxidase subunit 5B [Source:MGI Symbol;Acc:MGI:88475] | Cox5b | protein_coding |
| ENSMUSG00000061540 | 43.2015378 | -0.6621105 | 0.49757301 | 0.00645533 | 0.04471933 | Down | 18406 | orosomucoid 2 [Source:MGI Symbol;Acc:MGI:97444] | Orm2 | protein_coding |
| ENSMUSG00000061544 | 155.337057 | 0.39264204 | 0.18945222 | 0.00535986 | 0.03955973 | Up | 381067 | zinc finger protein 229 [Source:MGI Symbol;Acc:MGI:2679295] | Zfp229 | protein_coding |
| ENSMUSG00000061650 | 605.041404 | -0.6216197 | 0.15126134 | 3.62E-06 | 0.00035208 | Down | 192191 | mediator complex subunit 9 [Source:MGI Symbol;Acc:MGI:2183151] | Med9 | protein_coding |
| ENSMUSG00000061740 | 953.015658 | 0.33361886 | 0.15339708 | 0.00598667 | 0.04250589 | Up | 56448 | cytochrome P450, family 2, subfamily d, polypeptide 22 [Source:MGI Symbol;Acc:MGI:1929474] | Cyp2d22 | protein_coding |
| ENSMUSG00000061787 | 3401.33156 | -0.4441255 | 0.16230587 | 0.00076909 | 0.01130674 | Down | 20068 | ribosomal protein S17 [Source:MGI Symbol;Acc:MGI:1309526] | Rps17 | protein_coding |
| ENSMUSG00000061882 | 132.469511 | 0.70439318 | 0.25788554 | 0.00040876 | 0.00737813 | Up | 208908 | coiled-coil domain containing 62 [Source:MGI Symbol;Acc:MGI:2684996] | Ccdc62 | protein_coding |
| ENSMUSG00000061981 | 2172.26947 | -0.2020496 | 0.06873408 | 0.00165408 | 0.01831478 | Down | 14252 | flotillin 2 [Source:MGI Symbol;Acc:MGI:103309] | Flot2 | protein_coding |
| ENSMUSG00000062006 | 3614.89735 | -0.54862 | 0.15733073 | 4.89E-05 | 0.00186708 | Down | 619547 | ribosomal protein L34 [Source:MGI Symbol;Acc:MGI:1915686] | Rpl34 | protein_coding |
| ENSMUSG00000062031 | 686.755429 | -0.2496691 | 0.09697423 | 0.00278045 | 0.02572235 | Down | 212974 | protein glucosylgalactosylhydroxylysine glucosidase [Source:MGI Symbol;Acc:MGI:2444047] | Pgghg | protein_coding |
| ENSMUSG00000062078 | 5013.07505 | 0.38856036 | 0.09308188 | 5.57E-06 | 0.00044783 | Up | 19317 | quaking, KH domain containing RNA binding [Source:MGI Symbol;Acc:MGI:97837] | Qki | protein_coding |
| ENSMUSG00000062081 | 8.39882687 | -0.9238014 | 0.79060022 | 0.00581186 | 0.04166534 | Down | NA | predicted gene 6055 [Source:MGI Symbol;Acc:MGI:3648620] | Gm6055 | processed_pseudogene |
| ENSMUSG00000062127 | 564.328471 | 0.32290616 | 0.12856178 | 0.00276636 | 0.0256852 | Up | 80281 | CTTNBP2 N-terminal like [Source:MGI Symbol;Acc:MGI:1933137] | Cttnbp2nl | protein_coding |
| ENSMUSG00000062198 | 190.420881 | 0.42453557 | 0.18588336 | 0.0028971 | 0.02650557 | Up | 72658 | RIKEN cDNA 2700097O09 gene [Source:MGI Symbol;Acc:MGI:1919908] | 2700097O09Rik | protein_coding |
| ENSMUSG00000062270 | 2665.37499 | -0.3064348 | 0.12863353 | 0.00377824 | 0.03137911 | Down | 627352 | mortality factor 4 like 1 [Source:MGI Symbol;Acc:MGI:1096551] | Morf4l1 | protein_coding |
| ENSMUSG00000062296 | 49.848263 | 0.06275935 | 0.21264693 | 0.0044066 | 0.0346324 | Up | 320429 | tetratricopeptide repeat and ankyrin repeat containing 1 [Source:MGI Symbol;Acc:MGI:1341834] | Trank1 | protein_coding |
| ENSMUSG00000062519 | 259.231197 | 0.45342981 | 0.1865593 | 0.00175705 | 0.01902881 | Up | 272347 | zinc finger protein 398 [Source:MGI Symbol;Acc:MGI:1917856] | Zfp398 | protein_coding |
| ENSMUSG00000062580 | 1107.90754 | -0.4394006 | 0.15847462 | 0.00073153 | 0.01097291 | Down | 21854 | translocase of inner mitochondrial membrane 17a [Source:MGI Symbol;Acc:MGI:1343131] | Timm17a | protein_coding |

| | | | | | | | | | | |
|---|---|---|---|---|---|---|---|---|---|---|
| ENSMUSG00000062604 | 1434.78232 | 0.32756612 | 0.10115032 | 0.00030227 | 0.00614327 | Up | 20817 | serine/arginine-rich protein specific kinase 2 [Source:MGI Symbol;Acc:MGI:1201408] | Srpk2 | protein_coding |
| ENSMUSG00000062619 | 219.554248 | -0.5833159 | 0.21978413 | 0.00064866 | 0.01003147 | Down | 67101 | RIKEN cDNA 2310039H08 gene [Source:MGI Symbol;Acc:MGI:1914351] | 2310039H08Rik | protein_coding |
| ENSMUSG00000062647 | 5082.01158 | -0.4240943 | 0.17904263 | 0.00260663 | 0.02467643 | Down | 27176 | ribosomal protein L7A [Source:MGI Symbol;Acc:MGI:1353472] | Rpl7a | protein_coding |
| ENSMUSG00000062753 | 164.558196 | -0.7232181 | 0.26397532 | 0.00037326 | 0.00700994 | Down | 106672 | expressed sequence AI413582 [Source:MGI Symbol;Acc:MGI:2146839] | AI413582 | protein_coding |
| ENSMUSG00000062797 | 373.612429 | -0.460761 | 0.17438653 | 0.00097963 | 0.01322868 | Down | 67669 | heat shock protein nuclear import factor [Source:MGI Symbol;Acc:MGI:96738] | Hikeshi | protein_coding |
| ENSMUSG00000062933 | 322.695482 | -0.5418748 | 0.19871362 | 0.00061039 | 0.00960103 | Down | NA | predicted pseudogene 10123 [Source:MGI Symbol;Acc:MGI:3704342] | Gm10123 | processed_pseudogene |
| ENSMUSG00000062949 | 641.951509 | 0.63998154 | 0.1589667 | 5.05E-06 | 0.00042282 | Up | 320940 | ATPase, class VI, type 11C [Source:MGI Symbol;Acc:MGI:1859661] | Atp11c | protein_coding |
| ENSMUSG00000062963 | 1138.89866 | -0.5030516 | 0.17915776 | 0.00054117 | 0.00885332 | Down | 66155 | ubiquitin-fold modifier conjugating enzyme 1 [Source:MGI Symbol;Acc:MGI:1913405] | Ufc1 | protein_coding |
| ENSMUSG00000062997 | 2545.67448 | -1.3145758 | 0.29951666 | 5.74E-07 | 9.82E-05 | Down | 66489 | ribosomal protein L35 [Source:MGI Symbol;Acc:MGI:1913739] | Rpl35 | protein_coding |
| ENSMUSG00000063019 | 388.234648 | -0.6657565 | 0.16835424 | 6.29E-06 | 0.00047968 | Down | 69161 | mannosidase, beta A, lysosomal-like [Source:MGI Symbol;Acc:MGI:1916411] | Manbal | protein_coding |
| ENSMUSG00000063065 | 2543.05186 | -0.2885432 | 0.11322441 | 0.00340328 | 0.02929847 | Down | 26417 | mitogen-activated protein kinase 3 [Source:MGI Symbol;Acc:MGI:1346859] | Mapk3 | protein_coding |
| ENSMUSG00000063108 | 306.833244 | 0.48102445 | 0.1308148 | 3.06E-05 | 0.00143459 | Up | 22688 | zinc finger protein 26 [Source:MGI Symbol;Acc:MGI:99173] | Zfp26 | protein_coding |
| ENSMUSG00000063171 | 619.39805 | -0.4595115 | 0.19533856 | 0.00230949 | 0.02280711 | Down | NA | ribosomal protein S4-like [Source:MGI Symbol;Acc:MGI:1913434] | Rps4l | transcribed_processed_pseudogene |
| ENSMUSG00000063235 | 580.761396 | -0.4654177 | 0.13264195 | 5.21E-05 | 0.00191573 | Down | 66461 | protein tyrosine phosphatase, mitochondrial 1 [Source:MGI Symbol;Acc:MGI:1913711] | Ptpmt1 | protein_coding |
| ENSMUSG00000063236 | 302.045683 | -0.5533701 | 0.211136 | 0.00076037 | 0.01122243 | Down | 117171 | RIKEN cDNA 1110038F14 gene [Source:MGI Symbol;Acc:MGI:2152337] | 1110038F14Rik | protein_coding |
| ENSMUSG00000063275 | 327.875213 | -0.5931775 | 0.17925637 | 8.48E-05 | 0.00262438 | Down | 30963 | 3-hydroxyacyl-CoA dehydratase 1 [Source:MGI Symbol;Acc:MGI:1353592] | Hacd1 | protein_coding |
| ENSMUSG00000063316 | 3541.21004 | -0.6735091 | 0.1495714 | 5.55E-07 | 9.59E-05 | Down | 19942 | ribosomal protein L27 [Source:MGI Symbol;Acc:MGI:98036] | Rpl27 | protein_coding |
| ENSMUSG00000063358 | 2930.80766 | 0.16898887 | 0.0624483 | 0.0043771 | 0.03446729 | Up | 26413 | mitogen-activated protein kinase 1 [Source:MGI Symbol;Acc:MGI:1346858] | Mapk1 | protein_coding |
| ENSMUSG00000063457 | 4719.93078 | -0.8486407 | 0.24286221 | 2.80E-05 | 0.00134946 | Down | 20054 | ribosomal protein S15 [Source:MGI Symbol;Acc:MGI:98117] | Rps15 | protein_coding |
| ENSMUSG00000063550 | 1010.44442 | 0.32346898 | 0.12983804 | 0.00294317 | 0.0266658 | Up | 269966 | nucleoporin 98 [Source:MGI Symbol;Acc:MGI:109404] | Nup98 | protein_coding |
| ENSMUSG00000063605 | 240.575621 | -0.8873401 | 0.18994827 | 1.96E-07 | 4.89E-05 | Down | 234582 | coiled-coil domain containing 102A [Source:MGI Symbol;Acc:MGI:2686927] | Ccdc102a | protein_coding |
| ENSMUSG00000063663 | 174.657871 | 0.35119247 | 0.16208283 | 0.00548038 | 0.04015735 | Up | 382236 | bromodomain and WD repeat domain containing 3 [Source:MGI Symbol;Acc:MGI:3029414] | Brwd3 | protein_coding |
| ENSMUSG00000063696 | 14.6732796 | -0.5990089 | 0.41267044 | 0.00725436 | 0.04855734 | Down | NA | predicted pseudogene 8730 [Source:MGI Symbol;Acc:MGI:3644565] | Gm8730 | processed_pseudogene |
| ENSMUSG00000063787 | 452.155794 | -0.4357742 | 0.18009662 | 0.00201533 | 0.02089285 | Down | 66121 | coiled-coil-helix-coiled-coil-helix domain containing 1 [Source:MGI Symbol;Acc:MGI:1913371] | Chchd1 | protein_coding |
| ENSMUSG00000063802 | 449.144059 | -0.4341357 | 0.13609817 | 0.0001642 | 0.00411638 | Down | 66245 | HSPA (heat shock 70kDa) binding protein, cytoplasmic cochaperone 1 [Source:MGI Symbol;Acc:MGI:1913495] | Hspbp1 | protein_coding |
| ENSMUSG00000063838 | 109.902564 | -0.7837067 | 0.33910405 | 0.0010337 | 0.01355327 | Down | 58804 | CDC42 effector protein (Rho GTPase binding) 5 [Source:MGI Symbol;Acc:MGI:1929745] | Cdc42ep5 | protein_coding |
| ENSMUSG00000063884 | 817.707349 | 0.33012827 | 0.09485917 | 0.00012648 | 0.00341634 | Up | 69956 | pentatricopeptide repeat domain 3 [Source:MGI Symbol;Acc:MGI:1917206] | Ptcd3 | protein_coding |
| ENSMUSG00000063894 | 624.798297 | 0.46664872 | 0.19245342 | 0.00177078 | 0.01913928 | Up | 93681 | zinc finger with KRAB and SCAN domains 8 [Source:MGI Symbol;Acc:MGI:1913815] | Zkscan8 | protein_coding |
| ENSMUSG00000063904 | 809.363244 | -0.4030086 | 0.1378188 | 0.00050796 | 0.00849819 | Down | 75221 | dipeptidylpeptidase 3 [Source:MGI Symbol;Acc:MGI:1922471] | Dpp3 | protein_coding |
| ENSMUSG00000064080 | 5422.32902 | -0.4237401 | 0.18103742 | 0.00290788 | 0.02653846 | Down | 14115 | fibulin 2 [Source:MGI Symbol;Acc:MGI:95488] | Fbln2 | protein_coding |
| ENSMUSG00000064284 | 294.871466 | -0.4122953 | 0.16617875 | 0.00171662 | 0.01877879 | Down | 72355 | cysteine rich, DPF motif domain containing 1 [Source:MGI Symbol;Acc:MGI:1919605] | Cdpf1 | protein_coding |
| ENSMUSG00000064326 | 245.168286 | -0.4736651 | 0.22598282 | 0.00349831 | 0.02986344 | Down | 30954 | SIVA1, apoptosis-inducing factor [Source:MGI Symbol;Acc:MGI:1353606] | Siva1 | protein_coding |
| ENSMUSG00000064339 | 6298.85201 | -0.5572043 | 0.36951565 | 0.00702216 | 0.0475708 | Down | NA | mitochondrially encoded 16S rRNA [Source:MGI Symbol;Acc:MGI:102492] | mt-Rnr2 | Mt_rRNA |
| ENSMUSG00000064350 | 120.245127 | -1.0993517 | 0.48928022 | 0.00086916 | 0.01226722 | Down | NA | mitochondrially encoded tRNA tyrosine [Source:MGI Symbol;Acc:MGI:102470] | mt-Ty | Mt_tRNA |
| ENSMUSG00000064351 | 313672.589 | 0.42788124 | 0.20250999 | 0.00419683 | 0.0336513 | Up | 17708 | mitochondrially encoded cytochrome c oxidase I [Source:MGI Symbol;Acc:MGI:102504] | mt-Co1 | protein_coding |
| ENSMUSG00000064360 | 329.200457 | 0.52089826 | 0.30859051 | 0.00647791 | 0.0447803 | Up | 17718 | mitochondrially encoded NADH dehydrogenase 3 [Source:MGI Symbol;Acc:MGI:102499] | mt-Nd3 | protein_coding |
| ENSMUSG00000064363 | 73409.6209 | 0.39584762 | 0.19012195 | 0.00522536 | 0.03888499 | Up | 17719 | mitochondrially encoded NADH dehydrogenase 4 [Source:MGI Symbol;Acc:MGI:102498] | mt-Nd4 | protein_coding |
| ENSMUSG00000064367 | 97638.1055 | 0.60342215 | 0.27624248 | 0.00197015 | 0.02058206 | Up | 17721 | mitochondrially encoded NADH dehydrogenase 5 [Source:MGI Symbol;Acc:MGI:102496] | mt-Nd5 | protein_coding |
| ENSMUSG00000064370 | 165241.175 | 0.39609732 | 0.17456595 | 0.00343746 | 0.02946779 | Up | 17711 | mitochondrially encoded cytochrome b [Source:MGI Symbol;Acc:MGI:102501] | mt-Cytb | protein_coding |
| ENSMUSG00000064372 | 1736.90226 | 1.60881773 | 0.44624135 | 1.32E-05 | 0.0008203 | Up | NA | mitochondrially encoded tRNA proline [Source:MGI Symbol;Acc:MGI:102478] | mt-Tp | Mt_tRNA |
| ENSMUSG00000064582 | 16.1534644 | 1.05057338 | 0.62015862 | 0.00277737 | 0.02571326 | Up | 115488858 | predicted gene, 25135 [Source:MGI Symbol;Acc:MGI:5454912] | Gm25135 | snRNA |
| ENSMUSG00000065990 | 1267.06288 | -0.3619087 | 0.15077338 | 0.00292864 | 0.02661705 | Down | 66077 | aurora kinase A interacting protein 1 [Source:MGI Symbol;Acc:MGI:1913327] | Aurkaip1 | protein_coding |
| ENSMUSG00000066026 | 3991.25288 | -0.3641672 | 0.14197015 | 0.00191695 | 0.02020881 | Down | 20148 | dehydrogenase/reductase (SDR family) member 3 [Source:MGI Symbol;Acc:MGI:1315215] | Dhrs3 | protein_coding |
| ENSMUSG00000066043 | 496.784665 | 0.31509157 | 0.1482614 | 0.00747863 | 0.04964493 | Up | 100169 | phosphatase and actin regulator 4 [Source:MGI Symbol;Acc:MGI:2140327] | Phactr4 | protein_coding |
| ENSMUSG00000066232 | 1907.3301 | 0.28616143 | 0.12555485 | 0.00645875 | 0.04472393 | Up | 233726 | importin 7 [Source:MGI Symbol;Acc:MGI:2152414] | Ipo7 | protein_coding |
| ENSMUSG00000066235 | 269.549169 | -0.6149379 | 0.14118334 | 1.03E-06 | 0.00014754 | Down | 215494 | protein O-linked mannose beta 1,4-N-acetylglucosaminyltransferase 2 [Source:MGI Symbol;Acc:MGI:2143424] | Pomgnt2 | protein_coding |
| ENSMUSG00000066315 | 21.3477014 | -0.96226 | 0.49840803 | 0.00196241 | 0.02051433 | Down | NA | predicted gene 12918 [Source:MGI Symbol;Acc:MGI:3652005] | Gm12918 | processed_pseudogene |
| ENSMUSG00000066415 | 791.525315 | 0.36330489 | 0.10646873 | 0.00013572 | 0.00361827 | Up | 77853 | MSL complex subunit 2 [Source:MGI Symbol;Acc:MGI:1925103] | Msl2 | protein_coding |
| ENSMUSG00000066456 | 344.358051 | -0.4308082 | 0.1767347 | 0.00188879 | 0.01998914 | Down | 94353 | high mobility group nucleosomal binding domain 3 [Source:MGI Symbol;Acc:MGI:2138069] | Hmgn3 | protein_coding |
| ENSMUSG00000066607 | 224.032719 | -0.5582712 | 0.19913354 | 0.00046503 | 0.00794361 | Down | 319477 | inhibitory synaptic factor 1 [Source:MGI Symbol;Acc:MGI:2442108] | Insyn1 | protein_coding |
| ENSMUSG00000066798 | 303.427472 | 0.37859564 | 0.1588547 | 0.00280232 | 0.02590538 | Up | 241322 | zinc finger and BTB domain containing 6 [Source:MGI Symbol;Acc:MGI:2442998] | Zbtb6 | protein_coding |
| ENSMUSG00000066877 | 482.071349 | -0.3217054 | 0.10216429 | 0.00032177 | 0.00639795 | Down | 17974 | non-catalytic region of tyrosine kinase adaptor protein 2 [Source:MGI Symbol;Acc:MGI:1306821] | Nck2 | protein_coding |
| ENSMUSG00000066979 | 995.880996 | -0.2682874 | 0.11845001 | 0.00708252 | 0.04783037 | Down | 12237 | BUB3 mitotic checkpoint protein [Source:MGI Symbol;Acc:MGI:1343463] | Bub3 | protein_coding |

| | | | | | | | | | | |
|---|---|---|---|---|---|---|---|---|---|---|
| ENSMUSG00000067071 | 194.435158 | -0.8834901 | 0.22265213 | 4.42E-06 | 0.0003777 | Down | 55927 | hairy and enhancer of split 6 [Source:MGI Symbol;Acc:MGI:1859852] | Hes6 | protein_coding |
| ENSMUSG00000067336 | 2122.75249 | 0.53319953 | 0.12176479 | 1.40E-06 | 0.00018202 | Up | 12168 | bone morphogenetic protein receptor, type II (serine/threonine kinase) [Source:MGI Symbol;Acc:MGI:1095407] | Bmpr2 | protein_coding |
| ENSMUSG00000067377 | 297.415098 | -0.5979416 | 0.2435558 | 0.00099857 | 0.01330743 | Down | 56496 | tetraspanin 6 [Source:MGI Symbol;Acc:MGI:1926264] | Tspan6 | protein_coding |
| ENSMUSG00000067567 | 93.0356277 | -0.5159321 | 0.16291584 | 0.00016357 | 0.00410687 | Down | 70315 | histone deacetylase 8 [Source:MGI Symbol;Acc:MGI:1917565] | Hdac8 | protein_coding |
| ENSMUSG00000067713 | 285.016601 | -0.6261794 | 0.21748551 | 0.00027652 | 0.00577363 | Down | 19082 | protein kinase, AMP-activated, gamma 1 non-catalytic subunit [Source:MGI Symbol;Acc:MGI:108411] | Prkag1 | protein_coding |
| ENSMUSG00000067736 | 248.148745 | 0.67048139 | 0.29526693 | 0.00140752 | 0.01663216 | Up | NA | predicted gene 10222 [Source:MGI Symbol;Acc:MGI:3642643] | Gm10222 | unprocessed_pseudogene |
| ENSMUSG00000067786 | 19108.8701 | -0.5062042 | 0.27644545 | 0.00557231 | 0.04057467 | Down | 18111 | neuronatin [Source:MGI Symbol;Acc:MGI:104716] | Nnat | protein_coding |
| ENSMUSG00000067847 | 603.831996 | -1.1067446 | 0.26857374 | 1.85E-06 | 0.00021781 | Down | 67067 | reactive oxygen species modulator 1 [Source:MGI Symbol;Acc:MGI:1914317] | Romo1 | protein_coding |
| ENSMUSG00000067870 | 82.6324144 | -0.5070923 | 0.23772762 | 0.00293298 | 0.02661802 | Down | NA | ribosomal protein L31, pseudogene 8 [Source:MGI Symbol;Acc:MGI:3647726] | Rpl31-ps8 | processed_pseudogene |
| ENSMUSG00000067925 | 665.793535 | -0.4464695 | 0.21189577 | 0.00419141 | 0.0336513 | Down | 66158 | retrotransposon Gag like 8A [Source:MGI Symbol;Acc:MGI:1913408] | Rtl8a | protein_coding |
| ENSMUSG00000067931 | 146.824907 | 0.43296883 | 0.19863669 | 0.00354097 | 0.03010104 | Up | 381066 | zinc finger protein 948 [Source:MGI Symbol;Acc:MGI:3040683] | Zfp948 | protein_coding |
| ENSMUSG00000067942 | 365.065704 | 0.37309272 | 0.15151485 | 0.00239319 | 0.02333577 | Up | 224585 | zinc finger protein 160 [Source:MGI Symbol;Acc:MGI:108187] | Zfp160 | protein_coding |
| ENSMUSG00000068184 | 155.763638 | -0.7492036 | 0.25348916 | 0.00019153 | 0.00456229 | Down | 75597 | NADH:ubiquinone oxidoreductase complex assembly factor 2 [Source:MGI Symbol;Acc:MGI:1922847] | Ndufaf2 | protein_coding |
| ENSMUSG00000068220 | 20597.2674 | -1.0214786 | 0.30397759 | 3.84E-05 | 0.00161218 | Down | 16852 | lectin, galactose binding, soluble 1 [Source:MGI Symbol;Acc:MGI:96777] | Lgals1 | protein_coding |
| ENSMUSG00000068267 | 4856.13575 | -0.4275528 | 0.15464682 | 0.00079559 | 0.01157063 | Down | 12616 | centromere protein B [Source:MGI Symbol;Acc:MGI:88376] | Cenpb | protein_coding |
| ENSMUSG00000068270 | 295.766297 | 0.54447209 | 0.21399316 | 0.00096491 | 0.01311446 | Up | 208431 | shroom family member 4 [Source:MGI Symbol;Acc:MGI:2685570] | Shroom4 | protein_coding |
| ENSMUSG00000068290 | 1603.82405 | -0.5451721 | 0.23714326 | 0.0017417 | 0.01892572 | Down | 77006 | DDRGK domain containing 1 [Source:MGI Symbol;Acc:MGI:1924256] | Ddrgk1 | protein_coding |
| ENSMUSG00000068335 | 198.575695 | -0.5048881 | 0.2590996 | 0.00387568 | 0.03195959 | Down | 13448 | docking protein 1 [Source:MGI Symbol;Acc:MGI:893587] | Dok1 | protein_coding |
| ENSMUSG00000068391 | 312.083682 | -0.3454646 | 0.14055533 | 0.00274771 | 0.02560015 | Down | 93696 | chromatin accessibility complex 1 [Source:MGI Symbol;Acc:MGI:2135796] | Chrac1 | protein_coding |
| ENSMUSG00000068396 | 14.3389063 | -0.6984137 | 0.42092873 | 0.00438247 | 0.03448783 | Down | 619547 | ribosomal protein L34, pseudogene 1 [Source:MGI Symbol;Acc:MGI:3704270] | Rpl34-ps1 | processed_pseudogene |
| ENSMUSG00000068417 | 100.330137 | -0.7621936 | 0.24945103 | 0.0001403 | 0.00367613 | Down | 667034 | purine-nucleoside phosphorylase 2 [Source:MGI Symbol;Acc:MGI:3712328] | Pnp2 | protein_coding |
| ENSMUSG00000068457 | 539.475766 | 0.36970979 | 0.14711106 | 0.00215579 | 0.02185892 | Up | 22290 | ubiquitously transcribed tetratricopeptide repeat containing, Y-linked [Source:MGI Symbol;Acc:MGI:894810] | Uty | protein_coding |
| ENSMUSG00000068758 | 327.530533 | -0.7485172 | 0.23910928 | 0.00011108 | 0.00311648 | Down | 16188 | interleukin 3 receptor, alpha chain [Source:MGI Symbol;Acc:MGI:96553] | Il3ra | protein_coding |
| ENSMUSG00000068874 | 3808.19609 | -0.4358856 | 0.1262901 | 9.10E-05 | 0.00275215 | Down | 20341 | selenium binding protein 1 [Source:MGI Symbol;Acc:MGI:96825] | Selenbp1 | protein_coding |
| ENSMUSG00000068922 | 330.308153 | -0.3197691 | 0.12897829 | 0.00318877 | 0.028199 | Down | 229524 | misato 1, mitochondrial distribution and morphology regulator [Source:MGI Symbol;Acc:MGI:2385175] | Msto1 | protein_coding |
| ENSMUSG00000068966 | 262.340647 | 0.32508207 | 0.15415537 | 0.0073615 | 0.04911195 | Up | 241311 | zinc finger and BTB domain containing 34 [Source:MGI Symbol;Acc:MGI:2685195] | Zbtb34 | protein_coding |
| ENSMUSG00000069020 | 422.819526 | -0.5013799 | 0.22118317 | 0.00220403 | 0.02212886 | Down | 68205 | ubiquitin related modifier 1 [Source:MGI Symbol;Acc:MGI:1915455] | Urm1 | protein_coding |
| ENSMUSG00000069495 | 542.056448 | 0.35046682 | 0.12777736 | 0.00126745 | 0.01542296 | Up | 227867 | enhancer of polycomb homolog 2 [Source:MGI Symbol;Acc:MGI:1278321] | Epc2 | protein_coding |
| ENSMUSG00000069515 | 1146.06784 | -0.5891746 | 0.3204735 | 0.00384188 | 0.03171305 | Down | 17110 | lysozyme 1 [Source:MGI Symbol;Acc:MGI:96902] | Lyz1 | protein_coding |
| ENSMUSG00000069539 | 730.291068 | 0.30663767 | 0.09914691 | 0.00053152 | 0.00878387 | Up | 213326 | SCY1-like 2 (S. cerevisiae) [Source:MGI Symbol;Acc:MGI:1289172] | Scyl2 | protein_coding |
| ENSMUSG00000069662 | 3694.33645 | -0.3835426 | 0.15321127 | 0.00166651 | 0.01839198 | Down | 17118 | myristoylated alanine rich protein kinase C substrate [Source:MGI Symbol;Acc:MGI:96907] | Marcks | protein_coding |
| ENSMUSG00000070003 | 674.470718 | -0.524523 | 0.29655461 | 0.00537917 | 0.03959432 | Down | 76900 | single stranded DNA binding protein 4 [Source:MGI Symbol;Acc:MGI:1924150] | Ssbp4 | protein_coding |
| ENSMUSG00000070283 | 403.604292 | -0.3946315 | 0.16121245 | 0.00220201 | 0.02212222 | Down | 66706 | NADH:ubiquinone oxidoreductase complex assembly factor 3 [Source:MGI Symbol;Acc:MGI:1913956] | Ndufaf3 | protein_coding |
| ENSMUSG00000070284 | 227.937095 | -0.3642389 | 0.17088482 | 0.0056417 | 0.04080562 | Down | 331026 | GDP-mannose pyrophosphorylase B [Source:MGI Symbol;Acc:MGI:2660880] | Gmppb | protein_coding |
| ENSMUSG00000070306 | 65.6449426 | 0.01143516 | 0.19869523 | 3.36E-09 | 3.04E-06 | Up | 270150 | coiled-coil domain containing 153 [Source:MGI Symbol;Acc:MGI:2448587] | Ccdc153 | protein_coding |
| ENSMUSG00000070319 | 901.762654 | -0.4349763 | 0.19392186 | 0.00297148 | 0.02687731 | Down | 53356 | eukaryotic translation initiation factor 3, subunit G [Source:MGI Symbol;Acc:MGI:1858258] | Eif3g | protein_coding |
| ENSMUSG00000070343 | 39.7051493 | -1.4157241 | 0.29686239 | 9.16E-08 | 2.66E-05 | Down | NA | predicted gene 10288 [Source:MGI Symbol;Acc:MGI:3704227] | Gm10288 | processed_pseudogene |
| ENSMUSG00000070371 | 335.801078 | -0.3632733 | 0.15112876 | 0.0029151 | 0.02655961 | Down | 77613 | protease, serine 36 [Source:MGI Symbol;Acc:MGI:1924863] | Prss36 | protein_coding |
| ENSMUSG00000070436 | 10793.0288 | -0.6030284 | 0.25848583 | 0.00147325 | 0.01705841 | Down | 12406 | serine (or cysteine) peptidase inhibitor, clade H, member 1 [Source:MGI Symbol;Acc:MGI:88283] | Serpinh1 | protein_coding |
| ENSMUSG00000070520 | 318.113098 | -0.4516206 | 0.17077319 | 0.00101462 | 0.01342082 | Down | 66647 | NSE3 homolog, SMC5-SMC6 complex component [Source:MGI Symbol;Acc:MGI:1913897] | Nsmce3 | protein_coding |
| ENSMUSG00000070544 | 1704.64031 | 0.24192295 | 0.08474499 | 0.00185802 | 0.01975364 | Up | 21969 | topoisomerase (DNA) I [Source:MGI Symbol;Acc:MGI:98788] | Top1 | protein_coding |
| ENSMUSG00000070547 | 39.3351177 | 0.91498188 | 0.35777103 | 0.00050513 | 0.00846843 | Up | 233231 | MAS-related GPR, member B1 [Source:MGI Symbol;Acc:MGI:3033115] | Mrgprb1 | protein_coding |
| ENSMUSG00000070953 | 279.971742 | -0.389586 | 0.12522177 | 0.00029303 | 0.00602561 | Down | 227746 | Rab9 effector protein with kelch motifs [Source:MGI Symbol;Acc:MGI:2139530] | Rabepk | protein_coding |
| ENSMUSG00000071014 | 607.144261 | -0.5548932 | 0.28208778 | 0.00340981 | 0.02932366 | Down | 230075 | NADH:ubiquinone oxidoreductase subunit B6 [Source:MGI Symbol;Acc:MGI:2684983] | Ndufb6 | protein_coding |
| ENSMUSG00000071074 | 1473.46628 | -0.4777884 | 0.14444855 | 0.0001232 | 0.00336363 | Down | 28064 | Yip1 domain family, member 3 [Source:MGI Symbol;Acc:MGI:106280] | Yipf3 | protein_coding |
| ENSMUSG00000071076 | 1204.17351 | -0.4617355 | 0.27220199 | 0.00716581 | 0.04816792 | Down | 16478 | jun D proto-oncogene [Source:MGI Symbol;Acc:MGI:96648] | Jund | protein_coding |
| ENSMUSG00000071078 | 16.7393328 | -1.0383619 | 0.5862108 | 0.00243113 | 0.0236206 | Down | 75692 | nuclear receptor 2C2-associated protein [Source:MGI Symbol;Acc:MGI:1922942] | Nr2c2ap | protein_coding |
| ENSMUSG00000071266 | 172.287764 | 0.37356894 | 0.17346418 | 0.00501822 | 0.0379028 | Up | 74149 | zinc finger protein 946 [Source:MGI Symbol;Acc:MGI:1921399] | Zfp946 | protein_coding |
| ENSMUSG00000071415 | 3876.95255 | -0.5591562 | 0.19053387 | 0.000303 | 0.00614505 | Down | 65019 | ribosomal protein L23 [Source:MGI Symbol;Acc:MGI:1929455] | Rpl23 | protein_coding |
| ENSMUSG00000071632 | 215.453756 | -0.4958091 | 0.15562193 | 0.00016297 | 0.00410464 | Down | 72307 | RIKEN cDNA 2510002D24 gene [Source:MGI Symbol;Acc:MGI:1919557] | 2510002D24Rik | protein_coding |
| ENSMUSG00000071637 | 962.5909 | -0.7694697 | 0.30055425 | 0.00056726 | 0.00914197 | Down | 12609 | CCAAT/enhancer binding protein (C/EBP), delta [Source:MGI Symbol;Acc:MGI:103573] | Cebpd | protein_coding |
| ENSMUSG00000071648 | 105.770202 | -0.417626 | 0.21224104 | 0.00580032 | 0.04160095 | Down | 19881 | rod outer segment membrane protein 1 [Source:MGI Symbol;Acc:MGI:97998] | Rom1 | protein_coding |
| ENSMUSG00000071649 | 623.921141 | -0.3930595 | 0.15276176 | 0.00150294 | 0.01730352 | Down | 72727 | beta-1,3-glucuronyltransferase 3 (glucuronosyltransferase I) [Source:MGI Symbol;Acc:MGI:1919977] | B3gat3 | protein_coding |

| | | | | | | | | | | |
|---|---|---|---|---|---|---|---|---|---|---|
| ENSMUSG00000071654 | 339.954418 | -0.8455171 | 0.24104494 | 2.60E-05 | 0.00126867 | Down | 107197 | ubiquinol-cytochrome c reductase complex assembly factor 3 [Source:MGI Symbol;Acc:MGI:2147553] | Uqcc3 | protein_coding |
| ENSMUSG00000071796 | 119.290933 | 0.58632583 | 0.19438029 | 0.00022365 | 0.00504031 | Up | NA | RIKEN cDNA 6820431F20 gene [Source:MGI Symbol;Acc:MGI:3694236] | 6820431F20Rik | transcribed_unprocessed_pseudogene |
| ENSMUSG00000072235 | 16363.3199 | -0.8969744 | 0.33791703 | 0.00037587 | 0.00704267 | Down | 22142 | tubulin, alpha 1A [Source:MGI Symbol;Acc:MGI:98869] | Tuba1a | protein_coding |
| ENSMUSG00000072501 | 1009.86069 | 0.31558653 | 0.11471617 | 0.00150171 | 0.01730159 | Up | 239510 | PHD finger protein 20-like 1 [Source:MGI Symbol;Acc:MGI:2444412] | Phf20l1 | protein_coding |
| ENSMUSG00000072653 | 216.801365 | 0.35655542 | 0.17183154 | 0.00648655 | 0.04482092 | Up | NA | zinc finger protein 783 [Source:MGI Symbol;Acc:MGI:3040704] | Zfp783 | transcribed_unprocessed_pseudogene |
| ENSMUSG00000072769 | 94.5045886 | 0.97099647 | 0.3068556 | 7.95E-05 | 0.00251852 | Up | NA | predicted gene 10419 [Source:MGI Symbol;Acc:MGI:3642823] | Gm10419 | lncRNA |
| ENSMUSG00000072774 | 63.8486 | 0.58385559 | 0.22778741 | 0.00083929 | 0.01191802 | Up | 626391 | zinc finger protein 951 [Source:MGI Symbol;Acc:MGI:2441896] | Zfp951 | protein_coding |
| ENSMUSG00000072875 | 171.584633 | -0.8820876 | 0.21480613 | 2.62E-06 | 0.00028051 | Down | 14761 | G protein-coupled receptor 27 [Source:MGI Symbol;Acc:MGI:1202299] | Gpr27 | protein_coding |
| ENSMUSG00000072941 | 11560.2606 | -0.8433167 | 0.18116776 | 2.26E-07 | 5.32E-05 | Down | 20657 | superoxide dismutase 3, extracellular [Source:MGI Symbol;Acc:MGI:103181] | Sod3 | protein_coding |
| ENSMUSG00000073155 | 1720.71029 | -0.4207272 | 0.18862579 | 0.00335188 | 0.02902525 | Down | 67705 | RIKEN cDNA 1810058I24 gene [Source:MGI Symbol;Acc:MGI:1914955] | 1810058I24Rik | protein_coding |
| ENSMUSG00000073176 | 210.116635 | 0.39726302 | 0.17115461 | 0.00301232 | 0.02708091 | Up | 78619 | zinc finger protein 449 [Source:MGI Symbol;Acc:MGI:1925869] | Zfp449 | protein_coding |
| ENSMUSG00000073209 | 151.339129 | -0.9027505 | 0.47466673 | 0.00205039 | 0.02112144 | Down | 619665 | Kruppel-like factor 14 [Source:MGI Symbol;Acc:MGI:3577024] | Klf14 | protein_coding |
| ENSMUSG00000073411 | 25728.2444 | -0.4450551 | 0.15633018 | 0.00057455 | 0.00921372 | Down | 14964 | histocompatibility 2, D region locus 1 [Source:MGI Symbol;Acc:MGI:95896] | H2-D1 | protein_coding |
| ENSMUSG00000073418 | 25602.3042 | -0.5488886 | 0.19337392 | 0.00041445 | 0.00741488 | Down | 12268 | complement component 4B (Chido blood group) [Source:MGI Symbol;Acc:MGI:88228] | C4b | protein_coding |
| ENSMUSG00000073421 | 4746.07361 | -0.6026296 | 0.30241587 | 0.0029296 | 0.02661705 | Down | 14961 | histocompatibility 2, class II antigen A, beta 1 [Source:MGI Symbol;Acc:MGI:103070] | H2-Ab1 | protein_coding |
| ENSMUSG00000073616 | 495.654742 | -0.5811731 | 0.28989449 | 0.00295068 | 0.02671892 | Down | 66915 | COP9 signalosome subunit 9 [Source:MGI Symbol;Acc:MGI:1914165] | Cops9 | protein_coding |
| ENSMUSG00000073643 | 263.03482 | 0.425001 | 0.13110024 | 0.00018107 | 0.00441012 | Up | 69368 | WD repeat and FYVE domain containing 1 [Source:MGI Symbol;Acc:MGI:1916618] | Wdfy1 | protein_coding |
| ENSMUSG00000073647 | 49.4617185 | 0.78145461 | 0.30265758 | 0.00055373 | 0.00897737 | Up | NA | predicted gene 10557 [Source:MGI Symbol;Acc:MGI:3708638] | Gm10557 | processed_pseudogene |
| ENSMUSG00000073664 | 1334.87845 | 0.37126801 | 0.13592421 | 0.00110615 | 0.01418587 | Up | 269198 | neurobeachin like 1 [Source:MGI Symbol;Acc:MGI:2444343] | Nbeal1 | protein_coding |
| ENSMUSG00000073676 | 1048.41302 | -0.4574419 | 0.23993022 | 0.00557026 | 0.04057467 | Down | 15528 | heat shock protein 1 (chaperonin 10) [Source:MGI Symbol;Acc:MGI:104680] | Hspe1 | protein_coding |
| ENSMUSG00000073684 | 489.895954 | -0.5248598 | 0.13886761 | 1.73E-05 | 0.00097817 | Down | 67513 | Fanconi anemia core complex associated protein 20 [Source:MGI Symbol;Acc:MGI:1914763] | Faap20 | protein_coding |
| ENSMUSG00000073702 | 3619.81894 | -0.4819994 | 0.14599586 | 0.00010064 | 0.00291573 | Down | 114641 | ribosomal protein L31 [Source:MGI Symbol;Acc:MGI:2149632] | Rpl31 | protein_coding |
| ENSMUSG00000073705 | 9.03181851 | -1.4960162 | 0.66529165 | 0.00091849 | 0.01269858 | Down | 69928 | centromere protein S [Source:MGI Symbol;Acc:MGI:1917178] | Cenps | protein_coding |
| ENSMUSG00000073792 | 137.72633 | 0.39165612 | 0.1421578 | 0.00096154 | 0.01311446 | Up | 320438 | asparagine-linked glycosylation 6 (alpha-1,3,-glucosyltransferase) [Source:MGI Symbol;Acc:MGI:2444031] | Alg6 | protein_coding |
| ENSMUSG00000073802 | 283.686388 | -0.828893 | 0.39362845 | 0.00152137 | 0.01741052 | Down | 12579 | cyclin dependent kinase inhibitor 2B [Source:MGI Symbol;Acc:MGI:104737] | Cdkn2b | protein_coding |
| ENSMUSG00000073968 | 510.280123 | -0.2888325 | 0.1004237 | 0.00097831 | 0.01322185 | Down | 101700 | tripartite motif-containing 68 [Source:MGI Symbol;Acc:MGI:2142077] | Trim68 | protein_coding |
| ENSMUSG00000073985 | 48.5587703 | 0.48287272 | 0.23291215 | 0.00375205 | 0.03126921 | Up | NA | predicted gene 10602 [Source:MGI Symbol;Acc:MGI:3708759] | Gm10602 | lncRNA |
| ENSMUSG00000074064 | 981.141569 | -0.3581998 | 0.13825972 | 0.00194666 | 0.02045506 | Down | 56690 | malonyl-CoA decarboxylase [Source:MGI Symbol;Acc:MGI:1928485] | Mlycd | protein_coding |
| ENSMUSG00000074165 | 206.37814 | 0.41247617 | 0.16304279 | 0.00166174 | 0.01836389 | Up | 67607 | zinc finger protein 788 [Source:MGI Symbol;Acc:MGI:1914857] | Zfp788 | protein_coding |
| ENSMUSG00000074178 | 24.7823496 | -1.3111274 | 0.46595422 | 0.00019314 | 0.00456711 | Down | NA | predicted gene 10638 [Source:MGI Symbol;Acc:MGI:3704316] | Gm10638 | lncRNA |
| ENSMUSG00000074227 | 506.233818 | -0.5699049 | 0.2885713 | 0.00316232 | 0.02802613 | Down | 20733 | serine protease inhibitor, Kunitz type 2 [Source:MGI Symbol;Acc:MGI:1338031] | Spint2 | protein_coding |
| ENSMUSG00000074247 | 1215.72928 | -0.6276657 | 0.11736949 | 8.66E-09 | 5.41E-06 | Down | 66498 | DET1 and DDB1 associated 1 [Source:MGI Symbol;Acc:MGI:1913748] | Dda1 | protein_coding |
| ENSMUSG00000074269 | 19.8868821 | 0.76359512 | 0.46366648 | 0.0039573 | 0.03238603 | Up | 73673 | REC114 meiotic recombination protein [Source:MGI Symbol;Acc:MGI:1920923] | Rec114 | protein_coding |
| ENSMUSG00000074415 | 715.234013 | 0.53922343 | 0.20059017 | 0.00068017 | 0.01039449 | Up | 73144 | Mir100 Mirlet7a-2 Mir125b-1 cluster host gene [Source:MGI Symbol;Acc:MGI:1920394] | Mir100hg | lncRNA |
| ENSMUSG00000074457 | 1323.92593 | -0.5568994 | 0.20720794 | 0.00061049 | 0.00960103 | Down | 67860 | S100 calcium binding protein A16 [Source:MGI Symbol;Acc:MGI:1915110] | S100a16 | protein_coding |
| ENSMUSG00000074576 | 198.047165 | -0.7929701 | 0.17281336 | 3.07E-07 | 6.47E-05 | Down | 69372 | molybdenum cofactor synthesis 3 [Source:MGI Symbol;Acc:MGI:1916622] | Mocs3 | protein_coding |
| ENSMUSG00000074698 | 1799.31047 | 0.21708035 | 0.04871851 | 3.93E-06 | 0.00036162 | Up | 12995 | casein kinase 2, alpha 1 polypeptide [Source:MGI Symbol;Acc:MGI:88543] | Csnk2a1 | protein_coding |
| ENSMUSG00000074796 | 47.8673664 | 0.67210746 | 0.37584645 | 0.00359495 | 0.03038501 | Up | 269356 | solute carrier family 4, sodium bicarbonate transporter-like, member 11 [Source:MGI Symbol;Acc:MGI:2138987] | Slc4a11 | protein_coding |
| ENSMUSG00000074811 | 153.075036 | -0.4709459 | 0.19985166 | 0.00185489 | 0.01974618 | Down | 20170 | HPS6, biogenesis of lysosomal organelles complex 2 subunit 3 [Source:MGI Symbol;Acc:MGI:2181763] | Hps6 | protein_coding |
| ENSMUSG00000074884 | 1530.27407 | -1.3288236 | 0.31248109 | 9.89E-07 | 0.00014478 | Down | 378702 | small EDRK-rich factor 2 [Source:MGI Symbol;Acc:MGI:1337041] | Serf2 | protein_coding |
| ENSMUSG00000074922 | 291.020263 | -0.3406739 | 0.11033078 | 0.00041678 | 0.00743736 | Down | 68034 | family with sequence similarity 122, member A [Source:MGI Symbol;Acc:MGI:1915284] | Fam122a | protein_coding |
| ENSMUSG00000074968 | 7.84469181 | 0.20508134 | 0.38196154 | 0.0064538 | 0.04471933 | Up | 228432 | anoctamin 3 [Source:MGI Symbol;Acc:MGI:3613666] | Ano3 | protein_coding |
| ENSMUSG00000074994 | 640.637346 | 0.47198595 | 0.17801056 | 0.0008802 | 0.01237994 | Up | 99003 | glutamine and serine rich 1 [Source:MGI Symbol;Acc:MGI:2138986] | Qser1 | protein_coding |
| ENSMUSG00000075012 | 34.6842904 | -0.6463698 | 0.43234018 | 0.00582683 | 0.0417218 | Down | 14221 | four jointed box 1 [Source:MGI Symbol;Acc:MGI:1341907] | Fjx1 | protein_coding |
| ENSMUSG00000075227 | 390.195307 | -0.7984762 | 0.17467863 | 3.43E-07 | 7.05E-05 | Down | 29805 | zinc finger, HIT domain containing 2 [Source:MGI Symbol;Acc:MGI:1352481] | Znhit2 | protein_coding |
| ENSMUSG00000075296 | 639.8035 | -0.4647567 | 0.23676766 | 0.00489169 | 0.03727269 | Down | 621603 | aldehyde dehydrogenase 3 family, member B2 [Source:MGI Symbol;Acc:MGI:2147613] | Aldh3b2 | protein_coding |
| ENSMUSG00000075324 | 112.853692 | 0.90557039 | 0.29273655 | 0.00010724 | 0.00305101 | Up | 60344 | fidgetin [Source:MGI Symbol;Acc:MGI:1890647] | Fign | protein_coding |
| ENSMUSG00000075376 | 624.753426 | 0.63055786 | 0.12167104 | 2.15E-08 | 9.39E-06 | Up | 319817 | ring finger and CCCH-type zinc finger domains 2 [Source:MGI Symbol;Acc:MGI:2442789] | Rc3h2 | protein_coding |
| ENSMUSG00000075467 | 678.293297 | -0.6785223 | 0.18948058 | 2.41E-05 | 0.00120579 | Down | 52838 | DNL-type zinc finger [Source:MGI Symbol;Acc:MGI:106559] | Dnlz | protein_coding |
| ENSMUSG00000075470 | 1341.01157 | 0.23678263 | 0.09331576 | 0.00443346 | 0.03479306 | Up | 380959 | asparagine-linked glycosylation 10B (alpha-1,2-glucosyltransferase) [Source:MGI Symbol;Acc:MGI:2146159] | Alg10b | protein_coding |
| ENSMUSG00000075702 | 468.451589 | -0.5509423 | 0.20964348 | 0.00075093 | 0.01114106 | Down | 114679 | selenoprotein M [Source:MGI Symbol;Acc:MGI:2149786] | Selenom | protein_coding |
| ENSMUSG00000076540 | 9.8160328 | 0.00746939 | 0.19841294 | 3.33E-05 | 0.00147973 | Up | NA | immunoglobulin kappa variable 4-80 [Source:MGI Symbol;Acc:MGI:4439653] | Igkv4-80 | IG_V_gene |
| ENSMUSG00000076594 | 11.0152963 | -0.0039938 | 0.1982708 | 0.00159655 | 0.01788111 | Down | NA | immunoglobulin kappa variable 6-13 [Source:MGI Symbol;Acc:MGI:1330829] | Igkv6-13 | IG_V_gene |
| ENSMUSG00000078153 | 92.3139378 | -0.4662186 | 0.24108526 | 0.00505024 | 0.03798203 | Down | 621823 | protease (prosome, macropain) activator subunit 2B [Source:MGI Symbol;Acc:MGI:1341073] | Psme2b | protein_coding |

| | | | | | | | | | | |
|---|---|---|---|---|---|---|---|---|---|---|
| ENSMUSG00000078193 | 81.9585833 | -0.6981131 | 0.31041197 | 0.00137031 | 0.0163561 | Down | NA | predicted gene 2000 [Source:MGI Symbol;Acc:MGI:3780170] | Gm2000 | protein_coding |
| ENSMUSG00000078201 | 183.608718 | -0.3754786 | 0.16832865 | 0.00421889 | 0.03379483 | Down | 227615 | transmembrane protein 203 [Source:MGI Symbol;Acc:MGI:2443597] | Tmem203 | protein_coding |
| ENSMUSG00000078238 | 30.4112841 | -0.7224609 | 0.31382505 | 0.00120883 | 0.01502486 | Down | NA | predicted gene 12854 [Source:MGI Symbol;Acc:MGI:3650410] | Gm12854 | processed_pseudogene |
| ENSMUSG00000078317 | 228.751787 | -0.6295012 | 0.16911608 | 1.80E-05 | 0.0010079 | Down | 14070 | factor 8-associated gene A [Source:MGI Symbol;Acc:MGI:95474] | F8a | protein_coding |
| ENSMUSG00000078348 | 841.379677 | -1.0846387 | 0.25823019 | 1.41E-06 | 0.00018218 | Down | 66125 | splicing factor 3b, subunit 5 [Source:MGI Symbol;Acc:MGI:1913375] | Sf3b5 | protein_coding |
| ENSMUSG00000078440 | 158.141479 | -0.7734437 | 0.31585615 | 0.00075845 | 0.01122126 | Down | 102115 | deoxyhypusine hydroxylase/monooxygenase [Source:MGI Symbol;Acc:MGI:1915964] | Dohh | protein_coding |
| ENSMUSG00000078572 | 204.548574 | -0.5066452 | 0.21278043 | 0.00163852 | 0.01819392 | Down | 208501 | NADH:ubiquinone oxidoreductase complex assembly factor 8 [Source:MGI Symbol;Acc:MGI:1913676] | Ndufaf8 | protein_coding |
| ENSMUSG00000078640 | 13.4233268 | -1.0907709 | 0.66353196 | 0.00291941 | 0.02656911 | Down | NA | predicted gene 11627 [Source:MGI Symbol;Acc:MGI:3650659] | Gm11627 | protein_coding |
| ENSMUSG00000078713 | 430.345672 | -0.6977345 | 0.18679114 | 1.41E-05 | 0.00086456 | Down | 68512 | translocase of outer mitochondrial membrane 5 [Source:MGI Symbol;Acc:MGI:1915762] | Tomm5 | protein_coding |
| ENSMUSG00000078812 | 5986.09631 | -0.3721423 | 0.13835502 | 0.00124709 | 0.01526747 | Down | 276770 | eukaryotic translation initiation factor 5A [Source:MGI Symbol;Acc:MGI:106248] | Eif5a | protein_coding |
| ENSMUSG00000078851 | 60.3055471 | -0.4958687 | 0.29498544 | 0.00678939 | 0.04640038 | Down | 319162 | H2A.W histone [Source:MGI Symbol;Acc:MGI:2448458] | H2aw | protein_coding |
| ENSMUSG00000078919 | 172.584517 | 0.63141655 | 0.21378239 | 0.00024414 | 0.00535957 | Up | 13480 | dolichol-phosphate (beta-D) mannosyltransferase 1 [Source:MGI Symbol;Acc:MGI:1330239] | Dpm1 | protein_coding |
| ENSMUSG00000079003 | 289.083202 | -0.4318438 | 0.20073236 | 0.00377084 | 0.03134966 | Down | 666704 | sterile alpha motif domain containing 1 [Source:MGI Symbol;Acc:MGI:2142433] | Samd1 | protein_coding |
| ENSMUSG00000079013 | 12.6878134 | 1.01760613 | 0.6926365 | 0.00378522 | 0.03139658 | Up | 238395 | serine (or cysteine) peptidase inhibitor, clade A (alpha-1 antiproteinase, antitrypsin), member 3J [Source:MGI Symbol;Acc:MGI:2182843] | Serpina3j | protein_coding |
| ENSMUSG00000079037 | 9276.94718 | -0.3913485 | 0.15185017 | 0.00151978 | 0.01741052 | Down | 19122 | prion protein [Source:MGI Symbol;Acc:MGI:97769] | Prnp | protein_coding |
| ENSMUSG00000079105 | 2016.45884 | 0.90767782 | 1.26752589 | 0.00621152 | 0.04360395 | Up | 109828 | complement component 7 [Source:MGI Symbol;Acc:MGI:88235] | C7 | protein_coding |
| ENSMUSG00000079139 | 415.830734 | -0.381875 | 0.16459275 | 0.00321693 | 0.02832928 | Down | NA | predicted gene 4204 [Source:MGI Symbol;Acc:MGI:3782381] | Gm4204 | processed_pseudogene |
| ENSMUSG00000079197 | 1499.77681 | -0.4900101 | 0.19907605 | 0.00146032 | 0.01698185 | Down | 19188 | proteasome (prosome, macropain) activator subunit 2 (PA28 beta) [Source:MGI Symbol;Acc:MGI:1096365] | Psme2 | protein_coding |
| ENSMUSG00000079427 | 152.880964 | -0.6965603 | 0.20112514 | 3.91E-05 | 0.00161915 | Down | 100039707 | 5, 10-methenyltetrahydrofolate synthetase-like [Source:MGI Symbol;Acc:MGI:3780550] | Mthfsl | protein_coding |
| ENSMUSG00000079457 | 147.637326 | -1.7286124 | 0.67134174 | 0.00030811 | 0.00618692 | Down | 665378 | predicted pseudogene 7609 [Source:MGI Symbol;Acc:MGI:3644536] | Gm7609 | protein_coding |
| ENSMUSG00000079478 | 226.130025 | -0.810316 | 0.22897409 | 2.43E-05 | 0.00120656 | Down | 56390 | zinc ribbon domain containing 2 [Source:MGI Symbol;Acc:MGI:1913482] | Znrd2 | protein_coding |
| ENSMUSG00000079480 | 116.845646 | -0.6230927 | 0.39976175 | 0.00549157 | 0.04018491 | Down | 69713 | peptidyl-prolyl cis/trans isomerase, NIMA-interacting, 4 (parvulin) [Source:MGI Symbol;Acc:MGI:1916963] | Pin4 | protein_coding |
| ENSMUSG00000079484 | 74.1143408 | 0.48805769 | 0.28165844 | 0.00661475 | 0.04549394 | Up | 227696 | phytanoyl-CoA dioxygenase domain containing 1 [Source:MGI Symbol;Acc:MGI:3612860] | Phyhd1 | protein_coding |
| ENSMUSG00000079509 | 572.508437 | 0.40005936 | 0.14178146 | 0.00078529 | 0.01147214 | Up | 22764 | zinc finger protein X-linked [Source:MGI Symbol;Acc:MGI:99211] | Zfx | protein_coding |
| ENSMUSG00000079523 | 1437.09935 | -1.0482998 | 0.49689431 | 0.00119676 | 0.01493198 | Down | 19240 | thymosin, beta 10 [Source:MGI Symbol;Acc:MGI:109146] | Tmsb10 | protein_coding |
| ENSMUSG00000079547 | 515.09677 | -0.6420843 | 0.37960149 | 0.00432712 | 0.0342731 | Down | 14999 | histocompatibility 2, class II, locus Mb1 [Source:MGI Symbol;Acc:MGI:95922] | H2-DMb1 | protein_coding |
| ENSMUSG00000079553 | 26.8505629 | -0.7919809 | 0.707988 | 0.00636573 | 0.04436348 | Down | 100502766 | kinesin family member C1 [Source:MGI Symbol;Acc:MGI:109596] | Kifc1 | protein_coding |
| ENSMUSG00000079557 | 2216.88856 | -0.5001859 | 0.11789822 | 2.58E-06 | 0.00027895 | Down | 224703 | membrane associated ring-CH-type finger 2 [Source:MGI Symbol;Acc:MGI:1925915] | Marchf2 | protein_coding |
| ENSMUSG00000079593 | 158.291401 | -0.6874662 | 0.21950745 | 0.0001215 | 0.00333402 | Down | 71159 | RIKEN cDNA 4933416I08 gene [Source:MGI Symbol;Acc:MGI:1918409] | 4933416I08Rik | protein_coding |
| ENSMUSG00000079614 | 1192.04383 | 0.22688086 | 0.09191014 | 0.00595774 | 0.04231895 | Up | 72124 | SEH1-like (S. cerevisiae [Source:MGI Symbol;Acc:MGI:1919374] | Seh1l | protein_coding |
| ENSMUSG00000079645 | 21.6091676 | 0.95204697 | 0.41875941 | 0.00100463 | 0.01334051 | Up | NA | predicted gene 17193 [Source:MGI Symbol;Acc:MGI:4938020] | Gm17193 | lncRNA |
| ENSMUSG00000079658 | 807.029843 | -0.3388699 | 0.1618243 | 0.00666442 | 0.04571935 | Down | 67923 | elongin C [Source:MGI Symbol;Acc:MGI:1915173] | Eloc | protein_coding |
| ENSMUSG00000080268 | 329.545155 | -0.5467497 | 0.12759952 | 2.10E-06 | 0.00024018 | Down | 107392 | breast cancer metastasis-suppressor 1 [Source:MGI Symbol;Acc:MGI:2388804] | Brms1 | protein_coding |
| ENSMUSG00000080848 | 30.0816694 | -0.7088058 | 0.29428729 | 0.00099817 | 0.01330743 | Down | NA | predicted pseudogene 9385 [Source:MGI Symbol;Acc:MGI:3646182] | Gm9385 | processed_pseudogene |
| ENSMUSG00000080875 | 22.7268498 | -0.8701757 | 0.38471287 | 0.00108262 | 0.01400251 | Down | NA | predicted gene 7332 [Source:MGI Symbol;Acc:MGI:3643344] | Gm7332 | processed_pseudogene |
| ENSMUSG00000080921 | 26.0222175 | -0.7085791 | 0.51509757 | 0.00592092 | 0.04211266 | Down | NA | ribosomal protein L38, pseudogene 2 [Source:MGI Symbol;Acc:MGI:3646625] | Rpl38-ps2 | processed_pseudogene |
| ENSMUSG00000081051 | 37.6319803 | -0.8443189 | 0.28152173 | 0.00015109 | 0.00389592 | Down | NA | predicted pseudogene 15427 [Source:MGI Symbol;Acc:MGI:3642341] | Gm15427 | processed_pseudogene |
| ENSMUSG00000081058 | 146.591726 | -1.5170886 | 0.33169902 | 2.24E-07 | 5.32E-05 | Down | 97114 | H3 clustered histone 15 [Source:MGI Symbol;Acc:MGI:2448357] | H3c15 | protein_coding |
| ENSMUSG00000081189 | 402.864661 | -0.6147615 | 0.26820642 | 0.00146705 | 0.01700975 | Down | NA | heat shock protein 1 (chaperonin), pseudogene 4 [Source:MGI Symbol;Acc:MGI:3649750] | Hspd1-ps4 | processed_pseudogene |
| ENSMUSG00000081534 | 5218.25748 | -0.457702 | 0.15755317 | 0.00045329 | 0.00781704 | Down | 67739 | solute carrier family 48 (heme transporter), member 1 [Source:MGI Symbol;Acc:MGI:1914989] | Slc48a1 | protein_coding |
| ENSMUSG00000082284 | 27.4496898 | -0.9725333 | 0.48595841 | 0.00159824 | 0.01788111 | Down | NA | H3.3 histone A, pseudogene 1 [Source:MGI Symbol;Acc:MGI:1101782] | H3f3a-ps1 | processed_pseudogene |
| ENSMUSG00000083282 | 1318.9579 | -0.6365118 | 0.12739189 | 4.83E-08 | 1.63E-05 | Down | 56464 | cathepsin F [Source:MGI Symbol;Acc:MGI:1861434] | Ctsf | protein_coding |
| ENSMUSG00000083443 | 455.51839 | -0.5272156 | 0.25059042 | 0.00285528 | 0.02623535 | Down | NA | predicted gene 15519 [Source:MGI Symbol;Acc:MGI:3782965] | Gm15519 | processed_pseudogene |
| ENSMUSG00000083621 | 24.2630292 | -0.9489681 | 0.42235296 | 0.00101655 | 0.01342593 | Down | NA | predicted gene 14586 [Source:MGI Symbol;Acc:MGI:3705507] | Gm14586 | processed_pseudogene |
| ENSMUSG00000083701 | 33.1305613 | -1.8699631 | 0.74260629 | 0.00035863 | 0.00685683 | Down | NA | predicted pseudogene 4912 [Source:MGI Symbol;Acc:MGI:3643140] | Gm4912 | unprocessed_pseudogene |
| ENSMUSG00000083716 | 13.4099119 | -1.0191536 | 0.44032869 | 0.00088763 | 0.01239863 | Down | NA | predicted gene 13436 [Source:MGI Symbol;Acc:MGI:3651005] | Gm13436 | processed_pseudogene |
| ENSMUSG00000084416 | 62.096593 | -0.5901691 | 0.28946705 | 0.00272491 | 0.02545336 | Down | NA | ribosomal protein L10A, pseudogene 1 [Source:MGI Symbol;Acc:MGI:3705885] | Rpl10a-ps1 | processed_pseudogene |
| ENSMUSG00000084799 | 34.5029982 | 0.70010899 | 0.35121445 | 0.00240983 | 0.02344176 | Up | 100380944 | INO80 complex subunit D, opposite strand [Source:MGI Symbol;Acc:MGI:3651861] | Ino80dos | lncRNA |
| ENSMUSG00000084808 | 109.73108 | 0.55534859 | 0.23560723 | 0.00149798 | 0.01727087 | Up | NA | RIKEN cDNA 9430091E24 gene [Source:MGI Symbol;Acc:MGI:1924618] | 9430091E24Rik | lncRNA |
| ENSMUSG00000084817 | 30.5998661 | -1.1728178 | 0.45298439 | 0.0003904 | 0.0071905 | Down | NA | predicted pseudogene 5526 [Source:MGI Symbol;Acc:MGI:3643066] | Gm5526 | processed_pseudogene |
| ENSMUSG00000084834 | 20.4444043 | -1.8540199 | 0.8036947 | 0.00062236 | 0.00972131 | Down | 68306 | RIKEN cDNA 4930565N06 gene [Source:MGI Symbol;Acc:MGI:1915556] | 4930565N06Rik | lncRNA |
| ENSMUSG00000084899 | 21.5140654 | 0.91466733 | 0.6138951 | 0.00398205 | 0.03250675 | Up | NA | predicted gene 15344 [Source:MGI Symbol;Acc:MGI:3708098] | Gm15344 | lncRNA |

| | | | | | | | | | | |
|---|---|---|---|---|---|---|---|---|---|---|
| ENSMUSG00000085042 | 29.8071887 | -1.2209355 | 0.55372568 | 0.00092902 | 0.01282231 | Down | NA | abhydrolase domain containing 11, opposite strand [Source:MGI Symbol;Acc:MGI:1917062] | Abhd11os | transcribed_unitary_pseudogene |
| ENSMUSG00000085185 | 11.1035204 | -1.0825873 | 0.57188827 | 0.00211661 | 0.02155757 | Down | NA | cDNA sequence BC028777 [Source:MGI Symbol;Acc:MGI:3642753] | BC028777 | lncRNA |
| ENSMUSG00000085218 | 8.98340151 | -1.0629812 | 1.06029369 | 0.00643266 | 0.04463843 | Down | 102640562 | expressed sequence BB218582 [Source:MGI Symbol;Acc:MGI:2139448] | BB218582 | lncRNA |
| ENSMUSG00000085334 | 273.168248 | 0.78731626 | 0.18964658 | 2.26E-06 | 0.00024959 | Up | NA | predicted gene 12940 [Source:MGI Symbol;Acc:MGI:3702626] | Gm12940 | lncRNA |
| ENSMUSG00000085347 | 8.05272433 | -0.9330418 | 0.67182603 | 0.0048132 | 0.03688225 | Down | 70162 | hexokinase 1, opposite strand [Source:MGI Symbol;Acc:MGI:1917412] | Hk1os | lncRNA |
| ENSMUSG00000085395 | 12.8828838 | -1.026091 | 0.6858212 | 0.00362763 | 0.03059148 | Down | NA | predicted gene 13056 [Source:MGI Symbol;Acc:MGI:3650725] | Gm13056 | lncRNA |
| ENSMUSG00000085438 | 2584.9542 | 0.22438912 | 0.08954407 | 0.00547121 | 0.04012631 | Up | NA | Opa interacting protein 5, opposite strand 1 [Source:MGI Symbol;Acc:MGI:1913852] | Oip5os1 | lncRNA |
| ENSMUSG00000085519 | 36.6521847 | -2.0952333 | 0.69287062 | 8.25E-05 | 0.00259284 | Down | NA | predicted gene 13703 [Source:MGI Symbol;Acc:MGI:3651307] | Gm13703 | lncRNA |
| ENSMUSG00000085573 | 15.3003778 | 1.19065571 | 0.66536448 | 0.00223008 | 0.02228018 | Up | NA | predicted gene 15418 [Source:MGI Symbol;Acc:MGI:3705275] | Gm15418 | lncRNA |
| ENSMUSG00000085793 | 390.210948 | 0.34155307 | 0.12764388 | 0.00152617 | 0.01741052 | Up | 217708 | lin-52 DREAM MuvB core complex component [Source:MGI Symbol;Acc:MGI:3045391] | Lin52 | protein_coding |
| ENSMUSG00000086003 | 20.2079163 | -0.7869298 | 0.4652328 | 0.00344165 | 0.02948815 | Down | 100039440 | RIKEN cDNA B230206L02 gene [Source:MGI Symbol;Acc:MGI:1924598] | B230206L02Rik | lncRNA |
| ENSMUSG00000086287 | 38.1450515 | -1.021535 | 0.32599269 | 8.40E-05 | 0.00261825 | Down | 100502834 | predicted gene 15972 [Source:MGI Symbol;Acc:MGI:3801965] | Gm15972 | lncRNA |
| ENSMUSG00000086382 | 24.855407 | -0.8608305 | 0.39987246 | 0.00138382 | 0.01647125 | Down | 102633437 | cholinergic receptor, nicotinic, alpha polypeptide 1 (muscle), opposite strand [Source:MGI Symbol;Acc:MGI:3651120] | Chrna1os | lncRNA |
| ENSMUSG00000086460 | 15.2054767 | -1.0852556 | 0.83533543 | 0.00449758 | 0.03509281 | Down | NA | predicted gene 12236 [Source:MGI Symbol;Acc:MGI:3651548] | Gm12236 | lncRNA |
| ENSMUSG00000086546 | 26.8341865 | -1.2761084 | 0.88720463 | 0.00306493 | 0.02744749 | Down | NA | predicted gene 13709 [Source:MGI Symbol;Acc:MGI:3651118] | Gm13709 | lncRNA |
| ENSMUSG00000086784 | 1815.89273 | -0.4468987 | 0.22000999 | 0.00466858 | 0.03604617 | Down | 664994 | isochorismatase domain containing 2a [Source:MGI Symbol;Acc:MGI:3609243] | Isoc2a | protein_coding |
| ENSMUSG00000086841 | 144.195047 | -1.2287813 | 0.39485766 | 7.84E-05 | 0.00249877 | Down | NA | RIKEN cDNA 2410006H16 gene [Source:MGI Symbol;Acc:MGI:1916471] | 2410006H16Rik | lncRNA |
| ENSMUSG00000087174 | 134.029956 | 0.37078673 | 0.16561686 | 0.00417299 | 0.03354292 | Up | 71445 | RIKEN cDNA 5530601H04 gene [Source:MGI Symbol;Acc:MGI:1918695] | 5530601H04Rik | lncRNA |
| ENSMUSG00000087260 | 675.731031 | -0.4177131 | 0.16423552 | 0.00151147 | 0.01736481 | Down | 68576 | late endosomal/lysosomal adaptor, MAPK and MTOR activator 5 [Source:MGI Symbol;Acc:MGI:1915826] | Lamtor5 | protein_coding |
| ENSMUSG00000087354 | 30.6760711 | 0.75430592 | 0.39346337 | 0.00253037 | 0.02420843 | Up | 67394 | RIKEN cDNA 4930404I05 gene [Source:MGI Symbol;Acc:MGI:1914644] | 4930404I05Rik | lncRNA |
| ENSMUSG00000087370 | 478.180698 | 0.54451561 | 0.10383746 | 1.95E-08 | 9.20E-06 | Up | 621976 | transmembrane protein 170B [Source:MGI Symbol;Acc:MGI:3647046] | Tmem170b | protein_coding |
| ENSMUSG00000087396 | 61.7753393 | 0.52517742 | 0.25064995 | 0.0030115 | 0.02708091 | Up | 74396 | RIKEN cDNA 4933407K13 gene [Source:MGI Symbol;Acc:MGI:1921646] | 4933407K13Rik | lncRNA |
| ENSMUSG00000087445 | 94.1407223 | 1.34742615 | 0.2411479 | 1.26E-09 | 1.61E-06 | Up | NA | predicted gene 14286 [Source:MGI Symbol;Acc:MGI:3650190] | Gm14286 | lncRNA |
| ENSMUSG00000087478 | 23.895938 | 1.01800428 | 0.32686619 | 9.59E-05 | 0.00283782 | Up | 75060 | RIKEN cDNA 4930506C21 gene [Source:MGI Symbol;Acc:MGI:1922310] | 4930506C21Rik | lncRNA |
| ENSMUSG00000087590 | 87.3604176 | -0.5881131 | 0.33794021 | 0.00467499 | 0.03606139 | Down | 69749 | erythrocyte membrane protein band 4.1 like 4a, opposite strand [Source:MGI Symbol;Acc:MGI:1916999] | Epb41l4aos | lncRNA |
| ENSMUSG00000087672 | 43.6647868 | 0.96660393 | 0.28957593 | 4.76E-05 | 0.00184042 | Up | NA | predicted gene 15122 [Source:MGI Symbol;Acc:MGI:3705268] | Gm15122 | lncRNA |
| ENSMUSG00000087690 | 10.9720038 | 1.45239458 | 0.66754704 | 0.00116325 | 0.01464591 | Up | NA | predicted gene 16031 [Source:MGI Symbol;Acc:MGI:3801966] | Gm16031 | lncRNA |
| ENSMUSG00000089762 | 154.271245 | -1.1607121 | 0.32038103 | 1.47E-05 | 0.00088884 | Down | 72500 | immediate early response 5-like [Source:MGI Symbol;Acc:MGI:1919750] | Ier5l | protein_coding |
| ENSMUSG00000089768 | 13.4174817 | -0.817391 | 0.7791873 | 0.00656308 | 0.04523432 | Down | 666244 | thymosin beta 15b1 [Source:MGI Symbol;Acc:MGI:3843059] | Tmsb15b1 | protein_coding |
| ENSMUSG00000089810 | 147.789037 | 0.5101196 | 0.17721234 | 0.00042367 | 0.00750544 | Up | NA | predicted gene 16536 [Source:MGI Symbol;Acc:MGI:4414956] | Gm16536 | lncRNA |
| ENSMUSG00000089872 | 415.766214 | -0.3037169 | 0.13232442 | 0.00544087 | 0.03995793 | Down | 320119 | ribosomal protein S6 kinase polypeptide 1 [Source:MGI Symbol;Acc:MGI:2443419] | Rps6kc1 | protein_coding |
| ENSMUSG00000089875 | 90.7785834 | 0.5222283 | 0.16262961 | 0.00013888 | 0.00366247 | Up | NA | ethanol decreased 2 [Source:MGI Symbol;Acc:MGI:1261417] | Etohd2 | lncRNA |
| ENSMUSG00000090015 | 125.122405 | 0.40562262 | 0.15807199 | 0.0015366 | 0.01748044 | Up | 100642166 | predicted gene 15446 [Source:MGI Symbol;Acc:MGI:3709333] | Gm15446 | protein_coding |
| ENSMUSG00000090100 | 252.378518 | 0.69149309 | 0.17529483 | 6.29E-06 | 0.00047968 | Up | 140810 | tau tubulin kinase 2 [Source:MGI Symbol;Acc:MGI:2155779] | Ttbk2 | protein_coding |
| ENSMUSG00000090112 | 670.242969 | 0.34434801 | 0.12417051 | 0.00123669 | 0.0152312 | Up | 268281 | SNF2 histone linker PHD RING helicase [Source:MGI Symbol;Acc:MGI:1917581] | Shprh | protein_coding |
| ENSMUSG00000090186 | 78.2384228 | -1.5671585 | 0.630874 | 0.00041195 | 0.00739578 | Down | NA | predicted gene, 52951 [Source:MGI Symbol;Acc:MGI:6388830] | Gm52951 | unprocessed_pseudogene |
| ENSMUSG00000090213 | 853.761332 | -0.4048234 | 0.13556218 | 0.00046817 | 0.00798777 | Down | 407243 | plasmanylethanolamine desaturase 1 [Source:MGI Symbol;Acc:MGI:2142624] | Peds1 | protein_coding |
| ENSMUSG00000090222 | 76.5654129 | 0.66778949 | 0.36321877 | 0.0033277 | 0.02892378 | Up | NA | interferon activated gene 203, pseudogene [Source:MGI Symbol;Acc:MGI:3840117] | Ifi203-ps | unprocessed_pseudogene |
| ENSMUSG00000090264 | 11.3842901 | 1.10109637 | 0.49446148 | 0.00109159 | 0.01406252 | Up | 108112 | eukaryotic translation initiation factor 4E binding protein 3 [Source:MGI Symbol;Acc:MGI:1270847] | Eif4ebp3 | protein_coding |
| ENSMUSG00000090266 | 374.762418 | -0.5364489 | 0.207261 | 0.00086833 | 0.01226617 | Down | 74319 | methyltransferase like 23 [Source:MGI Symbol;Acc:MGI:1921569] | Mettl23 | protein_coding |
| ENSMUSG00000090622 | 66.5351305 | 1.04299952 | 0.29787929 | 2.40E-05 | 0.00120579 | Up | 320700 | RIKEN cDNA A930033H14 gene [Source:MGI Symbol;Acc:MGI:2444562] | A930033H14Rik | protein_coding |
| ENSMUSG00000090698 | 299.586905 | 1.43767386 | 0.4456158 | 5.06E-05 | 0.0018818 | Up | 381823 | apolipoprotein L domain containing 1 [Source:MGI Symbol;Acc:MGI:2685921] | Apold1 | protein_coding |
| ENSMUSG00000090785 | 39.8501165 | -0.7621138 | 0.37775887 | 0.00203608 | 0.02105422 | Down | NA | predicted gene 17116 [Source:MGI Symbol;Acc:MGI:4937943] | Gm17116 | lncRNA |
| ENSMUSG00000090841 | 6879.35264 | -0.7262551 | 0.21721636 | 5.97E-05 | 0.0020692 | Down | 17904 | myosin, light polypeptide 6, alkali, smooth muscle and non-muscle [Source:MGI Symbol;Acc:MGI:109318] | Myl6 | protein_coding |
| ENSMUSG00000090862 | 3378.93122 | -0.6119613 | 0.17852722 | 5.31E-05 | 0.00193347 | Down | 68052 | ribosomal protein S13 [Source:MGI Symbol;Acc:MGI:1915302] | Rps13 | protein_coding |
| ENSMUSG00000091199 | 78.343337 | -1.3250863 | 0.83810438 | 0.00244944 | 0.02374113 | Down | NA | predicted gene 2619 [Source:MGI Symbol;Acc:MGI:3780787] | Gm2619 | lncRNA |
| ENSMUSG00000091243 | 1577.51922 | -0.8202304 | 0.30991549 | 0.00041777 | 0.00744158 | Down | 73569 | vestigial like family member 3 [Source:MGI Symbol;Acc:MGI:1920819] | Vgll3 | protein_coding |
| ENSMUSG00000091337 | 896.358244 | -0.3776557 | 0.15887596 | 0.00293724 | 0.02662691 | Down | 58521 | EP300 interacting inhibitor of differentiation 1 [Source:MGI Symbol;Acc:MGI:1889651] | Eid1 | protein_coding |
| ENSMUSG00000091475 | 26.0311909 | -1.0527207 | 0.3429496 | 0.0001078 | 0.00305615 | Down | 72834 | cytoplasmic endogenous regulator of oxidative phosphorylation 1 [Source:MGI Symbol;Acc:MGI:1920084] | Cerox1 | lncRNA |
| ENSMUSG00000091534 | 10.4307173 | 0.84121764 | 0.57966965 | 0.00485834 | 0.0370882 | Up | NA | predicted gene 17195 [Source:MGI Symbol;Acc:MGI:4938022] | Gm17195 | processed_pseudogene |
| ENSMUSG00000091537 | 948.994591 | -0.483676 | 0.15836787 | 0.00026398 | 0.00562038 | Down | 115487184 | translational machinery associated 7 [Source:MGI Symbol;Acc:MGI:1913417] | Tma7 | protein_coding |
| ENSMUSG00000091803 | 125.792279 | 0.89066497 | 0.40022259 | 0.00114059 | 0.01446433 | Up | 66272 | cytochrome c oxidase assembly protein 16 [Source:MGI Symbol;Acc:MGI:1913522] | Cox16 | protein_coding |
| ENSMUSG00000091845 | 11.6208422 | -1.1418592 | 0.44196556 | 0.00042516 | 0.00751546 | Down | NA | ribosomal protein L36, pseudogene 12 [Source:MGI Symbol;Acc:MGI:3782787] | Rpl36-ps12 | processed_pseudogene |
| ENSMUSG00000091955 | 43.6424798 | -1.2911854 | 0.38925001 | 3.99E-05 | 0.00163653 | Down | NA | predicted pseudogene 9844 [Source:MGI Symbol;Acc:MGI:3704288] | Gm9844 | protein_coding |
| ENSMUSG00000092074 | 43.5762163 | -0.6631551 | 0.3611685 | 0.00338909 | 0.02923832 | Down | 100310872 | dynein light chain Tctex-type 1A [Source:MGI Symbol;Acc:MGI:3807506] | Dynlt1a | protein_coding |
| ENSMUSG00000092981 | 200.812975 | 0.73036109 | 0.2518815 | 0.00023782 | 0.00527371 | Up | 100628593 | microRNA 5125 [Source:MGI Symbol;Acc:MGI:4950450] | Mir5125 | miRNA |
| ENSMUSG00000093577 | 134.247432 | -0.5986347 | 0.26852699 | 0.00172809 | 0.01881773 | Down | NA | predicted gene 20632 [Source:MGI Symbol;Acc:MGI:5313079] | Gm20632 | lncRNA |
| ENSMUSG00000093661 | 1018.78925 | 0.3266871 | 0.09937195 | 0.00025057 | 0.00545876 | Up | 66892 | eukaryotic translation initiation factor 4E member 3 [Source:MGI Symbol;Acc:MGI:1914142] | Eif4e3 | protein_coding |
| ENSMUSG00000093674 | 5246.0824 | -0.5980072 | 0.2734097 | 0.00216396 | 0.02189366 | Down | 67945 | ribosomal protein L41 [Source:MGI Symbol;Acc:MGI:1915195] | Rpl41 | protein_coding |

| | | | | | | | | | | |
|---|---|---|---|---|---|---|---|---|---|---|
| ENSMUSG00000093803 | 641.066905 | -0.4414609 | 0.18234127 | 0.00182195 | 0.01954925 | Down | 19054 | protein phosphatase 2 (formerly 2A), regulatory subunit B'', delta [Source:MGI Symbol;Acc:MGI:1335093] | Ppp2r3d | protein_coding |
| ENSMUSG00000094392 | 36.7327655 | -1.1453877 | 0.4054301 | 0.00020068 | 0.00468402 | Down | NA | predicted gene 3788 [Source:MGI Symbol;Acc:MGI:3781961] | Gm3788 | processed_pseudo gene |
| ENSMUSG00000095098 | 502.85467 | -0.9163165 | 0.26715371 | 3.61E-05 | 0.00154455 | Down | 240514 | coiled-coil domain containing 85B [Source:MGI Symbol;Acc:MGI:2147607] | Ccdc85b | protein_coding |
| ENSMUSG00000095123 | 177.210166 | 0.69863427 | 0.24883175 | 0.00032641 | 0.00647569 | Up | NA | predicted gene, 21781 [Source:MGI Symbol;Acc:MGI:5433945] | Gm21781 | lncRNA |
| ENSMUSG00000095677 | 108.42334 | -0.5780572 | 0.20209525 | 0.00036936 | 0.00696084 | Down | 100040531 | dynein light chain Tctex-type 1F [Source:MGI Symbol;Acc:MGI:3780996] | Dynlt1f | protein_coding |
| ENSMUSG00000095742 | 148.164484 | -1.8758526 | 0.43440999 | 6.60E-07 | 0.00010939 | Down | NA | | | protein_coding |
| ENSMUSG00000096145 | 76.5029075 | -0.7030321 | 0.26912921 | 0.00056056 | 0.00906097 | Down | 27973 | vitamin K epoxide reductase complex, subunit 1 [Source:MGI Symbol;Acc:MGI:106442] | Vkorc1 | protein_coding |
| ENSMUSG00000096255 | 68.0449927 | -0.7365057 | 0.45809076 | 0.00415471 | 0.03342906 | Down | 21648 | dynein light chain Tctex-type 1B [Source:MGI Symbol;Acc:MGI:98643] | Dynlt1b | protein_coding |
| ENSMUSG00000096472 | 180.920532 | -0.6742084 | 0.22667576 | 0.00019815 | 0.00464036 | Down | 12581 | cyclin dependent kinase inhibitor 2D [Source:MGI Symbol;Acc:MGI:105387] | Cdkn2d | protein_coding |
| ENSMUSG00000097032 | 22.1031906 | 1.31348203 | 0.3730106 | 2.17E-05 | 0.00112369 | Up | 319587 | RIKEN cDNA 4930539J05 gene [Source:MGI Symbol;Acc:MGI:1922477] | 4930539J05Rik | lncRNA |
| ENSMUSG00000097042 | 134.072043 | 0.91529869 | 0.26717436 | 3.43E-05 | 0.00149515 | Up | NA | predicted gene, 17491 [Source:MGI Symbol;Acc:MGI:4937125] | Gm17491 | lncRNA |
| ENSMUSG00000097099 | 63.0219679 | 0.55364797 | 0.32354427 | 0.00551856 | 0.0403098 | Up | 100038755 | predicted gene 9917 [Source:MGI Symbol;Acc:MGI:3704343] | Gm9917 | lncRNA |
| ENSMUSG00000097124 | 1320.77704 | 0.70378052 | 0.22741881 | 0.00013589 | 0.00361827 | Up | NA | RIKEN cDNA A530020G20 gene [Source:MGI Symbol;Acc:MGI:2442825] | A530020G20Rik | lncRNA |
| ENSMUSG00000097140 | 18.9800498 | 0.86974064 | 0.39399604 | 0.0014098 | 0.0166441 | Up | NA | predicted gene, 26779 [Source:MGI Symbol;Acc:MGI:5477273] | Gm26779 | lncRNA |
| ENSMUSG00000097187 | 54.3287196 | -0.6651424 | 0.3822073 | 0.00425895 | 0.03396482 | Down | NA | predicted gene, 19426 [Source:MGI Symbol;Acc:MGI:5011611] | Gm19426 | protein_coding |
| ENSMUSG00000097231 | 10.5296374 | 1.05786441 | 0.82618741 | 0.00502299 | 0.03791754 | Up | NA | predicted gene, 26852 [Source:MGI Symbol;Acc:MGI:5477346] | Gm26852 | lncRNA |
| ENSMUSG00000097277 | 123.835411 | 0.41348202 | 0.18255342 | 0.00324917 | 0.02851582 | Up | 100504421 | RIKEN cDNA 2900076A07 gene [Source:MGI Symbol;Acc:MGI:1920242] | 2900076A07Rik | lncRNA |
| ENSMUSG00000097286 | 62.5334892 | 0.75519628 | 0.24670534 | 0.00014016 | 0.00367613 | Up | NA | predicted gene, 26684 [Source:MGI Symbol;Acc:MGI:5477178] | Gm26684 | lncRNA |
| ENSMUSG00000097309 | 30.9037784 | 0.58206767 | 0.39866396 | 0.00717055 | 0.04816792 | Up | NA | predicted gene, 17249 [Source:MGI Symbol;Acc:MGI:4936883] | Gm17249 | lncRNA |
| ENSMUSG00000097327 | 47.9992939 | -0.5658478 | 0.25879109 | 0.00217129 | 0.02191828 | Down | 319887 | RIKEN cDNA E030030I06 gene [Source:MGI Symbol;Acc:MGI:2442914] | E030030I06Rik | transcribed_unpro cessed_pseudogen e |
| ENSMUSG00000097339 | 68.2898555 | 0.77672453 | 0.24975724 | 0.00011833 | 0.00327475 | Up | NA | predicted gene, 26671 [Source:MGI Symbol;Acc:MGI:5477165] | Gm26671 | lncRNA |
| ENSMUSG00000097392 | 160.953276 | 0.75815209 | 0.17797798 | 1.48E-06 | 0.00018768 | Up | 100042165 | THO complex subunit 2-like [Source:MGI Symbol;Acc:MGI:3040669] | Thoc2l | protein_coding |
| ENSMUSG00000097415 | 342.278321 | 0.38660446 | 0.17919736 | 0.00464923 | 0.03596513 | Up | 108167440 | expressed sequence AU020206 [Source:MGI Symbol;Acc:MGI:2142134] | AU020206 | lncRNA |
| ENSMUSG00000097654 | 9.05884263 | 1.44657512 | 0.72826679 | 0.00157027 | 0.01772687 | Up | NA | predicted gene, 26714 [Source:MGI Symbol;Acc:MGI:5477208] | Gm26714 | lncRNA |
| ENSMUSG00000097743 | 90.9646435 | 0.62137682 | 0.16627344 | 1.68E-05 | 0.00096154 | Up | 100503823 | predicted gene, 16973 [Source:MGI Symbol;Acc:MGI:4439897] | Gm16973 | lncRNA |
| ENSMUSG00000097762 | 21.1782913 | 1.21660917 | 0.64696714 | 0.0017827 | 0.01924253 | Up | NA | RIKEN cDNA 4732463B04 gene [Source:MGI Symbol;Acc:MGI:3642483] | 4732463B04Rik | lncRNA |
| ENSMUSG00000097887 | 22.0047422 | -0.8175039 | 0.50134628 | 0.00371958 | 0.03108259 | Down | NA | predicted gene, 26542 [Source:MGI Symbol;Acc:MGI:5477036] | Gm26542 | lncRNA |
| ENSMUSG00000097970 | 25.7759382 | 0.8163093 | 0.45936924 | 0.00300479 | 0.02707309 | Up | NA | predicted gene, 27028 [Source:MGI Symbol;Acc:MGI:5504143] | Gm27028 | lncRNA |
| ENSMUSG00000098066 | 24.2676028 | 0.56422548 | 0.36945329 | 0.00717187 | 0.04816792 | Up | NA | predicted gene, 26944 [Source:MGI Symbol;Acc:MGI:5504059] | Gm26944 | lncRNA |
| ENSMUSG00000098098 | 278.244734 | 0.94895997 | 0.3044464 | 9.33E-05 | 0.00280234 | Up | NA | braveheart long non-coding RNA [Source:MGI Symbol;Acc:MGI:5434104] | Bvht | lncRNA |
| ENSMUSG00000098188 | 2238.41376 | 0.53151007 | 0.18062502 | 0.00032682 | 0.00647569 | Up | 268301 | sosondowah ankyrin repeat domain family member C [Source:MGI Symbol;Acc:MGI:3606051] | Sowahc | protein_coding |
| ENSMUSG00000098912 | 70.3517205 | 0.75059955 | 0.21837335 | 4.07E-05 | 0.00165266 | Up | 319830 | RIKEN cDNA 1500004A13 gene [Source:MGI Symbol;Acc:MGI:2442808] | 1500004A13Rik | lncRNA |
| ENSMUSG00000099083 | 1431.3136 | 0.50616739 | 0.17919492 | 0.00053789 | 0.00880853 | Up | 223922 | activating transcription factor 7 [Source:MGI Symbol;Acc:MGI:2443472] | Atf7 | protein_coding |
| ENSMUSG00000099137 | 81.6412178 | 0.51508427 | 0.30596609 | 0.00653786 | 0.04511787 | Up | NA | predicted gene 10603 [Source:MGI Symbol;Acc:MGI:3642592] | Gm10603 | lncRNA |
| ENSMUSG00000099398 | 57.4278797 | 0.81381975 | 0.36666196 | 0.00125377 | 0.01530269 | Up | 383435 | membrane-spanning 4-domains, subfamily A, member 14 [Source:MGI Symbol;Acc:MGI:2686122] | Ms4a14 | protein_coding |
| ENSMUSG00000099719 | 191.737511 | -1.7817881 | 0.45691567 | 4.00E-06 | 0.00036162 | Down | NA | predicted gene 28802 [Source:MGI Symbol;Acc:MGI:5579508] | Gm28802 | unprocessed_pseu dogene |
| ENSMUSG00000099764 | 25.9476084 | -1.2288462 | 0.38615564 | 6.79E-05 | 0.00225926 | Down | NA | ribosomal protein S10, pseudogene 2 [Source:MGI Symbol;Acc:MGI:3645627] | Rps10-ps2 | processed_pseudo gene |
| ENSMUSG00000100164 | 55.4519891 | -0.6101565 | 0.30388924 | 0.00277394 | 0.02571326 | Down | 67170 | RIKEN cDNA 2610306M01 gene [Source:MGI Symbol;Acc:MGI:1914420] | 2610306M01Rik | lncRNA |
| ENSMUSG00000100774 | 14.3501797 | 1.60440855 | 0.58362193 | 0.00022187 | 0.00501301 | Up | NA | predicted gene 7329 [Source:MGI Symbol;Acc:MGI:3646271] | Gm7329 | processed_pseudo gene |
| ENSMUSG00000100954 | 28.6661498 | -1.3959876 | 0.42986723 | 5.17E-05 | 0.00191161 | Down | NA | predicted gene 10138 [Source:MGI Symbol;Acc:MGI:3708522] | Gm10138 | lncRNA |
| ENSMUSG00000101089 | 803.086977 | -0.7860617 | 0.29629523 | 0.0004328 | 0.00759277 | Down | 100503074 | RIKEN cDNA 2610016A17 gene [Source:MGI Symbol;Acc:MGI:1919653] | 2610016A17Rik | lncRNA |
| ENSMUSG00000101335 | 27.7616167 | 1.23065466 | 0.35976533 | 2.99E-05 | 0.00141298 | Up | NA | predicted gene 28229 [Source:MGI Symbol;Acc:MGI:5578935] | Gm28229 | lncRNA |
| ENSMUSG00000101925 | 172.432655 | -0.4764467 | 0.25474092 | 0.00549921 | 0.0402046 | Down | NA | predicted gene 28156 [Source:MGI Symbol;Acc:MGI:5578862] | Gm28156 | lncRNA |
| ENSMUSG00000102252 | 270.78139 | -0.7416021 | 0.26844743 | 0.00033696 | 0.00659511 | Down | 20646 | small nuclear ribonucleoprotein N [Source:MGI Symbol;Acc:MGI:98347] | Snrpn | protein_coding |
| ENSMUSG00000102457 | 12.6611854 | -0.8712871 | 0.59595918 | 0.00454562 | 0.03538268 | Down | NA | predicted gene, 38342 [Source:MGI Symbol;Acc:MGI:5611570] | Gm38342 | TEC |
| ENSMUSG00000102496 | 19.5034949 | 1.53730035 | 0.41683717 | 1.16E-05 | 0.00075871 | Up | NA | predicted gene, 36989 [Source:MGI Symbol;Acc:MGI:5610217] | Gm36989 | TEC |
| ENSMUSG00000102559 | 22.9276958 | 0.63996334 | 0.44550025 | 0.00644243 | 0.04467328 | Up | NA | predicted gene, 37570 [Source:MGI Symbol;Acc:MGI:5610798] | Gm37570 | TEC |
| ENSMUSG00000102704 | 9.64552974 | 1.39370418 | 0.59858834 | 0.00075125 | 0.01114106 | Up | NA | predicted gene, 38309 [Source:MGI Symbol;Acc:MGI:5611537] | Gm38309 | TEC |
| ENSMUSG00000102752 | 249.411366 | -0.8407102 | 0.34267707 | 0.0007176 | 0.01086315 | Down | 665574 | predicted gene 7694 [Source:MGI Symbol;Acc:MGI:3649135] | Gm7694 | protein_coding |
| ENSMUSG00000102869 | 3149.17016 | 0.1937093 | 0.07705147 | 0.00659196 | 0.04538255 | Up | 347740 | non-coding RNA activated by DNA damage [Source:MGI Symbol;Acc:MGI:1914767] | Norad | lncRNA |
| ENSMUSG00000102976 | 191.786169 | 0.44919746 | 0.23549035 | 0.0057536 | 0.04141219 | Up | 70579 | zinc finger CCCH type containing 11A [Source:MGI Symbol;Acc:MGI:1917829] | Zc3h11a | protein_coding |
| ENSMUSG00000103041 | 149.439074 | 0.70029398 | 0.19880681 | 3.14E-05 | 0.00144359 | Up | NA | predicted gene, 37305 [Source:MGI Symbol;Acc:MGI:5610533] | Gm37305 | lncRNA |
| ENSMUSG00000103149 | 69.6752974 | 2.04095217 | 0.51229612 | 2.82E-06 | 0.00029173 | Up | NA | EST AA914427 [Source:MGI Symbol;Acc:MGI:1858439] | AA914427 | TEC |
| ENSMUSG00000103160 | 68.3157246 | 0.77156277 | 0.31183607 | 0.00072551 | 0.01090276 | Up | NA | RIKEN cDNA C130012C08 gene [Source:MGI Symbol;Acc:MGI:2145684] | C130012C08Rik | TEC |
| ENSMUSG00000103313 | 12.4715183 | -1.5698815 | 0.66663132 | 0.00065089 | 0.01005104 | Down | NA | predicted gene, 38357 [Source:MGI Symbol;Acc:MGI:5611585] | Gm38357 | TEC |
| ENSMUSG00000103411 | 12.3614253 | 1.21866588 | 0.5849501 | 0.00128714 | 0.01560413 | Up | NA | predicted gene, 18300 [Source:MGI Symbol;Acc:MGI:5010485] | Gm18300 | processed_pseudo gene |
| ENSMUSG00000103509 | 14.1924922 | -1.6347738 | 0.58820031 | 0.00022134 | 0.00500793 | Down | NA | predicted gene, 38372 [Source:MGI Symbol;Acc:MGI:5611600] | Gm38372 | TEC |
| ENSMUSG00000103527 | 28.4077107 | 1.15980352 | 0.43872074 | 0.00034396 | 0.00667501 | Up | NA | predicted gene, 37261 [Source:MGI Symbol;Acc:MGI:5610489] | Gm37261 | TEC |
| ENSMUSG00000103821 | 13.5147179 | 0.76271329 | 0.51447697 | 0.00514989 | 0.03841138 | Up | NA | RIKEN cDNA D430013B06 gene [Source:MGI Symbol;Acc:MGI:3026994] | D430013B06Rik | TEC |
| ENSMUSG00000103857 | 21.2098317 | 0.74367566 | 0.49794459 | 0.00496385 | 0.0376812 | Up | NA | predicted gene, 37249 [Source:MGI Symbol;Acc:MGI:5610477] | Gm37249 | TEC |
| ENSMUSG00000103901 | 18.3721271 | 0.69553533 | 0.43773339 | 0.00485689 | 0.0370882 | Up | NA | predicted gene, 37499 [Source:MGI Symbol;Acc:MGI:5610727] | Gm37499 | TEC |
| ENSMUSG00000103983 | 44.0350972 | 0.92350039 | 0.40648322 | 0.00096337 | 0.01311446 | Up | NA | predicted gene, 20045 [Source:MGI Symbol;Acc:MGI:5012230] | Gm20045 | lncRNA |
| ENSMUSG00000104156 | 58.3649385 | 0.64125848 | 0.31665138 | 0.00248837 | 0.02401876 | Up | NA | predicted gene, 38102 [Source:MGI Symbol;Acc:MGI:5611330] | Gm38102 | lncRNA |
| ENSMUSG00000104184 | 12.8067139 | 1.34294156 | 0.5914573 | 0.00084271 | 0.01195619 | Up | NA | predicted gene, 37818 [Source:MGI Symbol;Acc:MGI:5611046] | Gm37818 | TEC |
| ENSMUSG00000104399 | 57.3373723 | 1.01193789 | 0.28065671 | 1.69E-05 | 0.00096384 | Up | NA | predicted gene, 37963 [Source:MGI Symbol;Acc:MGI:5611191] | Gm37963 | lncRNA |
| ENSMUSG00000104548 | 34.2321816 | 0.95429647 | 0.32802253 | 0.00018082 | 0.00441012 | Up | NA | predicted gene 43857 [Source:MGI Symbol;Acc:MGI:5663994] | Gm43857 | TEC |
| ENSMUSG00000104569 | 8.82860242 | 0.8373075 | 0.72177473 | 0.00651388 | 0.04497148 | Up | NA | predicted gene 43054 [Source:MGI Symbol;Acc:MGI:5663191] | Gm43054 | TEC |

| | | | | | | | | | | |
|---|---|---|---|---|---|---|---|---|---|---|
| ENSMUSG00000104814 | 64.4088396 | 0.83233712 | 0.29635065 | 0.00027298 | 0.00571464 | Up | NA | predicted gene 42979 [Source:MGI Symbol;Acc:MGI:5663116] | Gm42979 | TEC |
| ENSMUSG00000104950 | 40.0676295 | 0.9882349 | 0.29447786 | 4.22E-05 | 0.00168414 | Up | NA | RIKEN cDNA 4833413G10 gene [Source:MGI Symbol;Acc:MGI:1921834] | 4833413G10Rik | lncRNA |
| ENSMUSG00000105677 | 39.2962849 | 0.70317621 | 0.34621705 | 0.00223244 | 0.0222901 | Up | NA | predicted gene 43328 [Source:MGI Symbol;Acc:MGI:5663465] | Gm43328 | lncRNA |
| ENSMUSG00000106087 | 21.1716355 | 1.04244735 | 0.39054294 | 0.00034201 | 0.00664584 | Up | NA | predicted gene 43609 [Source:MGI Symbol;Acc:MGI:5663746] | Gm43609 | TEC |
| ENSMUSG00000106251 | 13.9383929 | 1.3549147 | 0.74052079 | 0.00191701 | 0.02020881 | Up | NA | predicted gene 42658 [Source:MGI Symbol;Acc:MGI:5662795] | Gm42658 | lncRNA |
| ENSMUSG00000106535 | 17.6572194 | 1.27702419 | 0.49552808 | 0.00038767 | 0.00714987 | Up | NA | predicted gene 43031 [Source:MGI Symbol;Acc:MGI:5663168] | Gm43031 | lncRNA |
| ENSMUSG00000106743 | 20.9117073 | 1.07453688 | 0.56214198 | 0.00185665 | 0.019752 | Up | NA | predicted gene 42847 [Source:MGI Symbol;Acc:MGI:5662984] | Gm42847 | TEC |
| ENSMUSG00000106864 | 466.569851 | 0.32986918 | 0.09119513 | 7.55E-05 | 0.00242819 | Up | 71752 | general transcription factor IIIC, polypeptide 2, beta [Source:MGI Symbol;Acc:MGI:1919002] | Gtf3c2 | protein_coding |
| ENSMUSG00000106870 | 37.6776025 | 0.89839436 | 0.33584711 | 0.00037677 | 0.00705139 | Up | NA | predicted gene 43681 [Source:MGI Symbol;Acc:MGI:5663818] | Gm43681 | TEC |
| ENSMUSG00000106951 | 81.6227081 | 0.56831925 | 0.28488844 | 0.00319315 | 0.02819979 | Up | 319982 | RIKEN cDNA 5930430L01 gene [Source:MGI Symbol;Acc:MGI:2443110] | 5930430L01Rik | lncRNA |
| ENSMUSG00000107000 | 68.6867711 | 0.63111574 | 0.36931213 | 0.00450877 | 0.03515976 | Up | NA | predicted gene 43481 [Source:MGI Symbol;Acc:MGI:5663618] | Gm43481 | TEC |
| ENSMUSG00000107002 | 699.516249 | -0.585043 | 0.14600261 | 5.95E-06 | 0.00046465 | Down | 106264 | RIKEN cDNA 0610012G03 gene [Source:MGI Symbol;Acc:MGI:1913301] | 0610012G03Rik | protein_coding |
| ENSMUSG00000107096 | 66.4898681 | 0.78107668 | 0.23205559 | 5.01E-05 | 0.00187013 | Up | NA | predicted gene 43597 [Source:MGI Symbol;Acc:MGI:5663734] | Gm43597 | lncRNA |
| ENSMUSG00000107143 | 22.9255715 | -0.6246881 | 0.46879361 | 0.00755743 | 0.04988808 | Down | NA | predicted gene 6598 [Source:MGI Symbol;Acc:MGI:3646314] | Gm6598 | lncRNA |
| ENSMUSG00000107216 | 11.0035206 | 1.95788376 | 0.57008751 | 2.99E-05 | 0.00141298 | Up | NA | RIKEN cDNA 2210412B16 gene [Source:MGI Symbol;Acc:MGI:1919625] | 2210412B16Rik | TEC |
| ENSMUSG00000107655 | 146.288944 | 0.6641064 | 0.36390483 | 0.00342809 | 0.02941857 | Up | NA | predicted gene, 44220 [Source:MGI Symbol;Acc:MGI:5690612] | Gm44220 | TEC |
| ENSMUSG00000107741 | 119.236001 | 0.61938858 | 0.1890934 | 8.84E-05 | 0.00268763 | Up | NA | predicted gene 2011 [Source:MGI Symbol;Acc:MGI:3780180] | Gm2011 | transcribed_unprocessed_pseudogene |
| ENSMUSG00000107962 | 121.132705 | 0.64711073 | 0.35597146 | 0.00358864 | 0.03034741 | Up | NA | predicted gene, 43980 [Source:MGI Symbol;Acc:MGI:5690372] | Gm43980 | TEC |
| ENSMUSG00000108231 | 10.3457428 | -1.3883226 | 0.50729992 | 0.00026645 | 0.00565819 | Down | NA | predicted gene 4045 [Source:MGI Symbol;Acc:MGI:3782220] | Gm4045 | processed_pseudogene |
| ENSMUSG00000108298 | 15.4022262 | 0.78310006 | 0.5638115 | 0.00538525 | 0.03962108 | Up | NA | predicted gene, 44226 [Source:MGI Symbol;Acc:MGI:5690618] | Gm44226 | lncRNA |
| ENSMUSG00000108425 | 65.5092611 | 0.86394356 | 0.38464063 | 0.00111371 | 0.01424975 | Up | NA | predicted gene 44706 [Source:MGI Symbol;Acc:MGI:5753282] | Gm44706 | lncRNA |
| ENSMUSG00000108461 | 14.6424687 | -1.1601288 | 0.58123557 | 0.00156455 | 0.01769928 | Down | NA | expressed sequence AV356131 [Source:MGI Symbol;Acc:MGI:2142184] | AV356131 | lncRNA |
| ENSMUSG00000108563 | 26.4686851 | 0.77726496 | 0.36677203 | 0.0016512 | 0.01830969 | Up | NA | predicted gene 44686 [Source:MGI Symbol;Acc:MGI:5753262] | Gm44686 | lncRNA |
| ENSMUSG00000108614 | 9.09145503 | 0.16285981 | 0.30490395 | 0.0054989 | 0.0402046 | Up | NA | RIKEN cDNA 2610306O10 gene [Source:MGI Symbol;Acc:MGI:1917710] | 2610306O10Rik | TEC |
| ENSMUSG00000108621 | 696.216392 | 0.39071506 | 0.12326802 | 0.00027082 | 0.00570043 | Up | 102637087 | predicted gene, 33989 [Source:MGI Symbol;Acc:MGI:5593148] | Gm33989 | lncRNA |
| ENSMUSG00000108681 | 11.4058576 | 1.31163319 | 0.57474246 | 0.00094063 | 0.01292775 | Up | NA | predicted gene, 17825 [Source:MGI Symbol;Acc:MGI:5010010] | Gm17825 | processed_pseudogene |
| ENSMUSG00000109118 | 23.3086213 | -0.778004 | 0.4474521 | 0.00326479 | 0.02860654 | Down | NA | predicted gene, 32031 [Source:MGI Symbol;Acc:MGI:5591190] | Gm32031 | lncRNA |
| ENSMUSG00000109284 | 14.4838997 | -0.9035156 | 0.55576666 | 0.00329241 | 0.02878642 | Down | 381914 | RIKEN cDNA B230311B06 gene [Source:MGI Symbol;Acc:MGI:2686541] | B230311B06Rik | lncRNA |
| ENSMUSG00000109378 | 29.939985 | 0.78787699 | 0.34867076 | 0.00120687 | 0.01501193 | Up | NA | predicted gene, 49396 [Source:MGI Symbol;Acc:MGI:6121629] | Gm49396 | protein_coding |
| ENSMUSG00000109498 | 12.3032956 | 1.4891095 | 0.48584288 | 9.99E-05 | 0.00291573 | Up | NA | predicted gene 45222 [Source:MGI Symbol;Acc:MGI:5753798] | Gm45222 | TEC |
| ENSMUSG00000109559 | 46.1239153 | 0.82216645 | 0.30980424 | 0.00042763 | 0.00754266 | Up | NA | predicted gene, 34280 [Source:MGI Symbol;Acc:MGI:5593439] | Gm34280 | lncRNA |
| ENSMUSG00000109572 | 26.777216 | -1.1680935 | 0.47789587 | 0.00054967 | 0.00892046 | Down | 100862066 | cilia and flagella associated protein 99 [Source:MGI Symbol;Acc:MGI:5434801] | Cfap99 | protein_coding |
| ENSMUSG00000109603 | 64.3498146 | -0.6444945 | 0.27317902 | 0.00117853 | 0.01478398 | Down | 102634919 | predicted gene, 32389 [Source:MGI Symbol;Acc:MGI:5591548] | Gm32389 | lncRNA |
| ENSMUSG00000109998 | 16.3245696 | 1.40927704 | 0.65654594 | 0.00103327 | 0.01355327 | Up | NA | predicted gene 45437 [Source:MGI Symbol;Acc:MGI:5791273] | Gm45437 | lncRNA |
| ENSMUSG00000110159 | 17.1038948 | 1.07687413 | 0.50248411 | 0.00124989 | 0.01528956 | Up | NA | predicted gene 45501 [Source:MGI Symbol;Acc:MGI:5791337] | Gm45501 | TEC |
| ENSMUSG00000110170 | 29.7654035 | 1.39836484 | 0.75358136 | 0.00169369 | 0.01861564 | Up | NA | ST6 (alpha-N-acetyl-neuraminyl-2,3-beta-galactosyl-1,3)-N-acetylgalactosaminide alpha-2,6-sialyltransferase 2 [Source:MGI Symbol;Acc:MGI:107553] | St6galnac2 | protein_coding |
| ENSMUSG00000110357 | 44.0121667 | 0.59865978 | 0.42640027 | 0.0073269 | 0.04890116 | Up | NA | RIKEN cDNA A030001D20 gene [Source:MGI Symbol;Acc:MGI:3696869] | A030001D20Rik | lncRNA |
| ENSMUSG00000110496 | 34.9989772 | 1.43323727 | 0.73481987 | 0.00138675 | 0.01649175 | Up | NA | predicted gene 45909 [Source:MGI Symbol;Acc:MGI:5805024] | Gm45909 | TEC |
| ENSMUSG00000110580 | 46.9826775 | 0.67161372 | 0.4060644 | 0.00447547 | 0.03497063 | Up | NA | RIKEN cDNA D830024N08 gene [Source:MGI Symbol;Acc:MGI:2442966] | D830024N08Rik | lncRNA |
| ENSMUSG00000110588 | 10.6506145 | -1.8847055 | 0.73647341 | 0.00048956 | 0.0082584 | Down | NA | predicted gene 45774 [Source:MGI Symbol;Acc:MGI:5804889] | Gm45774 | lncRNA |
| ENSMUSG00000110884 | 50.5768856 | -0.8977842 | 0.43400535 | 0.00153776 | 0.01748136 | Down | NA | predicted gene, 47416 [Source:MGI Symbol;Acc:MGI:6096354] | Gm47416 | lncRNA |
| ENSMUSG00000111057 | 8.73249778 | 0.88112909 | 0.69652314 | 0.00555432 | 0.04051636 | Up | NA | predicted gene 3442 [Source:MGI Symbol;Acc:MGI:3781619] | Gm3442 | processed_pseudogene |
| ENSMUSG00000111658 | 74.8108734 | -1.1368874 | 0.39063597 | 0.00015489 | 0.00398141 | Down | 622037 | RIKEN cDNA B930092H01 gene [Source:MGI Symbol;Acc:MGI:4437738] | B930092H01Rik | lncRNA |
| ENSMUSG00000111681 | 30.5253665 | 0.90935528 | 0.4147986 | 0.00121374 | 0.01505136 | Up | NA | predicted gene, 47640 [Source:MGI Symbol;Acc:MGI:6096715] | Gm47640 | TEC |
| ENSMUSG00000111774 | 29.9811853 | -0.863703 | 0.43321307 | 0.00185146 | 0.01974549 | Down | NA | predicted gene, 38398 [Source:MGI Symbol;Acc:MGI:5618692] | Gm38398 | lncRNA |
| ENSMUSG00000111840 | 84.746239 | 0.85905415 | 0.57134142 | 0.00405865 | 0.03286198 | Up | NA | predicted gene, 48832 [Source:MGI Symbol;Acc:MGI:6098557] | Gm48832 | lncRNA |
| ENSMUSG00000111942 | 100.304001 | 0.85778205 | 0.47424562 | 0.00259087 | 0.02457199 | Up | NA | predicted gene 5182 [Source:MGI Symbol;Acc:MGI:3649174] | Gm5182 | processed_pseudogene |
| ENSMUSG00000112278 | 54.6345655 | -1.0665051 | 0.42054349 | 0.0004525 | 0.00781178 | Down | NA | predicted gene, 30025 [Source:MGI Symbol;Acc:MGI:5589184] | Gm30025 | lncRNA |
| ENSMUSG00000112327 | 44.3991574 | -2.0190679 | 0.76989053 | 0.00027252 | 0.00571231 | Down | NA | predicted gene, 36827 [Source:MGI Symbol;Acc:MGI:5595986] | Gm36827 | lncRNA |
| ENSMUSG00000112352 | 92.5685989 | 0.49878773 | 0.24496186 | 0.00368759 | 0.03089471 | Up | 105245123 | predicted gene, 40617 [Source:MGI Symbol;Acc:MGI:5623502] | Gm40617 | lncRNA |
| ENSMUSG00000112547 | 16.9563707 | 1.52130914 | 0.39525926 | 5.88E-06 | 0.00046162 | Up | NA | predicted gene, 47096 [Source:MGI Symbol;Acc:MGI:6095829] | Gm47096 | lncRNA |
| ENSMUSG00000113165 | 91.271179 | -0.6273609 | 0.18280286 | 5.06E-05 | 0.0018818 | Down | NA | predicted gene, 47863 [Source:MGI Symbol;Acc:MGI:6097080] | Gm47863 | lncRNA |
| ENSMUSG00000113570 | 10.0212464 | -0.9034349 | 0.63457893 | 0.00457016 | 0.03555667 | Down | NA | predicted gene 5953 [Source:MGI Symbol;Acc:MGI:3647171] | Gm5953 | TEC |
| ENSMUSG00000113581 | 23.9232251 | 0.76249001 | 0.73150356 | 0.00710426 | 0.0479133 | Up | 353504 | deiodinase, iodothyronine type III, opposite strand [Source:MGI Symbol;Acc:MGI:2664395] | Dio3os | lncRNA |
| ENSMUSG00000113898 | 29.2262104 | 0.72728758 | 0.53463173 | 0.00588261 | 0.04191362 | Up | NA | predicted gene, 19144 [Source:MGI Symbol;Acc:MGI:5011329] | Gm19144 | processed_pseudogene |
| ENSMUSG00000114253 | 96.8469998 | 0.80430203 | 0.3383719 | 0.00087105 | 0.01228317 | Up | NA | predicted gene, 47798 [Source:MGI Symbol;Acc:MGI:6096974] | Gm47798 | lncRNA |
| ENSMUSG00000114298 | 32.368387 | -1.2793765 | 0.5597552 | 0.00073405 | 0.01099046 | Down | NA | predicted gene, 48371 [Source:MGI Symbol;Acc:MGI:6097844] | Gm48371 | lncRNA |
| ENSMUSG00000114796 | 86.3027583 | 1.00353124 | 0.41379935 | 0.00063834 | 0.0099043 | Up | NA | RIKEN cDNA A930028N01 gene [Source:MGI Symbol;Acc:MGI:1925688] | A930028N01Rik | lncRNA |
| ENSMUSG00000115219 | 39.7171067 | -0.8629049 | 0.31400916 | 0.00031837 | 0.00636162 | Down | 110599566 | EEF1A lysine methyltransferase 4 [Source:MGI Symbol;Acc:MGI:5903914] | Eef1akmt4 | protein_coding |
| ENSMUSG00000115441 | 20.0447595 | 1.03075041 | 0.31560557 | 5.87E-05 | 0.00204593 | Up | NA | predicted gene, 34759 [Source:MGI Symbol;Acc:MGI:5593918] | Gm34759 | lncRNA |
| ENSMUSG00000115553 | 8.39928905 | -1.3715047 | 0.80599577 | 0.00247381 | 0.02389243 | Down | NA | predicted gene, 49124 [Source:MGI Symbol;Acc:MGI:6118528] | Gm49124 | lncRNA |
| ENSMUSG00000115624 | 52.0227426 | 0.5208661 | 0.27444347 | 0.00446689 | 0.03492039 | Up | NA | predicted gene, 49204 [Source:MGI Symbol;Acc:MGI:6118653] | Gm49204 | TEC |
| ENSMUSG00000116041 | 16.2811146 | 1.30549346 | 0.93361768 | 0.00341913 | 0.02938078 | Up | NA | predicted gene, 20369 [Source:MGI Symbol;Acc:MGI:5012554] | Gm20369 | processed_pseudogene |
| ENSMUSG00000116564 | 395.431259 | 0.32774401 | 0.12293132 | 0.00181057 | 0.01945285 | Up | 67045 | RIO kinase 2 [Source:MGI Symbol;Acc:MGI:1914295] | Riok2 | protein_coding |
| ENSMUSG00000116718 | 50.9767382 | 0.73439358 | 0.35149539 | 0.00186292 | 0.0197925 | Up | NA | predicted gene, 49668 [Source:MGI Symbol;Acc:MGI:6215110] | Gm49668 | lncRNA |
| ENSMUSG00000116720 | 43.1229929 | 0.52610927 | 0.28807952 | 0.00502776 | 0.03793586 | Up | NA | predicted gene, 31641 [Source:MGI Symbol;Acc:MGI:5590800] | Gm31641 | lncRNA |
| ENSMUSG00000116858 | 81.2006928 | 0.5986765 | 0.22090453 | 0.00053028 | 0.00877232 | Up | NA | predicted gene, 49797 [Source:MGI Symbol;Acc:MGI:6215329] | Gm49797 | lncRNA |
| ENSMUSG00000116903 | 134.942992 | 0.6423166 | 0.28502982 | 0.00153033 | 0.01744575 | Up | 100503040 | predicted gene, 19522 [Source:MGI Symbol;Acc:MGI:5011707] | Gm19522 | lncRNA |
| ENSMUSG00000116908 | 37.8609493 | -1.6975502 | 0.54660372 | 7.23E-05 | 0.00235696 | Down | NA | predicted gene, 49599 [Source:MGI Symbol;Acc:MGI:6215007] | Gm49599 | TEC |
| ENSMUSG00000116946 | 61.8940755 | 0.5193857 | 0.30987121 | 0.00647552 | 0.0447803 | Up | NA | predicted gene, 41442 [Source:MGI Symbol;Acc:MGI:5624327] | Gm41442 | lncRNA |
| ENSMUSG00000117113 | 50.9933764 | 0.92304767 | 0.27759251 | 4.99E-05 | 0.00187013 | Up | NA | predicted gene, 49883 [Source:MGI Symbol;Acc:MGI:6270565] | Gm49883 | TEC |
