# Supplementary material for: The effect of aldafermin expressing-Escherichia coli Nissle 1917 along with dietary change on visceral adipose tissue in MASLD mouse model
Source: Int J Obes (Lond). 2025 Apr 10;49(7):1334–44. doi: 10.1038/s41366-025-01774-w (PMC12283412; doi:10.1038/s41366-025-01774-w)
Supplement: Supplementary file 4 — Supplementary table 5 [file 41366_2025_1774_MOESM4_ESM.pdf]

# Supplementary table 5. DEGs observed in eVAT when EcN was compared to CTRL

**EcN vs CTRL eVAT**

| ENSEMBL | baseMean | log2FoldChange | lfcSE | pvalue | padj | UD | entrezgene_id | description | external_gene_name | gene_biotype |
|---|---|---|---|---|---|---|---|---|---|---|
| ENSMUSG00000000093 | 140.186431 | 1.70408537 | 0.71550337 | 0.0004514 | 0.02990199 | Up | 21385 | T-box 2 [Source:MGI Symbol;Acc:MGI:98494] | Tbx2 | protein_coding |
| ENSMUSG00000000157 | 5.85424264 | 0.0040573 | 0.0624855 | 0.00077704 | 0.04063604 | Up | 16415 | integrin beta 2-like [Source:MGI Symbol;Acc:MGI:1277979] | Itgb2l | protein_coding |
| ENSMUSG00000000303 | 47.3274107 | 4.27003475 | 1.96371238 | 0.00042101 | 0.02870254 | Up | 12550 | cadherin 1 [Source:MGI Symbol;Acc:MGI:88354] | Cdh1 | protein_coding |
| ENSMUSG00000000320 | 52.5806853 | 2.46426441 | 0.74627518 | 3.10E-05 | 0.00653011 | Up | 11684 | arachidonate 12-lipoxygenase [Source:MGI Symbol;Acc:MGI:87998] | Alox12 | protein_coding |
| ENSMUSG00000000385 | 20.0453899 | 4.52500934 | 1.94245706 | 0.00033471 | 0.02487066 | Up | 50528 | transmembrane protease, serine 2 [Source:MGI Symbol;Acc:MGI:1354381] | Tmprss2 | protein_coding |
| ENSMUSG00000000567 | 116.191236 | 1.62204127 | 0.60560811 | 0.0002208 | 0.019409 | Up | 20682 | SRY (sex determining region Y)-box 9 [Source:MGI Symbol;Acc:MGI:98371] | Sox9 | protein_coding |
| ENSMUSG00000001225 | 36.5427473 | 4.88929673 | 1.84008085 | 0.00013454 | 0.01447491 | Up | 13487 | solute carrier family 26, member 3 [Source:MGI Symbol;Acc:MGI:107181] | Slc26a3 | protein_coding |
| ENSMUSG00000001504 | 17.6079953 | 3.36344022 | 1.28679142 | 0.00021524 | 0.01916466 | Up | 16372 | Iroquois homeobox 2 [Source:MGI Symbol;Acc:MGI:1197526] | Irx2 | protein_coding |
| ENSMUSG00000001670 | 47.1501716 | -0.0162813 | 0.06536706 | 0.00114966 | 0.04968317 | Down | 234724 | tyrosine aminotransferase [Source:MGI Symbol;Acc:MGI:98487] | Tat | protein_coding |
| ENSMUSG00000001763 | 61.350056 | 1.26459876 | 0.53481487 | 0.00049686 | 0.03187494 | Up | 232670 | tetraspanin 33 [Source:MGI Symbol;Acc:MGI:1919012] | Tspan33 | protein_coding |
| ENSMUSG00000002032 | 23.7985085 | 1.84655366 | 0.53316955 | 1.96E-05 | 0.0049459 | Up | 71687 | transmembrane protein 25 [Source:MGI Symbol;Acc:MGI:1918937] | Tmem25 | protein_coding |
| ENSMUSG00000002289 | 11762.857 | -0.4179257 | 0.17202574 | 0.00053488 | 0.0331802 | Down | 57875 | angiopoietin-like 4 [Source:MGI Symbol;Acc:MGI:1888999] | Angptl4 | protein_coding |
| ENSMUSG00000002324 | 36.7663416 | 3.98361163 | 1.36286682 | 8.23E-05 | 0.01200954 | Up | 56739 | REC8 meiotic recombination protein [Source:MGI Symbol;Acc:MGI:1929645] | Rec8 | protein_coding |
| ENSMUSG00000003309 | 12.6190398 | 0.00320953 | 0.06241331 | 0.00093797 | 0.04454146 | Up | 11768 | adaptor protein complex AP-1, mu 2 subunit [Source:MGI Symbol;Acc:MGI:1336974] | Ap1m2 | protein_coding |
| ENSMUSG00000003354 | 10.3473629 | 2.62818902 | 1.26468533 | 0.00080897 | 0.04153085 | Up | 105833 | coiled-coil domain containing 65 [Source:MGI Symbol;Acc:MGI:2146001] | Ccdc65 | protein_coding |
| ENSMUSG00000004035 | 249.138685 | 1.87125895 | 0.67126531 | 0.0001579 | 0.01560614 | Up | 68312 | glutathione S-transferase, mu 7 [Source:MGI Symbol;Acc:MGI:1915562] | Gstm7 | protein_coding |
| ENSMUSG00000004631 | 1973.87021 | -0.2609384 | 0.11514357 | 0.00113198 | 0.04922381 | Down | 20392 | sarcoglycan, epsilon [Source:MGI Symbol;Acc:MGI:1329042] | Sgce | protein_coding |
| ENSMUSG00000004864 | 18.1230217 | 2.03299792 | 0.72521564 | 0.00014862 | 0.01519856 | Up | 26415 | mitogen-activated protein kinase 13 [Source:MGI Symbol;Acc:MGI:1346864] | Mapk13 | protein_coding |
| ENSMUSG00000005220 | 13.2641767 | 2.77057286 | 1.07975749 | 0.00027691 | 0.02243217 | Up | 53419 | corin, serine peptidase [Source:MGI Symbol;Acc:MGI:1349451] | Corin | protein_coding |
| ENSMUSG00000005237 | 36.7782662 | 2.71324107 | 0.77581068 | 1.58E-05 | 0.00434587 | Up | 327954 | dynein, axonemal, heavy chain 2 [Source:MGI Symbol;Acc:MGI:107731] | Dnah2 | protein_coding |
| ENSMUSG00000005533 | 512.817427 | 0.64413033 | 0.27949758 | 0.0006363 | 0.03674817 | Up | 16001 | insulin-like growth factor I receptor [Source:MGI Symbol;Acc:MGI:96433] | Igf1r | protein_coding |
| ENSMUSG00000005681 | 86.6761438 | -0.0098611 | 0.06343676 | 0.00017371 | 0.01623723 | Down | 11807 | apolipoprotein A-II [Source:MGI Symbol;Acc:MGI:88050] | Apoa2 | protein_coding |
| ENSMUSG00000005873 | 9254.82744 | -0.3674623 | 0.15742646 | 0.00075634 | 0.04034378 | Down | 13476 | receptor accessory protein 5 [Source:MGI Symbol;Acc:MGI:1270152] | Reep5 | protein_coding |
| ENSMUSG00000006345 | 85.7619201 | 6.49238181 | 2.51280535 | 0.0001015 | 0.01292602 | Up | 14598 | gamma-glutamyltransferase 1 [Source:MGI Symbol;Acc:MGI:95706] | Ggt1 | protein_coding |
| ENSMUSG00000006538 | 40.1207253 | 5.71765793 | 1.70100293 | 1.77E-05 | 0.00474521 | Up | 16147 | Indian hedgehog [Source:MGI Symbol;Acc:MGI:96533] | Ihh | protein_coding |
| ENSMUSG00000006784 | 40.2615344 | 1.71564072 | 0.70360817 | 0.0003983 | 0.02779628 | Up | 74407 | outer dynein arm complex subunit 4 [Source:MGI Symbol;Acc:MGI:1921657] | Odad4 | protein_coding |
| ENSMUSG00000007034 | 31.8243273 | 3.88289903 | 1.64346368 | 0.00033592 | 0.02487106 | Up | 70129 | solute carrier family 44, member 4 [Source:MGI Symbol;Acc:MGI:1917379] | Slc44a4 | protein_coding |
| ENSMUSG00000007035 | 21.0377717 | 1.71040258 | 0.41945836 | 1.86E-06 | 0.00153749 | Up | 17687 | mutS homolog 5 [Source:MGI Symbol;Acc:MGI:1329021] | Msh5 | protein_coding |
| ENSMUSG00000008090 | 5840.59654 | -0.3124341 | 0.13803558 | 0.00112547 | 0.0490482 | Down | 116701 | fibroblast growth factor receptor-like 1 [Source:MGI Symbol;Acc:MGI:2150920] | Fgfrl1 | protein_coding |
| ENSMUSG00000008601 | 8.90841635 | 4.27118058 | 1.61815872 | 0.00023357 | 0.02018739 | Up | 53868 | RAB25, member RAS oncogene family [Source:MGI Symbol;Acc:MGI:1858203] | Rab25 | protein_coding |
| ENSMUSG00000009114 | 8.11631478 | 0.00437811 | 0.06251958 | 0.00011448 | 0.01374931 | Up | 76964 | RIKEN cDNA 2610028H24 gene [Source:MGI Symbol;Acc:MGI:1924214] | 2610028H24Rik | protein_coding |
| ENSMUSG00000009210 | 13.3468171 | 3.50558028 | 1.48495878 | 0.00040141 | 0.02789861 | Up | 75573 | proline rich 29 [Source:MGI Symbol;Acc:MGI:1922823] | Prr29 | protein_coding |
| ENSMUSG00000009545 | 58.782609 | 0.85574042 | 0.28716654 | 0.00010465 | 0.01292602 | Up | 16535 | potassium voltage-gated channel, subfamily Q, member 1 [Source:MGI Symbol;Acc:MGI:108083] | Kcnq1 | protein_coding |
| ENSMUSG00000010044 | 9.09587151 | 3.90050003 | 1.71836679 | 0.00050924 | 0.03236705 | Up | 114602 | zinc finger, MYND domain containing 10 [Source:MGI Symbol;Acc:MGI:2387863] | Zmynd10 | protein_coding |
| ENSMUSG00000010492 | 15.8960137 | 2.22723281 | 1.02662484 | 0.0007061 | 0.03868938 | Up | 100271841 | uridine-cytidine kinase 1-like 1, opposite strand [Source:MGI Symbol;Acc:MGI:3801877] | Uckl1os | lncRNA |
| ENSMUSG00000012126 | 48.5314651 | 2.13335746 | 0.60132388 | 1.37E-05 | 0.00408772 | Up | 67586 | UBX domain protein 11 [Source:MGI Symbol;Acc:MGI:1914836] | Ubxn11 | protein_coding |
| ENSMUSG00000012350 | 39.2133627 | 5.31498623 | 1.7859994 | 5.74E-05 | 0.00963581 | Up | 13661 | ets homologous factor [Source:MGI Symbol;Acc:MGI:1270840] | Ehf | protein_coding |
| ENSMUSG00000013418 | 20.7804485 | 2.7434655 | 1.49033493 | 0.00109968 | 0.04841886 | Up | 14422 | beta-1,4-N-acetyl-galactosaminyl transferase 2 [Source:MGI Symbol;Acc:MGI:1342058] | B4galnt2 | protein_coding |
| ENSMUSG00000014329 | 985.437949 | 0.5422935 | 0.22071159 | 0.00049239 | 0.03179384 | Up | 83675 | BicC family RNA binding protein 1 [Source:MGI Symbol;Acc:MGI:1933388] | Bicc1 | protein_coding |
| ENSMUSG00000015090 | 90.9258447 | 4.0363204 | 1.01082979 | 2.04E-06 | 0.00153749 | Up | 19215 | prostaglandin D2 synthase (brain) [Source:MGI Symbol;Acc:MGI:99261] | Ptgds | protein_coding |
| ENSMUSG00000016028 | 117.707569 | 2.77223696 | 0.97756399 | 0.00012407 | 0.01417611 | Up | 12614 | cadherin, EGF LAG seven-pass G-type receptor 1 [Source:MGI Symbol;Acc:MGI:1100883] | Celsr1 | protein_coding |
| ENSMUSG00000016552 | 205.024671 | 1.00873139 | 0.29924894 | 2.70E-05 | 0.00593543 | Up | 239554 | FAD-dependent oxidoreductase domain containing 2 [Source:MGI Symbol;Acc:MGI:106315] | Foxred2 | protein_coding |
| ENSMUSG00000016763 | 19.8626965 | 3.64281945 | 1.08422859 | 2.43E-05 | 0.00551004 | Up | 64706 | signal peptide, CUB domain, EGF-like 1 [Source:MGI Symbol;Acc:MGI:1890616] | Scube1 | protein_coding |
| ENSMUSG00000017057 | 274.85605 | 0.45556718 | 0.18560945 | 0.00048866 | 0.03179384 | Up | 16164 | interleukin 13 receptor, alpha 1 [Source:MGI Symbol;Acc:MGI:105052] | Il13ra1 | protein_coding |
| ENSMUSG00000017344 | 295.670051 | -0.5625116 | 0.25027228 | 0.00071685 | 0.03886597 | Down | 22370 | vitronectin [Source:MGI Symbol;Acc:MGI:98940] | Vtn | protein_coding |
| ENSMUSG00000017639 | 141.498203 | 1.31669732 | 0.58426899 | 0.00063348 | 0.03674817 | Up | 268451 | RAB11 family interacting protein 4 (class II) [Source:MGI Symbol;Acc:MGI:2442920] | Rab11fip4 | protein_coding |
| ENSMUSG00000017723 | 10.6032253 | 2.83390448 | 1.25908327 | 0.00056613 | 0.0343958 | Up | 67701 | WAP four-disulfide core domain 2 [Source:MGI Symbol;Acc:MGI:1914951] | Wfdc2 | protein_coding |
| ENSMUSG00000017832 | 6.53143288 | 2.72819617 | 1.28776562 | 0.00083287 | 0.04196648 | Up | 75482 | heat shock protein, alpha-crystallin-related, B9 [Source:MGI Symbol;Acc:MGI:1922732] | Hspb9 | protein_coding |
| ENSMUSG00000018166 | 112.811918 | 1.38879965 | 0.46084014 | 8.63E-05 | 0.0120438 | Up | 13867 | erb-b2 receptor tyrosine kinase 3 [Source:MGI Symbol;Acc:MGI:95411] | Erbb3 | protein_coding |
| ENSMUSG00000018340 | 12410.3395 | -0.274343 | 0.11450327 | 0.00077041 | 0.04049479 | Down | 11749 | annexin A6 [Source:MGI Symbol;Acc:MGI:88255] | Anxa6 | protein_coding |
| ENSMUSG00000018569 | 23.6727269 | 0.0028848 | 0.06239062 | 0.00013119 | 0.01441539 | Up | 53624 | claudin 7 [Source:MGI Symbol;Acc:MGI:1859285] | Cldn7 | protein_coding |
| ENSMUSG00000018581 | 41.0148387 | 0.00157325 | 0.06232068 | 0.00091447 | 0.04407147 | Up | 13411 | dynein, axonemal, heavy chain 11 [Source:MGI Symbol;Acc:MGI:1100864] | Dnah11 | protein_coding |
| ENSMUSG00000018648 | 36.9093725 | 1.41772704 | 0.45849262 | 6.79E-05 | 0.01046158 | Up | 56405 | dual specificity phosphatase 14 [Source:MGI Symbol;Acc:MGI:1927168] | Dusp14 | protein_coding |
| ENSMUSG00000019027 | 42.2218967 | 1.9743666 | 0.62637539 | 5.25E-05 | 0.00931096 | Up | 110084 | dynein, axonemal, heavy chain 1 [Source:MGI Symbol;Acc:MGI:107721] | Dnah1 | protein_coding |

| | | | | | | | | | | |
|---|---|---|---|---|---|---|---|---|---|---|
| ENSMUSG00000019359 | 4.07188001 | 4.86561213 | 1.54610547 | 1.40E-05 | 0.00411962 | Up | 71584 | glycerophosphodiester phosphodiesterase domain containing 2 [Source:MGI Symbol;Acc:MGI:1918834] | Gdpd2 | protein_coding |
| ENSMUSG00000019590 | 295.606703 | 0.84462351 | 0.27914588 | 8.43E-05 | 0.0120438 | Up | 13056 | cytochrome b-561 [Source:MGI Symbol;Acc:MGI:103253] | Cyb561 | protein_coding |
| ENSMUSG00000019734 | 58.5693763 | 1.5170591 | 0.6413642 | 0.00047807 | 0.03145046 | Up | 353499 | transmembrane channel-like gene family 4 [Source:MGI Symbol;Acc:MGI:2669035] | Tmc4 | protein_coding |
| ENSMUSG00000019767 | 44.7889696 | 1.48344277 | 0.70703507 | 0.00085848 | 0.0426477 | Up | 100504234 | coiled-coil domain containing 170 [Source:MGI Symbol;Acc:MGI:2685067] | Ccdc170 | protein_coding |
| ENSMUSG00000019779 | 75.0049183 | 1.20829566 | 0.33210864 | 1.04E-05 | 0.0035471 | Up | 14302 | fyn-related kinase [Source:MGI Symbol;Acc:MGI:103265] | Frk | protein_coding |
| ENSMUSG00000019982 | 24.4240442 | 3.01585906 | 0.74161096 | 1.82E-06 | 0.00153749 | Up | 17863 | myeloblastosis oncogene [Source:MGI Symbol;Acc:MGI:97249] | Myb | protein_coding |
| ENSMUSG00000020014 | 42.1548658 | 4.05978131 | 1.0642262 | 4.24E-06 | 0.00208608 | Up | 380654 | cilia and flagella associated protein 54 [Source:MGI Symbol;Acc:MGI:1922208] | Cfap54 | protein_coding |
| ENSMUSG00000020051 | 22.9128159 | -0.0090686 | 0.06326653 | 5.19E-05 | 0.00931096 | Down | 18478 | phenylalanine hydroxylase [Source:MGI Symbol;Acc:MGI:97473] | Pah | protein_coding |
| ENSMUSG00000020057 | 3587.55801 | -0.3416817 | 0.13213371 | 0.00048268 | 0.03165339 | Down | 71712 | DNA-damage regulated autophagy modulator 1 [Source:MGI Symbol;Acc:MGI:1918962] | Dram1 | protein_coding |
| ENSMUSG00000020155 | 14.9077016 | 3.41695656 | 1.44907717 | 0.00040247 | 0.02789861 | Up | 16533 | potassium large conductance calcium-activated channel, subfamily M, beta member 1 [Source:MGI Symbol;Acc:MGI:1334203] | Kcnmb1 | protein_coding |
| ENSMUSG00000020600 | 11.8713845 | 1.98045105 | 0.90593196 | 0.00071273 | 0.03874458 | Up | 328059 | solute carrier family 7 (cationic amino acid transporter, y+ system), member 15 [Source:MGI Symbol;Acc:MGI:3045351] | Slc7a15 | protein_coding |
| ENSMUSG00000020622 | 13.5811721 | 2.03135305 | 0.77416565 | 0.00026176 | 0.02180328 | Up | 70881 | 5'-nucleotidase, cytosolic IB [Source:MGI Symbol;Acc:MGI:1918131] | Nt5c1b | protein_coding |
| ENSMUSG00000020681 | 2579.14774 | 0.51549421 | 0.18485158 | 0.00021636 | 0.01918158 | Up | 11421 | angiotensin I converting enzyme (peptidyl-dipeptidase A) 1 [Source:MGI Symbol;Acc:MGI:87874] | Ace | protein_coding |
| ENSMUSG00000020875 | 53.5621027 | 2.86215228 | 0.70509689 | 1.79E-06 | 0.00153749 | Up | 15417 | homeobox B9 [Source:MGI Symbol;Acc:MGI:96190] | Hoxb9 | protein_coding |
| ENSMUSG00000020878 | 12.4004918 | 3.22971554 | 0.96562401 | 2.79E-05 | 0.00606434 | Up | 69297 | leucine rich repeat containing 46 [Source:MGI Symbol;Acc:MGI:1916547] | Lrrc46 | protein_coding |
| ENSMUSG00000020884 | 7.36174305 | -0.0067502 | 0.06282266 | 0.00108167 | 0.04812271 | Down | 11889 | asialoglycoprotein receptor 1 [Source:MGI Symbol;Acc:MGI:88081] | Asgr1 | protein_coding |
| ENSMUSG00000020904 | 16.7578002 | 5.27133237 | 1.90535766 | 0.00012421 | 0.01417611 | Up | 71860 | cilia and flagella associated protein 52 [Source:MGI Symbol;Acc:MGI:1919110] | Cfap52 | protein_coding |
| ENSMUSG00000020905 | 36.9772454 | 0.99762389 | 0.47967385 | 0.00094924 | 0.04475433 | Up | 216835 | ubiquitin specific peptidase 43 [Source:MGI Symbol;Acc:MGI:2444541] | Usp43 | protein_coding |
| ENSMUSG00000020926 | 86.3185916 | 2.2278114 | 0.63047924 | 1.48E-05 | 0.00419731 | Up | 11488 | a disintegrin and metallopeptidase domain 11 [Source:MGI Symbol;Acc:MGI:1098667] | Adam11 | protein_coding |
| ENSMUSG00000021071 | 20.3036788 | 1.62664487 | 0.40366141 | 2.38E-06 | 0.00153749 | Up | 94090 | tripartite motif-containing 9 [Source:MGI Symbol;Acc:MGI:2137354] | Trim9 | protein_coding |
| ENSMUSG00000021125 | 13.0445395 | 3.44261974 | 1.35300123 | 0.00026535 | 0.0219255 | Up | 11847 | arginase type II [Source:MGI Symbol;Acc:MGI:1330806] | Arg2 | protein_coding |
| ENSMUSG00000021135 | 9.46304451 | -0.0104088 | 0.06356117 | 0.00027839 | 0.02246358 | Down | 20493 | solute carrier family 10 (sodium/bile acid cotransporter family), member 1 [Source:MGI Symbol;Acc:MGI:97379] | Slc10a1 | protein_coding |
| ENSMUSG00000021338 | 193.811935 | 0.62403606 | 0.24603301 | 0.00037185 | 0.02639631 | Up | 68732 | capping protein regulator and myosin 1 linker 1 [Source:MGI Symbol;Acc:MGI:1915982] | Carmil1 | protein_coding |
| ENSMUSG00000021363 | 17.9321141 | 2.322829 | 0.965399 | 0.00041106 | 0.02823917 | Up | 17152 | male germ cell-associated kinase [Source:MGI Symbol;Acc:MGI:96913] | Mak | protein_coding |
| ENSMUSG00000021373 | 17.9706378 | 0.98013727 | 0.48492676 | 0.00111122 | 0.04862142 | Up | 67252 | CAP, adenylate cyclase-associated protein, 2 (yeast) [Source:MGI Symbol;Acc:MGI:1914502] | Cap2 | protein_coding |
| ENSMUSG00000021379 | 75.5340059 | 0.96204877 | 0.38305807 | 0.00035691 | 0.02568902 | Up | 15904 | inhibitor of DNA binding 4 [Source:MGI Symbol;Acc:MGI:99414] | Id4 | protein_coding |
| ENSMUSG00000021456 | 39.3341557 | 1.8067168 | 0.71013133 | 0.00030547 | 0.02372396 | Up | 14120 | fructose bisphosphatase 2 [Source:MGI Symbol;Acc:MGI:95491] | Fbp2 | protein_coding |
| ENSMUSG00000021638 | 12.4750925 | 3.4702048 | 1.22779821 | 0.00013358 | 0.01446075 | Up | 18260 | occludin [Source:MGI Symbol;Acc:MGI:106183] | Ocln | protein_coding |
| ENSMUSG00000021850 | 41.9256303 | 6.49096114 | 2.02604179 | 2.70E-05 | 0.00593543 | Up | 67082 | coiled-coil domain containing 198 [Source:MGI Symbol;Acc:MGI:1914332] | ccdc198 | protein_coding |
| ENSMUSG00000021879 | 44.7338858 | 5.50199813 | 1.29021575 | 5.98E-07 | 0.00102876 | Up | 110083 | dynein, axonemal, heavy chain 12 [Source:MGI Symbol;Acc:MGI:107720] | Dnah12 | protein_coding |
| ENSMUSG00000021922 | 88.4806934 | -0.0304747 | 0.07378305 | 4.83E-05 | 0.00890019 | Down | 16427 | inter alpha-trypsin inhibitor, heavy chain 4 [Source:MGI Symbol;Acc:MGI:109536] | Itih4 | protein_coding |
| ENSMUSG00000022018 | 7242.81954 | -0.406965 | 0.18449678 | 0.00081805 | 0.0416602 | Down | 66214 | regulator of cell cycle [Source:MGI Symbol;Acc:MGI:1913464] | Rgcc | protein_coding |
| ENSMUSG00000022041 | 57.3406617 | 1.97778355 | 0.86492416 | 0.00054258 | 0.03345719 | Up | 110902 | cholinergic receptor, nicotinic, alpha polypeptide 2 (neuronal) [Source:MGI Symbol;Acc:MGI:87886] | Chrna2 | protein_coding |
| ENSMUSG00000022056 | 29.6293181 | 0.00082607 | 0.06229918 | 3.80E-07 | 0.00091814 | Up | 11500 | a disintegrin and metallopeptidase domain 7 [Source:MGI Symbol;Acc:MGI:107247] | Adam7 | protein_coding |
| ENSMUSG00000022174 | 1567.12453 | -0.3858331 | 0.17740847 | 0.0010948 | 0.0483231 | Down | 13135 | defender against cell death 1 [Source:MGI Symbol;Acc:MGI:101912] | Dad1 | protein_coding |
| ENSMUSG00000022237 | 54.3044016 | 0.88103135 | 0.36989143 | 0.00050653 | 0.03229422 | Up | 67434 | ankyrin repeat domain 33B [Source:MGI Symbol;Acc:MGI:1917904] | Ankrd33b | protein_coding |
| ENSMUSG00000022270 | 107.097215 | 0.75568036 | 0.34713598 | 0.00081023 | 0.04153085 | Up | 66270 | reticulophagy regulator 1 [Source:MGI Symbol;Acc:MGI:1913520] | Retreg1 | protein_coding |
| ENSMUSG00000022297 | 220.768206 | 1.10750029 | 0.3395653 | 3.94E-05 | 0.0077516 | Up | 14368 | frizzled class receptor 6 [Source:MGI Symbol;Acc:MGI:108474] | Fzd6 | protein_coding |
| ENSMUSG00000022376 | 73.3654468 | 5.09201439 | 1.27374352 | 1.78E-06 | 0.00153749 | Up | 11514 | adenylate cyclase 8 [Source:MGI Symbol;Acc:MGI:1341110] | Adcy8 | protein_coding |
| ENSMUSG00000022449 | 12.9926263 | 2.42641087 | 0.80451279 | 8.50E-05 | 0.0120438 | Up | 223838 | a disintegrin-like and metallopeptidase (reprolysin type) with thrombospondin type 1 motif, 20 [Source:MGI Symbol;Acc:MGI:2660628] | Adamts20 | protein_coding |
| ENSMUSG00000022759 | 39.2383342 | 4.04788908 | 1.37344888 | 7.65E-05 | 0.01136906 | Up | 74685 | leucine rich repeat containing 74B [Source:MGI Symbol;Acc:MGI:1921935] | Lrrc74b | protein_coding |
| ENSMUSG00000022868 | 91.3162011 | -0.0096461 | 0.06338094 | 0.00039325 | 0.02763057 | Down | 11625 | alpha-2-HS-glycoprotein [Source:MGI Symbol;Acc:MGI:107189] | Ahsg | protein_coding |
| ENSMUSG00000022871 | 21.1995371 | -0.024829 | 0.06965401 | 0.00031134 | 0.02372396 | Down | 59083 | fetuin beta [Source:MGI Symbol;Acc:MGI:1890221] | Fetub | protein_coding |
| ENSMUSG00000022949 | 93.5405227 | 3.79197309 | 1.13655674 | 2.30E-05 | 0.00540757 | Up | 209195 | chloride intracellular channel 6 [Source:MGI Symbol;Acc:MGI:2146607] | Clic6 | protein_coding |
| ENSMUSG00000022995 | 303.250617 | 1.12124562 | 0.39567188 | 0.00014632 | 0.01503743 | Up | 13800 | ENAH actin regulator [Source:MGI Symbol;Acc:MGI:108360] | Enah | protein_coding |
| ENSMUSG00000023032 | 70.9569469 | 1.24654374 | 0.4861054 | 0.00030392 | 0.02372396 | Up | 59033 | solute carrier family 4 (anion exchanger), member 8 [Source:MGI Symbol;Acc:MGI:1928745] | Slc4a8 | protein_coding |
| ENSMUSG00000023084 | 38.9480952 | 4.76497706 | 1.19114525 | 1.91E-06 | 0.00153749 | Up | 74485 | leucine rich repeat containing 71 [Source:MGI Symbol;Acc:MGI:1921735] | Lrrc71 | protein_coding |
| ENSMUSG00000023243 | 86.6188828 | 1.35441865 | 0.45942485 | 0.00010561 | 0.01292602 | Up | 16529 | potassium channel, subfamily K, member 5 [Source:MGI Symbol;Acc:MGI:1336175] | Kcnk5 | protein_coding |
| ENSMUSG00000023267 | 70.3766381 | -0.0396373 | 0.08193991 | 0.00091179 | 0.04407147 | Down | 14409 | gamma-aminobutyric acid (GABA) C receptor, subunit rho 2 [Source:MGI Symbol;Acc:MGI:95626] | Gabrr2 | protein_coding |
| ENSMUSG00000023931 | 25.0966408 | 1.56004022 | 0.6072583 | 0.00029668 | 0.02339093 | Up | 211482 | EF hand domain family, member B [Source:MGI Symbol;Acc:MGI:3045296] | Efhb | protein_coding |
| ENSMUSG00000023972 | 170.510171 | 1.10871215 | 0.2749213 | 2.17E-06 | 0.00153749 | Up | 71461 | PTK7 protein tyrosine kinase 7 [Source:MGI Symbol;Acc:MGI:1918711] | Ptk7 | protein_coding |

| | | | | | | | | | | |
|---|---|---|---|---|---|---|---|---|---|---|
| ENSMUSG00000023999 | 9.88788243 | 2.66543798 | 1.3415271 | 0.00094012 | 0.04454146 | Up | 319991 | kinesin family member 6 [Source:MGI Symbol;Acc:MGI:1098238] | Kif6 | protein_coding |
| ENSMUSG00000024033 | 29.6612365 | 3.21291926 | 0.88794934 | 9.90E-06 | 0.00346458 | Up | 22092 | radial spoke head 1 homolog (Chlamydomonas) [Source:MGI Symbol;Acc:MGI:1194909] | Rsph1 | protein_coding |
| ENSMUSG00000024059 | 71.9060969 | 0.95389559 | 0.31502004 | 8.52E-05 | 0.0120438 | Up | 78785 | CAP-GLY domain containing linker protein family, member 4 [Source:MGI Symbol;Acc:MGI:1919100] | Clip4 | protein_coding |
| ENSMUSG00000024064 | 13.4621899 | 3.06487874 | 1.27544399 | 0.00037932 | 0.02683443 | Up | 71685 | polypeptide N-acetylgalactosaminyltransferase 14 [Source:MGI Symbol;Acc:MGI:1918935] | Galnt14 | protein_coding |
| ENSMUSG00000024112 | 86.5704808 | 3.70722716 | 1.17360904 | 4.16E-05 | 0.00801702 | Up | 58226 | calcium channel, voltage-dependent, T type, alpha 1H subunit [Source:MGI Symbol;Acc:MGI:1928842] | Cacna1h | protein_coding |
| ENSMUSG00000024180 | 244.314413 | 0.68363081 | 0.26790054 | 0.00034799 | 0.02554802 | Up | 60455 | post-glycosylphosphatidylinositol attachment to proteins 6 [Source:MGI Symbol;Acc:MGI:1926283] | Pgap6 | protein_coding |
| ENSMUSG00000024186 | 31.4428123 | 1.28848517 | 0.5745863 | 0.00066387 | 0.03788268 | Up | 50782 | regulator of G-protein signaling 11 [Source:MGI Symbol;Acc:MGI:1354739] | Rgs11 | protein_coding |
| ENSMUSG00000024388 | 29.7538175 | 2.19416857 | 0.85291468 | 0.00027426 | 0.02230486 | Up | 17922 | myosin VIIB [Source:MGI Symbol;Acc:MGI:107709] | Myo7b | protein_coding |
| ENSMUSG00000024665 | 355.883816 | 0.83278993 | 0.37084027 | 0.00070408 | 0.03868938 | Up | 56473 | fatty acid desaturase 2 [Source:MGI Symbol;Acc:MGI:1930079] | Fads2 | protein_coding |
| ENSMUSG00000024697 | 11.6521305 | 1.69251181 | 0.80131959 | 0.00088587 | 0.04345904 | Up | 14675 | guanine nucleotide binding protein, alpha 14 [Source:MGI Symbol;Acc:MGI:95769] | Gna14 | protein_coding |
| ENSMUSG00000024790 | 208.393128 | -0.5492303 | 0.18841576 | 0.00012848 | 0.01440481 | Down | 66406 | SAC3 domain containing 1 [Source:MGI Symbol;Acc:MGI:1913656] | Sac3d1 | protein_coding |
| ENSMUSG00000025059 | 353.811457 | 1.01562177 | 0.45605679 | 0.00069605 | 0.03860567 | Up | 14933 | glycerol kinase [Source:MGI Symbol;Acc:MGI:106594] | Gk | protein_coding |
| ENSMUSG00000025165 | 5.85857746 | 3.52609598 | 1.44103905 | 0.00043411 | 0.02902097 | Up | 209588 | secreted and transmembrane 1A [Source:MGI Symbol;Acc:MGI:2384805] | Sectm1a | protein_coding |
| ENSMUSG00000025504 | 129.859361 | 1.17593375 | 0.50767542 | 0.00055726 | 0.03412613 | Up | 98845 | EPS8-like 2 [Source:MGI Symbol;Acc:MGI:2138828] | Eps8l2 | protein_coding |
| ENSMUSG00000025576 | 13.3888932 | 3.71797184 | 1.54000744 | 0.00034877 | 0.02554802 | Up | 52897 | RNA binding protein, fox-1 homolog (C. elegans) 3 [Source:MGI Symbol;Acc:MGI:106368] | Rbfox3 | protein_coding |
| ENSMUSG00000025665 | 4.11986373 | 3.76449053 | 1.60989691 | 0.00055839 | 0.03412613 | Up | 67071 | ribosomal protein S6 kinase polypeptide 6 [Source:MGI Symbol;Acc:MGI:1914321] | Rps6ka6 | protein_coding |
| ENSMUSG00000025900 | 10.466168 | 5.06317205 | 1.79774566 | 0.00014483 | 0.01501778 | Up | 19888 | retinitis pigmentosa 1 (human) [Source:MGI Symbol;Acc:MGI:1341105] | Rp1 | protein_coding |
| ENSMUSG00000026175 | 37.9671692 | 4.4342344 | 1.31988552 | 2.08E-05 | 0.00511821 | Up | 22349 | villin 1 [Source:MGI Symbol;Acc:MGI:98930] | Vil1 | protein_coding |
| ENSMUSG00000026220 | 11.4131745 | 1.53284546 | 0.68501286 | 0.00069602 | 0.03860567 | Up | 71781 | solute carrier family 16 (monocarboxylic acid transporters), member 14 [Source:MGI Symbol;Acc:MGI:1919031] | Slc16a14 | protein_coding |
| ENSMUSG00000026235 | 64.9739072 | 0.64484111 | 0.29375012 | 0.00083042 | 0.04196648 | Up | 13838 | Eph receptor A4 [Source:MGI Symbol;Acc:MGI:98277] | Epha4 | protein_coding |
| ENSMUSG00000026303 | 106.346786 | 2.77033406 | 0.7686459 | 1.05E-05 | 0.0035471 | Up | 171531 | melanophilin [Source:MGI Symbol;Acc:MGI:2176380] | Mlph | protein_coding |
| ENSMUSG00000026421 | 2846.61692 | -0.3197878 | 0.10291419 | 9.71E-05 | 0.01275873 | Down | 13007 | cysteine and glycine-rich protein 1 [Source:MGI Symbol;Acc:MGI:88549] | Csrp1 | protein_coding |
| ENSMUSG00000026479 | 82.0858902 | 1.1563104 | 0.41316201 | 0.00016601 | 0.01594971 | Up | 16782 | laminin, gamma 2 [Source:MGI Symbol;Acc:MGI:99913] | Lamc2 | protein_coding |
| ENSMUSG00000026494 | 16.0461404 | 2.34843223 | 0.75836152 | 6.66E-05 | 0.01034153 | Up | 269152 | kinesin family member 26B [Source:MGI Symbol;Acc:MGI:2447076] | Kif26b | protein_coding |
| ENSMUSG00000026546 | 32.7162158 | 1.6615767 | 0.59846707 | 0.0001686 | 0.01605009 | Up | 71870 | cilia and flagella associated protein 45 [Source:MGI Symbol;Acc:MGI:1919120] | Cfap45 | protein_coding |
| ENSMUSG00000026611 | 9.06901398 | 2.72910353 | 0.97187606 | 0.00015393 | 0.01548545 | Up | 74717 | spermatogenesis associated 17 [Source:MGI Symbol;Acc:MGI:1921967] | Spata17 | protein_coding |
| ENSMUSG00000026679 | 23.2659205 | 2.65075937 | 0.86224968 | 6.51E-05 | 0.01027168 | Up | 71233 | enkurin, TRPC channel interacting protein [Source:MGI Symbol;Acc:MGI:1918483] | Enkur | protein_coding |
| ENSMUSG00000026715 | 31.1714336 | -0.0131429 | 0.06433211 | 9.88E-05 | 0.01275873 | Down | 11905 | serine (or cysteine) peptidase inhibitor, clade C (antithrombin), member 1 [Source:MGI Symbol;Acc:MGI:88095] | Serpinc1 | protein_coding |
| ENSMUSG00000026822 | 386.57899 | 2.46233574 | 0.8082661 | 6.99E-05 | 0.01069539 | Up | 16819 | lipocalin 2 [Source:MGI Symbol;Acc:MGI:96757] | Lcn2 | protein_coding |
| ENSMUSG00000026870 | 11.7566795 | 3.50681212 | 1.20137372 | 0.00010366 | 0.01292602 | Up | 77996 | cutA divalent cation tolerance homolog-like [Source:MGI Symbol;Acc:MGI:1925246] | Cutal | protein_coding |
| ENSMUSG00000026994 | 14.3096834 | 4.46675984 | 1.30367901 | 2.00E-05 | 0.00497925 | Up | 14425 | polypeptide N-acetylgalactosaminyltransferase 3 [Source:MGI Symbol;Acc:MGI:894695] | Galnt3 | protein_coding |
| ENSMUSG00000027001 | 189.722148 | -0.3136193 | 0.14115985 | 0.00107972 | 0.04812271 | Down | 68082 | dual specificity phosphatase 19 [Source:MGI Symbol;Acc:MGI:1915332] | Dusp19 | protein_coding |
| ENSMUSG00000027122 | 349.234772 | 0.2334392 | 0.09785938 | 0.00095807 | 0.04475433 | Up | 212772 | ADP-ribosylation factor-like 14 effector protein [Source:MGI Symbol;Acc:MGI:1926020] | Arl14ep | protein_coding |
| ENSMUSG00000027315 | 22.0230929 | 2.90855778 | 1.09140286 | 0.00020102 | 0.01829313 | Up | 20732 | serine protease inhibitor, Kunitz type 1 [Source:MGI Symbol;Acc:MGI:1338033] | Spint1 | protein_coding |
| ENSMUSG00000027356 | 48.6986915 | 6.80941973 | 2.08231998 | 1.92E-05 | 0.0049459 | Up | 241639 | fermitin family member 1 [Source:MGI Symbol;Acc:MGI:2443583] | Fermt1 | protein_coding |
| ENSMUSG00000027360 | 109.714661 | 1.34282091 | 0.50733182 | 0.00024034 | 0.02068642 | Up | 15186 | histidine decarboxylase [Source:MGI Symbol;Acc:MGI:96062] | Hdc | protein_coding |
| ENSMUSG00000027375 | 15.8235822 | 2.50254325 | 1.15378312 | 0.00067478 | 0.038189 | Up | 17153 | myelin and lymphocyte protein, T cell differentiation protein [Source:MGI Symbol;Acc:MGI:892970] | Mal | protein_coding |
| ENSMUSG00000027376 | 61.5589822 | 0.00223264 | 0.06235073 | 0.00017509 | 0.01629206 | Up | 192212 | prominin 2 [Source:MGI Symbol;Acc:MGI:2138997] | Prom2 | protein_coding |
| ENSMUSG00000027463 | 79.2235628 | 1.27680386 | 0.38926344 | 3.77E-05 | 0.00748576 | Up | 69698 | solute carrier protein family 52, member 3 [Source:MGI Symbol;Acc:MGI:1916948] | Slc52a3 | protein_coding |
| ENSMUSG00000027536 | 46.8873758 | 1.08537145 | 0.49954634 | 0.00077997 | 0.04068635 | Up | 66371 | charged multivesicular body protein 4C [Source:MGI Symbol;Acc:MGI:1913621] | Chmp4c | protein_coding |
| ENSMUSG00000027562 | 59.2550582 | 1.52242529 | 0.57144308 | 0.00022994 | 0.01995702 | Up | 12349 | carbonic anhydrase 2 [Source:MGI Symbol;Acc:MGI:88269] | Car2 | protein_coding |
| ENSMUSG00000027702 | 9.539582 | 4.87181786 | 1.82531735 | 0.00021223 | 0.01897812 | Up | 71827 | leucine rich repeat containing 34 [Source:MGI Symbol;Acc:MGI:1919077] | Lrrc34 | protein_coding |
| ENSMUSG00000027712 | 10641.2925 | -0.3755844 | 0.17783349 | 0.0011556 | 0.04971037 | Down | 11747 | annexin A5 [Source:MGI Symbol;Acc:MGI:106008] | Anxa5 | protein_coding |
| ENSMUSG00000027762 | 1514.49608 | -0.4125176 | 0.18340979 | 0.00083541 | 0.04196648 | Down | 84112 | succinate receptor 1 [Source:MGI Symbol;Acc:MGI:1934135] | Sucnr1 | protein_coding |
| ENSMUSG00000027886 | 4.9543769 | 4.86789288 | 2.64749928 | 0.00079433 | 0.04102126 | Up | 75504 | cilia and flagella associated protein 276 [Source:MGI Symbol;Acc:MGI:1922754] | Cfap276 | protein_coding |
| ENSMUSG00000027894 | 30.2295038 | 4.8559582 | 1.3519736 | 9.39E-06 | 0.00334496 | Up | 229706 | solute carrier family 6 (neurotransmitter transporter), member 17 [Source:MGI Symbol;Acc:MGI:2442535] | Slc6a17 | protein_coding |
| ENSMUSG00000028278 | 23.8291966 | 3.97290997 | 0.77491992 | 1.29E-08 | 5.31E-05 | Up | 52187 | Ras-related GTP binding D [Source:MGI Symbol;Acc:MGI:1098604] | Rragd | protein_coding |
| ENSMUSG00000028287 | 5.98426055 | 3.20579849 | 1.38812672 | 0.0005509 | 0.03386865 | Up | 75471 | RIKEN cDNA 1700009N14 gene [Source:MGI Symbol;Acc:MGI:1922721] | 1700009N14Rik | protein_coding |
| ENSMUSG00000028328 | 76.9841971 | 0.98161331 | 0.44014938 | 0.00069492 | 0.03860567 | Up | 21916 | tropomodulin 1 [Source:MGI Symbol;Acc:MGI:98775] | Tmod1 | protein_coding |
| ENSMUSG00000028392 | 19.5166166 | 2.98326223 | 0.95954877 | 5.68E-05 | 0.00963581 | Up | 192120 | B-box and SPRY domain containing [Source:MGI Symbol;Acc:MGI:2177191] | Bspry | protein_coding |
| ENSMUSG00000028441 | 9.68949072 | 3.92687153 | 1.75420414 | 0.00052363 | 0.03277795 | Up | 73721 | RIKEN cDNA 1110017D15 gene [Source:MGI Symbol;Acc:MGI:1920971] | 1110017D15Rik | protein_coding |
| ENSMUSG00000028488 | 13.2496719 | 3.89274133 | 1.33303041 | 9.76E-05 | 0.01275873 | Up | 20404 | SH3-domain GRB2-like 2 [Source:MGI Symbol;Acc:MGI:700009] | Sh3gl2 | protein_coding |
| ENSMUSG00000028544 | 4.74337688 | 4.22497982 | 2.01326318 | 0.00073053 | 0.03923737 | Up | 230612 | solute carrier family 5 (sodium/glucose cotransporter), member 9 [Source:MGI Symbol;Acc:MGI:2140201] | Slc5a9 | protein_coding |
| ENSMUSG00000028555 | 26.2697239 | 2.20689825 | 0.79264225 | 0.00015743 | 0.01560614 | Up | 230603 | tetratricopeptide repeat domain 39A [Source:MGI Symbol;Acc:MGI:2444350] | Ttc39a | protein_coding |

| | | | | | | | | | | |
|---|---|---|---|---|---|---|---|---|---|---|
| ENSMUSG00000028572 | 83.1168614 | 0.99810159 | 0.50336106 | 0.00115624 | 0.04971037 | Up | 77963 | hook microtubule tethering protein 1 [Source:MGI Symbol;Acc:MGI:1925213] | Hook1 | protein_coding |
| ENSMUSG00000028755 | 25.3772973 | 2.76932907 | 1.00784229 | 0.00016341 | 0.01580678 | Up | 72269 | cytidine deaminase [Source:MGI Symbol;Acc:MGI:1919519] | Cda | protein_coding |
| ENSMUSG00000028836 | 65.6098146 | 4.03964 | 0.88835176 | 1.93E-07 | 0.00066492 | Up | 230810 | solute carrier family 30 (zinc transporter), member 2 [Source:MGI Symbol;Acc:MGI:106637] | Slc30a2 | protein_coding |
| ENSMUSG00000028838 | 28.8985045 | 1.29505705 | 0.54739199 | 0.00050264 | 0.03214562 | Up | 56219 | exostosin-like glycosyltransferase 1 [Source:MGI Symbol;Acc:MGI:1888742] | Extl1 | protein_coding |
| ENSMUSG00000028943 | 216.031313 | 1.29390525 | 0.46756581 | 0.00017287 | 0.01623181 | Up | 56226 | espin [Source:MGI Symbol;Acc:MGI:1861630] | Espn | protein_coding |
| ENSMUSG00000029026 | 12.5019304 | 2.94998367 | 1.06543408 | 0.00015884 | 0.01562446 | Up | 22062 | transformation related protein 73 [Source:MGI Symbol;Acc:MGI:1336991] | Trp73 | protein_coding |
| ENSMUSG00000029030 | 2759.22054 | -0.2648048 | 0.10537906 | 0.00059114 | 0.03549746 | Down | 67808 | transformation related protein 63 regulated like [Source:MGI Symbol;Acc:MGI:1915058] | Tprgl | protein_coding |
| ENSMUSG00000029032 | 28.4236782 | 2.67910448 | 0.83925404 | 4.44E-05 | 0.00833777 | Up | 230972 | Rho guanine nucleotide exchange factor (GEF) 16 [Source:MGI Symbol;Acc:MGI:2446219] | Arhgef16 | protein_coding |
| ENSMUSG00000029049 | 60.4343945 | 0.80827293 | 0.38708228 | 0.00095592 | 0.04475433 | Up | 76866 | MORN repeat containing 1 [Source:MGI Symbol;Acc:MGI:1924116] | Morn1 | protein_coding |
| ENSMUSG00000029074 | 10.7248047 | 4.96780016 | 1.95221345 | 0.00026107 | 0.02180328 | Up | 330010 | tubulin tyrosine ligase-like family, member 10 [Source:MGI Symbol;Acc:MGI:1921855] | Ttll10 | protein_coding |
| ENSMUSG00000029086 | 213.713955 | 4.18508007 | 1.03024056 | 1.54E-06 | 0.00153749 | Up | 19126 | prominin 1 [Source:MGI Symbol;Acc:MGI:1100886] | Prom1 | protein_coding |
| ENSMUSG00000029093 | 92.4161625 | 2.97745451 | 0.86403605 | 1.80E-05 | 0.00475658 | Up | 81840 | sortilin-related VPS10 domain containing receptor 2 [Source:MGI Symbol;Acc:MGI:1932289] | Sorcs2 | protein_coding |
| ENSMUSG00000029110 | 2490.67859 | -0.209431 | 0.08021917 | 0.00083651 | 0.04196648 | Down | 19822 | ring finger protein 4 [Source:MGI Symbol;Acc:MGI:1201691] | Rnf4 | protein_coding |
| ENSMUSG00000029188 | 10.4780027 | 3.82612016 | 1.77684798 | 0.00061199 | 0.03601664 | Up | 20531 | solute carrier family 34 (sodium phosphate), member 2 [Source:MGI Symbol;Acc:MGI:1342284] | Slc34a2 | protein_coding |
| ENSMUSG00000029206 | 9.79671542 | 1.43646512 | 0.67745026 | 0.0009059 | 0.04407147 | Up | 70918 | NOL1/NOP2/Sun domain family, member 7 [Source:MGI Symbol;Acc:MGI:1918168] | Nsun7 | protein_coding |
| ENSMUSG00000029291 | 437.518394 | 0.2914696 | 0.11047622 | 0.00041087 | 0.02823917 | Up | 52822 | RUN and FYVE domain containing 3 [Source:MGI Symbol;Acc:MGI:106484] | Rufy3 | protein_coding |
| ENSMUSG00000029304 | 74.5562078 | 2.46082646 | 0.91820908 | 0.00020351 | 0.01842044 | Up | 20750 | secreted phosphoprotein 1 [Source:MGI Symbol;Acc:MGI:98389] | Spp1 | protein_coding |
| ENSMUSG00000029334 | 11.3520524 | 1.38012577 | 0.65740388 | 0.000993 | 0.0458891 | Up | 19092 | protein kinase, cGMP-dependent, type II [Source:MGI Symbol;Acc:MGI:108173] | Prkg2 | protein_coding |
| ENSMUSG00000029359 | 36.5852866 | 1.08214304 | 0.52794147 | 0.00101122 | 0.0465049 | Up | 57816 | tescalcin [Source:MGI Symbol;Acc:MGI:1930803] | Tesc | protein_coding |
| ENSMUSG00000029368 | 1167.7793 | -0.0167788 | 0.06565564 | 2.67E-05 | 0.00593543 | Down | 11657 | albumin [Source:MGI Symbol;Acc:MGI:87991] | Alb | protein_coding |
| ENSMUSG00000029438 | 321.987653 | 0.78976113 | 0.26975559 | 0.00011523 | 0.0137588 | Up | 77045 | B cell CLL/lymphoma 7A [Source:MGI Symbol;Acc:MGI:1924295] | Bcl7a | protein_coding |
| ENSMUSG00000029445 | 46.2315539 | -0.0213346 | 0.06779379 | 1.48E-05 | 0.00419731 | Down | 15445 | 4-hydroxyphenylpyruvic acid dioxygenase [Source:MGI Symbol;Acc:MGI:96213] | Hpd | protein_coding |
| ENSMUSG00000029477 | 4.6799996 | 7.20475186 | 2.74129954 | 1.42E-05 | 0.00412529 | Up | 74890 | MORN repeat containing 3 [Source:MGI Symbol;Acc:MGI:1922140] | Morn3 | protein_coding |
| ENSMUSG00000029610 | 201.001545 | -0.4508272 | 0.12909966 | 2.13E-05 | 0.00518515 | Down | 231872 | aminoacyl tRNA synthetase complex-interacting multifunctional protein 2 [Source:MGI Symbol;Acc:MGI:2385237] | Aimp2 | protein_coding |
| ENSMUSG00000029769 | 26.0780477 | 2.09853797 | 0.7276849 | 0.00012045 | 0.0140577 | Up | 232664 | coiled-coil domain containing 136 [Source:MGI Symbol;Acc:MGI:1918128] | Ccdc136 | protein_coding |
| ENSMUSG00000030077 | 15.7231626 | 2.73502665 | 0.93411462 | 0.00010365 | 0.01292602 | Up | 12661 | cell adhesion molecule L1-like [Source:MGI Symbol;Acc:MGI:1098266] | Chl1 | protein_coding |
| ENSMUSG00000030104 | 4127.86582 | -0.2755313 | 0.11858836 | 0.00093044 | 0.04438826 | Down | 192193 | ER degradation enhancer, mannosidase alpha-like 1 [Source:MGI Symbol;Acc:MGI:2180139] | Edem1 | protein_coding |
| ENSMUSG00000030125 | 26.2795137 | 2.68151037 | 0.91340475 | 9.69E-05 | 0.01275873 | Up | 16977 | leucine rich repeat containing 23 [Source:MGI Symbol;Acc:MGI:1315192] | Lrrc23 | protein_coding |
| ENSMUSG00000030276 | 89.9851996 | 1.49388339 | 0.53223359 | 0.00015518 | 0.01548545 | Up | 101100 | tubulin tyrosine ligase-like family, member 3 [Source:MGI Symbol;Acc:MGI:2141418] | Ttll3 | protein_coding |
| ENSMUSG00000030340 | 164.924421 | 1.36900721 | 0.55708755 | 0.00036949 | 0.02631941 | Up | 20276 | sodium channel, nonvoltage-gated 1 alpha [Source:MGI Symbol;Acc:MGI:101782] | Scnn1a | protein_coding |
| ENSMUSG00000030492 | 50.283293 | 0.00223474 | 0.06235081 | 0.00030834 | 0.02372396 | Up | 30962 | solute carrier family 7 (cationic amino acid transporter, y+ system), member 9 [Source:MGI Symbol;Acc:MGI:1353656] | Slc7a9 | protein_coding |
| ENSMUSG00000030630 | 2745.06256 | -0.4044171 | 0.18138836 | 0.00089928 | 0.04391566 | Down | 14085 | fumarylacetoacetate hydrolase [Source:MGI Symbol;Acc:MGI:95482] | Fah | protein_coding |
| ENSMUSG00000030650 | 46.0496448 | 2.77309481 | 0.74575253 | 6.95E-06 | 0.00281487 | Up | 74424 | transmembrane channel-like gene family 5 [Source:MGI Symbol;Acc:MGI:1921674] | Tmc5 | protein_coding |
| ENSMUSG00000030701 | 93.7573564 | 1.45184538 | 0.62077925 | 0.0005203 | 0.03266829 | Up | 27276 | pleckstrin homology domain containing, family B (evectins) member 1 [Source:MGI Symbol;Acc:MGI:1351469] | Plekhb1 | protein_coding |
| ENSMUSG00000030800 | 21.2441499 | 3.57938584 | 1.25236642 | 0.00011077 | 0.0133816 | Up | 76560 | protease, serine 8 (prostasin) [Source:MGI Symbol;Acc:MGI:1923810] | Prss8 | protein_coding |
| ENSMUSG00000030895 | 72.6395131 | -0.0099323 | 0.06345785 | 0.00012901 | 0.01440481 | Down | 15458 | hemopexin [Source:MGI Symbol;Acc:MGI:105112] | Hpx | protein_coding |
| ENSMUSG00000031022 | 11.2030015 | 5.02486669 | 1.76730621 | 0.00013371 | 0.01446075 | Up | 57355 | cDNA sequence BC051019 [Source:MGI Symbol;Acc:MGI:1928824] | BC051019 | protein_coding |
| ENSMUSG00000031027 | 10.6701375 | 4.63840672 | 1.75981525 | 0.00021031 | 0.01888824 | Up | 117229 | serine/threonine kinase 33 [Source:MGI Symbol;Acc:MGI:2152419] | Stk33 | protein_coding |
| ENSMUSG00000031099 | 30.3257207 | 1.34513258 | 0.67942223 | 0.00111322 | 0.04862142 | Up | 93761 | SWI/SNF related, matrix associated, actin dependent regulator of chromatin, subfamily a, member 1 [Source:MGI Symbol;Acc:MGI:1935127] | Smarca1 | protein_coding |
| ENSMUSG00000031138 | 3.26852196 | -0.0062337 | 0.06275129 | 0.00013627 | 0.01450975 | Down | 14071 | coagulation factor IX [Source:MGI Symbol;Acc:MGI:88384] | F9 | protein_coding |
| ENSMUSG00000031150 | 50.2546696 | 0.870325 | 0.37942921 | 0.00063687 | 0.03674817 | Up | 54648 | coiled-coil domain containing 120 [Source:MGI Symbol;Acc:MGI:1859619] | Ccdc120 | protein_coding |
| ENSMUSG00000031367 | 406.567257 | 0.94235591 | 0.42270199 | 0.00070442 | 0.03868938 | Up | 108012 | adaptor-related protein complex 1, sigma 2 subunit [Source:MGI Symbol;Acc:MGI:1889383] | Ap1s2 | protein_coding |
| ENSMUSG00000031432 | 1617.22709 | -0.556856 | 0.21000459 | 0.00027325 | 0.02230486 | Down | 19139 | phosphoribosyl pyrophosphate synthetase 1 [Source:MGI Symbol;Acc:MGI:97775] | Prps1 | protein_coding |
| ENSMUSG00000031548 | 456.020976 | 1.26362257 | 0.44634982 | 0.00014441 | 0.01501778 | Up | 20377 | secreted frizzled-related protein 1 [Source:MGI Symbol;Acc:MGI:892014] | Sfrp1 | protein_coding |
| ENSMUSG00000031554 | 11.2820411 | 1.89277243 | 0.92149194 | 0.00095895 | 0.04475433 | Up | 11499 | a disintegrin and metallopeptidase domain 5 [Source:MGI Symbol;Acc:MGI:104730] | Adam5 | protein_coding |
| ENSMUSG00000031642 | 277.052192 | 0.41519792 | 0.17984725 | 0.00068413 | 0.03840247 | Up | 59009 | SH3 domain containing ring finger 1 [Source:MGI Symbol;Acc:MGI:1913066] | Sh3rf1 | protein_coding |
| ENSMUSG00000031760 | 21.3232974 | 0.00317681 | 0.06241138 | 0.00035919 | 0.02576333 | Up | 17751 | metallothionein 3 [Source:MGI Symbol;Acc:MGI:97173] | Mt3 | protein_coding |
| ENSMUSG00000031778 | 112.576786 | 1.26103728 | 0.42408734 | 9.74E-05 | 0.01275873 | Up | 20312 | chemokine (C-X3-C motif) ligand 1 [Source:MGI Symbol;Acc:MGI:1097153] | Cx3cl1 | protein_coding |
| ENSMUSG00000031831 | 11.2593273 | 6.94699006 | 1.86776927 | 1.66E-06 | 0.00153749 | Up | 68270 | dynein, axonemal assembly factor 1 [Source:MGI Symbol;Acc:MGI:1915520] | Dnaaf1 | protein_coding |
| ENSMUSG00000031853 | 40.979788 | 2.95143601 | 0.92762252 | 4.38E-05 | 0.00830512 | Up | 234878 | mitogen-activated protein kinase kinase kinase 21 [Source:MGI Symbol;Acc:MGI:2385307] | Map3k21 | protein_coding |
| ENSMUSG00000031881 | 102.787599 | 0.00137063 | 0.06231351 | 0.00056267 | 0.03428628 | Up | 12556 | cadherin 16 [Source:MGI Symbol;Acc:MGI:106671] | Cdh16 | protein_coding |
| ENSMUSG00000031972 | 1466.38697 | -0.0332038 | 0.0762621 | 7.69E-06 | 0.00290098 | Down | 11459 | actin alpha 1, skeletal muscle [Source:MGI Symbol;Acc:MGI:87902] | Acta1 | protein_coding |

| | | | | | | | | | | |
|---|---|---|---|---|---|---|---|---|---|---|
| ENSMUSG00000032010 | 236.356182 | 0.86511777 | 0.33590738 | 0.00030703 | 0.02372396 | Up | 53376 | ubiquitin specific peptidase 2 [Source:MGI Symbol;Acc:MGI:1858178] | Usp2 | protein_coding |
| ENSMUSG00000032028 | 21.825973 | 0.98466679 | 0.44699078 | 0.00078308 | 0.04074606 | Up | 78252 | neurexophilin and PC-esterase domain family, member 2 [Source:MGI Symbol;Acc:MGI:1925502] | Nxpe2 | protein_coding |
| ENSMUSG00000032068 | 63.9966976 | 4.40676032 | 1.90242314 | 0.00030682 | 0.02372396 | Up | 76509 | placenta expressed transcript 1 [Source:MGI Symbol;Acc:MGI:1923759] | Plet1 | protein_coding |
| ENSMUSG00000032083 | 139.454666 | -0.0073532 | 0.06292298 | 0.00076948 | 0.04049479 | Down | 11806 | apolipoprotein A-I [Source:MGI Symbol;Acc:MGI:88049] | Apoa1 | protein_coding |
| ENSMUSG00000032221 | 20.4300803 | 2.28492076 | 0.83212868 | 0.00016375 | 0.01580678 | Up | 17427 | meiosis-specific nuclear structural protein 1 [Source:MGI Symbol;Acc:MGI:107933] | Mns1 | protein_coding |
| ENSMUSG00000032246 | 123.189043 | 1.45981958 | 0.46636726 | 5.90E-05 | 0.00963581 | Up | 75600 | calmodulin-like 4 [Source:MGI Symbol;Acc:MGI:1922850] | Calml4 | protein_coding |
| ENSMUSG00000032271 | 3503.70923 | -0.4356237 | 0.213059 | 0.00110165 | 0.04841886 | Down | 18113 | nicotinamide N-methyltransferase [Source:MGI Symbol;Acc:MGI:1099443] | Nnmt | protein_coding |
| ENSMUSG00000032278 | 23.2605836 | 4.14094928 | 1.19180528 | 1.54E-05 | 0.00431237 | Up | 74090 | progestin and adipoQ receptor family member V [Source:MGI Symbol;Acc:MGI:1921340] | Paqr5 | protein_coding |
| ENSMUSG00000032281 | 198.775785 | 5.15939613 | 1.19984226 | 4.89E-07 | 0.00091814 | Up | 94180 | acyl-CoA synthetase bubblegum family member 1 [Source:MGI Symbol;Acc:MGI:2385656] | Acsbg1 | protein_coding |
| ENSMUSG00000032285 | 281.956637 | 1.79169608 | 0.54475725 | 3.47E-05 | 0.00703069 | Up | 58233 | DnaJ heat shock protein family (Hsp40) member A4 [Source:MGI Symbol;Acc:MGI:1927638] | Dnaja4 | protein_coding |
| ENSMUSG00000032420 | 179.304788 | 1.42702679 | 0.67486512 | 0.00082445 | 0.04184434 | Up | 23959 | 5' nucleotidase, ecto [Source:MGI Symbol;Acc:MGI:99782] | Nt5e | protein_coding |
| ENSMUSG00000032507 | 23.2490868 | 1.35283795 | 0.66544671 | 0.0010193 | 0.04658156 | Up | 72179 | F-box and leucine-rich repeat protein 2 [Source:MGI Symbol;Acc:MGI:1919429] | Fbxl2 | protein_coding |
| ENSMUSG00000032514 | 46.9469708 | 6.20817606 | 1.80845354 | 1.25E-05 | 0.00384937 | Up | 74052 | tetratricopeptide repeat domain 21A [Source:MGI Symbol;Acc:MGI:1921302] | Ttc21a | protein_coding |
| ENSMUSG00000032561 | 67.9251932 | 2.14507283 | 0.97292863 | 0.00061928 | 0.03623927 | Up | 56318 | acid phosphatase, prostate [Source:MGI Symbol;Acc:MGI:1928480] | Acpp | protein_coding |
| ENSMUSG00000032595 | 17.8879679 | 4.30856923 | 1.07992634 | 2.35E-06 | 0.00153749 | Up | 69398 | cadherin-related family member 4 [Source:MGI Symbol;Acc:MGI:1916648] | Cdhr4 | protein_coding |
| ENSMUSG00000032680 | 18.2205987 | 2.65722052 | 1.15810411 | 0.00049536 | 0.03187494 | Up | 228778 | RIKEN cDNA 6820408C15 gene [Source:MGI Symbol;Acc:MGI:3045333] | 6820408C15Rik | protein_coding |
| ENSMUSG00000032940 | 9.7190573 | 3.30573729 | 1.30766632 | 0.00031468 | 0.02372396 | Up | 224344 | RNA binding motif protein 11 [Source:MGI Symbol;Acc:MGI:2447622] | Rbm11 | protein_coding |
| ENSMUSG00000033161 | 3204.64177 | 0.71445111 | 0.26746006 | 0.00025925 | 0.0217699 | Up | 11928 | ATPase, Na+/K+ transporting, alpha 1 polypeptide [Source:MGI Symbol;Acc:MGI:88105] | Atp1a1 | protein_coding |
| ENSMUSG00000033502 | 122.81053 | 1.15621857 | 0.39163141 | 0.00010454 | 0.01292602 | Up | 229776 | CDC14 cell division cycle 14A [Source:MGI Symbol;Acc:MGI:2442676] | Cdc14a | protein_coding |
| ENSMUSG00000033898 | 11.5758929 | -0.0138919 | 0.06456062 | 0.00019614 | 0.01792742 | Down | 545366 | complement factor H-related 2 [Source:MGI Symbol;Acc:MGI:3611575] | Cfhr2 | protein_coding |
| ENSMUSG00000033948 | 9.21162703 | 2.84014748 | 1.08492261 | 0.00025895 | 0.0217699 | Up | 74464 | zinc finger SWIM-type containing 5 [Source:MGI Symbol;Acc:MGI:1921714] | Zswim5 | protein_coding |
| ENSMUSG00000033998 | 46.2411027 | 7.64874702 | 2.13740477 | 5.51E-06 | 0.00243824 | Up | 16525 | potassium channel, subfamily K, member 1 [Source:MGI Symbol;Acc:MGI:109322] | Kcnk1 | protein_coding |
| ENSMUSG00000034177 | 27.4609314 | 3.15877309 | 0.9351753 | 2.30E-05 | 0.00540757 | Up | 207742 | ring finger protein 43 [Source:MGI Symbol;Acc:MGI:2442609] | Rnf43 | protein_coding |
| ENSMUSG00000034227 | 133.756488 | 7.03066776 | 2.05475504 | 9.37E-06 | 0.00334496 | Up | 15223 | forkhead box J1 [Source:MGI Symbol;Acc:MGI:1347474] | Foxj1 | protein_coding |
| ENSMUSG00000034245 | 133.925378 | 0.67371477 | 0.2499726 | 0.00024583 | 0.02081765 | Up | 232232 | histone deacetylase 11 [Source:MGI Symbol;Acc:MGI:2385252] | Hdac11 | protein_coding |
| ENSMUSG00000034427 | 56.6038613 | 7.14899955 | 1.97260953 | 5.88E-06 | 0.00247622 | Up | 217328 | myosin XVB [Source:MGI Symbol;Acc:MGI:2685534] | Myo15b | protein_coding |
| ENSMUSG00000034435 | 31.6140241 | 1.77282315 | 0.64684975 | 0.0001833 | 0.01690331 | Up | 238257 | transmembrane protein 30B [Source:MGI Symbol;Acc:MGI:2442082] | Tmem30b | protein_coding |
| ENSMUSG00000034706 | 25.4545012 | 2.05723626 | 0.65065624 | 5.11E-05 | 0.00926357 | Up | 432611 | dynein axonemal intermediate chain 2 [Source:MGI Symbol;Acc:MGI:2685574] | Dnai2 | protein_coding |
| ENSMUSG00000034739 | 15.7060931 | 3.79437603 | 1.28139163 | 8.26E-05 | 0.01200954 | Up | 259172 | membrane frizzled-related protein [Source:MGI Symbol;Acc:MGI:2385957] | Mfrp | protein_coding |
| ENSMUSG00000034917 | 37.2981926 | 2.38215922 | 0.69970975 | 2.27E-05 | 0.00540757 | Up | 27375 | tight junction protein 3 [Source:MGI Symbol;Acc:MGI:1351650] | Tjp3 | protein_coding |
| ENSMUSG00000034918 | 21.4925893 | 1.84210098 | 0.83224482 | 0.00066871 | 0.03805358 | Up | 268663 | cadherin-related family member 2 [Source:MGI Symbol;Acc:MGI:2687323] | Cdhr2 | protein_coding |
| ENSMUSG00000035095 | 39.1806959 | 1.81240965 | 0.81760957 | 0.0006477 | 0.03710695 | Up | 219148 | family with sequence similarity 167, member A [Source:MGI Symbol;Acc:MGI:3606565] | Fam167a | protein_coding |
| ENSMUSG00000035112 | 47.5131252 | 1.75979879 | 0.56797176 | 5.92E-05 | 0.00963581 | Up | 69847 | WNK lysine deficient protein kinase 4 [Source:MGI Symbol;Acc:MGI:1917097] | Wnk4 | protein_coding |
| ENSMUSG00000035179 | 10.8349706 | 3.21848455 | 1.22397983 | 0.00022724 | 0.01980609 | Up | 67752 | protein phosphatase 1, regulatory subunit 32 [Source:MGI Symbol;Acc:MGI:1915002] | Ppp1r32 | protein_coding |
| ENSMUSG00000035211 | 15.1005678 | 1.68487931 | 0.6050443 | 0.00018283 | 0.01690331 | Up | 446101 | X-ray radiation resistance associated 1 [Source:MGI Symbol;Acc:MGI:2181647] | Xrra1 | protein_coding |
| ENSMUSG00000035394 | 5.47109197 | 3.71843719 | 1.39947051 | 0.00029346 | 0.02325231 | Up | 74453 | cilia and flagella associated protein 53 [Source:MGI Symbol;Acc:MGI:1921703] | Cfap53 | protein_coding |
| ENSMUSG00000035472 | 7.92164989 | 3.19961787 | 0.95523981 | 3.26E-05 | 0.00668129 | Up | 217593 | solute carrier family 25 (mitochondrial oxodicarboxylate carrier), member 21 [Source:MGI Symbol;Acc:MGI:2445059] | Slc25a21 | protein_coding |
| ENSMUSG00000035498 | 15.0056651 | 3.41394359 | 1.15985826 | 9.04E-05 | 0.01247139 | Up | 109332 | CUB domain containing protein 1 [Source:MGI Symbol;Acc:MGI:2442010] | Cdcp1 | protein_coding |
| ENSMUSG00000035578 | 34.0779959 | 1.58687957 | 0.6191846 | 0.00029847 | 0.0234432 | Up | 69707 | IQ motif containing G [Source:MGI Symbol;Acc:MGI:1916957] | Iqcg | protein_coding |
| ENSMUSG00000035769 | 95.0167514 | 1.95995556 | 0.67416246 | 0.00011005 | 0.01337183 | Up | 102448 | xylulokinase homolog (H. influenzae) [Source:MGI Symbol;Acc:MGI:2142985] | Xylb | protein_coding |
| ENSMUSG00000035783 | 4303.23063 | -1.3103251 | 0.30393796 | 7.02E-07 | 0.00111595 | Down | 11475 | actin alpha 2, smooth muscle, aorta [Source:MGI Symbol;Acc:MGI:87909] | Acta2 | protein_coding |
| ENSMUSG00000035860 | 48.1610167 | 2.56652263 | 1.31576409 | 0.00093795 | 0.04454146 | Up | 68764 | cadherin-related family member 3 [Source:MGI Symbol;Acc:MGI:1916014] | Cdhr3 | protein_coding |
| ENSMUSG00000035910 | 228.483744 | 0.00105204 | 0.06230428 | 1.03E-10 | 7.11E-07 | Up | 195208 | doublecortin domain containing 2a [Source:MGI Symbol;Acc:MGI:2652818] | Dcdc2a | protein_coding |
| ENSMUSG00000035984 | 21.5951535 | 2.43410481 | 0.8110128 | 8.24E-05 | 0.01200954 | Up | 75533 | NME/NM23 family member 5 [Source:MGI Symbol;Acc:MGI:1922783] | Nme5 | protein_coding |
| ENSMUSG00000036377 | 8.38449554 | 2.31354047 | 0.95165755 | 0.00043146 | 0.02893691 | Up | 320827 | capping protein inhibiting regulator of actin [Source:MGI Symbol;Acc:MGI:2444817] | Cracd | protein_coding |
| ENSMUSG00000036523 | 56.9432719 | 0.00259441 | 0.06237104 | 0.00104633 | 0.04739899 | Up | 268527 | gene regulated by estrogen in breast cancer protein [Source:MGI Symbol;Acc:MGI:2149712] | Greb1 | protein_coding |
| ENSMUSG00000036641 | 8.35063605 | 3.21243909 | 1.28395057 | 0.00034525 | 0.02547085 | Up | 227933 | coiled-coil domain containing 148 [Source:MGI Symbol;Acc:MGI:3039583] | Ccdc148 | protein_coding |
| ENSMUSG00000036853 | 25.108037 | 0.00244047 | 0.06236204 | 0.00087959 | 0.04345904 | Up | 171166 | mucolipin 3 [Source:MGI Symbol;Acc:MGI:1890500] | Mcoln3 | protein_coding |
| ENSMUSG00000036856 | 36.2521388 | 2.53970781 | 0.81843231 | 5.84E-05 | 0.00963581 | Up | 22417 | wingless-type MMTV integration site family, member 4 [Source:MGI Symbol;Acc:MGI:98957] | Wnt4 | protein_coding |
| ENSMUSG00000037053 | 14.8051689 | -0.0081615 | 0.0630561 | 0.00107816 | 0.04812271 | Down | 12007 | alpha-2-glycoprotein 1, zinc [Source:MGI Symbol;Acc:MGI:103163] | Azgp1 | protein_coding |
| ENSMUSG00000037143 | 10.6365015 | 5.54648243 | 1.89515654 | 9.06E-05 | 0.01247139 | Up | 78774 | cilia and flagella associated protein 61 [Source:MGI Symbol;Acc:MGI:1926024] | Cfap61 | protein_coding |

| | | | | | | | | | | |
|---|---|---|---|---|---|---|---|---|---|---|
| ENSMUSG00000037443 | 564.4431 | 0.53369501 | 0.24038664 | 0.00084542 | 0.0421834 | Up | 70012 | centrosomal protein 85 [Source:MGI Symbol;Acc:MGI:1917262] | Cep85 | protein_coding |
| ENSMUSG00000037683 | 8.97993614 | 2.52986841 | 1.18561014 | 0.00075973 | 0.04034378 | Up | 70882 | armadillo repeat containing 3 [Source:MGI Symbol;Acc:MGI:1918132] | Armc3 | protein_coding |
| ENSMUSG00000037703 | 185.875495 | 1.0261947 | 0.40322829 | 0.00032287 | 0.02425249 | Up | 241638 | leucine zipper, putative tumor suppressor family member 3 [Source:MGI Symbol;Acc:MGI:2656976] | Lzts3 | protein_coding |
| ENSMUSG00000037738 | 21.6589375 | 1.65008468 | 0.73374944 | 0.00062524 | 0.03638185 | Up | 330721 | NIMA (never in mitosis gene a)-related expressed kinase 5 [Source:MGI Symbol;Acc:MGI:2142824] | Nek5 | protein_coding |
| ENSMUSG00000037784 | 140.238385 | 0.83788365 | 0.40112924 | 0.00096167 | 0.04475433 | Up | 72507 | DAZ interacting protein 1-like [Source:MGI Symbol;Acc:MGI:1919757] | Dzip1l | protein_coding |
| ENSMUSG00000037797 | 13.9217897 | -1.1431328 | 0.49768037 | 0.00060452 | 0.03596602 | Down | 26876 | alcohol dehydrogenase 4 (class II), pi polypeptide [Source:MGI Symbol;Acc:MGI:1349472] | Adh4 | protein_coding |
| ENSMUSG00000037813 | 51.8280261 | 2.84491751 | 1.01011798 | 0.00013044 | 0.01441539 | Up | 228846 | RIKEN cDNA D630003M21 gene [Source:MGI Symbol;Acc:MGI:3606579] | D630003M21Rik | protein_coding |
| ENSMUSG00000037989 | 86.4722074 | 3.97556392 | 1.04068844 | 4.10E-06 | 0.00208608 | Up | 75607 | WNK lysine deficient protein kinase 2 [Source:MGI Symbol;Acc:MGI:1922857] | Wnk2 | protein_coding |
| ENSMUSG00000038011 | 44.3234624 | 3.59331202 | 1.28573622 | 0.00012516 | 0.01420581 | Up | 56087 | dynein, axonemal, heavy chain 10 [Source:MGI Symbol;Acc:MGI:1860299] | Dnah10 | protein_coding |
| ENSMUSG00000038060 | 71.1921575 | 1.39582163 | 0.59664955 | 0.00051646 | 0.03262568 | Up | 320256 | deleted in lung and esophageal cancer 1 [Source:MGI Symbol;Acc:MGI:2443671] | Dlec1 | protein_coding |
| ENSMUSG00000038132 | 6.8164835 | 4.16369767 | 1.19654819 | 2.99E-05 | 0.00640811 | Up | 666794 | RNA binding motif protein 24 [Source:MGI Symbol;Acc:MGI:3610364] | Rbm24 | protein_coding |
| ENSMUSG00000038370 | 55.6493327 | 3.47011011 | 0.9831298 | 1.27E-05 | 0.00387114 | Up | 66425 | Purkinje cell protein 4-like 1 [Source:MGI Symbol;Acc:MGI:1913675] | Pcp4l1 | protein_coding |
| ENSMUSG00000038541 | 38.4421388 | 0.00156524 | 0.06232038 | 0.00109099 | 0.04825815 | Up | 94224 | steroid 5 alpha-reductase 2 [Source:MGI Symbol;Acc:MGI:2150380] | Srd5a2 | protein_coding |
| ENSMUSG00000038576 | 57.1112627 | 3.30263656 | 1.07550123 | 5.84E-05 | 0.00963581 | Up | 96935 | sushi domain containing 4 [Source:MGI Symbol;Acc:MGI:2138351] | Susd4 | protein_coding |
| ENSMUSG00000038677 | 46.2916846 | 3.01882686 | 0.75413035 | 2.21E-06 | 0.00153749 | Up | 268935 | signal peptide, CUB domain, EGF-like 3 [Source:MGI Symbol;Acc:MGI:3045253] | Scube3 | protein_coding |
| ENSMUSG00000038879 | 29.4141493 | 1.75520524 | 0.54862252 | 4.68E-05 | 0.00870053 | Up | 223473 | NIPA-like domain containing 2 [Source:MGI Symbol;Acc:MGI:1924488] | Nipal2 | protein_coding |
| ENSMUSG00000039021 | 27.3269484 | 2.78109723 | 1.1003697 | 0.00028529 | 0.02284226 | Up | 338348 | tetratricopeptide repeat domain 16 [Source:MGI Symbol;Acc:MGI:2443048] | Ttc16 | protein_coding |
| ENSMUSG00000039084 | 19.3981247 | 1.95489915 | 0.87926035 | 0.00063992 | 0.03682109 | Up | 12643 | chondroadherin [Source:MGI Symbol;Acc:MGI:1096866] | Chad | protein_coding |
| ENSMUSG00000039110 | 22.3851426 | 2.60323895 | 1.15486563 | 0.00054186 | 0.03345719 | Up | 104601 | MYCBP associated protein [Source:MGI Symbol;Acc:MGI:2388726] | Mycbpap | protein_coding |
| ENSMUSG00000039533 | 23.5857648 | 0.00349355 | 0.06243618 | 0.00048899 | 0.03179384 | Up | 75104 | monocyte to macrophage differentiation-associated 2 [Source:MGI Symbol;Acc:MGI:1922354] | Mmd2 | protein_coding |
| ENSMUSG00000039543 | 23.6731791 | 2.9273279 | 1.00556241 | 0.00010137 | 0.01292602 | Up | 76670 | cilia and flagella associated protein 70 [Source:MGI Symbol;Acc:MGI:1923920] | Cfap70 | protein_coding |
| ENSMUSG00000039552 | 40.9812301 | 5.63146131 | 2.47827836 | 0.0002635 | 0.02186019 | Up | 212892 | radial spoke head 4 homolog A (Chlamydomonas) [Source:MGI Symbol;Acc:MGI:3027894] | Rsph4a | protein_coding |
| ENSMUSG00000039676 | 13.9857903 | 2.50099839 | 0.96938761 | 0.00027276 | 0.02230486 | Up | 75568 | calcyphosine-like [Source:MGI Symbol;Acc:MGI:1922818] | Capsl | protein_coding |
| ENSMUSG00000039963 | 32.4139499 | 3.71087246 | 1.22518722 | 6.36E-05 | 0.01011188 | Up | 207607 | coiled-coil domain containing 40 [Source:MGI Symbol;Acc:MGI:2443893] | Ccdc40 | protein_coding |
| ENSMUSG00000040140 | 4.97346341 | 3.82016907 | 1.54320257 | 0.00041796 | 0.02858904 | Up | 210510 | tudor domain containing 6 [Source:MGI Symbol;Acc:MGI:2679727] | Tdrd6 | protein_coding |
| ENSMUSG00000040183 | 33.6149697 | 1.21429755 | 0.56904301 | 0.00084069 | 0.04204865 | Up | 140577 | ankyrin repeat domain 6 [Source:MGI Symbol;Acc:MGI:2154278] | Ankrd6 | protein_coding |
| ENSMUSG00000040205 | 165.347886 | 0.00164806 | 0.06232358 | 7.63E-05 | 0.01136906 | Up | 16433 | CUB and zona pellucida-like domains 1 [Source:MGI Symbol;Acc:MGI:1202881] | Cuzd1 | protein_coding |
| ENSMUSG00000040350 | 62.4230943 | 2.08843049 | 0.56224605 | 7.38E-06 | 0.0028776 | Up | 94089 | tripartite motif-containing 7 [Source:MGI Symbol;Acc:MGI:2137353] | Trim7 | protein_coding |
| ENSMUSG00000040434 | 12.4399627 | 2.29808328 | 1.01168042 | 0.00058029 | 0.03494774 | Up | 228366 | LARGE xylosyl- and glucuronyltransferase 2 [Source:MGI Symbol;Acc:MGI:2443769] | Large2 | protein_coding |
| ENSMUSG00000040473 | 82.6016061 | 1.83985148 | 0.64876042 | 0.00013723 | 0.01453726 | Up | 207686 | cilia and flagella associated protein 69 [Source:MGI Symbol;Acc:MGI:2443778] | Cfap69 | protein_coding |
| ENSMUSG00000040606 | 209.743091 | 0.62924619 | 0.28234107 | 0.00076805 | 0.04049479 | Up | 71529 | kazrin, periplakin interacting protein [Source:MGI Symbol;Acc:MGI:1918779] | Kazn | protein_coding |
| ENSMUSG00000040728 | 16.1908676 | 3.22783451 | 1.18056159 | 0.000167 | 0.01597103 | Up | 207920 | epithelial splicing regulatory protein 1 [Source:MGI Symbol;Acc:MGI:1917326] | Esrp1 | protein_coding |
| ENSMUSG00000040812 | 18.2267813 | 2.00940066 | 0.71049903 | 0.0001454 | 0.01501778 | Up | 271813 | ATP/GTP binding protein-like 2 [Source:MGI Symbol;Acc:MGI:2443254] | Agbl2 | protein_coding |
| ENSMUSG00000040860 | 190.29472 | 1.58944681 | 0.4598562 | 1.95E-05 | 0.0049459 | Up | 230872 | ciliary rootlet coiled-coil, rootletin [Source:MGI Symbol;Acc:MGI:3529431] | Crocc | protein_coding |
| ENSMUSG00000040936 | 57.2497278 | 1.14091284 | 0.56042011 | 0.00101729 | 0.04658156 | Up | 209012 | unc-51-like kinase 4 [Source:MGI Symbol;Acc:MGI:1921622] | Ulk4 | protein_coding |
| ENSMUSG00000040938 | 67.5502252 | 1.1958828 | 0.59819489 | 0.00108327 | 0.04812271 | Up | 216867 | solute carrier family 16 (monocarboxylic acid transporters), member 11 [Source:MGI Symbol;Acc:MGI:2663709] | Slc16a11 | protein_coding |
| ENSMUSG00000041046 | 16.0910662 | 3.10890485 | 0.96843968 | 4.19E-05 | 0.00801702 | Up | 56089 | receptor (calcitonin) activity modifying protein 3 [Source:MGI Symbol;Acc:MGI:1860292] | Ramp3 | protein_coding |
| ENSMUSG00000041144 | 46.2979396 | 1.46643501 | 0.52851489 | 0.00017091 | 0.01619453 | Up | 227058 | dynein, axonemal, heavy chain 7B [Source:MGI Symbol;Acc:MGI:2684953] | Dnah7b | protein_coding |
| ENSMUSG00000041165 | 3.83631728 | 4.77050558 | 2.28765874 | 0.00053066 | 0.03301784 | Up | 74288 | spermatid maturation 1 [Source:MGI Symbol;Acc:MGI:1921538] | Spem1 | protein_coding |
| ENSMUSG00000041351 | 74.9783421 | 2.0022804 | 0.66836451 | 8.57E-05 | 0.0120438 | Up | 110351 | Rap1 GTPase-activating protein [Source:MGI Symbol;Acc:MGI:109338] | Rap1gap | protein_coding |
| ENSMUSG00000041673 | 26.3658019 | 1.27640537 | 0.60689866 | 0.00088782 | 0.04345904 | Up | 67580 | leucine rich repeat containing 18 [Source:MGI Symbol;Acc:MGI:1914830] | Lrrc18 | protein_coding |
| ENSMUSG00000042096 | 83.8877616 | 0.00166782 | 0.06232439 | 1.14E-15 | 2.36E-11 | Up | 13142 | D-amino acid oxidase [Source:MGI Symbol;Acc:MGI:94859] | Dao | protein_coding |
| ENSMUSG00000042195 | 5.36518424 | 2.69777499 | 1.33180973 | 0.00108749 | 0.04820651 | Up | 72022 | solute carrier family 35, member F2 [Source:MGI Symbol;Acc:MGI:1919272] | Slc35f2 | protein_coding |
| ENSMUSG00000042216 | 30.85523 | 1.8872324 | 0.49004885 | 4.17E-06 | 0.00208608 | Up | 52850 | small G protein signaling modulator 1 [Source:MGI Symbol;Acc:MGI:107320] | Sgsm1 | protein_coding |
| ENSMUSG00000042246 | 57.8565866 | 1.14451424 | 0.35092335 | 4.07E-05 | 0.00794125 | Up | 209760 | transmembrane channel-like gene family 7 [Source:MGI Symbol;Acc:MGI:2443317] | Tmc7 | protein_coding |
| ENSMUSG00000042428 | 98.9567726 | 1.32211339 | 0.41745858 | 5.27E-05 | 0.00931096 | Up | 17309 | mannoside acetylglucosaminyltransferase 3 [Source:MGI Symbol;Acc:MGI:104532] | Mgat3 | protein_coding |
| ENSMUSG00000042540 | 6.26849378 | 7.66640951 | 2.76493392 | 5.62E-06 | 0.00243824 | Up | 217698 | acyl-CoA thioesterase 5 [Source:MGI Symbol;Acc:MGI:2384969] | Acot5 | protein_coding |
| ENSMUSG00000042707 | 21.5752797 | 4.25280635 | 1.47622265 | 9.48E-05 | 0.01275873 | Up | 75563 | dynein, axonemal, light intermediate polypeptide 1 [Source:MGI Symbol;Acc:MGI:1922813] | Dnali1 | protein_coding |
| ENSMUSG00000042750 | 6.23398704 | 3.29279135 | 1.51668673 | 0.00072778 | 0.03923737 | Up | 12069 | brain expressed X-linked 2 [Source:MGI Symbol;Acc:MGI:1338017] | Bex2 | protein_coding |
| ENSMUSG00000042788 | 12.0580286 | 4.69819959 | 1.61625816 | 0.00010575 | 0.01292602 | Up | 329831 | family with sequence similarity 166, member B [Source:MGI Symbol;Acc:MGI:2445194] | Fam166b | protein_coding |

| | | | | | | | | | | |
|---|---|---|---|---|---|---|---|---|---|---|
| ENSMUSG00000043020 | 17.6210895 | 6.26949047 | 2.14899744 | 6.62E-05 | 0.01034153 | Up | 242253 | dynein axonemal intermediate chain 3 [Source:MGI Symbol;Acc:MGI:3045269] | Dnai3 | protein_coding |
| ENSMUSG00000043168 | 31.8199908 | 6.44567758 | 3.16744635 | 0.00035559 | 0.02568902 | Up | 74644 | RIKEN cDNA 4930426D05 gene [Source:MGI Symbol;Acc:MGI:1921894] | 4930426D05Rik | lncRNA |
| ENSMUSG00000043190 | 220.173041 | -0.4313494 | 0.14902623 | 0.0001546 | 0.01548545 | Down | 218341 | Rieske (Fe-S) domain containing [Source:MGI Symbol;Acc:MGI:2145198] | Rfesd | protein_coding |
| ENSMUSG00000043541 | 24.165179 | 2.72309459 | 0.86287408 | 4.97E-05 | 0.00908598 | Up | 320662 | cancer susceptibility candidate 1 [Source:MGI Symbol;Acc:MGI:2444480] | Casc1 | protein_coding |
| ENSMUSG00000043592 | 12.6471166 | 4.66232072 | 1.57359448 | 8.53E-05 | 0.0120438 | Up | 76589 | unc-5 family C-terminal like [Source:MGI Symbol;Acc:MGI:1923839] | Unc5cl | protein_coding |
| ENSMUSG00000043621 | 52.4434315 | 2.58784866 | 1.03972701 | 0.00031392 | 0.02372396 | Up | 212190 | UBX domain protein 10 [Source:MGI Symbol;Acc:MGI:2443123] | Ubxn10 | protein_coding |
| ENSMUSG00000043969 | 41.2642081 | 1.1696877 | 0.57263809 | 0.0010045 | 0.04631687 | Up | 13797 | empty spiracles homeobox 2 [Source:MGI Symbol;Acc:MGI:95388] | Emx2 | protein_coding |
| ENSMUSG00000044026 | 272.589316 | 0.77445029 | 0.32766442 | 0.00052835 | 0.0329735 | Up | 240660 | solute carrier family 35, member G1 [Source:MGI Symbol;Acc:MGI:2444789] | Slc35g1 | protein_coding |
| ENSMUSG00000044084 | 3.287452 | 4.84191458 | 2.1087846 | 0.0003552 | 0.02568902 | Up | 108803 | SPEM family member 2 [Source:MGI Symbol;Acc:MGI:1918293] | Spem2 | protein_coding |
| ENSMUSG00000044206 | 96.6382589 | 1.37024982 | 0.66548606 | 0.000959 | 0.04475433 | Up | 278180 | V-set and immunoglobulin domain containing 4 [Source:MGI Symbol;Acc:MGI:2679720] | Vsig4 | protein_coding |
| ENSMUSG00000044770 | 24.0017962 | 1.2154094 | 0.53373419 | 0.00062432 | 0.03638185 | Up | 268297 | Scm polycomb group protein like 4 [Source:MGI Symbol;Acc:MGI:2446140] | Scml4 | protein_coding |
| ENSMUSG00000044807 | 78.3859845 | 0.47413665 | 0.22317873 | 0.00103375 | 0.04693233 | Up | 30944 | zinc finger protein 354C [Source:MGI Symbol;Acc:MGI:1353621] | Zfp354c | protein_coding |
| ENSMUSG00000044948 | 163.031931 | 1.5633985 | 0.40130621 | 3.73E-06 | 0.00208608 | Up | 100048534 | cilia and flagella associated protein 43 [Source:MGI Symbol;Acc:MGI:1289258] | Cfap43 | protein_coding |
| ENSMUSG00000045275 | 55.455065 | 1.3158071 | 0.46970842 | 0.00016123 | 0.01578463 | Up | 385668 | Leber congenital amaurosis 5-like [Source:MGI Symbol;Acc:MGI:3041157] | Lca5l | protein_coding |
| ENSMUSG00000045326 | 77.749235 | 3.1637859 | 1.17472073 | 0.00017237 | 0.01623181 | Up | 320181 | fibronectin type III domain containing 7 [Source:MGI Symbol;Acc:MGI:2443535] | Fndc7 | protein_coding |
| ENSMUSG00000045394 | 83.9638137 | 5.28479478 | 1.39310237 | 3.90E-06 | 0.00208608 | Up | 17075 | epithelial cell adhesion molecule [Source:MGI Symbol;Acc:MGI:106653] | Epcam | protein_coding |
| ENSMUSG00000046160 | 5.92960879 | -0.0174947 | 0.06584326 | 0.00079203 | 0.04100509 | Down | 50914 | oligodendrocyte transcription factor 1 [Source:MGI Symbol;Acc:MGI:1355334] | Olig1 | protein_coding |
| ENSMUSG00000046192 | 10.2770583 | 0.00319004 | 0.06241213 | 0.0006059 | 0.03596602 | Up | 214704 | IQ motif and ubiquitin domain containing [Source:MGI Symbol;Acc:MGI:3041159] | Iqub | protein_coding |
| ENSMUSG00000046196 | 3.69291439 | 6.76496876 | 2.9311351 | 0.0001388 | 0.01462901 | Up | 67737 | tetratricopeptide repeat domain 39D [Source:MGI Symbol;Acc:MGI:1914987] | Ttc39d | protein_coding |
| ENSMUSG00000046432 | 80.1582283 | 0.97968532 | 0.49226558 | 0.00113427 | 0.04922381 | Up | 12070 | brain expressed X-linked 3 [Source:MGI Symbol;Acc:MGI:1338016] | Bex3 | protein_coding |
| ENSMUSG00000046807 | 30.5734514 | 3.57625597 | 1.03374312 | 1.67E-05 | 0.00454897 | Up | 192734 | leucine rich repeat containing 75B [Source:MGI Symbol;Acc:MGI:2143657] | Lrrc75b | protein_coding |
| ENSMUSG00000046818 | 44.44662 | 2.28749951 | 0.6745653 | 2.36E-05 | 0.00542574 | Up | 73284 | DNA-damage-inducible transcript 4-like [Source:MGI Symbol;Acc:MGI:1920534] | Ddit4l | protein_coding |
| ENSMUSG00000047021 | 89.4208203 | 0.00112763 | 0.06230626 | 1.77E-10 | 9.16E-07 | Up | 241116 | cilia and flagella associated protein 65 [Source:MGI Symbol;Acc:MGI:2444274] | Cfap65 | protein_coding |
| ENSMUSG00000047040 | 21.8991294 | 3.75009555 | 1.22356072 | 5.97E-05 | 0.00963581 | Up | 217138 | proline rich 15-like [Source:MGI Symbol;Acc:MGI:2387599] | Prr15l | protein_coding |
| ENSMUSG00000047139 | 386.217116 | 1.11322401 | 0.46917017 | 0.00049252 | 0.03179384 | Up | 12484 | CD24a antigen [Source:MGI Symbol;Acc:MGI:88323] | Cd24a | protein_coding |
| ENSMUSG00000047182 | 1415.25922 | -0.4463792 | 0.16746756 | 0.00032565 | 0.02436494 | Down | 16369 | insulin receptor substrate 3 [Source:MGI Symbol;Acc:MGI:1194882] | Irs3 | protein_coding |
| ENSMUSG00000047361 | 17.2248052 | 5.41692506 | 1.31654371 | 1.57E-06 | 0.00153749 | Up | 381260 | predicted gene 973 [Source:MGI Symbol;Acc:MGI:2685819] | Gm973 | protein_coding |
| ENSMUSG00000047394 | 32.9588269 | 2.27772502 | 0.95735851 | 0.00042955 | 0.02893691 | Up | 70113 | outer dense fiber of sperm tails 3B [Source:MGI Symbol;Acc:MGI:1917363] | Odf3b | protein_coding |
| ENSMUSG00000047502 | 6.45030442 | 1.94648075 | 0.93555506 | 0.00098406 | 0.04557804 | Up | 381538 | maestro heat-like repeat family member 7 [Source:MGI Symbol;Acc:MGI:2685873] | Mroh7 | protein_coding |
| ENSMUSG00000047631 | 8.94310927 | -0.0081501 | 0.06306324 | 0.00102152 | 0.04658156 | Down | 103161 | apolipoprotein F [Source:MGI Symbol;Acc:MGI:104539] | Apof | protein_coding |
| ENSMUSG00000047671 | 23.3366817 | 2.1289707 | 1.08244338 | 0.00103249 | 0.04693233 | Up | 242646 | dynein light chain Tctex-type 4 [Source:MGI Symbol;Acc:MGI:3045358] | Dynlt4 | protein_coding |
| ENSMUSG00000048029 | 17.489886 | 2.91771944 | 1.25295648 | 0.00044587 | 0.02971049 | Up | 226265 | enolase 4 [Source:MGI Symbol;Acc:MGI:2441717] | Eno4 | protein_coding |
| ENSMUSG00000048038 | 80.8811195 | 5.20540892 | 1.1720751 | 2.72E-07 | 0.00080161 | Up | 329366 | coiled-coil domain containing 187 [Source:MGI Symbol;Acc:MGI:3045295] | Ccdc187 | protein_coding |
| ENSMUSG00000048416 | 24.5165969 | 2.27143313 | 0.89150956 | 0.00028866 | 0.02302258 | Up | 17349 | myeloid leukemia factor 1 [Source:MGI Symbol;Acc:MGI:1341819] | Mlf1 | protein_coding |
| ENSMUSG00000048644 | 41.3295428 | 1.18992156 | 0.48162395 | 0.00038786 | 0.02734478 | Up | 330695 | cortexin 1 [Source:MGI Symbol;Acc:MGI:88566] | Ctxn1 | protein_coding |
| ENSMUSG00000049134 | 8.45433479 | 1.89084787 | 0.87984464 | 0.00080741 | 0.04153085 | Up | 18175 | nebulin-related anchoring protein [Source:MGI Symbol;Acc:MGI:1098765] | Nrap | protein_coding |
| ENSMUSG00000049362 | 2.72148842 | 0.01369003 | 0.06443494 | 0.00068219 | 0.03839793 | Up | 259002 | olfactory receptor 173 [Source:MGI Symbol;Acc:MGI:3030007] | Olfr173 | protein_coding |
| ENSMUSG00000049493 | 20.1355527 | 3.7592284 | 1.8072963 | 0.00059478 | 0.03555063 | Up | 102502 | plastin 1 (I-isoform) [Source:MGI Symbol;Acc:MGI:104809] | Pls1 | protein_coding |
| ENSMUSG00000049571 | 36.281874 | 3.77036156 | 0.94057175 | 2.06E-06 | 0.00153749 | Up | 212124 | cilia and flagella associated protein 46 [Source:MGI Symbol;Acc:MGI:2444387] | Cfap46 | protein_coding |
| ENSMUSG00000049721 | 3.3455329 | 4.48200605 | 2.28840815 | 0.00081255 | 0.04154684 | Up | 53897 | galactose-3-O-sulfotransferase 1 [Source:MGI Symbol;Acc:MGI:1858277] | Gal3st1 | protein_coding |
| ENSMUSG00000050052 | 1757.45011 | -0.3957829 | 0.17251873 | 0.00074637 | 0.03994223 | Down | 72148 | testis development related protein [Source:MGI Symbol;Acc:MGI:1919398] | Tdrp | protein_coding |
| ENSMUSG00000050071 | 8.24071965 | 3.87987211 | 1.85293184 | 0.0007313 | 0.03923737 | Up | 19716 | brain expressed X-linked 1 [Source:MGI Symbol;Acc:MGI:1328321] | Bex1 | protein_coding |
| ENSMUSG00000050089 | 9.71409857 | 0.00073601 | 0.06229748 | 1.09E-05 | 0.00361938 | Up | 11643 | A kinase (PRKA) anchor protein 4 [Source:MGI Symbol;Acc:MGI:102794] | Akap4 | protein_coding |
| ENSMUSG00000050122 | 12.2294474 | 2.83536446 | 1.12924896 | 0.00031351 | 0.02372396 | Up | 70853 | von Willebrand factor A domain containing 3B [Source:MGI Symbol;Acc:MGI:1918103] | Vwa3b | protein_coding |
| ENSMUSG00000050640 | 24.758491 | 3.88957574 | 0.95048335 | 1.54E-06 | 0.00153749 | Up | 231503 | transmembrane protein 150C [Source:MGI Symbol;Acc:MGI:3041258] | Tmem150c | protein_coding |
| ENSMUSG00000050914 | 13.0321522 | 1.58598191 | 0.66300583 | 0.00049019 | 0.03179384 | Up | 654824 | ankyrin repeat domain 37 [Source:MGI Symbol;Acc:MGI:3603344] | Ankrd37 | protein_coding |
| ENSMUSG00000050953 | 1418.65423 | 1.33994562 | 0.37134352 | 1.14E-05 | 0.00368076 | Up | 14609 | gap junction protein, alpha 1 [Source:MGI Symbol;Acc:MGI:95713] | Gja1 | protein_coding |
| ENSMUSG00000051497 | 36.0905664 | 3.60927178 | 1.74085287 | 0.00060772 | 0.03597054 | Up | 16517 | potassium inwardly-rectifying channel, subfamily J, member 16 [Source:MGI Symbol;Acc:MGI:1314842] | Kcnj16 | protein_coding |
| ENSMUSG00000052221 | 18.3413861 | 2.98587468 | 1.46522019 | 0.00077272 | 0.04051295 | Up | 210762 | protein phosphatase 1, regulatory subunit 36 [Source:MGI Symbol;Acc:MGI:2684916] | Ppp1r36 | protein_coding |
| ENSMUSG00000052387 | 27.4787457 | 1.75421798 | 0.59439964 | 0.00010316 | 0.01292602 | Up | 226025 | transient receptor potential cation channel, subfamily M, member 3 [Source:MGI Symbol;Acc:MGI:2443101] | Trpm3 | protein_coding |
| ENSMUSG00000052512 | 94.4183826 | 2.04807104 | 0.79380964 | 0.00026674 | 0.0219523 | Up | 78286 | neuron navigator 2 [Source:MGI Symbol;Acc:MGI:2183691] | Nav2 | protein_coding |
| ENSMUSG00000052566 | 123.651862 | 0.77970324 | 0.33838899 | 0.00059546 | 0.03555063 | Up | 170833 | hook microtubule tethering protein 2 [Source:MGI Symbol;Acc:MGI:2181664] | Hook2 | protein_coding |

| | | | | | | | | | | |
|---|---|---|---|---|---|---|---|---|---|---|
| ENSMUSG00000052861 | 60.226633 | 4.41969783 | 1.04579434 | 7.82E-07 | 0.0011532 | Up | 330355 | dynein, axonemal, heavy chain 6 [Source:MGI Symbol;Acc:MGI:107744] | Dnah6 | protein_coding |
| ENSMUSG00000053111 | 14.6988358 | 3.00705629 | 1.06168211 | 0.00013073 | 0.01441539 | Up | 66930 | fibronectin type 3 and ankyrin repeat domains 1 [Source:MGI Symbol;Acc:MGI:1914180] | Fank1 | protein_coding |
| ENSMUSG00000053182 | 23.1609871 | 5.09747849 | 1.58547234 | 3.27E-05 | 0.00668129 | Up | 208166 | predicted gene 609 [Source:MGI Symbol;Acc:MGI:2685455] | Gm609 | protein_coding |
| ENSMUSG00000053414 | 40.1274755 | 3.22101518 | 0.81487686 | 2.72E-06 | 0.00165123 | Up | 26559 | hormonally upregulated Neu-associated kinase [Source:MGI Symbol;Acc:MGI:1347352] | Hunk | protein_coding |
| ENSMUSG00000053552 | 18.2305891 | 1.01163444 | 0.46499226 | 0.00083701 | 0.04196648 | Up | 228598 | early B cell factor 4 [Source:MGI Symbol;Acc:MGI:2385972] | Ebf4 | protein_coding |
| ENSMUSG00000054065 | 47.2916458 | 1.03040203 | 0.45077758 | 0.00061614 | 0.03615809 | Up | 56460 | plakophilin 3 [Source:MGI Symbol;Acc:MGI:1891830] | Pkp3 | protein_coding |
| ENSMUSG00000054252 | 132.054051 | 1.14888058 | 0.51957699 | 0.00070122 | 0.03868938 | Up | 14184 | fibroblast growth factor receptor 3 [Source:MGI Symbol;Acc:MGI:95524] | Fgfr3 | protein_coding |
| ENSMUSG00000054320 | 71.8945494 | 1.08388456 | 0.50054387 | 0.00078744 | 0.04086971 | Up | 270091 | leucine rich repeat containing 36 [Source:MGI Symbol;Acc:MGI:2448585] | Lrrc36 | protein_coding |
| ENSMUSG00000054889 | 123.142998 | 2.78875781 | 0.98334437 | 0.00012333 | 0.01417611 | Up | 109620 | desmoplakin [Source:MGI Symbol;Acc:MGI:109611] | Dsp | protein_coding |
| ENSMUSG00000054942 | 160.348207 | 0.59085656 | 0.25996308 | 0.0006971 | 0.03860567 | Up | 215708 | mitoguardin 1 [Source:MGI Symbol;Acc:MGI:1924567] | Miga1 | protein_coding |
| ENSMUSG00000055629 | 11.6226614 | 2.1168111 | 1.04525951 | 0.00096281 | 0.04475433 | Up | 330671 | beta-1,4-N-acetyl-galactosaminyl transferase 4 [Source:MGI Symbol;Acc:MGI:2652891] | B4galnt4 | protein_coding |
| ENSMUSG00000055809 | 39.1070931 | 1.19292209 | 0.57054126 | 0.0009174 | 0.04407147 | Up | 436022 | dynein, axonemal assembly factor 3 [Source:MGI Symbol;Acc:MGI:3588207] | Dnaaf3 | protein_coding |
| ENSMUSG00000055945 | 12.527845 | 3.9277452 | 1.40800911 | 0.00014076 | 0.01476014 | Up | 320111 | proline rich 18 [Source:MGI Symbol;Acc:MGI:2443403] | Prr18 | protein_coding |
| ENSMUSG00000056162 | 59.1169867 | 1.0818166 | 0.52222905 | 0.00096411 | 0.04475433 | Up | 338403 | carnosine dipeptidase 1 (metallopeptidase M20 family) [Source:MGI Symbol;Acc:MGI:2451097] | Cndp1 | protein_coding |
| ENSMUSG00000056174 | 85.0923461 | 1.10030318 | 0.51222775 | 0.0008188 | 0.0416602 | Up | 329941 | collagen, type VIII, alpha 2 [Source:MGI Symbol;Acc:MGI:88464] | Col8a2 | protein_coding |
| ENSMUSG00000056215 | 15.3425637 | 2.8793833 | 1.37096568 | 0.00071115 | 0.03874458 | Up | 74354 | leucine-rich repeats and guanylate kinase domain containing [Source:MGI Symbol;Acc:MGI:1921604] | Lrguk | protein_coding |
| ENSMUSG00000056598 | 44.436947 | 2.07855901 | 0.66302778 | 5.55E-05 | 0.00963581 | Up | 74665 | dynein regulatory complex subunit 3 [Source:MGI Symbol;Acc:MGI:1921915] | Drc3 | protein_coding |
| ENSMUSG00000056752 | 45.4026279 | 6.24336904 | 1.68487496 | 5.03E-06 | 0.00236347 | Up | 237806 | dynein, axonemal, heavy chain 9 [Source:MGI Symbol;Acc:MGI:1289279] | Dnah9 | protein_coding |
| ENSMUSG00000057068 | 21.7221945 | 2.18239331 | 0.85581901 | 0.00029379 | 0.02325231 | Up | 384198 | family with sequence similarity 47, member E [Source:MGI Symbol;Acc:MGI:2686227] | Fam47e | protein_coding |
| ENSMUSG00000057130 | 369.55447 | -0.4349414 | 0.18969227 | 0.00067957 | 0.03835472 | Down | 27366 | thioredoxin-like 4A [Source:MGI Symbol;Acc:MGI:1351613] | Txnl4a | protein_coding |
| ENSMUSG00000057465 | 12.2918985 | -0.003782 | 0.06246316 | 9.21E-05 | 0.01259834 | Down | 20209 | serum amyloid A 2 [Source:MGI Symbol;Acc:MGI:98222] | Saa2 | protein_coding |
| ENSMUSG00000057897 | 50.5326038 | 2.01978976 | 0.53539956 | 5.99E-06 | 0.00247622 | Up | 12323 | calcium/calmodulin-dependent protein kinase II, beta [Source:MGI Symbol;Acc:MGI:88257] | Camk2b | protein_coding |
| ENSMUSG00000058488 | 26.4211434 | 0.00222609 | 0.06235044 | 0.00024166 | 0.02071354 | Up | 16591 | klotho [Source:MGI Symbol;Acc:MGI:1101771] | Kl | protein_coding |
| ENSMUSG00000058620 | 8.23036085 | 2.85139873 | 1.28481845 | 0.0006729 | 0.03818719 | Up | 11552 | adrenergic receptor, alpha 2b [Source:MGI Symbol;Acc:MGI:87935] | Adra2b | protein_coding |
| ENSMUSG00000058799 | 7077.22223 | -0.3630396 | 0.16748487 | 0.00106524 | 0.04800937 | Down | 53605 | nucleosome assembly protein 1-like 1 [Source:MGI Symbol;Acc:MGI:1855693] | Nap1l1 | protein_coding |
| ENSMUSG00000059891 | 13.4483208 | 1.49017641 | 0.74380427 | 0.00111332 | 0.04862142 | Up | 22116 | testis-specific serine kinase substrate [Source:MGI Symbol;Acc:MGI:1347560] | Tsks | protein_coding |
| ENSMUSG00000060176 | 31.1564517 | 2.03627507 | 0.62108036 | 3.53E-05 | 0.00708415 | Up | 75050 | kinesin family member 27 [Source:MGI Symbol;Acc:MGI:1922300] | Kif27 | protein_coding |
| ENSMUSG00000060512 | 15.8337576 | 2.22764802 | 0.79599657 | 0.00015259 | 0.01545128 | Up | 76261 | RIKEN cDNA 0610040J01 gene [Source:MGI Symbol;Acc:MGI:1923511] | 0610040J01Rik | protein_coding |
| ENSMUSG00000060613 | 13.9724958 | -0.0091401 | 0.06327362 | 0.0002459 | 0.02081765 | Down | 226105 | cytochrome P450, family 2, subfamily c, polypeptide 70 [Source:MGI Symbol;Acc:MGI:2385878] | Cyp2c70 | protein_coding |
| ENSMUSG00000060680 | 7.68137656 | -0.0154623 | 0.06513921 | 3.23E-05 | 0.00668129 | Down | NA | predicted gene 8894 [Source:MGI Symbol;Acc:MGI:3643084] | Gm8894 | processed_pseudo gene |
| ENSMUSG00000060716 | 41.5965 | 3.13699553 | 0.84721089 | 7.09E-06 | 0.00281726 | Up | 211945 | pleckstrin homology domain containing, family H (with MyTH4 domain) member 1 [Source:MGI Symbol;Acc:MGI:2144989] | Plekhh1 | protein_coding |
| ENSMUSG00000061048 | 126.602291 | 0.86678227 | 0.31816993 | 0.00021822 | 0.01926384 | Up | 12560 | cadherin 3 [Source:MGI Symbol;Acc:MGI:88356] | Cdh3 | protein_coding |
| ENSMUSG00000061718 | 76.5916769 | 5.62850967 | 1.30434006 | 4.68E-07 | 0.00091814 | Up | 19049 | protein phosphatase 1, regulatory inhibitor subunit 1B [Source:MGI Symbol;Acc:MGI:94860] | Ppp1r1b | protein_coding |
| ENSMUSG00000061859 | 156.160761 | 0.91594778 | 0.41288183 | 0.00071007 | 0.03874458 | Up | 12695 | PATJ, crumbs cell polarity complex component [Source:MGI Symbol;Acc:MGI:1277960] | Patj | protein_coding |
| ENSMUSG00000062296 | 49.848263 | 5.58562397 | 1.43730043 | 2.62E-06 | 0.00164246 | Up | 320429 | tetratricopeptide repeat and ankyrin repeat containing 1 [Source:MGI Symbol;Acc:MGI:1341834] | Trank1 | protein_coding |
| ENSMUSG00000062859 | 19.8428974 | 4.09176605 | 1.08688928 | 5.67E-06 | 0.00243824 | Up | 21463 | t-complex protein 11 [Source:MGI Symbol;Acc:MGI:98544] | Tcp11 | protein_coding |
| ENSMUSG00000063130 | 77.4206938 | 7.51428446 | 2.83419691 | 7.43E-05 | 0.01120492 | Up | 70405 | calmodulin-like 3 [Source:MGI Symbol;Acc:MGI:1917655] | Calml3 | protein_coding |
| ENSMUSG00000063529 | 5.44070113 | 3.01959677 | 1.02449908 | 0.00012843 | 0.01440481 | Up | 380842 | stathmin domain containing 1 [Source:MGI Symbol;Acc:MGI:2686420] | Stmnd1 | protein_coding |
| ENSMUSG00000063704 | 59.3448092 | 4.06123964 | 1.74149522 | 0.00032672 | 0.02436494 | Up | 332110 | mitogen-activated protein kinase 15 [Source:MGI Symbol;Acc:MGI:2652894] | Mapk15 | protein_coding |
| ENSMUSG00000064280 | 12.7086863 | 4.72153934 | 1.60536029 | 9.62E-05 | 0.01275873 | Up | 75172 | coiled-coil domain containing 146 [Source:MGI Symbol;Acc:MGI:1922422] | Ccdc146 | protein_coding |
| ENSMUSG00000064339 | 6298.85201 | -0.835818 | 0.36160145 | 0.00057612 | 0.03479774 | Down | NA | mitochondrially encoded 16S rRNA [Source:MGI Symbol;Acc:MGI:102492] | mt-Rnr2 | Mt_rRNA |
| ENSMUSG00000064350 | 120.245127 | -1.4534969 | 0.4655186 | 5.77E-05 | 0.00963581 | Down | NA | mitochondrially encoded tRNA tyrosine [Source:MGI Symbol;Acc:MGI:102470] | mt-Ty | Mt_tRNA |
| ENSMUSG00000066154 | 121.989391 | -0.0084387 | 0.06312331 | 0.00061152 | 0.03601664 | Down | 17842 | major urinary protein 3 [Source:MGI Symbol;Acc:MGI:97235] | Mup3 | protein_coding |
| ENSMUSG00000066196 | 13.3686768 | 3.67839276 | 0.99120122 | 7.72E-06 | 0.00290098 | Up | 433700 | sperm associated antigen 8 [Source:MGI Symbol;Acc:MGI:3056295] | Spag8 | protein_coding |
| ENSMUSG00000066366 | 147.245919 | -0.0433315 | 0.08689896 | 0.00020421 | 0.01842044 | Down | 20700 | serine (or cysteine) peptidase inhibitor, clade A, member 1A [Source:MGI Symbol;Acc:MGI:891971] | Serpina1a | protein_coding |
| ENSMUSG00000067158 | 194.888875 | 1.17080818 | 0.52803428 | 0.00069247 | 0.03860567 | Up | 12829 | collagen, type IV, alpha 4 [Source:MGI Symbol;Acc:MGI:104687] | Col4a4 | protein_coding |
| ENSMUSG00000067235 | 214.909326 | -1.0840779 | 0.53594288 | 0.00101308 | 0.0465049 | Down | 15007 | histocompatibility 2, Q region locus 10 [Source:MGI Symbol;Acc:MGI:95929] | H2-Q10 | protein_coding |
| ENSMUSG00000068428 | 12.659953 | 4.23285266 | 1.49019998 | 0.00011728 | 0.01384407 | Up | 239789 | geminin coiled-coil domain containing [Source:MGI Symbol;Acc:MGI:2685452] | Gmnc | protein_coding |
| ENSMUSG00000068587 | 25.7321563 | 2.07650369 | 0.73080038 | 0.00013618 | 0.01450975 | Up | 232714 | maltase-glucoamylase [Source:MGI Symbol;Acc:MGI:1203495] | Mgam | protein_coding |
| ENSMUSG00000069601 | 52.2501634 | 3.13031264 | 0.81341609 | 4.07E-06 | 0.00208608 | Up | 11735 | ankyrin 3, epithelial [Source:MGI Symbol;Acc:MGI:88026] | Ank3 | protein_coding |
| ENSMUSG00000070306 | 65.6449426 | 0.00129339 | 0.06231105 | 2.65E-12 | 2.74E-08 | Up | 270150 | coiled-coil domain containing 153 [Source:MGI Symbol;Acc:MGI:2448587] | Ccdc153 | protein_coding |
| ENSMUSG00000070473 | 58.6037394 | 1.23027298 | 0.42425215 | 0.00012002 | 0.0140577 | Up | 12739 | claudin 3 [Source:MGI Symbol;Acc:MGI:1329044] | Cldn3 | protein_coding |
| ENSMUSG00000070498 | 48.9916914 | 3.54078703 | 2.11857011 | 0.00107373 | 0.04811308 | Up | 208151 | transmembrane protein 132B [Source:MGI Symbol;Acc:MGI:3609245] | Tmem132b | protein_coding |
| ENSMUSG00000070803 | 32.2772252 | 3.51439155 | 0.87959709 | 2.25E-06 | 0.00153749 | Up | 56222 | Cbp/p300-interacting transactivator, with Glu/Asp-rich carboxy-terminal domain, 4 [Source:MGI Symbol;Acc:MGI:1861694] | Cited4 | protein_coding |
| ENSMUSG00000070880 | 1.86365229 | 3.70741611 | 1.94598289 | 0.00092793 | 0.04438826 | Up | 14415 | glutamate decarboxylase 1 [Source:MGI Symbol;Acc:MGI:95632] | Gad1 | protein_coding |

| | | | | | | | | | | |
|---|---|---|---|---|---|---|---|---|---|---|
| ENSMUSG00000071177 | 68.620777 | -0.0125731 | 0.06411151 | 0.00088323 | 0.04345904 | Down | 20703 | serine (or cysteine) peptidase inhibitor, clade A, member 1D [Source:MGI Symbol;Acc:MGI:891968] | Serpina1d | protein_coding |
| ENSMUSG00000071178 | 99.9370677 | -0.0184949 | 0.06642857 | 1.88E-06 | 0.00153749 | Down | 20701 | serine (or cysteine) peptidase inhibitor, clade A, member 1B [Source:MGI Symbol;Acc:MGI:891970] | Serpina1b | protein_coding |
| ENSMUSG00000071398 | 27.4919372 | 4.01340575 | 1.03879129 | 3.74E-06 | 0.00208608 | Up | 73667 | RIKEN cDNA 2410004P03 gene [Source:MGI Symbol;Acc:MGI:1920917] | 2410004P03Rik | protein_coding |
| ENSMUSG00000071550 | 80.507257 | 6.37069535 | 1.86167552 | 1.12E-05 | 0.00368047 | Up | 212517 | cilia and flagella associated protein 44 [Source:MGI Symbol;Acc:MGI:1277238] | Cfap44 | protein_coding |
| ENSMUSG00000071551 | 18.3333109 | 2.6126398 | 1.10078538 | 0.00042434 | 0.02873997 | Up | 432720 | aldo-keto reductase family 1, member C19 [Source:MGI Symbol;Acc:MGI:2653678] | Akr1c19 | protein_coding |
| ENSMUSG00000072663 | 34.551423 | 2.05661898 | 0.84785637 | 0.00039473 | 0.02764026 | Up | 320277 | sperm flagellar 2 [Source:MGI Symbol;Acc:MGI:2443727] | Spef2 | protein_coding |
| ENSMUSG00000072849 | 119.6799 | -0.0075728 | 0.06296104 | 0.00076698 | 0.04049479 | Down | 20704 | serine (or cysteine) peptidase inhibitor, clade A, member 1E [Source:MGI Symbol;Acc:MGI:891967] | Serpina1e | protein_coding |
| ENSMUSG00000072949 | 51.840491 | 2.09275198 | 0.54517011 | 4.58E-06 | 0.00219838 | Up | 26897 | acyl-CoA thioesterase 1 [Source:MGI Symbol;Acc:MGI:1349396] | Acot1 | protein_coding |
| ENSMUSG00000072966 | 28.3820628 | 1.76424311 | 0.6617335 | 0.00022685 | 0.01980609 | Up | 245607 | G protein-coupled receptor associated sorting protein 2 [Source:MGI Symbol;Acc:MGI:2442071] | Gprasp2 | protein_coding |
| ENSMUSG00000073077 | 13.4172113 | 2.06313263 | 0.69396173 | 9.87E-05 | 0.01275873 | Up | 636104 | cilia and flagella associated protein 47 [Source:MGI Symbol;Acc:MGI:3781475] | Cfap47 | protein_coding |
| ENSMUSG00000073600 | 64.5530052 | 2.0433944 | 0.59135434 | 1.91E-05 | 0.0049459 | Up | 381148 | proline rich basic protein 1 [Source:MGI Symbol;Acc:MGI:2686460] | Prob1 | protein_coding |
| ENSMUSG00000073650 | 28.0471946 | 2.75421993 | 0.81363683 | 2.34E-05 | 0.00542574 | Up | 241112 | ciliogenesis associated TTC17 interacting protein [Source:MGI Symbol;Acc:MGI:2685062] | Catip | protein_coding |
| ENSMUSG00000074259 | 49.0193812 | 4.35727184 | 1.00396655 | 4.84E-07 | 0.00091814 | Up | 546134 | GRAM domain containing 2 [Source:MGI Symbol;Acc:MGI:3528937] | Gramd2 | protein_coding |
| ENSMUSG00000074715 | 77.8028735 | 4.62262221 | 1.3230829 | 1.21E-05 | 0.0038445 | Up | 56838 | chemokine (C-C motif) ligand 28 [Source:MGI Symbol;Acc:MGI:1861731] | Ccl28 | protein_coding |
| ENSMUSG00000074738 | 135.592414 | 0.73664885 | 0.34508866 | 0.0009126 | 0.04407147 | Up | 230991 | fibronectin type III domain containing 10 [Source:MGI Symbol;Acc:MGI:2444790] | Fndc10 | protein_coding |
| ENSMUSG00000075028 | 70.7441158 | 0.86407203 | 0.31184864 | 0.00018512 | 0.01699536 | Up | 100042784 | PR domain containing 11 [Source:MGI Symbol;Acc:MGI:2685553] | Prdm11 | protein_coding |
| ENSMUSG00000075225 | 52.773083 | 1.96584153 | 0.82997094 | 0.00045308 | 0.02990199 | Up | 75973 | coiled-coil domain containing 162 [Source:MGI Symbol;Acc:MGI:1923223] | Ccdc162 | protein_coding |
| ENSMUSG00000075304 | 9.5982885 | 2.93617233 | 1.11888956 | 0.00024432 | 0.02081765 | Up | 64406 | trans-acting transcription factor 5 [Source:MGI Symbol;Acc:MGI:1927715] | Sp5 | protein_coding |
| ENSMUSG00000075569 | 25.4991431 | 2.84769516 | 0.73370961 | 3.83E-06 | 0.00208608 | Up | 18861 | radial spoke head 10 homolog B (Chlamydomonas) [Source:MGI Symbol;Acc:MGI:1922386] | Rsph10b | protein_coding |
| ENSMUSG00000076540 | 9.8160328 | 0.00081832 | 0.06229901 | 5.66E-06 | 0.00243824 | Up | NA | immunoglobulin kappa variable 4-80 [Source:MGI Symbol;Acc:MGI:4439653] | Igkv4-80 | IG_V_gene |
| ENSMUSG00000078161 | 22.9699332 | 4.55816317 | 1.89907371 | 0.00028178 | 0.02264881 | Up | 209601 | glutamate rich 3 [Source:MGI Symbol;Acc:MGI:1919095] | Erich3 | protein_coding |
| ENSMUSG00000078439 | 47.9751691 | 1.23711234 | 0.58527048 | 0.0008676 | 0.04297841 | Up | 72273 | small integral membrane protein 24 [Source:MGI Symbol;Acc:MGI:1919523] | Smim24 | protein_coding |
| ENSMUSG00000078451 | 33.2481117 | 2.08100274 | 0.86998731 | 0.0004241 | 0.02873997 | Up | 73075 | peptidylprolyl isomerase (cyclophilin)-like 6 [Source:MGI Symbol;Acc:MGI:1920325] | Ppil6 | protein_coding |
| ENSMUSG00000078490 | 43.3828706 | 2.76007299 | 0.68663911 | 2.15E-06 | 0.00153749 | Up | 544678 | cilia and flagella associated protein 74 [Source:MGI Symbol;Acc:MGI:1917130] | Cfap74 | protein_coding |
| ENSMUSG00000078706 | 22.4512506 | 6.06004373 | 1.99732872 | 5.75E-05 | 0.00963581 | Up | NA | predicted gene 53 [Source:MGI Symbol;Acc:MGI:2684899] | Gm53 | lncRNA |
| ENSMUSG00000079015 | 30.8301035 | -0.0150637 | 0.06497296 | 0.00011611 | 0.01378447 | Down | 20702 | serine (or cysteine) peptidase inhibitor, clade A, member 1C [Source:MGI Symbol;Acc:MGI:891969] | Serpina1c | protein_coding |
| ENSMUSG00000079056 | 136.040678 | 1.0191016 | 0.28376075 | 1.24E-05 | 0.00384937 | Up | 56461 | Kv channel interacting protein 3, calsenilin [Source:MGI Symbol;Acc:MGI:1929258] | Kcnip3 | protein_coding |
| ENSMUSG00000079139 | 415.830734 | -0.4505873 | 0.17978565 | 0.00043006 | 0.02893691 | Down | NA | predicted gene 4204 [Source:MGI Symbol;Acc:MGI:3782381] | Gm4204 | processed_pseudo gene |
| ENSMUSG00000079235 | 7.61536131 | 3.84191987 | 1.71530597 | 0.00057306 | 0.03471466 | Up | 100502861 | coiled-coil domain containing 13 [Source:MGI Symbol;Acc:MGI:1920144] | Ccdc13 | protein_coding |
| ENSMUSG00000079277 | 7.90191885 | 1.94203982 | 0.8501639 | 0.00064848 | 0.03710695 | Up | 15434 | homeobox D3 [Source:MGI Symbol;Acc:MGI:96207] | Hoxd3 | protein_coding |
| ENSMUSG00000079343 | 6.79021256 | 1.65260707 | 0.73984013 | 0.00075953 | 0.04034378 | Up | 317677 | complement component 1, s subcomponent 2 [Source:MGI Symbol;Acc:MGI:3644269] | C1s2 | protein_coding |
| ENSMUSG00000079465 | 65.1530632 | 1.64725877 | 0.57703469 | 0.00013321 | 0.01446075 | Up | 12828 | collagen, type IV, alpha 3 [Source:MGI Symbol;Acc:MGI:104688] | Col4a3 | protein_coding |
| ENSMUSG00000084989 | 32.6613257 | 6.01492056 | 1.66960134 | 8.37E-06 | 0.00308797 | Up | 381284 | ciliary rootlet coiled-coil, rootletin family member 2 [Source:MGI Symbol;Acc:MGI:3045962] | Crocc2 | protein_coding |
| ENSMUSG00000085336 | 5.04789448 | 2.10256582 | 0.97959151 | 0.00093005 | 0.04438826 | Up | 102637448 | predicted gene 11732 [Source:MGI Symbol;Acc:MGI:3652087] | Gm11732 | lncRNA |
| ENSMUSG00000085852 | 7.91280404 | 1.75835205 | 0.8168329 | 0.00091735 | 0.04407147 | Up | 639658 | predicted gene 13807 [Source:MGI Symbol;Acc:MGI:3650023] | Gm13807 | lncRNA |
| ENSMUSG00000086075 | 5.36177665 | 2.23902326 | 0.91439676 | 0.00051979 | 0.03266829 | Up | NA | predicted gene 15728 [Source:MGI Symbol;Acc:MGI:3783171] | Gm15728 | lncRNA |
| ENSMUSG00000086245 | 11.7481843 | 1.9656154 | 0.9885204 | 0.00106677 | 0.04800937 | Up | NA | predicted gene 16170 [Source:MGI Symbol;Acc:MGI:3805548] | Gm16170 | lncRNA |
| ENSMUSG00000086904 | 2.65995234 | 6.17729508 | 2.76327598 | 0.00015182 | 0.01544874 | Up | NA | predicted gene 13404 [Source:MGI Symbol;Acc:MGI:3649379] | Gm13404 | lncRNA |
| ENSMUSG00000087095 | 24.0087616 | 0.967309 | 0.47306593 | 0.00107278 | 0.04811308 | Up | 329078 | Emx2 opposite strand/antisense transcript (non-protein coding) [Source:MGI Symbol;Acc:MGI:3052329] | Emx2os | lncRNA |
| ENSMUSG00000087690 | 10.9720038 | 0.02895818 | 0.07242855 | 0.0003128 | 0.02372396 | Up | NA | predicted gene 16031 [Source:MGI Symbol;Acc:MGI:3801966] | Gm16031 | lncRNA |
| ENSMUSG00000090291 | 14.198942 | 4.89175939 | 1.59042735 | 5.96E-05 | 0.00963581 | Up | 278795 | leucine rich repeat containing 10B [Source:MGI Symbol;Acc:MGI:2685551] | Lrrc10b | protein_coding |
| ENSMUSG00000090336 | 6.70149595 | 2.70903824 | 1.3546878 | 0.00105465 | 0.04767177 | Up | 403185 | CFAP97 domain containing 2 [Source:MGI Symbol;Acc:MGI:2685952] | Cfap97d2 | protein_coding |
| ENSMUSG00000091415 | 11.5163403 | 3.21346289 | 1.61477638 | 0.00085886 | 0.0426477 | Up | 633979 | adenylate kinase 9 [Source:MGI Symbol;Acc:MGI:2685080] | Ak9 | protein_coding |
| ENSMUSG00000093587 | 3.10448096 | 3.34921956 | 1.64778166 | 0.00114455 | 0.04956593 | Up | NA | predicted gene, 20554 [Source:MGI Symbol;Acc:MGI:5295661] | Gm20554 | lncRNA |
| ENSMUSG00000094282 | 4.50343968 | 4.85847214 | 2.39893482 | 0.00071885 | 0.03887253 | Up | 546886 | cilia and flagella associated protein 73 [Source:MGI Symbol;Acc:MGI:3779542] | Cfap73 | protein_coding |
| ENSMUSG00000095079 | 257.77967 | 3.20706788 | 1.46873332 | 0.00051405 | 0.03257298 | Up | NA | immunoglobulin heavy constant alpha [Source:MGI Symbol;Acc:MGI:96444] | Igha | IG_C_gene |
| ENSMUSG00000095442 | 63.5342954 | 7.8196876 | 3.29207002 | 0.00012401 | 0.01417611 | Up | NA | immunoglobulin heavy variable 1-4 [Source:MGI Symbol;Acc:MGI:4439618] | Ighv1-4 | IG_V_gene |
| ENSMUSG00000095794 | 44.8495411 | 5.91103715 | 2.09029959 | 7.19E-05 | 0.01092085 | Up | NA | immunoglobulin kappa variable 6-17 [Source:MGI Symbol;Acc:MGI:1330833] | Igkv6-17 | IG_V_gene |
| ENSMUSG00000096141 | 2.61292468 | 5.8228735 | 2.78346998 | 0.00031259 | 0.02372396 | Up | 627872 | dynein, axonemal, heavy chain 7A [Source:MGI Symbol;Acc:MGI:2685838] | Dnah7a | protein_coding |
| ENSMUSG00000097231 | 10.5296374 | 0.02436971 | 0.06927648 | 0.0008853 | 0.04345904 | Up | NA | predicted gene, 26852 [Source:MGI Symbol;Acc:MGI:5477346] | Gm26852 | lncRNA |
| ENSMUSG00000100594 | 139.603726 | -0.6920799 | 0.20557453 | 3.01E-05 | 0.00640811 | Down | 102640772 | RIKEN cDNA 2810414N06 gene [Source:MGI Symbol;Acc:MGI:1919065] | 2810414N06Rik | lncRNA |

| | | | | | | | | | | |
|---|---|---|---|---|---|---|---|---|---|---|
| ENSMUSG00000101431 | 28.5620429 | -0.8919513 | 0.28516992 | 6.10E-05 | 0.0097703 | Down | NA | predicted gene 7901 [Source:MGI Symbol;Acc:MGI:3645047] | Gm7901 | processed_pseudogene |
| ENSMUSG00000102496 | 19.5034949 | 0.03779815 | 0.07989894 | 0.00115751 | 0.04971037 | Up | NA | predicted gene, 36989 [Source:MGI Symbol;Acc:MGI:5610217] | Gm36989 | TEC |
| ENSMUSG00000102737 | 4.01739847 | 2.53415836 | 0.94499519 | 0.00041148 | 0.02823917 | Up | 74227 | RIKEN cDNA 1700016A09 gene [Source:MGI Symbol;Acc:MGI:1921477] | 1700016A09Rik | TEC |
| ENSMUSG00000108841 | 13.6770768 | 3.58733674 | 1.49135249 | 0.00035674 | 0.02568902 | Up | 268729 | FERM and PDZ domain containing 2 [Source:MGI Symbol;Acc:MGI:2685472] | Frmpd2 | protein_coding |
| ENSMUSG00000109311 | 12.0742218 | 2.66964623 | 0.96278664 | 0.00016298 | 0.01580678 | Up | 101521 | expressed sequence AI314278 [Source:MGI Symbol;Acc:MGI:2141898] | AI314278 | lncRNA |
| ENSMUSG00000110170 | 29.7654035 | 1.73987676 | 0.69800309 | 0.00035614 | 0.02568902 | Up | NA | ST6 (alpha-N-acetyl-neuraminyl-2,3-beta-galactosyl-1,3)-N-acetylgalactosaminide alpha-2,6-sialyltransferase 2 [Source:MGI Symbol;Acc:MGI:107553] | St6galnac2 | protein_coding |
| ENSMUSG00000110332 | 32.0453676 | 2.48639513 | 1.0618137 | 0.00045225 | 0.02990199 | Up | NA | predicted gene, 19935 [Source:MGI Symbol;Acc:MGI:5012120] | Gm19935 | protein_coding |
| ENSMUSG00000110622 | 5.03007892 | 7.08900396 | 3.29102088 | 0.00036408 | 0.02602351 | Up | 637079 | IQ motif containing N [Source:MGI Symbol;Acc:MGI:3708784] | Iqcn | protein_coding |
| ENSMUSG00000112657 | 8.86551753 | 4.39472958 | 1.48916748 | 9.80E-05 | 0.01275873 | Up | NA | cDNA sequence BC106175 [Source:MGI Symbol;Acc:MGI:3628444] | BC106175 | transcribed_processed_pseudogene |
| ENSMUSG00000114994 | 3.80004343 | 6.33035433 | 3.35173648 | 0.00090675 | 0.04407147 | Up | NA | predicted gene, 49097 [Source:MGI Symbol;Acc:MGI:6118490] | Gm49097 | lncRNA |
