# Supplementary material for: The effect of aldafermin expressing-Escherichia coli Nissle 1917 along with dietary change on visceral adipose tissue in MASLD mouse model
Source: Int J Obes (Lond). 2025 Apr 10;49(7):1334–44. doi: 10.1038/s41366-025-01774-w (PMC12283412; doi:10.1038/s41366-025-01774-w)
Supplement: Supplementary file 5 — Supplementary table 6 [file 41366_2025_1774_MOESM5_ESM.pdf]

Supplementary table 6. DEGs observed in liver when EcNA was compared to CTRL

EcNA vs CTRL liver

ENSEMBL	baseMean	log2FoldChange	lfcSE	pvalue	padj	UD	entrezgene_id	description	external_gene_name	gene_biotype
ENSMUSG00000017723	95.5561063	-0.061448	0.11743662	0.00076288	0.04958899	Down	67701	WAP four-disulfide core domain 2 [Source:MGI Symbol;Acc:MGI:1914951]	Wfdc2	protein_coding
ENSMUSG00000037894	938.184098	-0.2998693	0.12317308	0.00076395	0.04958899	Down	51788	H2A.Z variant histone 1 [Source:MGI Symbol;Acc:MGI:1888388]	H2az1	protein_coding
ENSMUSG00000004626	487.833797	-0.2164719	0.08303844	0.0007544	0.04948701	Down	20911	syntaxin binding protein 2 [Source:MGI Symbol;Acc:MGI:107370]	Stxbp2	protein_coding
ENSMUSG00000016344	1322.01464	-0.2626868	0.10401807	0.00074662	0.04923722	Down	66496	pancreatic progenitor cell differentiation and proliferation factor [Source:MGI Symbol;Acc:MGI:1913746]	Ppdpf	protein_coding
ENSMUSG00000030086	52.335563	-0.6982407	0.3103668	0.00073955	0.04903176	Down	66098	coiled-coil-helix-coiled-coil-helix domain containing 6 [Source:MGI Symbol;Acc:MGI:1913348]	Chchd6	protein_coding
ENSMUSG00000066072	3215.81213	-0.0140618	0.07671262	0.00073894	0.04903176	Down	13117	cytochrome P450, family 4, subfamily a, polypeptide 10 [Source:MGI Symbol;Acc:MGI:88611]	Cyp4a10	protein_coding
ENSMUSG00000021039	252.03803	0.27306093	0.10954413	0.00073013	0.04893066	Up	66354	SNW domain containing 1 [Source:MGI Symbol;Acc:MGI:1913604]	Snw1	protein_coding
ENSMUSG00000003623	7521.43337	-0.607086	0.26767841	0.00071948	0.04847858	Down	74114	carnitine O-octanoyltransferase [Source:MGI Symbol;Acc:MGI:1921364]	Crot	protein_coding
ENSMUSG00000024424	7123.88659	0.40767619	0.16253261	0.00070255	0.0475967	Up	72747	tetratricopeptide repeat domain 39C [Source:MGI Symbol;Acc:MGI:1919997]	Ttc39c	protein_coding
ENSMUSG00000062382	155.152735	-0.3745731	0.15888007	0.00069224	0.04740129	Down	434624	ferritin light polypeptide 1, pseudogene 1 [Source:MGI Symbol;Acc:MGI:3779109]	Ftl1-ps1	protein_coding
ENSMUSG00000079641	1324.91971	0.46088898	0.19656678	0.00069584	0.04740129	Up	67248	ribosomal protein L39 [Source:MGI Symbol;Acc:MGI:1914498]	Rpl39	protein_coding
ENSMUSG00000063015	3161.25019	-0.2249557	0.09218047	0.0006779	0.04669242	Down	12453	cyclin I [Source:MGI Symbol;Acc:MGI:1341077]	Ccni	protein_coding
ENSMUSG00000112774	128.539717	-0.1066828	0.34371261	0.00065841	0.04560291	Down	NA	predicted gene, 36041 [Source:MGI Symbol;Acc:MGI:5595200]	Gm36041	lncRNA
ENSMUSG00000020865	3555.63105	-0.8127349	0.35701882	0.00064808	0.04513989	Down	76408	ATP-binding cassette, sub-family C (CFTR/MRP), member 3 [Source:MGI Symbol;Acc:MGI:1923658]	Abcc3	
ENSMUSG00000067656	24.9158516	-0.0219023	0.07947375	0.00063482	0.0444658	Down	171405	solute carrier family 22, member 27 [Source:MGI Symbol;Acc:MGI:3042283]	Slc22a27	protein_coding
ENSMUSG00000020407	30.0173764	-0.7721604	0.33361603	0.00062937	0.04433479	Down	22271	uridine phosphorylase 1 [Source:MGI Symbol;Acc:MGI:1097668]	Upp1	protein_coding
ENSMUSG00000006218	76.1452647	-0.5793709	0.24607441	0.00061107	0.04355068	Down	277743	family with sequence similarity 131, member C [Source:MGI Symbol;Acc:MGI:2685539]	Fam131c	protein_coding
ENSMUSG00000026131	1423.91856	0.36868086	0.15032756	0.00061473	0.04355068	Up	13518	dystonin [Source:MGI Symbol;Acc:MGI:104627]	Dst	protein_coding
ENSMUSG00000035441	361.136902	-0.3396449	0.13765793	0.00061376	0.04355068	Down	338367	myosin ID [Source:MGI Symbol;Acc:MGI:107728]	Myo1d	protein_coding
ENSMUSG00000041769	432.03343	-0.3061111	0.12128677	0.00060984	0.04355068	Down	52432	protein phosphatase 2, regulatory subunit B, delta [Source:MGI Symbol;Acc:MGI:1289252]	Ppp2r2d	protein_coding
ENSMUSG00000005225	431.630137	0.25989868	0.10180496	0.00060028	0.04352186	Up	231999	pleckstrin homology domain containing, family A (phosphoinositide binding specific) member 8 [Source:MGI Symbol;Acc:MGI:2681164]	Plekha8	protein_coding
ENSMUSG00000068220	405.177295	-0.0610308	0.11700675	0.00059343	0.0432788	Down	16852	lectin, galactose binding, soluble 1 [Source:MGI Symbol;Acc:MGI:96777]	Lgals1	protein_coding
ENSMUSG00000022210	2062.61996	-0.2651668	0.09997641	0.00058812	0.04314516	Down	28200	dehydrogenase/reductase (SDR family) member 4 [Source:MGI Symbol;Acc:MGI:90169]	Dhrs4	protein_coding
ENSMUSG00000071713	91.4266681	0.47452181	0.19957201	0.00058554	0.04314516	Up	12983	colony stimulating factor 2 receptor, beta, low-affinity (granulocyte-macrophage) [Source:MGI Symbol;Acc:MGI:1339759]	Csf2rb	protein_coding
ENSMUSG00000020003	1401.94646	-0.2783096	0.1079752	0.00057626	0.04304595	Down	18634	peroxisomal biogenesis factor 7 [Source:MGI Symbol;Acc:MGI:1321392]	Pex7	protein_coding
ENSMUSG00000062908	5217.34077	-0.4089603	0.16631315	0.00057983	0.04304595	Down	11364	acyl-Coenzyme A dehydrogenase, medium chain [Source:MGI Symbol;Acc:MGI:87867]	Acadm	protein_coding
ENSMUSG00000090733	1386.64663	0.57793799	0.24495318	0.00057659	0.04304595	Up	57294	ribosomal protein S27 [Source:MGI Symbol;Acc:MGI:1888676]	Rps27	protein_coding
ENSMUSG00000007415	912.620227	-0.178686	0.06329653	0.00054235	0.04219371	Down	67210	GATA zinc finger domain containing 1 [Source:MGI Symbol;Acc:MGI:1914460]	Gatad1	protein_coding
ENSMUSG00000018899	372.040664	0.44705587	0.1828553	0.00055241	0.04219371	Up	16362	interferon regulatory factor 1 [Source:MGI Symbol;Acc:MGI:96590]	Irf1	protein_coding
ENSMUSG00000021226	79.4932275	-0.03567	0.08760685	0.0005535	0.04219371	Down	171210	acyl-CoA thioesterase 2 [Source:MGI Symbol;Acc:MGI:2159605]	Acot2	protein_coding
ENSMUSG00000022956	1098.68821	0.30638028	0.11847422	0.00055814	0.04219371	Up	28080	ATP synthase, H+ transporting, mitochondrial F1 complex, O subunit [Source:MGI Symbol;Acc:MGI:106341]	Atp5o	protein_coding
ENSMUSG00000023274	32.1185778	1.43141047	0.61402412	0.00053859	0.04219371	Up	12504	CD4 antigen [Source:MGI Symbol;Acc:MGI:88335]	Cd4	protein_coding
ENSMUSG00000028420	1221.57929	-0.2113012	0.07714186	0.00053792	0.04219371	Down	52076	transmembrane protein 38B [Source:MGI Symbol;Acc:MGI:1098718]	Tmem38b	protein_coding
ENSMUSG00000039063	1324.45315	-0.2649925	0.10171688	0.00055803	0.04219371	Down	67856	enoyl Coenzyme A hydratase domain containing 3 [Source:MGI Symbol;Acc:MGI:1915106]	Echdc3	protein_coding
ENSMUSG00000049553	291.967851	0.41439281	0.1684838	0.00055213	0.04219371	Up	20019	polymerase (RNA) I polypeptide A [Source:MGI Symbol;Acc:MGI:1096397]	Polr1a	protein_coding
ENSMUSG00000029650	801.030079	0.47739592	0.19334234	0.00052662	0.041853	Up	71706	solute carrier family 46, member 3 [Source:MGI Symbol;Acc:MGI:1918956]	Slc46a3	protein_coding
ENSMUSG00000028669	393.022625	-0.3467386	0.13663952	0.00051817	0.04144678	Down	66193	PITH (C-terminal proteasome-interacting domain of thioredoxin-like) domain containing 1 [Source:MGI Symbol;Acc:MGI:1913443]	Pithd1	protein_coding
ENSMUSG00000001794	2120.01062	-0.2155772	0.08106796	0.00051418	0.04139447	Down	12336	calpain, small subunit 1 [Source:MGI Symbol;Acc:MGI:88266]	Capns1	protein_coding
ENSMUSG00000029030	892.015721	-0.2362039	0.08746746	0.00049719	0.04028851	Down	67808	transformation related protein 63 regulated like [Source:MGI Symbol;Acc:MGI:1915058]	Tprgl	protein_coding
ENSMUSG00000032402	277.321626	0.4298631	0.17336403	0.00049375	0.0402735	Up	17127	SMAD family member 3 [Source:MGI Symbol;Acc:MGI:1201674]	Smad3	protein_coding
ENSMUSG00000033257	276.587437	0.39307455	0.15569611	0.000488	0.04006734	Up	67534	tubulin tyrosine ligase-like family, member 4 [Source:MGI Symbol;Acc:MGI:1914784]	Ttll4	protein_coding
ENSMUSG00000006777	19.2141842	-0.0403312	0.09142354	0.00047005	0.03885128	Down	94179	keratin 23 [Source:MGI Symbol;Acc:MGI:2148866]	Krt23	protein_coding
ENSMUSG00000013858	2489.31986	-0.214173	0.07691953	0.00046609	0.03878243	Down	216157	transmembrane protein 259 [Source:MGI Symbol;Acc:MGI:2177957]	Tmem259	protein_coding
ENSMUSG00000005125	144.258131	-0.5983451	0.24316669	0.00046239	0.03873419	Down	17988	N-myc downstream regulated gene 1 [Source:MGI Symbol;Acc:MGI:1341799]	Ndrg1	protein_coding
ENSMUSG00000094786	156.248558	0.72071031	0.29452225	0.00045008	0.03796019	Up	NA	predicted gene 14403 [Source:MGI Symbol;Acc:MGI:3649813]	Gm14403	protein_coding
ENSMUSG00000028407	42.6111773	0.61286017	0.24514624	0.00042414	0.03601672	Up	100504309	small integral membrane protein 27 [Source:MGI Symbol;Acc:MGI:1913684]	Smim27	protein_coding
ENSMUSG00000019923	830.036349	-0.2664266	0.09752981	0.00041812	0.0357506	Down	52696	ZW10 interactor [Source:MGI Symbol;Acc:MGI:1289227]	Zwint	protein_coding
ENSMUSG00000098708	25.9867837	0.87133994	0.35151728	0.00041555	0.0357506	Up	NA	predicted gene 27252 [Source:MGI Symbol;Acc:MGI:5521095]	Gm27252	lncRNA
ENSMUSG00000020308	441.959344	-0.3041971	0.11128839	0.00041126	0.03565571	Down	110012	tubulin polyglutamylase complex subunit 1 [Source:MGI Symbol;Acc:MGI:106618]	Tpgs1	protein_coding

ENSMUSG00000002393	1680.84588	-0.2667755	0.09809409	0.00039497	0.03448461	Down	13864	nuclear receptor subfamily 2, group F, member 6 [Source:MGI Symbol;Acc:MGI:1352453]	Nr2f6	protein_coding
ENSMUSG00000002831	64.8013225	-0.0286228	0.08292523	0.0003903	0.03448461	Down	57435	perilipin 4 [Source:MGI Symbol;Acc:MGI:1929709]	Plin4	protein_coding
ENSMUSG00000037926	314.200931	0.42714877	0.16448346	0.00039245	0.03448461	Up	237860	slingshot protein phosphatase 2 [Source:MGI Symbol;Acc:MGI:2679255]	Ssh2	protein_coding
ENSMUSG00000073468	397.145978	-0.3341495	0.12557631	0.00039085	0.03448461	Down	106489	SFT2 domain containing 1 [Source:MGI Symbol;Acc:MGI:1918689]	Sft2d1	protein_coding
ENSMUSG00000033066	162.315532	-0.7041781	0.27942	0.00038198	0.03431728	Down	14457	growth arrest specific 7 [Source:MGI Symbol;Acc:MGI:1202388]	Gas7	protein_coding
ENSMUSG00000025494	396.329751	-0.3749444	0.14219446	0.00037423	0.0338688	Down	24058	single immunoglobulin and toll-interleukin 1 receptor (TIR) domain [Source:MGI Symbol;Acc:MGI:1344402]	Sigirr	protein_coding
ENSMUSG00000063354	530.715007	0.62840467	0.24572878	0.00037426	0.0338688	Up	72027	solute carrier family 39 (zinc transporter), member 4 [Source:MGI Symbol;Acc:MGI:1919277]	Slc39a4	protein_coding
ENSMUSG00000023961	234.269977	0.30420344	0.11229563	0.00036879	0.03386815	Up	224794	ectonucleotide pyrophosphatase/phosphodiesterase 4 [Source:MGI Symbol;Acc:MGI:2682634]	Enpp4	protein_coding
ENSMUSG00000052271	25.0032774	2.52242612	1.0349861	0.00035623	0.03295895	Up	17341	basic helix-loop-helix family, member a15 [Source:MGI Symbol;Acc:MGI:891976]	Bhlha15	protein_coding
ENSMUSG00000027947	867.362111	0.75034233	0.29214111	0.00035253	0.03286247	Up	16194	interleukin 6 receptor, alpha [Source:MGI Symbol;Acc:MGI:105304]	Il6ra	protein_coding
ENSMUSG00000035674	616.045505	0.59077051	0.22772655	0.00034344	0.03225727	Up	66091	NADH:ubiquinone oxidoreductase subunit A3 [Source:MGI Symbol;Acc:MGI:1913341]	Ndufa3	protein_coding
ENSMUSG00000044646	58.0000496	1.590598	0.63547178	0.00034326	0.03225727	Up	207259	zinc finger and BTB domain containing 7C [Source:MGI Symbol;Acc:MGI:2443302]	Zbtb7c	protein_coding
ENSMUSG00000052435	152.717751	-0.5462667	0.21024288	0.00033756	0.03219285	Down	110794	CCAAT/enhancer binding protein (C/EBP), epsilon [Source:MGI Symbol;Acc:MGI:103572]	Cebpe	protein_coding
ENSMUSG00000040661	509.860809	0.38635773	0.14426552	0.00033456	0.03215377	Up	81000	RAD54 like 2 (S. cerevisiae) [Source:MGI Symbol;Acc:MGI:1933196]	Rad54l2	protein_coding
ENSMUSG00000074884	890.935862	-0.4003759	0.14875407	0.00032005	0.03100018	Down	378702	small EDRK-rich factor 2 [Source:MGI Symbol;Acc:MGI:1337041]	Serf2	protein_coding
ENSMUSG00000001156	114.740954	0.61151844	0.23347245	0.00031596	0.03084446	Up	17119	MAX dimerization protein 1 [Source:MGI Symbol;Acc:MGI:96908]	Mxd1	protein_coding
ENSMUSG00000015312	91.5232579	-0.0384482	0.08991947	0.00030865	0.03037013	Down	17873	growth arrest and DNA-damage-inducible 45 beta [Source:MGI Symbol;Acc:MGI:107776]	Gadd45b	protein_coding
ENSMUSG00000034892	757.842462	0.77997775	0.3016808	0.00030865	0.03037013	Up	20090	ribosomal protein S29 [Source:MGI Symbol;Acc:MGI:107681]	Rps29	protein_coding
ENSMUSG00000037876	554.482107	0.32189352	0.11661697	0.00030759	0.03037013	Up	108829	jumonji domain containing 1C [Source:MGI Symbol;Acc:MGI:1918614]	Jmjd1c	protein_coding
ENSMUSG00000020015	326.121783	0.28900569	0.10395181	0.00029406	0.02963984	Up	237459	cyclin-dependent kinase 17 [Source:MGI Symbol;Acc:MGI:97517]	Cdk17	protein_coding
ENSMUSG00000067288	537.45046	0.68566636	0.26071956	0.00028714	0.02938854	Up	54127	ribosomal protein S28 [Source:MGI Symbol;Acc:MGI:1859516]	Rps28	protein_coding
ENSMUSG00000091867	36.8082384	-0.0196095	0.07856428	0.00028919	0.02938854	Down	233005	cytochrome P450, family 2, subfamily a, polypeptide 22 [Source:MGI Symbol;Acc:MGI:3648316]	Cyp2a22	protein_coding
ENSMUSG00000029250	603.401512	0.26247001	0.09258975	0.00028084	0.02901539	Up	231329	polymerase (RNA) II (DNA directed) polypeptide B [Source:MGI Symbol;Acc:MGI:2388280]	Polr2b	protein_coding
ENSMUSG00000017146	24.5027531	0.96008722	0.36483788	0.00027529	0.02868085	Up	12189	breast cancer 1, early onset [Source:MGI Symbol;Acc:MGI:104537]	Brca1	protein_coding
ENSMUSG00000066861	13.1120053	1.10848118	0.41949316	0.00027498	0.02868085	Up	23960	2'-5' oligoadenylate synthetase 1G [Source:MGI Symbol;Acc:MGI:97429]	Oas1g	protein_coding
ENSMUSG00000073079	30.8704189	0.93190576	0.35214652	0.00026689	0.02828165	Up	24067	signal recognition particle 54A [Source:MGI Symbol;Acc:MGI:1346087]	Srp54a	protein_coding
ENSMUSG00000024525	63.7969424	-0.6704805	0.25049669	0.00026029	0.02781956	Down	114663	inositol monophosphatase 2 [Source:MGI Symbol;Acc:MGI:2149728]	Impa2	protein_coding
ENSMUSG00000062116	74.5926895	-0.4852418	0.17763546	0.00025336	0.02735733	Down	232853	zinc finger protein 954 [Source:MGI Symbol;Acc:MGI:1917764]	Zfp954	protein_coding
ENSMUSG00000073888	92.4322063	-0.5767172	0.21376577	0.00025376	0.02735733	Down	100039863	chemokine (C-C motif) ligand 27A [Source:MGI Symbol;Acc:MGI:1343459]	Ccl27a	protein_coding
ENSMUSG00000000440	192.896436	-0.9582699	0.35969466	0.00023971	0.02630002	Down	19016	peroxisome proliferator activated receptor gamma [Source:MGI Symbol;Acc:MGI:97747]	Pparg	protein_coding
ENSMUSG00000035509	70.322952	-0.5151	0.18702152	0.00023805	0.02630002	Down	213311	F-box and leucine-rich repeat protein 21 [Source:MGI Symbol;Acc:MGI:2442921]	Fbxl21	protein_coding
ENSMUSG00000032776	268.519599	0.40588022	0.14559758	0.00023253	0.02597243	Up	244049	multiple C2 domains, transmembrane 2 [Source:MGI Symbol;Acc:MGI:2685335]	Mctp2	protein_coding
ENSMUSG00000113902	346.175709	0.74235412	0.27320652	0.000222	0.02502191	Up	102631912	NADH:ubiquinone oxidoreductase subunit B1 [Source:MGI Symbol;Acc:MGI:3780865]	Ndufb1	protein_coding
ENSMUSG00000032116	1963.88416	0.27031881	0.09132819	0.00021487	0.02444048	Up	16430	STT3, subunit of the oligosaccharyltransferase complex, homolog A (S. cerevisiae) [Source:MGI Symbol;Acc:MGI:105124]	Stt3a	protein_coding
ENSMUSG00000018326	1777.92987	-0.175811	0.05513509	0.00021134	0.02439569	Down	54401	tyrosine 3-monooxygenase/tryptophan 5-monooxygenase activation protein, beta polypeptide [Source:MGI Symbol;Acc:MGI:1891917]	Ywhab	protein_coding
ENSMUSG00000027502	1250.49551	-0.3262053	0.11331007	0.00021251	0.02439569	Down	66404	replication termination factor 2 [Source:MGI Symbol;Acc:MGI:1913654]	Rtf2	protein_coding
ENSMUSG00000021738	313.007605	0.39898756	0.14111841	0.00020555	0.02404131	Up	246103	ataxin 7 [Source:MGI Symbol;Acc:MGI:2179277]	Atxn7	protein_coding
ENSMUSG00000087579	233.115282	-0.8920528	0.32304251	0.00018922	0.02234292	Down	668215	Hectd2, opposite strand [Source:MGI Symbol;Acc:MGI:1919243]	Hectd2os	lncRNA
ENSMUSG00000021360	363.556933	0.38636609	0.13287767	0.0001828	0.02179173	Up	14538	glucosaminyl (N-acetyl) transferase 2, I-branching enzyme [Source:MGI Symbol;Acc:MGI:1100870]	Gcnt2	protein_coding
ENSMUSG00000028672	4452.557	-0.4622056	0.15895752	0.00017929	0.02158048	Down	15356	3-hydroxy-3-methylglutaryl-Coenzyme A lyase [Source:MGI Symbol;Acc:MGI:96158]	Hmgcl	protein_coding
ENSMUSG00000089809	192.140582	0.72239587	0.25688434	0.00017578	0.0213661	Up	320292	RasGEF domain family, member 1B [Source:MGI Symbol;Acc:MGI:2443755]	Rasgef1b	protein_coding
ENSMUSG00000023967	467.329369	-0.2660718	0.08830028	0.00016587	0.0203605	Down	68565	mitochondrial ribosomal protein S18A [Source:MGI Symbol;Acc:MGI:1915815]	Mrps18a	protein_coding
ENSMUSG00000026615	1889.16748	0.23265083	0.07509737	0.00016262	0.0203605	Up	107508	glutamyl-prolyl-tRNA synthetase [Source:MGI Symbol;Acc:MGI:97838]	Eprs	protein_coding
ENSMUSG00000028240	995.186548	1.33829104	0.47954872	0.00016375	0.0203605	Up	13122	cytochrome P450, family 7, subfamily a, polypeptide 1 [Source:MGI Symbol;Acc:MGI:106091]	Cyp7a1	protein_coding
ENSMUSG00000040466	1562.6926	-0.3275801	0.11062797	0.00016541	0.0203605	Down	233016	biliverdin reductase B (flavin reductase (NADPH)) [Source:MGI Symbol;Acc:MGI:2385271]	Blvrb	protein_coding
ENSMUSG00000029131	801.159368	0.27968692	0.09301354	0.00015916	0.02034357	Up	23950	DnaJ heat shock protein family (Hsp40) member B6 [Source:MGI Symbol;Acc:MGI:1344381]	Dnajb6	protein_coding
ENSMUSG00000030278	147.665025	-0.0299519	0.08380073	0.00015869	0.02034357	Down	14311	cell death-inducing DFFA-like effector c [Source:MGI Symbol;Acc:MGI:95585]	Cidec	protein_coding
ENSMUSG00000022508	738.680018	1.83921831	0.65685914	0.00015238	0.01988654	Up	12053	B cell leukemia/lymphoma 6 [Source:MGI Symbol;Acc:MGI:107187]	Bcl6	protein_coding
ENSMUSG00000034641	95.4149181	0.64610935	0.22484542	0.0001512	0.01988654	Up	217305	CD300 molecule like family member d [Source:MGI Symbol;Acc:MGI:2442358]	Cd300ld	protein_coding

ENSMUSG00000022620	959.90531	-0.5246395	0.180644	0.0001402	0.01869033	Down	11883	arylsulfatase A [Source:MGI Symbol;Acc:MGI:88077]	Arsa	protein_coding
ENSMUSG00000079334	597.480428	-0.3478707	0.11482667	0.00013617	0.01835046	Down	56441	N(alpha)-acetyltransferase 80, NatH catalytic subunit [Source:MGI Symbol;Acc:MGI:1888902]	Naa80	protein_coding
ENSMUSG00000024526	138.180413	-0.0114482	0.07608301	0.00012673	0.0181397	Down	12683	cell death-inducing DNA fragmentation factor, alpha subunit-like effector A [Source:MGI Symbol;Acc:MGI:1270845]	Cidea	protein_coding
ENSMUSG00000025278	1312.18608	0.34764084	0.11651732	0.00013314	0.0181397	Up	286940	filamin, beta [Source:MGI Symbol;Acc:MGI:2446089]	Flnb	protein_coding
ENSMUSG00000031808	115.40815	-0.560478	0.19050892	0.00013024	0.0181397	Down	26457	solute carrier family 27 (fatty acid transporter), member 1 [Source:MGI Symbol;Acc:MGI:1347098]	Slc27a1	protein_coding
ENSMUSG00000053897	461.223682	-0.2819452	0.09115647	0.00012883	0.0181397	Down	67547	solute carrier family 39 (metal ion transporter), member 8 [Source:MGI Symbol;Acc:MGI:1914797]	Slc39a8	protein_coding
ENSMUSG00000071669	62.0220175	0.6163289	0.2096835	0.00013193	0.0181397	Up	74478	sorting nexin 29 [Source:MGI Symbol;Acc:MGI:1921728]	Snx29	protein_coding
ENSMUSG00000079429	93.1134246	0.59784199	0.20453515	0.00012918	0.0181397	Up	100040766	maestro heat-like repeat family member 2A [Source:MGI Symbol;Acc:MGI:3705228]	Mroh2a	protein_coding
ENSMUSG00000032231	216.313755	-0.9139134	0.31111041	0.00011241	0.01639636	Down	12306	annexin A2 [Source:MGI Symbol;Acc:MGI:88246]	Anxa2	protein_coding
ENSMUSG00000011257	738.371292	0.29037304	0.0922353	0.00010671	0.01575018	Up	230721	poly(A) binding protein, cytoplasmic 4 [Source:MGI Symbol;Acc:MGI:2385206]	Pabpc4	protein_coding
ENSMUSG00000024026	6601.03024	-0.2970304	0.09478841	0.00010538	0.01574062	Down	109801	glyoxalase 1 [Source:MGI Symbol;Acc:MGI:95742]	Glo1	protein_coding
ENSMUSG00000019944	931.989255	0.5002093	0.16323169	0.00010019	0.01514852	Up	69288	Rho-related BTB domain containing 1 [Source:MGI Symbol;Acc:MGI:1916538]	Rhobtb1	protein_coding
ENSMUSG00000050856	491.216862	0.65528035	0.21750979	9.83E-05	0.01504996	Up	11958	ATP synthase, H+ transporting, mitochondrial F1F0 complex, subunit E [Source:MGI Symbol;Acc:MGI:106636]	Atp5k	protein_coding
ENSMUSG00000017868	1412.99981	-0.7706991	0.25672546	9.59E-05	0.01485761	Down	27219	serum/glucocorticoid regulated kinase 2 [Source:MGI Symbol;Acc:MGI:1351318]	Sgk2	protein_coding
ENSMUSG00000020719	4650.5744	0.30035129	0.09420824	9.47E-05	0.01485761	Up	13207	DEAD box helicase 5 [Source:MGI Symbol;Acc:MGI:105037]	Ddx5	protein_coding
ENSMUSG00000022445	16150.2118	-0.3234323	0.10302898	9.52E-05	0.01485761	Down	76279	cytochrome P450, family 2, subfamily d, polypeptide 26 [Source:MGI Symbol;Acc:MGI:1923529]	Cyp2d26	protein_coding
ENSMUSG00000049109	22.5828291	-0.0223423	0.07974486	9.52E-05	0.01485761	Down	210757	thymocyte selection associated [Source:MGI Symbol;Acc:MGI:2443552]	Themis	protein_coding
ENSMUSG00000086813	15.397116	-1.1815415	0.38786675	8.99E-05	0.01467076	Down	NA	predicted gene 13657 [Source:MGI Symbol;Acc:MGI:3650031]	Gm13657	lncRNA
ENSMUSG00000002058	118.583622	-0.7128864	0.23376743	8.18E-05	0.01400196	Down	22248	unc-119 lipid binding chaperone [Source:MGI Symbol;Acc:MGI:1328357]	Unc119	protein_coding
ENSMUSG00000024924	31.7512168	-0.0304938	0.08418235	8.47E-05	0.01400196	Down	22359	very low density lipoprotein receptor [Source:MGI Symbol;Acc:MGI:98935]	Vldlr	protein_coding
ENSMUSG00000027602	753.835534	-0.3095522	0.10130607	8.34E-05	0.01400196	Down	66734	microtubule-associated protein 1 light chain 3 alpha [Source:MGI Symbol;Acc:MGI:1915661]	Map1lc3a	protein_coding
ENSMUSG00000037440	544.4651	-0.0413015	0.09266819	8.24E-05	0.01400196	Down	22361	vanin 1 [Source:MGI Symbol;Acc:MGI:108395]	Vnn1	protein_coding
ENSMUSG00000052712	1297.29605	-0.3472397	0.11005367	8.44E-05	0.01400196	Down	80748	cDNA sequence BC004004 [Source:MGI Symbol;Acc:MGI:2136782]	BC004004	protein_coding
ENSMUSG00000008734	26.2851085	-0.0204938	0.07896468	6.61E-05	0.01170846	Down	64297	G protein-coupled receptor, family C, group 5, member B [Source:MGI Symbol;Acc:MGI:1927596]	Gprc5b	protein_coding
ENSMUSG00000037622	2864.77275	-0.3818625	0.11713348	6.48E-05	0.01163543	Down	230796	WD and tetratricopeptide repeats 1 [Source:MGI Symbol;Acc:MGI:2685541]	Wdtc1	protein_coding
ENSMUSG00000034525	268.236152	0.44442467	0.13832083	6.04E-05	0.01101262	Up	218333	interactor of little elongation complex ELL subunit 1 [Source:MGI Symbol;Acc:MGI:2385865]	Ice1	protein_coding
ENSMUSG00000012187	35.3603701	-0.0281399	0.08276121	5.87E-05	0.01085865	Down	68393	monoacylglycerol O-acyltransferase 1 [Source:MGI Symbol;Acc:MGI:1915643]	Mogat1	protein_coding
ENSMUSG00000057228	1361.39746	0.38247697	0.11613182	5.82E-05	0.01085865	Up	23923	aminoadipate aminotransferase [Source:MGI Symbol;Acc:MGI:1345167]	Aadat	protein_coding
ENSMUSG00000021259	62.8426129	-0.0452451	0.09667006	5.22E-05	0.00996054	Down	13116	cytochrome P450, family 46, subfamily a, polypeptide 1 [Source:MGI Symbol;Acc:MGI:1341877]	Cyp46a1	protein_coding
ENSMUSG00000023495	226.267155	-0.646774	0.19975388	4.95E-05	0.00968358	Down	59092	poly(rC) binding protein 4 [Source:MGI Symbol;Acc:MGI:1890471]	Pcbp4	protein_coding
ENSMUSG00000030451	685.208952	0.38566486	0.11687734	5.00E-05	0.00968358	Up	15204	HECT and RLD domain containing E3 ubiquitin protein ligase 2 [Source:MGI Symbol;Acc:MGI:103234]	Herc2	protein_coding
ENSMUSG00000032392	403.241687	0.4565689	0.13778005	4.48E-05	0.00895561	Up	214424	poly (ADP-ribose) polymerase family, member 16 [Source:MGI Symbol;Acc:MGI:2446133]	Parp16	protein_coding
ENSMUSG00000032350	5254.27119	-0.5693238	0.17126538	4.16E-05	0.00844584	Down	14629	glutamate-cysteine ligase, catalytic subunit [Source:MGI Symbol;Acc:MGI:104990]	Gclc	protein_coding
ENSMUSG00000073563	547.265647	0.33513808	0.09940404	4.15E-05	0.00844584	Up	70425	casein kinase 1, gamma 3 [Source:MGI Symbol;Acc:MGI:1917675]	Csnk1g3	protein_coding
ENSMUSG00000019851	1937.13984	-0.2872571	0.08394219	3.95E-05	0.00830001	Down	64058	PERP, TP53 apoptosis effector [Source:MGI Symbol;Acc:MGI:1929938]	Perp	protein_coding
ENSMUSG00000025396	558.047733	-1.2815043	0.39237542	3.71E-05	0.00801353	Down	27400	hydroxysteroid (17-beta) dehydrogenase 6 [Source:MGI Symbol;Acc:MGI:1351670]	Hsd17b6	protein_coding
ENSMUSG00000052302	171.36613	1.09279941	0.33293491	3.73E-05	0.00801353	Up	74694	TBC1 domain family, member 30 [Source:MGI Symbol;Acc:MGI:1921944]	Tbc1d30	protein_coding
ENSMUSG00000074183	14.0244918	-0.044509	0.0959285	3.75E-05	0.00801353	Down	14857	glutathione S-transferase, alpha 1 (Ya) [Source:MGI Symbol;Acc:MGI:1095417]	Gsta1	protein_coding
ENSMUSG00000021340	3781.59737	0.28734765	0.08287439	3.48E-05	0.00784541	Up	14756	glycosylphosphatidylinositol specific phospholipase D1 [Source:MGI Symbol;Acc:MGI:106604]	Gpld1	protein_coding
ENSMUSG00000023045	625.07241	-0.3698697	0.10764049	3.30E-05	0.00769417	Down	223920	sterol O-acyltransferase 2 [Source:MGI Symbol;Acc:MGI:1332226]	Soat2	protein_coding
ENSMUSG00000039481	26.6578852	-1.128911	0.3386728	3.35E-05	0.00769417	Down	18188	neurturin [Source:MGI Symbol;Acc:MGI:108417]	Nrtn	protein_coding
ENSMUSG00000115431	162.912482	-0.8312709	0.25124316	3.22E-05	0.00768107	Down	NA	predicted pseudogene 3219 [Source:MGI Symbol;Acc:MGI:3781398]	Gm3219	processed_pseudogene
ENSMUSG00000027322	104.688684	0.81797271	0.24352422	2.95E-05	0.00717499	Up	20612	sialic acid binding Ig-like lectin 1, sialoadhesin [Source:MGI Symbol;Acc:MGI:99668]	Siglec1	protein_coding
ENSMUSG00000029311	2805.28762	-0.4239412	0.12400275	2.84E-05	0.00704381	Down	114664	hydroxysteroid (17-beta) dehydrogenase 11 [Source:MGI Symbol;Acc:MGI:2149821]	Hsd17b11	protein_coding
ENSMUSG00000035413	198.779883	-0.5578948	0.16145535	2.58E-05	0.00652665	Down	103743	transmembrane protein 98 [Source:MGI Symbol;Acc:MGI:1923457]	Tmem98	protein_coding
ENSMUSG00000061780	41.5587177	-0.0156622	0.07722881	2.50E-05	0.00644622	Down	11537	complement factor D (adipsin) [Source:MGI Symbol;Acc:MGI:87931]	Cfd	protein_coding
ENSMUSG00000071547	77.4703159	-0.6220427	0.17988625	2.28E-05	0.00602082	Down	70021	5'-nucleotidase domain containing 2 [Source:MGI Symbol;Acc:MGI:1917271]	Nt5dc2	protein_coding
ENSMUSG00000070407	1954.46817	0.55145373	0.15549493	1.85E-05	0.00499343	Up	54710	heparan sulfate (glucosamine) 3-O-sulfotransferase 3B1 [Source:MGI Symbol;Acc:MGI:1333853]	Hs3st3b1	protein_coding
ENSMUSG00000034744	187.261824	-0.4141006	0.1166498	1.66E-05	0.00461707	Down	56174	N-acetylglucosamine kinase [Source:MGI Symbol;Acc:MGI:1860418]	Nagk	protein_coding
ENSMUSG00000052133	61.6340217	-1.4630328	0.41782379	1.68E-05	0.00461707	Down	20357	sema domain, seven thrombospondin repeats (type 1 and type 1-like), transmembrane domain (TM) and short cytoplasmic domain, (semaphorin) 5B [Source:MGI Symbol;Acc:MGI:107555]	Sema5b	protein_coding
ENSMUSG00000049721	40.8000776	-1.4533912	0.41017245	1.47E-05	0.00422511	Down	53897	galactose-3-O-sulfotransferase 1 [Source:MGI Symbol;Acc:MGI:1858277]	Gal3st1	protein_coding

ENSMUSG00000014599	159.96888	0.85104915	0.23877654	1.43E-05	0.00421342	Up	12977	colony stimulating factor 1 (macrophage) [Source:MGI Symbol;Acc:MGI:1339753]	Csf1	protein_coding
ENSMUSG00000027999	196.826572	-0.8127922	0.22760937	1.40E-05	0.00421342	Down	66350	phospholipase A2, group XIIA [Source:MGI Symbol;Acc:MGI:1913600]	Pla2g12a	protein_coding
ENSMUSG00000022237	658.185535	0.54480894	0.1495153	1.33E-05	0.00411853	Up	67434	ankyrin repeat domain 33B [Source:MGI Symbol;Acc:MGI:1917904]	Ankrd33b	protein_coding
ENSMUSG00000043801	77.734961	-0.5682126	0.15620913	1.21E-05	0.00383846	Down	NA	ornithine decarboxylase antizyme 1, pseudogene [Source:MGI Symbol;Acc:MGI:108188]	Oaz1-ps	processed_pseudogene
ENSMUSG00000028494	7881.21351	-0.561312	0.15339205	1.16E-05	0.00379884	Down	11520	perilipin 2 [Source:MGI Symbol;Acc:MGI:87920]	Plin2	protein_coding
ENSMUSG00000026880	1061.40003	-0.4414628	0.1199263	1.08E-05	0.00360419	Down	13830	stomatin [Source:MGI Symbol;Acc:MGI:95403]	Stom	protein_coding
ENSMUSG00000042978	614.720545	0.84986186	0.23316917	1.07E-05	0.00360419	Up	104175	SH3-binding kinase 1 [Source:MGI Symbol;Acc:MGI:2135937]	Sbk1	protein_coding
ENSMUSG00000091803	77.3456761	1.31003029	0.35997114	1.01E-05	0.00358419	Up	66272	cytochrome c oxidase assembly protein 16 [Source:MGI Symbol;Acc:MGI:1913522]	Cox16	protein_coding
ENSMUSG00000007837	557.521813	-0.2727088	0.07108557	9.10E-06	0.00331684	Down	65116	proline-rich Gla (G-carboxyglutamic acid) polypeptide 2 [Source:MGI Symbol;Acc:MGI:1929596]	Prrg2	protein_coding
ENSMUSG00000032763	444.941119	-0.3818695	0.10083868	7.55E-06	0.00283728	Down	216136	ilvB (bacterial acetolactate synthase)-like [Source:MGI Symbol;Acc:MGI:1351911]	Ilvbl	protein_coding
ENSMUSG00000024219	420.684716	0.51802463	0.13713451	6.57E-06	0.0025463	Up	224650	ankyrin repeat and SAM domain containing 1 [Source:MGI Symbol;Acc:MGI:2446180]	Anks1	protein_coding
ENSMUSG00000045438	586.496018	-0.3558926	0.09173351	6.07E-06	0.00242809	Down	68033	cytochrome c oxidase assembly protein 19 [Source:MGI Symbol;Acc:MGI:1915283]	Cox19	protein_coding
ENSMUSG00000016756	3637.47785	0.36887292	0.09369783	4.75E-06	0.00196447	Up	12763	cytidine monophospho-N-acetylneuraminic acid hydroxylase [Source:MGI Symbol;Acc:MGI:103227]	Cmah	protein_coding
ENSMUSG00000053898	4311.92562	-0.6303427	0.16195488	4.55E-06	0.00194326	Down	51798	enoyl coenzyme A hydratase 1, peroxisomal [Source:MGI Symbol;Acc:MGI:1858208]	Ech1	protein_coding
ENSMUSG00000026348	446.401397	1.08839474	0.27962193	3.86E-06	0.00170982	Up	266645	amino carboxymuconate semialdehyde decarboxylase [Source:MGI Symbol;Acc:MGI:2386323]	Acmsd	protein_coding
ENSMUSG00000030102	854.537447	0.52538314	0.13395896	3.76E-06	0.00170982	Up	16438	inositol 1,4,5-trisphosphate receptor 1 [Source:MGI Symbol;Acc:MGI:96623]	Itpr1	protein_coding
ENSMUSG00000025991	69913.2943	0.49861053	0.12455483	3.03E-06	0.00154528	Up	227231	carbamoyl-phosphate synthetase 1 [Source:MGI Symbol;Acc:MGI:891996]	Cps1	protein_coding
ENSMUSG00000056133	43.8287117	-1.0914172	0.27590732	3.14E-06	0.00154528	Down	667055	unc-93 homolog A2 [Source:MGI Symbol;Acc:MGI:3712668]	Unc93a2	protein_coding
ENSMUSG00000058546	924.255375	0.77329356	0.19569438	3.24E-06	0.00154528	Up	108168140	ribosomal protein L23A [Source:MGI Symbol;Acc:MGI:3040672]	Rpl23a	protein_coding
ENSMUSG00000063704	258.002006	-0.9629648	0.24286593	3.04E-06	0.00154528	Down	332110	mitogen-activated protein kinase 15 [Source:MGI Symbol;Acc:MGI:2652894]	Mapk15	protein_coding
ENSMUSG00000026688	221.952921	-0.9190394	0.2295098	2.63E-06	0.00147987	Down	66447	microsomal glutathione S-transferase 3 [Source:MGI Symbol;Acc:MGI:1913697]	Mgst3	protein_coding
ENSMUSG00000035836	8730.33002	0.51434848	0.12617103	2.56E-06	0.00147987	Up	71773	UDP glucuronosyltransferase 2 family, polypeptide B1 [Source:MGI Symbol;Acc:MGI:1919023]	Ugt2b1	protein_coding
ENSMUSG00000028715	2818.60987	-0.0111533	0.0760343	1.71E-06	0.00106051	Down	13119	cytochrome P450, family 4, subfamily a, polypeptide 14 [Source:MGI Symbol;Acc:MGI:1096550]	Cyp4a14	protein_coding
ENSMUSG00000010175	1357.64854	0.92984588	0.21959962	9.68E-07	0.00063175	Up	19130	prospero homeobox 1 [Source:MGI Symbol;Acc:MGI:97772]	Prox1	protein_coding
ENSMUSG00000010608	648.179958	0.34473177	0.07943911	8.88E-07	0.00063175	Up	67039	RNA binding motif protein 25 [Source:MGI Symbol;Acc:MGI:1914289]	Rbm25	protein_coding
ENSMUSG00000032788	547.473353	0.6188908	0.14520416	9.43E-07	0.00063175	Up	216134	pyridoxal (pyridoxine, vitamin B6) kinase [Source:MGI Symbol;Acc:MGI:1351869]	Pdxk	protein_coding
ENSMUSG00000039960	2239.82465	-0.3495233	0.08080984	7.98E-07	0.00061869	Down	69581	ras homolog family member U [Source:MGI Symbol;Acc:MGI:1916831]	Rhou	protein_coding
ENSMUSG00000096674	1234.5629	3.09303954	0.72188251	6.59E-07	0.00054497	Up	100039150	major urinary protein 15 [Source:MGI Symbol;Acc:MGI:3780235]	Mup15	protein_coding
ENSMUSG00000031271	835.209894	-1.2072916	0.27774206	4.96E-07	0.00045098	Down	331535	serine (or cysteine) peptidase inhibitor, clade A (alpha-1 antiproteinase, antitrypsin), member 7 [Source:MGI Symbol;Acc:MGI:3041197]	Serpina7	protein_coding
ENSMUSG00000063065	643.096144	-0.3394397	0.07694391	5.09E-07	0.00045098	Down	26417	mitogen-activated protein kinase 3 [Source:MGI Symbol;Acc:MGI:1346859]	Mapk3	protein_coding
ENSMUSG00000023232	270.669307	-0.6912294	0.15370694	3.14E-07	0.0003246	Down	230779	serine incorporator 2 [Source:MGI Symbol;Acc:MGI:1919132]	Serinc2	protein_coding
ENSMUSG00000041930	85.0537893	1.4513193	0.32325684	2.94E-07	0.0003246	Up	433940	family with sequence similarity 222, member A [Source:MGI Symbol;Acc:MGI:3605543]	Fam222a	protein_coding
ENSMUSG00000027997	322.918941	-0.6663609	0.1437386	1.64E-07	0.00020297	Down	12368	caspase 6 [Source:MGI Symbol;Acc:MGI:1312921]	Casp6	protein_coding
ENSMUSG00000084309	38.1094769	2.26082758	0.4797291	9.75E-08	0.00013434	Up	NA	major urinary protein, pseudogene 20 [Source:MGI Symbol;Acc:MGI:3651976]	Mup-ps20	unprocessed_pseudogene
ENSMUSG00000034575	364.591589	0.72641759	0.15125925	7.17E-08	0.00011119	Up	210106	terminal nucleotidyltransferase 4A [Source:MGI Symbol;Acc:MGI:2682295]	Tent4a	protein_coding
ENSMUSG00000038949	184.814559	0.76858995	0.15899563	5.99E-08	0.00011119	Up	226744	consortin, connexin sorting protein [Source:MGI Symbol;Acc:MGI:2445141]	Cnst	protein_coding
ENSMUSG00000091498	271.02325	-0.8665954	0.1803633	6.51E-08	0.00011119	Down	NA	mitochondrial pyruvate carrier 1, pseudogene [Source:MGI Symbol;Acc:MGI:3781628]	Mpc1-ps	processed_pseudogene
ENSMUSG00000030498	787.554128	-0.5509679	0.1101795	3.02E-08	9.37E-05	Down	14453	growth arrest specific 2 [Source:MGI Symbol;Acc:MGI:95657]	Gas2	protein_coding
ENSMUSG00000022708	1393.42617	0.95174704	0.17986569	5.50E-09	2.27E-05	Up	56490	zinc finger and BTB domain containing 20 [Source:MGI Symbol;Acc:MGI:1929213]	Zbtb20	protein_coding
ENSMUSG00000064372	896.08151	1.46636521	0.24351666	7.62E-11	4.72E-07	Up	NA	mitochondrially encoded tRNA proline [Source:MGI Symbol;Acc:MGI:102478]	mt-Tp	Mt_tRNA
ENSMUSG00000046876	354.658888	1.48363416	0.16870165	7.05E-20	8.74E-16	Up	20238	ataxin 1 [Source:MGI Symbol;Acc:MGI:104783]	Atxn1	protein_coding
