# Supplementary material for: The effect of aldafermin expressing-Escherichia coli Nissle 1917 along with dietary change on visceral adipose tissue in MASLD mouse model
Source: Int J Obes (Lond). 2025 Apr 10;49(7):1334–44. doi: 10.1038/s41366-025-01774-w (PMC12283412; doi:10.1038/s41366-025-01774-w)
Supplement: Supplementary file 6 — Supplementary table 7 [file 41366_2025_1774_MOESM6_ESM.pdf]

Supplementary table 7. DEGs observed in liver when EcNA was compared to EcN

EcNA vs EcN liver

ENSEMBL	baseMean	log2FoldChange	lfcSE	pvalue	padj	UD	entrezgene_id	description	external_gene_name	gene_biotype
ENSMUSG00000000440	192.896436	-0.9059357	0.36606312	0.00039652	0.0402601	Down	19016	peroxisome proliferator activated receptor gamma [Source:MGI Symbol;Acc:MGI:97747]	Pparg	protein_coding
ENSMUSG00000001642	152.660098	0.5314078	0.20850809	0.00038206	0.03938906	Up	11677	aldo-keto reductase family 1, member B3 (aldose reductase) [Source:MGI Symbol;Acc:MGI:1353494]	Akr1b3	protein_coding
ENSMUSG00000001665	800.692544	-0.6183332	0.25686224	0.0005058	0.04539172	Down	103140	glutathione S-transferase, theta 3 [Source:MGI Symbol;Acc:MGI:2143526]	Gstt3	protein_coding
ENSMUSG00000003053	23179.3247	-0.5722383	0.14929285	5.54E-06	0.00441512	Down	13095	cytochrome P450, family 2, subfamily c, polypeptide 29 [Source:MGI Symbol;Acc:MGI:103238]	Cyp2c29	protein_coding
ENSMUSG00000003355	218.562018	1.10734939	0.28400189	3.76E-06	0.00402222	Up	66120	FK506 binding protein 11 [Source:MGI Symbol;Acc:MGI:1913370]	Fkbp11	protein_coding
ENSMUSG00000004460	1994.87988	0.5417801	0.18222154	0.00011822	0.02037296	Up	67838	DnaJ heat shock protein family (Hsp40) member B11 [Source:MGI Symbol;Acc:MGI:1915088]	Dnajb11	protein_coding
ENSMUSG00000006442	597.05183	0.57047594	0.22205365	0.000378	0.03938906	Up	20810	spermidine synthase [Source:MGI Symbol;Acc:MGI:102690]	Srm	protein_coding
ENSMUSG00000007594	300.563618	0.90814015	0.22211338	1.74E-06	0.00229421	Up	330790	hyaluronan and proteoglycan link protein 4 [Source:MGI Symbol;Acc:MGI:2679531]	Hapln4	protein_coding
ENSMUSG00000008153	476.143088	-1.2622446	0.49753567	0.000317	0.03660133	Down	232370	calsyntenin 3 [Source:MGI Symbol;Acc:MGI:2178323]	Clstn3	protein_coding
ENSMUSG00000008734	26.2851085	-0.0199099	0.07635963	1.37E-05	0.00681899	Down	64297	G protein-coupled receptor, family C, group 5, member B [Source:MGI Symbol;Acc:MGI:1927596]	Gprc5b	protein_coding
ENSMUSG00000010175	1357.64854	0.83703113	0.22245788	6.59E-06	0.00458632	Up	19130	prospero homeobox 1 [Source:MGI Symbol;Acc:MGI:97772]	Prox1	protein_coding
ENSMUSG00000010651	22415.3063	-1.0605691	0.42803451	0.00037713	0.03938906	Down	235674	acetyl-Coenzyme A acyltransferase 1B [Source:MGI Symbol;Acc:MGI:3605455]	Acaa1b	protein_coding
ENSMUSG00000011257	738.371292	0.41024469	0.0904587	2.94E-07	0.0008172	Up	230721	poly(A) binding protein, cytoplasmic 4 [Source:MGI Symbol;Acc:MGI:2385206]	Pabpc4	protein_coding
ENSMUSG00000012187	35.3603701	-0.0259196	0.07922002	7.24E-05	0.0161992	Down	68393	monoacylglycerol O-acyltransferase 1 [Source:MGI Symbol;Acc:MGI:1915643]	Mogat1	protein_coding
ENSMUSG00000013629	324.884253	0.83260355	0.25107709	3.55E-05	0.01122101	Up	69719	carbamoyl-phosphate synthetase 2, aspartate transcarbamylase, and dihydroorotase [Source:MGI Symbol;Acc:MGI:1916969]	Cad	protein_coding
ENSMUSG00000014599	159.96888	0.74617016	0.24430669	8.30E-05	0.01724108	Up	12977	colony stimulating factor 1 (macrophage) [Source:MGI Symbol;Acc:MGI:1339753]	Csf1	protein_coding
ENSMUSG00000015488	604.011953	0.35329035	0.13763571	0.00046433	0.04334747	Up	381356	calcium channel flower domain containing 1 [Source:MGI Symbol;Acc:MGI:1924317]	Cacfd1	protein_coding
ENSMUSG00000019768	262.685638	0.6567964	0.24223183	0.00023608	0.03188262	Up	13982	estrogen receptor 1 (alpha) [Source:MGI Symbol;Acc:MGI:1352467]	Esr1	protein_coding
ENSMUSG00000019851	1937.13984	-0.244102	0.08524655	0.00030542	0.03660133	Down	64058	PERP, TP53 apoptosis effector [Source:MGI Symbol;Acc:MGI:1929938]	Perp	protein_coding
ENSMUSG00000020019	152.87275	-0.4657515	0.18071177	0.00037638	0.03938906	Down	57764	netrin 4 [Source:MGI Symbol;Acc:MGI:1888978]	Ntn4	protein_coding
ENSMUSG00000020173	387.262686	0.6298169	0.2563703	0.00043975	0.04226995	Up	12808	cordon-bleu WH2 repeat [Source:MGI Symbol;Acc:MGI:105056]	Cobl	protein_coding
ENSMUSG00000020218	6.00123192	2.19858167	0.78257809	0.00019377	0.02778625	Up	24117	Wnt inhibitory factor 1 [Source:MGI Symbol;Acc:MGI:1344332]	Wif1	protein_coding
ENSMUSG00000020340	23.2000393	-0.8368492	0.34255538	0.00047531	0.04407711	Down	76884	cytoplasmic FMR1 interacting protein 2 [Source:MGI Symbol;Acc:MGI:1924134]	Cyfip2	protein_coding
ENSMUSG00000020553	1484.07546	-0.676053	0.23445486	0.00013626	0.02256355	Down	18559	phosphatidylcholine transfer protein [Source:MGI Symbol;Acc:MGI:107375]	Pctp	protein_coding
ENSMUSG00000020571	5835.04413	0.42812269	0.16684341	0.00043699	0.04226995	Up	71853	protein disulfide isomerase associated 6 [Source:MGI Symbol;Acc:MGI:1919103]	Pdia6	protein_coding
ENSMUSG00000020642	77.1397224	-0.9401606	0.34767184	0.00022573	0.03078272	Down	108089	ring finger protein 144A [Source:MGI Symbol;Acc:MGI:1344401]	Rnf144a	protein_coding
ENSMUSG00000020672	120.963223	0.89844999	0.31360578	0.00013991	0.02289565	Up	268534	syntrophin, gamma 2 [Source:MGI Symbol;Acc:MGI:1919541]	Sntg2	protein_coding
ENSMUSG00000020865	3555.63105	-1.1930351	0.32646856	1.00E-05	0.00581548	Down	76408	ATP-binding cassette, sub-family C (CFTR/MRP), member 3 [Source:MGI Symbol;Acc:MGI:1923658]	Abcc3	protein_coding
ENSMUSG00000021236	4462.61757	-0.5691899	0.20816441	0.00022539	0.03078272	Down	12499	ectonucleoside triphosphate diphosphohydrolase 5 [Source:MGI Symbol;Acc:MGI:1321385]	Entpd5	protein_coding
ENSMUSG00000021259	62.8426129	-0.0362183	0.08605032	0.00040599	0.04062823	Down	13116	cytochrome P450, family 46, subfamily a, polypeptide 1 [Source:MGI Symbol;Acc:MGI:1341877]	Cyp46a1	protein_coding
ENSMUSG00000021287	57.8908072	-0.7260996	0.28042725	0.00031744	0.03660133	Down	74335	X-ray repair complementing defective repair in Chinese hamster cells 3 [Source:MGI Symbol;Acc:MGI:1921585]	Xrcc3	protein_coding
ENSMUSG00000021360	363.556933	0.58792034	0.12806708	2.22E-07	0.00077339	Up	14538	glucosaminyl (N-acetyl) transferase 2, I-branching enzyme [Source:MGI Symbol;Acc:MGI:1100870]	Gcnt2	protein_coding
ENSMUSG00000021610	1654.48464	0.21980326	0.08055833	0.00051782	0.04617226	Up	218335	CLPTM1-like [Source:MGI Symbol;Acc:MGI:2442892]	Clptm1l	protein_coding
ENSMUSG00000022136	5199.47275	0.43215755	0.1485393	0.00016068	0.02511218	Up	100037258	DnaJ heat shock protein family (Hsp40) member C3 [Source:MGI Symbol;Acc:MGI:107373]	Dnajc3	protein_coding
ENSMUSG00000022210	2062.61996	-0.2691609	0.10012269	0.00039053	0.03994285	Down	28200	dehydrogenase/reductase (SDR family) member 4 [Source:MGI Symbol;Acc:MGI:90169]	Dhrs4	protein_coding
ENSMUSG00000022215	901.91891	-0.8764302	0.2718543	4.30E-05	0.01211996	Down	68680	fat storage-inducing transmembrane protein 1 [Source:MGI Symbol;Acc:MGI:1915930]	Fitm1	protein_coding
ENSMUSG00000022304	3760.24093	-0.368703	0.14245706	0.00040257	0.04057791	Down	64705	dihydropyrimidinase [Source:MGI Symbol;Acc:MGI:1928679]	Dpys	protein_coding
ENSMUSG00000022391	1028.36632	0.35108004	0.12097727	0.00018286	0.02677499	Up	19387	RAN GTPase activating protein 1 [Source:MGI Symbol;Acc:MGI:103071]	Rangap1	protein_coding
ENSMUSG00000022474	62.5500881	-0.6769052	0.28310854	0.00052834	0.04664861	Down	29858	phosphomannomutase 1 [Source:MGI Symbol;Acc:MGI:1353418]	Pmm1	protein_coding
ENSMUSG00000022508	738.680018	1.72543035	0.66927091	0.00027961	0.035039	Up	12053	B cell leukemia/lymphoma 6 [Source:MGI Symbol;Acc:MGI:107187]	Bcl6	protein_coding
ENSMUSG00000022708	1393.42617	0.71017155	0.18488018	5.50E-06	0.00441512	Up	56490	zinc finger and BTB domain containing 20 [Source:MGI Symbol;Acc:MGI:1929213]	Zbtb20	protein_coding
ENSMUSG00000022844	1115.66778	0.69203862	0.19873718	2.03E-05	0.00872661	Up	72599	protein disulfide isomerase associated 5 [Source:MGI Symbol;Acc:MGI:1919849]	Pdia5	protein_coding
ENSMUSG00000023044	3742.09888	-1.259122	0.39165755	4.25E-05	0.01211996	Down	246277	cysteine sulfinic acid decarboxylase [Source:MGI Symbol;Acc:MGI:2180098]	Csad	protein_coding
ENSMUSG00000023393	103.449144	0.68207204	0.22392693	8.71E-05	0.01764312	Up	228993	solute carrier family 17, member 9 [Source:MGI Symbol;Acc:MGI:1919107]	Slc17a9	protein_coding
ENSMUSG00000023961	234.269977	0.29057984	0.11399774	0.00057434	0.04816677	Up	224794	ectonucleotide pyrophosphatase/phosphodiesterase 4 [Source:MGI Symbol;Acc:MGI:2682634]	Enpp4	protein_coding
ENSMUSG00000024026	6601.03024	-0.2608203	0.09618986	0.00042331	0.04205929	Down	109801	glyoxalase 1 [Source:MGI Symbol;Acc:MGI:95742]	Glo1	protein_coding
ENSMUSG00000024371	558.186663	0.4970054	0.20749704	0.00057537	0.04816677	Up	12263	complement component 2 (within H-2S) [Source:MGI Symbol;Acc:MGI:88226]	C2	protein_coding
ENSMUSG00000024526	138.180413	-0.0106481	0.07340904	0.00014452	0.02310593	Down	12683	cell death-inducing DNA fragmentation factor, alpha subunit-like effector A [Source:MGI Symbol;Acc:MGI:1270845]	Cidea	protein_coding
ENSMUSG00000024924	31.7512168	-0.0295417	0.08142859	3.38E-05	0.01112898	Down	22359	very low density lipoprotein receptor [Source:MGI Symbol;Acc:MGI:98935]	Vldlr	protein_coding
ENSMUSG00000025512	605.364877	0.39906504	0.16193444	0.00054769	0.04761469	Up	68038	chitinase domain containing 1 [Source:MGI Symbol;Acc:MGI:1915288]	Chid1	protein_coding

ENSMUSG00000025792	4681.43844	-0.6387003	0.20239104	7.24E-05	0.0161992	Down	27376	solute carrier family 25 (mitochondrial carrier, dicarboxylate transporter), member 10 [Source:MGI Symbol;Acc:MGI:1353497]	Slc25a10	protein_coding
ENSMUSG00000025935	3055.2371	0.32225454	0.08625411	1.26E-05	0.0064728	Up	72265	translocating chain-associating membrane protein 1 [Source:MGI Symbol;Acc:MGI:1919515]	Tram1	protein_coding
ENSMUSG00000026131	1423.91856	0.38888377	0.14931201	0.00043469	0.04226995	Up	13518	dystonin [Source:MGI Symbol;Acc:MGI:104627]	Dst	protein_coding
ENSMUSG00000026249	350.928538	0.93603316	0.28257744	3.22E-05	0.01112898	Up	20720	serine (or cysteine) peptidase inhibitor, clade E, member 2 [Source:MGI Symbol;Acc:MGI:101780]	Serpine2	protein_coding
ENSMUSG00000026348	446.401397	0.97636193	0.2841113	2.23E-05	0.00884989	Up	266645	amino carboxymuconate semialdehyde decarboxylase [Source:MGI Symbol;Acc:MGI:2386323]	Acmsd	protein_coding
ENSMUSG00000026615	1889.16748	0.23823973	0.07533231	0.00011864	0.02037296	Up	107508	glutamyl-prolyl-tRNA synthetase [Source:MGI Symbol;Acc:MGI:97838]	Eprs	protein_coding
ENSMUSG00000026688	221.952921	-1.0352794	0.22676647	2.09E-07	0.00077339	Down	66447	microsomal glutathione S-transferase 3 [Source:MGI Symbol;Acc:MGI:1913697]	Mgst3	protein_coding
ENSMUSG00000026853	749.413394	-0.0558996	0.10818713	0.00057951	0.04816677	Down	12908	carnitine acetyltransferase [Source:MGI Symbol;Acc:MGI:109501]	Crat	protein_coding
ENSMUSG00000026880	1061.40003	-0.3079767	0.12640598	0.00050062	0.04521837	Down	13830	stomatin [Source:MGI Symbol;Acc:MGI:95403]	Stom	protein_coding
ENSMUSG00000027079	236.612546	0.58516493	0.22355663	0.00031839	0.03660133	Up	98985	CLP1, cleavage and polyadenylation factor I subunit [Source:MGI Symbol;Acc:MGI:2138968]	Clp1	protein_coding
ENSMUSG00000027439	703.722024	0.25220243	0.09509525	0.0005453	0.04761469	Up	74533	GDNF-inducible zinc finger protein 1 [Source:MGI Symbol;Acc:MGI:1921783]	Gzf1	protein_coding
ENSMUSG00000027502	1250.49551	-0.3019969	0.11484341	0.00044244	0.04226995	Down	66404	replication termination factor 2 [Source:MGI Symbol;Acc:MGI:1913654]	Rtf2	protein_coding
ENSMUSG00000027602	753.835534	-0.3135614	0.10151637	9.85E-05	0.01826454	Down	66734	microtubule-associated protein 1 light chain 3 alpha [Source:MGI Symbol;Acc:MGI:1915661]	Map1lc3a	protein_coding
ENSMUSG00000027642	3417.57524	0.31547565	0.07491198	1.69E-06	0.00229421	Up	20014	ribophorin II [Source:MGI Symbol;Acc:MGI:98085]	Rpn2	protein_coding
ENSMUSG00000027800	183.321068	-0.6762252	0.20184728	3.15E-05	0.01112898	Down	17112	transmembrane 4 superfamily member 1 [Source:MGI Symbol;Acc:MGI:104678]	Tm4sf1	protein_coding
ENSMUSG00000027808	6041.9516	0.42420881	0.12288181	3.40E-05	0.01112898	Up	28146	stress-associated endoplasmic reticulum protein 1 [Source:MGI Symbol;Acc:MGI:92638]	Serp1	protein_coding
ENSMUSG00000027828	4767.43306	0.33051959	0.10099044	5.68E-05	0.01463335	Up	67437	signal sequence receptor, gamma [Source:MGI Symbol;Acc:MGI:1914687]	Ssr3	protein_coding
ENSMUSG00000028076	3335.04799	-0.3289215	0.10139255	5.61E-05	0.01463335	Down	12479	CD1d1 antigen [Source:MGI Symbol;Acc:MGI:107674]	Cd1d1	protein_coding
ENSMUSG00000028672	4452.557	-0.4112368	0.16310561	0.00058174	0.04816677	Down	15356	3-hydroxy-3-methylglutaryl-Coenzyme A lyase [Source:MGI Symbol;Acc:MGI:96158]	Hmgcl	protein_coding
ENSMUSG00000028715	2818.60987	-0.0103919	0.07336985	1.98E-06	0.00229421	Down	13119	cytochrome P450, family 4, subfamily a, polypeptide 14 [Source:MGI Symbol;Acc:MGI:1096550]	Cyp4a14	protein_coding
ENSMUSG00000028757	2195.12917	0.39514747	0.11036486	1.96E-05	0.00872661	Up	13200	dolichyl-di-phosphooligosaccharide-protein glycotransferase [Source:MGI Symbol;Acc:MGI:1194508]	Ddost	protein_coding
ENSMUSG00000028970	27.086897	1.05728502	0.4217115	0.00037818	0.03938906	Up	18669	ATP-binding cassette, sub-family B (MDR/TAP), member 1B [Source:MGI Symbol;Acc:MGI:97568]	Abcb1b	protein_coding
ENSMUSG00000028999	310.906335	0.53849632	0.19952776	0.00025621	0.03329024	Up	72772	RAD50 interactor 1 [Source:MGI Symbol;Acc:MGI:1916233]	Rint1	protein_coding
ENSMUSG00000029201	2461.50827	-0.3731373	0.13894636	0.00031659	0.03660133	Down	22235	UDP-glucose dehydrogenase [Source:MGI Symbol;Acc:MGI:1306785]	Ugdh	protein_coding
ENSMUSG00000029380	515.865416	3.25446874	1.00692069	3.43E-05	0.01112898	Up	14825	chemokine (C-X-C motif) ligand 1 [Source:MGI Symbol;Acc:MGI:108068]	Cxcl1	protein_coding
ENSMUSG00000029767	1706.6445	0.26075562	0.07805065	6.80E-05	0.0161992	Up	12321	calumenin [Source:MGI Symbol;Acc:MGI:1097158]	Calu	protein_coding
ENSMUSG00000029772	389.685595	0.66177134	0.1909216	2.13E-05	0.00872661	Up	74340	S-adenosylhomocysteine hydrolase-like 2 [Source:MGI Symbol;Acc:MGI:1921590]	Ahcyl2	protein_coding
ENSMUSG00000030082	2919.73631	0.42897564	0.11627728	1.15E-05	0.00638853	Up	53421	Sec61 alpha 1 subunit (S. cerevisiae) [Source:MGI Symbol;Acc:MGI:1858417]	Sec61a1	protein_coding
ENSMUSG00000030278	147.665025	-0.0291222	0.08112885	7.12E-05	0.0161992	Down	14311	cell death-inducing DFFA-like effector c [Source:MGI Symbol;Acc:MGI:95585]	Cidec	protein_coding
ENSMUSG00000030470	288.295763	-0.5272604	0.17730566	0.00010738	0.01914891	Down	13009	cysteine and glycine-rich protein 3 [Source:MGI Symbol;Acc:MGI:1330824]	Csrp3	protein_coding
ENSMUSG00000030483	109.8738	-0.0222544	0.07732766	0.0001756	0.02633108	Down	13088	cytochrome P450, family 2, subfamily b, polypeptide 10 [Source:MGI Symbol;Acc:MGI:88598]	Cyp2b10	protein_coding
ENSMUSG00000030498	787.554128	-0.4810837	0.11161046	1.05E-06	0.00208257	Down	14453	growth arrest specific 2 [Source:MGI Symbol;Acc:MGI:95657]	Gas2	protein_coding
ENSMUSG00000030545	1258.27645	-0.7064738	0.21272709	3.44E-05	0.01112898	Down	18631	peroxisomal biogenesis factor 11 alpha [Source:MGI Symbol;Acc:MGI:1338788]	Pex11a	protein_coding
ENSMUSG00000030659	43.0267229	1.41113903	0.50304568	0.00015883	0.02510626	Up	53322	nucleobindin 2 [Source:MGI Symbol;Acc:MGI:1858179]	Nucb2	protein_coding
ENSMUSG00000031271	835.209895	-1.1693583	0.27861299	1.00E-06	0.00208257	Down	331535	serine (or cysteine) peptidase inhibitor, clade A (alpha-1 antiproteinase, antitrypsin), member 7 [Source:MGI Symbol;Acc:MGI:3041197]	Serpina7	protein_coding
ENSMUSG00000031808	115.40815	-0.5281743	0.19410715	0.00024477	0.03273847	Down	26457	solute carrier family 27 (fatty acid transporter), member 1 [Source:MGI Symbol;Acc:MGI:1347098]	Slc27a1	protein_coding
ENSMUSG00000031924	11170.2965	-0.3400815	0.12613135	0.00032883	0.03717816	Down	66427	cytochrome b5 type B [Source:MGI Symbol;Acc:MGI:1913677]	Cyb5b	protein_coding
ENSMUSG00000032042	3985.94114	0.29165866	0.08158634	2.31E-05	0.00894205	Up	67398	signal recognition particle receptor ('docking protein') [Source:MGI Symbol;Acc:MGI:1914648]	Srpr	protein_coding
ENSMUSG00000032116	1963.88416	0.34683556	0.08975156	6.46E-06	0.00458632	Up	16430	STT3, subunit of the oligosaccharyltransferase complex, homolog A (S. cerevisiae) [Source:MGI Symbol;Acc:MGI:105124]	Stt3a	protein_coding
ENSMUSG00000032285	50.3652057	0.95694222	0.30838277	6.85E-05	0.0161992	Up	58233	DnaJ heat shock protein family (Hsp40) member A4 [Source:MGI Symbol;Acc:MGI:1927638]	Dnaja4	protein_coding
ENSMUSG00000032350	5254.27119	-0.5259442	0.17353138	9.71E-05	0.01825233	Down	14629	glutamate-cysteine ligase, catalytic subunit [Source:MGI Symbol;Acc:MGI:104990]	Gclc	protein_coding
ENSMUSG00000032557	625.985694	0.39493339	0.13872946	0.00019756	0.02792707	Up	66663	ubiquitin-like modifier activating enzyme 5 [Source:MGI Symbol;Acc:MGI:1913913]	Uba5	protein_coding
ENSMUSG00000032602	1663.55182	-0.3757667	0.13595309	0.00025404	0.03329024	Down	57279	solute carrier family 25 (mitochondrial carnitine/acylcarnitine translocase), member 20 [Source:MGI Symbol;Acc:MGI:1928738]	Slc25a20	protein_coding
ENSMUSG00000032604	807.978803	0.20845173	0.07198424	0.00033142	0.03717816	Up	97541	glutaminyl-tRNA synthetase [Source:MGI Symbol;Acc:MGI:1915851]	Qars	protein_coding
ENSMUSG00000032808	656.649033	-1.360681	0.53671708	0.00031387	0.03660133	Down	13097	cytochrome P450, family 2, subfamily c, polypeptide 38 [Source:MGI Symbol;Acc:MGI:1306819]	Cyp2c38	protein_coding
ENSMUSG00000034088	8671.89532	0.30361981	0.08137567	1.23E-05	0.0064728	Up	110611	high density lipoprotein (HDL) binding protein [Source:MGI Symbol;Acc:MGI:99256]	Hdlbp	protein_coding
ENSMUSG00000034575	364.591589	0.46550369	0.15910009	0.00014392	0.02310593	Up	210106	terminal nucleotidyltransferase 4A [Source:MGI Symbol;Acc:MGI:2682295]	Tent4a	protein_coding
ENSMUSG00000035357	64.3862076	-0.7741359	0.26542189	0.00012616	0.02114359	Down	55983	PDZ domain containing RING finger 3 [Source:MGI Symbol;Acc:MGI:1933157]	Pdzrn3	protein_coding
ENSMUSG00000035413	198.779883	-0.6094077	0.16004176	5.71E-06	0.00441512	Down	103743	transmembrane protein 98 [Source:MGI Symbol;Acc:MGI:1923457]	Tmem98	protein_coding
ENSMUSG00000035441	361.136903	-0.4134805	0.13377898	9.10E-05	0.0178475	Down	338367	myosin ID [Source:MGI Symbol;Acc:MGI:107728]	Myo1d	protein_coding
ENSMUSG00000035910	39.748268	-0.954161	0.36366286	0.0002746	0.03472443	Down	195208	doublecortin domain containing 2a [Source:MGI Symbol;Acc:MGI:2652818]	Dcdc2a	protein_coding

ENSMUSG00000036323	1658.15872	0.23251389	0.07891448	0.00026803	0.03420465	Up	66661	signal recognition particle 72 [Source:MGI Symbol;Acc:MGI:1333795]	Srp72	protein_coding
ENSMUSG00000037211	104.880963	-0.5847051	0.18220788	5.53E-05	0.01463335	Down	24063	sprouty RTK signaling antagonist 1 [Source:MGI Symbol;Acc:MGI:1345139]	Spry1	protein_coding
ENSMUSG00000037440	544.4651	-0.038144	0.08791654	8.75E-05	0.01764312	Down	22361	vanin 1 [Source:MGI Symbol;Acc:MGI:108395]	Vnn1	protein_coding
ENSMUSG00000037470	1608.64723	0.4969791	0.09756859	1.78E-08	0.00012357	Up	320011	UDP-glucose glycoprotein glucosyltransferase 1 [Source:MGI Symbol;Acc:MGI:2443162]	Uggt1	protein_coding
ENSMUSG00000038312	1209.37814	0.40795892	0.09828311	1.82E-06	0.00229421	Up	108687	ER degradation enhancer, mannosidase alpha-like 2 [Source:MGI Symbol;Acc:MGI:1915540]	Edem2	protein_coding
ENSMUSG00000038576	806.685031	0.68431739	0.24053346	0.00016973	0.0259446	Up	96935	sushi domain containing 4 [Source:MGI Symbol;Acc:MGI:2138351]	Susd4	protein_coding
ENSMUSG00000038599	71.7096534	1.92506644	0.59519442	4.02E-05	0.01189969	Up	170725	calpain 8 [Source:MGI Symbol;Acc:MGI:2181366]	Capn8	protein_coding
ENSMUSG00000038776	4691.38936	-0.5563183	0.22310011	0.00043355	0.04226995	Down	13849	epoxide hydrolase 1, microsomal [Source:MGI Symbol;Acc:MGI:95405]	Ephx1	protein_coding
ENSMUSG00000038949	184.814559	0.44616422	0.16970916	0.00034823	0.03875074	Up	226744	consortin, connexin sorting protein [Source:MGI Symbol;Acc:MGI:2445141]	Cnst	protein_coding
ENSMUSG00000039485	28.627981	0.91505052	0.36295255	0.00038228	0.03938906	Up	72480	TSPY-like 4 [Source:MGI Symbol;Acc:MGI:106393]	Tspyl4	protein_coding
ENSMUSG00000039519	4058.42481	0.99428275	0.39414639	0.00033003	0.03717816	Up	13123	cytochrome P450, family 7, subfamily b, polypeptide 1 [Source:MGI Symbol;Acc:MGI:104978]	Cyp7b1	protein_coding
ENSMUSG00000039745	439.097959	-0.3697538	0.14631044	0.00044367	0.04226995	Down	53415	HIV-1 Tat interactive protein 2 [Source:MGI Symbol;Acc:MGI:1859271]	Htatip2	protein_coding
ENSMUSG00000040466	1562.6926	-0.3084787	0.11180057	0.00030565	0.03660133	Down	233016	biliverdin reductase B (flavin reductase (NADPH)) [Source:MGI Symbol;Acc:MGI:2385271]	Blvrb	protein_coding
ENSMUSG00000041044	178.984817	-0.5657839	0.23309077	0.00048107	0.044316	Down	239037	leucine-rich repeat, immunoglobulin-like and transmembrane domains 1 [Source:MGI Symbol;Acc:MGI:2385320]	Lrit1	protein_coding
ENSMUSG00000041891	4569.54319	0.27078024	0.0807945	5.50E-05	0.01463335	Up	70361	lectin, mannose-binding, 1 [Source:MGI Symbol;Acc:MGI:1917611]	Lman1	protein_coding
ENSMUSG00000041895	284.238971	0.34153817	0.1367436	0.00055564	0.04800628	Up	52639	WD repeat domain, phosphoinositide interacting 1 [Source:MGI Symbol;Acc:MGI:1261864]	Wipi1	protein_coding
ENSMUSG00000042747	811.050027	0.42140891	0.13666663	9.48E-05	0.01806545	Up	66059	keratinocyte associated protein 2 [Source:MGI Symbol;Acc:MGI:1913309]	Krtcap2	protein_coding
ENSMUSG00000042978	614.720545	0.64826006	0.24538355	0.00028939	0.03584854	Up	104175	SH3-binding kinase 1 [Source:MGI Symbol;Acc:MGI:2135937]	Sbk1	protein_coding
ENSMUSG00000044071	12.5923409	-0.0380672	0.08804951	8.09E-06	0.00535594	Down	268354	TAFA chemokine like family member 2 [Source:MGI Symbol;Acc:MGI:2143691]	Tafa2	protein_coding
ENSMUSG00000044646	58.0000497	2.00990703	0.60560433	3.03E-05	0.01112898	Up	207259	zinc finger and BTB domain containing 7C [Source:MGI Symbol;Acc:MGI:2443302]	Zbtb7c	protein_coding
ENSMUSG00000044813	1103.97702	0.65483842	0.23656363	0.00018862	0.02733069	Up	230126	src homology 2 domain-containing transforming protein B [Source:MGI Symbol;Acc:MGI:98294]	Shb	protein_coding
ENSMUSG00000045438	586.496018	-0.3303009	0.09273605	2.13E-05	0.00872661	Down	68033	cytochrome c oxidase assembly protein 19 [Source:MGI Symbol;Acc:MGI:1915283]	Cox19	protein_coding
ENSMUSG00000045969	236.198859	0.34718118	0.10363024	4.36E-05	0.01211996	Up	26356	inhibitor of growth family, member 1 [Source:MGI Symbol;Acc:MGI:1349481]	Ing1	protein_coding
ENSMUSG00000046876	354.658888	1.54028987	0.16917255	4.11E-21	5.72E-17	Up	20238	ataxin 1 [Source:MGI Symbol;Acc:MGI:104783]	Atxn1	protein_coding
ENSMUSG00000048486	931.130481	-0.3598584	0.1461769	0.00057196	0.04816677	Down	228859	fat storage-inducing transmembrane protein 2 [Source:MGI Symbol;Acc:MGI:2444508]	Fitm2	protein_coding
ENSMUSG00000049047	77.4427769	0.83686361	0.27350407	8.10E-05	0.01707519	Up	71703	armadillo repeat containing, X-linked 3 [Source:MGI Symbol;Acc:MGI:1918953]	Armcx3	protein_coding
ENSMUSG00000049109	22.5828291	-0.0205907	0.07659372	0.00011661	0.02037296	Down	210757	thymocyte selection associated [Source:MGI Symbol;Acc:MGI:2443552]	Themis	protein_coding
ENSMUSG00000049721	40.8000776	-1.5610551	0.40613847	4.57E-06	0.00423431	Down	53897	galactose-3-O-sulfotransferase 1 [Source:MGI Symbol;Acc:MGI:1858277]	Gal3st1	protein_coding
ENSMUSG00000050097	13.8838595	3.3820504	0.87313373	4.24E-06	0.00421418	Up	234669	carboxyesterase 2B [Source:MGI Symbol;Acc:MGI:2448547]	Ces2b	protein_coding
ENSMUSG00000052133	61.6340217	-0.0727797	0.14555002	7.52E-05	0.0161992	Down	20357	sema domain, seven thrombospondin repeats (type 1 and type 1-like), transmembrane domain (TM) and short cytoplasmic domain, (semaphorin) 5B [Source:MGI Symbol;Acc:MGI:107555]	Sema5b	protein_coding
ENSMUSG00000052271	25.0032774	3.5906757	0.98284613	8.48E-06	0.00536461	Up	17341	basic helix-loop-helix family, member a15 [Source:MGI Symbol;Acc:MGI:891976]	Bhlha15	protein_coding
ENSMUSG00000052302	171.36613	0.9235256	0.34558771	0.00024795	0.03284727	Up	74694	TBC1 domain family, member 30 [Source:MGI Symbol;Acc:MGI:1921944]	Tbc1d30	protein_coding
ENSMUSG00000052544	17.6696741	-0.0973287	0.28097763	0.000179	0.02648821	Down	20447	ST6 (alpha-N-acetyl-neuraminyl-2,3-beta-galactosyl-1,3)-N-acetylgalactosaminide alpha-2,6-sialyltransferase 3 [Source:MGI Symbol;Acc:MGI:1341828]	St6galnac3	protein_coding
ENSMUSG00000052712	1297.29606	-0.3162531	0.11158914	0.00025847	0.03329024	Down	80748	cDNA sequence BC004004 [Source:MGI Symbol;Acc:MGI:2136782]	BC004004	protein_coding
ENSMUSG00000053898	4311.92562	-0.5810488	0.1637352	1.63E-05	0.00780066	Down	51798	enoyl coenzyme A hydratase 1, peroxisomal [Source:MGI Symbol;Acc:MGI:1858208]	Ech1	protein_coding
ENSMUSG00000056035	11688.6833	-0.9938316	0.34240728	0.000121	0.02052505	Down	13112	cytochrome P450, family 3, subfamily a, polypeptide 11 [Source:MGI Symbol;Acc:MGI:88609]	Cyp3a11	protein_coding
ENSMUSG00000056133	43.8287117	-0.7427183	0.30229011	0.00045009	0.04259032	Down	667055	unc-93 homolog A2 [Source:MGI Symbol;Acc:MGI:3712668]	Unc93a2	protein_coding
ENSMUSG00000056973	8395.36178	-0.8543278	0.22974553	9.16E-06	0.00553916	Down	104158	carboxylesterase 1D [Source:MGI Symbol;Acc:MGI:2148202]	Ces1d	protein_coding
ENSMUSG00000057228	1361.39746	0.40832152	0.11554765	2.01E-05	0.00872661	Up	23923	aminoadipate aminotransferase [Source:MGI Symbol;Acc:MGI:1345167]	Aadat	protein_coding
ENSMUSG00000058260	15.0261478	2.13072353	0.5165709	1.66E-06	0.00229421	Up	71907	serine (or cysteine) peptidase inhibitor, clade A (alpha-1 antiproteinase, antitrypsin), member 9 [Source:MGI Symbol;Acc:MGI:1919157]	Serpina9	protein_coding
ENSMUSG00000058569	1373.05242	0.47249715	0.18117444	0.00038011	0.03938906	Up	67511	transmembrane p24 trafficking protein 9 [Source:MGI Symbol;Acc:MGI:1914761]	Tmed9	protein_coding
ENSMUSG00000058794	34.8257304	-0.0346556	0.08477752	0.00057014	0.04816677	Down	18022	nuclear factor, erythroid derived 2 [Source:MGI Symbol;Acc:MGI:97308]	Nfe2	protein_coding
ENSMUSG00000060613	9269.26501	0.74181505	0.24524913	9.11E-05	0.0178475	Up	226105	cytochrome P450, family 2, subfamily c, polypeptide 70 [Source:MGI Symbol;Acc:MGI:2385878]	Cyp2c70	protein_coding
ENSMUSG00000061778	570.71427	-0.3678658	0.12779863	0.00020352	0.02830894	Down	76763	motile sperm domain containing 2 [Source:MGI Symbol;Acc:MGI:1924013]	Mospd2	protein_coding
ENSMUSG00000062116	74.5926895	-0.467414	0.18111092	0.00037896	0.03938906	Down	232853	zinc finger protein 954 [Source:MGI Symbol;Acc:MGI:1917764]	Zfp954	protein_coding
ENSMUSG00000062762	2963.41842	-0.3015382	0.10260831	0.00019876	0.02792707	Down	13663	etoposide induced 2.4 mRNA [Source:MGI Symbol;Acc:MGI:108090]	Ei24	protein_coding
ENSMUSG00000063558	1167.49937	-0.600343	0.25260658	0.00056102	0.04816677	Down	11761	aldehyde oxidase 1 [Source:MGI Symbol;Acc:MGI:88035]	Aox1	protein_coding
ENSMUSG00000063704	258.002006	-0.7770085	0.25164128	7.30E-05	0.0161992	Down	332110	mitogen-activated protein kinase 15 [Source:MGI Symbol;Acc:MGI:2652894]	Mapk15	protein_coding
ENSMUSG00000066072	3215.81213	-0.0133434	0.07405136	0.00046363	0.04334747	Down	13117	cytochrome P450, family 4, subfamily a, polypeptide 10 [Source:MGI Symbol;Acc:MGI:88611]	Cyp4a10	protein_coding
ENSMUSG00000067144	1320.39979	0.73843308	0.29027265	0.00035387	0.0389934	Up	108114	solute carrier family 22 (organic anion transporter), member 7 [Source:MGI Symbol;Acc:MGI:1859559]	Slc22a7	protein_coding

ENSMUSG00000067656	24.9158516	-0.0223398	0.07738992	6.88E-05	0.0161992	Down	171405	solute carrier family 22, member 27 [Source:MGI Symbol;Acc:MGI:3042283]	Slc22a27	protein_coding
ENSMUSG00000068220	405.177295	-0.0568507	0.10975651	0.0004972	0.04520323	Down	16852	lectin, galactose binding, soluble 1 [Source:MGI Symbol;Acc:MGI:96777]	Lgals1	protein_coding
ENSMUSG00000068877	8449.89731	2.29760674	0.78658604	0.00010307	0.01886371	Up	20342	selenium binding protein 2 [Source:MGI Symbol;Acc:MGI:104859]	Selenbp2	protein_coding
ENSMUSG00000069255	224.12378	-0.4305199	0.1455253	0.00016457	0.0254345	Down	105352	dual specificity phosphatase 22 [Source:MGI Symbol;Acc:MGI:1915926]	Dusp22	protein_coding
ENSMUSG00000072949	433.637566	-0.0311351	0.08235434	0.00029122	0.03584854	Down	26897	acyl-CoA thioesterase 1 [Source:MGI Symbol;Acc:MGI:1349396]	Acot1	protein_coding
ENSMUSG00000073842	22351.8223	1.19333011	0.38817063	6.54E-05	0.0161992	Up	100041658	major urinary protein 7 [Source:MGI Symbol;Acc:MGI:3709615]	Mup7	protein_coding
ENSMUSG00000075704	558.369297	-0.319328	0.11644352	0.00031397	0.03660133	Down	26462	thioredoxin reductase 2 [Source:MGI Symbol;Acc:MGI:1347023]	Txnrd2	protein_coding
ENSMUSG00000079111	1869.03408	0.33770689	0.09868186	3.71E-05	0.01147188	Up	66913	KDEL (Lys-Asp-Glu-Leu) endoplasmic reticulum protein retention receptor 2 [Source:MGI Symbol;Acc:MGI:1914163]	Kdelr2	protein_coding
ENSMUSG00000079164	103.889478	-0.0534611	0.10547963	9.26E-05	0.01789025	Down	53791	toll-like receptor 5 [Source:MGI Symbol;Acc:MGI:1858171]	Tlr5	protein_coding
ENSMUSG00000084309	38.1094769	1.52118443	0.49755236	7.39E-05	0.0161992	Up	NA	major urinary protein, pseudogene 20 [Source:MGI Symbol;Acc:MGI:3651976]	Mup-ps20	unprocessed_pseudogene
ENSMUSG00000085006	18.9714267	2.03626232	0.66723513	7.57E-05	0.0161992	Up	NA	cDNA sequence BC021767 [Source:MGI Symbol;Acc:MGI:3615512]	BC021767	transcribed_unprocessed_pseudogene
ENSMUSG00000095079	32.9992184	-0.0375469	0.08708197	0.00052987	0.04664861	Down	NA	immunoglobulin heavy constant alpha [Source:MGI Symbol;Acc:MGI:96444]	Igha	IG_C_gene
ENSMUSG00000104348	23.1890093	-1.1698123	0.39359713	0.00010466	0.01890693	Down	NA	predicted gene, 37691 [Source:MGI Symbol;Acc:MGI:5610919]	Gm37691	TEC
ENSMUSG00000106538	9.28531423	-0.0321039	0.08289764	0.00049718	0.04520323	Down	NA	predicted gene, 30301 [Source:MGI Symbol;Acc:MGI:5589460]	Gm30301	lncRNA
ENSMUSG00000112774	128.539717	-1.1245949	0.34370553	3.81E-05	0.01151995	Down	NA	predicted gene, 36041 [Source:MGI Symbol;Acc:MGI:5595200]	Gm36041	lncRNA
