# Supplementary material for: The effect of aldafermin expressing-Escherichia coli Nissle 1917 along with dietary change on visceral adipose tissue in MASLD mouse model
Source: Int J Obes (Lond). 2025 Apr 10;49(7):1334–44. doi: 10.1038/s41366-025-01774-w (PMC12283412; doi:10.1038/s41366-025-01774-w)
Supplement: Supplementary file 7 — Supplementary table 8 [file 41366_2025_1774_MOESM7_ESM.pdf]

EcN vs CTRL liver

ENSEMBL	baseMean	log2FoldChange	lfcSE	pvalue	padj	UD	entrezgene_id	description	external_gene_name	gene_biotype
ENSMUSG00000017718	598.746771	0.487963835	0.12821826	5.13E-06	0.01268642	Up	71562	arylformamidase [Source:MGI Symbol;Acc:MGI:2448704]	Afmid	protein_coding
ENSMUSG00000019944	931.989255	0.573811637	0.16107586	1.34E-05	0.02899242	Up	69288	Rho-related BTB domain containing 1 [Source:MGI Symbol;Acc:MGI:1916538]	Rhobtb1	protein_coding
ENSMUSG00000056978	27.4369354	3.729443469	0.95702349	3.34E-06	0.00962823	Up	66438	hepcidin antimicrobial peptide 2 [Source:MGI Symbol;Acc:MGI:2153530]	Hamp2	protein_coding
ENSMUSG00000058546	924.255375	0.849240028	0.19408943	4.80E-07	0.00207682	Up	268449	ribosomal protein L23A [Source:MGI Symbol;Acc:MGI:3040672]	Rpl23a	protein_coding
ENSMUSG00000060143	128.2365	1.995083976	0.57131642	1.63E-05	0.03138616	Up	NA	predicted gene 10076 [Source:MGI Symbol;Acc:MGI:3704451]	Gm10076	lncRNA
ENSMUSG00000064372	896.08151	1.214180924	0.24687475	3.52E-08	0.00027355	Up	NA	mitochondrially encoded tRNA proline [Source:MGI Symbol;Acc:MGI:102478]	mt-Tp	Mt_tRNA
ENSMUSG00000086165	15.3021581	1.828452568	0.43457145	1.13E-06	0.00389948	Up	NA	predicted gene 15690 [Source:MGI Symbol;Acc:MGI:3783131]	Gm15690	lncRNA
ENSMUSG00000091803	77.3456761	1.690689818	0.34767419	4.74E-08	0.00027355	Up	66272	cytochrome c oxidase assembly protein 16 [Source:MGI Symbol;Acc:MGI:1913522]	Cox16	protein_coding
