# Supplementary material for: The effect of aldafermin expressing-Escherichia coli Nissle 1917 along with dietary change on visceral adipose tissue in MASLD mouse model
Source: Int J Obes (Lond). 2025 Apr 10;49(7):1334–44. doi: 10.1038/s41366-025-01774-w (PMC12283412; doi:10.1038/s41366-025-01774-w)
Supplement: Supplementary file 8 — Supplementary table 9 [file 41366_2025_1774_MOESM8_ESM.pdf]

Enriched pathways

EcNA vs EcN eVAT

Gene Set	Description	Size	Leading Edge Number	ES	NES	P Value	FDR	gene symbol
mmu00190	Oxidative phosphorylation	21	12	057943	25184	<2.2e-16	<2.2e-16	Atp6v0d2<br>ND6<br>ND4L<br>ND5<br>ATP6<br>ND2<br>CYTB<br>ND4<br>COX1<br>ND3
mmu05012	Parkinson disease	21	11	056938	2439	<2.2e-16	00014234	ND6<br>ND4L<br>ND5<br>ATP6<br>ND2<br>CYTB<br>ND4<br>COX1<br>ND3<br>ND1
mmu01212	Fatty acid metabolism	8	7	080919	23671	<2.2e-16	00014234	Elovl6<br>Acaca<br>Ehhadh<br>Acsl1<br>Acat2<br>Hsd17b12<br>Cpt2
mmu00620	Pyruvate metabolism	10	8	071929	23061	<2.2e-16	00021351	Acaca<br>Acacb<br>Me1<br>Pdhb<br>Pdha1<br>Pcx<br>Acat2<br>Dlat
mmu04120	Ubiquitin mediated proteolysis	13	11	058452	20847	<2.2e-16	0018301	Cul4b<br>Xiap<br>Cdc27<br>Traf6<br>Cul1<br>Ube3a<br>Cul2<br>Cul5<br>Ube4a<br>Ube2q2
mmu04146	Peroxisome	12	10	062054	20998	<2.2e-16	0018504	Ehhadh<br>Acsl1<br>Pex3<br>Nudt12<br>Pex11a<br>Abcd2<br>Pex1<br>Pex13<br>Abcd3<br>Nudt7
mmu04714	Thermogenesis	30	19	044276	21074	<2.2e-16	0019927	ND6<br>ND4L<br>ND5<br>ATP6<br>ND2<br>CYTB<br>ND4<br>COX1<br>ND3<br>Acsl1

mmu01100	Metabolic pathways	145	38	027831	2039	<2.2e-16	0023486	Pah<br>Cyp2c70<br>Elovl6<br>St6galnac5<br>Atp6v0d2<br>Acly<br>ND6<br>B3galt2<br>ND4L<br>Acaca
mmu00640	Propanoate metabolism	10	7	06253	20284	<2.2e-16	0023565	Acaca<br>Acacb<br>Ehhadh<br>Acat2<br>Acss3<br>Aldh6a1<br>Dbt
mmu00061	Fatty acid biosynthesis	4	3	08551	19253	00026316	0048964	Acaca<br>Acacb<br>Acsl1
mmu05322	Systemic lupus erythematosus	9	7	-073336	-18405	<2.2e-16	012335	H2-DMb2<br>H2afj<br>Hist2h3c2<br>C1qa<br>Hist1h4h<br>Cd40<br>C4b
mmu03010	Ribosome	30	28	-054135	-18801	00011806	015136	Rpl35<br>Rpl22l1<br>Rps23<br>Rps16<br>Rpl13<br>Rpl23<br>LOC100044627<br>LOC100862455<br>Rps15<br>Rps9
mmu05217	Basal cell carcinoma	8	5	-068158	-1659	0008427	065896	Wnt2<br>Gli1<br>Cdkn1a<br>Wnt5b<br>Gadd45a
mmu00310	Lysine degradation	5	2	-058904	-12451	020382	072218	Ogdhl<br>Aass
mmu04960	Aldosterone-regulated sodium reabsorption	8	2	-051065	-12474	020584	073095	Atp1a4<br>Fxyd2
mmu04918	Thyroid hormone synthesis	11	3	-04629	-12517	019257	073336	Atp1a4<br>Fxyd2<br>Lrp2
mmu05150	Staphylococcus aureus infection	4	3	-064742	-12597	018297	074057	H2-DMb2<br>C1qa<br>C4b
mmu04976	Bile secretion	6	4	-057287	-12688	017775	07462	Atp1a4<br>Fxyd2<br>Atp1a1<br>Ephx1
mmu04971	Gastric acid secretion	10	4	-047675	-1252	020217	074927	Atp1a4<br>Calml4<br>Kcnq1<br>Atp1a1
mmu04722	Neurotrophin signaling pathway	15	3	-042784	-12615	019255	075298	Mapk13<br>Calml4<br>Bex3

EcNA vs CTRL eVAT

Gene Set	Description	Size	Leading Edge Number	ES	NES	P Value	FDR	gene symbol

mmu03010	Ribosome	60	49	-048939	-22509	<2.2e-16	00092669	Rpl35<br>Rpl22l1<br>Rplp1<br>Mrpl14<br>Rps16<br>Mrps12<br>Rps15<br>Rps23<br>Rpl13<br>Rpl32
mmu05230	Central carbon metabolism in cancer	11	7	054328	20478	00035336	0054868	Pdk1<br>Ntrk3<br>Pdhb<br>Pdha1<br>Pik3cb<br>Akt3 Pten
mmu04922	Glucagon signaling pathway	21	9	043015	21325	<2.2e-16	0056061	Acaca<br>Acacb<br>Ppara<br>Prkaa2<br>Plcb4<br>Pdhb<br>Pdha1<br>Sirt1<br>Ppp4r3a
mmu04713	Circadian entrainment	16	5	045319	20122	00087719	0056299	Adcy10<br>Per3<br>Per2<br>Cacna1c<br>Plcb4
mmu00061	Fatty acid biosynthesis	6	4	074822	20758	00030488	0058446	Acaca<br>Fasn<br>Acacb<br>Acsl1
mmu04060	Cytokine-cytokine receptor interaction	21	9	-054353	-19202	00012755	0080437	Mstn<br>Il13ra2<br>Bmp3<br>Lep<br>Crlf2<br>Tnfrsf12a<br>Il3ra<br>Ccl24<br>Il17d
mmu03018	RNA degradation	21	16	038154	19142	00085837	0080939	Patl1<br>Pan3<br>Tent4b<br>Pan2<br>Cnot6<br>Dhx36<br>Pnpt1<br>Tob2<br>Dis3<br>Cnot6l
mmu00020	Citrate cycle (TCA cycle)	6	5	067454	19318	00092879	00827	Acly<br>Pdhb<br>Pdha1<br>Cs<br>Dlat
mmu04070	Phosphatidylinositol signaling system	13	9	047255	18527	0015094	0083137	Plcb4<br>Inpp4b<br>Pik3cb<br>Inpp1<br>Dgka<br>Dgke<br>Ippk<br>Pten<br>Mtmr1
mmu04310	Wnt signaling pathway	15	5	-059004	-19399	00026525	008456	Sfrp5<br>Wnt2<br>Sfrp4<br>Nkd1<br>Serpinf1
mmu00640	Propanoate metabolism	6	3	065201	18563	0011494	0090784	Acaca<br>Acss2<br>Acacb

mmu04710	Circadian rhythm	7	4	061127	18639	0012539	0095423	Per3<br>Per2<br>Rorc<br>Prkaa2
mmu04974	Protein digestion and absorption	10	6	-066897	-19548	00083799	0098538	Atp1a4<br>Col12a1<br>Col6a1<br>Fxyd2<br>Col6a2<br>Col18a1
mmu00620	Pyruvate metabolism	8	6	067046	2145	<2.2e-16	010258	Atp1a4<br>Col12a1<br>Col6a1<br>Fxyd2<br>Col6a2<br>Col18a1
mmu05219	Bladder cancer	12	5	-060204	-18257	00069061	011254	Cdkn2a<br>Thbs1<br>Cdkn1a<br>E2f1<br>Dapk3
mmu05217	Basal cell carcinoma	9	7	-066352	-18335	00057887	011908	Wnt2<br>Gadd45a<br>Cdkn1a<br>Gadd45b<br>Bax<br>Fzd2<br>Wnt11
mmu05016	Huntington disease	45	28	-041923	-18527	00056243	013082	Uqcr11<br>Ndufa2<br>Atp5e<br>Cltb<br>Ndufc2<br>Bax<br>Polr2l<br>Ndufa11<br>Polr2f<br>Cox5b
mmu00590	Arachidonic acid metabolism	7	5	-072705	-18356	00077042	013437	Pla2g2e<br>Cyp4f14<br>Ltc4s<br>Fam213b<br>Gpx7
mmu04115	p53 signaling pathway	15	8	-060845	-19623	<2.2e-16	0139	Cdkn2a<br>Thbs1<br>Gtse1<br>Gadd45a<br>Cdkn1a<br>Gadd45b<br>Bax<br>Bbc3
mmu05322	Systemic lupus erythematosus	20	16	-049293	-17686	0013784	016745	Hist2h3c2<br>H2afj<br>Hist1h4h<br>Hist1h4c<br>C1qa<br>H2afz<br>H2-DMb1<br>H2-Ab1<br>H2-DMa<br>H3f3a

EcN vs CTRL eVAT

Gene Set	Description	Size	Leading Edge Number	ES	NES	P Value	FDR	gene symbol
mmu00350	Tyrosine metabolism	4	2	-094732	-20543	<2.2e-16	00053327	Adh4<br>Fah

mmu04610	Complement and coagulation cascades	11	11	-057919	-1823	<2.2e-16	0037329	C1s2<br>Vsig4<br>Vtn<br>Serpina1a<br>Serpina1b<br>Serpina1c<br>Serpinc1<br>Serpina1d<br>Serpina1e<br>F9
mmu04979	Cholesterol metabolism	3	1	-0936	-1774	00082645	0050661	Angptl4
mmu00360	Phenylalanine metabolism	3	3	-089778	-16906	0015748	0084657	Hpd<br>Tat<br>Pah
mmu04810	Regulation of actin cytoskeleton	3	3	-069778	-13317	013656	035367	Fgfr3<br>Enah<br>Itgb2l
mmu05165	Human papillomavirus infection	11	11	-041176	-13605	<2.2e-16	035885	Wnt4<br>Spp1<br>Chad<br>Col4a3<br>Col4a4<br>Lamc2<br>Fzd6<br>H2-Q10<br>Patj<br>Hdac11
mmu04974	Protein digestion and absorption	6	6	-057718	-13698	010101	040929	Col4a3<br>Kcnk5<br>Col4a4<br>Kcnq1<br>Atp1a1<br>Slc7a9
mmu04713	Circadian entrainment	6	3	055795	11287	027772	059009	Calml3<br>Adcy8<br>Cacna1h
mmu04218	Cellular senescence	4	1	061701	11183	029036	059039	Calml3
mmu05167	Kaposi sarcoma-associated herpesvirus infection	4	1	061701	11183	029036	059039	Calml3
mmu04750	Inflammatory mediator regulation of TRP channels	7	2	054492	11382	029335	05935	Calml3<br>Adcy8
mmu04923	Regulation of lipolysis in adipocytes	3	1	066711	11322	028553	059436	Adcy8
mmu05152	Tuberculosis	5	1	057218	11114	031488	059545	Calml3
mmu04976	Bile secretion	4	1	062476	11409	027784	060148	Adcy8
mmu05215	Prostate cancer	4	2	059714	11026	033577	060543	Tmprss2<br>Spint1
mmu05133	Pertussis	5	1	05889	11414	027752	061563	Calml3
mmu05170	Human immunodeficiency virus 1 infection	6	1	057192	11509	026897	062168	Calml3
mmu04510	Focal adhesion	7	7	-042152	-11448	022892	065593	Spp1<br>Chad<br>Col4a3<br>Col4a4<br>Lamc2<br>Igf1r<br>Vtn
mmu01521	EGFR tyrosine kinase inhibitor resistance	3	3	-062222	-11583	023729	069676	Erbb3<br>Fgfr3<br>Igf1r
mmu04933	AGE-RAGE signaling pathway in diabetic complications	3	3	-049333	-09276	054615	074918	Mapk13<br>Col4a3<br>Col4a4

EcNA vs EcN liver

Gene Set	Description	Size	Leading Edge Number	ES	NES	P Value	FDR	gene symbol
mmu00071	Fatty acid degradation	3	1	-093989	-17244	00065502	004131	Acaa1b
mmu03320	PPAR signaling pathway	5	3	-079058	-17579	0012407	0041869	Acaa1b<br>Pparg<br>Slc27a1
mmu05204	Chemical carcinogenesis	8	6	-069288	-17804	0012755	0068665	Cyp2c38<br>Mgst3<br>Cyp3a11<br>Gstt3<br>Cyp2c29<br>Ephx1

mmu04146	Peroxisome	6	4	-064786	-15129	0066351	016636	Acaa1b<br>Pex11a<br>Ech1<br>Hmgcl
mmu00982	Drug metabolism	3	3	-084713	-15333	0039216	017216	Mgst3<br>Gstt3<br>Aox1
mmu00830	Retinol metabolism	9	4	-059724	-15409	0036932	020264	Cyp2c38<br>Cyp3a11<br>Aox1<br>Cyp2c29
mmu00980	Metabolism of xenobiotics by cytochrome P450	3	3	-080892	-14478	0077088	022059	Mgst3<br>Gstt3<br>Ephx1
mmu00280	Valine, leucine and isoleucine degradation	3	3	-075159	-13633	011597	031004	Acaa1b<br>Aox1<br>Hmgcl
mmu05225	Hepatocellular carcinoma	3	2	-069165	-12589	022422	03106	Mgst3 Gstt3
mmu01100	Metabolic pathways	33	6	-032687	-13414	0037736	031206	Gal3st1<br>Cyp2c38<br>Csad<br>Acaa1b<br>Cyp3a11<br>Ces1d
mmu04141	Protein processing in endoplasmic reticulum	12	12	058784	14372	0074581	04732	Dnajb11<br>Uggt1<br>Dnajc3<br>Sec61a1<br>Pdia6<br>Edem2<br>Ddost<br>Stt3a<br>Ssr3<br>Tram1
mmu00240	Pyrimidine metabolism	3	1	03174	05243	095489	096531	Cad
mmu00983	Drug metabolism	5	1	053392	10378	043333	1	Ces2b
mmu00510	N-Glycan biosynthesis	3	3	057962	09792	051471	1	Ddost<br>Stt3a<br>Rpn2
mmu03060	Protein export	3	3	052866	08885	065357	1	Sec61a1<br>Srpr<br>Srp72
mmu00270	Cysteine and methionine metabolism	3	2	045882	07782	078269	1	Ahcyl2 Srm
mmu00380	Tryptophan metabolism	3	1	039632	06636	084737	1	Acmsd
mmu04976	Bile secretion	4	2	033987	06193	087993	1	Abcb1b Slc22a7

EcNA vs CTRL liver

Gene Set	Description	Size	Leading Edge Number	ES	NES	P Value	FDR	gene symbol
mmu00590	Arachidonic acid metabolism	3	1	-091341	-16505	00041667	035597	Pla2g12a
mmu04062	Chemokine signaling pathway	3	3	-070621	-12806	01879	059318	Ccl27a<br>Ccl27b<br>Mapk3
mmu00071	Fatty acid degradation	3	1	-071029	-13003	017021	061943	Acadm
mmu04360	Axon guidance	3	1	-065618	-12155	023789	06207	Sema5b
mmu05225	Hepatocellular carcinoma	5	1	-047314	-10486	039355	067152	Mgst3
mmu00830	Retinol metabolism	5	1	-060284	-13124	019403	067908	Hsd17b6
mmu05216	Thyroid cancer	3	1	-067764	-12192	019583	067991	Pparg
mmu00480	Glutathione metabolism	3	2	-081842	-14924	0058091	068604	Mgst3<br>Gclc
mmu00980	Metabolism of xenobiotics by cytochrome P450	3	1	-057666	-10495	043662	071551	Mgst3
mmu00982	Drug metabolism	3	1	-057666	-10495	043662	071551	Mgst3
mmu04210	Apoptosis	5	2	024344	04948	098911	098039	Itpr1<br>Csf2rb
mmu05210	Colorectal cancer	3	3	030508	05244	097575	099357	Smad3<br>Mapk3<br>Gadd45b
mmu05212	Pancreatic cancer	3	3	030508	05244	097575	099357	Smad3<br>Mapk3<br>Gadd45b
mmu05220	Chronic myeloid leukemia	3	3	030508	05244	097575	099357	Smad3<br>Mapk3<br>Gadd45b
mmu05226	Gastric cancer	3	3	030508	05244	097575	099357	Smad3<br>Mapk3<br>Gadd45b

mmu04640	Hematopoietic cell lineage	3	3	088701	15413	00194	1	Cd4<br>Csf1<br>Il6ra
mmu03010	Ribosome	6	5	068929	14438	0096948	1	Rps29<br>Rpl23a<br>Rps28<br>Rps27<br>Rpl39
mmu04750	Inflammatory mediator regulation of TRP channels	3	1	073951	12841	018252	1	Itpr1
mmu05169	Epstein-Barr virus infection	3	1	072978	12532	022202	1	Oas1g
mmu04120	Ubiquitin mediated proteolysis	3	3	068362	11795	029423	1	Brca1<br>Rhobtb1<br>Herc2
